# Differences in thermal expansion and motion ability for herringbone and face-to-face π-stacked solids

**DOI:** 10.1107/S2052252521009593

**Published:** 2021-11-03

**Authors:** Xiaodan Ding, Ethan Zahid, Daniel K. Unruh, Kristin M. Hutchins

**Affiliations:** a Department of Chemistry and Biochemistry, Texas Tech University, Lubbock, TX 79409, USA

**Keywords:** thermal expansion, pedal motion, halogen bonding, π-stacking, crystal engineering, intermolecular interactions, properties of solids

## Abstract

A series of aromatic organic molecules functionalized with different halogen atoms (I/ Br), motion-capable groups (olefin, azo or imine) and molecular length were designed and synthesized. The molecules self-assemble in the solid state through halogen bonding and exhibit molecular packing sustained by either herringbone or face-to-face π-stacking, two common motifs in organic semiconductor molecules. Interestingly, dynamic pedal motion is only achieved in solids with herringbone packing. On average, solids with herringbone packing exhibit larger thermal expansion within the halogen-bonded sheets due to motion occurrence and molecular twisting, whereas molecules with face-to-face π-stacking do not undergo motion or twisting. Thermal expansion along the π-stacked direction is surprisingly similar, but slightly larger for the face-to-face π-stacked solids due to larger changes in π-stacking distances with temperature changes. The results speak to the importance of crystal packing and intermolecular interaction strength when designing aromatic-based solids for organic electronics applications.

## Abstract 2

A series of halogenated compounds with motion-capable moieties were designed and synthesized, and they exhibit different π-stacking arrangements. The thermal expansion behaviors are influenced by crystal packing, halogen-bond strength and pedal motion ability.

## 1. Introduction

Thermal expansion (TE) is a fundamental property of materials, and it describes the response to temperature change (Yao *et al.*, 2019 ▸; Saha, 2017 ▸). An increase in the size or dimension of a material upon heating is positive thermal expansion (PTE) (Das *et al.*, 2010 ▸; Bhattacharya & Saha, 2014 ▸). Negative thermal expansion (NTE) or zero thermal expansion (ZTE) are rarer behaviors, which correspond to a decrease or nearly no change in size upon heating, respectively (Chen *et al.*, 2015 ▸; Wu *et al.*, 2016 ▸ *a*; Zhu *et al.*, 2018 ▸). TE is capable of controlling phase transitions and mechanical properties of organic crystals and has potential applications in thermally responsive materials (Naumov *et al.*, 2015 ▸; Reddy *et al.*, 2010 ▸). Moreover, understanding TE in materials such as organic semiconductors is important because it influences bandwidth narrowing (Li *et al.*, 2012 ▸; van der Lee *et al.*, 2018 ▸) and performance can be reduced if TE properties in a device are mismatched (Mei *et al.*, 2017 ▸; Wu *et al.*, 2016 ▸ *b*).

**Figure 1:**
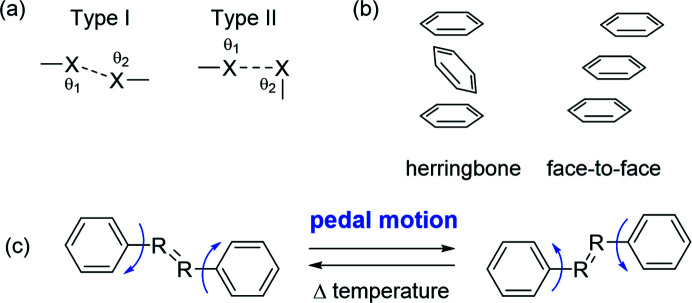
Geometries of (*a*) type-I and type-II halogen⋯halogen interactions and (*b*) herringbone and offset face-to-face π-stacking. (*c*) Pedal motion in a di­phenyl molecule with a motion-capable group.

One aspect that affects the TE of a solid material is the strength of the interactions holding it together. Stronger interactions are less affected by temperature and lead to smaller expansion when compared with weaker interactions (Saraswatula *et al.*, 2018 ▸). Halogen bonding is quickly becoming a widely utilized interaction in materials science (Cavallo *et al.*, 2016 ▸; Saccone & Catalano, 2019 ▸). Recently, halogen bonds have been used to control solid-state packing in organic semiconductor-based materials and improve device performance (Wilson *et al.*, 2015 ▸; Weldeab *et al.*, 2018 ▸; Zhang & Wang, 2021 ▸; Li *et al.*, 2018 ▸). Halogen⋯halogen interactions are a subset of halogen bonds and can be classified into two distinct types based on the angles (θ) between the halogens [C—X⋯X, Fig. 1 ▸(*a*)] (Mukherjee *et al.*, 2014 ▸). Saraswatula and Saha have studied the TE behavior of halogen⋯halogen interactions within a series of isostructural organic molecules (Saraswatula & Saha, 2014 ▸). The TE responses of the solids correlated with the strength of the inter-halogen interactions, and the trend was I⋯I < Br⋯Br < Cl⋯Cl (where < refers to a smaller distance change). However, bond strength is not the only parameter that influences TE.

A continued interest in our group is to investigate the effect of molecular pedal motion (Harada & Ogawa, 2009 ▸) on TE behaviors [Fig. 1 ▸(*c*)]. Recently, our group demonstrated that dynamic pedal motion leads to large PTE in organic solids (Hutchins *et al.*, 2018 ▸; Juneja *et al.*, 2019 ▸; Ding *et al.*, 2020 ▸). The most widely studied motion-capable moieties are olefin (C=C) and azo (N=N) groups (Harada & Ogawa, 2001 ▸; Peedikakkal, 2017 ▸). Imine (C=N) groups are also capable of pedal motion, and though imines have been well studied in Schiff base chemistry (Kiefer *et al.*, 2019 ▸; Hadjoudis *et al.*, 2004 ▸, 2011 ▸; Hadjoudis & Mavridis, 2004 ▸), pedal motion of imines has been investigated to a lesser extent (Harada *et al.*, 2004*a* ▸,*b* ▸). To date, our group has only investigated TE properties for motion-capable moieties substituted with pyridine rings (Juneja *et al.*, 2019 ▸; Ding *et al.*, 2020 ▸).

**Figure 2:**
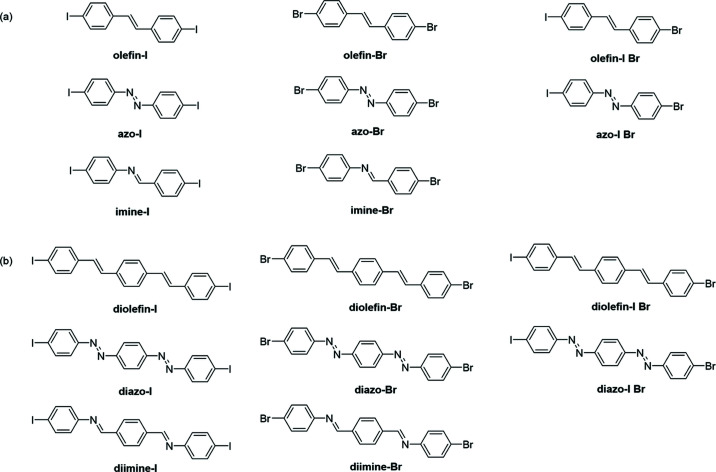
Structures and abbreviations of halogenated molecules in this work with (*a*) one motion group and (*b*) two motion groups. The compound abbreviations are shown in bold.

Here, we designed and synthesized a series of aromatic, di-halogenated molecules functionalized with one or two olefin, azo and imine groups [Figs. 2 ▸(*a*) and 2 ▸(*b*)]. The compounds outlined in Fig. 2 ▸ were chosen to systematically tune three features and investigate the impact on TE: motion group identity, number of motion groups and halogen-bond strength. We demonstrate that the molecules crystallize into packing arrangements analogous to those frequently observed in organic semiconductor materials, herringbone or face-to-face π-stacked [Fig. 1 ▸(*b*)]. We show that herringbone packing supports solid-state pedal motion, yet face-to-face stacking does not. Furthermore, the herringbone and face-to-face π-stacked solids undergo different degrees of TE along two directions. Surprisingly, the TE along the π-stacked direction is similar even though the interactions and packing along the direction are clearly different. Overall, we demonstrate the comprehensive influence of crystal packing motion and noncovalent interaction strength on TE behaviors in π-stacked solids.

## 2. Experimental

The 16 compounds in Figs. 2 ▸(*a*) and 2 ▸(*b*) were synthesized using literature or modified-literature procedures and characterized by variable-temperature single-crystal X-ray diffraction (VT SCXRD) and ^1^H NMR spectroscopy (pages S2–S9, Tables S1–S34 and Figs. S18–S36 of the supporting information). The diolefin compounds were not soluble in common deuterated solvents, so powder X-ray diffraction was used to characterize these three compounds in place of NMR (Figs. S37–S39). VT SCXRD studies were performed in the range 290–190 K, and full crystallographic datasets were collected at 20 K intervals. Each crystal was mounted at 190 K and warmed to collect additional datasets. The morphology and color of each crystal was characterized using a Leica DM2700M microscope equipped with a camera. TE coefficients were calculated using the software *PASCal* (Cliffe & Goodwin, 2012 ▸).

A search of the Cambridge Structural Database (CSD; version 5.42 + 2 updates) demonstrated that the single-crystal structures of imine-Br (Bernstein & Izak, 1975 ▸; Marin *et al.*, 2013 ▸; Ashokkumar *et al.*, 2021 ▸), azo-I (Grebel-Koehler *et al.*, 2003 ▸) and azo-Br (Amit & Hope, 1966 ▸; Howard *et al.*, 1994 ▸; Karanam & Choudhury, 2013 ▸) have been previously published at one temperature. For imine-Br and azo-I, we obtained crystals that are identical to the published structures (Marin *et al.*, 2013 ▸; Grebel-Koehler *et al.*, 2003 ▸) and conducted VT SCXRD studies here. Azo-Br has been reported to crystallize as two polymorphs (a and b) (Amit & Hope, 1966 ▸; Howard *et al.*, 1994 ▸; Karanam & Choudhury, 2013 ▸), and we conducted VT SCXRD experiments for both. The polymorph azo-Br(a) crystallizes in the face-to-face packing arrangement (isostructural to others) and is discussed below. The polymorph azo-Br(b) crystallizes in an arrangement that differs from the other 16 solids discussed here; thus, it is not a focus of this work and full details are given in the supporting information (pages S86–S87).

## 3. Results and discussion

We incorporated three different motion groups to systematically investigate if one motion group undergoes dynamic motion more readily than another, if the number of motion groups impacts motion ability, and to study the influence of motion on TE. In total, 12 of the 16 halogenated molecules are symmetrical (olefin-I, olefin-Br, imine-I, imine-Br, azo-I, azo-Br, diolefin-I, diolefin-Br, di­imine-I, di­imine-Br, diazo-I and diazo-Br) and four are unsymmetrical (olefin-I Br, azo-I Br, diolefin-I Br and diazo-I Br). The two unsymmetrical imine compounds are not included in this manuscript. The unsymmetrical nature of the single-imine molecule was not distinguishable by X-ray diffraction due to an inversion center in the imine, and the iodine and bromine occupying the same crystallographic site. The synthesis of the unsymmetrical double-imine molecule was unsuccessful due to competing reactions that afford the symmetrical products.

In the solid state, the halogenated molecules are expected to assemble into an extended structure through *X*⋯*X* forces. The symmetrical compounds were chosen to include two iodo or two bromo groups. We expected the stronger I⋯I interactions to be less affected by temperature changes, while Br⋯Br interactions are weaker and should be more affected. The unsymmetrical donor molecules feature one iodo and one bromo group and offer a subtle interplay between the two symmetrical systems.

**Figure 3:**
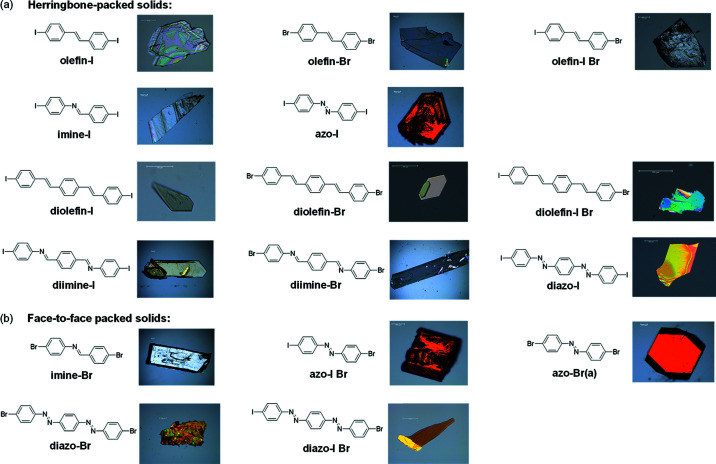
Images of each crystal with the corresponding molecular structure for (*a*) herringbone and (*b*) face-to-face π-stacked arrangements.

Single-crystal analysis revealed the three single olefin compounds (olefin-I, olefin-Br and olefin-I Br), imine-I, azo-I (Grebel-Koehler *et al.*, 2003 ▸) and three diolefin compounds (diolefin-I, diolefin-Br and diolefin-I Br) to crystallize in the orthorhombic space group *Pccn*. Imine-Br (Marin *et al.*, 2013 ▸), azo-Br(a) (Amit & Hope, 1966 ▸), azo-I Br, the two di­imine compounds (di­imine-I and di­imine-Br) and diazo-I crystallized in the monoclinic space group *P*2_1_/*c*. The other two diazo compounds diazo-Br and diazo-I Br crystallized in the monoclinic space group *P*2_1_/*n*. The asymmetric units of di­imine-I, di­imine-Br and diazo-I contain one-half of two unique molecules, while in the other 13 crystals, the asymmetric units contain one-half of the molecule. In the unsymmetrical iodo-bromo compounds, olefin-I Br, azo-I Br, diolefin-I Br and diazo-I Br, the iodine and bromine atoms sit at nearly identical crystallographic positions. The site occupancy for each atom was constrained at 0.5 with SIMU restraints being used primarily to maintain reasonable ADP values for the two atoms. For the single imine compounds, imine-I and imine-Br, an inversion center lies at the center of the bridge group; thus, the carbon and nitro­gen atoms occupy the same crystallographic space. The halogen⋯halogen interaction distances and angles for each solid are shown in Table S36. Of the 16 solids, 11 self-assembled into herringbone packed structures and five into face-to-face π-stacked structures. The morphology and color of each crystal are shown below (Fig. 3 ▸).

### 3.1. Molecular motion

**Figure 4:**
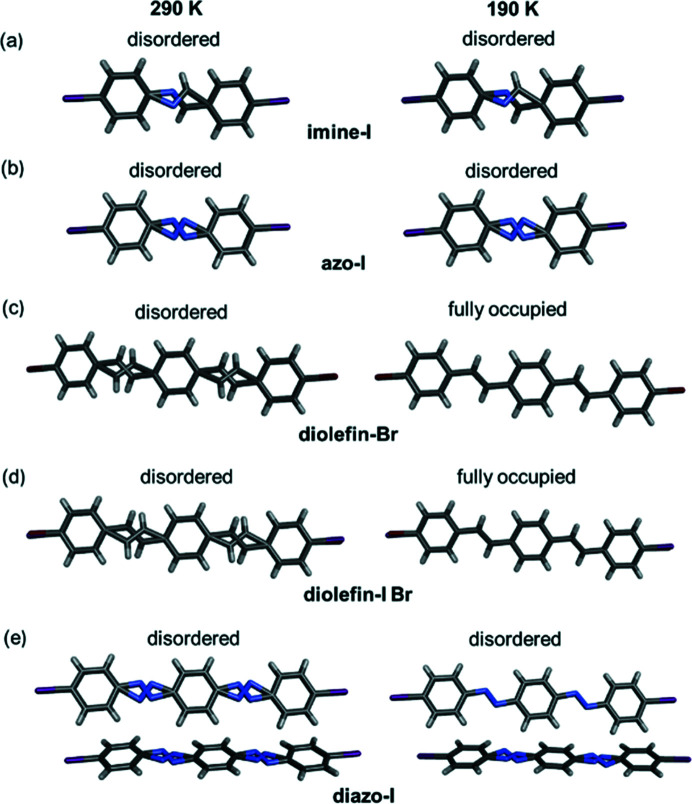
X-ray crystal structures at 290 and 190 K highlighting unresolved or resolved disorder within (*a*) imine-I, (*b*) azo-I, (*c*) diolefin-Br, (*d*) diolefin-I Br and (*e*) diazo-I. Disorder is only shown for the bridge groups for clarity.

Motion was characterized using VT SCXRD and observed through disorder of the motion-capable molecule. If disorder is present, the site occupancies of the major and minor sites are quantified. If the site occupancies change with temperature, the motion is dynamic, and if the occupancies remain nearly constant, the disorder is static (Harada & Ogawa, 2001 ▸, 2009 ▸). The molecules imine-I, azo-I, diolefin-Br, diolefin-I Br and diazo-I exhibited disorder during the variable-temperature experiments. The site occupancies of each orientation were allowed to freely refine in the five compounds, and the sum of the two site occupancies was set to a total of one. VT SCXRD experiments demonstrated that all five compounds undergo dynamic pedal motion as confirmed by changes in the site occupancies as a function of temperature (Table 1 ▸). The overall change in the site occupancies between 190 and 290 K ranged from 3 to 27% for the five solids. Imine-I and azo-I undergo dynamic pedal motion over the entire temperature range studied [190–290 K, Figs. 4 ▸(*a*) and 4 ▸(*b*)]. The disorder resolves at 250 K in diolefin-Br and 230 K in diolefin-I Br [Figs. 4 ▸(*c*) and 4 ▸(*d*)]. Diazo-I includes two crystallographically unique molecules. Disorder in one of the two molecules resolves at 250 K, and the second molecule exhibits pedal motion over the entire temperature range [Fig. 4 ▸(*e*)]. There was no evidence of pedal motion in the other 11 structures.

### 3.2. Compounds with herringbone crystal packing

In total, 11 of the 16 solids crystallize with the extended packing sustained by herringbone π-stacking. The molecules described below self-assemble into 2D sheets through type-II halogen⋯halogen forces. The sheets assemble into herringbone-packed layers that interact through C—H⋯π interactions, type-I halogen⋯halogen bonds and C—H⋯*X* forces.

**Figure 5:**
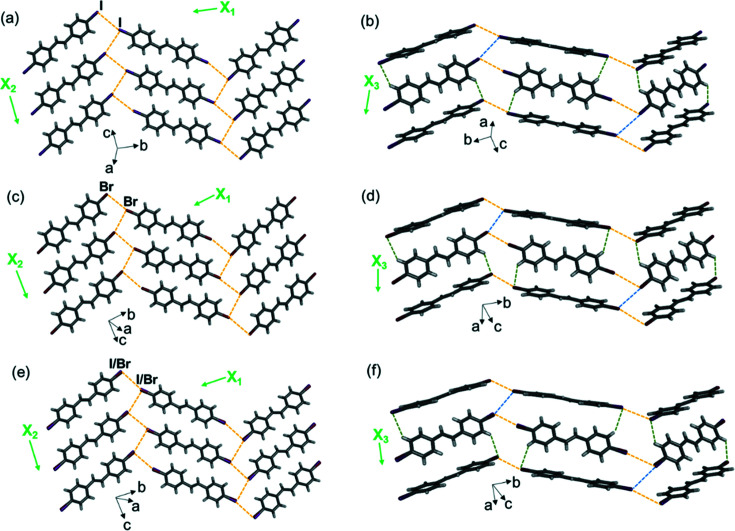
Single-crystal X-ray structures showing 2D halogen-bonded sheets, layers and TE axes for (*a*) and (*b*) olefin-I, (*c*) and (*d*) olefin-Br, and (*e*) and (*f*) olefin-I Br. Type-II halogen bonds are shown with yellow dashed lines, type-I halogen bonds are shown with blue dashed lines and C—H⋯*X* forces are shown with green dashed lines. Structures are shown at 290 K for olefin-Br and olefin-I Br, and 270 K for olefin-I (due to poor data quality at 290 K).

The three single-olefin compounds, olefin-I, olefin-Br and olefin-I Br, are isostructural (IUCr Online Dictionary of Crystallography, 2019 ▸) and do not exhibit pedal motion. The molecules self-assemble into 2D sheets, which extend in the *bc* plane [Figs. 5 ▸(*a*), 5 ▸(*c*) and 5(*e*)]. The sheets further assemble into layers that stack along the crystallographic *a* axis [Figs. 5 ▸(*b*), 5 ▸(*d*) and 5(*f*)].

**Figure 6:**
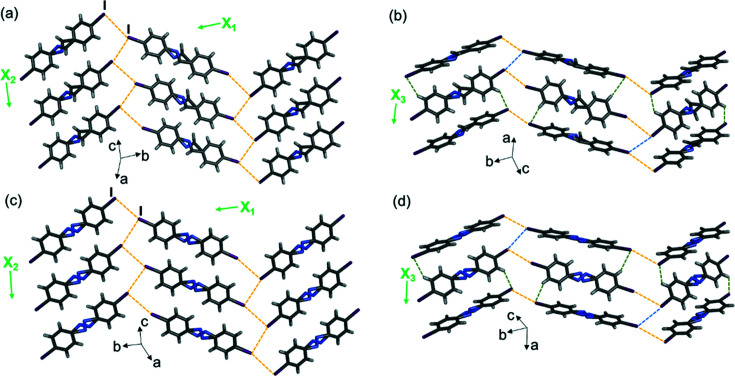
Single-crystal X-ray structures at 290 K showing 2D halogen-bonded sheets, layers and TE axes for (*a*) and (*b*) imine-I, and (*c*) and (*d*) azo-I. Disorder in the aromatic rings and halogens has been omitted for clarity. Type-II halogen bonds are shown with yellow dashed lines, type-I halogen bonds are shown with blue dashed lines and C—H⋯I forces are shown with green dashed lines.

Unlike the single motion group olefin series, the single imine and azo compounds are not isostructural within a series. Two of the molecules that undergo dynamic pedal motion, imine-I and azo-I, crystallize in the same space group as the three single-olefin molecules and exhibit crystal packing that is isostructural to the olefins (Fig. 6 ▸).

**Figure 7:**
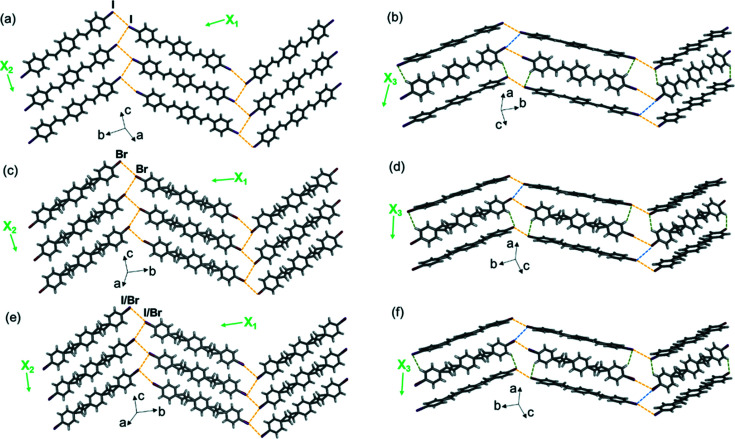
Single-crystal X-ray structures at 290 K showing 2D halogen-bonded sheets, layers and TE axes for (*a*) and (*b*) diolefin-I, (*c*) and (*d*) diolefin-Br, and (*e*) and (*f*) diolefin-I Br. Type-II halogen⋯halogen bonds are shown with yellow dashed lines, type-I bonds are shown with blue dashed lines and C—H⋯*X* forces are shown with green dashed lines.

The three diolefin molecules diolefin-I, diolefin-Br and diolefin-I Br crystallize in the same space group as the molecules above and exhibit isostructural crystal packing. The 2D sheets extend in the *bc* plane [Figs. 7 ▸(*a*), 7 ▸(*c*) and 7(*e*)] and further stack into herringbone-packed layers along the crystallographic *a* axis [Figs. 7 ▸(*b*), 7 ▸(*d*) and 7(*f*)].

**Figure 8:**
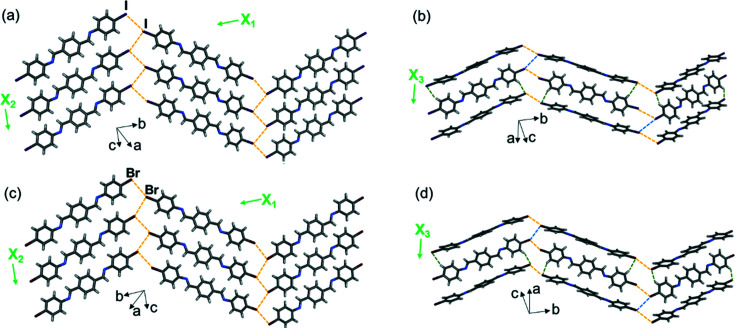
Single-crystal X-ray structures at 290 K, highlighting the 2D halogen-bonded sheets, layers and TE axes for (*a*) and (*b*) di­imine-I, and (*c*) and (*d*) di­imine-Br. Type-II halogen⋯halogen bonds are shown with yellow dashed lines, type-I bonds are shown with blue dashed lines and C—H⋯*X* bonds are shown with green dashed lines.

The two di­imine compounds di­imine-I and di­imine-Br crystallize in a different space group than all the molecules above; however, the crystal packing is still isostructural. The 2D halogen-bonded sheets self-assemble in the *bc* plane [Figs. 8 ▸(*a*) and 8 ▸(*c*)] and then stack into herringbone-packed layers along the crystallographic *a* axis [Figs. 8 ▸(*b*) and 8 ▸(*d*)].

**Figure 9:**
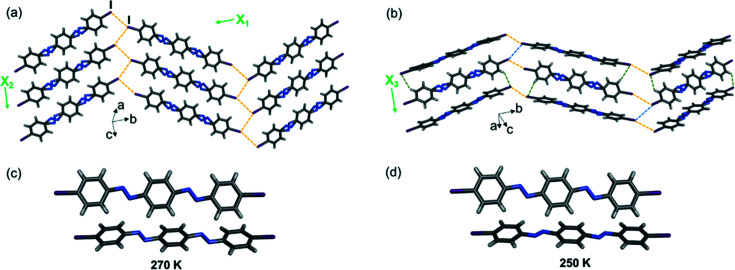
(*a*) and (*b*) Single-crystal X-ray structures at 290 K showing 2D halogen-bonded sheets, herringbone packing and TE axes for diazo-I. Disorder in the aromatic rings has been omitted for clarity. Type-II halogen⋯halogen bonds are shown with yellow dashed lines, type-I bonds are shown with blue dashed lines and C—H⋯*X* bonds are shown with green dashed lines. (*c*) and (*d*) Conformational switch in major sites of diazo-I (bottom molecule) between 270 and 250 K. Only the major sites are shown for both molecules.

The three diazo molecules are not isostructural within a series. The compound diazo-I, which undergoes pedal motion over the entire temperature range, crystallizes in the same space group as the two di­imine molecules and exhibits crystal packing that is isostructural with all the compounds outlined above [Figs. 9 ▸(*a*) and 9 ▸(*b*)]. Diazo-I contains two crystallographically unique molecules and, as outlined in the molecular motion section, one molecule is fully ordered from 190–250 K and exhibits disorder at 270 and 290 K. The second molecule is disordered at all the temperatures studied; however, between 250 and 270 K the positions of major and minor conformations switch [Figs. 9 ▸(*c*) and 9 ▸(*d*)]. The conformational change is accompanied by a 0.13 Å increase in the *b* axis between 250 and 270 K, while the β angle decreases by 0.58° and approaches 90° (Table S29).

### 3.3. Compounds with face-to-face π-stacked crystal packing

Out of the 16 solids, 5 crystallize with the extended packing sustained by face-to-face π-stacking. The five structures described below also self-assemble into 2D sheets through type-II halogen⋯halogen forces, similar to the structures discussed above. However, the sheets in the structures described below assemble into face-to-face π-stacked layers that interact through C—H⋯*X* forces, rather than herringbone-packed layers.

**Figure 10:**
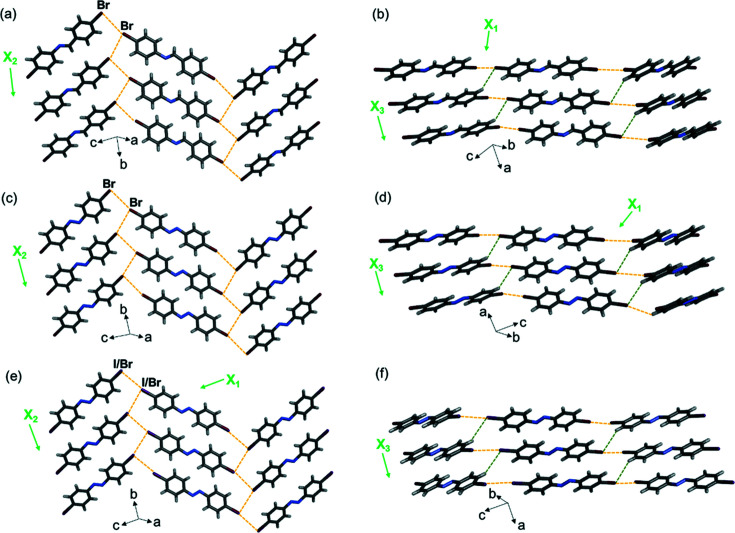
Single-crystal X-ray structures at 290 K highlighting the 2D halogen-bonded sheets, layered packing and TE axes for (*a*) and (*b*) imine-Br, (*c*) and (*d*) azo-Br(a), and (*e*) and (*f*) azo-I Br. Type-II halogen⋯halogen bonds are shown with yellow dashed lines.

The three molecules imine-Br, azo-Br(a) and azo-I Br crystallize in the same space group and exhibit isostructural crystal packing. The 2D sheets extend approximately in the *bc* plane [Figs. 10 ▸(*a*), 10 ▸(*c*) and 10(*e*)] and then pack into face-to-face π-stacked layers along the crystallographic *a* axis [Figs. 10 ▸(*b*), 10 ▸(*d*) and 10(*f*)]. However, the arrangement of the molecules within the 2D sheets differs from the previous structures. Within the sheet, neighboring halogen-bonded molecules are twisted from planarity by ∼55°, whereas in the all the previously described structures, the molecules are much closer to planarity (deviation by 19–32°).

**Figure 11:**
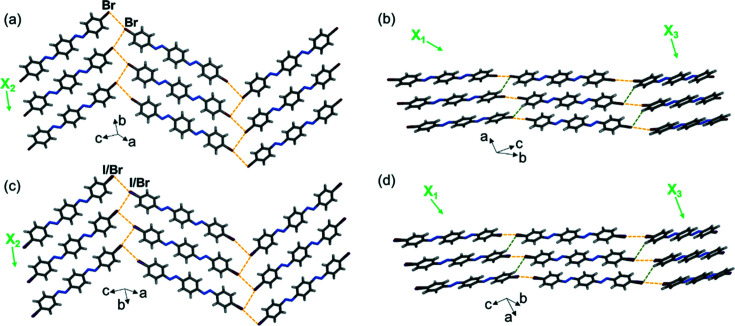
Single-crystal X-ray structures at 290 K highlighting 2D halogen-bonded sheets, layered packing and TE axes for (*a*) and (*b*) diazo-Br, and (*c*) and (*d*) diazo-I Br. Type-II halogen⋯halogen bonds are shown with yellow dashed lines and C—H⋯*X* bonds are shown with green dashed lines.

The two diazo compounds diazo-Br and diazo-I Br crystallize in a different space group than imine-Br, azo-Br(a) and azo-I Br; however, all five solids exhibit isostructural crystal packing. Within the 2D sheets, which extend approximately in the *bc* plane [Figs. 11 ▸(*a*) and 11 ▸(*c*)], the neighboring halogen-bonded molecules are twisted from planarity by ∼54°. The face-to-face layers are further packed along the crystallographic *a* axis [Figs. 11 ▸(*b*) and 11 ▸(*d*)].

### 3.4. Influence of packing and motion group on pedal motion

As highlighted above, 5 of the 16 single-component solids undergo pedal motion and all five compounds exhibit herringbone crystal packing. Thus, solid-state packing has a clear influence on motion ability.

In the single motion group series, zero of three olefin molecules, one of three azo structures and one of two imine molecules undergo pedal motion in the solid state. In the double-motion group series, two of three double-olefin molecules, one of three double-azo molecules and zero of two double-imine molecules undergo pedal motion in the solid state. At first glance, the identity of the motion group does not appear to significantly impact the occurrence of pedal motion in these single-component halogenated solids. However, examination of the five solids that undergo motion suggests that the degree of motion is influenced by identity. The overall change of the site occupancy between 290 and 190 K reveals that azo groups undergo larger changes (azo-I = 20%, diazo-I = 27 and 24%), the imine lies in the middle (imine-I = 9%) and the olefins undergo the smallest overall changes (diolefin-Br = 3%, diolefin-I Br = 7%).

The pedal motion behaviors in four of the five solids (imine-I, azo-I, diolefin-I Br and diazo-I) were further analyzed by a van’t Hoff plot analysis (Harada & Ogawa, 2004 ▸, 2009 ▸; Harada *et al.*, 2004*b* ▸; Vande Velde *et al.*, 2015 ▸) (Figs. S57–S60). Analysis for diolefin-Br was not performed because disorder is only present at two temperatures. The natural logarithm of the ratio between occupancies of major and minor sites was plotted against the reciprocal of temperature. For the disordered solids imine-I, azo-I and diolefin-I Br, the plots are linear, so the entropic and enthalpic differences between the two sites remain constant during the temperature range and the pedal motion reaches thermodynamic equilibrium. For one of the two unique molecules in diazo-I, the plot is linear in the lower temperature range from 190 to 250 K. On reaching 270 K, the minor conformation of the molecule switches to the major conformation and remains the major site at 290 K. Nonlinearity in van’t Hoff plots has been observed in cases where the enthalpy and entropy are different at high and low temperatures, which can indicate a phase transition (Vande Velde *et al.*, 2011 ▸). In diazo-I, the conformational switching of the major and minor sites corresponds to a phase transition and is the cause of nonlinearity.

### 3.5. Thermal expansion analysis

In order to determine the TE behaviors of these halogenated molecules and the impact of crystal packing and motion on TE, *PASCal* (Cliffe & Goodwin, 2012 ▸) was used to calculate the principal axes (*X*_1_, *X*_2_ and *X*_3_) and TE coefficients (α_*X*_1__ and α_*X*_2__, α_*X*_3__) for each crystal using the VT SCXRD data (Table 2 ▸, Figs. S1–S17). The single-crystal data at 290 K for olefin-I were excluded because the crystal began to disintegrate and data quality was low. Since diazo-I undergoes a phase transition, only the VT SCXRD data between 190 and 250 K was used for the TE calculations. The crystal packing of diazo-I at 250 K is shown in Fig. S62, which is identical to the packing at 290 K [Figs. 9 ▸(*a*) and 9 ▸(*b*)]. The principal axes for each solid are highlighted in Figs. 5 ▸–11 ▸ ▸ ▸ ▸ ▸ ▸. As a benchmark, a TE coefficient ≥ 100 MK^−1^ has been termed ‘colossal’ TE (Goodwin *et al.*, 2008 ▸).

On average, the solids with herringbone packing exhibit different TE behaviors from the face-to-face packed solids along the *X*_1_ and *X*_2_ axes, while the behavior along the *X*_3_ axis is similar. The herringbone solids undergo minimal TE along the *X*_1_ axis, appreciable PTE along the *X*_2_ axis and colossal PTE along *X*_3_ (average: α_*X*_1__ = −2, α_*X*_2__ = 73, α_*X*_3__ = 124 MK^−1^). The face-to-face packed solids undergo PTE along the *X*_1_ and *X*_2_ axes and colossal PTE along *X*_3_ (average: α_*X*_1__ = 9, α_*X*_2__ = 46, α_*X*_3__ = 130 MK^−1^). The key differences in TE behavior of the solids arise from differences in halogen-bond strength, crystal packing and motion occurrence.

For the solids with herringbone crystal packing, the *X*_1_ axis lies along the direction of the 2D halogen-bonded sheet. The *X*_1_ axis corresponds to the crystallographic *b* axis, and the TE coefficients range from −18 to 24 MK^−1^ (Table 2 ▸). Specifically, the molecules with a single motion group exhibit nearly ZTE or PTE, whereas the molecules with two motion groups all exhibit NTE along *X*_1_. The ZTE in olefin-I and olefin-I Br arises from minimal changes along the crystallographic *b* axis (in the third decimal place) as a function of temperature (Tables S1, S2, S5 and S6). The solids olefin-Br, imine-I and azo-I undergo slight to moderate PTE along *X*_1_, and the length of the crystallographic *b* axis increases gradually with increasing temperature in each case. The NTE in diolefin-Br, diolefin-I Br, di­imine-I and di­imine-Br arises from a decrease in the length of the crystallographic *b* axis as the temperature is increased. For diolefin-I, the length of the *b* axis also decreases upon heating overall to afford NTE, but there is a slight increase in length between 230 and 250 K (Tables S19 and S20).

The influence of molecular width on TE has been investigated by Saha and coworkers who showed that the direction of longer molecular width experiences less TE (Rather *et al.*, 2019 ▸). The direction of longer molecular width contains more covalent bonds and less intermolecular interactions; thus, the covalent bonds within the molecule are stronger and will expand less than noncovalent bonds between molecules. In the herringbone structures, the *b* axis corresponds to the longest width of the molecules and does indeed exhibit the smallest expansion. Perhaps more interesting is that when comparing the shorter (one motion group) to the longer (two motion group) molecules, the longer molecules exhibit less expansion along *X*_1_.

The intermolecular interactions that contribute to TE along *X*_1_ are primarily type-II halogen⋯halogen bonds (Table S37). In the symmetrical single- and double-olefin molecules, the type-II I⋯I distances increase by 0.03 Å in both olefin-I and diolefin-I, whereas the type-II Br⋯Br bond lengths increase by 0.04 Å in both olefin-Br and diolefin-Br on heating. In the two di­imine compounds, the type-II bond lengths increase on heating by 0.03 Å on average for di­imine-I and 0.04 Å on average for di­imine-Br. The larger increase in the Br⋯Br bond lengths is expected since they are weaker than I⋯I bonds. In imine-I and azo-I, the type-II I⋯I separations increase by *ca* 0.06 Å on warming. The larger increase in bond length for these iodinated solids could be due to appreciable pedal motion over the entire temperature range, which includes a small repositioning of the iodine atoms. For the unsymmetrical solids, the iodine and bromine atoms sit at nearly identical crystallographic positions with site occupancies of 0.5 and 0.5. This prevented a clear comparison of bond length changes between the symmetrical and unsymmetrical solids because, at any point, the contact could be I⋯I, Br⋯Br or I⋯Br (see Table S37 for all three values). We also calculated the centroid of the I/Br atoms on each side of the type-II bond and measured the distance between the centroids. For olefin-I Br, the centroid separation decreases by 0.01 Å on heating, and the separation increases by 0.03 Å for diolefin-I Br on heating. The type-I halogen⋯halogen interactions also contribute slightly to the expansion along *X*_1_ in the herringbone-packed compounds, and the distances increase by *ca* 0.03–0.09 Å on warming.

Diazo-I exhibits slightly larger PTE along *X*_1_ than the other herringbone solids, but the temperature range used for the calculation only includes data from 190–250 K due to the conformational change at 270 K. The PTE arises from an increase in the length of the *b* axis with increasing temperature, the type-II I⋯I bonds increase by an average of 0.02 Å, and the type-I I⋯I bonds increase by 0.05 Å.

In the isostructural molecules that exhibit face-to-face π-stacked crystal packing, imine-Br exhibits ZTE and the other four molecules undergo slight PTE along *X*_1_. The ZTE in imine-Br arises from minimal changes along the crystallographic *c* axis (in the third decimal place) as a function of temperature (Tables S9 and S10). Unlike the herringbone solids, expansion along *X*_1_ for the face-to-face π-stacked solids encompasses the *a* and/or *c* crystallographic axes (Table 2 ▸). In all five solids, the direction of the *X*_1_ axis includes the C—H⋯*X* forces, which increase by *ca* 0.03–0.06 Å on warming (Table S37). In imine-Br, diazo-Br and diazo-I Br, the π⋯π-stacking interactions also contribute to *X*_1_ and increase by *ca* 0.05–0.07 Å on warming. In one case, namely azo-I Br, the type-II halogen⋯halogen bonds lie along *X*_1_, akin to the herringbone structures, and the centroid I/Br distance increases by 0.03 Å on heating. The difference in the direction of the *X*_1_ axis and included intermolecular forces for the face-to-face solids with respect to the structure results in larger average TE coefficients compared with the herringbone solids.

**Figure 12:**
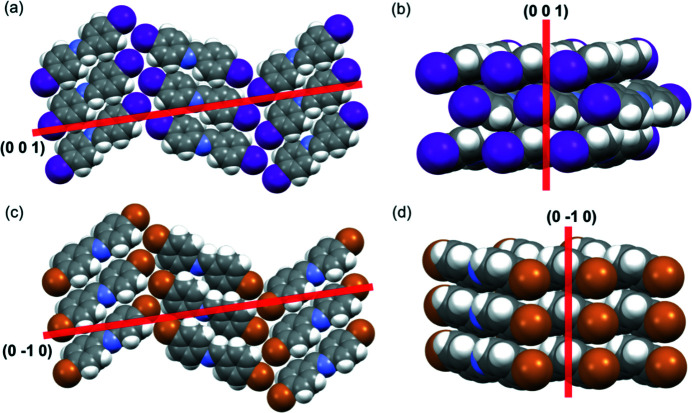
Single-crystal X-ray structures highlighting *X*_2_ axes for representative (*a*) and (*b*) herringbone structures (imine-I), and (*c*) and (*d*) face-to-face π-stacked structures (imine-Br). The *X*_2_ planes are shown in red and going into the page.

The *X*_2_ axis corresponds to the *c* axis in the herringbone solids and the *b* axis for the face-to-face π-stacked solids. Importantly, in both the herringbone and the face-to-face π-stacked solids, the direction of *X*_2_ with respect to the structure is nearly identical. The *X*_2_ axis lies along the vertical direction of the 2D halogen-bonded sheet and includes the type-II halogen⋯halogen contacts [Figs. 12 ▸(*a*) and 12 ▸(*c*); Table S38]. The difference in the two structure types arises in the arrangement of neighboring molecules within the 2D sheet. In the herringbone structures, molecules within a sheet are parallel, but lie edge-to-face between the sheets [Fig. 12 ▸(*b*)]. In the face-to-face structures, molecules within a sheet and between sheets are parallel [Fig. 12 ▸(*d*)]. On heating, the solids in the herringbone arrangement twist further from co-planarity, and for solids that undergo pedal motion, the conformational interconversion affects the *X*_2_ axis [Fig. 12 ▸(*b*)]. On the other hand, molecules in the face-to-face structures remain coplanar and do not undergo motion. This structural difference results in a difference in TE behaviors; thus, the solids with herringbone packing exhibit larger PTE along *X*_2_. Additionally, on average, the *c*-axis length in the herringbone solids experiences a larger increase on heating than the *b* axis length in the face-to-face π-stacked molecules.

All 16 solids in the herringbone and face-to-face categories exhibit colossal (Goodwin *et al.*, 2008 ▸) PTE along *X*_3_, and on average, the solids exhibit similar TE coefficients (Table 2 ▸). The *X*_3_ axis in both types of solids lies along the π-stacking direction. For the herringbone solids, this encompasses the C—H⋯π interactions, whereas in the face-to-face stacked solids, this includes π⋯π interactions. On heating, the C—H⋯π distances increase by *ca* 0.04–0.05 Å on average in the herringbone solids, whereas the π⋯π distances increase by *ca* 0.05–0.07 Å on average in the face-to-face stacked solids (Table S39). These differences in interaction type and distance change over the temperature range afford a slightly higher TE average for the face-to-face π-stacked solids. The solid imine-Br exhibits the largest PTE along *X*_3_ (α_*X*_3__ = 162 MK^−1^) due to the largest increase in π⋯π distance (0.07 Å).

The volumetric TE coefficient for all 16 solids is also colossal and ranges from 166 to 212 MK^−1^. The volume of each crystal increases gradually on warming (Figs. S40–S56) and the volume increases by *ca* 1.5–2.1% on heating. On average, the solids with herringbone packing exhibit slightly larger volumetric TE (average α_V_ = 197 MK^−1^) than the solids with face-to-face π-stacking (average α_V_ = 187 MK^−1^), owing to the significantly larger expansion along *X*_2_.

### 3.6. Unsymmetrical olefins

The unsymmetrical I⋯Br systems were designed to offer an interplay between the symmetrical I⋯I and Br⋯Br solids with regard to bond strength and TE along the halogen-bonding direction. All six olefins crystallized in the herringbone packing motif, and the type-II halogen⋯halogen interactions contribute to TE along the *X*_1_ and *X*_2_ axes. Gratifyingly, the solid olefin-I Br exhibits a TE coefficient between olefin-I and olefin-Br for both α_*X*_1__ and α_*X*_2__. The solid diolefin-I Br exhibits a TE coefficient between diolefin-I and diolefin-Br for α_*X*_1__; however, the TE coefficient of diolefin-I Br along *X*_2_ is within the error of diolefin-Br.

## 4. Conclusions

Here, we report the TE behaviors and the impact of crystal packing and pedal motion on TE in 16 di-halogenated molecules with one or two motion-capable moieties, which self-assemble into herringbone or face-to-face π-stacked structures. Dynamic pedal motion was successfully achieved in five solids, which all exhibit herringbone packing; thus, crystal packing influences motion ability. Moreover, the degree of pedal motion is affected by the identity of the motion group, and the azo groups undergo the largest changes. Halogen bonds contribute to TE along *X*_1_ in the herringbone solids and *X*_2_ in all 16 solids. The type-II I⋯I bonds are stronger and undergo less expansion than the Br⋯Br bonds. On average, herringbone-packed solids exhibit larger TE along one direction of the 2D halogen-bonded sheet and larger volumetric expansion, which results from twisting within the herringbone arrangement and pedal motion. The TE along *X*_3_ corresponds to the π-stacking direction. The degree of TE is similar in both types of molecules, but face-to-face packed solids experience slightly more TE along *X*_3_ due to the larger increase in the π⋯π distances. Solid-state packing arrangements and noncovalent interaction strengths both contribute to TE in aromatic based solids, which are important in the field of organic electronics. We are continuing to investigate TE properties of aromatic and halogen-bonded solids.

## 5. Related literature

The following references are cited in the supporting information: Wang *et al.* (2012 ▸, 2018 ▸); Mizoshita *et al.* (2008 ▸); Huskić *et al.* (2012 ▸); Nguyen *et al.* (2011 ▸); Albers *et al.* (2011 ▸); Tian *et al.* (2017 ▸); Sheldrick (2015 ▸); Rigaku (2018 ▸); Oxford Diffraction (2015 ▸).

## Supplementary Material

Crystal structure: contains datablock(s) global, olefinI_190, olefinI_210, olefinI_230, olefinI_250, olefinI_270, olefinBr_190, olefinBr_210, olefinBr_230, olefinBr_250, olefinBr_270, olefinBr_290, olefinIBr_190K, olefinIBr_210K, olefinIBr_230K, olefinIBr_250K, olefinIBr_270K, olefinIBr_290K. DOI: 10.1107/S2052252521009593/lq5042sup1.cif

Supporting information, figures and tables. DOI: 10.1107/S2052252521009593/lq5042sup2.pdf

Combined cif and checkcif files. DOI: 10.1107/S2052252521009593/lq5042sup3.zip

CCDC references: 2093499, 2093500, 2093501, 2093502, 2093503, 2093504, 2093505, 2093506, 2093507, 2093508, 2093509, 2093510, 2093511, 2093512, 2093513, 2093514, 2093515

## Figures and Tables

**Table 1:** Site occupancies of the major conformations within molecules that undergo dynamic pedal motion

Crystal	290 K	270 K	250 K	230 K	210 K	190 K
Imine-I	0.78 (1)	0.79 (1)	0.83 (1)	0.84 (1)	0.86 (1)	0.87 (1)
Azo-I	0.73 (1)	0.78 (1)	0.80 (1)	0.85 (1)	0.88 (1)	0.93 (1)
Diolefin-Br	0.97 (1)	0.98 (1)	1.00	1.00	1.00	1.00
Diolefin-I Br	0.93 (1)	0.95 (1)	0.97 (1)	1.00	1.00	1.00
Diazo-I	0.73 (1)	0.80 (1)	1.00	1.00	1.00	1.00
	0.72 (1)	0.71 (1)	0.70 (2)	0.82 (1)	0.91 (1)	0.96 (1)

**Table 2:** TE coefficients for crystals with errors denoted in parentheses and approximate crystallographic axes denoted in brackets The solids are divided into sections based on crystal packing. The average values for solids with the same packing are provided at the bottom of each section.

Crystal	α_*X*_1__ (MK^−1^) [axis]	α_*X*_2__ (MK^−1^) [axis]	α_*X*_3__ (MK^−1^) [axis]	α_V_ (MK^−1^)
Herringbone crystal packing				
Olefin-I	−2 (1) [0 1 0]	68 (5) [0 0 1]	128 (1) [−1 0 0]	194 (3)
Olefin-Br	11 (1) [0 1 0]	83 (2) [0 0 1]	114 (1) [−1 0 0]	209 (2)
Olefin-I Br	0.5 (0.5) [0 1 0]	75 (1) [0 0 1]	115 (2) [−1 0 0]	191 (2)
Imine-I	8 (1) [0 −1 0]	70 (1) [0 0 1]	134 (1) [1 0 0]	212 (2)
Azo-I	24 (1) [0 −1 0]	60 (1) [0 0 1]	120 (1) [1 0 0]	205 (3)
Diolefin-I	−18 (4) [0 −1 0]	71 (1) [0 0 1]	135 (4) [1 0 0]	194 (9)
Diolefin-Br	−4 (1) [0 1 0]	76 (2) [0 0 1]	114 (3) [−1 0 0]	187 (3)
Diolefin-I Br	−15 (1) [0 1 0]	78 (1) [0 0 1]	131 (3) [−1 0 0]	195 (3)
Di­imine-I	−9 (1) [0 1 0]	74 (2) [0 0 −1]	120 (1) [−1 0 0]	186 (3)
Di­imine-Br	−12 (1) [0 1 0]	75 (1) [0 0 −1]	133 (1) [−1 0 0]	196 (3)
Average coefficients for herringbone	−2	73	124	197

Herringbone crystal packing (with a phase transition)				
Diazo-I†	38 (4) [0 1 0]	41 (4) [1 0 -2]	120 (3) [-3 0 -2]	199 (2)

Face-to-face π-stacked crystal packing				
Imine-Br	0 (1) [1 0 2]	43 (1) [0 −1 0]	162 (3) [1 0 0]	206 (2)
Azo-Br(a)	16 (1) [1 0 9]	52 (1) [0 −1 0]	122 (2) [1 0 0]	191 (3)
Azo-I Br	12 (1) [0 0 1]	52 (2) [0 −1 0]	134 (3) [1 0 0]	199 (3)
Diazo-Br	9 (2) [−1 0 4]	42 (1) [0 1 0]	114 (3) [−1 0 0]	166 (4)
Diazo-I Br	10 (1) [1 0 −2]	42 (1) [0 −1 0]	120 (2) [1 0 0]	173 (2)
Average coefficients for π-stacked	9	46	130	187

† Only the VT SCXRD data between 190 and 250 K for diazo-I are used for the TE calculations due to a phase transition. The diazo-I coefficients are not included with the average values for the other herringbone structures because the calculations do not encompass the same temperature range.
