# Supplementary material for: Differences in thermal expansion and motion ability for herringbone and face-to-face π-stacked solids
Source: IUCrJ. 2021 Nov 3;9(Pt 1):31–42. doi: 10.1107/S2052252521009593 (PMC8733877; doi:10.1107/S2052252521009593)
Supplement: Supplementary file 2 [file m-09-00031-sup2.pdf]

IUCrJ

Volume 9 (2022)

Supporting information for article:

Differences in thermal expansion and motion ability for herringbone and face-to-face π-stacked solids

Xiaodan Ding, Ethan Zahid, Daniel K. Unruh and Kristin M. Hutchins

Supplementary Information

Differences in thermal expansion and motion ability for herringbone and face-to-face π-stacked solids

Xiaodan Ding, Ethan Zahid, Daniel K. Unruh and Kristin M. Hutchins*

Department of Chemistry and Biochemistry, Texas Tech University, Lubbock, Texas, 79409, United States.

Email: kristin.hutchins@ttu.edu

1. Materials, Synthesis, and Crystallization S2-S9
2. X-ray Diffraction Information and Data Tables S10-S44
3. Thermal Expansion Data and Intermolecular Interaction Distances S45-S51
4. Expansivity Indicatrix Diagrams S52-S60
5. NMR Spectra of the Compounds S61-S70
6. PXRD Patterns S71-S72
7. Variation of the Unit Cell Parameters S73-S81
8. van't Hoff Plots S82-S84
9. Single-Crystal X-ray Structures of **diazo-I** S85
10. Single-Crystal X-ray Structures and Analysis of **azo-Br**(b) S86-87
11. References S88

1. Materials, Synthesis, and Crystallization

Materials

4-Iodobenzyl bromide, triethyl phosphite, 4-iodobenzaldehyde, potassium *tert*-butoxide, 4-bromobenzyl bromide, 4-bromobenzaldehyde, 4-iodoaniline, 4-bromoaniline, and benzene-1,4-diamine were all purchased from Oakwood Chemical (730 Columbia Hwy, SC, USA). Oxone was purchased from Alfa Aesar (Ward Hill, MA, USA). Xylenes and acetone were purchased from Avantor Performance Materials (Center Valley, PA, USA). Terephthalaldehyde was purchased from Tokyo Chemical Industry (Portland, OR, USA). Magnesium sulfate, sodium bicarbonate, hexanes, chloroform, toluene, acetonitrile, acetic acid, ethyl acetate, tetrahydrofuran (THF), and dichloromethane (DCM) were all purchased from Fisher Chemical (Fair Lawn, NJ, USA). Ethanol (200 proof) was purchased from Pharmco (Shelbyville, KY, USA).

Synthesis of olefin-I

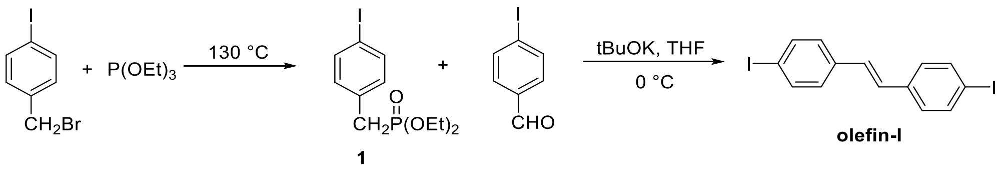

Olefin-I was synthesized using a modified literature procedure.[1-3] In the first step, the mixture of 4-iodobenzyl bromide (2.38 g, 8 mmol) and triethyl phosphite (1.6 mL, 9.2 mmol) were refluxed at 130 ºC for 2 h. After cooling to room temperature, ethyl acetate and water were added to the mixture. The organic layer was separated and dried with magnesium sulfate. After evaporating the solvent under reduced pressure, the excess triethyl phosphite was removed by heating at 160 ºC to afford compound **1** (2.43 g, 86% yield, Figure S18). In the second step, to the mixture of potassium *tert*-butoxide (0.84 g, 7.5 mmol) and 10 mL of THF, compound **1** (0.97 g, 2.75 mmol) was added dropwise. The solution was stirred for a few minutes at 0 ºC and then 4-iodobenzaldehyde (0.58 g, 2.5 mmol) was added. The reaction mixture was kept at 0 ºC and stirred for 2 h. Ice water was then added to quench the reaction. The resulting solid was filtered and washed with water and ethanol to afford **olefin-I** (*trans only*, 0.58 g, 54% yield, Figure S19). Single crystals were grown from ethanol.

Synthesis of olefin-Br

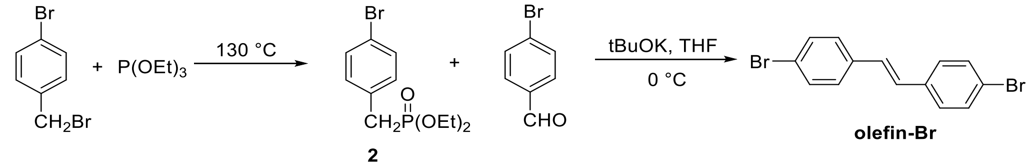

Olefin-Br was synthesized using a modified literature procedure.[1-3] In the first step, the mixture of 4-bromobenzyl bromide (2 g, 8 mmol) and triethyl phosphite (1.6 mL, 9.2 mmol) were refluxed at 130 ºC for 2 h. After cooling to room temperature, ethyl acetate and water were added to the mixture. The organic layer was separated and dried with magnesium sulfate. After evaporating the solvent under reduced pressure, the excess triethyl phosphite was removed by heating at 160 ºC to afford compound **2** (1.81 g, 74% yield, Figure S20). In the second step, to the mixture of potassium *tert*-butoxide (0.84 g, 7.5 mmol) and 10 mL of THF, compound **2** (0.84 g, 2.75 mmol) was added dropwise. The solution was stirred for a few minutes at 0 ºC and then 4-bromobenzaldehyde (0.46 g, 2.5 mmol) was added. The reaction mixture was kept at 0 ºC and stirred for 2 h. Ice water was then added to quench the reaction. The resulting solid was filtered and washed with water and ethanol to afford **olefin-Br** (*trans only,* 0.29 g, 34% yield, Figure S21). Single crystals were grown from ethanol.

Synthesis of olefin-I Br

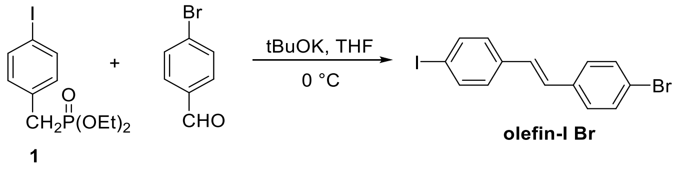

Olefin-I Br was synthesized using a modified literature procedure.[1-3] To the mixture of potassium *tert*-butoxide (0.84 g, 7.5 mmol) and 10 mL of THF, compound **1** (0.97 g, 2.75 mmol, for preparation, see synthesis of **olefin-I** above) was added dropwise. The solution was stirred for a few minutes at 0 ºC and then 4-bromobenzaldehyde (0.46 g, 2.5 mmol) was added. The reaction mixture was kept at 0 ºC and stirred for 2 h. Ice water was then added to quench the reaction. The resulting solid was filtered and washed with water and ethanol to afford **olefin-I Br** (*trans only*, 0.41 g, 43% yield, Figure S22). Single crystals were grown from ethanol.

Synthesis of imine-I

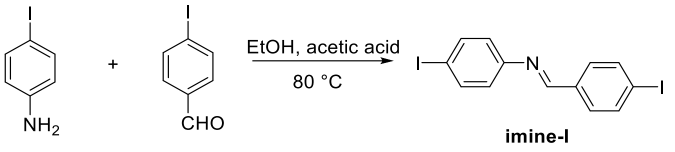

Imine-I was synthesized using a literature procedure.[4] 4-Iodoaniline (1.10 g, 5 mmol) was dissolved in 5 mL of ethanol. A solution of 4-iodobenzaldehyde (1.16 g, 5 mmol) dissolved in 15 mL of ethanol was then added slowly under vigorous stirring. A few drops of acetic acid were also added. The mixture was refluxed overnight under N_2, then cooled to room temperature and left to sit undisturbed in the fume hood for 24 h. The resulting precipitate was filtered and washed with ethanol. The solid was dried under vacuum to give **imine-I** (1.65 g, 75% yield, Figure S23). Single crystals were grown from acetonitrile.

Synthesis of imine-Br

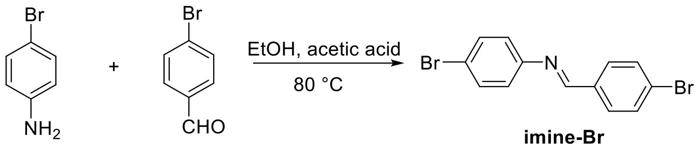

Imine-Br was synthesized using a literature procedure.[4] 4-Bromoaniline (0.86 g, 5 mmol) was dissolved in 5 mL of ethanol. The solution of 4-bromobenzaldehyde (0.93 g, 5 mmol) dissolved in 15 mL of ethanol was then added slowly under vigorous stirring. A few drops of acetic acid were also added. The mixture was refluxed overnight under N_2, then cooled to room temperature and left to sit undisturbed in the fume hood for 24 h. The resulting precipitate was filtered out and washed with ethanol. The solid was dried under vacuum to give **imine-Br** (1.31 g, 77% yield, Figure S24). Single crystals were grown from ethanol.

Synthesis of azo-I

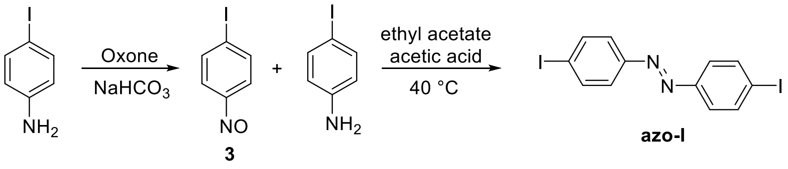

Azo-I was synthesized using a modified literature procedure.[5,6] In the first step, 4-iodoaniline (0.05 g, 0.25 mmol), oxone (0.31 g, 0.5 mmol), and sodium bicarbonate (0.08 g, 1 mmol) were added to a milling jar with two milling balls and milled at 1500 rpm for 20 min using a FTS1000 Ball Mill purchased from Form-Tech Scientific. Compound **3** (0.02 g, 34% yield, Figure S25)

was then isolated from the mixture by sublimation. In the second step, compound **3** (0.43 g, 1.85 mmol) was dissolved in 20 mL of a mixture of acetic acid and ethyl acetate (1:1 *v*:*v*). 4-Iodoaniline (0.32 g, 1.48 mmol) was then added under stirring. The mixture was stirred at 40 °C overnight. After cooling to room temperature, the precipitate was filtered and washed with ethyl acetate to give **azo-I** (0.27 g, 42% yield, Figure S26). Single crystals were grown from acetone.

Synthesis of azo-Br

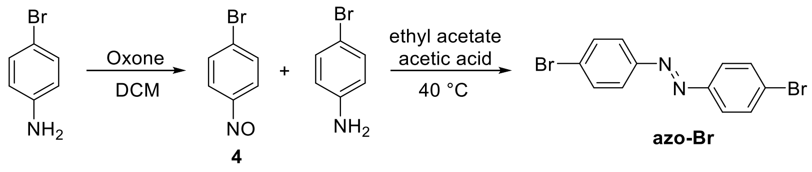

Azo-Br was synthesized using a modified literature procedure.[6] In the first step, 4-bromoaniline (0.41 g, 2.37 mmol) was dissolved in 5 mL of DCM. Oxone (2.91 g, 4.73 mmol) dissolved in 20 mL of water was then added. The mixture was stirred under N_2 at room temperature for 4 h. The reaction mixture was extracted with DCM and dried with magnesium sulfate. After evaporation under reduced pressure, compound **4** (0.36 g, 82% yield, Figure S27) was obtained, which was used in the next step without further purification. In the second step, compound **4** (0.35 g, 1.85 mmol) was dissolved in 20 mL of a mixture of acetic acid and ethyl acetate (1:1 *v*:*v*). 4-Bromoaniline (0.25 g, 1.48 mmol) was then added under stirring. The mixture was stirred at 40 °C overnight. After cooling to room temperature, the precipitate was filtered and washed with ethyl acetate to afford **azo-Br** (0.17 g, 34% yield, Figure S28). Single crystals of polymorph-a **azo-Br(a)** were grown from benzene,[7] and single crystals of polymorph-b **azo-Br(b)** were grown from acetone.

Synthesis of azo-I Br

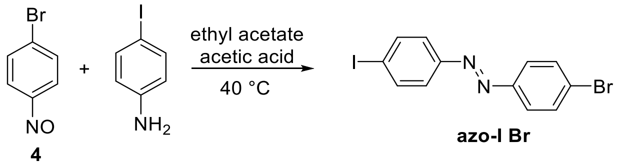

Azo-I Br was synthesized using a modified literature procedure.[6] Compound **4** (0.35 g, 1.85 mmol, for preparation, see synthesis of **azo-Br** above) was dissolved in 20 mL of a mixture of acetic acid and ethyl acetate (1:1 *v*:*v*). 4-Iodoaniline (0.32 g, 1.48 mmol) was then added under

stirring. The mixture was stirred at 40 ºC overnight. After cooling to room temperature, the precipitate was filtered and washed with ethyl acetate to afford **azo-I Br** (0.19 g, 33% yield, Figure S29). Single crystals were grown from acetonitrile.

Synthesis of diolefin-I

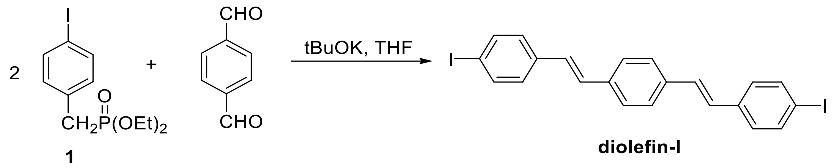

Diolefin-I was synthesized using a modified literature procedure.[1-3] The mixture of compound **1** (0.97 g, 2.75 mmol, for preparation, see synthesis of **olefin-I** above) and potassium *tert*-butoxide (0.84 g, 7.5 mmol) in 40 mL of THF was stirred for several minutes in an ice bath. Terephthalaldehyde (0.17 g, 1.25 mmol) was then added under stirring. The reaction mixture was stirred overnight at room temperature. Water was then added to quench the reaction. The resulting solid was filtered and washed with water and ethanol to afford **diolefin-I** (*trans only*, 0.30 g, 45% yield, Figure S37). Single crystals were grown from the mixture of toluene and DCM (approximately 1:1 *v*:*v*). The bulk solid was insoluble in deuterated solvents, so PXRD was used for characterization.

Synthesis of diolefin-Br

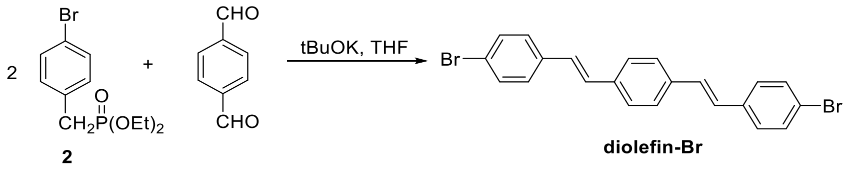

Diolefin-Br was synthesized using a modified literature procedure.[1-3] The mixture of compound **2** (0.84 g, 2.75 mmol, for preparation, see synthesis of **olefin-Br** above) and potassium *tert*-butoxide (0.84 g, 7.5 mmol) in 40 mL of THF was stirred for several minutes in an ice bath. Terephthalaldehyde (0.17 g, 1.25 mmol) was then added with stirring. The reaction mixture was stirred overnight at room temperature. Water was then added to quench the reaction. The resulting solid was filtered and washed with water and ethanol to afford **diolefin-Br** (*trans only*, 0.15 g, 27% yield, Figure S38). Single crystals were grown from the mixture of toluene and DCM (approximately 1:1 *v*:*v*). The bulk solid was insoluble in deuterated solvents, so PXRD was used for characterization.

Synthesis of diolefin-I Br

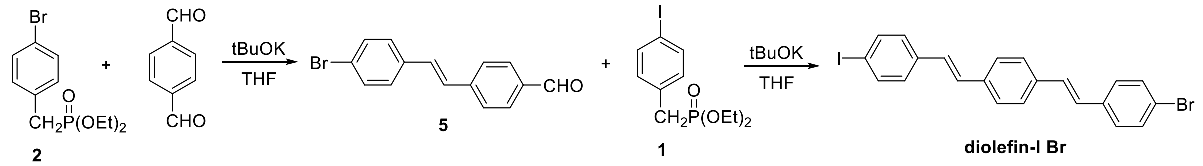

Diolefin-I Br was synthesized using a modified literature procedure.[1-3,8] In the first step, terephthalaldehyde (1.61 g, 12.02 mmol) was dissolved in 85 mL of THF. Compound **2** (1.02 g, 3.32 mmol, for preparation, see synthesis of **olefin-Br** above) and potassium *tert*-butoxide (0.58 g, 5.2 mmol) were then added. The mixture was stirred under N_2 for 40 min and the additional potassium *tert*-butoxide (0.58 g, 5.2 mmol) was added. The mixture was stirred under N_2 for another 30 min. The reaction mixture was filtered, and the solvent was removed under reduced pressure. The crude product was purified through column chromatography with DCM and hexanes (1:1 *v*:*v*) to form compound **5** (0.37 g, 39% yield, Figure S30). In the second step, the mixture of compound **1** (0.35 g, 1 mmol, for preparation, see synthesis of **olefin-I** above) and potassium *tert*-butoxide (0.31 g, 2.72 mmol) in 30 mL of THF was stirred for several minutes in an ice bath. Compound **5** (0.26 g, 0.9 mmol) was then added with stirring. The reaction mixture was stirred overnight at room temperature. Water was then added to quench the reaction. The resulting solid was filtered and washed with water and ethanol to afford **diolefin-I Br** (*trans only*, 0.31 g, 71% yield, Figure S39). Single crystals were grown from the mixture of toluene and DCM (approximately 1:1 *v*:*v*). The bulk solid was insoluble in deuterated solvents, so PXRD was used for characterization.

Synthesis of diimine-I

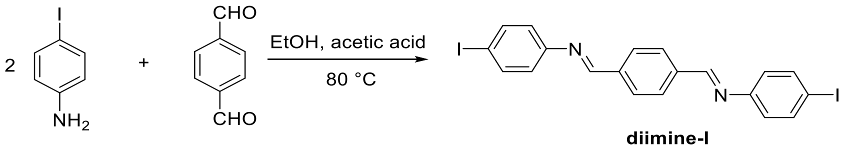

Diimine-I was synthesized using a literature procedure.[4] 4-Iodoaniline (1.10 g, 5 mmol) was dissolved in 5 mL of ethanol. A solution of terephthalaldehyde (0.34 g, 2.5 mmol) dissolved in 30 mL of ethanol was then added slowly under vigorous stirring. A few drops of acetic acid were also added. The mixture was refluxed overnight under N_2, then cooled to room temperature and left to sit undisturbed in the fume hood for 24 h. The resulting precipitate was filtered and washed with ethanol. The solid was dried under vacuum to give **diimine-I** (1.22 g, 91% yield, Figure S31). Single crystals were grown from toluene.

Synthesis of diimine-Br

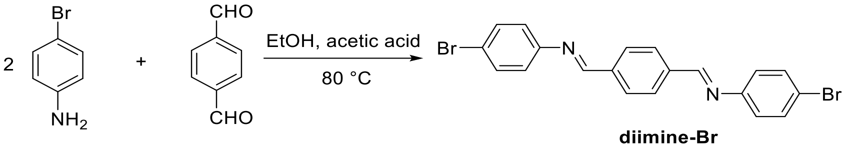

Diimine-Br was synthesized using a literature procedure.[4] 4-Bromoaniline (0.86 g, 5 mmol) was dissolved in 5 mL of ethanol. A solution of terephthalaldehyde (0.34 g, 2.5 mmol) dissolved in 30 mL of ethanol was then added slowly under vigorous stirring. A few drops of acetic acid were also added. The mixture was refluxed overnight under N_2, then cooled to room temperature and left to sit undisturbed in the fume hood for 24 h. The resulting precipitate was filtered and washed with ethanol. The solid was dried under vacuum to give **diimine-Br** (0.98 g, 89% yield, Figure S32). Single crystals were grown from chloroform.

Synthesis of diazo-I

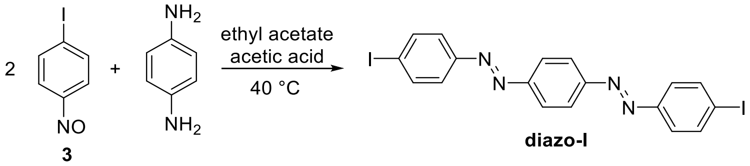

Diazo-I was synthesized using a modified literature procedure.[5,6] Compound **3** (0.20 g, 0.85 mmol, for preparation, see synthesis of **azo-I** above) was dissolved in 20 mL of a mixture of acetic acid and ethyl acetate (1:1 *v*:*v*). Benzene-1,4-diamine (0.04 g, 0.36 mmol) was then added under stirring. The mixture was stirred at 40 ºC overnight. After cooling to room temperature, the precipitate was filtered and washed with ethyl acetate to afford **diazo-I** (0.06 g, 31% yield, Figure S33). Single crystals were grown from toluene.

Synthesis of diazo-Br

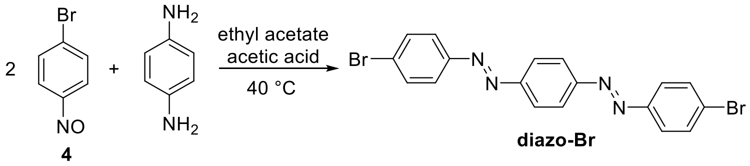

Diazo-Br was synthesized using a modified literature procedure.[6] Compound **4** (0.34 g, 1.85 mmol, for preparation, see synthesis of **azo-Br** above) was dissolved in 20 mL of a mixture of acetic acid and ethyl acetate (1:1 *v*:*v*). Benzene-1,4-diamine (0.08 g, 0.74 mmol) was then added under stirring. The mixture was stirred at 40 ºC overnight. After cooling to room

temperature, the precipitate was filtered and washed with ethyl acetate to afford **diazo-Br** (0.11 g, 33% yield, Figure S34). Single crystals were grown from toluene.

Synthesis of diazo-I Br

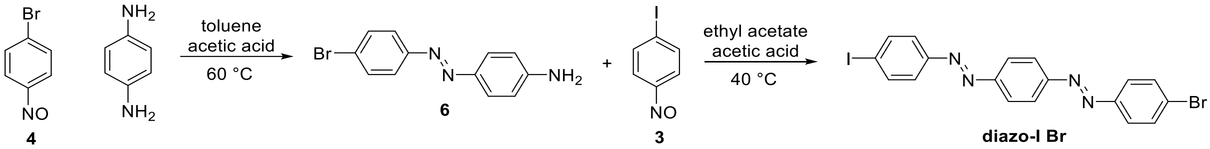

Diazo-I Br was synthesized using a modified literature procedure.[5,6,9] In the first step, benzene--1,4-diamine (0.17 g, 1.60 mmol) was dissolved in 8 mL of toluene. Compound **4** (0.37 g, 2 mmol, for preparation, see synthesis of **azo-Br** above) and acetic acid (0.39 g, 6.5 mmol) were then added. The mixture was stirred at 60 ºC overnight under N_2. The mixture was filtered and extracted with DCM and water. The organic phase was dried with magnesium sulfate and evaporated under reduced pressure. The crude product was purified through column chromatography with hexanes and ethyl acetate (4:1 *v*:*v*) to afford compound **6** (0.31g, 70% yield, Figure S35). In the second step, compound **3** (0.14 g, 0.62 mmol, for preparation, see synthesis of **azo-I** above) was dissolved in 6 mL of a mixture of acetic acid and ethyl acetate (1:1 *v*:*v*). Compound **6** (0.14 g, 0.52 mmol) was then added under stirring. The mixture was stirred at 40 ºC overnight. After cooling to room temperature, the precipitate was filtered and washed with ethyl acetate to afford **diazo-I Br** (0.14 g, 55% yield, Figure S36). Single crystals were grown from toluene.

2. X-ray Diffraction Information and Data Tables

Data were collected on a Rigaku XtaLAB Synergy-*i*Kappa diffractometer equipped with a PhotonJet-*i* X-ray source operated at 50 W (50 kV, 1 mA) to generate Cu Kα radiation (λ = 1.54178 Å) and a HyPix-6000HE HPC (hybrid photon counting) detector. Crystals were transferred from the vial and placed on a glass slide in polyisobutylene. A Zeiss Stemi 305 microscope was used to identify a suitable specimen for X-ray diffraction from a representative sample of the material. The crystal and a small amount of the oil were collected on a Hampton Research20 micron cryoloop and transferred to the instrument where it was placed under a cold nitrogen stream (Oxford). Data were collected at temperatures of 290 K, 270 K, 250 K, 230 K, 210 K, and 190 K with a transition rate of 2 K/minute between the temperatures. The sample was optically centered with the aid of a video camera to ensure that no translations were observed as the crystal was rotated through all positions. The crystal was measured for size, morphology, and color.

After data collection, the unit cell was re-determined using a subset of the full data collection for each temperature. Intensity data were corrected for Lorentz, polarization, and background effects using *CrysAlis^{Pro}*.[10] A numerical absorption correction was applied based on a Gaussian integration over a multifaceted crystal and followed by a semi-empirical correction for adsorption applied using the program *SCALE3 ABSPACK.*[11] The *SHELX-2014*,[12] series of programs was used for the solution and refinement of the crystal structures. Hydrogen atoms bound to carbon atoms were located in the difference Fourier map and were geometrically constrained using the appropriate AFIX commands.

The single crystal data of **olefin-I** at 290 K was not included because the crystal began to disintegrate and data quality was low.

In the single crystal data of **diazo-I** at 270 and 290 K, the *β*-angle of the unit cell goes nearly to 90°. However, the presence of the disordered azo moieties prevents the structure from being in a higher symmetry space group. We attempted to refine the data sets in an orthorhombic space group, but the initial R_{int} and R_1 values were higher than when we refined the structure in the monoclinic space group (the space group for the 250-190 K data sets). Thus, the structure was solved in the monoclinic space group at all six temperatures.

Table S1. X-ray data for **olefin-I** at 270 and 250 K.

compound formula	$C_{14}H_{10}I_2$	$C_{14}H_{10}I_2$
formula mass	432.02	432.02
crystal system	Orthorhombic	Orthorhombic
space group	*Pccn*	*Pccn*
a/Å	7.4485(3)	7.4287(3)
b/Å	28.9467(11)	28.9400(9)
c/Å	6.0127(3)	6.00927(19)
α/°	90	90
β/°	90	90
γ/°	90	90
V/Å^3	1296.39(9)	1291.91(7)
ρ_{calc}/g cm^{-3}	2.213	2.221
T/K	270(2)	250(2)
Z	4	4
radiation type	CuKα	CuKα
absorption coefficient, μ/mm^{-1}	37.848	37.979
no. of reflections measured	11640	12600
no. of independent reflections	1367	1360
no. of reflection (I > 2σ(I))	1140	1189
R_{int}	0.0645	0.0657
R_1 (I > 2σ(I))	0.0414	0.0361
wR(F^2) (I > 2σ(I))	0.1196	0.1002
R_1 (all data)	0.0483	0.0406
wR(F^2) (all data)	0.1338	0.1064
Goodness-of-fit	1.105	1.078
CCDC deposition number	2093503	2093502

Table S2. X-ray data for **olefin-I** at 230, 210, and 190 K.

compound formula	$C_{14}H_{10}I_2$	$C_{14}H_{10}I_2$	$C_{14}H_{10}I_2$
formula mass	432.02	432.02	432.02
crystal system	Orthorhombic	Orthorhombic	Orthorhombic
space group	*Pccn*	*Pccn*	*Pccn*
a/Å	7.40898(16)	7.39255(12)	7.3723(2)
b/Å	28.9461(6)	28.9478(4)	28.9496(8)
c/Å	6.00040(12)	5.99085(9)	5.98149(14)
α/°	90	90	90
β/°	90	90	90
γ/°	90	90	90
V/Å^3	1286.85(4)	1282.03(3)	1276.60(6)
ρ_{calc}/g cm^{-3}	2.230	2.238	2.248
T/K	230(2)	210(2)	190(2)
Z	4	4	4
radiation type	CuKα	CuKα	CuKα
absorption coefficient, μ/mm^{-1}	38.129	38.272	38.435
no. of reflections measured	12598	12977	12742
no. of independent reflections	1351	1341	1330
no. of reflection (I > 2σ(I))	1225	1228	1234
R_{int}	0.0528	0.0502	0.0501
R_1 (I > 2σ(I))	0.0281	0.0524	0.0534
wR(F^2) (I > 2σ(I))	0.0750	0.1213	0.1274
R_1 (all data)	0.0315	0.0542	0.0546
wR(F^2) (all data)	0.0773	0.1241	0.1294
Goodness-of-fit	1.074	1.154	1.143
CCDC deposition number	2093501	2093500	2093499

Table S3. X-ray data for **olefin-Br** at 290, 270, and 250 K.

compound formula	$C_{14}H_{10}Br_2$	$C_{14}H_{10}Br_2$	$C_{14}H_{10}Br_2$
formula mass	338.04	338.04	338.04
crystal system	Orthorhombic	Orthorhombic	Orthorhombic
space group	*Pccn*	*Pccn*	*Pccn*
a/Å	7.4125(2)	7.39602(15)	7.37825(13)
b/Å	27.6845(8)	27.6823(6)	27.6789(5)
c/Å	5.95711(19)	5.94943(12)	5.93730(11)
α/°	90	90	90
β/°	90	90	90
γ/°	90	90	90
V/Å^3	1222.46(6)	1218.08(4)	1212.53(4)
ρ_{calc}/g cm^{-3}	1.837	1.843	1.852
T/K	290(2)	270(2)	250(2)
Z	4	4	4
radiation type	CuKα	CuKα	CuKα
absorption coefficient, μ/mm^{-1}	8.136	8.165	8.203
no. of reflections measured	11242	14316	13410
no. of independent reflections	1211	1264	1252
no. of reflection (I > 2σ(I))	992	1073	1100
R_{int}	0.0567	0.0567	0.0539
R_1 (I > 2σ(I))	0.0352	0.0305	0.0284
wR(F^2) (I > 2σ(I))	0.0930	0.0757	0.0762
R_1 (all data)	0.0425	0.0365	0.0316
wR(F^2) (all data)	0.0992	0.0793	0.0780
Goodness-of-fit	1.063	1.054	1.049
CCDC deposition number	2093509	2093508	2093507

Table S4. X-ray data for **olefin-Br** at 230, 210, and 190 K.

compound formula	$C_{14}H_{10}Br_2$	$C_{14}H_{10}Br_2$	$C_{14}H_{10}Br_2$
formula mass	338.04	338.04	338.04
crystal system	Orthorhombic	Orthorhombic	Orthorhombic
space group	*Pccn*	*Pccn*	*Pccn*
a/Å	7.36051(14)	7.34625(11)	7.3293(2)
b/Å	27.6702(6)	27.6646(4)	27.6552(12)
c/Å	5.92784(11)	5.91922(8)	5.9082(2)
α/°	90	90	90
β/°	90	90	90
γ/°	90	90	90
V/Å^3	1207.30(4)	1202.97(3)	1197.55(8)
ρ_{calc}/g cm^{-3}	1.860	1.866	1.875
T/K	230(2)	210(2)	190(2)
Z	4	4	4
radiation type	CuKα	CuKα	CuKα
absorption coefficient, μ/mm^{-1}	8.238	8.268	8.305
no. of reflections measured	13730	14940	12105
no. of independent reflections	1246	1259	1230
no. of reflection (I > 2σ(I))	1111	1155	1028
R_{int}	0.0469	0.0480	0.0663
R_1 (I > 2σ(I))	0.0273	0.0287	0.0505
wR(F^2) (I > 2σ(I))	0.0747	0.0729	0.1382
R_1 (all data)	0.0306	0.0309	0.0609
wR(F^2) (all data)	0.0768	0.0744	0.1538
Goodness-of-fit	1.107	1.070	1.163
CCDC deposition number	2093506	2093505	2093504

Table S5. X-ray data for **olefin-I Br** at 290, 270, and 250 K.

compound formula	$C_{14}H_{10}BrI$	$C_{14}H_{10}BrI$	$C_{14}H_{10}BrI$
formula mass	385.03	385.03	385.03
crystal system	Orthorhombic	Orthorhombic	Orthorhombic
space group	*Pccn*	*Pccn*	*Pccn*
a/Å	7.44877(12)	7.42999(8)	7.41258(8)
b/Å	28.3440(4)	28.3468(3)	28.3456(3)
c/Å	6.00033(8)	5.99099(5)	5.98174(5)
α/°	90	90	90
β/°	90	90	90
γ/°	90	90	90
V/Å^3	1266.84(3)	1261.80(2)	1256.85(2)
ρ_{calc}/g cm^{-3}	2.019	2.027	2.035
T/K	290(2)	270(2)	250(2)
Z	4	4	4
radiation type	CuKα	CuKα	CuKα
absorption coefficient, μ/mm^{-1}	23.291	23.384	23.476
no. of reflections measured	13397	14520	14290
no. of independent reflections	1330	1326	1319
no. of reflection (I > 2σ(I))	1232	1259	1258
R_{int}	0.0585	0.0580	0.0558
R_1 (I > 2σ(I))	0.0312	0.0306	0.0299
wR(F^2) (I > 2σ(I))	0.0829	0.0830	0.0833
R_1 (all data)	0.0328	0.0317	0.0308
wR(F^2) (all data)	0.0838	0.0837	0.0840
Goodness-of-fit	1.125	1.099	1.101
CCDC deposition number	2093515	2093514	2093513

Table S6. X-ray data for **olefin-I Br** at 230, 210, and 190 K.

compound formula	$C_{14}H_{10}BrI$	$C_{14}H_{10}BrI$	$C_{14}H_{10}BrI$
formula mass	385.03	385.03	385.03
crystal system	Orthorhombic	Orthorhombic	Orthorhombic
space group	*Pccn*	*Pccn*	*Pccn*
a/Å	7.39554(8)	7.37950(7)	7.36421(7)
b/Å	28.3456(3)	28.3436(3)	28.3440(3)
c/Å	5.97312(5)	5.96456(5)	5.95563(5)
α/°	90	90	90
β/°	90	90	90
γ/°	90	90	90
$V/Å^3$	1252.15(2)	1247.557(19)	1243.13(2)
$\rho_{calc}/g\ cm^{-3}$	2.042	2.050	2.057
T/K	230(2)	210(2)	190(2)
Z	4	4	4
radiation type	CuKα	CuKα	CuKα
absorption coefficient, μ/mm^{-1}	23.564	23.651	23.735
no. of reflections measured	14149	14191	14193
no. of independent reflections	1313	1307	1305
no. of reflection ($I > 2\sigma(I)$)	1265	1264	1270
R_{int}	0.0549	0.0570	0.0570
R_1 ($I > 2\sigma(I)$)	0.0297	0.0297	0.0308
$wR(F^2)$ ($I > 2\sigma(I)$)	0.0812	0.0795	0.0802
R_1 (all data)	0.0302	0.0302	0.0312
$wR(F^2)$ (all data)	0.0815	0.0797	0.0804
Goodness-of-fit	1.105	1.135	1.153
CCDC deposition number	2093512	2093511	2093510

Table S7. X-ray data for **imine-I** at 290, 270, and 250 K.

compound formula	$C_{13}H_9I_2N$	$C_{13}H_9I_2N$	$C_{13}H_9I_2N$
formula mass	433.01	433.01	433.01
crystal system	Orthorhombic	Orthorhombic	Orthorhombic
space group	*Pccn*	*Pccn*	*Pccn*
a/Å	7.48800(10)	7.46643(8)	7.44717(12)
b/Å	28.7165(2)	28.7114(2)	28.7099(3)
c/Å	6.02480(10)	6.01595(6)	6.00627(9)
α/°	90	90	90
β/°	90	90	90
γ/°	90	90	90
$V/Å^3$	1295.51(3)	1289.65(2)	1284.18(3)
$\rho_{calc}/g\ cm^{-3}$	2.220	2.230	2.240
T/K	290(2)	270(2)	250(2)
Z	4	4	4
radiation type	CuKα	CuKα	CuKα
absorption coefficient, μ/mm^{-1}	37.899	38.072	38.234
no. of reflections measured	12381	12072	14216
no. of independent reflections	1310	1305	1319
no. of reflection ($I > 2\sigma(I)$)	1084	1107	1145
R_{int}	0.0723	0.0740	0.0728
R_1 ($I > 2\sigma(I)$)	0.0312	0.0295	0.0316
$wR(F^2)$ ($I > 2\sigma(I)$)	0.0838	0.0771	0.0802
R_1 (all data)	0.0372	0.0347	0.0361
$wR(F^2)$ (all data)	0.0905	0.0845	0.0871
Goodness-of-fit	1.027	1.081	1.069
CCDC deposition number	2093491	2093490	2093489

Table S8. X-ray data for **imine-I** at 230, 210, and 190 K.

compound formula	$C_{13}H_9I_2N$	$C_{13}H_9I_2N$	$C_{13}H_9I_2N$
formula mass	433.01	433.01	433.01
crystal system	Orthorhombic	Orthorhombic	Orthorhombic
space group	*Pccn*	*Pccn*	*Pccn*
a/Å	7.42795(8)	7.40853(8)	7.38838(8)
b/Å	28.7031(2)	28.6988(2)	28.6946(2)
c/Å	5.99925(6)	5.99136(5)	5.98258(6)
α/°	90	90	90
β/°	90	90	90
γ/°	90	90	90
$V/Å^3$	1279.07(2)	1273.86(2)	1268.35(2)
$\rho_{calc}/g\ cm^{-3}$	2.249	2.258	2.268
T/K	230(2)	210(2)	190(2)
Z	4	4	4
radiation type	CuKα	CuKα	CuKα
absorption coefficient, μ/mm^{-1}	38.386	38.544	38.711
no. of reflections measured	11778	14295	14566
no. of independent reflections	1326	1327	1322
no. of reflection (I > 2σ(I))	1151	1179	1193
R_{int}	0.0746	0.0719	0.0717
R_1 (I > 2σ(I))	0.0304	0.0271	0.0291
$wR(F^2)$ (I > 2σ(I))	0.0766	0.0710	0.0770
R_1 (all data)	0.0357	0.0319	0.0329
$wR(F^2)$ (all data)	0.0840	0.0784	0.0844
Goodness-of-fit	1.096	1.094	1.105
CCDC deposition number	2093488	2093487	2093486

Table S9. X-ray data for **imine-Br** at 290, 270, and 250 K.

compound formula	$C_{13}H_9Br_2N$	$C_{13}H_9Br_2N$	$C_{13}H_9Br_2N$
formula mass	339.03	339.03	339.03
crystal system	Monoclinic	Monoclinic	Monoclinic
space group	$P2_1/c$	$P2_1/c$	$P2_1/c$
a/Å	4.04957(9)	4.03549(11)	4.02157(7)
b/Å	5.89465(11)	5.88966(12)	5.88488(9)
c/Å	24.9465(5)	24.9442(5)	24.9463(4)
α/°	90	90	90
β/°	92.5589(18)	92.566(2)	92.5537(14)
γ/°	90	90	90
V/Å^3	594.90(2)	592.27(2)	589.805(16)
ρ_{calc}/g cm^{-3}	1.893	1.901	1.909
T/K	290(2)	270(2)	250(2)
Z	2	2	2
radiation type	CuKα	CuKα	CuKα
absorption coefficient, μ/mm^{-1}	8.387	8.424	8.460
no. of reflections measured	9624	9304	9935
no. of independent reflections	1212	1200	1213
no. of reflection (I > 2σ(I))	1056	1078	1103
R_{int}	0.0469	0.0487	0.0499
R_1 (I > 2σ(I))	0.0262	0.0281	0.0272
wR(F^2) (I > 2σ(I))	0.0690	0.0715	0.0697
R_1 (all data)	0.0305	0.0310	0.0296
wR(F^2) (all data)	0.0719	0.0732	0.0708
Goodness-of-fit	1.065	1.109	1.101
CCDC deposition number	2093497	2093496	2093495

Table S10. X-ray data for **imine-Br** at 230, 210, and 190 K.

compound formula	$C_{13}H_9Br_2N$	$C_{13}H_9Br_2N$	$C_{13}H_9Br_2N$
formula mass	339.03	339.03	339.03
crystal system	Monoclinic	Monoclinic	Monoclinic
space group	$P2_1/c$	$P2_1/c$	$P2_1/c$
a/Å	4.00914(7)	3.99682(7)	3.98510(7)
b/Å	5.88041(8)	5.87524(8)	5.86861(9)
c/Å	24.9488(4)	24.9435(4)	24.9445(4)
α/°	90	90	90
β/°	92.5377(13)	92.5186(13)	92.4943(15)
γ/°	90	90	90
V/Å^3	587.602(15)	585.164(15)	582.823(17)
ρ_{calc}/g cm^{-3}	1.916	1.924	1.932
T/K	230(2)	210(2)	190(2)
Z	2	2	2
radiation type	CuKα	CuKα	CuKα
absorption coefficient, μ/mm^{-1}	8.491	8.527	8.561
no. of reflections measured	9983	10335	10198
no. of independent reflections	1209	1209	1208
no. of reflection (I > 2σ(I))	1101	1122	1123
R_{int}	0.0459	0.0428	0.0453
R_1 (I > 2σ(I))	0.0255	0.0251	0.0249
wR(F^2) (I > 2σ(I))	0.0666	0.0659	0.0678
R_1 (all data)	0.0279	0.0272	0.0269
wR(F^2) (all data)	0.0682	0.0673	0.0691
Goodness-of-fit	1.087	1.081	1.096
CCDC deposition number	2093494	2093493	2093492

Table S11. X-ray data for **azo-I** at 290, 270, and 250 K.

compound formula	$C_{12}H_8I_2N_2$	$C_{12}H_8I_2N_2$	$C_{12}H_8I_2N_2$
formula mass	434.00	434.00	434.00
crystal system	Orthorhombic	Orthorhombic	Orthorhombic
space group	*Pccn*	*Pccn*	*Pccn*
a/Å	7.44541(17)	7.4262(2)	7.4085(2)
b/Å	28.3989(5)	28.3821(6)	28.3671(6)
c/Å	6.04700(12)	6.03869(15)	6.03045(15)
α/°	90	90	90
β/°	90	90	90
γ/°	90	90	90
V/Å^3	1278.59(4)	1272.78(5)	1267.34(5)
ρ_{calc}/g cm^{-3}	2.255	2.265	2.275
T/K	290(2)	270(2)	250(2)
Z	4	4	4
radiation type	CuKα	CuKα	CuKα
absorption coefficient, μ/mm^{-1}	38.427	38.602	38.768
no. of reflections measured	15122	15532	15507
no. of independent reflections	1334	1326	1324
no. of reflection (I > 2σ(I))	1175	1206	1208
R_{int}	0.0593	0.0577	0.0525
R_1 (I > 2σ(I))	0.0212	0.0219	0.0199
wR(F^2) (I > 2σ(I))	0.0562	0.0574	0.0509
R_1 (all data)	0.0246	0.0244	0.0221
wR(F^2) (all data)	0.0581	0.0589	0.0523
Goodness-of-fit	1.056	1.067	1.058
CCDC deposition number	2093411	2093410	2093409

Table S12. X-ray data for **azo-I** at 230, 210, and 190 K.

compound formula	$C_{12}H_8I_2N_2$	$C_{12}H_8I_2N_2$	$C_{12}H_8I_2N_2$
formula mass	434.00	434.00	434.00
crystal system	Orthorhombic	Orthorhombic	Orthorhombic
space group	*Pccn*	*Pccn*	*Pccn*
a/Å	7.39123(10)	7.37426(12)	7.35645(13)
b/Å	28.3542(3)	28.3456(4)	28.3296(4)
c/Å	6.02553(8)	6.01735(9)	6.01030(9)
α/°	90	90	90
β/°	90	90	90
γ/°	90	90	90
$V/Å^3$	1262.78(3)	1257.79(3)	1252.58(3)
$\rho_{calc}/g\ cm^{-3}$	2.283	2.292	2.301
T/K	230(2)	210(2)	190(2)
Z	4	4	4
radiation type	CuKα	CuKα	CuKα
absorption coefficient, μ/mm^{-1}	38.908	39.062	39.225
no. of reflections measured	14463	14857	15083
no. of independent reflections	1310	1304	1304
no. of reflection ($I > 2\sigma(I)$)	1203	1228	1237
R_{int}	0.0500	0.0525	0.0470
R_1 ($I > 2\sigma(I)$)	0.0214	0.0258	0.0203
$wR(F^2)$ ($I > 2\sigma(I)$)	0.0604	0.0722	0.0549
R_1 (all data)	0.0228	0.0270	0.0216
$wR(F^2)$ (all data)	0.0613	0.0731	0.0557
Goodness-of-fit	1.084	1.105	1.136
CCDC deposition number	2093408	2093407	2093406

Table S13. X-ray data for **azo-Br**(a) at 290, 270, and 250 K.

compound formula	$C_{12}H_8Br_2N_2$	$C_{12}H_8Br_2N_2$	$C_{12}H_8Br_2N_2$
formula mass	340.02	340.02	340.02
crystal system	Monoclinic	Monoclinic	Monoclinic
space group	$P2_1/c$	$P2_1/c$	$P2_1/c$
a/Å	3.99210(10)	3.98150(10)	3.97160(10)
b/Å	5.87440(10)	5.86860(10)	5.86160(10)
c/Å	24.6890(5)	24.6795(5)	24.6711(5)
α/°	90	90	90
β/°	92.925(2)	92.925(2)	92.918(2)
γ/°	90	90	90
V/Å^3	578.23(2)	575.91(2)	573.60(2)
ρ_{calc}/g cm^{-3}	1.953	1.961	1.969
T/K	290.05(10)	270.05(10)	250.05(10)
Z	2	2	2
radiation type	CuKα	CuKα	CuKα
absorption coefficient, μ/mm^{-1}	8.657	8.692	8.727
no. of reflections measured	8454	9184	8668
no. of independent reflections	1153	1156	1148
no. of reflection (I > 2σ(I))	1130	1140	1130
R_{int}	0.0546	0.0518	0.0516
R_1 (I > 2σ(I))	0.0333	0.0310	0.0306
wR(F^2) (I > 2σ(I))	0.0764	0.0720	0.0698
R_1 (all data)	0.0344	0.0317	0.0312
wR(F^2) (all data)	0.0770	0.0724	0.0701
Goodness-of-fit	1.217	1.250	1.213
CCDC deposition number	2093417	2093416	2093415

Table S14. X-ray data for **azo-Br**(a) at 230, 210, and 190 K.

compound formula	$C_{12}H_8Br_2N_2$	$C_{12}H_8Br_2N_2$	$C_{12}H_8Br_2N_2$
formula mass	340.02	340.02	340.02
crystal system	Monoclinic	Monoclinic	Monoclinic
space group	$P2_1/c$	$P2_1/c$	$P2_1/c$
a/Å	3.96190(10)	3.95240(10)	3.94400(10)
b/Å	5.85570(10)	5.85030(10)	5.84400(10)
c/Å	24.6640(5)	24.6570(5)	24.6497(5)
α/°	90	90	90
β/°	92.921(2)	92.925(2)	92.924(2)
γ/°	90	90	90
V/Å^3	571.45(2)	569.39(2)	567.40(2)
ρ_{calc}/g cm^{-3}	1.976	1.983	1.990
T/K	230.05(10)	210.05(10)	189(2)
Z	2	2	2
radiation type	CuKα	CuKα	CuKα
absorption coefficient, μ/mm^{-1}	8.760	8.792	8.823
no. of reflections measured	7954	8549	8633
no. of independent reflections	1129	1132	1138
no. of reflection (I > 2σ(I))	1109	1111	1122
R_{int}	0.0479	0.0484	0.0467
R_1 (I > 2σ(I))	0.0295	0.0285	0.0267
wR(F^2) (I > 2σ(I))	0.0684	0.0661	0.0657
R_1 (all data)	0.0303	0.0296	0.0273
wR(F^2) (all data)	0.0688	0.0678	0.0661
Goodness-of-fit	1.218	1.216	1.200
CCDC deposition number	2093414	2093413	2093412

Table S15. X-ray data for **azo-Br**(b) at 290, 270, and 250 K.

compound formula	$C_{12}H_8Br_2N_2$	$C_{12}H_8Br_2N_2$	$C_{12}H_8Br_2N_2$
formula mass	340.02	340.02	340.02
crystal system	Monoclinic	Monoclinic	Monoclinic
space group	$P2_1/c$	$P2_1/c$	$P2_1/c$
a/Å	10.13426(10)	10.12841(8)	10.12356(8)
b/Å	4.76896(6)	4.76413(4)	4.76029(4)
c/Å	11.79529(13)	11.77052(11)	11.74756(11)
α/°	90	90	90
β/°	92.3783(10)	92.3405(8)	92.2993(8)
γ/°	90	90	90
V/Å^3	569.574(11)	567.489(8)	565.671(9)
ρ_{calc}/g cm^{-3}	1.983	1.990	1.996
T/K	290(2)	270(2)	250(2)
Z	2	2	2
radiation type	CuKα	CuKα	CuKα
absorption coefficient, μ/mm^{-1}	8.789	8.821	8.850
no. of reflections measured	9260	11658	12073
no. of independent reflections	1153	1185	1174
no. of reflection ($I > 2\sigma(I)$)	1063	1120	1128
R_{int}	0.0585	0.0529	0.0515
R_1 ($I > 2\sigma(I)$)	0.0255	0.0232	0.0218
$wR(F^2)$ ($I > 2\sigma(I)$)	0.0678	0.0616	0.0605
R_1 (all data)	0.0273	0.0242	0.0227
$wR(F^2)$ (all data)	0.0701	0.0628	0.0616
Goodness-of-fit	1.099	1.085	1.113
CCDC deposition number	2093423	2093422	2093421

Table S16. X-ray data for **azo-Br**(b) at 230, 210, and 190 K.

compound formula	$C_{12}H_8Br_2N_2$	$C_{12}H_8Br_2N_2$	$C_{12}H_8Br_2N_2$
formula mass	340.02	340.02	340.02
crystal system	Monoclinic	Monoclinic	Monoclinic
space group	$P2_1/c$	$P2_1/c$	$P2_1/c$
a/Å	10.11911(8)	10.11287(8)	10.11009(11)
b/Å	4.75720(4)	4.75323(4)	4.75046(5)
c/Å	11.72589(10)	11.70238(10)	11.68261(13)
α/°	90	90	90
β/°	92.2614(7)	92.2133(7)	92.1740(10)
γ/°	90	90	90
V/Å^3	564.029(8)	562.100(8)	560.683(11)
ρ_{calc}/g cm^{-3}	2.002	2.009	2.014
T/K	230(2)	210(2)	190(2)
Z	2	2	2
radiation type	CuKα	CuKα	CuKα
absorption coefficient, μ/mm^{-1}	8.875	8.906	8.928
no. of reflections measured	12835	12540	12612
no. of independent reflections	1176	1174	1173
no. of reflection (I > 2σ(I))	1128	1136	1133
R_{int}	0.0497	0.0498	0.0521
R_1 (I > 2σ(I))	0.0211	0.0208	0.0227
wR(F^2) (I > 2σ(I))	0.0578	0.0552	0.0611
R_1 (all data)	0.0218	0.0214	0.0232
wR(F^2) (all data)	0.0585	0.0558	0.0615
Goodness-of-fit	1.086	0.999	1.109
CCDC deposition number	2093420	2093419	2093418

Table S17. X-ray data for **azo-I Br** at 290, 270, and 250 K.

compound formula	$C_{12}H_8BrIN_2$	$C_{12}H_8BrIN_2$	$C_{12}H_8BrIN_2$
formula mass	387.01	387.01	387.01
crystal system	Monoclinic	Monoclinic	Monoclinic
space group	$P2_1/c$	$P2_1/c$	$P2_1/c$
a/Å	4.08885(7)	4.07618(7)	4.06541(8)
b/Å	5.88027(10)	5.87432(10)	5.86837(11)
c/Å	25.2697(4)	25.2598(4)	25.2537(5)
α/°	90	90	90
β/°	93.9641(16)	93.9706(15)	93.9691(19)
γ/°	90	90	90
V/Å^3	606.121(18)	603.389(17)	601.04(2)
ρ_{calc}/g cm^{-3}	2.121	2.130	2.138
T/K	290(2)	270(2)	250(2)
Z	2	2	2
radiation type	CuKα	CuKα	CuKα
absorption coefficient, μ/mm^{-1}	24.394	24.505	24.601
no. of reflections measured	9775	9721	9283
no. of independent reflections	1253	1243	1236
no. of reflection (I > 2σ(I))	1162	1166	1164
R_{int}	0.0757	0.0777	0.0695
R_1 (I > 2σ(I))	0.0284	0.0277	0.0271
wR(F^2) (I > 2σ(I))	0.0710	0.0679	0.0668
R_1 (all data)	0.0301	0.0292	0.0282
wR(F^2) (all data)	0.0722	0.0693	0.0675
Goodness-of-fit	1.082	1.092	1.096
CCDC deposition number	2093429	2093428	2093427

Table S18. X-ray data for **azo-I Br** at 230, 210, and 190 K.

compound formula	$C_{12}H_8BrIN_2$	$C_{12}H_8BrIN_2$	$C_{12}H_8BrIN_2$
formula mass	387.01	387.01	387.01
crystal system	Monoclinic	Monoclinic	Monoclinic
space group	$P2_1/c$	$P2_1/c$	$P2_1/c$
a/Å	4.05483(5)	4.04385(4)	4.0347(3)
b/Å	5.86325(7)	5.85762(6)	5.8485(4)
c/Å	25.2481(3)	25.2411(3)	25.2404(18)
α/°	90	90	90
β/°	93.9639(11)	93.9650(10)	93.994(7)
γ/°	90	90	90
V/Å^3	598.825(12)	596.463(10)	594.16(7)
ρ_{calc}/g cm^{-3}	2.146	2.155	2.163
T/K	230(2)	210(2)	190(2)
Z	2	2	2
radiation type	CuKα	CuKα	CuKα
absorption coefficient, μ/mm^{-1}	24.692	24.789	24.886
no. of reflections measured	10454	12725	12519
no. of independent reflections	1236	1227	1223
no. of reflection ($I > 2\sigma(I)$)	1166	1171	1146
R_{int}	0.0650	0.0663	0.0702
R_1 ($I > 2\sigma(I)$)	0.0228	0.0216	0.0260
$wR(F^2)$ ($I > 2\sigma(I)$)	0.0581	0.0549	0.0656
R_1 (all data)	0.0240	0.0230	0.0284
$wR(F^2)$ (all data)	0.0591	0.0568	0.0679
Goodness-of-fit	1.100	1.058	1.250
CCDC deposition number	2093426	2093425	2093424

Table S19. X-ray data for **diolefin-I** at 290, 270, and 250 K.

compound formula	$C_{22}H_{16}I_2$	$C_{22}H_{16}I_2$	$C_{22}H_{16}I_2$
formula mass	534.15	534.15	534.15
crystal system	Orthorhombic	Orthorhombic	Orthorhombic
space group	*Pccn*	*Pccn*	*Pccn*
a/Å	7.48873(11)	7.46583(10)	7.4616(6)
b/Å	40.7467(3)	40.7716(3)	40.8296(16)
c/Å	5.99117(6)	5.98213(5)	5.9716(3)
α/°	90	90	90
β/°	90	90	90
γ/°	90	90	90
V/Å^3	1828.15(4)	1820.92(3)	1819.3(2)
ρ_{calc}/g cm^{-3}	1.941	1.948	1.950
T/K	290(2)	270(2)	250(2)
Z	4	4	4
radiation type	CuKα	CuKα	CuKα
absorption coefficient, μ/mm^{-1}	26.997	27.105	27.129
no. of reflections measured	29386	29758	15862
no. of independent reflections	1903	1894	1884
no. of reflection ($I > 2\sigma(I)$)	1683	1719	1684
R_{int}	0.0592	0.0564	0.0544
R_1 ($I > 2\sigma(I)$)	0.0324	0.0294	0.0311
wR(F^2) ($I > 2\sigma(I)$)	0.0915	0.0801	0.0837
R_1 (all data)	0.0364	0.0325	0.0347
wR(F^2) (all data)	0.0952	0.0826	0.0861
Goodness-of-fit	1.045	1.107	1.091
CCDC deposition number	2093473	2093472	2093471

Table S20. X-ray data for **diolefin-I** at 230, 210, and 190 K.

compound formula	$C_{22}H_{16}I_2$	$C_{22}H_{16}I_2$	$C_{22}H_{16}I_2$
formula mass	534.15	534.15	534.15
crystal system	Orthorhombic	Orthorhombic	Orthorhombic
space group	*Pccn*	*Pccn*	*Pccn*
a/Å	7.42486(9)	7.40722(9)	7.39117(10)
b/Å	40.8040(3)	40.8177(3)	40.8276(4)
c/Å	5.96406(5)	5.95673(5)	5.94846(5)
α/°	90	90	90
β/°	90	90	90
γ/°	90	90	90
$V/Å^3$	1806.90(3)	1800.99(3)	1795.03(3)
$\rho_{calc}/g\ cm^{-3}$	1.964	1.970	1.977
T/K	230(2)	210(2)	190(2)
Z	4	4	4
radiation type	CuKα	CuKα	CuKα
absorption coefficient, μ/mm^{-1}	27.315	27.405	27.496
no. of reflections measured	30029	30321	29838
no. of independent reflections	1891	1881	1875
no. of reflection ($I > 2\sigma(I)$)	1758	1762	1765
R_{int}	0.0575	0.0559	0.0550
R_1 ($I > 2\sigma(I)$)	0.0261	0.0242	0.0250
$wR(F^2)$ ($I > 2\sigma(I)$)	0.0691	0.0634	0.0675
R_1 (all data)	0.0284	0.0257	0.0263
$wR(F^2)$ (all data)	0.0712	0.0645	0.0684
Goodness-of-fit	1.049	0.997	1.084
CCDC deposition number	2093470	2093469	2093468

Table S21. X-ray data for **diolefin-Br** at 290, 270, and 250 K.

compound formula	$C_{22}H_{16}Br_2$	$C_{22}H_{16}Br_2$	$C_{22}H_{16}Br_2$
formula mass	440.17	440.17	440.17
crystal system	Orthorhombic	Orthorhombic	Orthorhombic
space group	*Pccn*	*Pccn*	*Pccn*
a/Å	7.44909(8)	7.42903(7)	7.41155(7)
b/Å	39.4937(4)	39.5057(3)	39.5112(3)
c/Å	5.93597(6)	5.92607(5)	5.91607(5)
α/°	90	90	90
β/°	90	90	90
γ/°	90	90	90
V/Å^3	1746.31(3)	1739.23(3)	1732.46(3)
ρ_{calc}/g cm^{-3}	1.674	1.681	1.688
T/K	290(2)	270(2)	250(2)
Z	4	4	4
radiation type	CuKα	CuKα	CuKα
absorption coefficient, μ/mm^{-1}	5.861	5.885	5.908
no. of reflections measured	29923	34371	34382
no. of independent reflections	1843	1840	1829
no. of reflection ($I > 2\sigma(I)$)	1641	1680	1697
R_{int}	0.0498	0.0482	0.0467
R_1 ($I > 2\sigma(I)$)	0.0263	0.0238	0.0218
$wR(F^2)$ ($I > 2\sigma(I)$)	0.0679	0.0644	0.0607
R_1 (all data)	0.0293	0.0259	0.0233
$wR(F^2)$ (all data)	0.0700	0.0659	0.0617
Goodness-of-fit	1.115	1.065	1.078
CCDC deposition number	2093479	2093478	2093477

Table S22. X-ray data for **diolefin-Br** at 230, 210, and 190 K.

compound formula	$C_{22}H_{16}Br_2$	$C_{22}H_{16}Br_2$	$C_{22}H_{16}Br_2$
formula mass	440.17	440.17	440.17
crystal system	Orthorhombic	Orthorhombic	Orthorhombic
space group	*Pccn*	*Pccn*	*Pccn*
a/Å	7.39509(6)	7.37918(6)	7.36426(6)
b/Å	39.5130(3)	39.5129(3)	39.5134(3)
c/Å	5.90727(4)	5.89885(4)	5.89107(4)
α/°	90	90	90
β/°	90	90	90
γ/°	90	90	90
V/Å^3	1726.12(2)	1719.95(2)	1714.22(2)
ρ_{calc}/g cm^{-3}	1.694	1.700	1.706
T/K	230(2)	210(2)	190(2)
Z	4	4	4
radiation type	CuKα	CuKα	CuKα
absorption coefficient, μ/mm^{-1}	5.930	5.951	5.971
no. of reflections measured	34297	34071	34953
no. of independent reflections	1825	1817	1816
no. of reflection (I > 2σ(I))	1702	1713	1736
R_{int}	0.0463	0.0473	0.0413
R_1 (I > 2σ(I))	0.0208	0.0201	0.0201
wR(F^2) (I > 2σ(I))	0.0602	0.0571	0.0555
R_1 (all data)	0.0221	0.0212	0.0209
wR(F^2) (all data)	0.0611	0.0578	0.0560
Goodness-of-fit	1.092	1.083	1.081
CCDC deposition number	2093476	2093475	2093474

Table S23. X-ray data for **diolefin-I Br** at 290, 270, and 250 K.

compound formula	$C_{22}H_{16}BrI$	$C_{22}H_{16}BrI$	$C_{22}H_{16}BrI$
formula mass	487.16	487.16	487.16
crystal system	Orthorhombic	Orthorhombic	Orthorhombic
space group	*Pccn*	*Pccn*	*Pccn*
a/Å	7.47790(10)	7.45535(7)	7.43466(7)
b/Å	40.1339(4)	40.1523(3)	40.1695(3)
c/Å	5.97100(10)	5.96072(4)	5.95124(4)
α/°	90	90	90
β/°	90	90	90
γ/°	90	90	90
V/Å^3	1792.00(4)	1784.34(3)	1777.32(3)
ρ_{calc}/g cm^{-3}	1.806	1.813	1.821
T/K	290.04(10)	270.04(10)	250.04(10)
Z	4	4	4
radiation type	CuKα	CuKα	CuKα
absorption coefficient, μ/mm^{-1}	16.627	16.698	16.764
no. of reflections measured	31558	31801	31607
no. of independent reflections	1903	1894	1886
no. of reflection ($I > 2\sigma(I)$)	1724	1754	1760
R_{int}	0.0491	0.0476	0.0459
R_1 ($I > 2\sigma(I)$)	0.0224	0.0222	0.0211
wR(F^2) ($I > 2\sigma(I)$)	0.0548	0.0535	0.0521
R_1 (all data)	0.0249	0.0243	0.0226
wR(F^2) (all data)	0.0561	0.0545	0.0529
Goodness-of-fit	1.065	1.115	1.089
CCDC deposition number	2093485	2093484	2093483

Table S24. X-ray data for **diolefin-I Br** at 230, 210, and 190 K.

compound formula	$C_{22}H_{16}BrI$	$C_{22}H_{16}BrI$	$C_{22}H_{16}BrI$
formula mass	487.16	487.16	487.16
crystal system	Orthorhombic	Orthorhombic	Orthorhombic
space group	*Pccn*	*Pccn*	*Pccn*
a/Å	7.41588(6)	7.39817(7)	7.38099(11)
b/Å	40.1789(3)	40.1869(3)	40.1967(6)
c/Å	5.94234(4)	5.93303(4)	5.92440(6)
α/°	90	90	90
β/°	90	90	90
γ/°	90	90	90
V/Å^3	1770.59(2)	1763.95(3)	1757.72(4)
ρ_{calc}/g cm^{-3}	1.828	1.834	1.841
T/K	230.04(10)	210.04(10)	190.00(10)
Z	4	4	4
radiation type	CuKα	CuKα	CuKα
absorption coefficient, μ/mm^{-1}	16.828	16.891	16.951
no. of reflections measured	31594	31477	30395
no. of independent reflections	1880	1870	1860
no. of reflection (I > 2σ(I))	1774	1781	1728
R_{int}	0.0466	0.0448	0.0574
R_1 (I > 2σ(I))	0.0216	0.0212	0.0264
wR(F^2) (I > 2σ(I))	0.0544	0.0545	0.0665
R_1 (all data)	0.0230	0.0222	0.0287
wR(F^2) (all data)	0.0552	0.0550	0.0681
Goodness-of-fit	1.060	1.127	1.077
CCDC deposition number	2093482	2093481	2093480

Table S25. X-ray data for **diimine-I** at 290, 270, and 250 K.

compound formula	$C_{20}H_{14}I_2N_2$	$C_{20}H_{14}I_2N_2$	$C_{20}H_{14}I_2N_2$
formula mass	536.13	536.13	536.13
crystal system	Monoclinic	Monoclinic	Monoclinic
space group	$P2_1/c$	$P2_1/c$	$P2_1/c$
a/Å	7.51877(11)	7.4993(3)	7.48192(10)
b/Å	40.4776(5)	40.4748(15)	40.4930(5)
c/Å	5.91959(8)	5.9105(2)	5.90037(7)
α/°	90	90	90
β/°	90.5114(13)	90.496(4)	90.4998(12)
γ/°	90	90	90
V/Å^3	1801.51(4)	1793.94(12)	1787.54(4)
ρ_{calc}/g cm^{-3}	1.977	1.985	1.992
T/K	290(2)	270(2)	250(2)
Z	4	4	4
radiation type	CuKα	CuKα	CuKα
absorption coefficient, μ/mm^{-1}	27.433	27.549	27.648
no. of reflections measured	34816	28131	34531
no. of independent reflections	3780	3719	3759
no. of reflection ($I > 2\sigma(I)$)	3199	3206	3359
R_{int}	0.0906	0.0802	0.0855
R_1 ($I > 2\sigma(I)$)	0.0487	0.0345	0.0363
wR(F^2) ($I > 2\sigma(I)$)	0.1245	0.0884	0.0932
R_1 (all data)	0.0565	0.0408	0.0402
wR(F^2) (all data)	0.1326	0.0941	0.0968
Goodness-of-fit	1.054	1.042	1.029
CCDC deposition number	2093460	2093459	2093458

Table S26. X-ray data for **diimine-I** at 230, 210, and 190 K.

compound formula	$C_{20}H_{14}I_2N_2$	$C_{20}H_{14}I_2N_2$	$C_{20}H_{14}I_2N_2$
formula mass	536.13	536.13	536.13
crystal system	Monoclinic	Monoclinic	Monoclinic
space group	$P2_1/c$	$P2_1/c$	$P2_1/c$
a/Å	7.46346(10)	7.44656(9)	7.42990(9)
b/Å	40.4985(5)	40.5029(5)	40.5096(5)
c/Å	5.89180(7)	5.88383(6)	5.87627(7)
α/°	90	90	90
β/°	90.5291(11)	90.5288(11)	90.5291(11)
γ/°	90	90	90
V/Å^3	1780.78(4)	1774.53(4)	1768.58(4)
ρ_{calc}/g cm^{-3}	2.000	2.007	2.014
T/K	230(2)	210(2)	190(2)
Z	4	4	4
radiation type	CuKα	CuKα	CuKα
absorption coefficient, μ/mm^{-1}	27.753	27.850	27.944
no. of reflections measured	34599	34350	30569
no. of independent reflections	3729	3719	3699
no. of reflection ($I > 2\sigma(I)$)	3388	3408	3412
R_{int}	0.0793	0.0778	0.0700
R_1 ($I > 2\sigma(I)$)	0.0377	0.0378	0.0383
wR(F^2) ($I > 2\sigma(I)$)	0.0947	0.0980	0.1004
R_1 (all data)	0.0410	0.0408	0.0411
wR(F^2) (all data)	0.0977	0.1014	0.1041
Goodness-of-fit	1.066	1.034	1.070
CCDC deposition number	2093457	2093456	2093455

Table S27. X-ray data for **diimine-Br** at 290, 270, and 250 K.

compound formula	$C_{20}H_{14}Br_2N_2$	$C_{20}H_{14}Br_2N_2$	$C_{20}H_{14}Br_2N_2$
formula mass	442.15	442.15	442.15
crystal system	Monoclinic	Monoclinic	Monoclinic
space group	$P2_1/c$	$P2_1/c$	$P2_1/c$
a/Å	7.48669(12)	7.46626(15)	7.44522(10)
b/Å	38.8999(4)	38.9089(5)	38.9215(4)
c/Å	5.93712(7)	5.92704(9)	5.91739(6)
α/°	90	90	90
β/°	90.4022(13)	90.4136(17)	90.4009(11)
γ/°	90	90	90
V/Å^3	1729.03(4)	1721.78(5)	1714.69(3)
ρ_{calc}/g cm^{-3}	1.699	1.706	1.713
T/K	290(2)	270(2)	250(2)
Z	4	4	4
radiation type	CuKα	CuKα	CuKα
absorption coefficient, μ/mm^{-1}	5.958	5.983	6.008
no. of reflections measured	39643	36190	39875
no. of independent reflections	3607	3585	3585
no. of reflection (I > 2σ(I))	3065	2976	3139
R_{int}	0.0768	0.0767	0.0795
R_1 (I > 2σ(I))	0.0468	0.0516	0.0498
wR(F^2) (I > 2σ(I))	0.1237	0.1407	0.1373
R_1 (all data)	0.0528	0.0589	0.0545
wR(F^2) (all data)	0.1271	0.1457	0.1400
Goodness-of-fit	1.087	1.085	1.063
CCDC deposition number	2093466	2093465	2093464

Table S28. X-ray data for **diimine-Br** at 230, 210, and 190 K.

compound formula	$C_{20}H_{14}Br_2N_2$	$C_{20}H_{14}Br_2N_2$	$C_{20}H_{14}Br_2N_2$
formula mass	442.15	442.15	442.15
crystal system	Monoclinic	Monoclinic	Monoclinic
space group	$P2_1/c$	$P2_1/c$	$P2_1/c$
a/Å	7.42568(9)	7.40704(9)	7.38842(9)
b/Å	38.9298(4)	38.9343(3)	38.9510(4)
c/Å	5.90945(6)	5.90140(6)	5.89251(5)
α/°	90	90	90
β/°	90.4212(10)	90.4197(10)	90.4280(10)
γ/°	90	90	90
V/Å^3	1708.26(3)	1701.85(3)	1695.74(3)
ρ_{calc}/g cm^{-3}	1.719	1.726	1.732
T/K	230(2)	210(2)	190(2)
Z	4	4	4
radiation type	CuKα	CuKα	CuKα
absorption coefficient, μ/mm^{-1}	6.030	6.053	6.075
no. of reflections measured	39709	39615	38733
no. of independent reflections	3567	3552	3537
no. of reflection (I > 2σ(I))	3194	3225	3238
R_{int}	0.0756	0.0734	0.0716
R_1 (I > 2σ(I))	0.0504	0.0481	0.0527
wR(F^2) (I > 2σ(I))	0.1416	0.1358	0.1294
R_1 (all data)	0.0540	0.0511	0.0552
wR(F^2) (all data)	0.1435	0.1373	0.1305
Goodness-of-fit	1.103	1.101	1.093
CCDC deposition number	2093463	2093462	2093461

Table S29. X-ray data for **diazo-I** at 290, 270, and 250 K.

compound formula	$C_{18}H_{12}I_2N_4$	$C_{18}H_{12}I_2N_4$	$C_{18}H_{12}I_2N_4$
formula mass	538.12	538.12	538.12
crystal system	Monoclinic	Monoclinic	Monoclinic
space group	$P2_1/c$	$P2_1/c$	$P2_1/c$
a/Å	7.34930(10)	7.31740(10)	7.31514(13)
b/Å	40.3729(4)	40.4302(4)	40.2986(5)
c/Å	5.96770(10)	5.96030(10)	5.95370(13)
α/°	90	90	90
β/°	90.0060(10)	90.0660(10)	90.6425(19)
γ/°	90	90	90
V/Å^3	1770.69(4)	1763.32(4)	1754.98(5)
ρ_{calc}/g cm^{-3}	2.019	2.027	2.037
T/K	290.05(10)	270.05(10)	250.05(10)
Z	4	4	4
radiation type	CuKα	CuKα	CuKα
absorption coefficient, μ/mm^{-1}	27.948	28.065	28.198
no. of reflections measured	24753	26033	21486
no. of independent reflections	3130	3130	3111
no. of reflection (I > 2σ(I))	2216	2428	2603
R_{int}	0.0624	0.0579	0.0605
R_1 (I > 2σ(I))	0.0400	0.0401	0.0537
wR(F^2) (I > 2σ(I))	0.1057	0.0884	0.1385
R_1 (all data)	0.0577	0.0523	0.0623
wR(F^2) (all data)	0.1151	0.0939	0.1476
Goodness-of-fit	1.083	1.056	1.062
CCDC deposition number	2093442	2093441	2093440

Table S30. X-ray data for **diazo-I** at 230, 210, and 190 K.

compound formula	$C_{18}H_{12}I_2N_4$	$C_{18}H_{12}I_2N_4$	$C_{18}H_{12}I_2N_4$
formula mass	538.12	538.12	538.12
crystal system	Monoclinic	Monoclinic	Monoclinic
space group	$P2_1/c$	$P2_1/c$	$P2_1/c$
a/Å	7.30390(10)	7.28910(10)	7.27480(10)
b/Å	40.2474(5)	40.2211(5)	40.2019(5)
c/Å	5.94590(10)	5.93830(10)	5.93060(10)
α/°	90	90	90
β/°	90.785(2)	90.858(2)	90.9050(10)
γ/°	90	90	90
V/Å^3	1747.71(4)	1740.77(4)	1734.25(4)
ρ_{calc}/g cm^{-3}	2.045	2.053	2.061
T/K	230.05(10)	210.05(10)	190.02(10)
Z	4	4	4
radiation type	CuKα	CuKα	CuKα
absorption coefficient, μ/mm^{-1}	28.315	28.428	28.535
no. of reflections measured	23488	26902	29043
no. of independent reflections	3550	3580	3580
no. of reflection (I > 2σ(I))	3019	3204	3271
R_{int}	0.0568	0.0553	0.0531
R_1 (I > 2σ(I))	0.0472	0.0442	0.0400
wR(F^2) (I > 2σ(I))	0.1189	0.1142	0.1046
R_1 (all data)	0.0549	0.0481	0.0431
wR(F^2) (all data)	0.1255	0.1168	0.1065
Goodness-of-fit	1.077	1.090	1.103
CCDC deposition number	2093439	2093438	2093437

Table S31. X-ray data for **diazo-Br** at 290, 270, and 250 K.

compound formula	$C_{18}H_{12}Br_2N_4$	$C_{18}H_{12}Br_2N_4$	$C_{18}H_{12}Br_2N_4$
formula mass	444.14	444.14	444.14
crystal system	Monoclinic	Monoclinic	Monoclinic
space group	$P2_1/n$	$P2_1/n$	$P2_1/n$
a/Å	3.94658(7)	3.93600(7)	3.92669(6)
b/Å	5.90597(8)	5.90113(9)	5.89602(7)
c/Å	34.9552(5)	34.9507(6)	34.9384(5)
α/°	90	90	90
β/°	91.2515(15)	91.2861(16)	91.3143(13)
γ/°	90	90	90
V/Å^3	814.56(2)	811.59(2)	808.676(19)
ρ_{calc}/g cm^{-3}	1.811	1.817	1.824
T/K	290(2)	270(2)	250(2)
Z	2	2	2
radiation type	CuKα	CuKα	CuKα
absorption coefficient, μ/mm^{-1}	6.364	6.387	6.410
no. of reflections measured	12433	13534	13769
no. of independent reflections	1664	1674	1675
no. of reflection (I > 2σ(I))	1492	1533	1547
R_{int}	0.0519	0.0505	0.0492
R_1 (I > 2σ(I))	0.0279	0.0283	0.0275
wR(F^2) (I > 2σ(I))	0.0731	0.0732	0.0682
R_1 (all data)	0.0309	0.0308	0.0297
wR(F^2) (all data)	0.0751	0.0744	0.0693
Goodness-of-fit	1.113	1.083	1.088
CCDC deposition number	2093448	2093447	2093446

Table S32. X-ray data for **diazo-Br** at 230, 210, and 190 K.

compound formula	$C_{18}H_{12}Br_2N_4$	$C_{18}H_{12}Br_2N_4$	$C_{18}H_{12}Br_2N_4$
formula mass	444.14	444.14	444.14
crystal system	Monoclinic	Monoclinic	Monoclinic
space group	$P2_1/n$	$P2_1/n$	$P2_1/n$
a/Å	3.91829(6)	3.90964(6)	3.90194(10)
b/Å	5.89122(7)	5.88619(7)	5.88142(12)
c/Å	34.9307(5)	34.9231(5)	34.9288(8)
α/°	90	90	90
β/°	91.3238(12)	91.3351(12)	91.343(2)
γ/°	90	90	90
V/Å^3	806.109(18)	803.465(18)	801.36(3)
ρ_{calc}/g cm^{-3}	1.830	1.836	1.841
T/K	230(2)	210(2)	190(2)
Z	2	2	2
radiation type	CuKα	CuKα	CuKα
absorption coefficient, μ/mm^{-1}	6.430	6.452	6.469
no. of reflections measured	14178	14147	12758
no. of independent reflections	1676	1670	1633
no. of reflection (I > 2σ(I))	1563	1569	1526
R_{int}	0.0486	0.0474	0.0572
R_1 (I > 2σ(I))	0.0270	0.0278	0.0414
wR(F^2) (I > 2σ(I))	0.0698	0.0690	0.0939
R_1 (all data)	0.0289	0.0294	0.0435
wR(F^2) (all data)	0.0708	0.0699	0.0947
Goodness-of-fit	1.132	1.090	1.158
CCDC deposition number	2093445	2093444	2093443

Table S33. X-ray data for **diazo-I Br** at 290, 270, and 250 K.

compound formula	$C_{18}H_{12}BrIN_4$	$C_{18}H_{12}BrIN_4$	$C_{18}H_{12}BrIN_4$
formula mass	491.13	491.13	491.13
crystal system	Monoclinic	Monoclinic	Monoclinic
space group	$P2_1/n$	$P2_1/n$	$P2_1/n$
a/Å	4.02175(5)	4.01141(4)	4.00144(4)
b/Å	5.89808(7)	5.89288(7)	5.88842(7)
c/Å	35.5519(4)	35.5462(4)	35.5378(4)
α/°	90	90	90
β/°	92.0206(10)	92.0454(9)	92.0671(9)
γ/°	90	90	90
V/Å^3	842.789(17)	839.734(16)	836.801(16)
ρ_{calc}/g cm^{-3}	1.935	1.942	1.949
T/K	290.05(10)	270.05(10)	250.06(10)
Z	2	2	2
radiation type	CuKα	CuKα	CuKα
absorption coefficient, μ/mm^{-1}	17.755	17.819	17.882
no. of reflections measured	11445	11724	12164
no. of independent reflections	1695	1704	1704
no. of reflection (I > 2σ(I))	1551	1576	1589
R_{int}	0.0494	0.0488	0.0496
R_1 (I > 2σ(I))	0.0263	0.0252	0.0250
wR(F^2) (I > 2σ(I))	0.0640	0.0636	0.0627
R_1 (all data)	0.0297	0.0282	0.0273
wR(F^2) (all data)	0.0667	0.0655	0.0645
Goodness-of-fit	1.063	1.047	1.048
CCDC deposition number	2093454	2093453	2093452

Table S34. X-ray data for **diazo-I Br** at 230, 210, and 190 K.

compound formula	$C_{18}H_{12}BrIN_4$	$C_{18}H_{12}BrIN_4$	$C_{18}H_{12}BrIN_4$
formula mass	491.13	491.13	491.13
crystal system	Monoclinic	Monoclinic	Monoclinic
space group	$P2_1/n$	$P2_1/n$	$P2_1/n$
a/Å	3.99201(4)	3.98303(4)	3.97438(5)
b/Å	5.88369(7)	5.87894(6)	5.87297(7)
c/Å	35.5287(4)	35.5219(4)	35.5177(4)
α/°	90	90	90
β/°	92.0859(9)	92.1041(9)	92.1233(10)
γ/°	90	90	90
V/Å^3	833.936(15)	831.219(15)	828.464(16)
ρ_{calc}/g cm^{-3}	1.956	1.962	1.969
T/K	230.06(10)	210.06(10)	190.00(10)
Z	2	2	2
radiation type	CuKα	CuKα	CuKα
absorption coefficient, μ/mm^{-1}	17.943	18.002	18.062
no. of reflections measured	12503	12542	12264
no. of independent reflections	1706	1712	1701
no. of reflection (I > 2σ(I))	1593	1621	1608
R_{int}	0.0481	0.0482	0.0475
R_1 (I > 2σ(I))	0.0247	0.0246	0.0250
wR(F^2) (I > 2σ(I))	0.0611	0.0620	0.0610
R_1 (all data)	0.0269	0.0259	0.0264
wR(F^2) (all data)	0.0629	0.0629	0.0619
Goodness-of-fit	1.065	1.057	1.054
CCDC deposition number	2093451	2093450	2093449

3. Thermal Expansion Data and Intermolecular Interaction Distances

The TE coefficients were calculated using the PASCal program.[13] The unit cell parameters from the crystallographic data sets collected from 290-190 K were used for the TE calculations.

Table S35. TE coefficients for the crystals organized by motion group. Errors are denoted in parentheses and approximate crystallographic axes are denoted in brackets.

**Crystal**	α_{X_1} **(MK^{-1}) [axis]**	α_{X_2} **(MK^{-1}) [axis]**	α_{X_3}**(MK^{-1}) [axis]**	α_V**(MK^{-1})**
**olefin-I**[a]	-2 (1) [0 1 0]	68 (5) [0 0 1]	128 (1) [-1 0 0]	194 (3)
**olefin-Br**	11 (1) [0 1 0]	83 (2) [0 0 1]	114 (1) [-1 0 0]	209 (2)
**olefin-I Br**	0.5 (0.5) [0 1 0]	75 (1) [0 0 1]	115 (2) [-1 0 0]	191 (2)
**imine-I**	8 (1) [0 -1 0]	70 (1) [0 0 1]	134 (1) [1 0 0]	212 (2)
**imine-Br**	0 (1) [1 0 2]	43 (1) [0 -1 0]	162 (3) [1 0 0]	206 (2)
**azo-I**	24 (1) [0 -1 0]	60 (1) [0 0 1]	120 (1) [1 0 0]	205 (3)
**azo-Br**(a)	16 (1) [1 0 9]	52 (1) [0 -1 0]	122 (2) [1 0 0]	191 (3)
**azo-Br**(b)	19 (1) [5 0 1]	39 (2) [0 -1 0]	100 (1) [-1 0 4]	159 (4)
**azo-I Br**	12 (1) [0 0 1]	52 (2) [0 -1 0]	134 (3) [1 0 0]	199 (3)
**diolefin-I**	-18 (4) [0 -1 0]	71 (1) [0 0 1]	135 (4) [1 0 0]	194 (9)
**diolefin-Br**	-4 (1) [0 1 0]	76 (2) [0 0 1]	114 (3) [-1 0 0]	187 (3)
**diolefin-I Br**	-15 (1) [0 1 0]	78 (1) [0 0 1]	131 (3) [-1 0 0]	195 (3)
**diimine-I**	-9 (1) [0 1 0]	74 (2) [0 0 -1]	120 (1) [-1 0 0]	186 (3)
**diimine-Br**	-12 (1) [0 1 0]	75 (1) [0 0 -1]	133 (1) [-1 0 0]	196 (3)
**diazo-I**[b]	38 (4) [0 1 0]	41 (4) [1 0 -2]	120 (3) [-3 0 -2]	199 (2)
**diazo-Br**	9 (2) [-1 0 4]	42 (1) [0 1 0]	114 (3) [-1 0 0]	166 (4)
**diazo-I Br**	10 (1) [1 0 -2]	42 (1) [0 -1 0]	120 (2) [1 0 0]	173 (2)

[a]Only data from 270-190 K was used for the TE calculations because the data quality at 290 K was low.

[b]Only data from 250-190 K was used for TE calculations because the solid undergoes phase transition between 270 and 250 K due to conformational switching.

Table 36. Distances and angles of halogen···halogen interactions. All values are calculated using the X-ray data at 290 K, except **olefin-I**, which is 270 K.

Crystal	Interaction	Distance (Å)	Angles (°)
**olefin-I**	type II I···I	4.053(7)	$\theta_1 = 164.54$, $\theta_2 = 99.01$ ($\theta_1 - \theta_2 = 65.53$)
	type I I···I	4.153(8)	$\theta_1 = \theta_2 = 127.25$
**olefin-Br**	type II Br···Br	3.875(7)	$\theta_1 = 162.06$, $\theta_2 = 96.39$ ($\theta_1 - \theta_2 = 65.67$)
	type I Br···Br	3.905(7)	$\theta_1 = \theta_2 = 127.57$
**olefin-I Br**	type II I···I	4.010(8)	$\theta_1 = 165.40$, $\theta_2 = 96.54$ ($\theta_1 - \theta_2 = 68.86$)
	type II Br···Br	3.931(1)	$\theta_1 = 160.11$, $\theta_2 = 100.06$ ($\theta_1 - \theta_2 = 60.05$)
	type II I···Br	4.093(1)	$\theta_1 = 159.49$, $\theta_2 = 94.32$ ($\theta_1 - \theta_2 = 65.17$)
		3.852(1)	$\theta_1 = 165.68$, $\theta_2 = 102.35$ ($\theta_1 - \theta_2 = 63.33$)
	type I I···I	4.039(7)	$\theta_1 = \theta_2 = 127.78$
	type I Br···Br	4.084(1)	$\theta_1 = \theta_2 = 125.83$
**imine-I**	type II I···I	4.082(5)	$\theta_1 = 164.41$, $\theta_2 = 100.04$ ($\theta_1 - \theta_2 = 64.37$)
	type I I···I	4.192(5)	$\theta_1 = \theta_2 = 126.97$
**imine-Br**	type II Br···Br	3.844(6)	$\theta_1 = 166.14$, $\theta_2 = 93.18$ ($\theta_1 - \theta_2 = 72.96$)
**azo-I**	type II I···I	4.099(5)	$\theta_1 = 165.02$, $\theta_2 = 99.42$ ($\theta_1 - \theta_2 = 65.60$)
	type I I···I	4.202(4)	$\theta_1 = \theta_2 = 126.88$
**azo-Br**(a)	type II Br···Br	3.842(6)	$\theta_1 = 166.20$, $\theta_2 = 93.34$ ($\theta_1 - \theta_2 = 72.86$)
**azo-Br**(b)	type II Br···Br	3.582(4)	$\theta_1 = 167.16$, $\theta_2 = 95.88$ ($\theta_1 - \theta_2 = 71.28$)
**azo-I Br**	type II I···I	3.879(9)	$\theta_1 = 166.70$, $\theta_2 = 94.05$ ($\theta_1 - \theta_2 = 72.65$)
	type II Br···Br	3.986(1)	$\theta_1 = 166.96$, $\theta_2 = 97.50$ ($\theta_1 - \theta_2 = 69.46$)
	type II I···Br	4.029(1)	$\theta_1 = 164.88$, $\theta_2 = 93.74$ ($\theta_1 - \theta_2 = 71.14$)
		3.836(1)	$\theta_1 = 168.83$, $\theta_2 = 97.90$ ($\theta_1 - \theta_2 = 70.93$)
**diolefin-I**	type II I I···I	4.050 (8)	$\theta_1 = 164.59$, $\theta_2 = 99.34$ ($\theta_1 - \theta_2 = 65.25$)
	type I I···I	4.173 (9)	$\theta_1 = \theta_2 = 127.23$
**diolefin-Br**	type II Br···Br	3.865 (8)	$\theta_1 = 162.42$, $\theta_2 = 96.16$ ($\theta_1 - \theta_2 = 66.26$)
	type I Br···Br	3.909 (8)	$\theta_1 = \theta_2 = 127.31$
**diolefin-I Br**	type II I···I	3.949 (4)	$\theta_1 = 164.01$, $\theta_2 = 96.73$ ($\theta_1 - \theta_2 = 67.28$)
	type II Br···Br	4.006 (6)	$\theta_1 = 162.93$, $\theta_2 = 100.31$ ($\theta_1 - \theta_2 = 62.62$)

	type II I···Br	3.877 (5)	θ_1 = 165.14, θ_2 = 101.26 (θ_1 - θ_2 = 63.88)
		4.081 (5)	θ_1 = 161.44, θ_2 = 95.82 (θ_1 - θ_2 = 65.52)
	type I I···I	4.003 (3)	θ_1 = θ_2 = 127.41
	type I Br···Br	4.183 (6)	θ_1 = θ_2 = 126.24
	type II I I···I	3.951 (8)	θ_1 = 157.42, θ_2 = 105.54 (θ_1 - θ_2 = 51.88)
**diimine-I**		4.135 (9)	θ_1 = 163.58, θ_2 = 104.21 (θ_1 - θ_2 = 59.37)
	type I I···I	4.325 (6)	θ_1 = 127.04, θ_2 = 126.29 (θ_1 - θ_2 = 0.75)
	type II Br···Br	3.721 (9)	θ_1 = 152.65, θ_2 = 101.46 (θ_1 - θ_2 = 51.19)
**diimine-Br**		3.950 (1)	θ_1 = 161.93, θ_2 = 99.67 (θ_1 - θ_2 = 62.26)
	type I Br···Br	4.073 (1)	θ_1 = 126.29, θ_2 = 123.11 (θ_1 - θ_2 = 3.18)
	type II I I···I	4.071 (1)	θ_1 = 158.00, θ_2 = 107.53 (θ_1 - θ_2 = 50.47)
**diazo-I**		4.070 (1)	θ_1 = 155.41, θ_2 = 110.27 (θ_1 - θ_2 = 45.14)
	type I I···I	4.349 (9)	θ_1 = 127.69, θ_2 = 126.93 (θ_1 - θ_2 = 0.76)
**diazo-Br**	type II Br···Br	3.853 (9)	θ_1 = 166.20, θ_2 = 92.96 (θ_1 - θ_2 = 73.24)
	type II I···I	3.847 (8)	θ_1 = 164.74, θ_2 = 95.17 (θ_1 - θ_2 = 69.57)
**diazo-I Br**	type II Br···Br	4.046 (1)	θ_1 = 166.76, θ_2 = 94.56 (θ_1 - θ_2 = 72.20)
	type II I···Br	3.969 (1)	θ_1 = 165.79, θ_2 = 92.97 (θ_1 - θ_2 = 72.82)
		3.915 (1)	θ_1 = 167.49, θ_2 = 97.08 (θ_1 - θ_2 = 70.41)

Table S37. Intermolecular interaction distances that contribute to the TE along X_1.

Crystal	X···X (Å) 290/270/250 K[a]	X···X (Å) 190K	Δ (Å)
**olefin-I**	type II I···I: 4.053<br>type I I···I: 4.153	4.028<br>4.105	0.025<br>0.048
**olefin-Br**	type II Br···Br: 3.875<br>type I Br···Br: 3.905	3.837<br>3.845	0.038<br>0.060
**olefin-I Br**	type II I···I: 4.010<br>type I I···I: 4.039<br>type II Br···Br: 3.931<br>type I Br···Br: 4.084<br>type II I···Br: 4.093; 3.852<br>centroid type II: 3.970<br>centroid type I: 4.061	3.874<br>3.893<br>4.080<br>4.176<br>4.062; 3.890<br>3.975<br>4.033	0.136<br>0.146<br>-0.149<br>-0.092<br>0.031; -0.038 (avg. -0.004)<br>-0.005<br>0.028
**imine-I**	type II I···I: 4.082<br>type I I···I: 4.192	4.027<br>4.113	0.055<br>0.079
**azo-I**	type II I···I: 4.099	4.042	0.057

	type I I···I: 4.202	4.115	0.087
**azo-I Br**	type II I···I: 3.879<br>type II Br···Br: 3.986<br>type II I···Br: 3.836; 4.029<br>centroid type II: 3.932	3.844<br>3.967<br>3.774; 4.032<br>3.901	0.035<br>0.019<br>0.062; -0.003 (avg. 0.030)<br>0.031
**diolefin-I**	type II I···I: 4.050<br>type I I···I: 4.173	4.016<br>4.110	0.034<br>0.063
**diolefin-Br**	type II Br···Br: 3.865<br>type I Br···Br: 3.909	3.829<br>3.849	0.036<br>0.060
**diolefin-I Br**	type II I···I: 3.949<br>type I I···I: 4.003<br>type II Br···Br: 4.006<br>type I Br···Br: 4.183<br>type II I···Br: 4.081; 3.877<br>centroid type II: 3.977<br>centroid type I: 4.093	3.883<br>3.903<br>4.023<br>4.153<br>4.092; 3.817<br>3.952<br>4.028	0.066<br>0.100<br>-0.017<br>0.030<br>-0.011; 0.060 (avg. 0.025)<br>0.025<br>0.065
**diimine-I**	type II I···I: 4.135; 3.951<br>type I I···I: 4.325	4.105; 3.919<br>4.274	0.030; 0.032 (avg. 0.031)<br>0.051
**diimine-Br**	type II Br···Br: 3.721; 3.950<br>type I Br···Br: 4.073	3.686; 3.912<br>4.007	0.035; 0.038 (avg. 0.037)<br>0.066
**diazo-I**	type II I···I: 3.984; 4.146<br>type I: 4.315	3.933; 4.162<br>4.267	0.051; -0.016 (avg. 0.018)<br>0.048

[a]All the bond length was calculated using the X-ray data at 290 K, except **olefin-I**, which is at 270 K, and **diazo-I**, which is at 250 K.

Crystal	C-H···X (Å)<br>290K	C-H···X (Å)<br>190K	Δ (Å)
**imine-Br**	C-H···Br: 4.021	3.985	0.036
**azo-Br**(a)	C-H···Br: 4.008	3.975	0.033
**azo-Br**(b)	C-H···Br: 4.150	4.098	0.052
**azo-I Br**	C-H···I: 4.112<br>C-H···Br: 4.173<br>C-H···centroid I/Br: 4.142	4.013<br>4.215<br>4.113	0.099<br>-0.042<br>0.029
**diazo-Br**	C-H···Br: 3.993	3.962	0.031
**diazo-I Br**	C-H···I: 4.185<br>C-H···Br: 4.292<br>C-H···centroid I/Br: 4.237	4.229<br>4.131<br>4.179	-0.044<br>0.161<br>0.058

Crystal	π-π stacking (Å)<br>290K	π-π stacking (Å)<br>190K	Δ (Å)
**imine-Br**	4.050	3.985	0.065
**diazo-Br**	3.947	3.902	0.045
**diazo-I Br**	4.022	3.974	0.048

Table S38. Intermolecular interaction distances that contribute to the TE along X_2.

Crystal	X···X (Å)<br>290/270/250 K[a]	X···X (Å)<br>190 K	Δ (Å)
**olefin-I**	type II I···I: 4.053	4.028	0.025
**olefin-Br**	type II Br···Br: 3.875	3.837	0.038
**olefin-I Br**	type II I···I: 4.010<br>type II Br···Br: 3.931<br>type II I···Br: 3.852; 4.093<br>centroid type II: 3.970	3.874<br>4.080<br>3.890; 4.062<br>3.975	0.136<br>-0.149<br>-0.038; 0.031 (avg. -0.004)<br>-0.005
**imine-I**	type II I···I: 4.082	4.027	0.055
**imine-Br**	type II Br···Br: 3.844	3.815	0.029
**azo-I**	type II I···I: 4.099	4.042	0.057
**azo-Br**(a)	type II Br···Br: 3.842	3.812	0.030
**azo-Br**(b)	type II Br···Br: 3.582	3.546	0.036
**azo-I Br**	type II I···I: 3.879<br>type II Br···Br: 3.986<br>type II I···Br: 3.836; 4.029<br>centroid type II: 3.932	3.844<br>3.967<br>3.774; 4.032<br>3.901	0.035<br>0.019<br>0.062; -0.003 (avg. 0.030)<br>0.031
**diolefin-I**	type II I···I: 4.050	4.016	0.034
**diolefin-Br**	type II Br···Br: 3.865	3.829	0.036
**diolefin-I Br**	type II I···I: 3.949<br>type II Br···Br: 4.006<br>type II I···Br: 4.081; 3.877<br>centroid type II: 3.977	3.883<br>4.023<br>4.092; 3.817<br>3.952	0.066<br>-0.017<br>-0.011; 0.060 (avg. 0.025)<br>0.025
**diimine-I**	type II I···I: 4.135; 3.951	4.105; 3.919	0.030; 0.032 (avg. 0.031)
**diimine-Br**	type II Br···Br: 3.721; 3.950	3.686; 3.912	0.035; 0.038 (avg. 0.037)
**diazo-I**	type II I···I: 3.984; 4.146	3.933; 4.162	0.051; -0.016 (avg. 0.018)
**diazo-Br**	type II Br···Br: 3.853	3.828	0.025
**diazo-I Br**	type II I···I: 3.847<br>type II Br···Br: 4.046<br>type II I···Br: 3.915; 3.969<br>centroid type II: 3.942	3.924<br>3.859<br>3.854; 3.916<br>3.885	-0.077<br>0.187<br>0.061; 0.053 (avg. 0.057)<br>0.057

[a]All the bond length was calculated using the X-ray data at 290 K, except **olefin-I**, which is at 270 K, and **diazo-I**, which is at 250 K.

Crystal	π···π (Å)<br>290 K	π ···π (Å)<br>190 K	Δ (Å)
**azo-Br**(b)	4.769	4.750	0.019

Table S39. Intermolecular interaction distances that contribute to the TE along X_3.

Crystal	C-H···π (Å) 290/270/250 K[a]	C-H···π (Å) 190 K	Δ (Å)	avg. Δ (Å)
**olefin-I**	3.623; 3.573	3.561; 3.547	0.062; 0.026	0.044
**olefin-Br**	3.585; 3.540	3.538; 3.502	0.047; 0.038	0.043
**olefin-I Br**	3.576; 3.610	3.533; 3.562	0.043; 0.048	0.046
**imine-I**	3.611; 3.623	3.564; 3.564	0.047; 0.059	0.053
**azo-I**	3.594; 3.610	3.534; 3.576	0.060; 0.034	0.047
**diolefin-I**	3.622; 3.599; 3.577	3.567; 3.548; 3.539	0.055; 0.051; 0.038	0.048
**diolefin-Br**	3.589; 3.566; 3.551	3.543; 3.520; 3.506	0.046; 0.046; 0.045	0.046
**diolefin-I Br**	3.575; 3.587; 3.613	3.525; 3.538; 3.561	0.050; 0.049; 0.052	0.050
**diimine-I**	3.643; 3.541; 3.575<br>3.540; 3.670; 3.624	3.594; 3.489; 3.524<br>3.484; 3.624; 3.576	0.049; 0.052; 0.051<br>0.056; 0.046; 0.048	0.050
**diimine-Br**	3.547; 3.668; 3.607<br>3.557; 3.532; 3.643	3.497; 3.617; 3.551<br>3.511; 3.485; 3.581	0.050; 0.051; 0.056<br>0.046; 0.047; 0.062	0.052
**diazo-I**	3.472; 3.563; 3.636	3.434; 3.529; 3.608	0.038; 0.034; 0.028	0.033

[a]All the bond length was calculated using the X-ray data at 290 K, except **olefin-I**, which is at 270 K, and **diazo-I**, which is at 250 K.

Crystal	π-π stacking (Å) 290 K	π-π stacking (Å) 190 K	Δ (Å)
**imine-Br**	4.050	3.985	0.065
**azo-I Br**	4.089	4.035	0.054
**azo-Br**(a)	3.992	3.944	0.048
**diazo-Br**	3.947	3.902	0.045
**diazo-I Br**	4.022	3.974	0.048

Crystal	X···X (Å) 290 K	X···X (Å) 190 K	Δ (Å)
**azo-Br**(b)	type II Br···Br: 3.582	3.546	0.036

Crystal	C-H···X (Å) 290/270/250 K[a]	C-H···X (Å) 190K	Δ (Å)
**olefin-I**	C-H···I: 3.991	3.965	0.026
**olefin-Br**	C-H···Br: 3.975	3.940	0.035
**olefin-I Br**	C-H···I: 3.993<br>C-H···Br: 3.973<br>C-H···centroid I/Br: 3.982	3.986<br>3.895<br>3.939	0.007<br>0.078<br>0.043
**imine-I**	C-H···I: 4.029	3.983	0.046
**azo-I**	C-H···I: 3.998	3.973	0.025
**diolefin-I**	C-H···I: 4.020	3.975	0.045
**diolefin-Br**	C-H···Br: 3.989	3.952	0.037
**diolefin-I Br**	C-H···I: 4.022	3.990	0.032

	C-H···Br: 3.953<br>C-H···centroid I/Br: 3.987	3.910<br>3.949	0.043<br>0.038
**diimine-I**	C-H···I: 4.087; 4.042	4.045; 4.000	0.042; 0.042 (avg. 0.042)
**diimine-Br**	C-H···Br: 4.006; 3.993	3.976; 3.955	0.030; 0.038 (avg. 0.034)
**diazo-I**	C-H···I: 4.020	4.002	0.018

[a]All the bond length was calculated using the X-ray data at 290 K, except **olefin-I**, which is at 270 K, and **diazo-I**, which is at 250 K.

4. Expansivity Indicatrix Diagrams

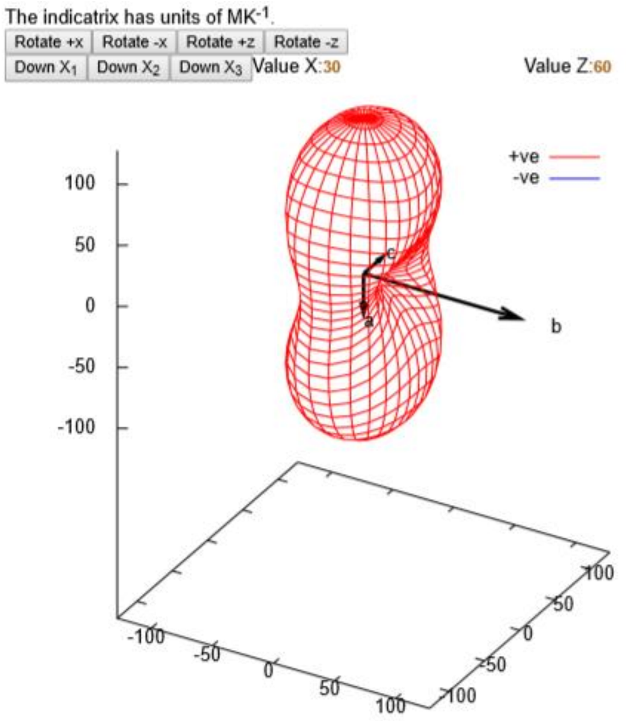

Figure S1. Thermal expansivity indicatrix for **olefin-I**.

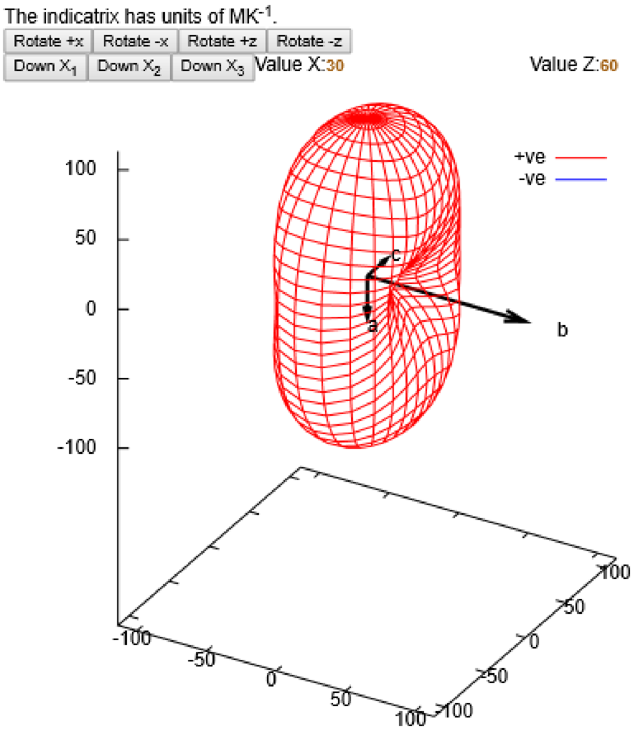

Figure S2. Thermal expansivity indicatrix for **olefin-Br**.

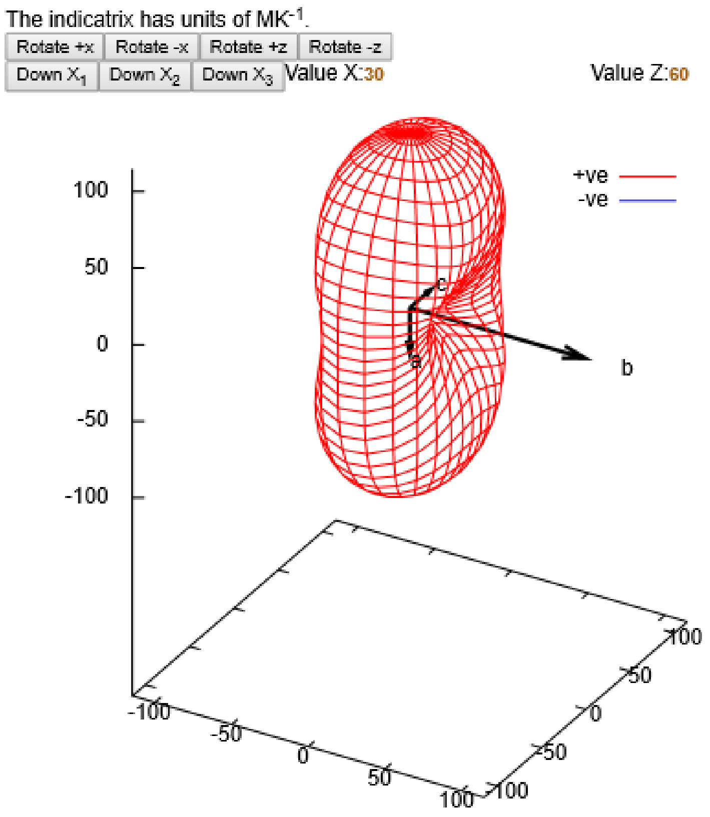

Figure S3. Thermal expansivity indicatrix for **olefin-I Br**.

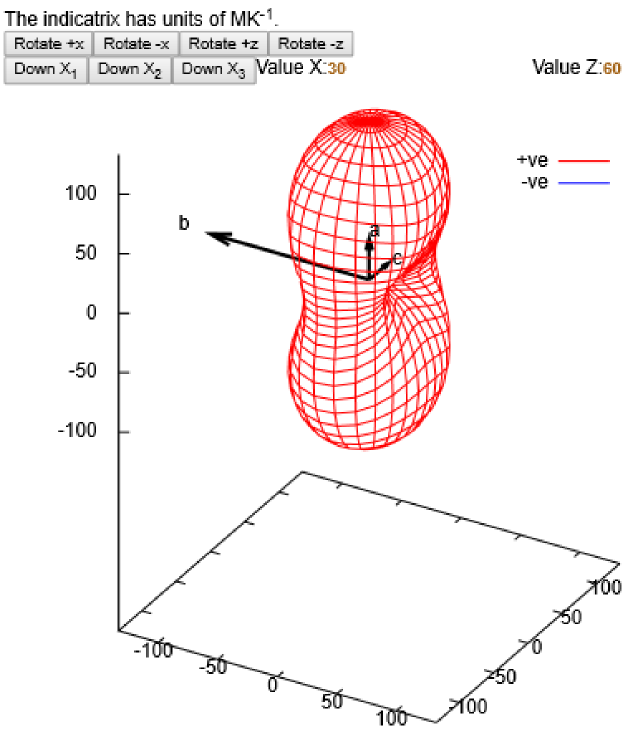

Figure S4. Thermal expansivity indicatrix for **imine-I**.

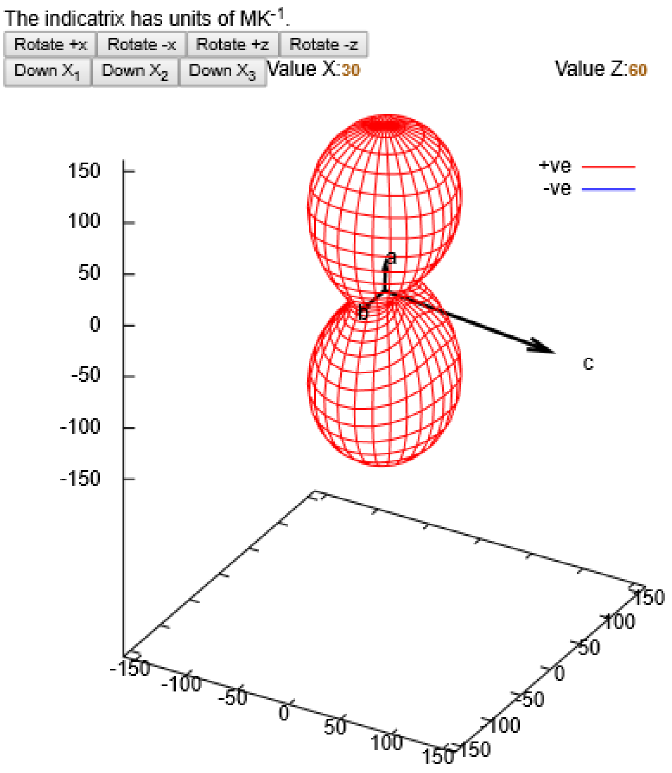

Figure S5. Thermal expansivity indicatrix for **imine-Br**.

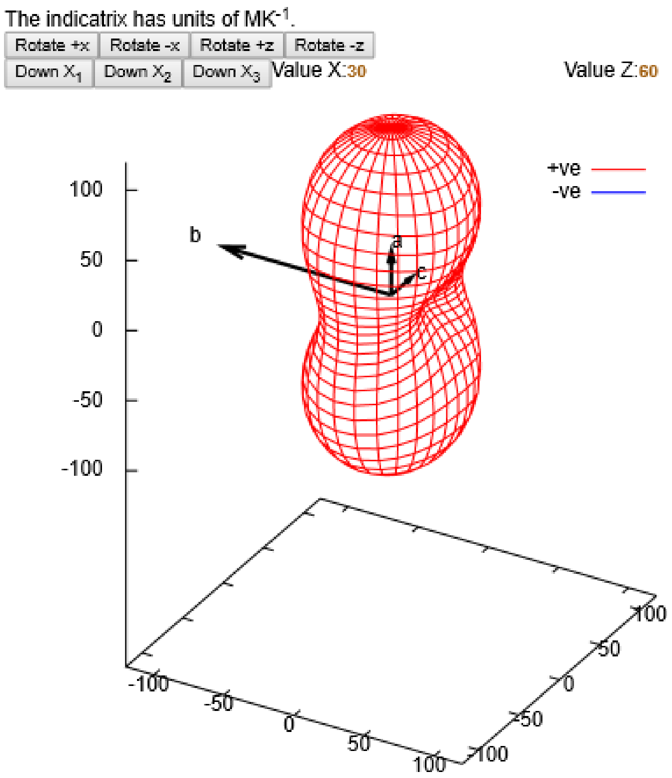

Figure S6. Thermal expansivity indicatrix for **azo-I**.

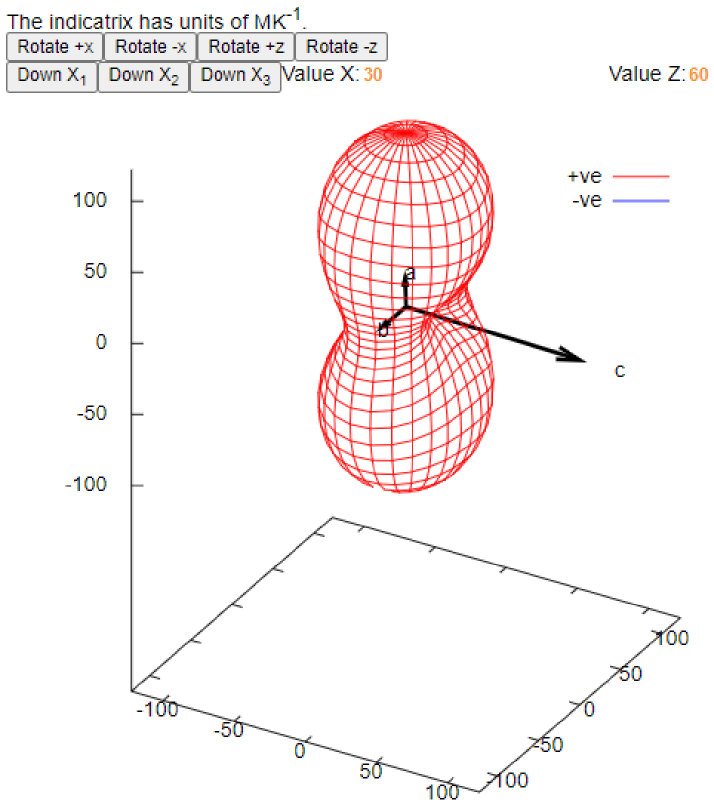

Figure S7. Thermal expansivity indicatrix for **azo-Br**(a).

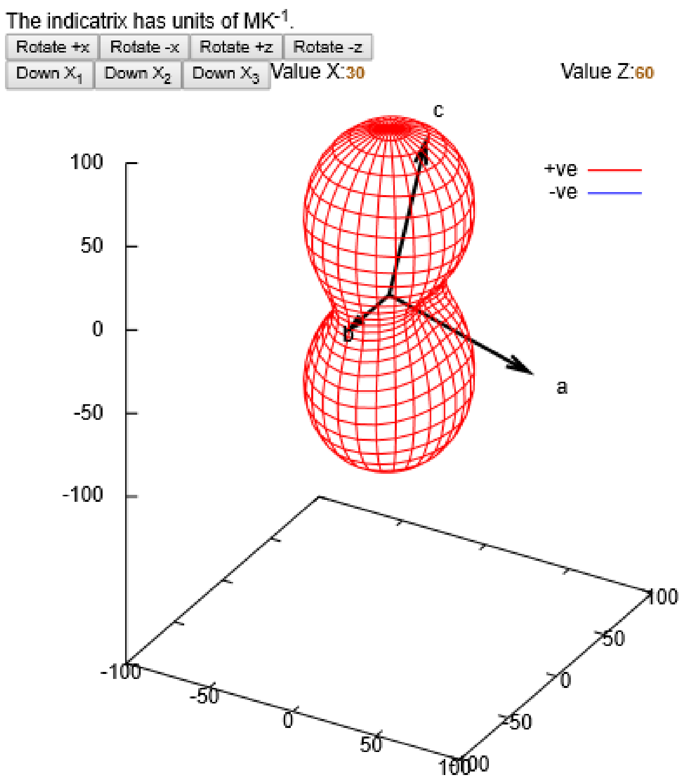

Figure S8. Thermal expansivity indicatrix for **azo-Br**(b).

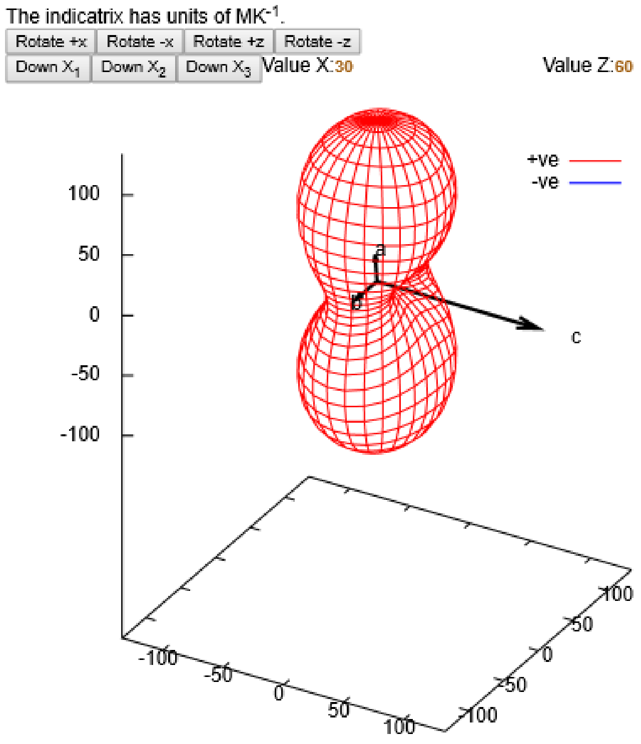

Figure S9. Thermal expansivity indicatrix for **azo-I Br**.

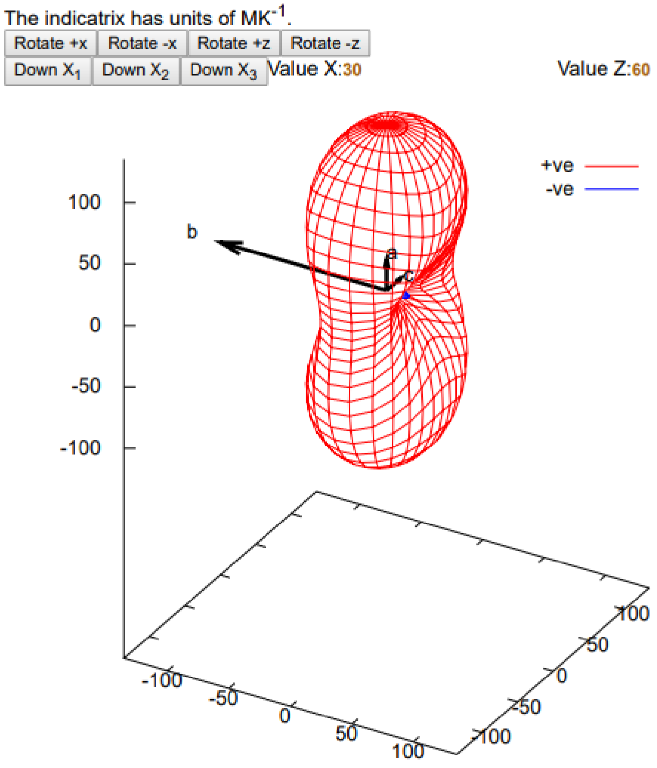

Figure S10. Thermal expansivity indicatrix for **diolefin-I**.

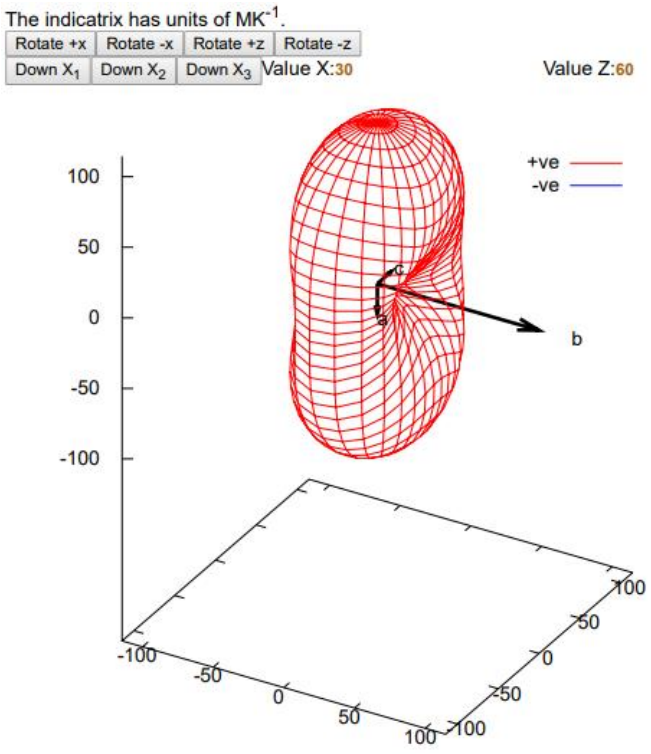

Figure S11. Thermal expansivity indicatrix for **diolefin-Br**.

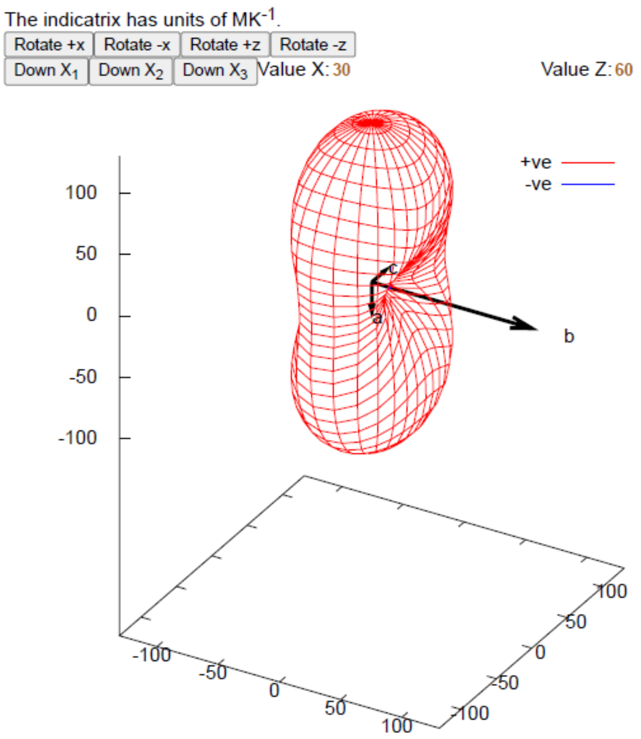

Figure S12. Thermal expansivity indicatrix for **diolefin-I Br**.

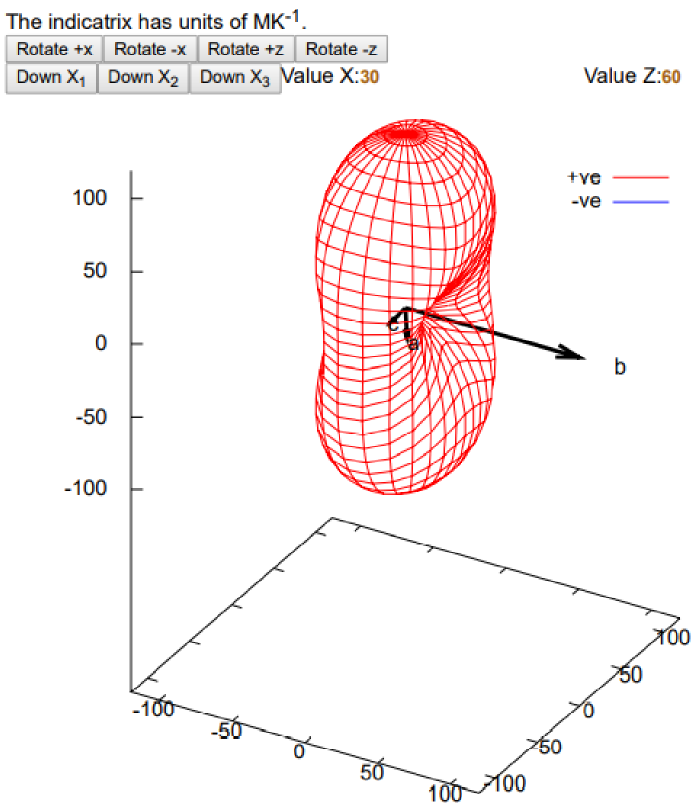

Figure S13. Thermal expansivity indicatrix for **diimine-I**.

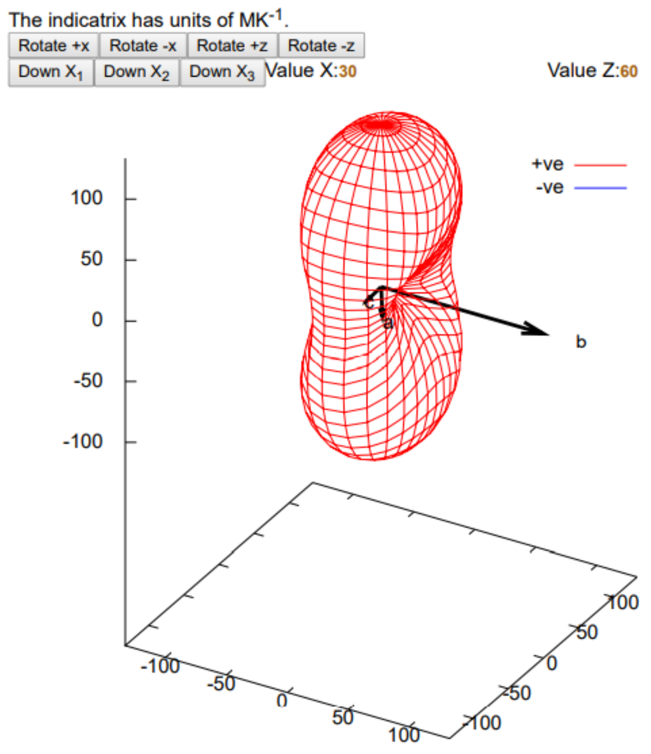

Figure S14. Thermal expansivity indicatrix for **diimine-Br**.

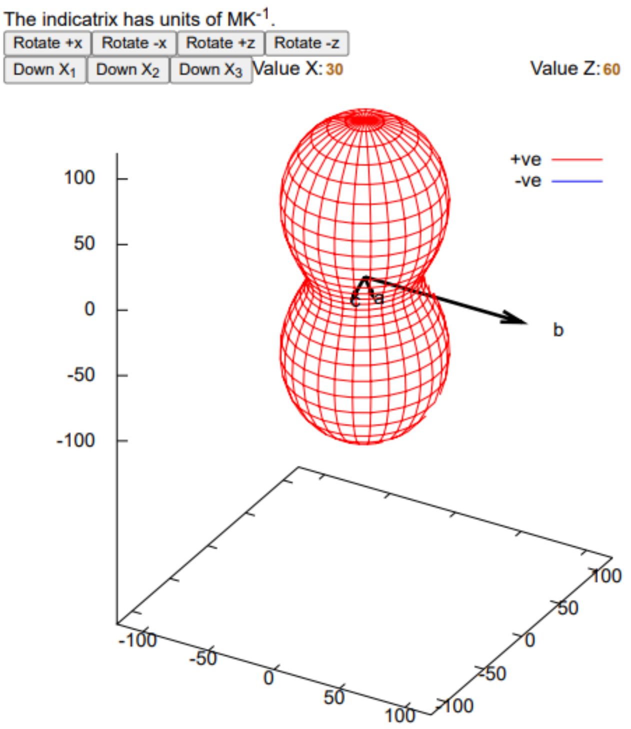

Figure S15. Thermal expansivity indicatrix for **diazo-I**.

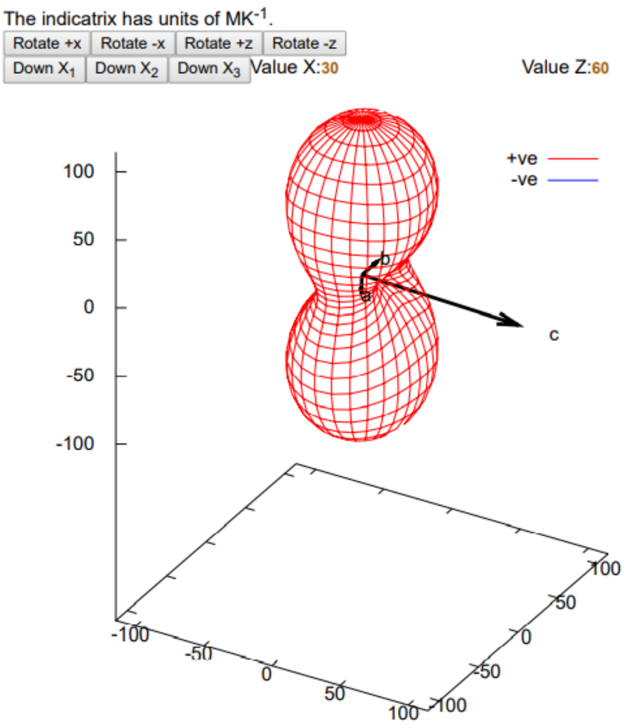

Figure S16. Thermal expansivity indicatrix for **diazo-Br**.

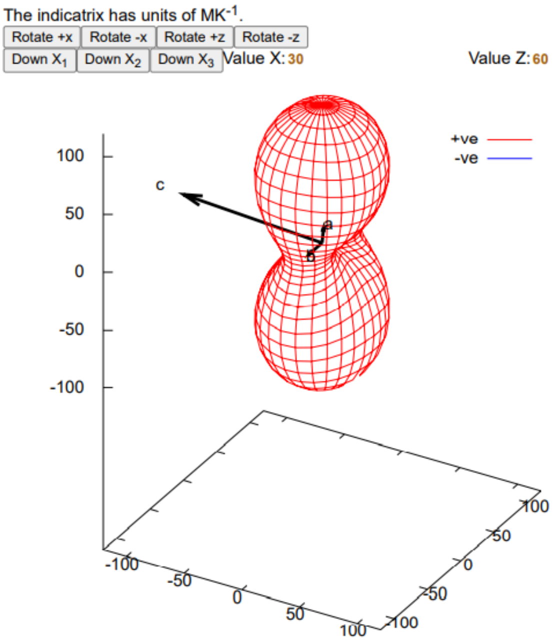

Figure S17. Thermal expansivity indicatrix for **diazo-I Br**.

5. NMR Spectra of the Compounds

All the compounds were dissolved in $CDCl_3$ for NMR experiments. NMR data was collected using a JOEL ECS 400 MHZ Spectrometer.

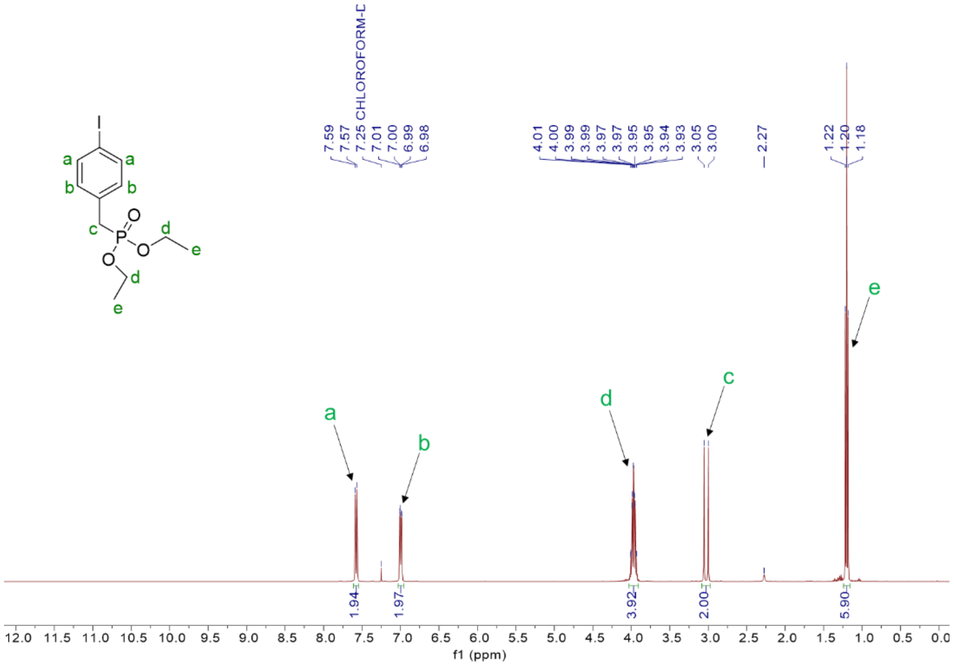

Figure S18. ^{1}H NMR spectrum for compound **1**.

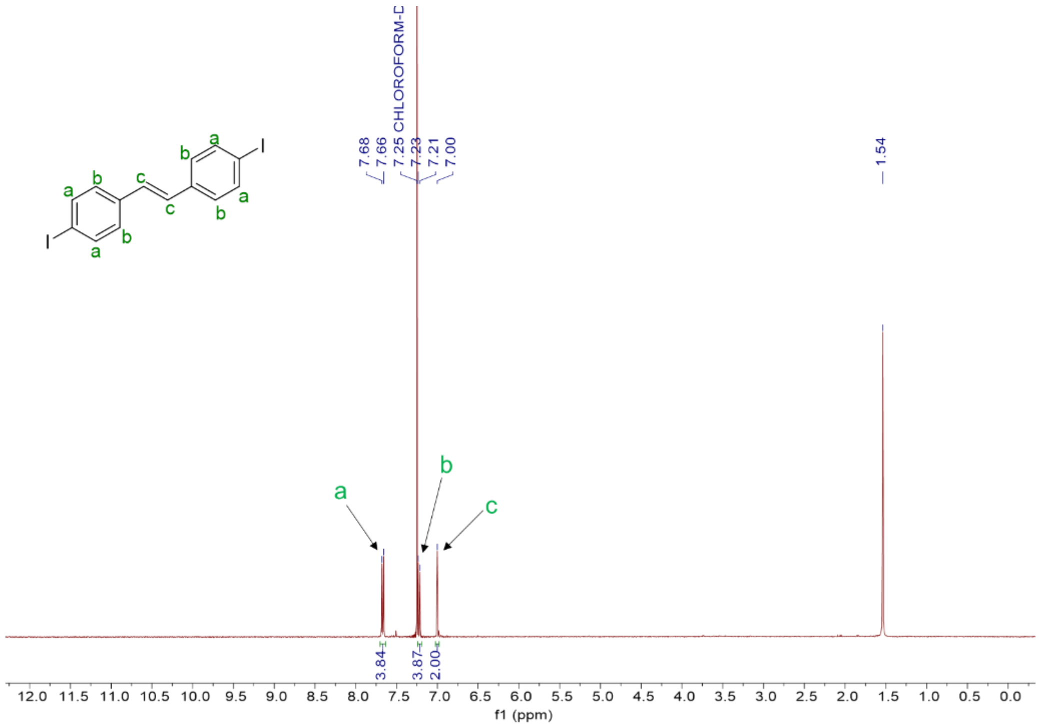

Figure S19. ^{1}H NMR spectrum for **olefin-I**.

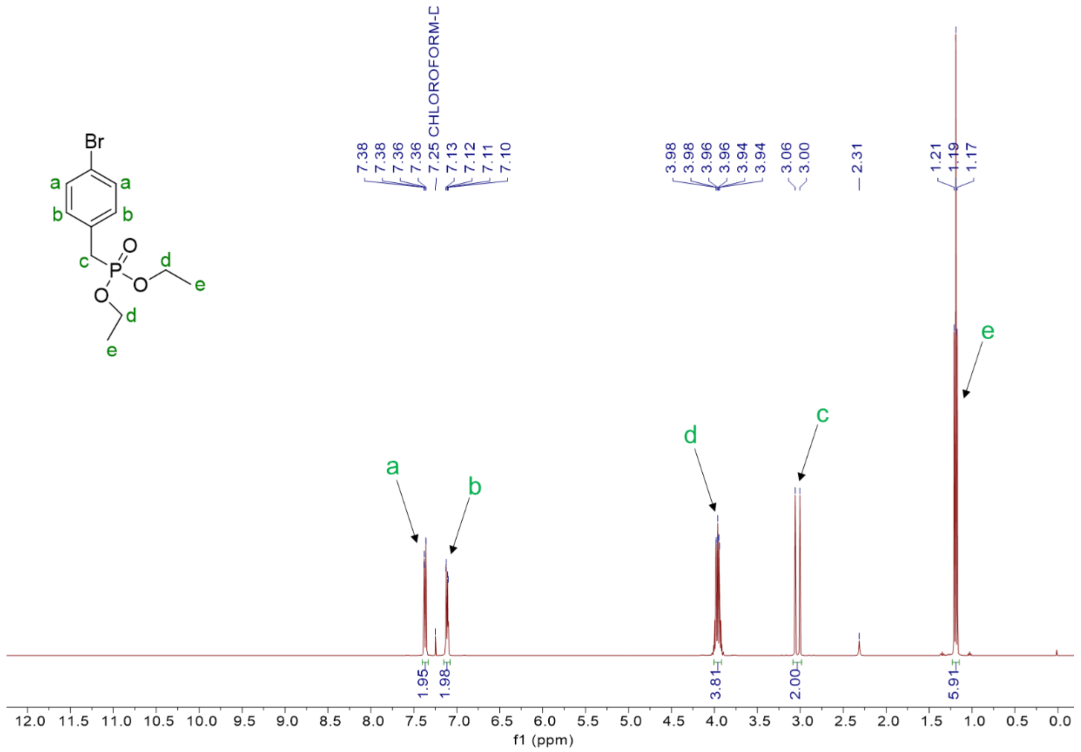

Figure S20. ^{1}H NMR spectrum for compound **2**.

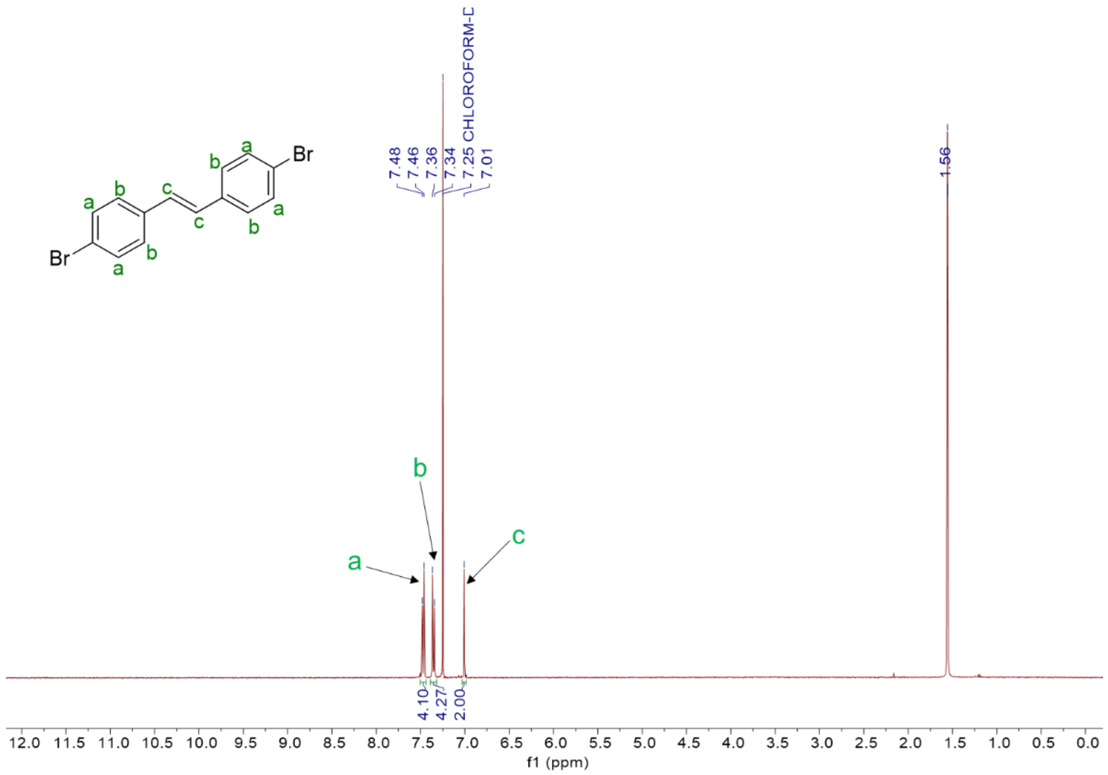

Figure S21. ^{1}H NMR spectrum for **olefin-Br**.

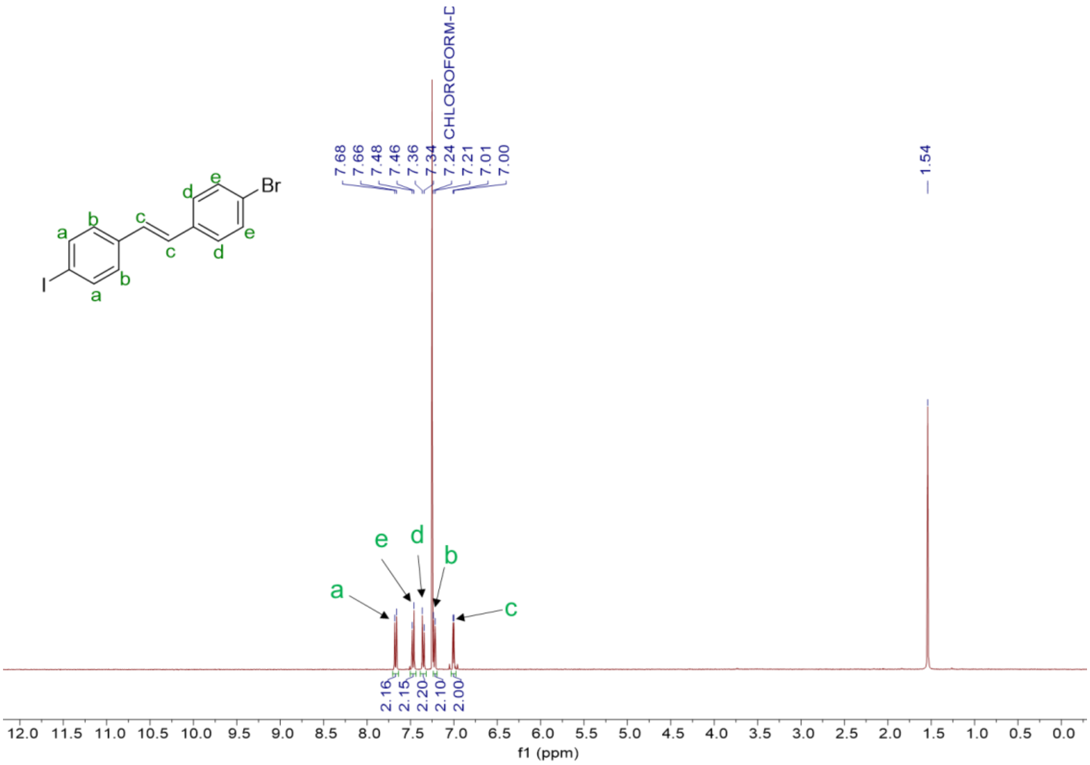

Figure S22. ^{1}H NMR spectrum for **olefin-I Br**.

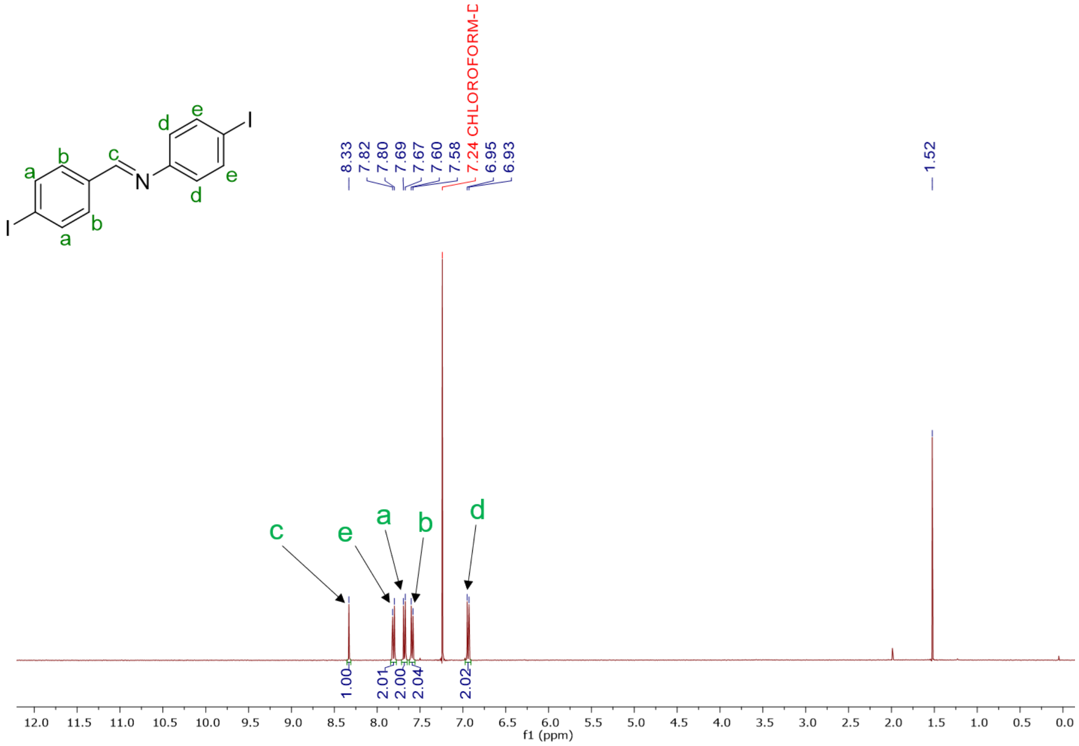

Figure S23. ^{1}H NMR spectrum for **imine-I**.

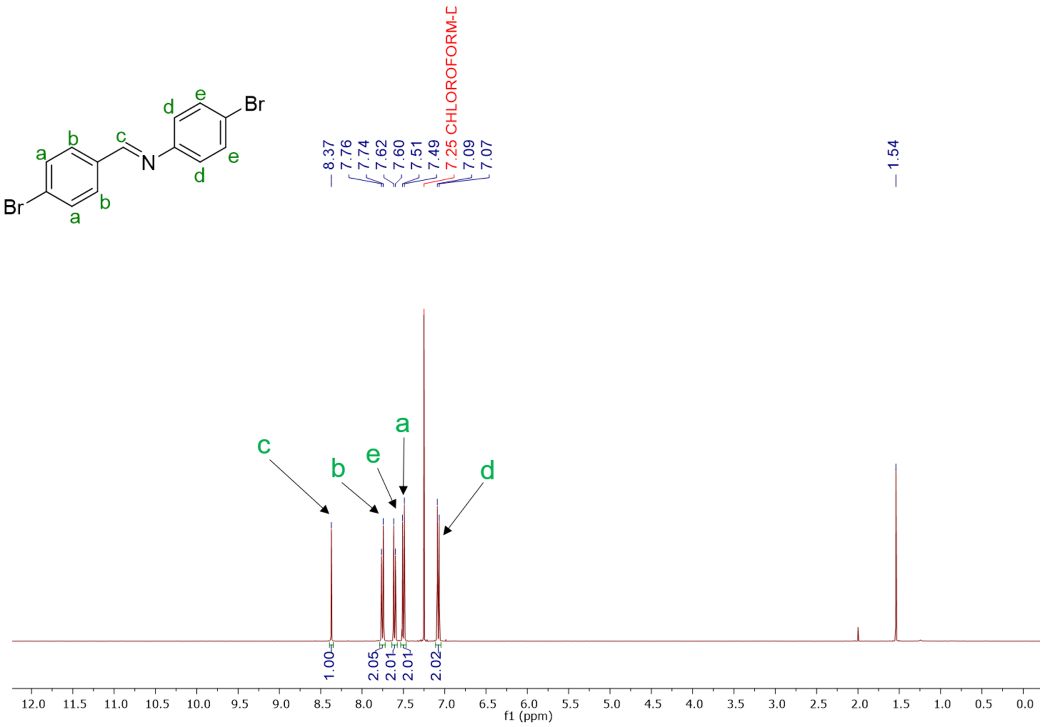

Figure S24. ^{1}H NMR spectrum for **imine-Br**.

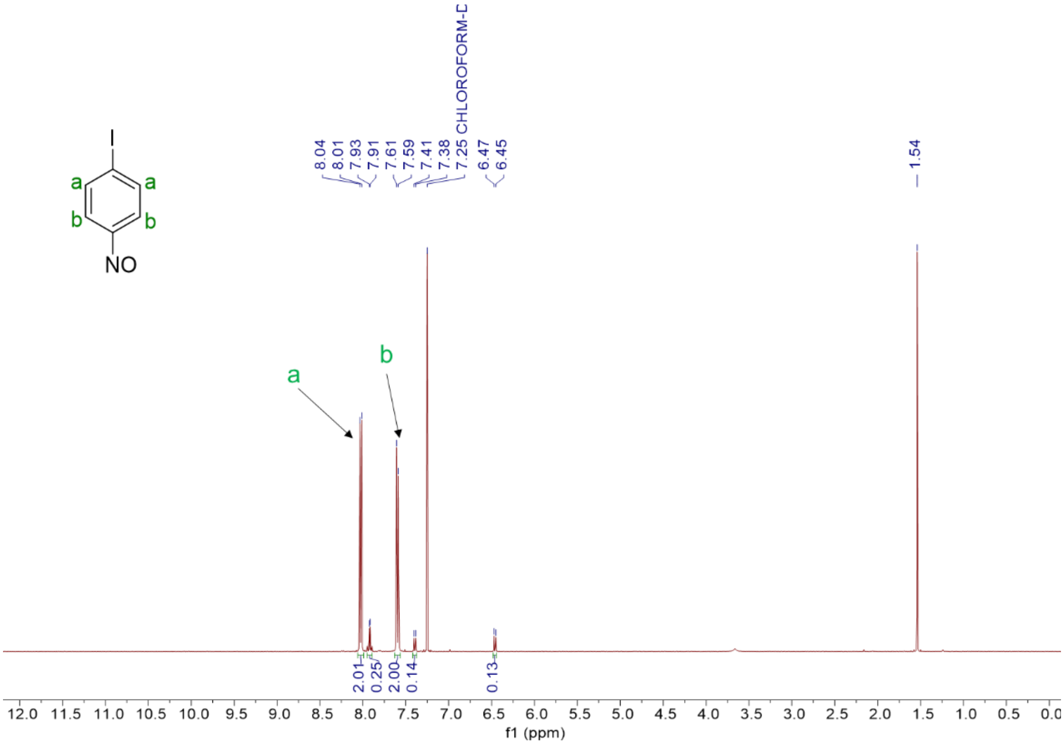

Figure S25. ^{1}H NMR spectrum for compound **3**.

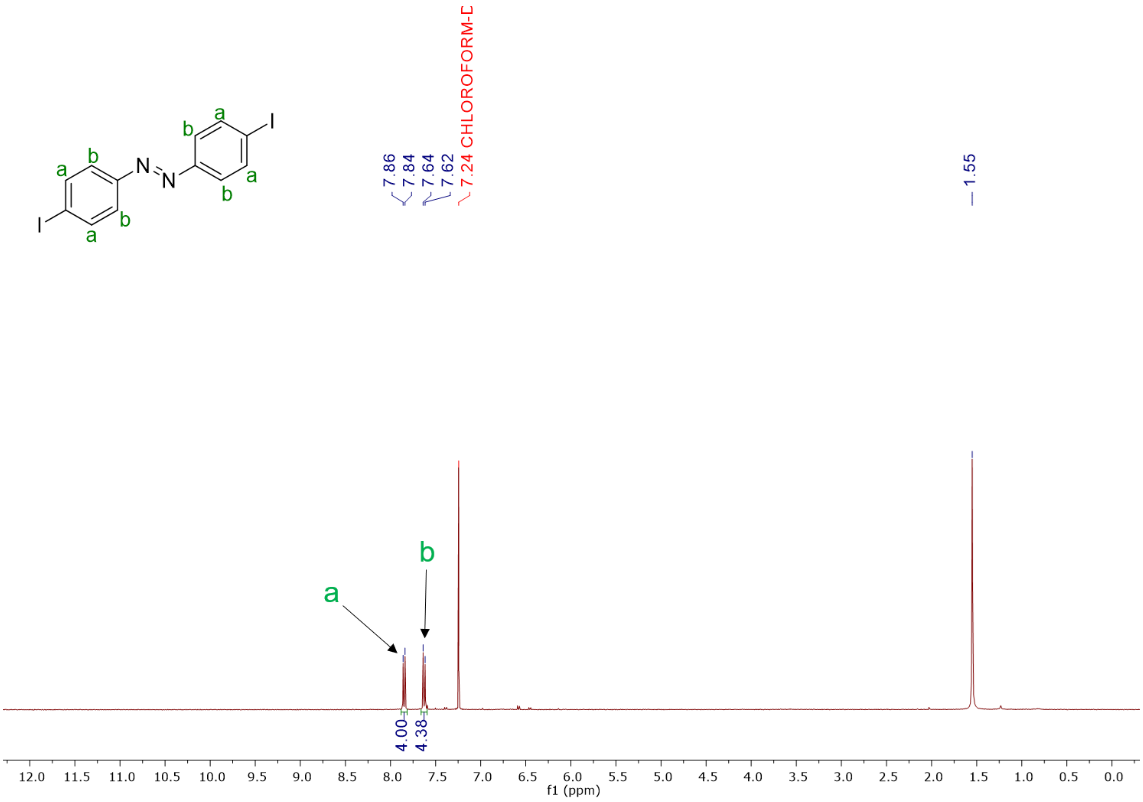

Figure S26. ^{1}H NMR spectrum for **azo-I**.

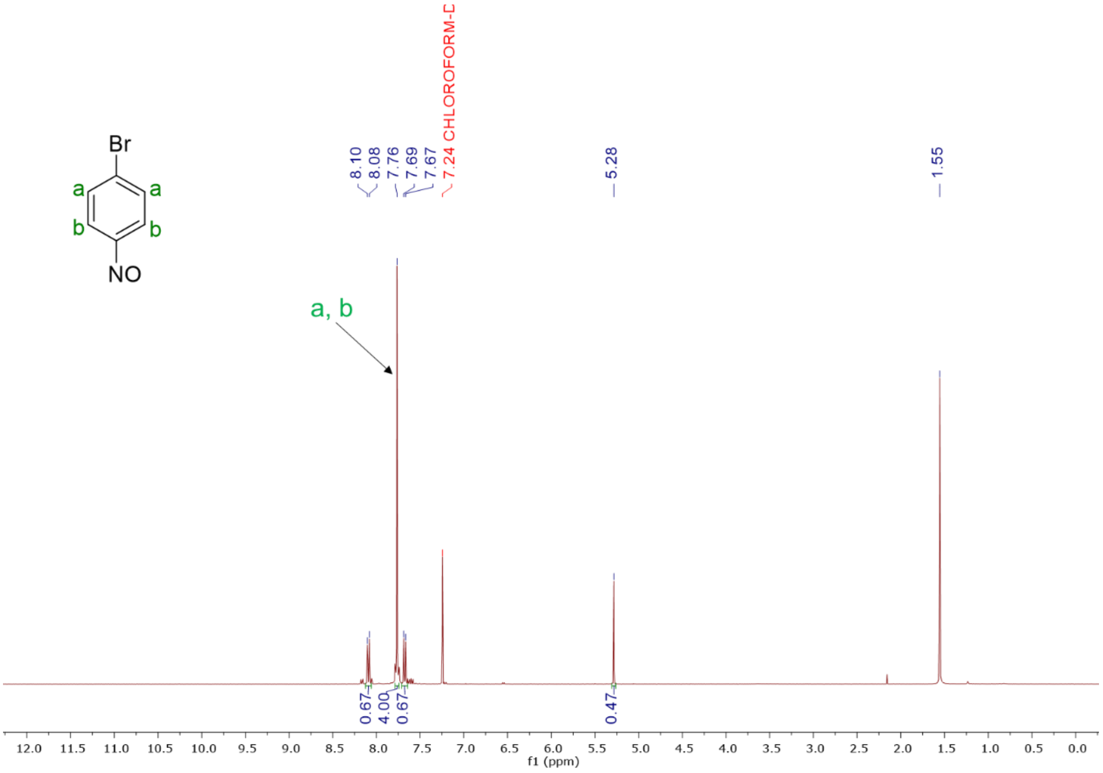

Figure S27. ^{1}H NMR spectrum for compound **4** (crude).

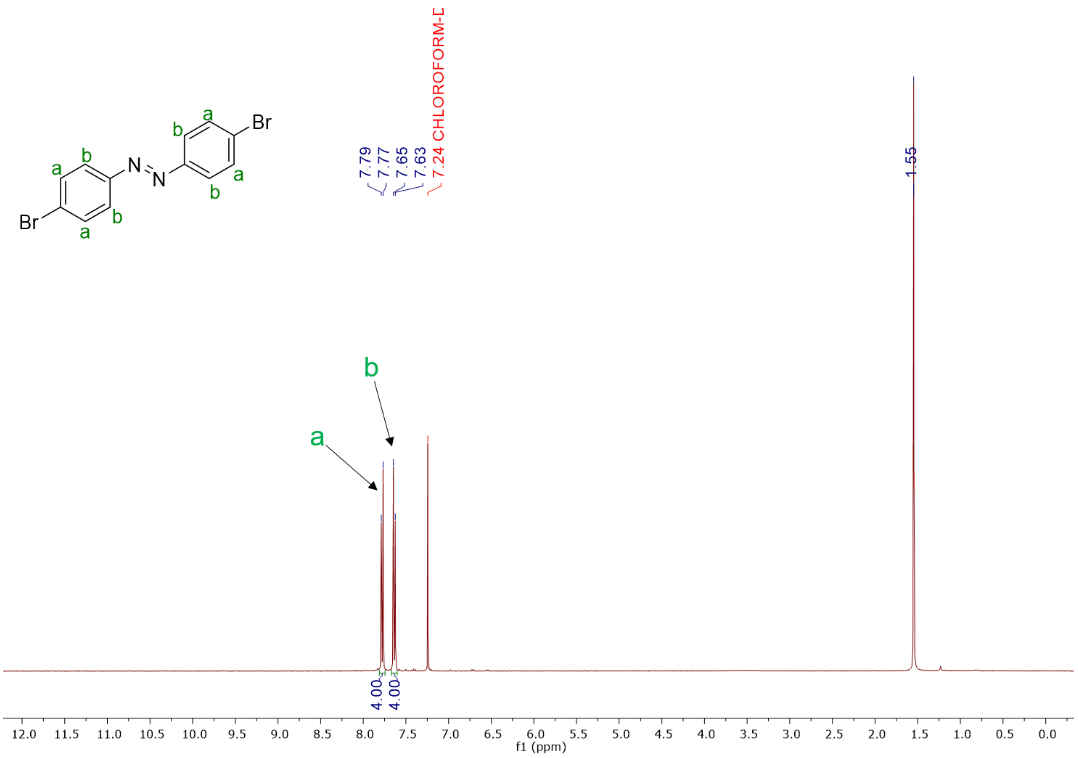

Figure S28. ^{1}H NMR spectrum for **azo-Br**.

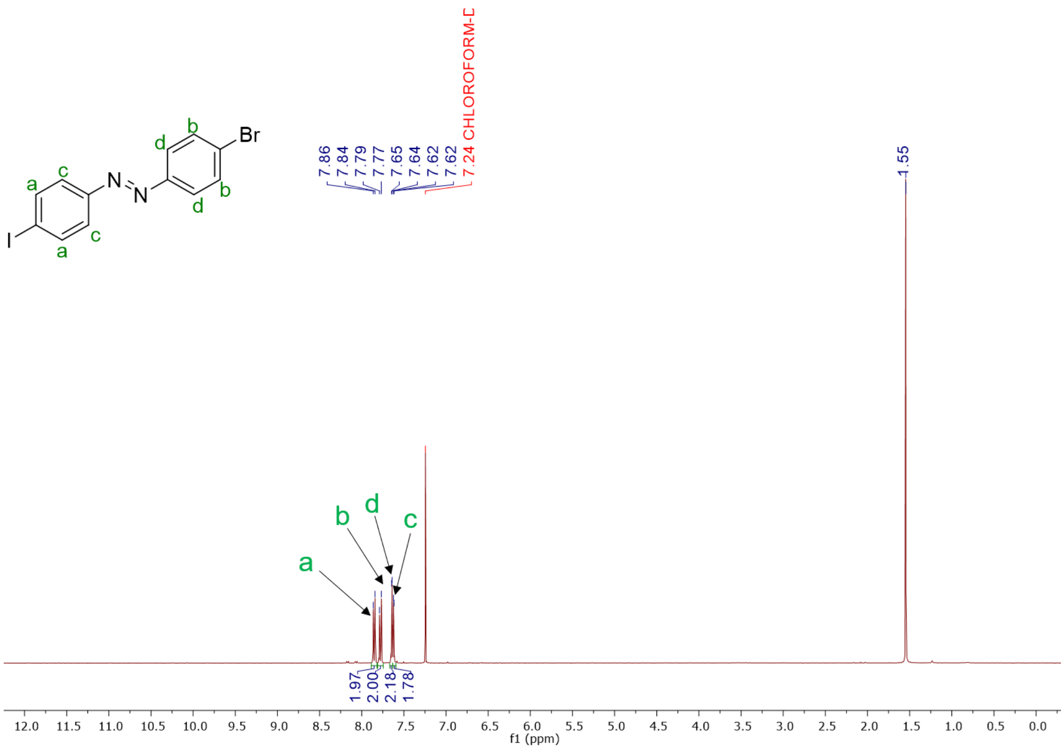

Figure S29. ^{1}H NMR spectrum for **azo-I Br**.

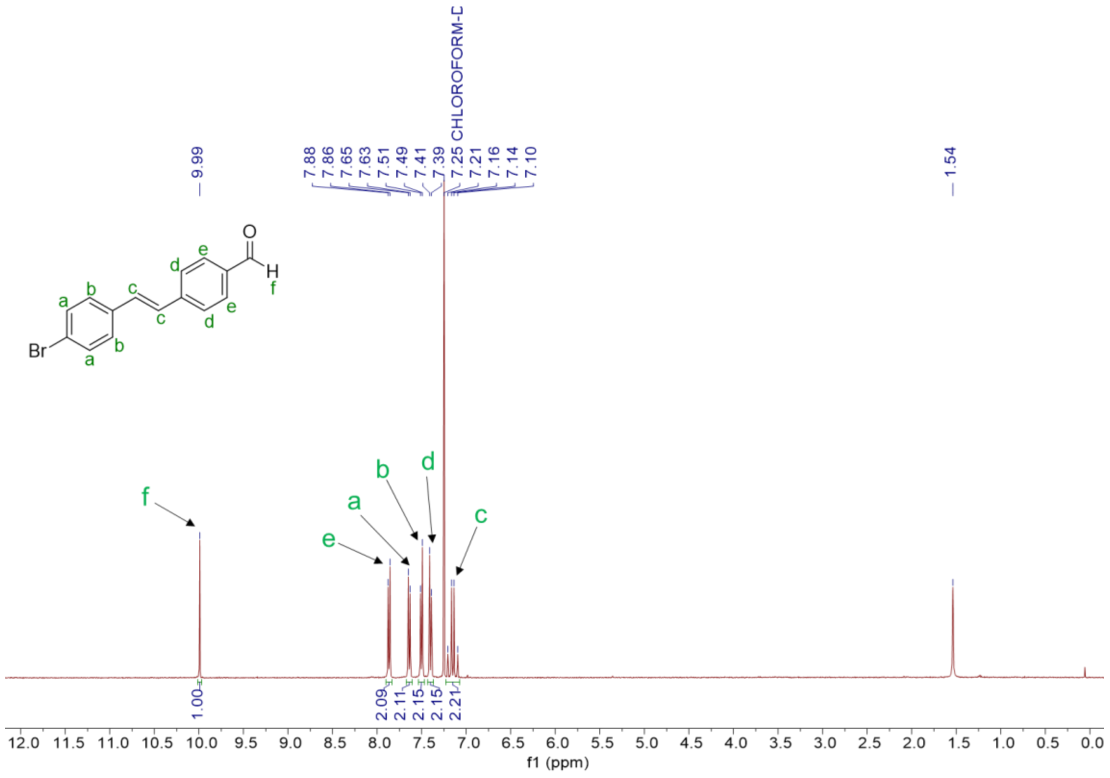

Figure S30. ^{1}H NMR spectrum for compound **5**.

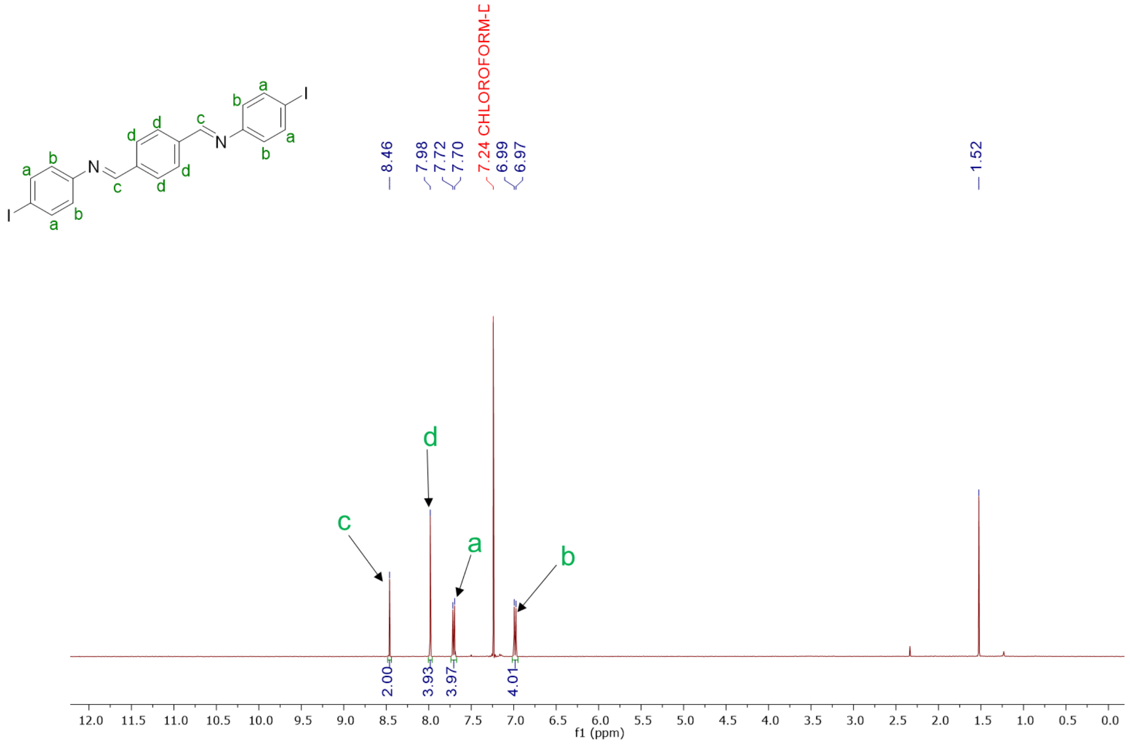

Figure S31. ^{1}H NMR spectrum for **diimine-I**.

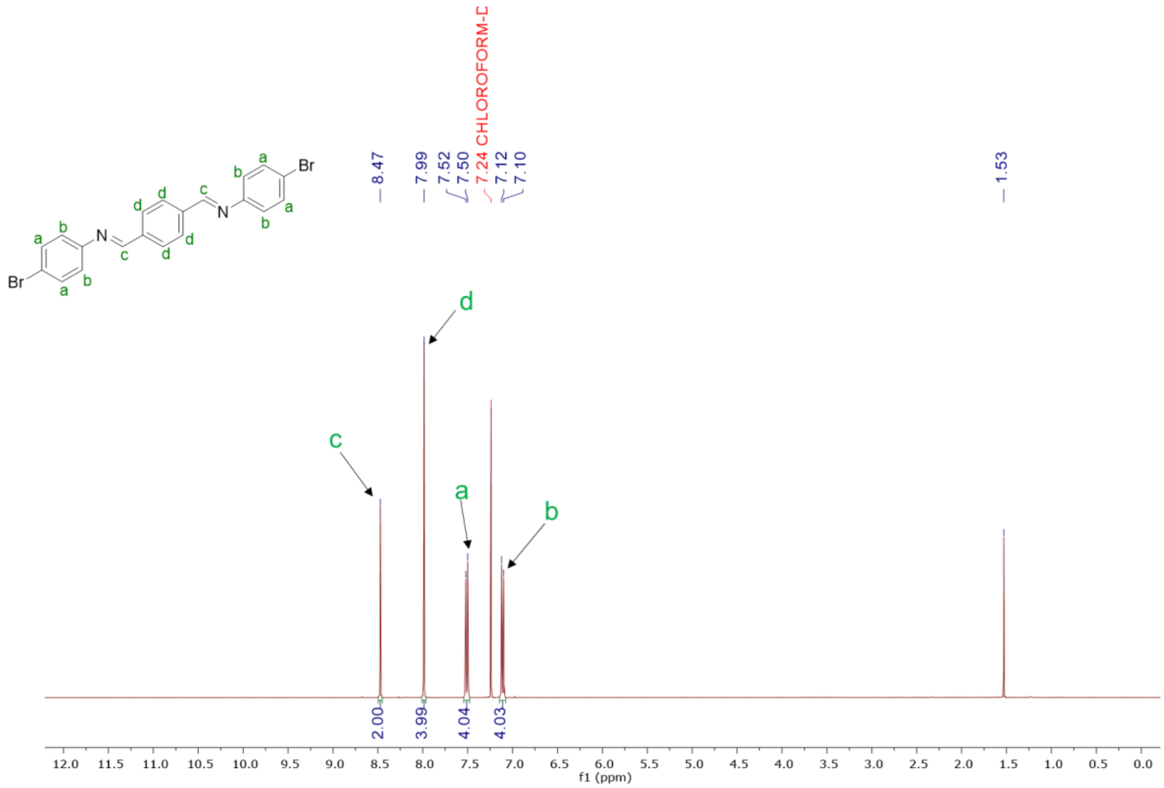

Figure S32. ^{1}H NMR spectrum for **diimine-Br**.

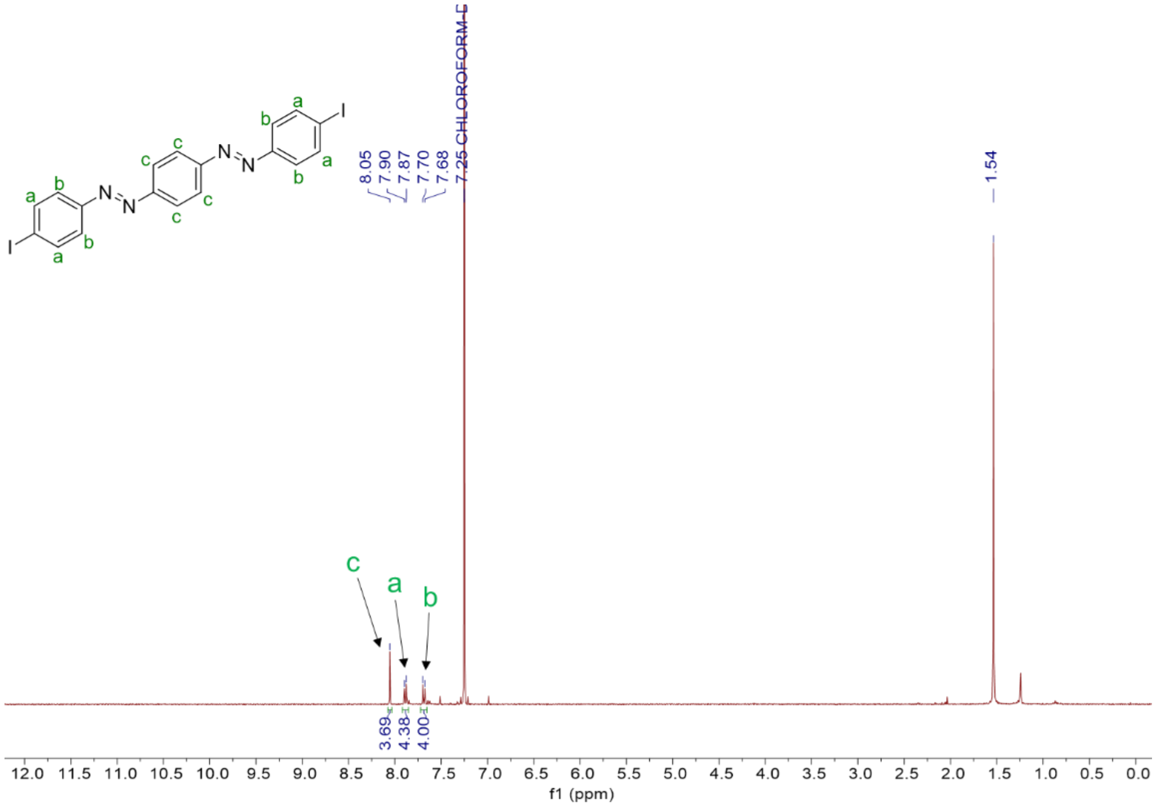

Figure S33. ^{1}H NMR spectrum for **diazo-I**.

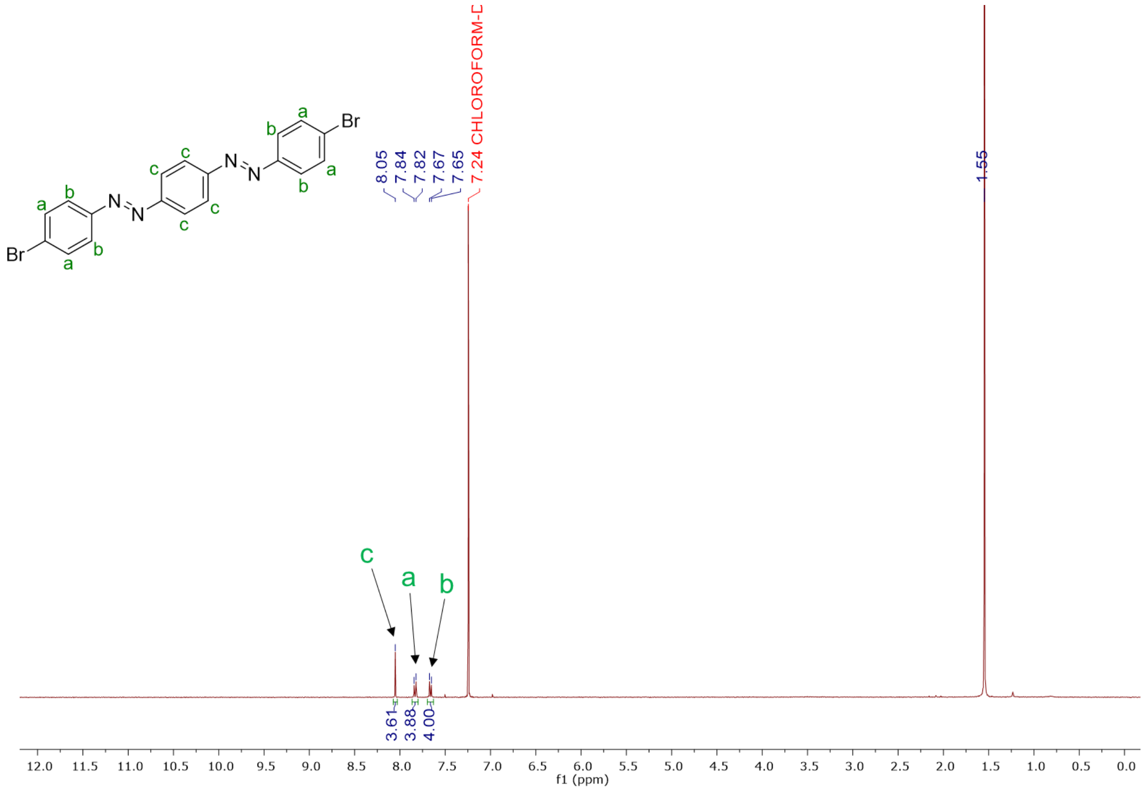

Figure S34. ^{1}H NMR spectrum for **diazo-Br**.

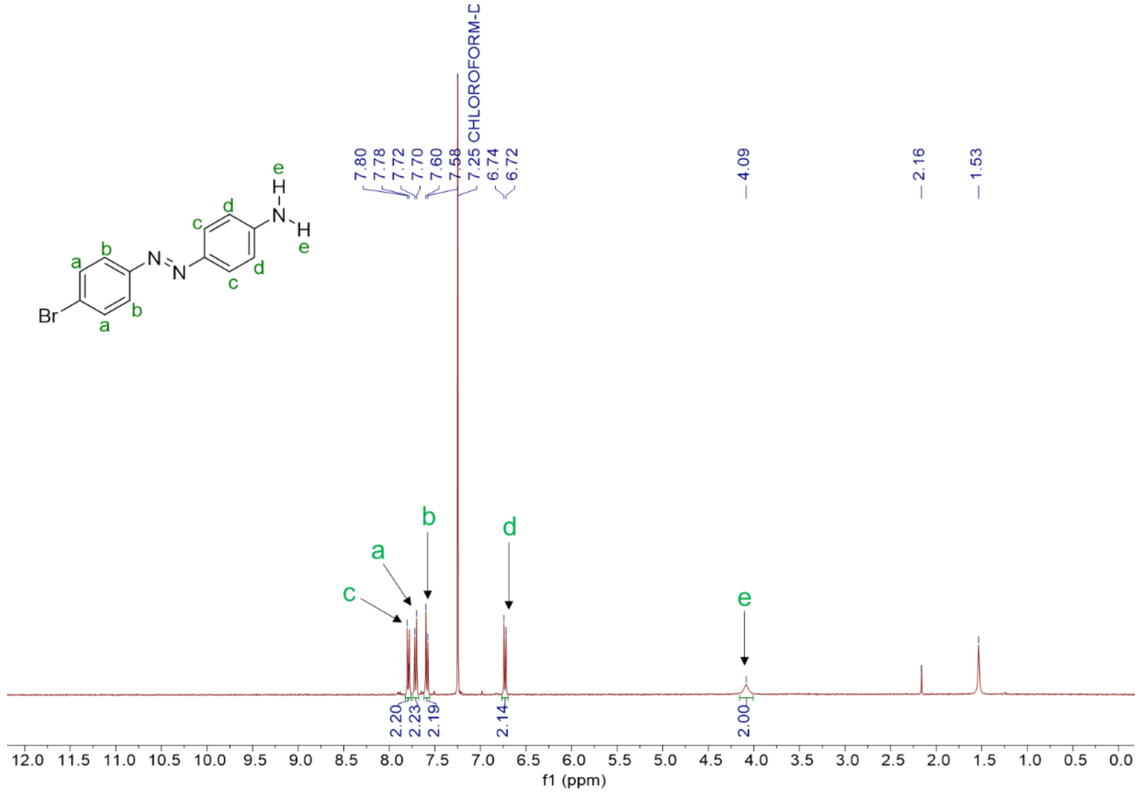

Figure S35. ^{1}H NMR spectrum for compound **6**.

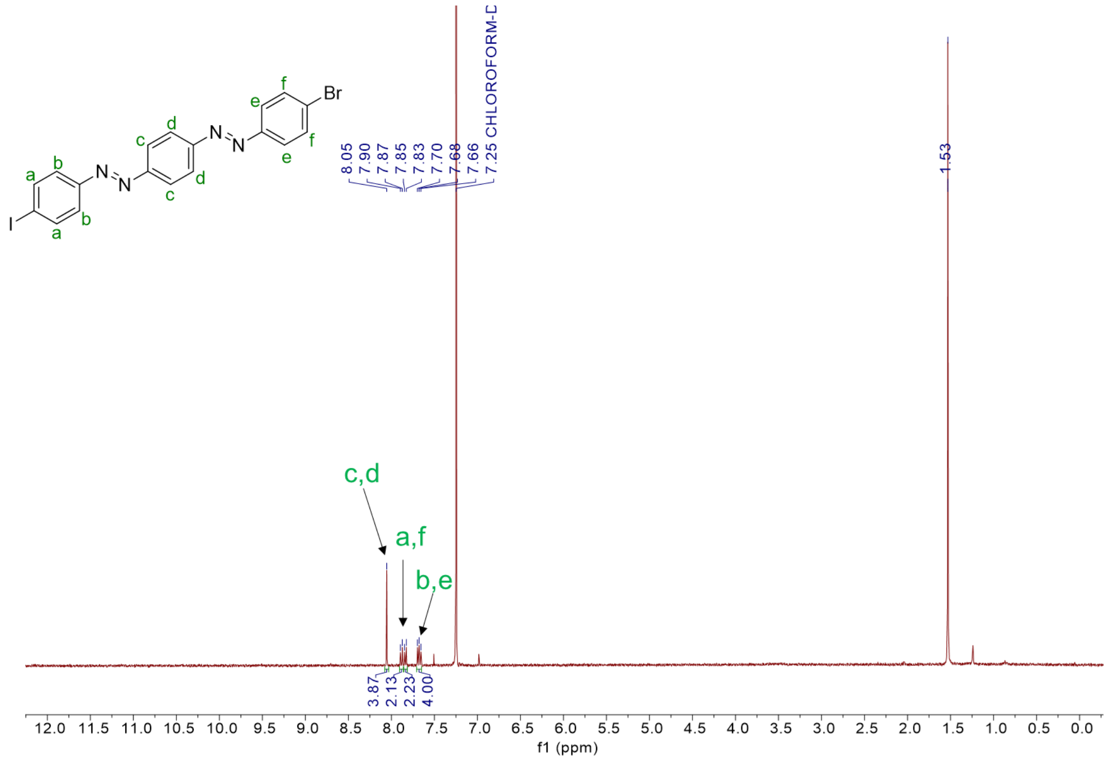

Figure S36. ^{1}H NMR spectrum for **diazo-I Br**.

6. PXRD Patterns

The diffraction patterns for all samples were collected on a Rigaku MiniFlex II powder diffractometer. An X-ray diffraction pattern was obtained by scanning a 2θ range of 3-60°, step size = 0.02°, and scan time of 2 degrees/minute. The X-ray source was Cu Kα radiation (λ =1.5418 Å) with an anode voltage of 30 kV and a current of 15 mA. Diffraction intensities were recorded on a position sensitive detector (D/teX Ultra). The sample was prepared as a standard powder mount, and the diffractogram was processed through the software MDI JADE 2020.

The diolefin molecules **diolefin-I**, **diolefin-Br**, and **diolefin-I Br** were not soluble in deuterated DMSO, chloroform, dichloromethane, and toluene, so PXRD patterns were collected for characterization. The simulated patterns from single-crystal X-ray diffraction data are provided for comparison.

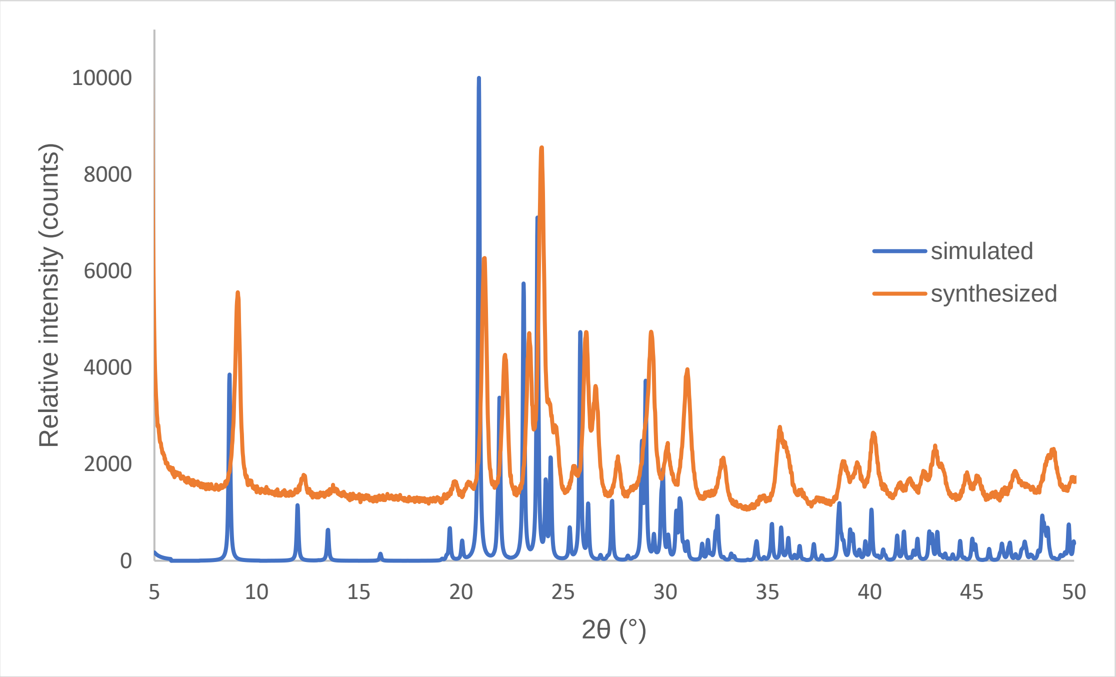

Figure S37. PXRD patterns for **diolefin-I**.

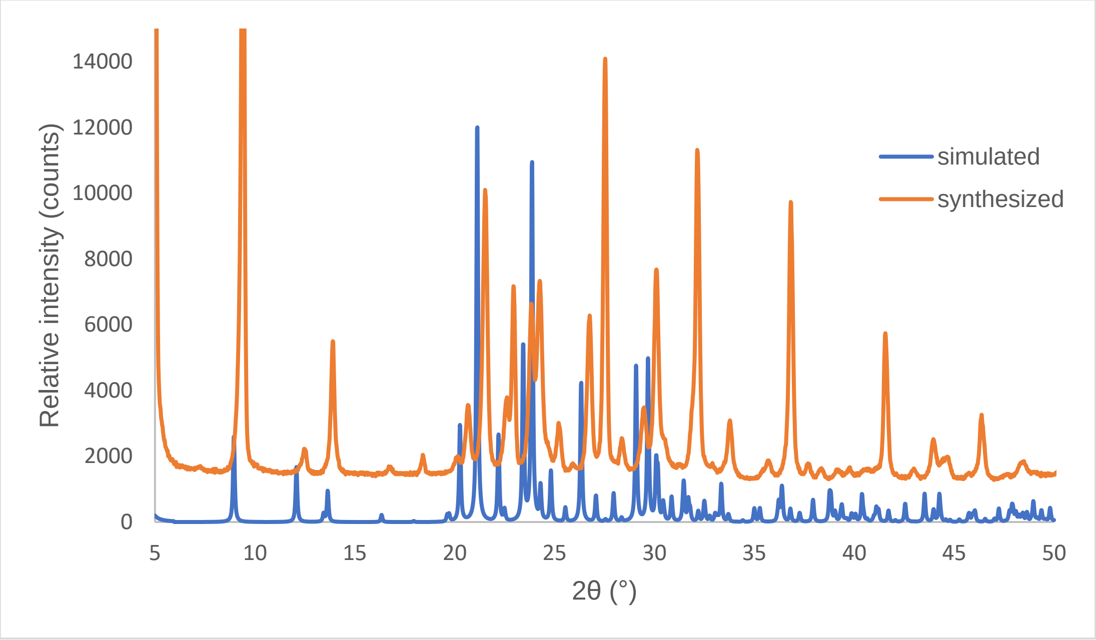

Figure S38. PXRD patterns for **diolefin-Br**.

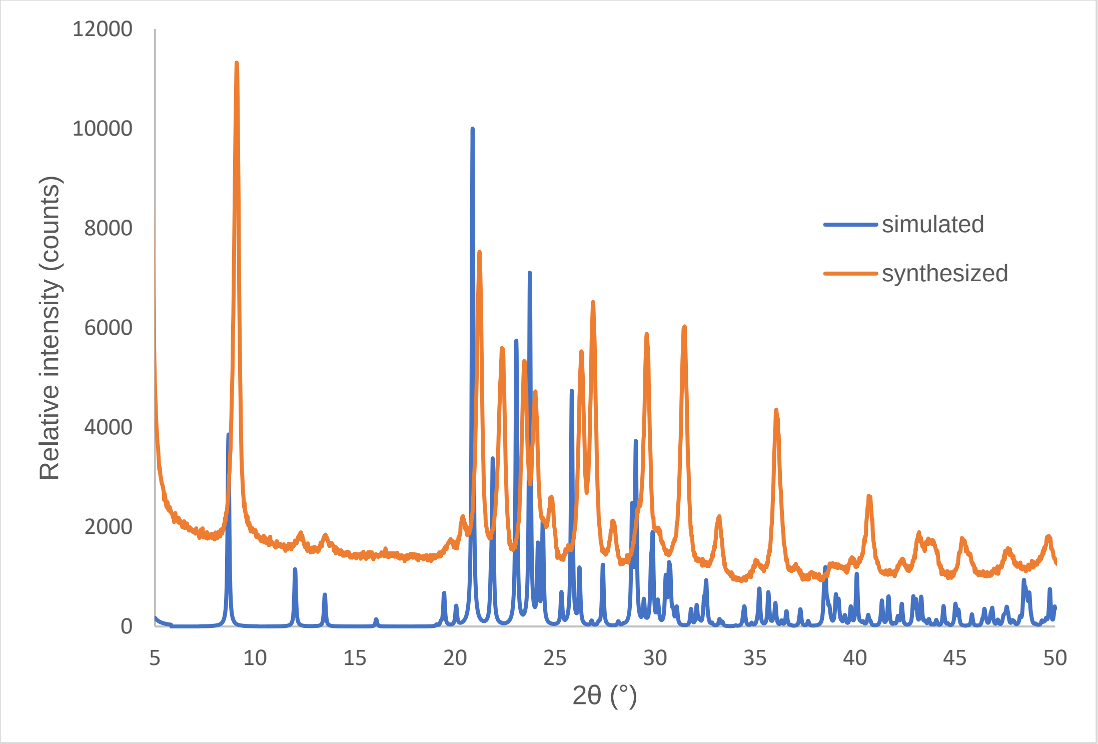

Figure S39. PXRD patterns for **diolefin-I Br**.

7. Variation of the Unit Cell Parameters

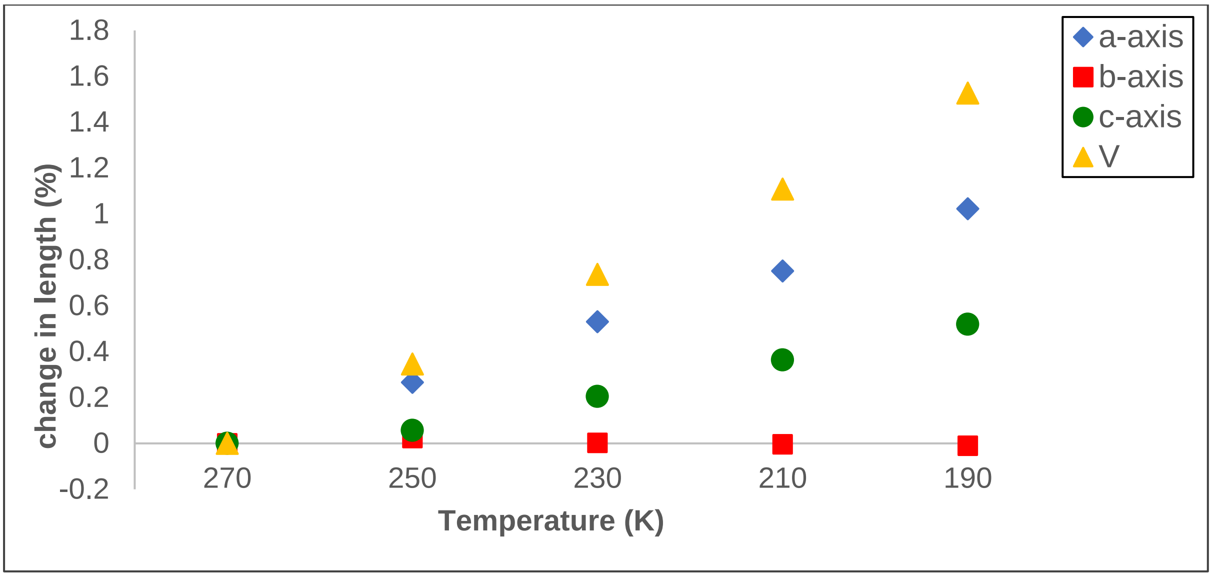

Figure S40. Percent change in length as a function of temperature for **olefin-I**.

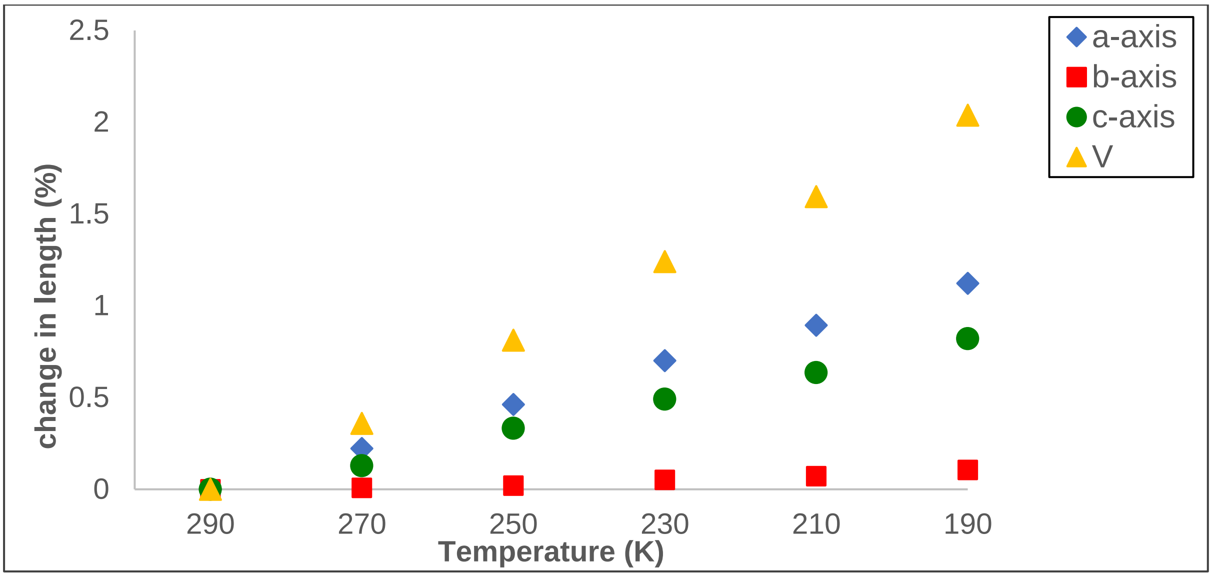

Figure S41. Percent change in length as a function of temperature for **olefin-Br**.

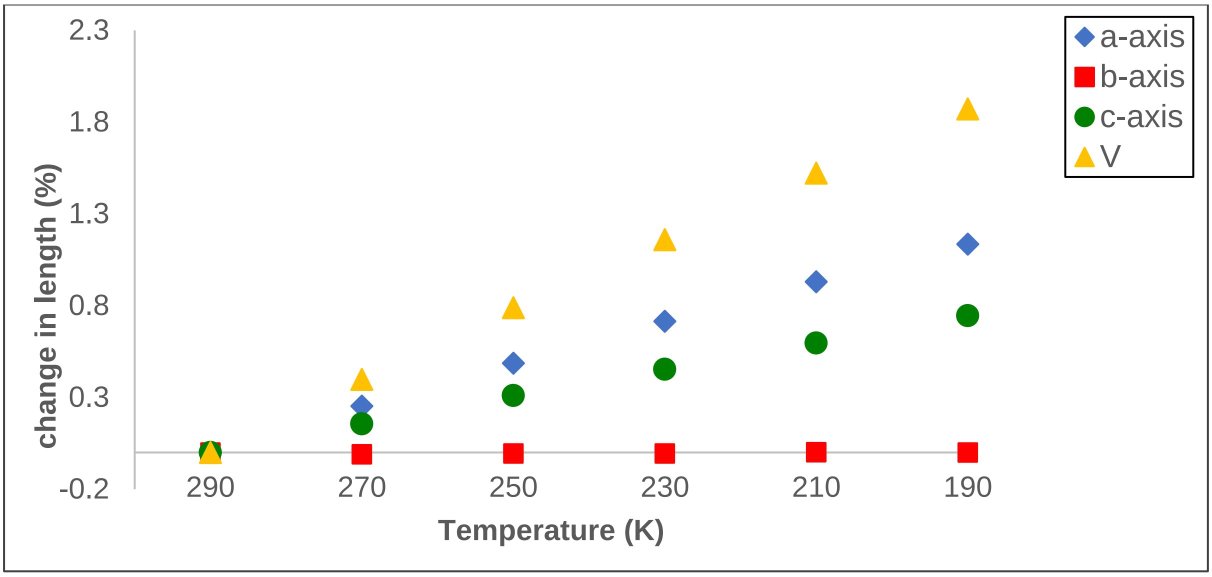

Figure S42. Percent change in length as a function of temperature for **olefin-I Br**.

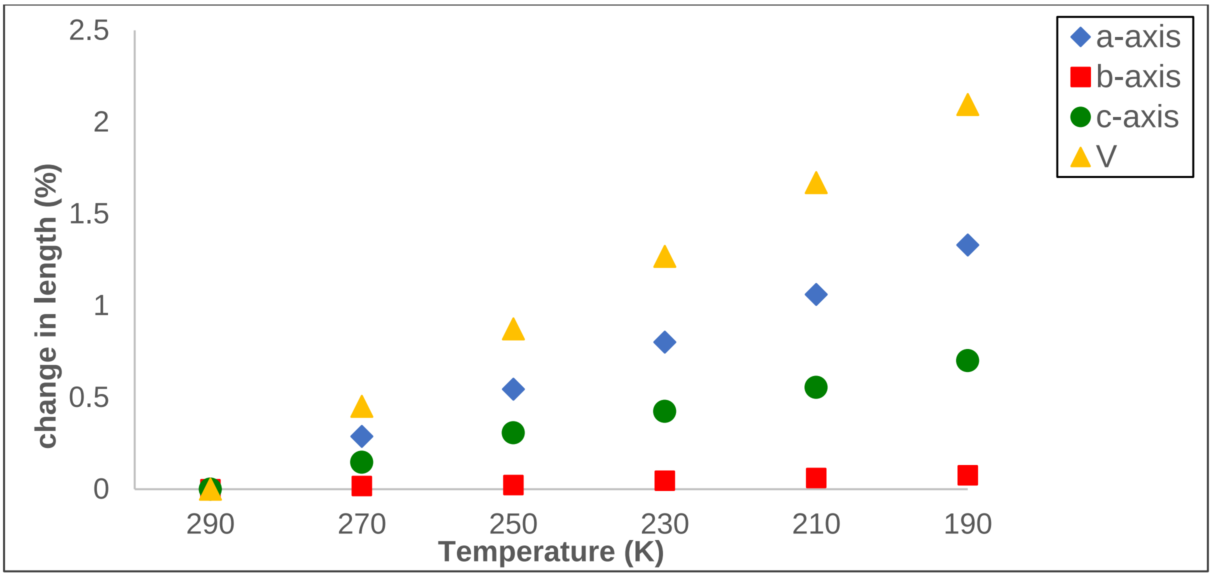

Figure S43. Percent change in length as a function of temperature for **imine-I**.

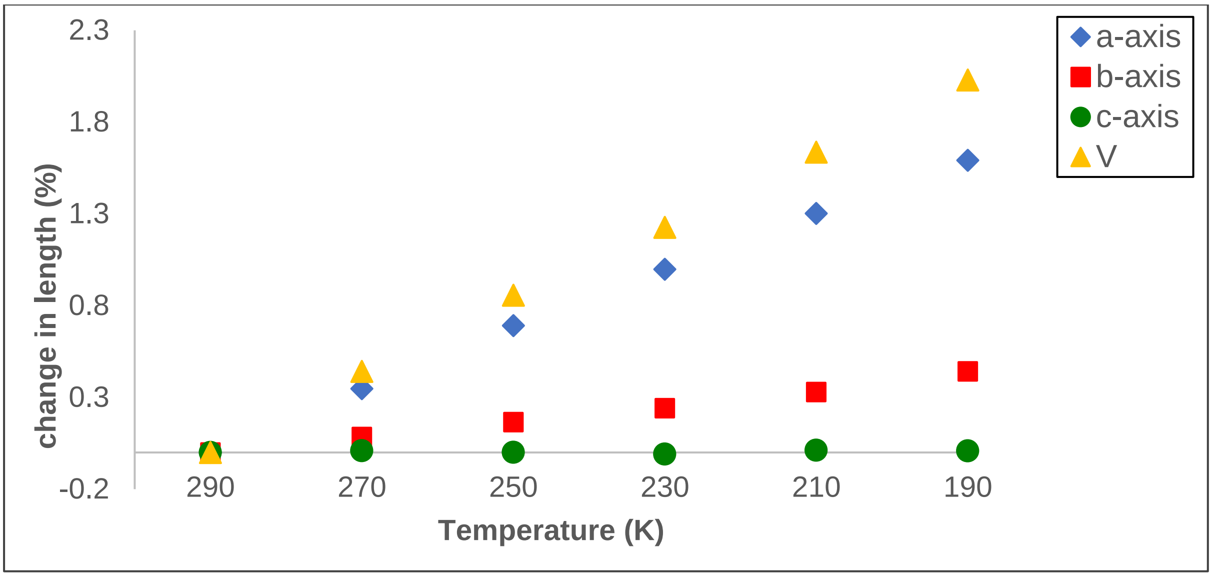

Figure S44. Percent change in length as a function of temperature for **imine-Br**.

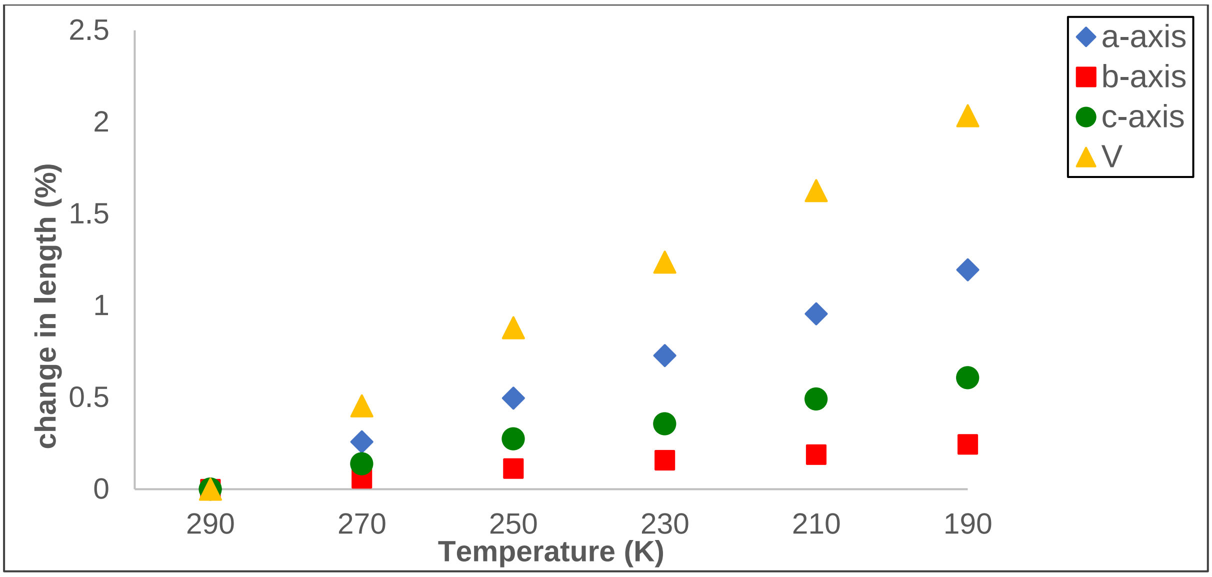

Figure S45. Percent change in length as a function of temperature for **azo-I**.

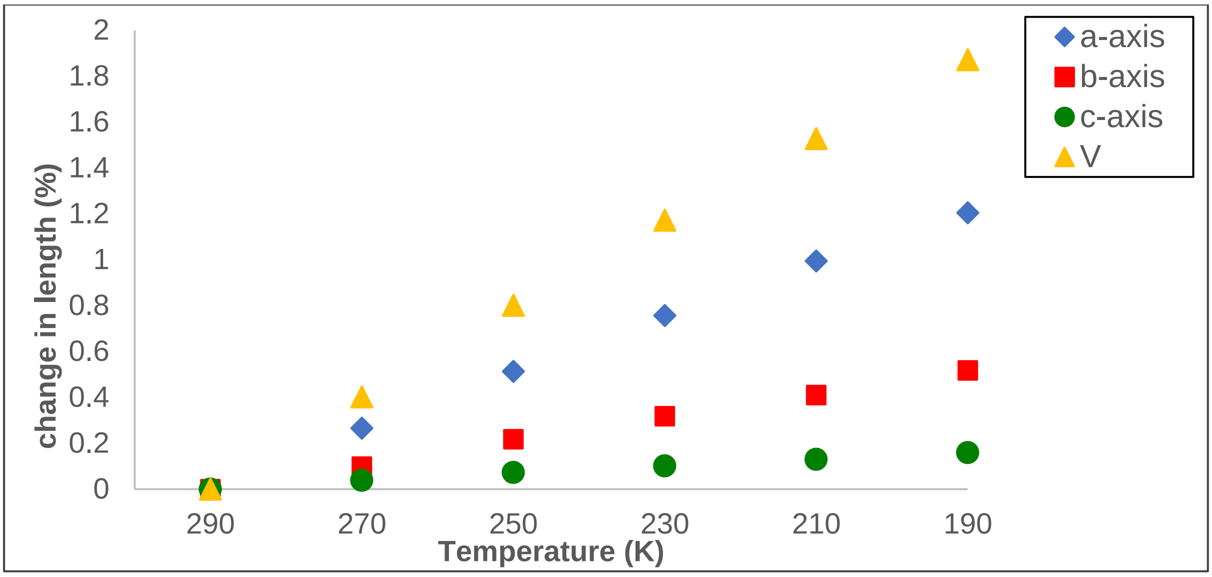

Figure S46. Percent change in length as a function of temperature for **azo-Br**(a).

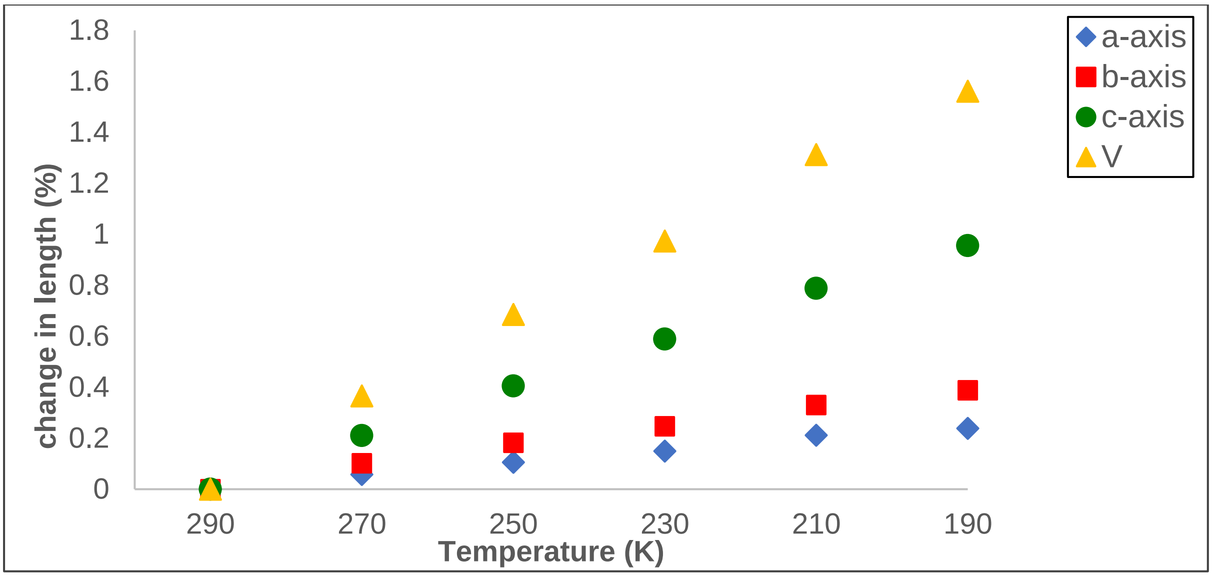

Figure S47. Percent change in length as a function of temperature for **azo-Br**(b).

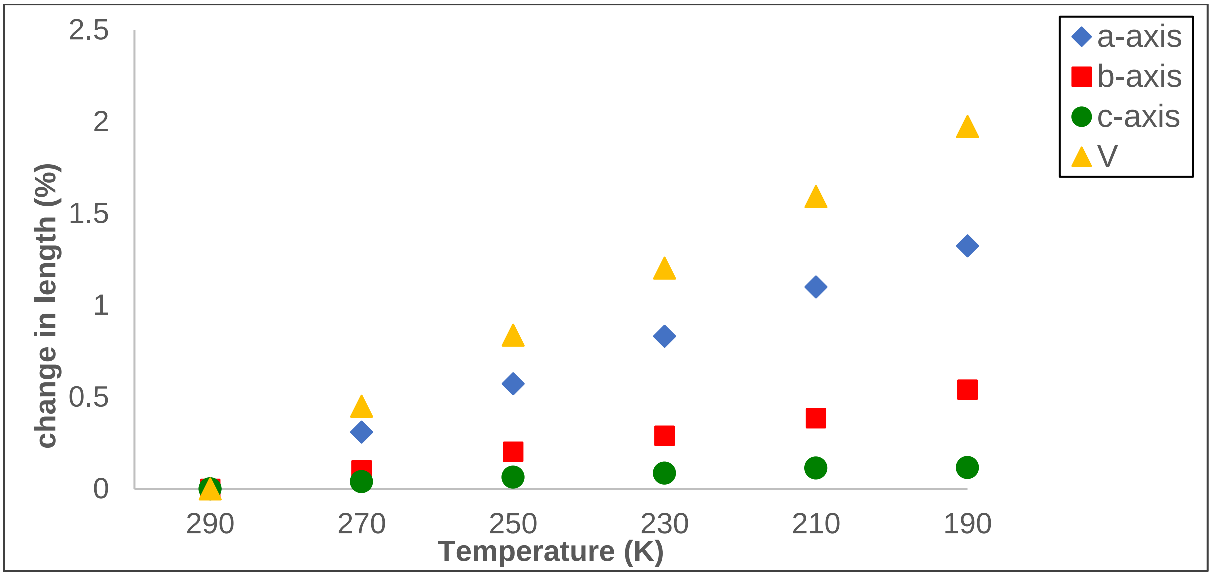

Figure S48. Percent change in length as a function of temperature for **azo-I Br**.

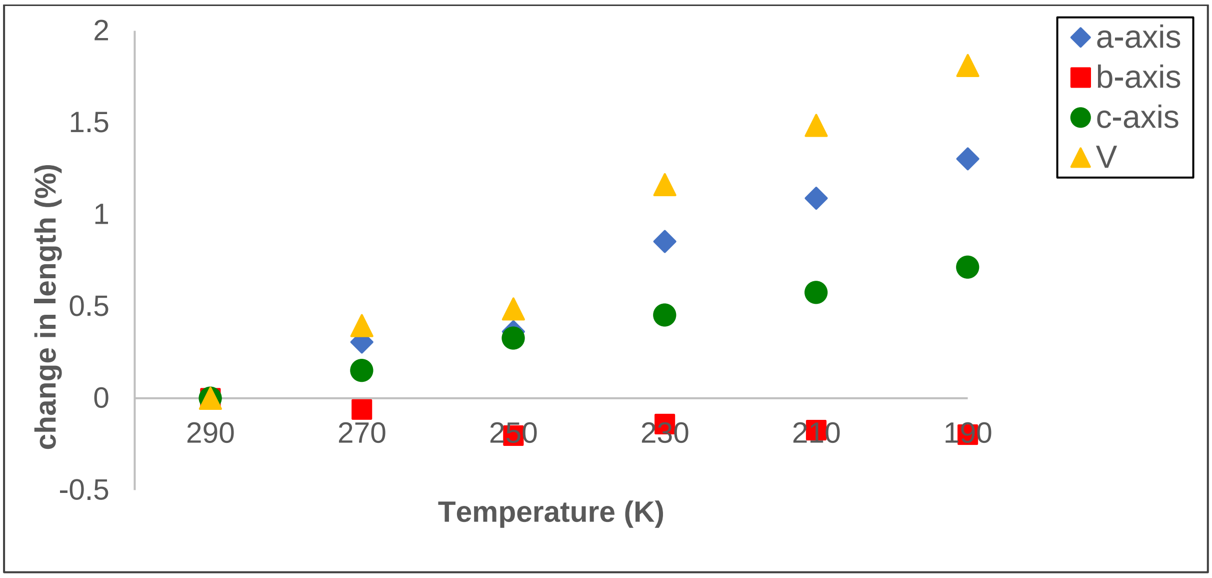

Figure S49. Percent change in length as a function of temperature for **diolefin-I**.

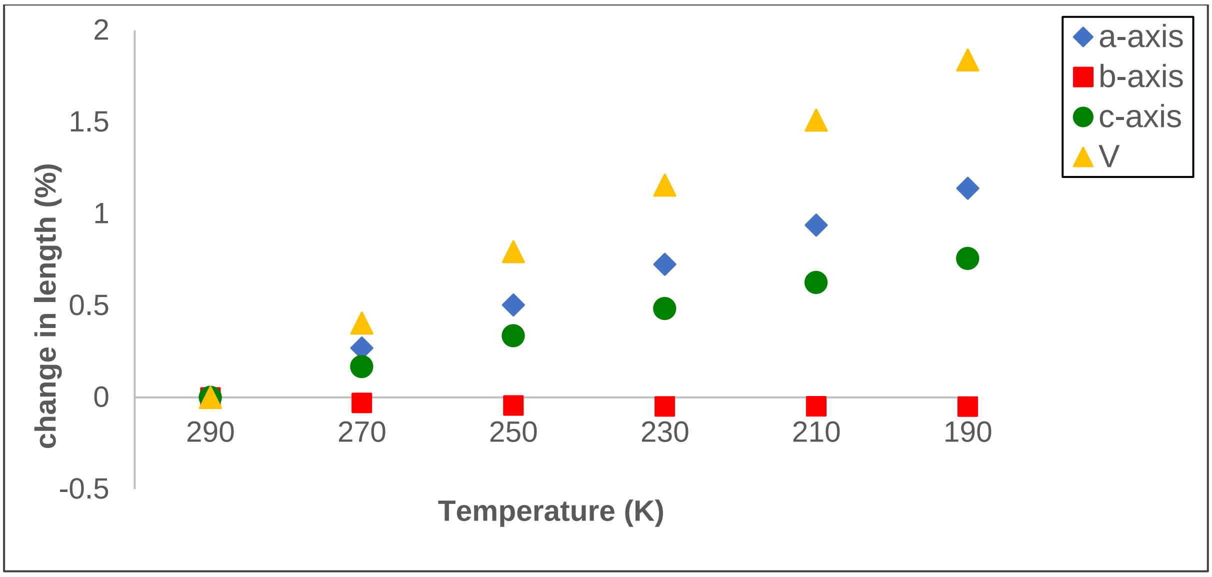

Figure S50. Percent change in length as a function of temperature for **diolefin-Br**.

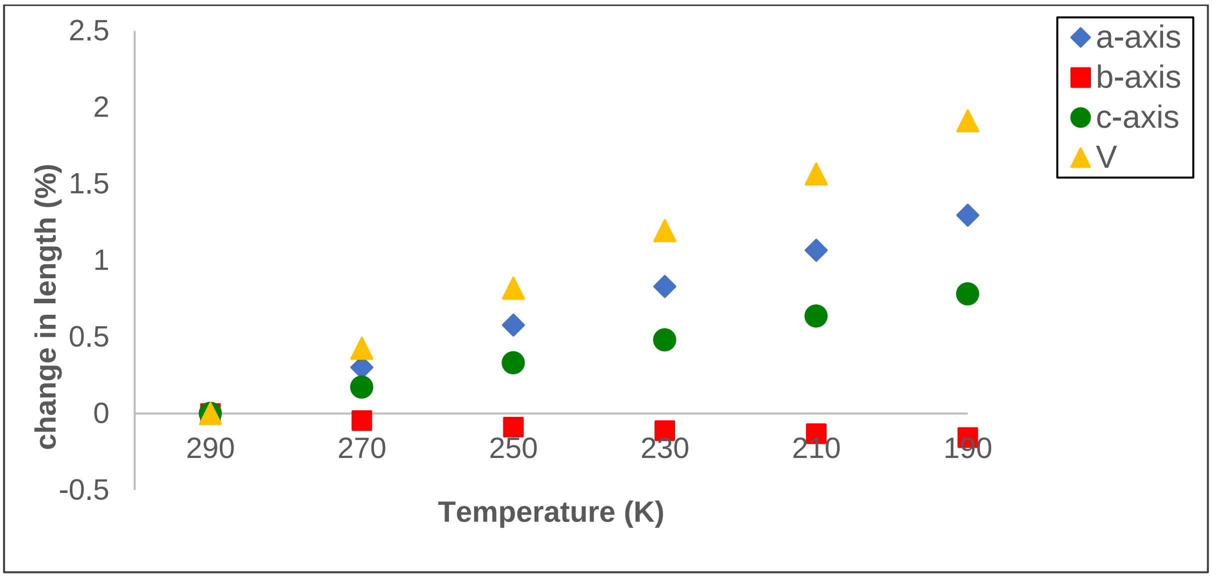

Figure S51. Percent change in length as a function of temperature for **diolefin-I Br**.

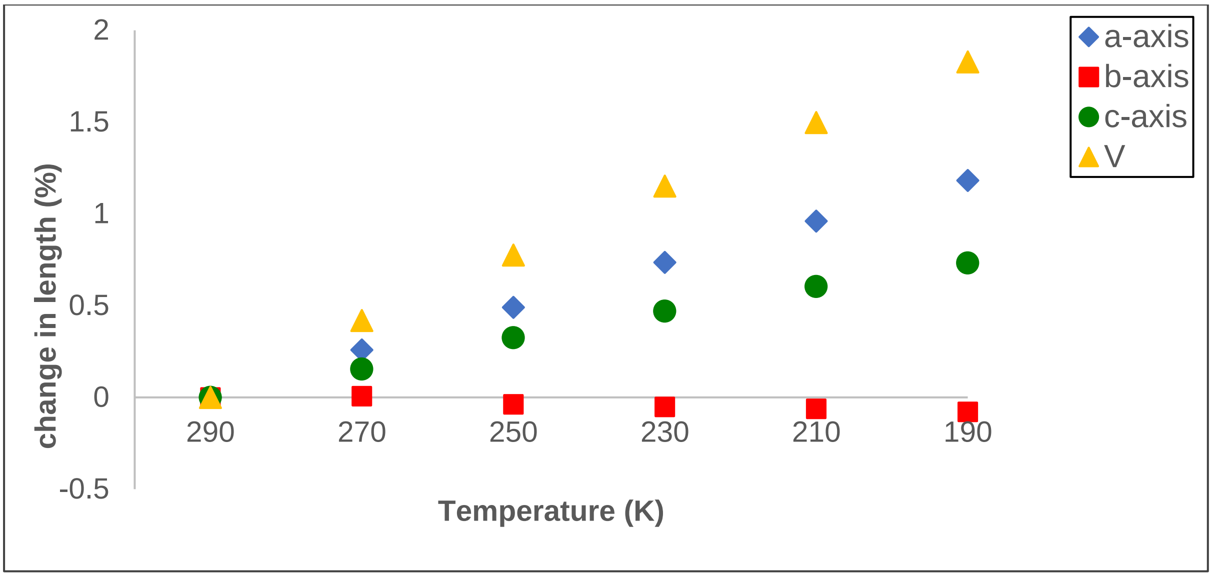

Figure S52. Percent change in length as a function of temperature for **diimine-I**.

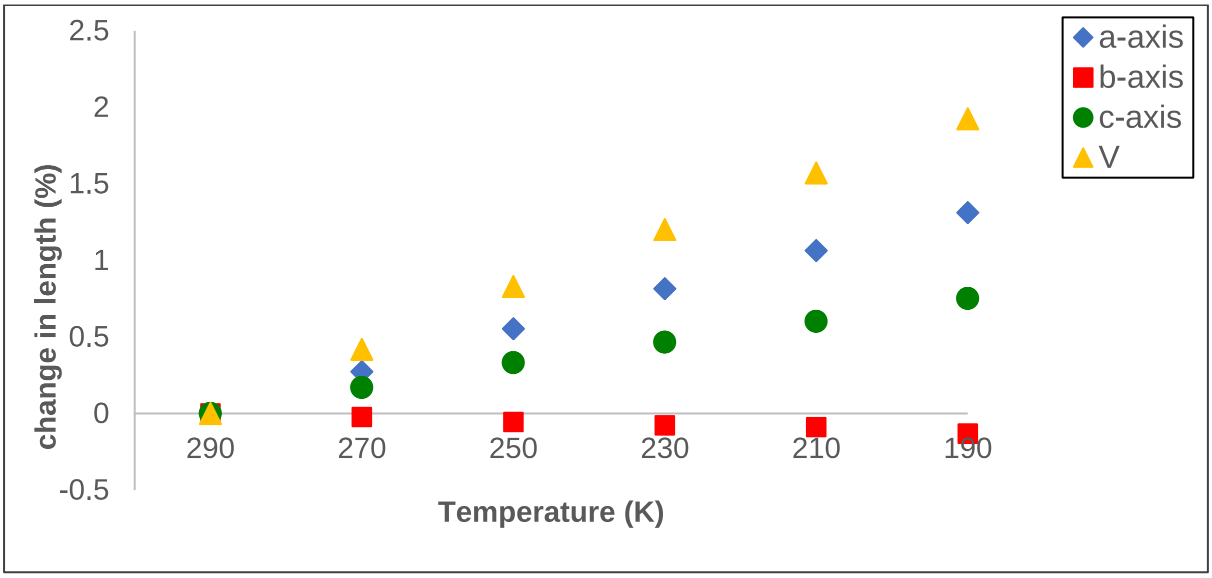

Figure S53. Percent change in length as a function of temperature for **diimine-Br**.

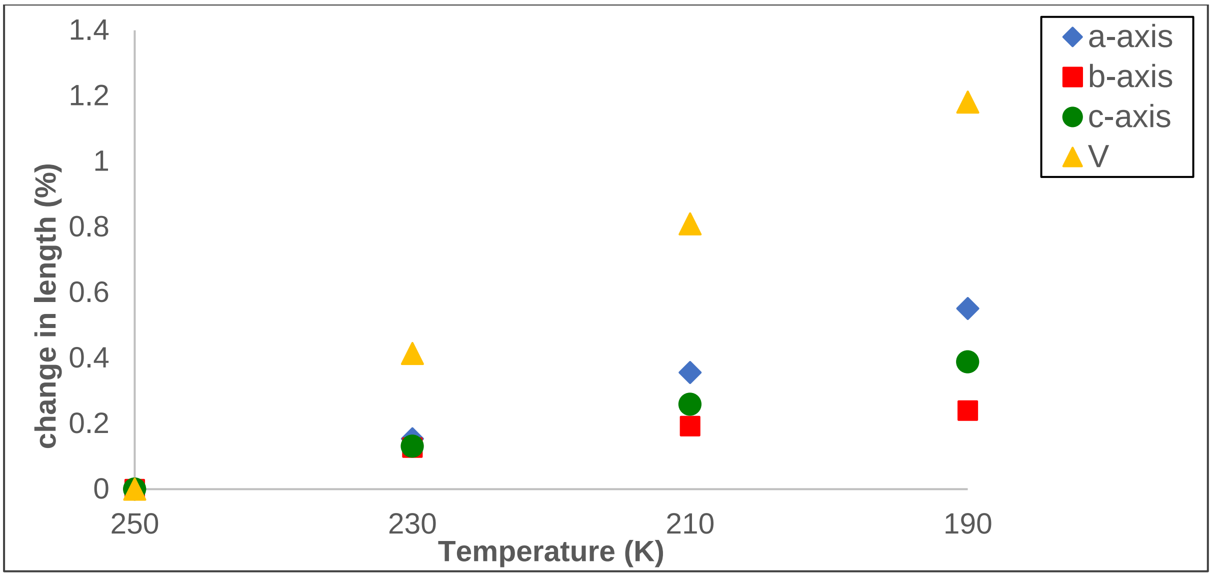

Figure S54. Percent change in length as a function of temperature for **diazo-I**.

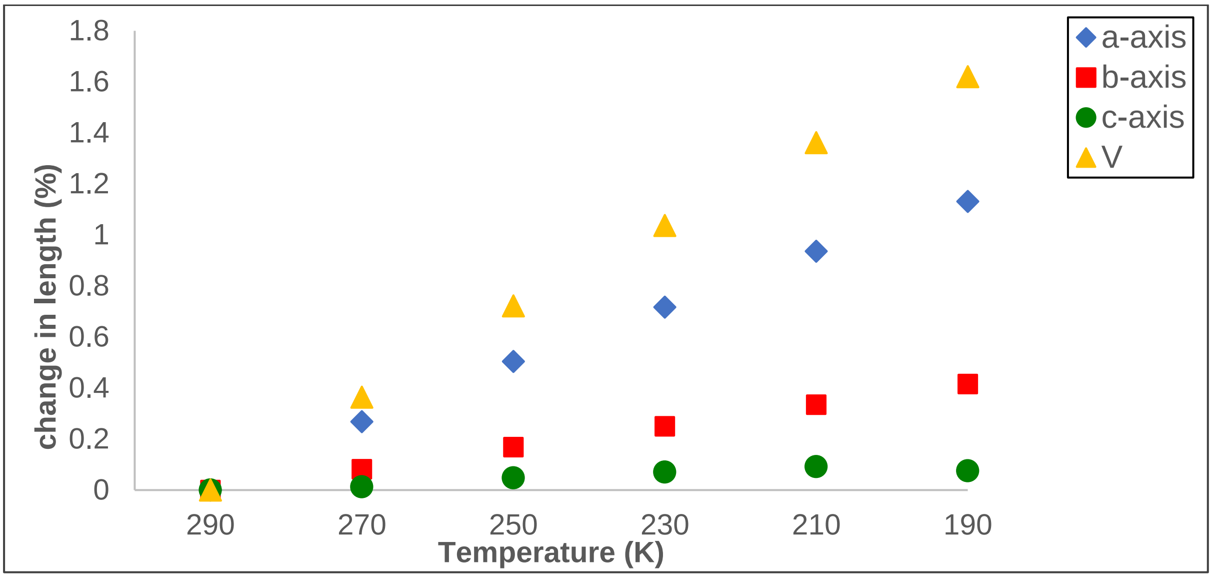

Figure S55. Percent change in length as a function of temperature for **diazo-Br**.

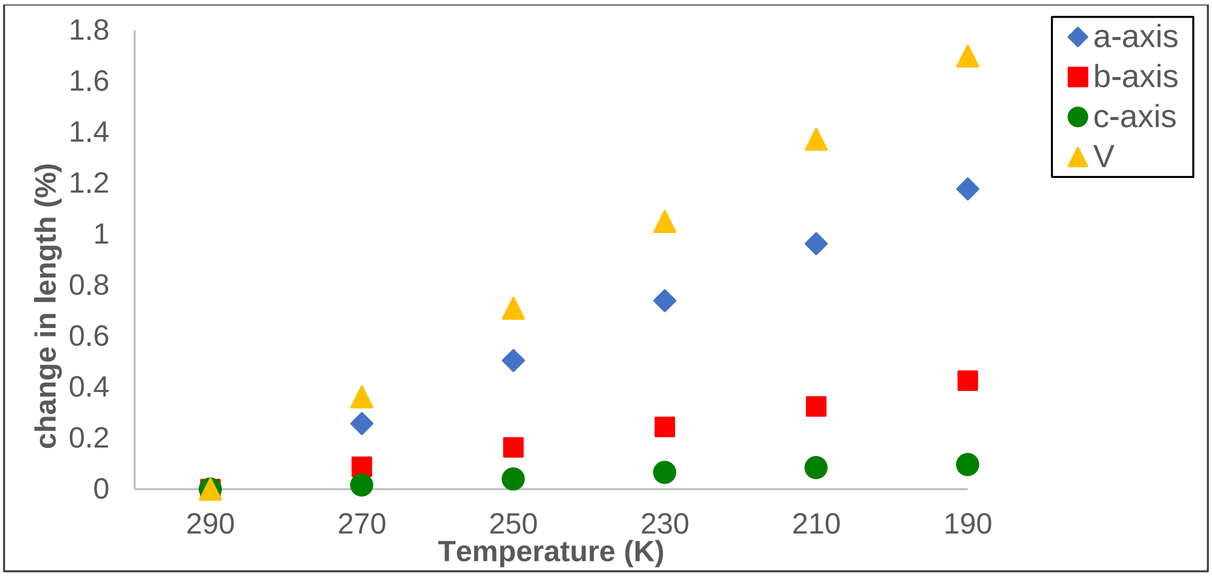

Figure S56. Percent change in length as a function of temperature for **diazo-I Br**.

8. van't Hoff Plots[14]

The van't Hoff plots for the solids that exhibit disorder are shown below. The plot was not done for the compound **diolefin-Br** that only exhibits disorder at two temperatures. Linear fits are provided on the graphs.

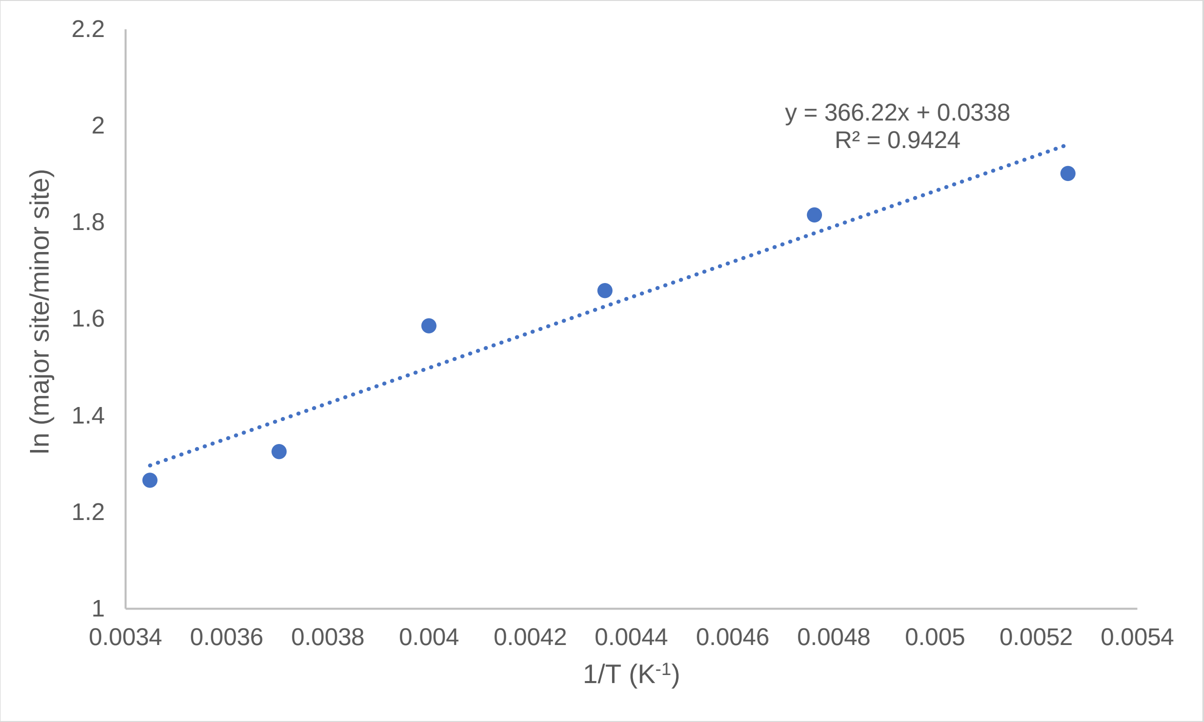

Figure S57. van't Hoff plot for **imine-I**.

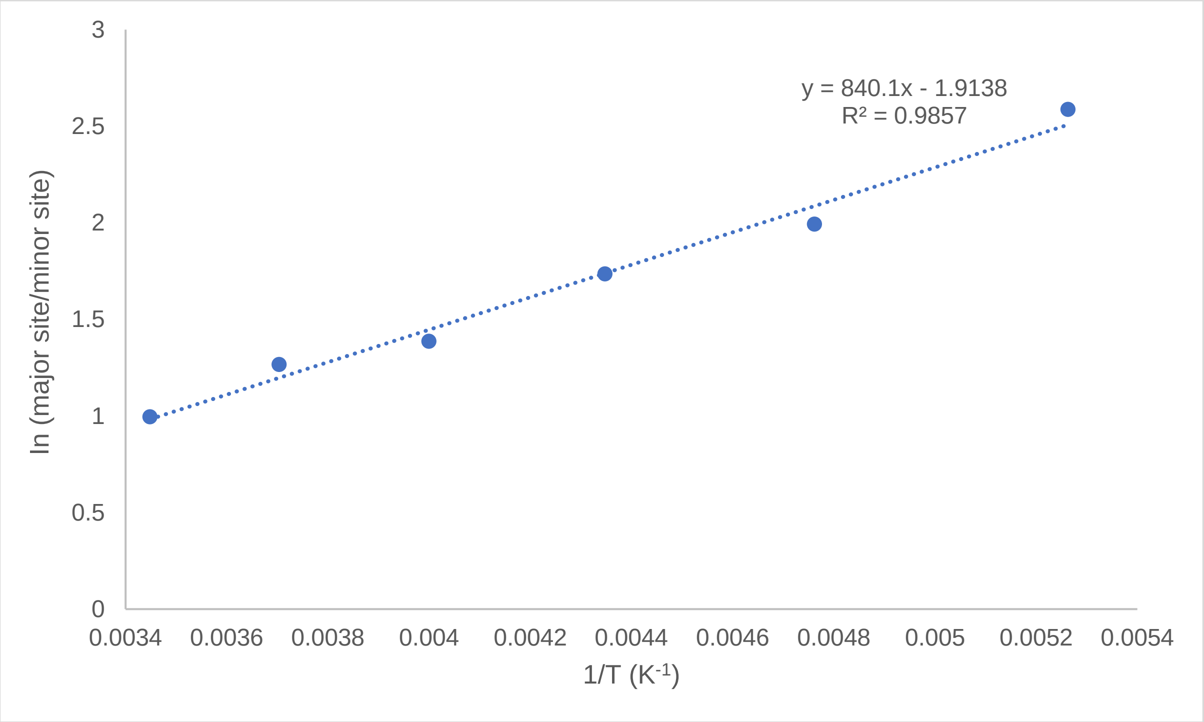

Figure S58. van't Hoff plot for **azo-I**.

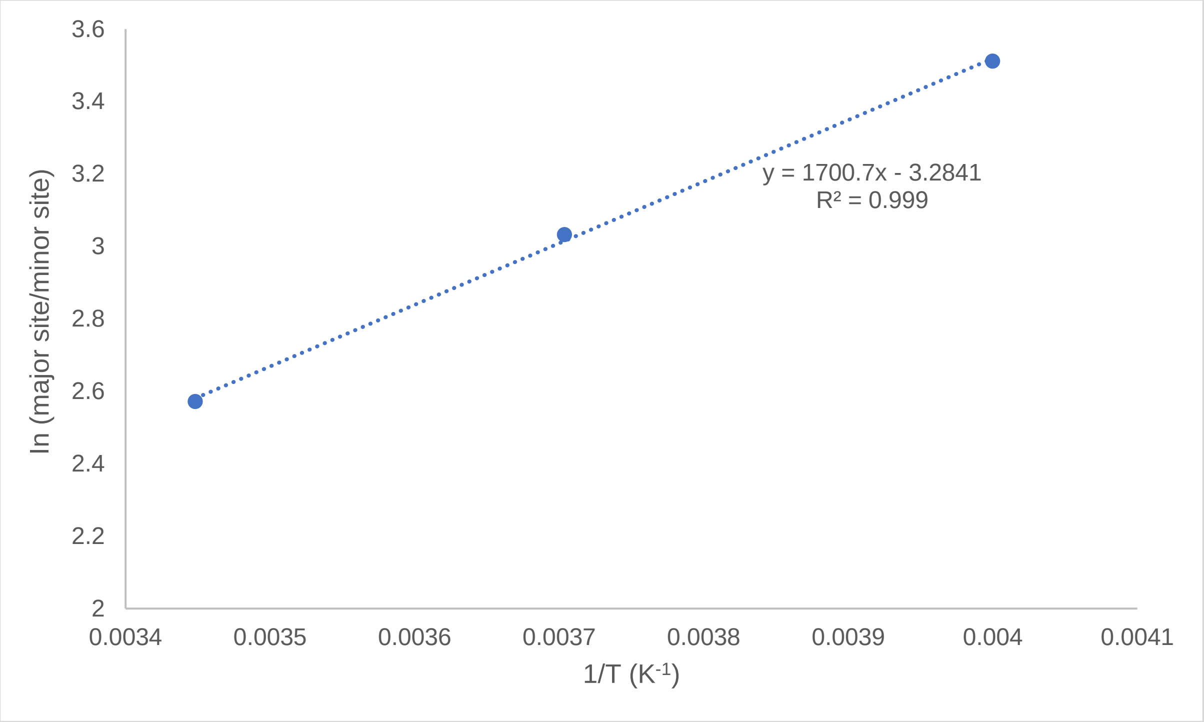

Figure S59. van’t Hoff plot for **diolefin-I Br**.

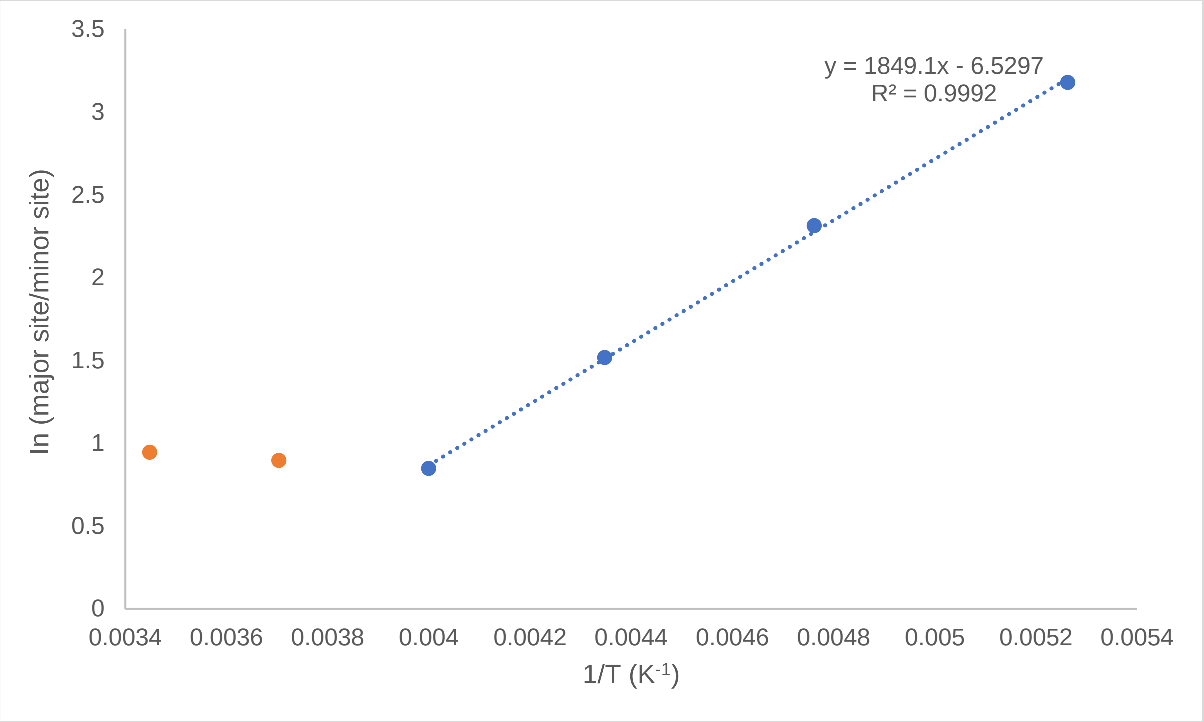

Figure S60. van’t Hoff plot for **diazo-I**. Data is only plotted for the molecule that exhibits disorder over the entire temperature range. The linear fit is only for the blue data points (250-190 K), and the orange data points show the 290-270 K data.

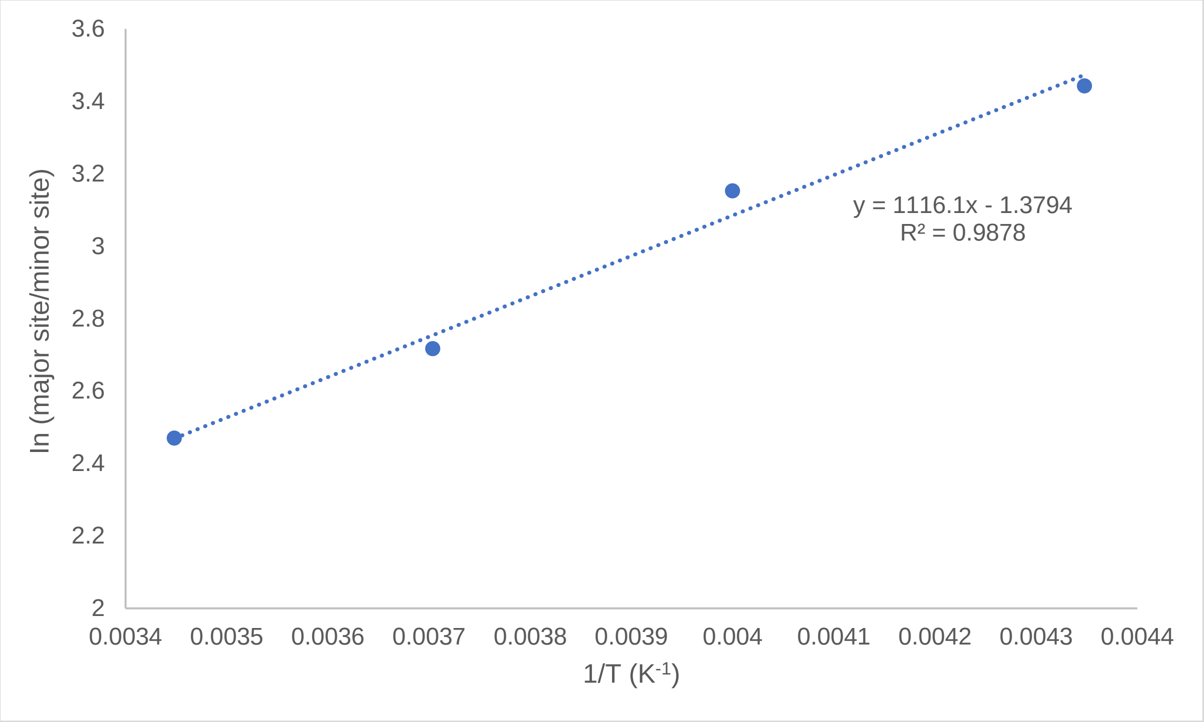

Figure S61. van’t Hoff plot for **azo-Br**(b).

9. Single-Crystal X-ray Structures of diazo-I

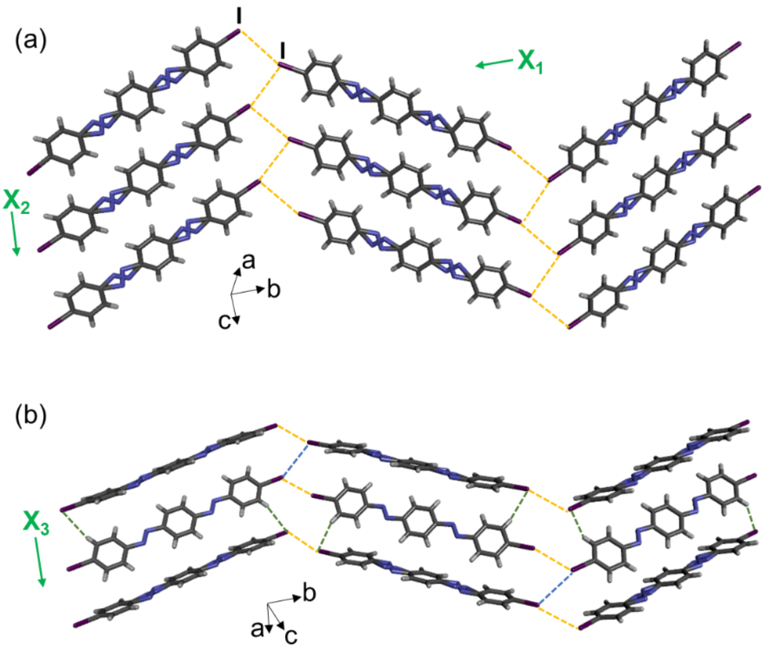

Figure S62. Single-crystal X-ray structures at 250 K showing 2D halogen-bonded sheet, herringbone packing, and TE axes for diazo-I. Disorder in aromatic rings is omitted for clarity. Type II halogen···halogen bonds shown with yellow dashed lines, type I bonds shown with blue dashed lines, and C-H···X bonds shown with green dashed lines.

10. Single-Crystal X-ray Structures and Analysis of azo-Br(b)

The polymorph **azo-Br**(b) crystallizes in an arrangement that is different from the other 16 solids discussed in the main text. The X-ray tables for **azo-Br**(b) are Tables S15-S16. The relevant figures in each section above are Figure S8, S47, and S61. The morphology difference between two polymorphs of **azo-Br** is shown below.

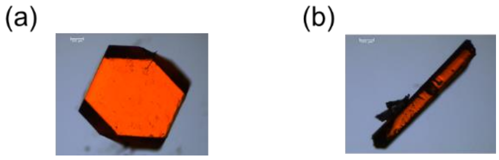

Figure S63. Microscopic image of (a) **azo-Br**(a) and (b) **azo-Br**(b).

Azo-Br(b) exhibits disorder over part of the temperature range studied (Figure S64), and the site occupancies of the major conformations are listed below.

**Temperature**	290 K	270 K	250 K	230 K	210 K	190 K
major occupancies	0.922(9)	0.938(8)	0.959(5)	0.969(4)	1.00	1.00

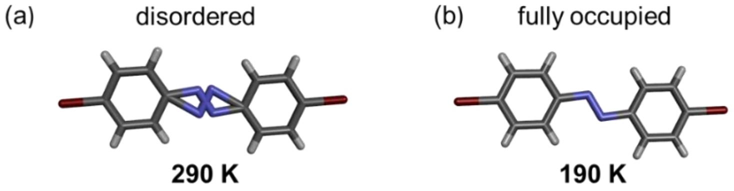

Figure S64. X-ray crystal structures at 290 and 190 K highlighting resolved disorder within **azo-Br**(b). Disorder is only shown for the bridge group for clarity.

The extended structure of **azo-Br**(b) is sustained by type II Br···Br halogen bonds to form a 2D halogen-bonded sheet that extends in the *ab* plane. The neighboring halogen-bonded molecules within the sheet are significantly deviated from planarity by 87°, resulting in formation of a zigzag-shaped sheet (Figure S65a). The sheets interact with each other via C-H···Br, Br···π, and π···π interactions (Figure S65b).

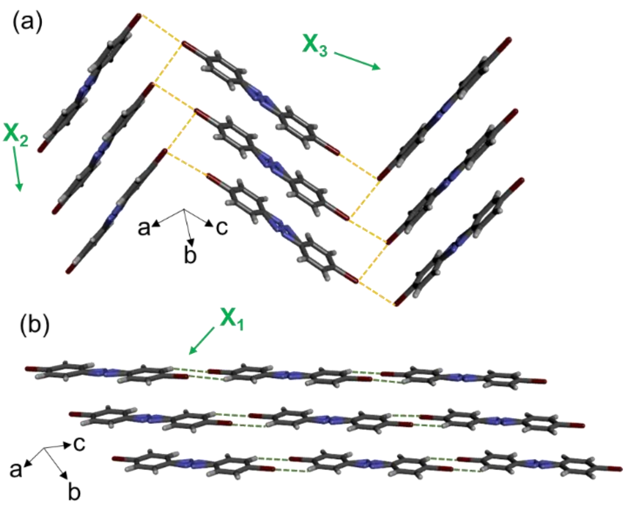

Figure S65. Single-crystal X-ray structures at 290 K showing stacked layers and TE axes for **azo-Br**(b). Disorder in aromatic rings is omitted for clarity. Type II halogen···halogen bonds shown with yellow dashed lines and C-H···X bonds shown with green dashed lines.

<u>TE analysis</u>: The solid **azo-Br**(b), which is not isostructural to the other solids, experiences moderate PTE along X_1 and X_2, and just reaches the colossal threshold along X_3 (α_{X_3} = 100 MK^{-1}). Akin to the face-to-face stacked solids, the C-H···Br interactions also contribute to expansion along X_1 in **azo-Br**(b) (Figure S65b), and the bond length increases by 0.05 Å upon heating. However, the interactions lying along X_2 and X_3 differ from the other molecules. The π···π stacking contributes to TE along X_2 in **azo-Br**(b) (Figure S65a), and the π···π distance increases by 0.02 Å upon heating. The direction of dynamic pedal motion in **azo-Br**(b) also lies along X_2; however, the impact of pedal motion which could lead to large PTE is offset by the small change in π···π distance. The type II Br···Br contacts also contribute slightly to TE along X_2 and are primarily included along X_3. The Br···Br separation increases by 0.04 Å upon warming.

11. References

(1) Wang, Q.; Ligorio, G.; Diez-Cabanes, V.; Cornil, D.; Kobin, B.; Hildebrandt, J.; Nardi M. V.; Timpel, M.; Hecht, S.; Cornil, J.; List-Kratochvil, E. J. W.; Koch, N. *Adv. Funct. Mater.* **2018**, *28*, 1800716.

(2) Wang, H.; Chen, Y.; Chen, Y.; Chen, C.; Lee, R.; Chan, L.; Jeng, R. *Polymer* **2012**, *53*, 4091-4103.

(3) Mizoshita, N.; Goto, Y.; Tani, T; Inagaki, S. *Adv. Funct. Mater*. **2008**, *18*, 3699-3705.

(4) Marin, L.; Harabagiu, V.; Lee, A. V. D.; Arvinte, A.; Barboiu, M. *J. Mol. Struct.* **2013**, *1049*, 377-385.

(5) Huskić, I.; Halasz, I.; Friščić, T.; Vančik, H. *Green Chem*. **2012**, *14*, 1597-1600.

(6) Nguyen, T. T. T.; Türp, D.; Wang, D.; Nölscher, B.; Laquai, F.; Müllen, K. *J. Am. Chem. Soc.* **2011**, *133*, 11194-11204.

(7) Amit, A. G.; Hope. H. *Acta Chem. Scand.* **1966**, *20*, 835-844.

(8) Albers, H. M. H. G.; Hendrickx, L. J. D.; van Tol, R. J. P.; Hausmann, J.; Perrakis, A.; Ovaa, H. *J. Med. Chem.* **2011**, *54*, 4619-4626.

(9) Tian, X.; Zhang, C.; Xu, Q.; Li, Z.; Shao, X. *Org. Biomol. Chem.* **2017**, *15*, 3320-3323.

(10) CrysAlis^{Pro} (2018) Oxford Diffraction Ltd.

(11) SCALE3 ABSPACK (2005) Oxford Diffraction Ltd.

(12) Sheldrick, G. M. Crystal structure refinement with SHELXL. *Acta. Cryst*. **2015**, *71*, 3-8.

(13) Cliffe, M. J.; Goodwin, A. L. *J. Appl. Cryst*. **2012**, *45*, 1321-1329.

(14) Harada, J.; Ogawa, K. *Chem. Soc. Rev*. **2009**, *38*, 2244-2252.
