# Supplementary material for: Differences in thermal expansion and motion ability for herringbone and face-to-face π-stacked solids
Source: IUCrJ. 2021 Nov 3;9(Pt 1):31–42. doi: 10.1107/S2052252521009593 (PMC8733877; doi:10.1107/S2052252521009593)

checkCIF/PLATON report

Structure factors have been supplied for datablock(s) azoBr_b_190K, azoBr_b_210K, azoBr_b_230K, azoBr_b_250K, azoBr_b_270K, azoBr_b_290K, azoIBr_190K, azoIBr_210K, azoIBr_230K, azoIBr_250K, azoIBr_270K, azoIBr_290K

THIS REPORT IS FOR GUIDANCE ONLY. IF USED AS PART OF A REVIEW PROCEDURE FOR PUBLICATION, IT SHOULD NOT REPLACE THE EXPERTISE OF AN EXPERIENCED CRYSTALLOGRAPHIC REFEREE.

No syntax errors found.   CIF dictionary   Interpreting this report

Datablock: azoBr_b_190K

```
Bond precision:  C-C = 0.0032 A          Wavelength=1.54178

Cell:           a=10.11009(11)    b=4.75046(5)      c=11.68261(13)
                alpha=90          beta=92.174(1)    gamma=90
Temperature:    190 K

                 Calculated                    Reported
Volume           560.684(11)                   560.683(11)
Space group      P 21/c                        P 21/c
Hall group       -P 2ybc                       -P 2ybc
Moiety formula   C12 H8 Br2 N2                 ?
Sum formula      C12 H8 Br2 N2                 C12 H8 Br2 N2
Mr               340.00                        340.02
Dx,g cm-3        2.014                         2.014
Z                2                             2
Mu (mm-1)        8.928                         8.928
F000             328.0                         328.0
F000'            325.83
h,k,lmax         12,6,14                       12,5,14
Nref             1184                          1173
Tmin,Tmax        0.332,0.452                   0.147,0.508
Tmin'            0.128

Correction method= # Reported T Limits: Tmin=0.147 Tmax=0.508
AbsCorr = GAUSSIAN

Data completeness= 0.991          Theta(max)= 77.348

R(reflections)= 0.0227( 1133)     wR2(reflections)= 0.0615( 1173)

S = 1.109                   Npar= 74
```

The following ALERTS were generated. Each ALERT has the format
test-name_ALERT_alert-type_alert-level.
Click on the hyperlinks for more details of the test.

Alert level G

```
PLAT434_ALERT_2_G Short Inter HL..HL Contact Br1      ..Br1            3.55 Ang.
                                        -x,1/2+y,3/2-z  =      2_556 Check
PLAT434_ALERT_2_G Short Inter HL..HL Contact Br1      ..Br1            3.55 Ang.
                                       -x,-1/2+y,3/2-z  =      2_546 Check
PLAT912_ALERT_4_G Missing # of FCF Reflections Above STh/L=  0.600        10 Note
PLAT978_ALERT_2_G Number C-C Bonds with Positive Residual Density.         4 Info
```

---

0 **ALERT level A** = Most likely a serious problem - resolve or explain
0 **ALERT level B** = A potentially serious problem, consider carefully
0 **ALERT level C** = Check. Ensure it is not caused by an omission or oversight
4 **ALERT level G** = General information/check it is not something unexpected

0 ALERT type 1 CIF construction/syntax error, inconsistent or missing data
3 ALERT type 2 Indicator that the structure model may be wrong or deficient
0 ALERT type 3 Indicator that the structure quality may be low
1 ALERT type 4 Improvement, methodology, query or suggestion
0 ALERT type 5 Informative message, check

Datablock: azoBr_b_210K

Bond precision: C-C = 0.0030 A   Wavelength=1.54178

Cell:	a=10.11287(8)	b=4.75323(4)	c=11.70238(10)
	alpha=90	beta=92.2133(7)	gamma=90
Temperature:	210 K		

	Calculated	Reported
Volume	562.100(8)	562.100(8)
Space group	P 21/c	P 21/c
Hall group	-P 2ybc	-P 2ybc
Moiety formula	C12 H8 Br2 N2	?
Sum formula	C12 H8 Br2 N2	C12 H8 Br2 N2
Mr	340.00	340.02
Dx,g cm-3	2.009	2.009
Z	2	2
Mu (mm-1)	8.906	8.906
F000	328.0	328.0
F000’	325.83	
h,k,lmax	12,6,14	12,5,14
Nref	1185	1174
Tmin,Tmax	0.333,0.453	0.140,0.516
Tmin’	0.128	

```
Correction method= # Reported T Limits: Tmin=0.140 Tmax=0.516
AbsCorr = GAUSSIAN

Data completeness= 0.991          Theta(max)= 77.248

R(reflections)= 0.0208( 1136)     wR2(reflections)= 0.0558( 1174)

S = 0.999                   Npar= 74
```

The following ALERTS were generated. Each ALERT has the format
test-name_ALERT_alert-type_alert-level.
Click on the hyperlinks for more details of the test.

Alert level G

```
PLAT142_ALERT_4_G s.u. on b - Axis Small or Missing .............    0.00004 Ang.
PLAT143_ALERT_4_G s.u. on c - Axis Small or Missing .............    0.00010 Ang.
PLAT434_ALERT_2_G Short Inter HL..HL Contact Br1      ..Br1             3.55 Ang.
                                             -x,1/2+y,3/2-z  =      2_556 Check
PLAT434_ALERT_2_G Short Inter HL..HL Contact Br1      ..Br1             3.55 Ang.
                                            -x,-1/2+y,3/2-z  =      2_546 Check
PLAT912_ALERT_4_G Missing # of FCF Reflections Above STh/L=  0.600         10 Note
PLAT978_ALERT_2_G Number C-C Bonds with Positive Residual Density.          5 Info
```

---

0 **ALERT level A** = Most likely a serious problem - resolve or explain
0 **ALERT level B** = A potentially serious problem, consider carefully
0 **ALERT level C** = Check. Ensure it is not caused by an omission or oversight
6 **ALERT level G** = General information/check it is not something unexpected

0 ALERT type 1 CIF construction/syntax error, inconsistent or missing data
3 ALERT type 2 Indicator that the structure model may be wrong or deficient
0 ALERT type 3 Indicator that the structure quality may be low
3 ALERT type 4 Improvement, methodology, query or suggestion
0 ALERT type 5 Informative message, check

Datablock: azoBr_b_230K

```
Bond precision:  C-C = 0.0000 A          Wavelength=1.54178

Cell:           a=10.11911(8)    b=4.75720(4)      c=11.72589(10)
                alpha=90         beta=92.2614(7)    gamma=90
Temperature:    230 K
```

	Calculated	Reported
Volume	564.029(8)	564.029(8)
Space group	P 21/c	P 21/c
Hall group	-P 2ybc	-P 2ybc
Moiety formula	C12 H8 Br2 N2	?
Sum formula	C12 H8 Br2 N2	C12 H8 Br2 N2
Mr	340.00	340.02
Dx,g cm-3	2.002	2.002
Z	2	2
Mu (mm-1)	8.875	8.875
F000	328.0	328.0
F000’	325.83	
h,k,lmax	12,6,14	12,5,14
Nref	1192	1176
Tmin,Tmax	0.334,0.454	0.139,0.518
Tmin’	0.129	

Correction method= # Reported T Limits: Tmin=0.139 Tmax=0.518
AbsCorr = GAUSSIAN

Data completeness= 0.987 Theta(max)= 77.284

R(reflections)= 0.0211(1128) wR2(reflections)= 0.0585(1176)

S = 1.086 Npar= 137

The following ALERTS were generated. Each ALERT has the format
test-name_ALERT_alert-type_alert-level.
Click on the hyperlinks for more details of the test.

Alert level C

```
PLAT088_ALERT_3_C Poor Data / Parameter Ratio ....................       8.58 Note
```

Author Response: Due to modeling every atom as a split site.

Alert level G

```
PLAT002_ALERT_2_G Number of Distance or Angle Restraints on AtSite          4 Note
PLAT003_ALERT_2_G Number of Uiso or Uij Restrained non-H Atoms ...         16 Report
PLAT142_ALERT_4_G s.u. on b - Axis Small or Missing .............    0.00004 Ang.
PLAT143_ALERT_4_G s.u. on c - Axis Small or Missing .............    0.00010 Ang.
PLAT172_ALERT_4_G The CIF-Embedded .res File Contains DFIX Records          2 Report
PLAT178_ALERT_4_G The CIF-Embedded .res File Contains SIMU Records          1 Report
PLAT187_ALERT_4_G The CIF-Embedded .res File Contains RIGU Records          1 Report
PLAT301_ALERT_3_G Main Residue  Disorder .............(Resd  1  )       100% Note
PLAT301_ALERT_3_G Main Residue  Disorder .............(Resd  2  )       100% Note
PLAT304_ALERT_4_G Non-Integer Number of Atoms in ..... (Resd  1  )      23.26 Check
PLAT304_ALERT_4_G Non-Integer Number of Atoms in ..... (Resd  2  )       0.74 Check
PLAT779_ALERT_4_G Suspect or Irrelevant (Bond) Angle(s) in CIF ...      36.50 Deg.
              C1B  -BR1B -C2B     1_555   1_555   1_555 ......     #   20 Check
```

```
PLAT860_ALERT_3_G Number of Least-Squares Restraints .............        360 Note
PLAT912_ALERT_4_G Missing # of FCF Reflections Above STh/L=  0.600         14 Note
PLAT978_ALERT_2_G Number C-C Bonds with Positive Residual Density.          7 Info
PLAT992_ALERT_5_G Repd & Actual _reflns_number_gt Values Differ by          2 Check
```

```
   0 ALERT level A = Most likely a serious problem - resolve or explain
   0 ALERT level B = A potentially serious problem, consider carefully
   1 ALERT level C = Check. Ensure it is not caused by an omission or oversight
  16 ALERT level G = General information/check it is not something unexpected

   0 ALERT type 1 CIF construction/syntax error, inconsistent or missing data
   3 ALERT type 2 Indicator that the structure model may be wrong or deficient
   4 ALERT type 3 Indicator that the structure quality may be low
   9 ALERT type 4 Improvement, methodology, query or suggestion
   1 ALERT type 5 Informative message, check
```

Datablock: azoBr_b_250K

```
Bond precision:  C-C = 0.0000 A          Wavelength=1.54178

Cell:           a=10.12356(8)    b=4.76029(4)       c=11.74756(11)
                alpha=90         beta=92.2993(8)    gamma=90
Temperature:    250 K

                 Calculated                  Reported
Volume           565.672(8)                  565.671(9)
Space group      P 21/c                      P 21/c
Hall group       -P 2ybc                     -P 2ybc
Moiety formula   C12 H8 Br2 N2               ?
Sum formula      C12 H8 Br2 N2               C12 H8 Br2 N2
Mr               340.00                      340.02
Dx,g cm-3        1.996                       1.996
Z                2                           2
Mu (mm-1)        8.850                       8.850
F000             328.0                       328.0
F000'            325.83
h,k,lmax         12,6,14                     12,5,14
Nref             1197                        1174
Tmin,Tmax        0.335,0.455                 0.135,0.528
Tmin'            0.130

Correction method= # Reported T Limits: Tmin=0.135 Tmax=0.528
AbsCorr = GAUSSIAN

Data completeness= 0.981          Theta(max)= 77.324

R(reflections)= 0.0218( 1128)     wR2(reflections)= 0.0616( 1174)
```

S = 1.113 Npar= 137

The following ALERTS were generated. Each ALERT has the format
test-name_ALERT_alert-type_alert-level.
Click on the hyperlinks for more details of the test.

Alert level C

```
PLAT088_ALERT_3_C Poor Data / Parameter Ratio ....................       8.57 Note
```

Author Response: Due to modeling every atom as a split site.

Alert level G

```
PLAT002_ALERT_2_G Number of Distance or Angle Restraints on AtSite          4 Note
PLAT003_ALERT_2_G Number of Uiso or Uij Restrained non-H Atoms ...         16 Report
PLAT142_ALERT_4_G s.u. on b - Axis Small or Missing .............     0.00004 Ang.
PLAT143_ALERT_4_G s.u. on c - Axis Small or Missing .............     0.00011 Ang.
PLAT172_ALERT_4_G The CIF-Embedded .res File Contains DFIX Records          2 Report
PLAT178_ALERT_4_G The CIF-Embedded .res File Contains SIMU Records          1 Report
PLAT187_ALERT_4_G The CIF-Embedded .res File Contains RIGU Records          1 Report
PLAT301_ALERT_3_G Main Residue  Disorder ..............(Resd  1  )       100% Note
PLAT301_ALERT_3_G Main Residue  Disorder ..............(Resd  2  )       100% Note
PLAT304_ALERT_4_G Non-Integer Number of Atoms in ..... (Resd  1  )      23.02 Check
PLAT304_ALERT_4_G Non-Integer Number of Atoms in ..... (Resd  2  )       0.98 Check
PLAT779_ALERT_4_G Suspect or Irrelevant (Bond) Angle(s) in CIF ...      35.50 Deg.
              C1B  -BR1B -C2B     1_555   1_555   1_555 ......     #   20 Check
PLAT860_ALERT_3_G Number of Least-Squares Restraints .............        360 Note
PLAT912_ALERT_4_G Missing # of FCF Reflections Above STh/L=  0.600         19 Note
PLAT978_ALERT_2_G Number C-C Bonds with Positive Residual Density.          4 Info
```

---

0 **ALERT level A** = Most likely a serious problem - resolve or explain
0 **ALERT level B** = A potentially serious problem, consider carefully
1 **ALERT level C** = Check. Ensure it is not caused by an omission or oversight
15 **ALERT level G** = General information/check it is not something unexpected

0 ALERT type 1 CIF construction/syntax error, inconsistent or missing data
3 ALERT type 2 Indicator that the structure model may be wrong or deficient
4 ALERT type 3 Indicator that the structure quality may be low
9 ALERT type 4 Improvement, methodology, query or suggestion
0 ALERT type 5 Informative message, check

Datablock: azoBr_b_270K

Bond precision: C-C = 0.0000 A Wavelength=1.54178

Cell:	a=10.12841(8)	b=4.76413(4)	c=11.77052(11)
	alpha=90	beta=92.3405(8)	gamma=90
Temperature:	270 K		

	Calculated	Reported
Volume	567.490(8)	567.489(8)
Space group	P 21/c	P 21/c
Hall group	-P 2ybc	-P 2ybc
Moiety formula	C12 H8 Br2 N2	?
Sum formula	C12 H8 Br2 N2	C12 H8 Br2 N2
Mr	340.00	340.02
Dx,g cm-3	1.990	1.990
Z	2	2
Mu (mm-1)	8.821	8.821
F000	328.0	328.0
F000’	325.83	
h,k,lmax	12,6,14	12,5,14
Nref	1200	1185
Tmin,Tmax	0.337,0.456	0.137,0.542
Tmin’	0.131	

Correction method= # Reported T Limits: Tmin=0.137 Tmax=0.542
AbsCorr = GAUSSIAN

Data completeness= 0.988 Theta(max)= 77.248

R(reflections)= 0.0232(1120) wR2(reflections)= 0.0628(1185)

S = 1.085 Npar= 128

The following ALERTS were generated. Each ALERT has the format
test-name_ALERT_alert-type_alert-level.
Click on the hyperlinks for more details of the test.

Alert level C

```
PLAT088_ALERT_3_C Poor Data / Parameter Ratio ....................       9.26 Note
```

Author Response: Due to modeling carbon and nitrogen atoms as split sites.

Alert level G

```
PLAT002_ALERT_2_G Number of Distance or Angle Restraints on AtSite          4 Note
PLAT003_ALERT_2_G Number of Uiso or Uij Restrained non-H Atoms ...         14 Report
PLAT142_ALERT_4_G s.u. on b - Axis Small or Missing .............    0.00004 Ang.
PLAT143_ALERT_4_G s.u. on c - Axis Small or Missing .............    0.00011 Ang.
PLAT172_ALERT_4_G The CIF-Embedded .res File Contains DFIX Records          2 Report
PLAT178_ALERT_4_G The CIF-Embedded .res File Contains SIMU Records          1 Report
PLAT187_ALERT_4_G The CIF-Embedded .res File Contains RIGU Records          1 Report
PLAT301_ALERT_3_G Main Residue  Disorder ..............(Resd  1  )        88% Note
PLAT434_ALERT_2_G Short Inter HL..HL Contact Br1      ..Br1             3.57 Ang.
                                         -x,-1/2+y,3/2-z  =      2_546 Check
PLAT434_ALERT_2_G Short Inter HL..HL Contact Br1      ..Br1             3.57 Ang.
                                          -x,1/2+y,3/2-z  =      2_556 Check
PLAT811_ALERT_5_G No ADDSYM Analysis: Too Many Excluded Atoms ....          ! Info
```

```
PLAT860_ALERT_3_G Number of Least-Squares Restraints .............        328 Note
PLAT912_ALERT_4_G Missing # of FCF Reflections Above STh/L=  0.600         11 Note
PLAT978_ALERT_2_G Number C-C Bonds with Positive Residual Density.          6 Info
```

```
   0 ALERT level A = Most likely a serious problem - resolve or explain
   0 ALERT level B = A potentially serious problem, consider carefully
   1 ALERT level C = Check. Ensure it is not caused by an omission or oversight
  14 ALERT level G = General information/check it is not something unexpected

   0 ALERT type 1 CIF construction/syntax error, inconsistent or missing data
   5 ALERT type 2 Indicator that the structure model may be wrong or deficient
   3 ALERT type 3 Indicator that the structure quality may be low
   6 ALERT type 4 Improvement, methodology, query or suggestion
   1 ALERT type 5 Informative message, check
```

Datablock: azoBr_b_290K

```
Bond precision:  C-C = 0.0000 A          Wavelength=1.54178

Cell:           a=10.13426(10)   b=4.76896(6)       c=11.79529(13)
                alpha=90         beta=92.3783(10)   gamma=90
Temperature:    290 K

                 Calculated                  Reported
Volume           569.574(11)                 569.574(11)
Space group      P 21/c                      P 21/c
Hall group       -P 2ybc                     -P 2ybc
Moiety formula   C12 H8 Br2 N2               ?
Sum formula      C12 H8 Br2 N2               C12 H8 Br2 N2
Mr               340.00                      340.02
Dx,g cm-3        1.982                       1.983
Z                2                           2
Mu (mm-1)        8.789                       8.789
F000             328.0                       328.0
F000'            325.83
h,k,lmax         12,6,14                     12,5,14
Nref             1205                        1153
Tmin,Tmax        0.338,0.457                 0.134,0.525
Tmin'            0.131

Correction method= # Reported T Limits: Tmin=0.134 Tmax=0.525
AbsCorr = GAUSSIAN

Data completeness= 0.957          Theta(max)= 77.237

R(reflections)= 0.0255( 1063)     wR2(reflections)= 0.0701( 1153)
```

```
S = 1.099                    Npar= 128
```

The following ALERTS were generated. Each ALERT has the format
test-name_ALERT_alert-type_alert-level.
Click on the hyperlinks for more details of the test.

Alert level C

```
PLAT088_ALERT_3_C Poor Data / Parameter Ratio ....................       9.01 Note
```

Author Response: Due to modeling carbon and nitrogen atoms as split sites. Due to modeling carbon and nitrogen atoms as split sites.

```
PLAT911_ALERT_3_C Missing FCF Refl Between Thmin & STh/L=    0.600          4 Report
```

Alert level G

```
PLAT002_ALERT_2_G Number of Distance or Angle Restraints on AtSite          4 Note
PLAT003_ALERT_2_G Number of Uiso or Uij Restrained non-H Atoms ...         14 Report
PLAT172_ALERT_4_G The CIF-Embedded .res File Contains DFIX Records          2 Report
PLAT178_ALERT_4_G The CIF-Embedded .res File Contains SIMU Records          1 Report
PLAT187_ALERT_4_G The CIF-Embedded .res File Contains RIGU Records          1 Report
PLAT301_ALERT_3_G Main Residue  Disorder ..............(Resd  1  )        88% Note
PLAT434_ALERT_2_G Short Inter HL..HL Contact Br1      ..Br1              3.58 Ang.
                                            -x,-1/2+y,3/2-z  =      2_546 Check
PLAT434_ALERT_2_G Short Inter HL..HL Contact Br1      ..Br1              3.58 Ang.
                                             -x,1/2+y,3/2-z  =      2_556 Check
PLAT811_ALERT_5_G No ADDSYM Analysis: Too Many Excluded Atoms ....          ! Info
PLAT860_ALERT_3_G Number of Least-Squares Restraints .............        328 Note
PLAT912_ALERT_4_G Missing # of FCF Reflections Above STh/L=  0.600         43 Note
PLAT978_ALERT_2_G Number C-C Bonds with Positive Residual Density.          5 Info
```

---

0 **ALERT level A** = Most likely a serious problem - resolve or explain
0 **ALERT level B** = A potentially serious problem, consider carefully
2 **ALERT level C** = Check. Ensure it is not caused by an omission or oversight
12 **ALERT level G** = General information/check it is not something unexpected

0 ALERT type 1 CIF construction/syntax error, inconsistent or missing data
5 ALERT type 2 Indicator that the structure model may be wrong or deficient
4 ALERT type 3 Indicator that the structure quality may be low
4 ALERT type 4 Improvement, methodology, query or suggestion
1 ALERT type 5 Informative message, check

Datablock: azoIBr_190K

Bond precision:	C-C = 0.0050 A		Wavelength=1.54178
Cell:	a=4.0347(3)	b=5.8485(4)	c=25.2404(18)
	alpha=90	beta=93.994(7)	gamma=90
Temperature:	190 K		

	Calculated	Reported
Volume	594.15(7)	594.16(7)
Space group	P 21/c	P 21/c
Hall group	-P 2ybc	-P 2ybc
Moiety formula	C12 H8 Br I N2	?
Sum formula	C12 H8 Br I N2	C12 H8 Br I N2
Mr	387.00	387.01
Dx,g cm-3	2.163	2.163
Z	2	2
Mu (mm-1)	24.886	24.886
F000	364.0	364.0
F000'	362.88	
h,k,lmax	5,7,31	4,7,31
Nref	1255	1223
Tmin,Tmax	0.101,0.706	0.368,1.000
Tmin'	0.028	

Correction method= # Reported T Limits: Tmin=0.368 Tmax=1.000
AbsCorr = GAUSSIAN

Data completeness= 0.975 Theta(max)= 77.327

R(reflections)= 0.0260(1146) wR2(reflections)= 0.0679(1223)

S = 1.250 Npar= 82

The following ALERTS were generated. Each ALERT has the format
test-name_ALERT_alert-type_alert-level.
Click on the hyperlinks for more details of the test.

Alert level G

```
PLAT230_ALERT_2_G Hirshfeld Test Diff for    Br1      --C1       .        5.5 s.u.
PLAT300_ALERT_4_G Atom Site Occupancy of I1         Constrained at        0.5 Check
PLAT300_ALERT_4_G Atom Site Occupancy of Br1        Constrained at        0.5 Check
PLAT301_ALERT_3_G Main Residue  Disorder .............(Resd  1  )        13% Note
PLAT779_ALERT_4_G Suspect or Irrelevant (Bond) Angle(s) in CIF ...      5.60 Deg.
              BR1  -C1   -I1      1_555   1_555   1_555 ......     #    7 Check
PLAT883_ALERT_1_G No Info/Value for _atom_sites_solution_primary .     Please Do !
PLAT912_ALERT_4_G Missing # of FCF Reflections Above STh/L=  0.600         16 Note
PLAT978_ALERT_2_G Number C-C Bonds with Positive Residual Density.          3 Info
```

---

0 **ALERT level A** = Most likely a serious problem - resolve or explain
0 **ALERT level B** = A potentially serious problem, consider carefully
0 **ALERT level C** = Check. Ensure it is not caused by an omission or oversight
8 **ALERT level G** = General information/check it is not something unexpected

1 ALERT type 1 CIF construction/syntax error, inconsistent or missing data
2 ALERT type 2 Indicator that the structure model may be wrong or deficient
1 ALERT type 3 Indicator that the structure quality may be low
4 ALERT type 4 Improvement, methodology, query or suggestion
0 ALERT type 5 Informative message, check

Datablock: azoIBr_210K

```
Bond precision:  C-C = 0.0040 A         Wavelength=1.54178

Cell:           a=4.04385(4)    b=5.85762(6)      c=25.2411(3)
                alpha=90        beta=93.965(1)    gamma=90
Temperature:    210 K
```

	Calculated	Reported
Volume	596.463(11)	596.463(10)
Space group	P 21/c	P 21/c
Hall group	-P 2ybc	-P 2ybc
Moiety formula	C12 H8 Br I N2	?
Sum formula	C12 H8 Br I N2	C12 H8 Br I N2
Mr	387.00	387.01
Dx,g cm-3	2.155	2.155
Z	2	2
Mu (mm-1)	24.789	24.789
F000	364.0	364.0
F000'	362.88	
h,k,lmax	5,7,31	4,7,31
Nref	1255	1227
Tmin,Tmax	0.102,0.707	0.344,1.000
Tmin'	0.028	

```
Correction method= # Reported T Limits: Tmin=0.344 Tmax=1.000
AbsCorr = GAUSSIAN

Data completeness= 0.978          Theta(max)= 77.019

R(reflections)= 0.0216( 1171)     wR2(reflections)= 0.0568( 1227)

S = 1.058                   Npar= 83
```

The following ALERTS were generated. Each ALERT has the format
test-name_ALERT_alert-type_alert-level.
Click on the hyperlinks for more details of the test.

Alert level G

```
PLAT230_ALERT_2_G Hirshfeld Test Diff for    I1       --C1       .        5.5 s.u.
PLAT230_ALERT_2_G Hirshfeld Test Diff for    Br1      --C1       .        6.5 s.u.
PLAT300_ALERT_4_G Atom Site Occupancy of I1        Constrained at        0.5 Check
PLAT300_ALERT_4_G Atom Site Occupancy of Br1       Constrained at        0.5 Check
PLAT301_ALERT_3_G Main Residue  Disorder .............(Resd  1  )        13% Note
PLAT779_ALERT_4_G Suspect or Irrelevant (Bond) Angle(s) in CIF ...      4.50 Deg.
              BR1  -C1   -I1      1_555   1_555   1_555 ......     #    7 Check
PLAT912_ALERT_4_G Missing # of FCF Reflections Above STh/L=  0.600        11 Note
PLAT933_ALERT_2_G Number of OMIT Records in Embedded .res File ...         1 Note
PLAT978_ALERT_2_G Number C-C Bonds with Positive Residual Density.         3 Info
```

```
   0 ALERT level A = Most likely a serious problem - resolve or explain
   0 ALERT level B = A potentially serious problem, consider carefully
   0 ALERT level C = Check. Ensure it is not caused by an omission or oversight
   9 ALERT level G = General information/check it is not something unexpected

   0 ALERT type 1 CIF construction/syntax error, inconsistent or missing data
   4 ALERT type 2 Indicator that the structure model may be wrong or deficient
   1 ALERT type 3 Indicator that the structure quality may be low
   4 ALERT type 4 Improvement, methodology, query or suggestion
   0 ALERT type 5 Informative message, check
```

Datablock: azoIBr_230K

```
Bond precision:  C-C = 0.0040 A          Wavelength=1.54178

Cell:            a=4.05483(5)    b=5.86325(7)        c=25.2481(3)
                 alpha=90        beta=93.9639(11)    gamma=90
Temperature:     230 K

                 Calculated                 Reported
Volume           598.825(12)                598.825(12)
Space group      P 21/c                     P 21/c
Hall group       -P 2ybc                    -P 2ybc
Moiety formula   C12 H8 Br I N2             ?
Sum formula      C12 H8 Br I N2             C12 H8 Br I N2
Mr               387.00                     387.01
Dx,g cm-3        2.146                      2.146
Z                2                          2
Mu (mm-1)        24.692                     24.692
F000             364.0                      364.0
F000'            362.88
h,k,lmax         5,7,31                     4,7,31
Nref             1266                       1236
Tmin,Tmax        0.103,0.708                0.347,1.000
Tmin'            0.029

Correction method= # Reported T Limits: Tmin=0.347 Tmax=1.000
AbsCorr = GAUSSIAN

Data completeness= 0.976          Theta(max)= 77.416

R(reflections)= 0.0228( 1166)     wR2(reflections)= 0.0591( 1236)

S = 1.100                  Npar= 83
```

The following ALERTS were generated. Each ALERT has the format
test-name_ALERT_alert-type_alert-level.
Click on the hyperlinks for more details of the test.

Alert level G

```
PLAT230_ALERT_2_G Hirshfeld Test Diff for    I1       --C1       .        5.0 s.u.
PLAT230_ALERT_2_G Hirshfeld Test Diff for    Br1      --C1       .        6.6 s.u.
PLAT300_ALERT_4_G Atom Site Occupancy of I1          Constrained at        0.5 Check
PLAT300_ALERT_4_G Atom Site Occupancy of Br1         Constrained at        0.5 Check
PLAT301_ALERT_3_G Main Residue  Disorder ..............(Resd  1  )        13% Note
PLAT779_ALERT_4_G Suspect or Irrelevant (Bond) Angle(s) in CIF ...       3.10 Deg.
              BR1  -C1   -I1      1_555   1_555   1_555 ......     #    7 Check
PLAT912_ALERT_4_G Missing # of FCF Reflections Above STh/L=  0.600         12 Note
PLAT978_ALERT_2_G Number C-C Bonds with Positive Residual Density.          2 Info
```

---

0 **ALERT level A** = Most likely a serious problem - resolve or explain
0 **ALERT level B** = A potentially serious problem, consider carefully
0 **ALERT level C** = Check. Ensure it is not caused by an omission or oversight
8 **ALERT level G** = General information/check it is not something unexpected

0 ALERT type 1 CIF construction/syntax error, inconsistent or missing data
3 ALERT type 2 Indicator that the structure model may be wrong or deficient
1 ALERT type 3 Indicator that the structure quality may be low
4 ALERT type 4 Improvement, methodology, query or suggestion
0 ALERT type 5 Informative message, check

Datablock: azoIBr_250K

Bond precision: C-C = 0.0050 A   Wavelength=1.54178

Cell:	a=4.06541(8)	b=5.86837(11)	c=25.2537(5)
	alpha=90	beta=93.9691(19)	gamma=90
Temperature:	250 K		

	Calculated	Reported
Volume	601.04(2)	601.04(2)
Space group	P 21/c	P 21/c
Hall group	-P 2ybc	-P 2ybc
Moiety formula	C12 H8 Br I N2	?
Sum formula	C12 H8 Br I N2	C12 H8 Br I N2
Mr	387.00	387.01
Dx,g cm-3	2.138	2.138
Z	2	2
Mu (mm-1)	24.601	24.601
F000	364.0	364.0
F000’	362.88	
h,k,lmax	5,7,31	4,7,31
Nref	1263	1236
Tmin,Tmax	0.104,0.709	0.377,1.000
Tmin’	0.029	

Correction method= # Reported T Limits: Tmin=0.377 Tmax=1.000
AbsCorr = GAUSSIAN

```
Data completeness= 0.979          Theta(max)= 76.833

R(reflections)= 0.0271( 1164)     wR2(reflections)= 0.0675( 1236)

S = 1.096                  Npar= 83
```

The following ALERTS were generated. Each ALERT has the format
test-name_ALERT_alert-type_alert-level.
Click on the hyperlinks for more details of the test.

Alert level C

```
PLAT911_ALERT_3_C Missing FCF Refl Between Thmin & STh/L=    0.600          3 Report
```

Alert level G

```
PLAT300_ALERT_4_G Atom Site Occupancy of I1         Constrained at        0.5 Check
PLAT300_ALERT_4_G Atom Site Occupancy of Br1        Constrained at        0.5 Check
PLAT301_ALERT_3_G Main Residue  Disorder ..............(Resd  1  )        13% Note
PLAT779_ALERT_4_G Suspect or Irrelevant (Bond) Angle(s) in CIF ...       2.00 Deg.
              BR1  -C1   -I1      1_555   1_555   1_555 ......     #    7 Check
PLAT912_ALERT_4_G Missing # of FCF Reflections Above STh/L=  0.600          7 Note
PLAT978_ALERT_2_G Number C-C Bonds with Positive Residual Density.          3 Info
```

---

0 **ALERT level A** = Most likely a serious problem - resolve or explain
0 **ALERT level B** = A potentially serious problem, consider carefully
1 **ALERT level C** = Check. Ensure it is not caused by an omission or oversight
6 **ALERT level G** = General information/check it is not something unexpected

0 ALERT type 1 CIF construction/syntax error, inconsistent or missing data
1 ALERT type 2 Indicator that the structure model may be wrong or deficient
2 ALERT type 3 Indicator that the structure quality may be low
4 ALERT type 4 Improvement, methodology, query or suggestion
0 ALERT type 5 Informative message, check

Datablock: azoIBr_270K

```
Bond precision:  C-C = 0.0050 A          Wavelength=1.54178

Cell:           a=4.07618(7)    b=5.87432(10)      c=25.2598(4)
                alpha=90        beta=93.9706(15)    gamma=90
Temperature:    270 K
```

	Calculated	Reported
Volume	603.389(18)	603.389(17)
Space group	P 21/c	P 21/c
Hall group	-P 2ybc	-P 2ybc
Moiety formula	C12 H8 Br I N2	?
Sum formula	C12 H8 Br I N2	C12 H8 Br I N2
Mr	387.00	387.01
Dx,g cm-3	2.130	2.130
Z	2	2
Mu (mm-1)	24.505	24.505
F000	364.0	364.0
F000’	362.88	
h,k,lmax	5,7,31	5,7,31
Nref	1272	1243
Tmin,Tmax	0.105,0.710	0.400,1.000
Tmin’	0.029	

Correction method= # Reported T Limits: Tmin=0.400 Tmax=1.000
AbsCorr = GAUSSIAN

Data completeness= 0.977   Theta(max)= 77.253

R(reflections)= 0.0277(1166)   wR2(reflections)= 0.0693(1243)

S = 1.092   Npar= 83

The following ALERTS were generated. Each ALERT has the format
test-name_ALERT_alert-type_alert-level.
Click on the hyperlinks for more details of the test.

Alert level C

```
PLAT911_ALERT_3_C Missing FCF Refl Between Thmin & STh/L=    0.600          3 Report
```

Alert level G

```
PLAT300_ALERT_4_G Atom Site Occupancy of I1         Constrained at        0.5 Check
PLAT300_ALERT_4_G Atom Site Occupancy of Br1        Constrained at        0.5 Check
PLAT301_ALERT_3_G Main Residue  Disorder .............(Resd  1  )        13% Note
PLAT779_ALERT_4_G Suspect or Irrelevant (Bond) Angle(s) in CIF ...       1.60 Deg.
              BR1  -C1   -I1      1_555   1_555   1_555 ......     #    7 Check
PLAT912_ALERT_4_G Missing # of FCF Reflections Above STh/L=  0.600         27 Note
PLAT978_ALERT_2_G Number C-C Bonds with Positive Residual Density.          3 Info
```

---

0 **ALERT level A** = Most likely a serious problem - resolve or explain
0 **ALERT level B** = A potentially serious problem, consider carefully
1 **ALERT level C** = Check. Ensure it is not caused by an omission or oversight
6 **ALERT level G** = General information/check it is not something unexpected

0 ALERT type 1 CIF construction/syntax error, inconsistent or missing data

1 ALERT type 2 Indicator that the structure model may be wrong or deficient
2 ALERT type 3 Indicator that the structure quality may be low
4 ALERT type 4 Improvement, methodology, query or suggestion
0 ALERT type 5 Informative message, check

Datablock: azoIBr_290K

Bond precision: C-C = 0.0050 A Wavelength=1.54178

Cell:	a=4.08885(7)	b=5.88027(10)	c=25.2697(4)
	alpha=90	beta=93.9641(16)	gamma=90
Temperature:	290 K		

	Calculated	Reported
Volume	606.120(18)	606.121(18)
Space group	P 21/c	P 21/c
Hall group	-P 2ybc	-P 2ybc
Moiety formula	C12 H8 Br I N2	?
Sum formula	C12 H8 Br I N2	C12 H8 Br I N2
Mr	387.00	387.01
Dx,g cm-3	2.121	2.121
Z	2	2
Mu (mm-1)	24.395	24.394
F000	364.0	364.0
F000’	362.88	
h,k,lmax	5,7,31	5,7,31
Nref	1280	1253
Tmin,Tmax	0.107,0.711	0.396,1.000
Tmin’	0.030	

Correction method= # Reported T Limits: Tmin=0.396 Tmax=1.000
AbsCorr = GAUSSIAN

Data completeness= 0.979 Theta(max)= 77.205

R(reflections)= 0.0284(1162) wR2(reflections)= 0.0722(1253)

S = 1.082 Npar= 83

The following ALERTS were generated. Each ALERT has the format
test-name_ALERT_alert-type_alert-level.
Click on the hyperlinks for more details of the test.

Alert level G

PLAT300_ALERT_4_G Atom Site Occupancy of I1 Constrained at 0.5 Check
PLAT300_ALERT_4_G Atom Site Occupancy of Br1 Constrained at 0.5 Check
PLAT301_ALERT_3_G Main Residue Disorder ....(Resd 1) 13% Note

```
PLAT779_ALERT_4_G Suspect or Irrelevant (Bond) Angle(s) in CIF ...       1.70 Deg.
              BR1  -C1   -I1      1_555   1_555   1_555 ......     #    7 Check
PLAT912_ALERT_4_G Missing # of FCF Reflections Above STh/L=  0.600         26 Note
PLAT978_ALERT_2_G Number C-C Bonds with Positive Residual Density.          3 Info
```

```
   0 ALERT level A = Most likely a serious problem - resolve or explain
   0 ALERT level B = A potentially serious problem, consider carefully
   0 ALERT level C = Check. Ensure it is not caused by an omission or oversight
   6 ALERT level G = General information/check it is not something unexpected

   0 ALERT type 1 CIF construction/syntax error, inconsistent or missing data
   1 ALERT type 2 Indicator that the structure model may be wrong or deficient
   1 ALERT type 3 Indicator that the structure quality may be low
   4 ALERT type 4 Improvement, methodology, query or suggestion
   0 ALERT type 5 Informative message, check
```

Validation response form

Please find below a validation response form (VRF) that can be filled in and pasted into your CIF.

```
# start Validation Reply Form
_vrf_PLAT911_azoBr_b_290K
;
PROBLEM: Missing FCF Refl Between Thmin & STh/L=    0.600          4 Report
RESPONSE: ...
;
_vrf_PLAT911_azoIBr_250K
;
PROBLEM: Missing FCF Refl Between Thmin & STh/L=    0.600          3 Report
RESPONSE: ...
;
_vrf_PLAT911_azoIBr_270K
;
PROBLEM: Missing FCF Refl Between Thmin & STh/L=    0.600          3 Report
RESPONSE: ...
;
# end Validation Reply Form
```

It is advisable to attempt to resolve as many as possible of the alerts in all categories. Often the minor alerts point to easily fixed oversights, errors and omissions in your CIF or refinement strategy, so attention to these fine details can be worthwhile. In order to resolve some of the more serious problems it may be necessary to carry out additional measurements or structure refinements. However, the purpose of your study may justify the reported deviations and the more serious of these should normally be commented upon in the discussion or experimental section of a paper or in the "special_details" fields of the CIF. checkCIF was carefully designed to identify outliers and unusual parameters, but every test has its limitations and alerts that are not important in a particular case may appear. Conversely, the absence of alerts does not guarantee there are no aspects of the results needing attention. It is up to the individual to critically assess their own results and, if necessary, seek expert advice.

Publication of your CIF in IUCr journals

A basic structural check has been run on your CIF. These basic checks will be run on all CIFs submitted for publication in IUCr journals (*Acta Crystallographica*, *Journal of Applied Crystallography*, *Journal of Synchrotron Radiation*); however, if you intend to submit to *Acta Crystallographica Section C* or *E* or *IUCrData*, you should make sure that full publication checks are run on the final version of your CIF prior to submission.

Publication of your CIF in other journals

Please refer to the *Notes for Authors* of the relevant journal for any special instructions relating to CIF submission.

PLATON version of 03/06/2021; check.def file version of 02/06/2021

Datablock azoBr_b_190K - ellipsoid plot

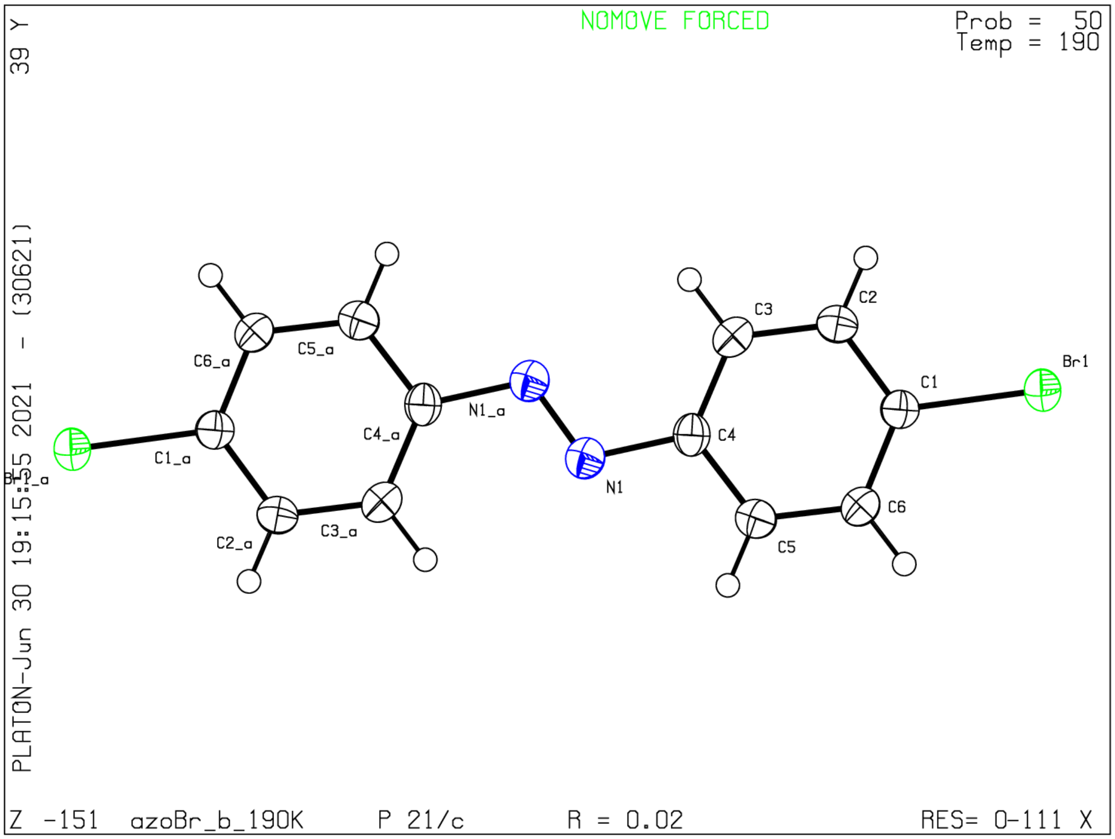

Datablock azoBr_b_210K - ellipsoid plot

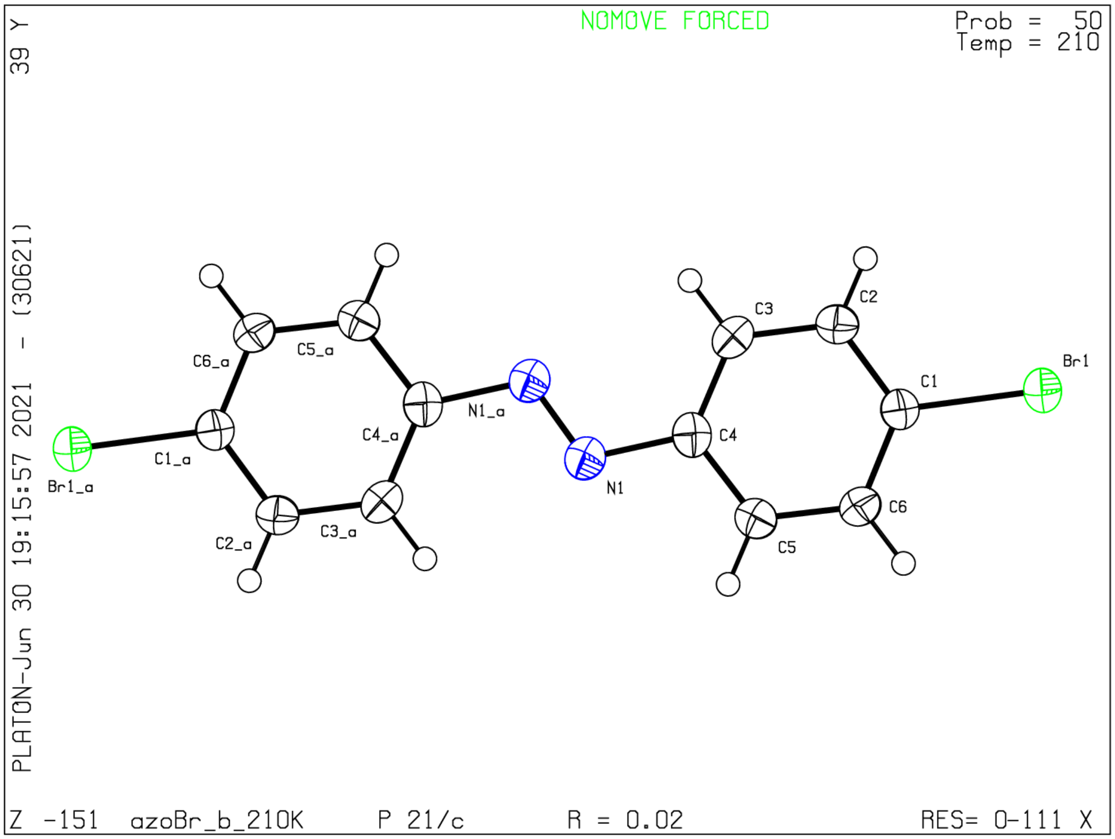

Datablock azoBr_b_230K - ellipsoid plot

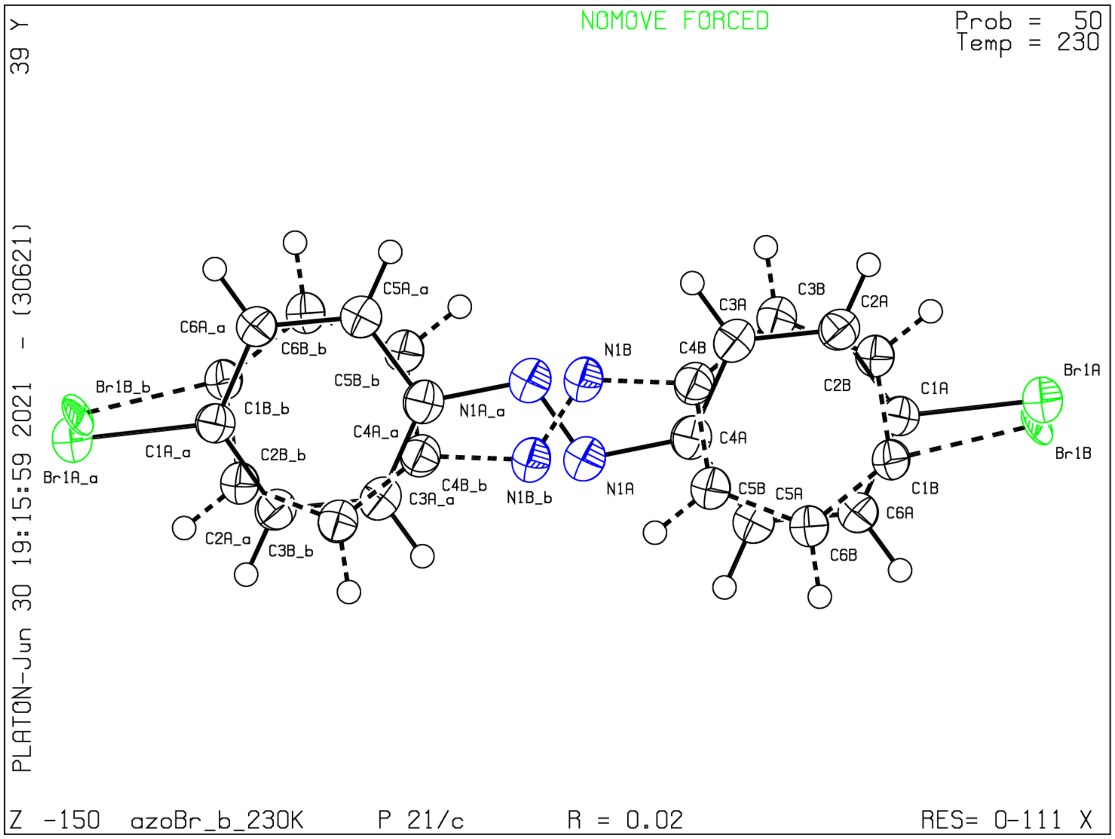

Datablock azoBr_b_250K - ellipsoid plot

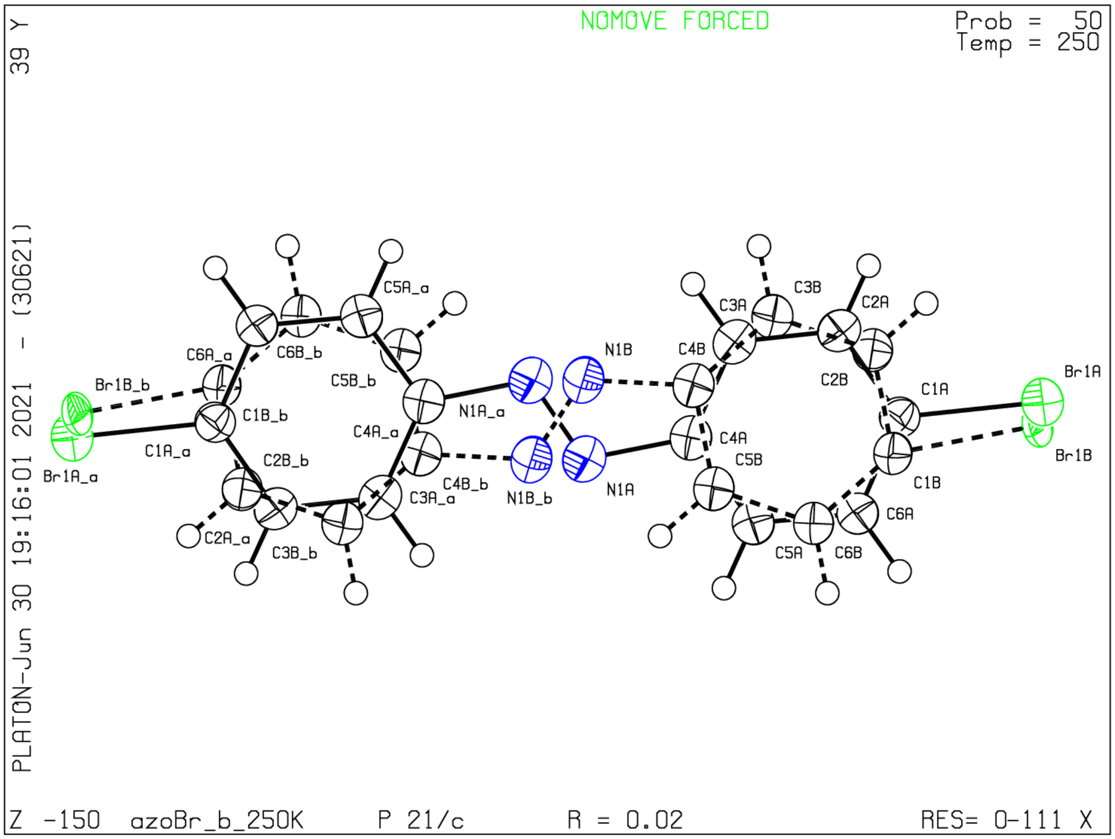

Datablock azoBr_b_270K - ellipsoid plot

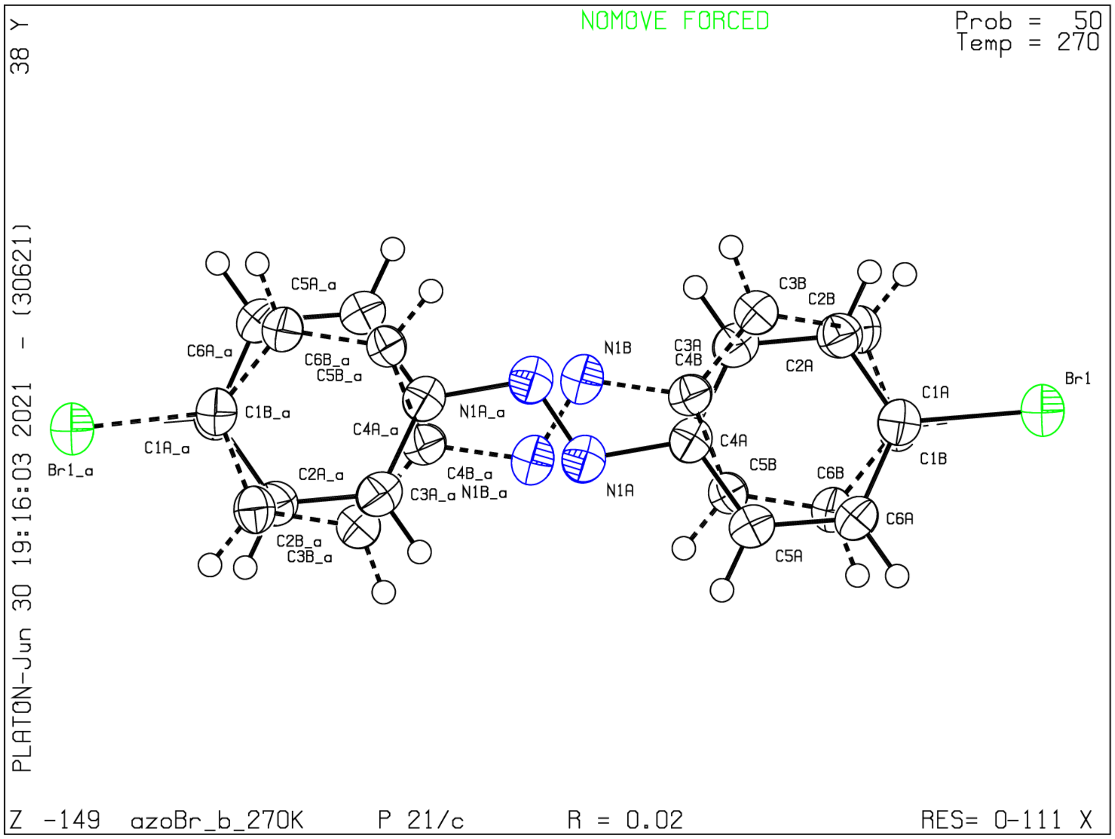

Datablock azoBr_b_290K - ellipsoid plot

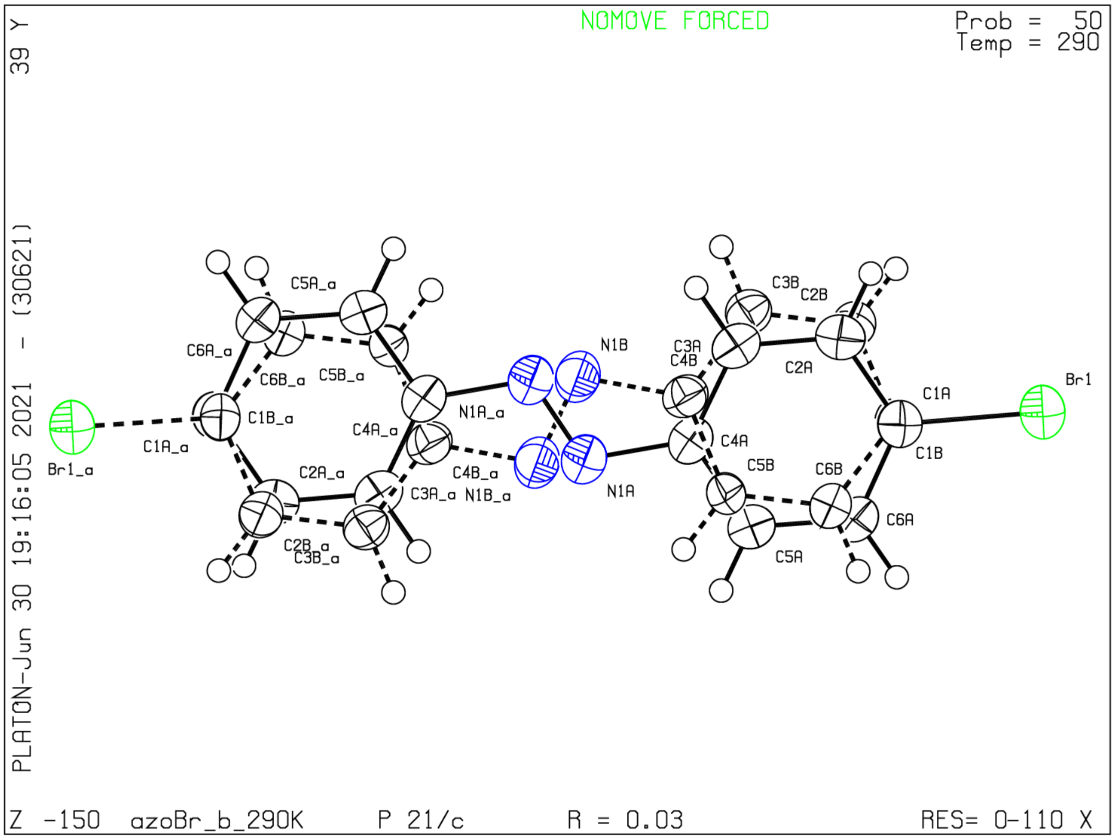

Datablock azoIBr_190K - ellipsoid plot

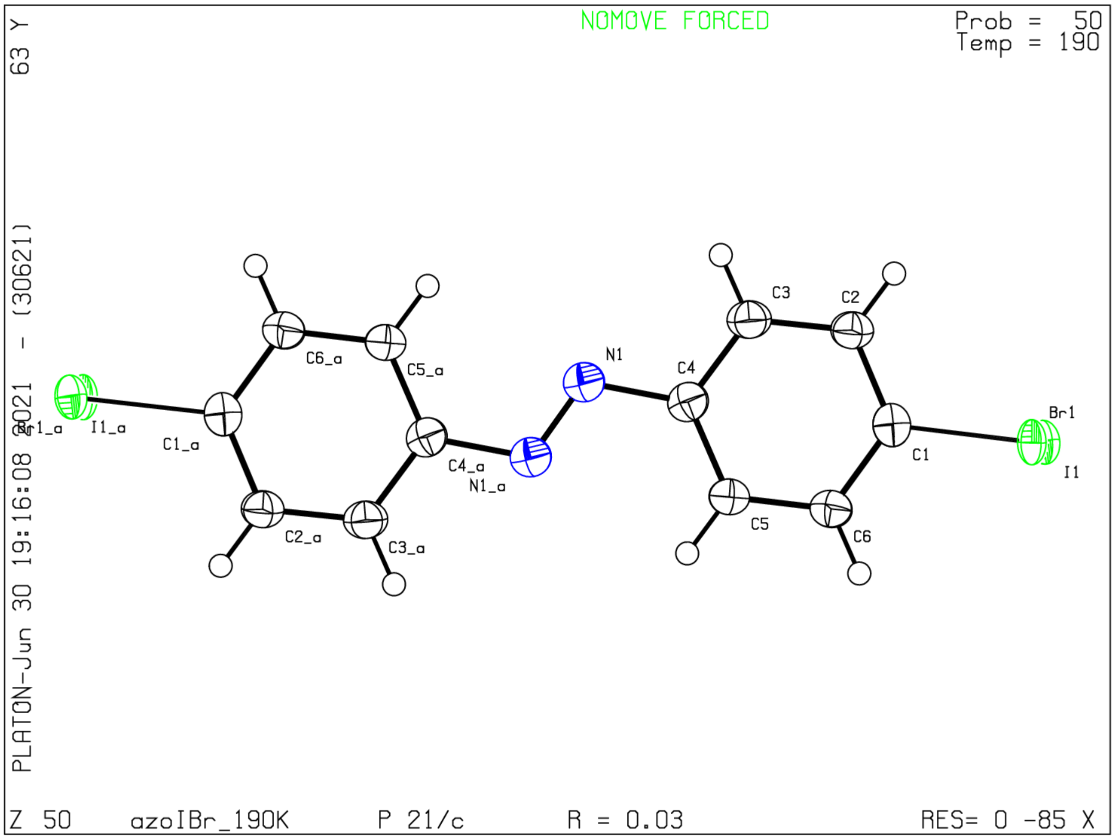

Datablock azoIBr_210K - ellipsoid plot

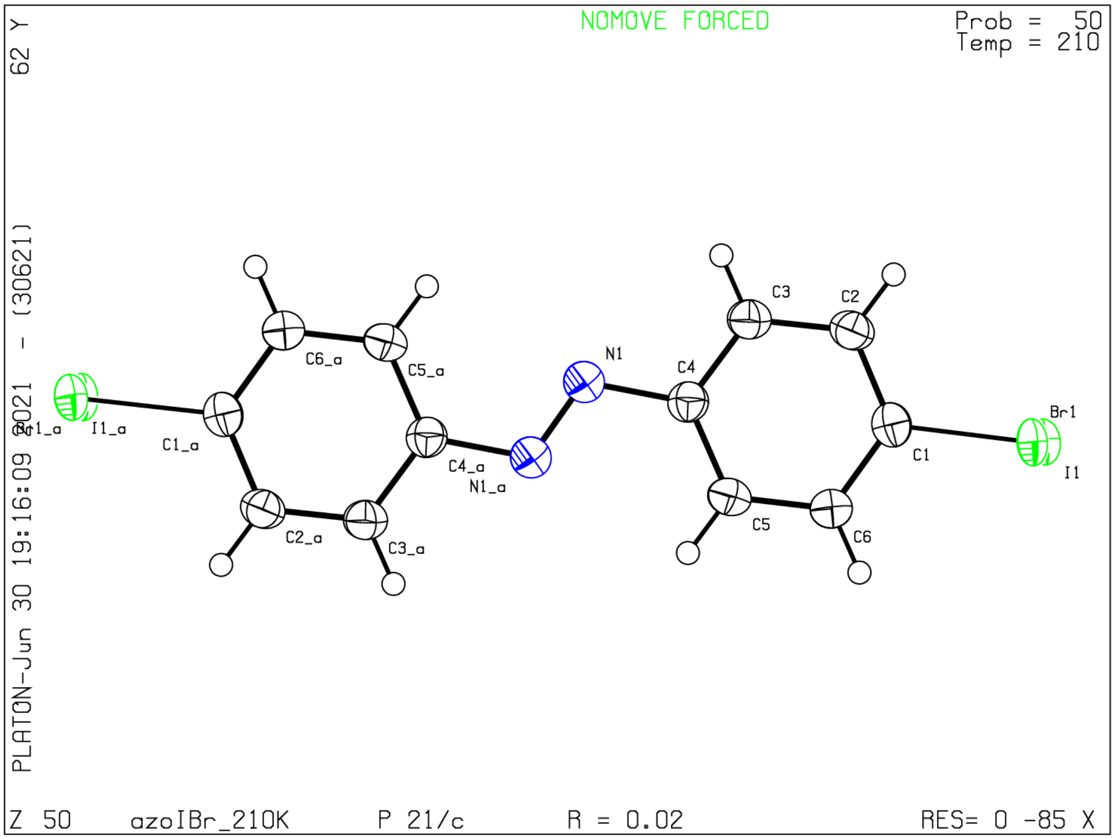

Datablock azoIBr_230K - ellipsoid plot

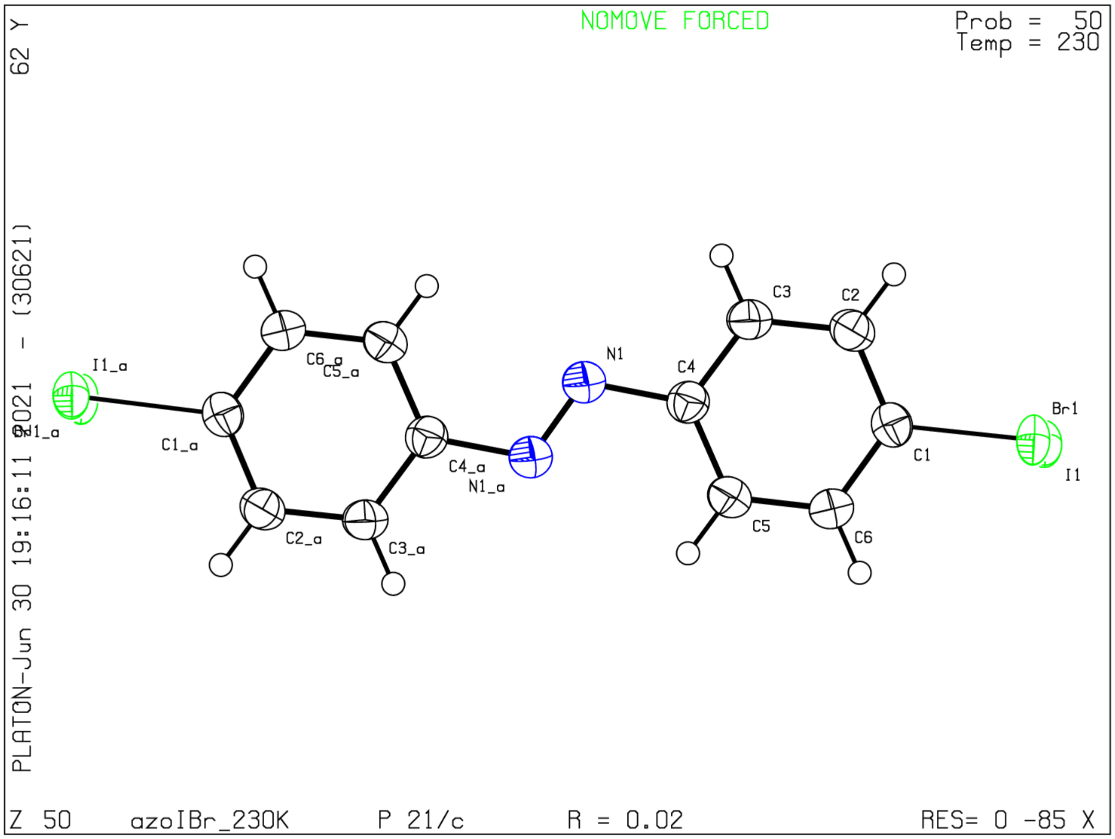

Datablock azoIBr_250K - ellipsoid plot

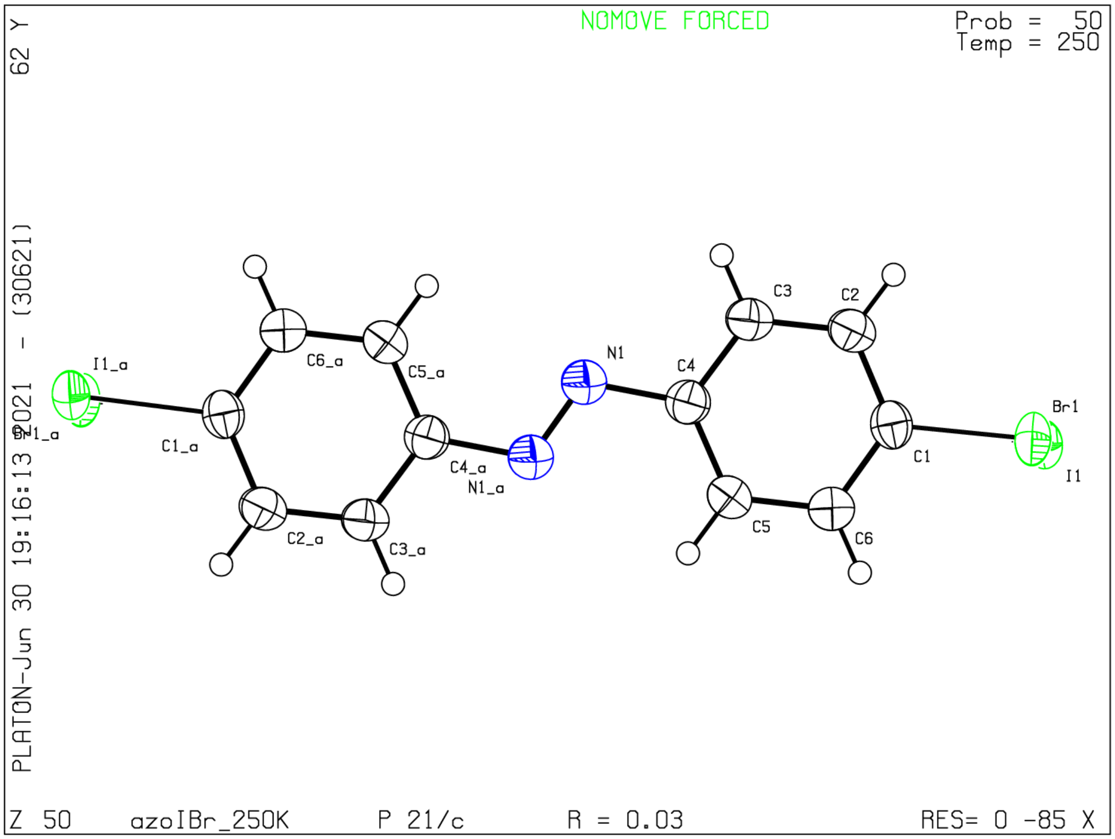

Datablock azoIBr_270K - ellipsoid plot

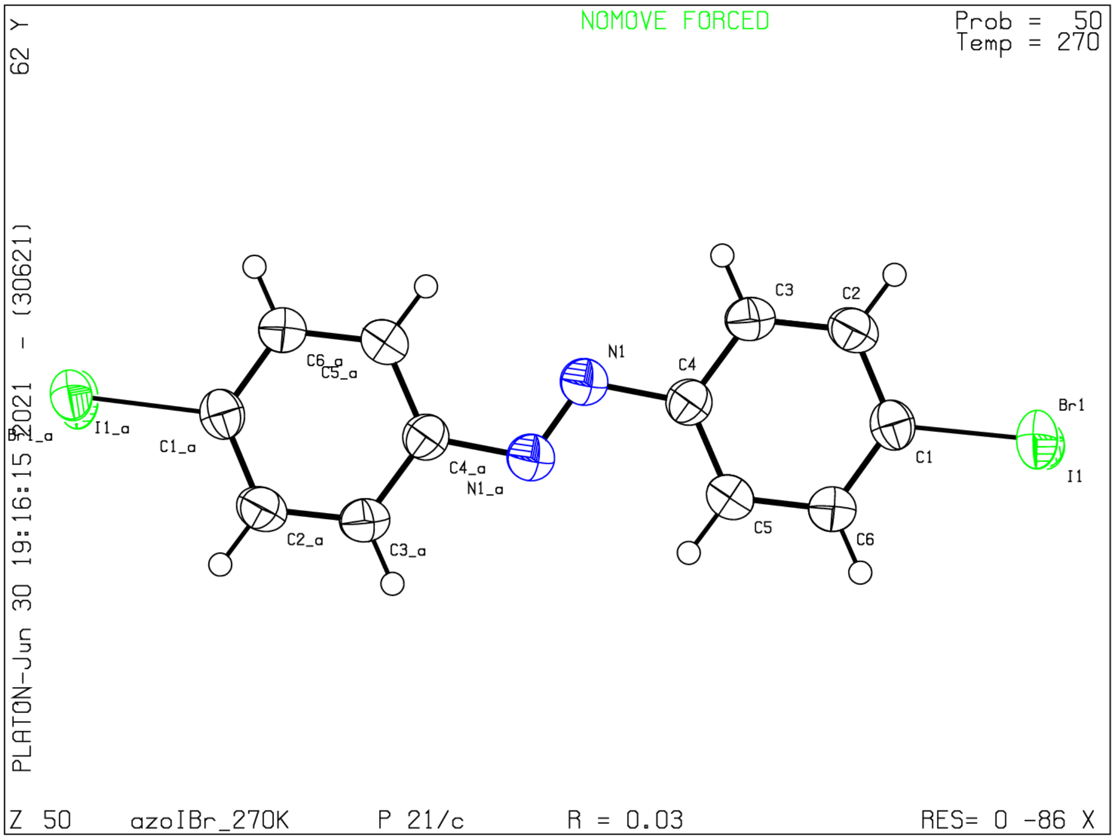

Datablock azoIBr_290K - ellipsoid plot

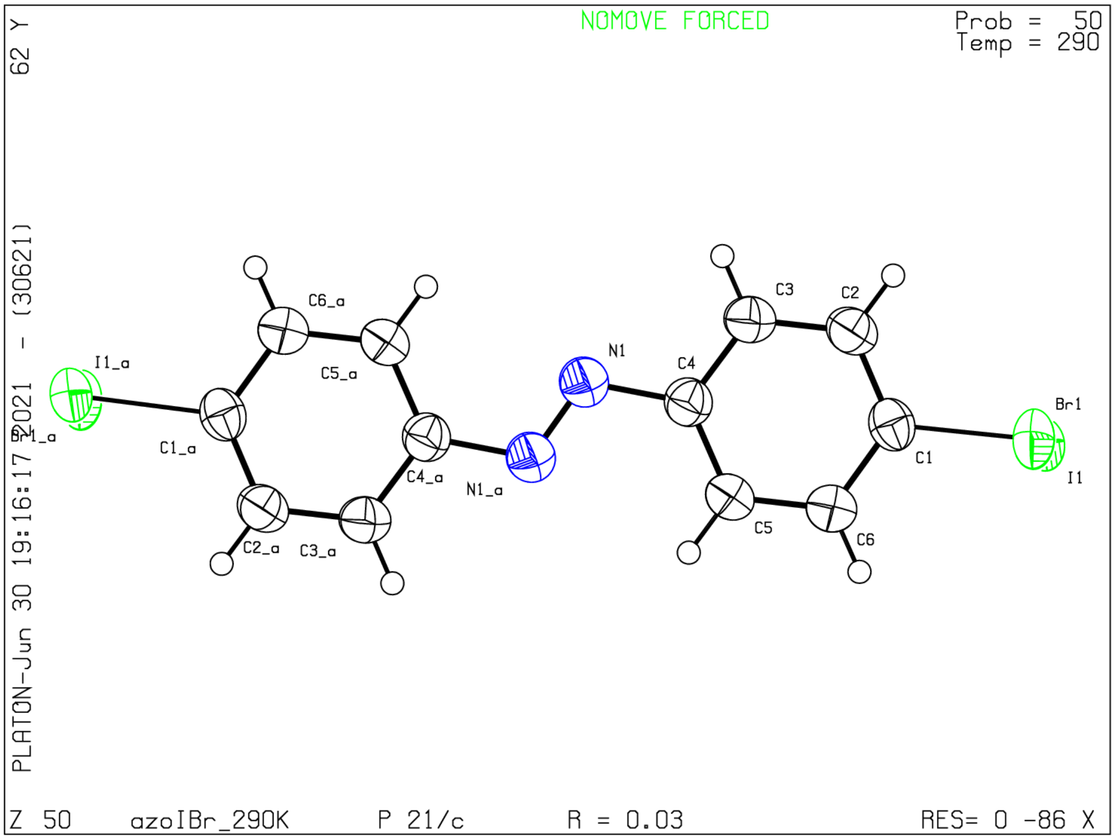

Supplement: Supplementary file 3 [file m-09-00031-sup3.zip › azo-Br b + azo-I Br.pdf]
