# Supplementary material for: Differences in thermal expansion and motion ability for herringbone and face-to-face π-stacked solids
Source: IUCrJ. 2021 Nov 3;9(Pt 1):31–42. doi: 10.1107/S2052252521009593 (PMC8733877; doi:10.1107/S2052252521009593)

checkCIF/PLATON report

Structure factors have been supplied for datablock(s) diolefinBr_190K, diolefinBr_210K, diolefinBr_230K, diolefinBr_250K, diolefinBr_270K, diolefinBr_290K, diolefinI_190K, diolefinI_210K, diolefinI_230K, diolefinI_250K, diolefinI_270K, diolefinI_290K

THIS REPORT IS FOR GUIDANCE ONLY. IF USED AS PART OF A REVIEW PROCEDURE FOR PUBLICATION, IT SHOULD NOT REPLACE THE EXPERTISE OF AN EXPERIENCED CRYSTALLOGRAPHIC REFEREE.

No syntax errors found. CIF dictionary Interpreting this report

Datablock: diolefinI_190K

```
Bond precision:  C-C = 0.0041 A          Wavelength=1.54178

Cell:            a=7.39117(10)     b=40.8276(4)     c=5.94846(5)
                 alpha=90          beta=90          gamma=90
Temperature:     190 K

                Calculated                Reported
Volume          1795.03(3)                1795.03(3)
Space group     P c c n                   P c c n
Hall group      -P 2ab 2ac                -P 2ab 2ac
Moiety formula  C22 H16 I2                ?
Sum formula     C22 H16 I2                C22 H16 I2
Mr              534.15                    534.15
Dx,g cm-3       1.977                     1.977
Z               4                         4
Mu (mm-1)       27.496                    27.496
F000            1016.0                    1016.0
F000'           1016.43
h,k,lmax        9,51,7                    9,51,7
Nref            1904                      1875
Tmin,Tmax       0.115,0.561               0.436,1.000
Tmin'           0.013

Correction method= # Reported T Limits: Tmin=0.436 Tmax=1.000
AbsCorr = GAUSSIAN

Data completeness= 0.985          Theta(max)= 77.308

R(reflections)= 0.0250( 1765)     wR2(reflections)= 0.0684( 1875)

S = 1.084                   Npar= 109
```

The following ALERTS were generated. Each ALERT has the format
test-name_ALERT_alert-type_alert-level.
Click on the hyperlinks for more details of the test.

Alert level G

```
PLAT142_ALERT_4_G s.u. on b - Axis Small or Missing .............    0.00040 Ang.
PLAT143_ALERT_4_G s.u. on c - Axis Small or Missing .............    0.00005 Ang.
PLAT912_ALERT_4_G Missing # of FCF Reflections Above STh/L=  0.600        29 Note
PLAT978_ALERT_2_G Number C-C Bonds with Positive Residual Density.         5 Info
```

---

0 **ALERT level A** = Most likely a serious problem - resolve or explain
0 **ALERT level B** = A potentially serious problem, consider carefully
0 **ALERT level C** = Check. Ensure it is not caused by an omission or oversight
4 **ALERT level G** = General information/check it is not something unexpected

0 ALERT type 1 CIF construction/syntax error, inconsistent or missing data
1 ALERT type 2 Indicator that the structure model may be wrong or deficient
0 ALERT type 3 Indicator that the structure quality may be low
3 ALERT type 4 Improvement, methodology, query or suggestion
0 ALERT type 5 Informative message, check

Datablock: diolefinI_210K

Bond precision: C-C = 0.0040 A Wavelength=1.54178

Cell:	a=7.40722(9)	b=40.8177(3)	c=5.95673(5)
	alpha=90	beta=90	gamma=90
Temperature:	210 K		

	Calculated	Reported
Volume	1800.99(3)	1800.99(3)
Space group	P c c n	P c c n
Hall group	-P 2ab 2ac	-P 2ab 2ac
Moiety formula	C22 H16 I2	?
Sum formula	C22 H16 I2	C22 H16 I2
Mr	534.15	534.15
Dx,g cm-3	1.970	1.970
Z	4	4
Mu (mm-1)	27.405	27.405
F000	1016.0	1016.0
F000’	1016.43	
h,k,lmax	9,51,7	9,51,7
Nref	1912	1881
Tmin,Tmax	0.116,0.562	0.436,1.000
Tmin’	0.013	

Correction method= # Reported T Limits: Tmin=0.436 Tmax=1.000
AbsCorr = GAUSSIAN

```
Data completeness= 0.984          Theta(max)= 77.398

R(reflections)= 0.0242( 1762)     wR2(reflections)= 0.0645( 1881)

S = 0.997                   Npar= 109
```

The following ALERTS were generated. Each ALERT has the format
test-name_ALERT_alert-type_alert-level.
Click on the hyperlinks for more details of the test.

Alert level G

```
PLAT142_ALERT_4_G s.u. on b - Axis Small or Missing .............    0.00030 Ang.
PLAT143_ALERT_4_G s.u. on c - Axis Small or Missing .............    0.00005 Ang.
PLAT912_ALERT_4_G Missing # of FCF Reflections Above STh/L=  0.600        31 Note
PLAT978_ALERT_2_G Number C-C Bonds with Positive Residual Density.         4 Info
```

---

0 **ALERT level A** = Most likely a serious problem - resolve or explain
0 **ALERT level B** = A potentially serious problem, consider carefully
0 **ALERT level C** = Check. Ensure it is not caused by an omission or oversight
4 **ALERT level G** = General information/check it is not something unexpected

0 ALERT type 1 CIF construction/syntax error, inconsistent or missing data
1 ALERT type 2 Indicator that the structure model may be wrong or deficient
0 ALERT type 3 Indicator that the structure quality may be low
3 ALERT type 4 Improvement, methodology, query or suggestion
0 ALERT type 5 Informative message, check

Datablock: diolefinI_230K

```
Bond precision:  C-C = 0.0041 A          Wavelength=1.54178

Cell:            a=7.42486(9)      b=40.8040(3)      c=5.96406(5)
                 alpha=90          beta=90           gamma=90
Temperature:     230 K
```

	Calculated	Reported
Volume	1806.90(3)	1806.90(3)
Space group	P c c n	P c c n
Hall group	-P 2ab 2ac	-P 2ab 2ac
Moiety formula	C22 H16 I2	?
Sum formula	C22 H16 I2	C22 H16 I2
Mr	534.15	534.15
Dx,g cm-3	1.964	1.964
Z	4	4
Mu (mm-1)	27.315	27.315
F000	1016.0	1016.0
F000’	1016.43	
h,k,lmax	9,51,7	9,51,7
Nref	1919	1891
Tmin,Tmax	0.117,0.563	0.436,1.000
Tmin’	0.013	

Correction method= # Reported T Limits: Tmin=0.436 Tmax=1.000
AbsCorr = GAUSSIAN

Data completeness= 0.985   Theta(max)= 77.384

R(reflections)= 0.0261(1758)   wR2(reflections)= 0.0712(1891)

S = 1.049   Npar= 109

The following ALERTS were generated. Each ALERT has the format
test-name_ALERT_alert-type_alert-level.
Click on the hyperlinks for more details of the test.

Alert level G

```
PLAT142_ALERT_4_G s.u. on b - Axis Small or Missing .............    0.00030 Ang.
PLAT143_ALERT_4_G s.u. on c - Axis Small or Missing .............    0.00005 Ang.
PLAT912_ALERT_4_G Missing # of FCF Reflections Above STh/L=  0.600        29 Note
PLAT978_ALERT_2_G Number C-C Bonds with Positive Residual Density.         4 Info
```

---

0 **ALERT level A** = Most likely a serious problem - resolve or explain
0 **ALERT level B** = A potentially serious problem, consider carefully
0 **ALERT level C** = Check. Ensure it is not caused by an omission or oversight
4 **ALERT level G** = General information/check it is not something unexpected

0 ALERT type 1 CIF construction/syntax error, inconsistent or missing data
1 ALERT type 2 Indicator that the structure model may be wrong or deficient
0 ALERT type 3 Indicator that the structure quality may be low
3 ALERT type 4 Improvement, methodology, query or suggestion
0 ALERT type 5 Informative message, check

Datablock: diolefinI_250K

Bond precision: C-C = 0.0055 A Wavelength=1.54178

Cell:	a=7.4616(6)	b=40.8296(16)	c=5.9716(3)
	alpha=90	beta=90	gamma=90
Temperature:	250 K		

	Calculated	Reported
Volume	1819.27(19)	1819.3(2)
Space group	P c c n	P c c n
Hall group	-P 2ab 2ac	-P 2ab 2ac
Moiety formula	C22 H16 I2	?
Sum formula	C22 H16 I2	C22 H16 I2
Mr	534.15	534.15
Dx,g cm-3	1.950	1.950
Z	4	4
Mu (mm-1)	27.129	27.129
F000	1016.0	1016.0
F000’	1016.43	
h,k,lmax	9,51,7	9,51,7
Nref	1928	1884
Tmin,Tmax	0.119,0.566	0.469,1.000
Tmin’	0.014	

Correction method= # Reported T Limits: Tmin=0.469 Tmax=1.000
AbsCorr = GAUSSIAN

Data completeness= 0.977 Theta(max)= 77.149

R(reflections)= 0.0311(1684) wR2(reflections)= 0.0861(1884)

S = 1.091 Npar= 109

The following ALERTS were generated. Each ALERT has the format
test-name_ALERT_alert-type_alert-level.
Click on the hyperlinks for more details of the test.

Alert level C

PLAT911_ALERT_3_C Missing FCF Refl Between Thmin & STh/L= 0.600 2 Report

---

Alert level G

PLAT910_ALERT_3_G Missing # of FCF Reflection(s) Below Theta(Min). 1 Note
PLAT912_ALERT_4_G Missing # of FCF Reflections Above STh/L= 0.600 42 Note
PLAT913_ALERT_3_G Missing # of Very Strong Reflections in FCF .... 1 Note
PLAT978_ALERT_2_G Number C-C Bonds with Positive Residual Density. 4 Info

---

0 **ALERT level A** = Most likely a serious problem - resolve or explain
0 **ALERT level B** = A potentially serious problem, consider carefully
1 **ALERT level C** = Check. Ensure it is not caused by an omission or oversight
4 **ALERT level G** = General information/check it is not something unexpected

```
   0 ALERT type 1 CIF construction/syntax error, inconsistent or missing data
   1 ALERT type 2 Indicator that the structure model may be wrong or deficient
   3 ALERT type 3 Indicator that the structure quality may be low
   1 ALERT type 4 Improvement, methodology, query or suggestion
   0 ALERT type 5 Informative message, check
```

Datablock: diolefinI_270K

```
Bond precision:  C-C = 0.0050 A         Wavelength=1.54178

Cell:            a=7.46583(10)     b=40.7716(3)     c=5.98213(5)
                 alpha=90          beta=90          gamma=90
Temperature:     270 K

                Calculated                Reported
Volume          1820.92(3)                1820.92(3)
Space group     P c c n                   P c c n
Hall group      -P 2ab 2ac                -P 2ab 2ac
Moiety formula  C22 H16 I2                ?
Sum formula     C22 H16 I2                C22 H16 I2
Mr              534.15                    534.15
Dx,g cm-3       1.948                     1.948
Z               4                         4
Mu (mm-1)       27.105                    27.105
F000            1016.0                    1016.0
F000'           1016.43
h,k,lmax        9,51,7                    9,50,7
Nref            1935                      1894
Tmin,Tmax       0.120,0.566               0.446,1.000
Tmin'           0.014

Correction method= # Reported T Limits: Tmin=0.446 Tmax=1.000
AbsCorr = GAUSSIAN

Data completeness= 0.979          Theta(max)= 77.356

R(reflections)= 0.0294( 1719)     wR2(reflections)= 0.0826( 1894)

S = 1.107                  Npar= 109
```

The following ALERTS were generated. Each ALERT has the format
test-name_ALERT_alert-type_alert-level.
Click on the hyperlinks for more details of the test.

Alert level C

```
PLAT911_ALERT_3_C Missing FCF Refl Between Thmin & STh/L=    0.600          3 Report
```

Alert level G

```
PLAT142_ALERT_4_G s.u. on b - Axis Small or Missing .............    0.00030 Ang.
PLAT143_ALERT_4_G s.u. on c - Axis Small or Missing .............    0.00005 Ang.
PLAT912_ALERT_4_G Missing # of FCF Reflections Above STh/L=  0.600        38 Note
PLAT913_ALERT_3_G Missing # of Very Strong Reflections in FCF ....         1 Note
PLAT978_ALERT_2_G Number C-C Bonds with Positive Residual Density.         7 Info
```

0 **ALERT level A** = Most likely a serious problem - resolve or explain
0 **ALERT level B** = A potentially serious problem, consider carefully
1 **ALERT level C** = Check. Ensure it is not caused by an omission or oversight
5 **ALERT level G** = General information/check it is not something unexpected

0 ALERT type 1 CIF construction/syntax error, inconsistent or missing data
1 ALERT type 2 Indicator that the structure model may be wrong or deficient
2 ALERT type 3 Indicator that the structure quality may be low
3 ALERT type 4 Improvement, methodology, query or suggestion
0 ALERT type 5 Informative message, check

Datablock: diolefinI_290K

Bond precision: C-C = 0.0059 A   Wavelength=1.54178

Cell:	a=7.48873(11)	b=40.7467(3)	c=5.99117(6)
	alpha=90	beta=90	gamma=90
Temperature:	290 K		

	Calculated	Reported
Volume	1828.15(4)	1828.15(4)
Space group	P c c n	P c c n
Hall group	-P 2ab 2ac	-P 2ab 2ac
Moiety formula	C22 H16 I2	?
Sum formula	C22 H16 I2	C22 H16 I2
Mr	534.15	534.15
Dx,g cm-3	1.941	1.941
Z	4	4
Mu (mm-1)	26.997	26.997
F000	1016.0	1016.0
F000’	1016.43	
h,k,lmax	9,51,7	9,50,7
Nref	1947	1903
Tmin,Tmax	0.121,0.567	0.445,1.000
Tmin’	0.014	

Correction method= # Reported T Limits: Tmin=0.445 Tmax=1.000
AbsCorr = GAUSSIAN

Data completeness= 0.977   Theta(max)= 77.388

```
R(reflections)= 0.0324( 1683)     wR2(reflections)= 0.0952( 1903)

S = 1.045                   Npar= 109
```

```
The following ALERTS were generated. Each ALERT has the format
       test-name_ALERT_alert-type_alert-level.
Click on the hyperlinks for more details of the test.
```

Alert level C

```
PLAT911_ALERT_3_C Missing FCF Refl Between Thmin & STh/L=    0.600          3 Report
```

Alert level G

```
PLAT142_ALERT_4_G s.u. on b - Axis Small or Missing ..............    0.00030 Ang.
PLAT912_ALERT_4_G Missing # of FCF Reflections Above STh/L=  0.600         41 Note
PLAT913_ALERT_3_G Missing # of Very Strong Reflections in FCF ....          1 Note
PLAT978_ALERT_2_G Number C-C Bonds with Positive Residual Density.          4 Info
```

```
   0 ALERT level A = Most likely a serious problem - resolve or explain
   0 ALERT level B = A potentially serious problem, consider carefully
   1 ALERT level C = Check. Ensure it is not caused by an omission or oversight
   4 ALERT level G = General information/check it is not something unexpected

   0 ALERT type 1 CIF construction/syntax error, inconsistent or missing data
   1 ALERT type 2 Indicator that the structure model may be wrong or deficient
   2 ALERT type 3 Indicator that the structure quality may be low
   2 ALERT type 4 Improvement, methodology, query or suggestion
   0 ALERT type 5 Informative message, check
```

Datablock: diolefinBr_190K

```
Bond precision:   C-C = 0.0020 A           Wavelength=1.54178

Cell:             a=7.36426(6)      b=39.5134(3)      c=5.89107(4)
                  alpha=90          beta=90           gamma=90
Temperature:      190 K
```

	Calculated	Reported
Volume	1714.23(2)	1714.22(2)
Space group	P c c n	P c c n
Hall group	-P 2ab 2ac	-P 2ab 2ac
Moiety formula	C22 H16 Br2	?
Sum formula	C22 H16 Br2	C22 H16 Br2
Mr	440.15	440.17
Dx,g cm-3	1.706	1.706
Z	4	4
Mu (mm-1)	5.971	5.971
F000	872.0	872.0
F000’	868.12	
h,k,lmax	9,50,7	9,50,7
Nref	1819	1816
Tmin,Tmax	0.463,0.742	0.648,1.000
Tmin’	0.339	

Correction method= # Reported T Limits: Tmin=0.648 Tmax=1.000
AbsCorr = GAUSSIAN

Data completeness= 0.998 Theta(max)= 77.286

R(reflections)= 0.0201(1736) wR2(reflections)= 0.0560(1816)

S = 1.081 Npar= 110

The following ALERTS were generated. Each ALERT has the format
test-name_ALERT_alert-type_alert-level.
Click on the hyperlinks for more details of the test.

Alert level G

```
PLAT142_ALERT_4_G s.u. on b - Axis Small or Missing .............    0.00030 Ang.
PLAT143_ALERT_4_G s.u. on c - Axis Small or Missing .............    0.00004 Ang.
PLAT912_ALERT_4_G Missing # of FCF Reflections Above STh/L=  0.600         3 Note
PLAT978_ALERT_2_G Number C-C Bonds with Positive Residual Density.        14 Info
```

---

0 **ALERT level A** = Most likely a serious problem - resolve or explain
0 **ALERT level B** = A potentially serious problem, consider carefully
0 **ALERT level C** = Check. Ensure it is not caused by an omission or oversight
4 **ALERT level G** = General information/check it is not something unexpected

0 ALERT type 1 CIF construction/syntax error, inconsistent or missing data
1 ALERT type 2 Indicator that the structure model may be wrong or deficient
0 ALERT type 3 Indicator that the structure quality may be low
3 ALERT type 4 Improvement, methodology, query or suggestion
0 ALERT type 5 Informative message, check

Datablock: diolefinBr_210K

Bond precision:	C-C = 0.0020 A		Wavelength=1.54178
Cell:	a=7.37918(6)	b=39.5129(3)	c=5.89885(4)
	alpha=90	beta=90	gamma=90
Temperature:	210 K		

	Calculated	Reported
Volume	1719.94(2)	1719.95(2)
Space group	P c c n	P c c n
Hall group	-P 2ab 2ac	-P 2ab 2ac
Moiety formula	C22 H16 Br2	?
Sum formula	C22 H16 Br2	C22 H16 Br2
Mr	440.15	440.17
Dx,g cm-3	1.700	1.700
Z	4	4
Mu (mm-1)	5.951	5.951
F000	872.0	872.0
F000’	868.12	
h,k,lmax	9,50,7	9,50,7
Nref	1823	1817
Tmin,Tmax	0.464,0.743	0.647,1.000
Tmin’	0.340	

Correction method= # Reported T Limits: Tmin=0.647 Tmax=1.000
AbsCorr = GAUSSIAN

Data completeness= 0.997 Theta(max)= 77.289

R(reflections)= 0.0201(1713) wR2(reflections)= 0.0578(1817)

S = 1.083 Npar= 110

The following ALERTS were generated. Each ALERT has the format
test-name_ALERT_alert-type_alert-level.
Click on the hyperlinks for more details of the test.

Alert level G

PLAT142_ALERT_4_G	s.u. on b - Axis Small or Missing	0.00030 Ang.
PLAT143_ALERT_4_G	s.u. on c - Axis Small or Missing	0.00004 Ang.
PLAT912_ALERT_4_G	Missing # of FCF Reflections Above STh/L= 0.600	5 Note
PLAT913_ALERT_3_G	Missing # of Very Strong Reflections in FCF	1 Note
PLAT978_ALERT_2_G	Number C-C Bonds with Positive Residual Density.	12 Info

---

0 **ALERT level A** = Most likely a serious problem - resolve or explain
0 **ALERT level B** = A potentially serious problem, consider carefully
0 **ALERT level C** = Check. Ensure it is not caused by an omission or oversight
5 **ALERT level G** = General information/check it is not something unexpected

0 ALERT type 1 CIF construction/syntax error, inconsistent or missing data

```
   1 ALERT type 2 Indicator that the structure model may be wrong or deficient
   1 ALERT type 3 Indicator that the structure quality may be low
   3 ALERT type 4 Improvement, methodology, query or suggestion
   0 ALERT type 5 Informative message, check
```

Datablock: diolefinBr_230K

```
Bond precision:   C-C = 0.0020 A          Wavelength=1.54178

Cell:             a=7.39509(6)      b=39.5130(3)      c=5.90727(4)
                  alpha=90          beta=90           gamma=90
Temperature:      230 K
```

	Calculated	Reported
Volume	1726.12(2)	1726.12(2)
Space group	P c c n	P c c n
Hall group	-P 2ab 2ac	-P 2ab 2ac
Moiety formula	C22 H16 Br2	?
Sum formula	C22 H16 Br2	C22 H16 Br2
Mr	440.15	440.17
Dx,g cm-3	1.694	1.694
Z	4	4
Mu (mm-1)	5.930	5.930
F000	872.0	872.0
F000’	868.12	
h,k,lmax	9,50,7	9,50,7
Nref	1835	1825
Tmin,Tmax	0.466,0.743	0.647,1.000
Tmin’	0.341	

```
Correction method= # Reported T Limits: Tmin=0.647 Tmax=1.000
AbsCorr = GAUSSIAN

Data completeness= 0.995          Theta(max)= 77.449

R(reflections)= 0.0208( 1702)     wR2(reflections)= 0.0611( 1825)

S = 1.092                    Npar= 110
```

The following ALERTS were generated. Each ALERT has the format
test-name_ALERT_alert-type_alert-level.
Click on the hyperlinks for more details of the test.

Alert level C

```
PLAT911_ALERT_3_C Missing FCF Refl Between Thmin & STh/L=    0.600          2 Report
```

Alert level G

```
PLAT142_ALERT_4_G s.u. on b - Axis Small or Missing .............    0.00030 Ang.
PLAT143_ALERT_4_G s.u. on c - Axis Small or Missing .............    0.00004 Ang.
PLAT912_ALERT_4_G Missing # of FCF Reflections Above STh/L=  0.600         8 Note
PLAT913_ALERT_3_G Missing # of Very Strong Reflections in FCF ....         1 Note
PLAT933_ALERT_2_G Number of OMIT Records in Embedded .res File ...         2 Note
PLAT978_ALERT_2_G Number C-C Bonds with Positive Residual Density.         9 Info
```

```
   0 ALERT level A = Most likely a serious problem - resolve or explain
   0 ALERT level B = A potentially serious problem, consider carefully
   1 ALERT level C = Check. Ensure it is not caused by an omission or oversight
   6 ALERT level G = General information/check it is not something unexpected

   0 ALERT type 1 CIF construction/syntax error, inconsistent or missing data
   2 ALERT type 2 Indicator that the structure model may be wrong or deficient
   2 ALERT type 3 Indicator that the structure quality may be low
   3 ALERT type 4 Improvement, methodology, query or suggestion
   0 ALERT type 5 Informative message, check
```

Datablock: diolefinBr_250K

```
Bond precision:  C-C = 0.0020 A          Wavelength=1.54178

Cell:            a=7.41155(7)     b=39.5112(3)     c=5.91607(5)
                 alpha=90         beta=90          gamma=90
Temperature:     250 K
```

	Calculated	Reported
Volume	1732.46(3)	1732.46(3)
Space group	P c c n	P c c n
Hall group	-P 2ab 2ac	-P 2ab 2ac
Moiety formula	C22 H16 Br2	?
Sum formula	C22 H16 Br2	C22 H16 Br2
Mr	440.15	440.17
Dx,g cm-3	1.688	1.688
Z	4	4
Mu (mm-1)	5.908	5.908
F000	872.0	872.0
F000’	868.12	
h,k,lmax	9,50,7	9,50,7
Nref	1837	1829
Tmin,Tmax	0.467,0.744	0.647,1.000
Tmin’	0.342	

```
Correction method= # Reported T Limits: Tmin=0.647 Tmax=1.000
AbsCorr = GAUSSIAN

Data completeness= 0.996          Theta(max)= 77.356
```

R(reflections)= 0.0218(1697) wR2(reflections)= 0.0617(1829)

S = 1.078 Npar= 110

The following ALERTS were generated. Each ALERT has the format
test-name_ALERT_alert-type_alert-level.
Click on the hyperlinks for more details of the test.

Alert level C

PLAT911_ALERT_3_C Missing FCF Refl Between Thmin & STh/L= 0.600 3 Report

---

Alert level G

PLAT142_ALERT_4_G s.u. on b - Axis Small or Missing .... 0.00030 Ang.
PLAT143_ALERT_4_G s.u. on c - Axis Small or Missing .... 0.00005 Ang.
PLAT912_ALERT_4_G Missing # of FCF Reflections Above STh/L= 0.600 5 Note
PLAT913_ALERT_3_G Missing # of Very Strong Reflections in FCF .... 1 Note
PLAT933_ALERT_2_G Number of OMIT Records in Embedded .res File ... 3 Note
PLAT978_ALERT_2_G Number C-C Bonds with Positive Residual Density. 8 Info

---

0 **ALERT level A** = Most likely a serious problem - resolve or explain
0 **ALERT level B** = A potentially serious problem, consider carefully
1 **ALERT level C** = Check. Ensure it is not caused by an omission or oversight
6 **ALERT level G** = General information/check it is not something unexpected

0 ALERT type 1 CIF construction/syntax error, inconsistent or missing data
2 ALERT type 2 Indicator that the structure model may be wrong or deficient
2 ALERT type 3 Indicator that the structure quality may be low
3 ALERT type 4 Improvement, methodology, query or suggestion
0 ALERT type 5 Informative message, check

Datablock: diolefinBr_270K

Bond precision: C-C = 0.0023 A Wavelength=1.54178

Cell:	a=7.42903(7)	b=39.5057(3)	c=5.92607(5)
	alpha=90	beta=90	gamma=90
Temperature:	270 K		

	Calculated	Reported
Volume	1739.24(3)	1739.23(3)
Space group	P c c n	P c c n
Hall group	-P 2ab 2ac	-P 2ab 2ac
Moiety formula	C22 H16 Br2	?
Sum formula	C22 H16 Br2	C22 H16 Br2
Mr	440.15	440.17
Dx,g cm-3	1.681	1.681
Z	4	4
Mu (mm-1)	5.885	5.885
F000	872.0	872.0
F000’	868.12	
h,k,lmax	9,50,7	9,50,7
Nref	1848	1840
Tmin,Tmax	0.428,0.745	0.648,1.000
Tmin’	0.376	

Correction method= # Reported T Limits: Tmin=0.648 Tmax=1.000
AbsCorr = GAUSSIAN

Data completeness= 0.996 Theta(max)= 77.370

R(reflections)= 0.0238(1680) wR2(reflections)= 0.0659(1840)

S = 1.065 Npar= 130

The following ALERTS were generated. Each ALERT has the format
test-name_ALERT_alert-type_alert-level.
Click on the hyperlinks for more details of the test.

Alert level C

```
PLAT911_ALERT_3_C Missing FCF Refl Between Thmin & STh/L=    0.600          3 Report
```

Alert level G

```
PLAT002_ALERT_2_G Number of Distance or Angle Restraints on AtSite          4 Note
PLAT003_ALERT_2_G Number of Uiso or Uij Restrained non-H Atoms ...          4 Report
PLAT142_ALERT_4_G s.u. on b - Axis Small or Missing .............     0.00030 Ang.
PLAT143_ALERT_4_G s.u. on c - Axis Small or Missing .............     0.00005 Ang.
PLAT172_ALERT_4_G The CIF-Embedded .res File Contains DFIX Records          1 Report
PLAT178_ALERT_4_G The CIF-Embedded .res File Contains SIMU Records          1 Report
PLAT187_ALERT_4_G The CIF-Embedded .res File Contains RIGU Records          1 Report
PLAT301_ALERT_3_G Main Residue  Disorder ..............(Resd  1  )        17% Note
PLAT410_ALERT_2_G Short Intra H...H Contact  H5       ..H7B      .       1.66 Ang.
                                                        x,y,z  =      1_555 Check
PLAT410_ALERT_2_G Short Intra H...H Contact  H11      ..H8B      .       1.72 Ang.
                                                  1-x,1-y,-z  =      5_665 Check
PLAT722_ALERT_1_G Angle   Calc      121.00, Rep      119.40 Dev...       1.60 Degree
              C8B     -C7B     -H7B        1_555  1_555  1_555     #   28 Check
PLAT860_ALERT_3_G Number of Least-Squares Restraints ............         137 Note
PLAT912_ALERT_4_G Missing # of FCF Reflections Above STh/L=  0.600          5 Note
PLAT913_ALERT_3_G Missing # of Very Strong Reflections in FCF ....          1 Note
```

```
PLAT933_ALERT_2_G Number of OMIT Records in Embedded .res File ...          3 Note
PLAT978_ALERT_2_G Number C-C Bonds with Positive Residual Density.         10 Info
```

```
   0 ALERT level A = Most likely a serious problem - resolve or explain
   0 ALERT level B = A potentially serious problem, consider carefully
   1 ALERT level C = Check. Ensure it is not caused by an omission or oversight
  16 ALERT level G = General information/check it is not something unexpected

   1 ALERT type 1 CIF construction/syntax error, inconsistent or missing data
   6 ALERT type 2 Indicator that the structure model may be wrong or deficient
   4 ALERT type 3 Indicator that the structure quality may be low
   6 ALERT type 4 Improvement, methodology, query or suggestion
   0 ALERT type 5 Informative message, check
```

Datablock: diolefinBr_290K

```
Bond precision:  C-C = 0.0027 A          Wavelength=1.54178

Cell:            a=7.44909(8)     b=39.4937(4)     c=5.93597(6)
                 alpha=90         beta=90          gamma=90
Temperature:     290 K
```

	Calculated	Reported
Volume	1746.32(3)	1746.31(3)
Space group	P c c n	P c c n
Hall group	-P 2ab 2ac	-P 2ab 2ac
Moiety formula	C22 H16 Br2	?
Sum formula	C22 H16 Br2	C22 H16 Br2
Mr	440.15	440.17
Dx,g cm-3	1.674	1.674
Z	4	4
Mu (mm-1)	5.861	5.861
F000	872.0	872.0
F000’	868.12	
h,k,lmax	9,50,7	9,50,7
Nref	1858	1843
Tmin,Tmax	0.430,0.746	0.650,1.000
Tmin’	0.377	

```
Correction method= # Reported T Limits: Tmin=0.650 Tmax=1.000
AbsCorr = GAUSSIAN

Data completeness= 0.992          Theta(max)= 77.433

R(reflections)= 0.0263( 1641)     wR2(reflections)= 0.0700( 1843)

S = 1.115                   Npar= 130
```

```
The following ALERTS were generated. Each ALERT has the format
       test-name_ALERT_alert-type_alert-level.
Click on the hyperlinks for more details of the test.
```

Alert level C

```
PLAT911_ALERT_3_C Missing FCF Refl Between Thmin & STh/L=    0.600          3 Report
```

Alert level G

```
PLAT002_ALERT_2_G Number of Distance or Angle Restraints on AtSite          4 Note
PLAT003_ALERT_2_G Number of Uiso or Uij Restrained non-H Atoms ...          4 Report
PLAT172_ALERT_4_G The CIF-Embedded .res File Contains DFIX Records          1 Report
PLAT178_ALERT_4_G The CIF-Embedded .res File Contains SIMU Records          1 Report
PLAT187_ALERT_4_G The CIF-Embedded .res File Contains RIGU Records          1 Report
PLAT301_ALERT_3_G Main Residue  Disorder ..............(Resd  1  )        17% Note
PLAT410_ALERT_2_G Short Intra H...H Contact  H5       ..H7B      .       1.61 Ang.
                                                     x,y,z  =      1_555 Check
PLAT410_ALERT_2_G Short Intra H...H Contact  H11      ..H8B      .       1.81 Ang.
                                                1-x,1-y,-z  =      5_665 Check
PLAT860_ALERT_3_G Number of Least-Squares Restraints ............        137 Note
PLAT912_ALERT_4_G Missing # of FCF Reflections Above STh/L=  0.600         12 Note
PLAT913_ALERT_3_G Missing # of Very Strong Reflections in FCF ....          1 Note
PLAT933_ALERT_2_G Number of OMIT Records in Embedded .res File ...          3 Note
PLAT978_ALERT_2_G Number C-C Bonds with Positive Residual Density.          8 Info
```

```
   0 ALERT level A = Most likely a serious problem - resolve or explain
   0 ALERT level B = A potentially serious problem, consider carefully
   1 ALERT level C = Check. Ensure it is not caused by an omission or oversight
  13 ALERT level G = General information/check it is not something unexpected

   0 ALERT type 1 CIF construction/syntax error, inconsistent or missing data
   6 ALERT type 2 Indicator that the structure model may be wrong or deficient
   4 ALERT type 3 Indicator that the structure quality may be low
   4 ALERT type 4 Improvement, methodology, query or suggestion
   0 ALERT type 5 Informative message, check
```

Validation response form

Please find below a validation response form (VRF) that can be filled in and pasted into your CIF.

```
# start Validation Reply Form
_vrf_PLAT911_diolefinI_250K
;
PROBLEM: Missing FCF Refl Between Thmin & STh/L=    0.600          2 Report
RESPONSE: ...
;
_vrf_PLAT911_diolefinI_270K
;
PROBLEM: Missing FCF Refl Between Thmin & STh/L=    0.600          3 Report
RESPONSE: ...
;
_vrf_PLAT911_diolefinI_290K
;
PROBLEM: Missing FCF Refl Between Thmin & STh/L=    0.600          3 Report
RESPONSE: ...
;
_vrf_PLAT911_diolefinBr_230K
```

```
;
PROBLEM: Missing FCF Refl Between Thmin & STh/L=    0.600          2 Report
RESPONSE: ...
;
_vrf_PLAT911_diolefinBr_250K
;
PROBLEM: Missing FCF Refl Between Thmin & STh/L=    0.600          3 Report
RESPONSE: ...
;
_vrf_PLAT911_diolefinBr_270K
;
PROBLEM: Missing FCF Refl Between Thmin & STh/L=    0.600          3 Report
RESPONSE: ...
;
_vrf_PLAT911_diolefinBr_290K
;
PROBLEM: Missing FCF Refl Between Thmin & STh/L=    0.600          3 Report
RESPONSE: ...
;
# end Validation Reply Form
```

It is advisable to attempt to resolve as many as possible of the alerts in all categories. Often the minor alerts point to easily fixed oversights, errors and omissions in your CIF or refinement strategy, so attention to these fine details can be worthwhile. In order to resolve some of the more serious problems it may be necessary to carry out additional measurements or structure refinements. However, the purpose of your study may justify the reported deviations and the more serious of these should normally be commented upon in the discussion or experimental section of a paper or in the "special_details" fields of the CIF. checkCIF was carefully designed to identify outliers and unusual parameters, but every test has its limitations and alerts that are not important in a particular case may appear. Conversely, the absence of alerts does not guarantee there are no aspects of the results needing attention. It is up to the individual to critically assess their own results and, if necessary, seek expert advice.

Publication of your CIF in IUCr journals

A basic structural check has been run on your CIF. These basic checks will be run on all CIFs submitted for publication in IUCr journals (*Acta Crystallographica*, *Journal of Applied Crystallography*, *Journal of Synchrotron Radiation*); however, if you intend to submit to *Acta Crystallographica Section C* or *E* or *IUCrData*, you should make sure that full publication checks are run on the final version of your CIF prior to submission.

Publication of your CIF in other journals

Please refer to the *Notes for Authors* of the relevant journal for any special instructions relating to CIF submission.

PLATON version of 03/06/2021; check.def file version of 02/06/2021

Datablock diolefinI_190K - ellipsoid plot

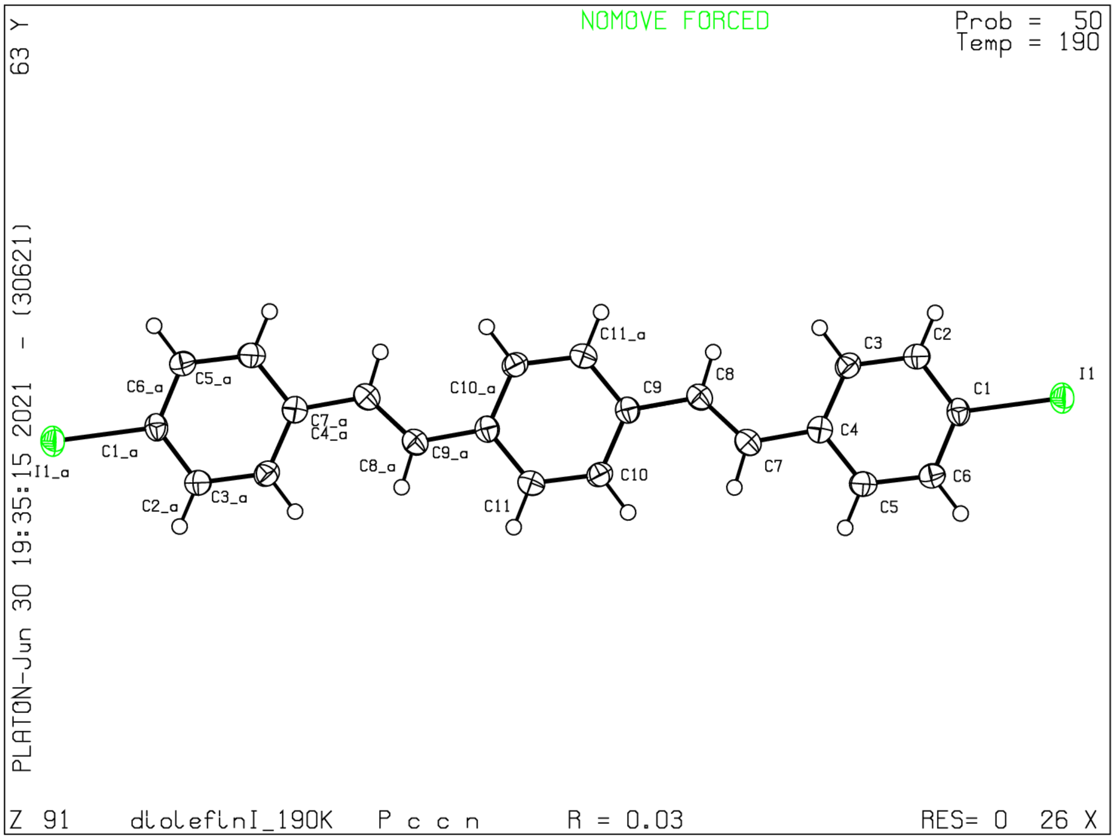

Datablock diolefinI_210K - ellipsoid plot

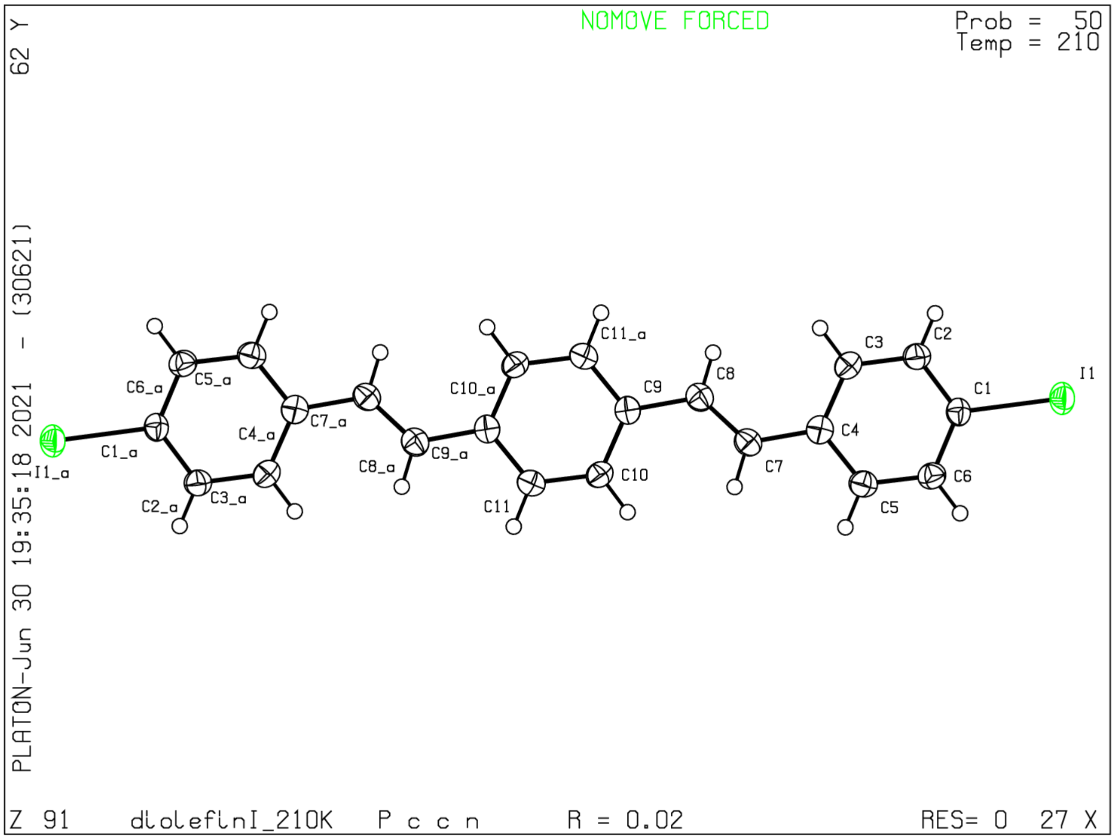

Datablock diolefinI_230K - ellipsoid plot

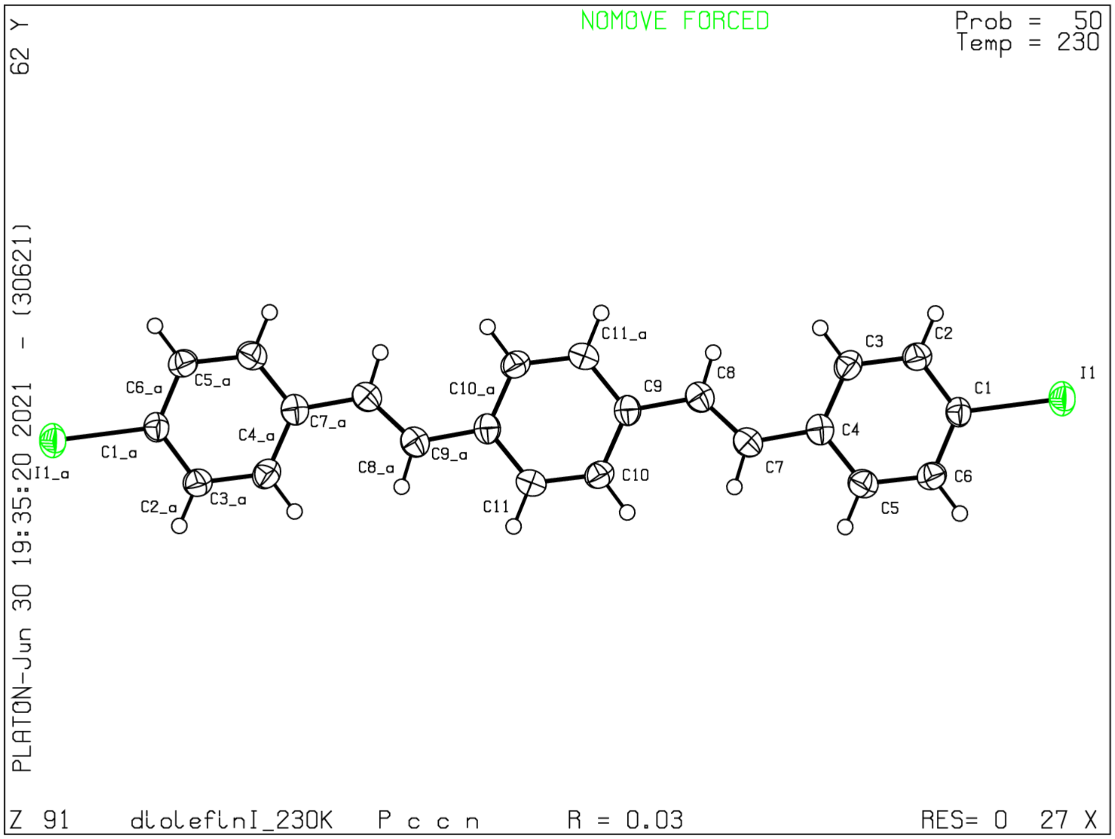

Datablock diolefinI_250K - ellipsoid plot

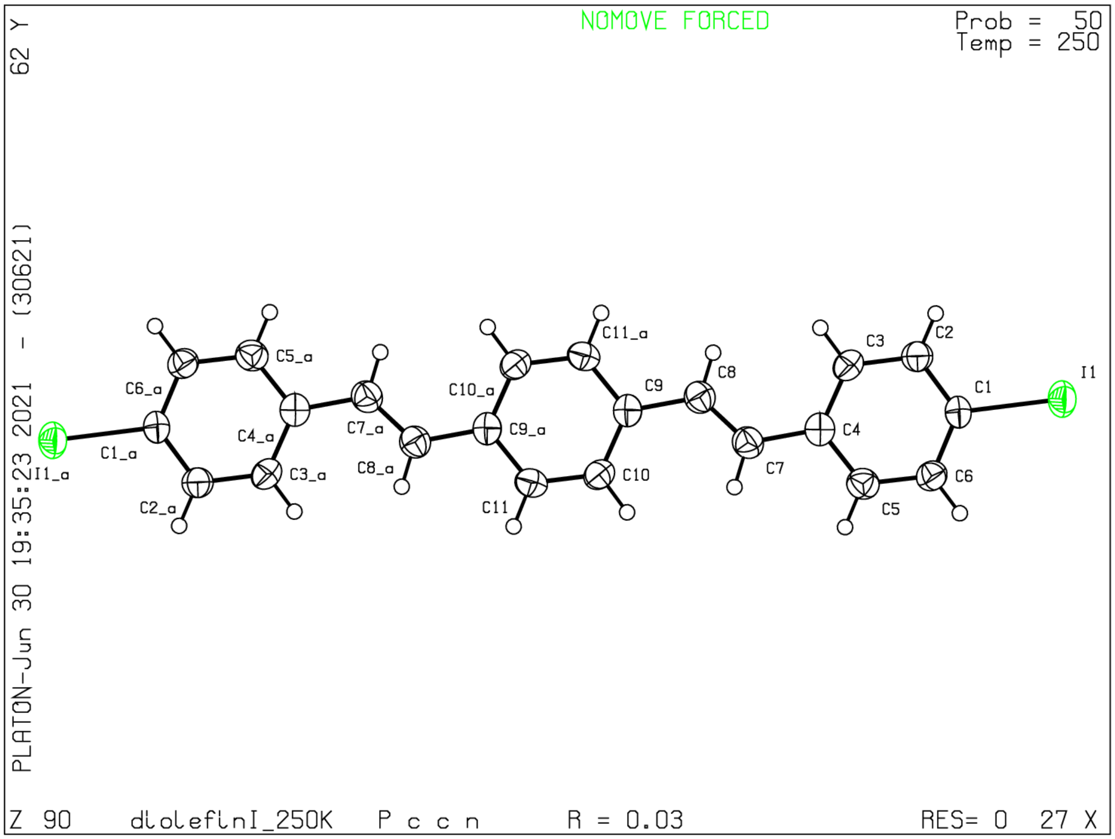

Datablock diolefinI_270K - ellipsoid plot

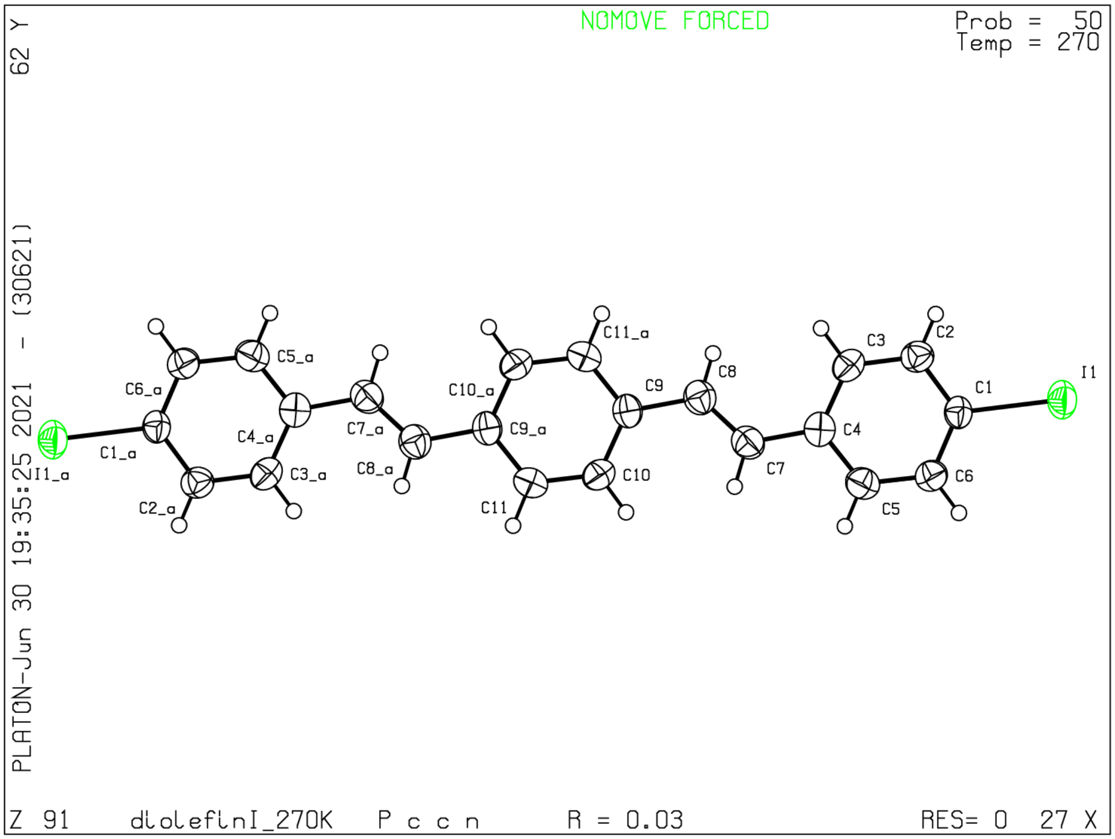

Datablock diolefinI_290K - ellipsoid plot

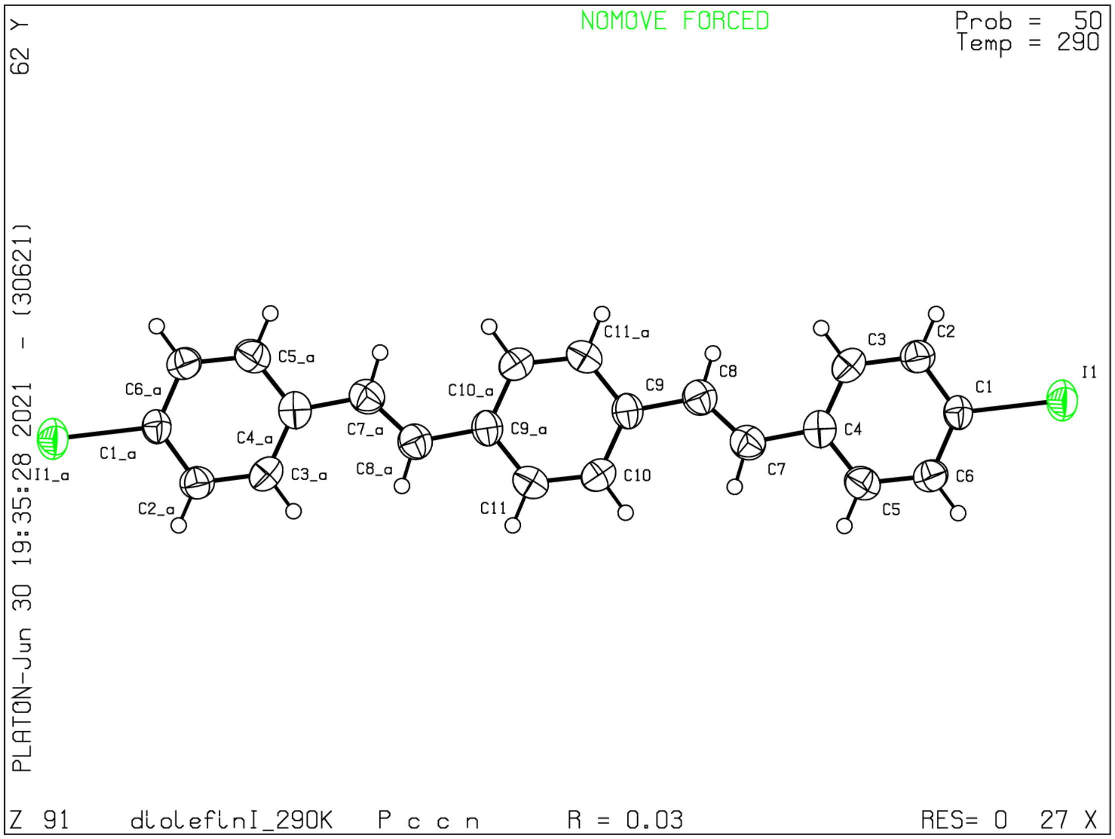

Datablock diolefinBr_190K - ellipsoid plot

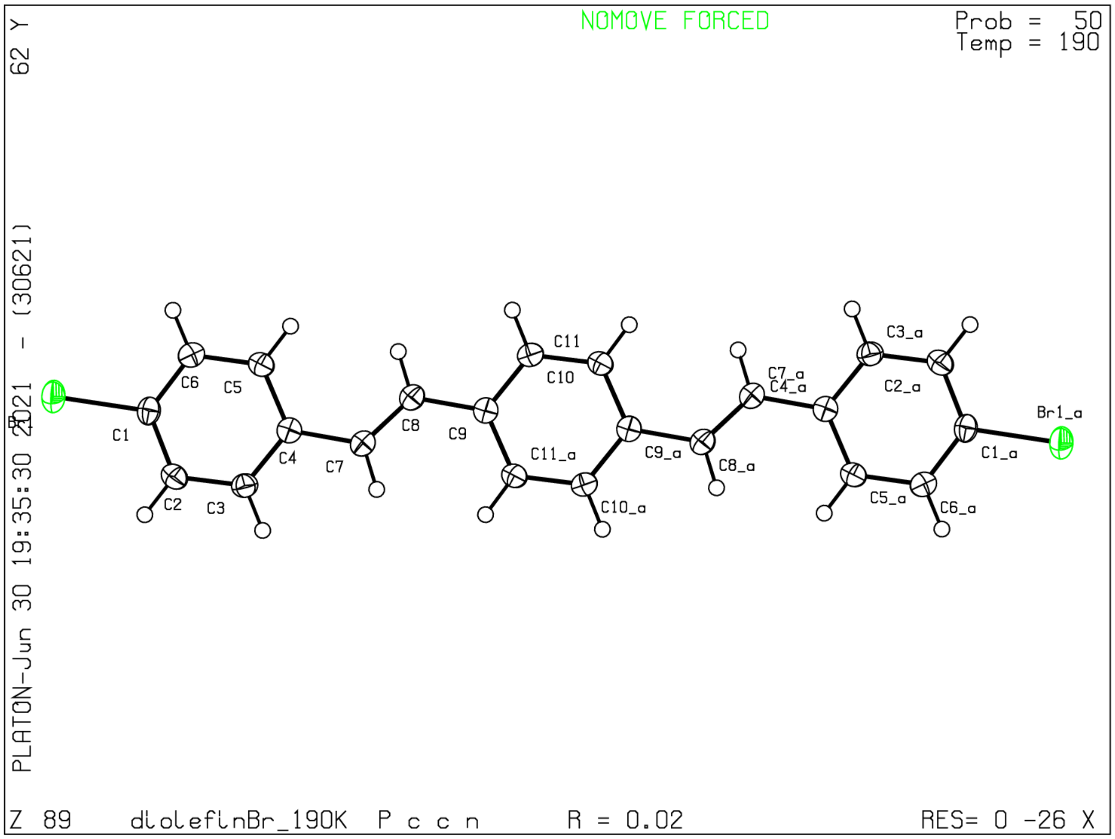

Datablock diolefinBr_210K - ellipsoid plot

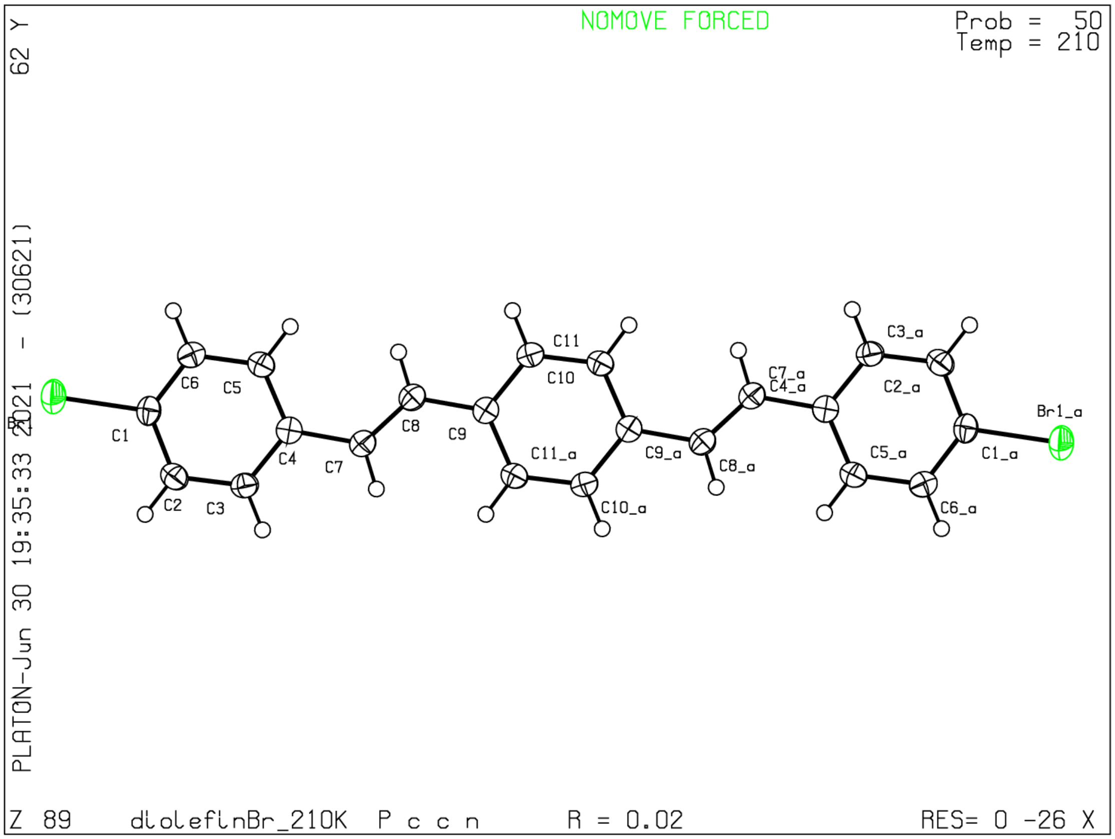

Datablock diolefinBr_230K - ellipsoid plot

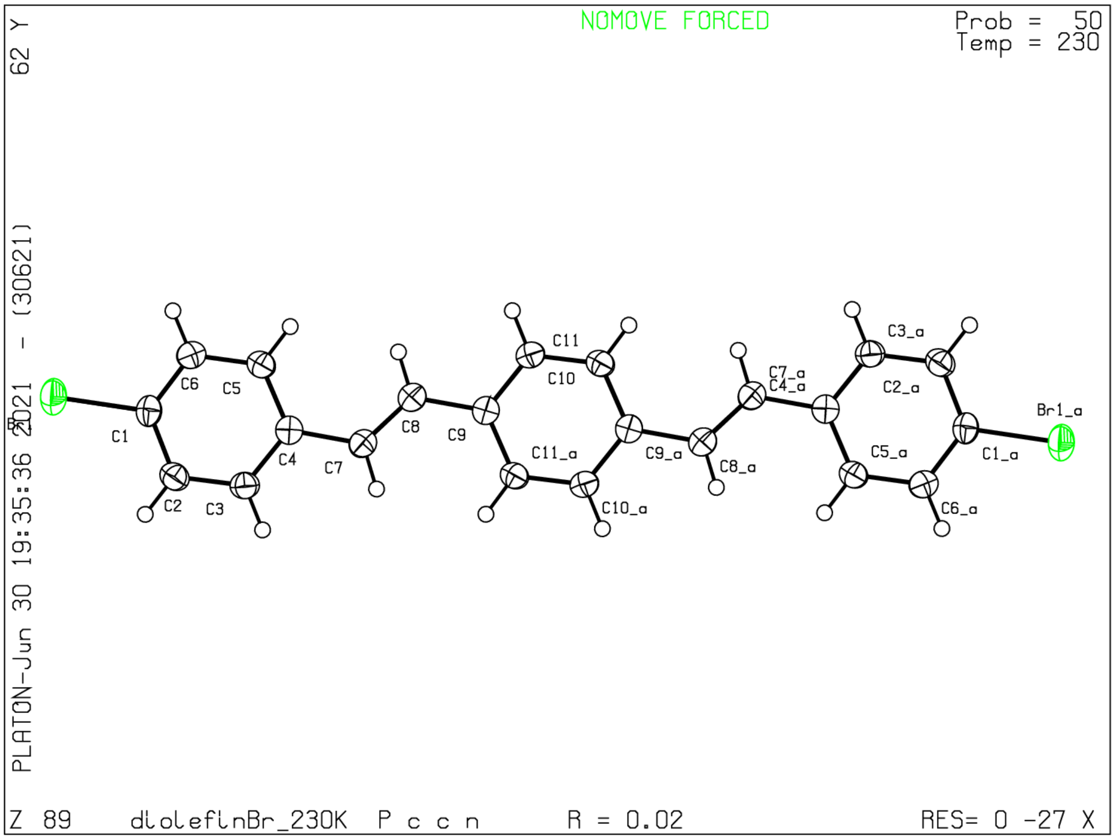

Datablock diolefinBr_250K - ellipsoid plot

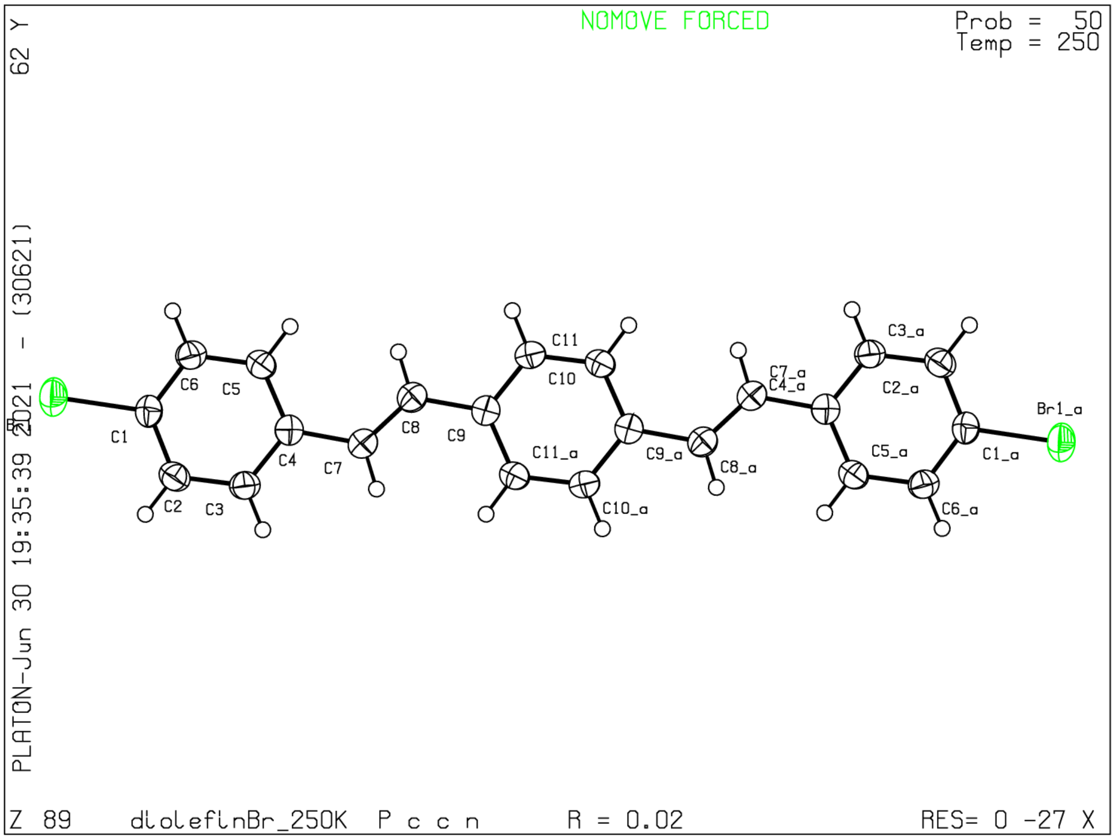

Datablock diolefinBr_270K - ellipsoid plot

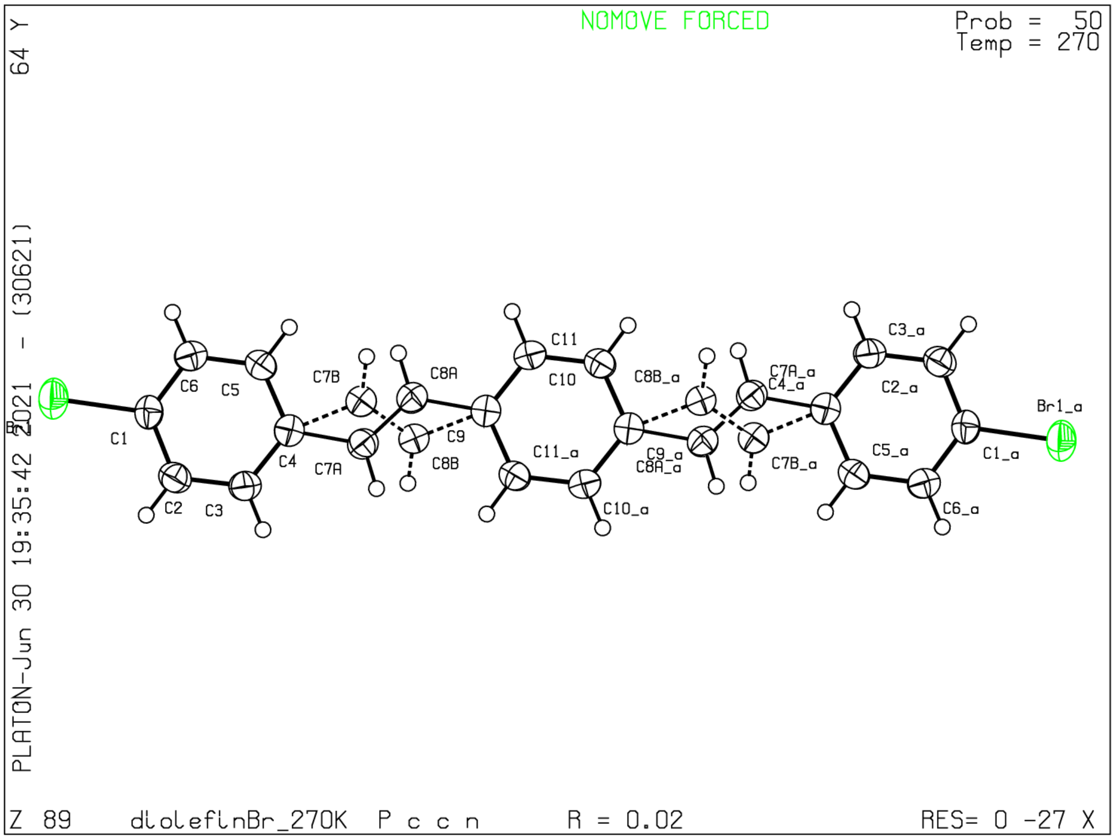

Datablock diolefinBr_290K - ellipsoid plot

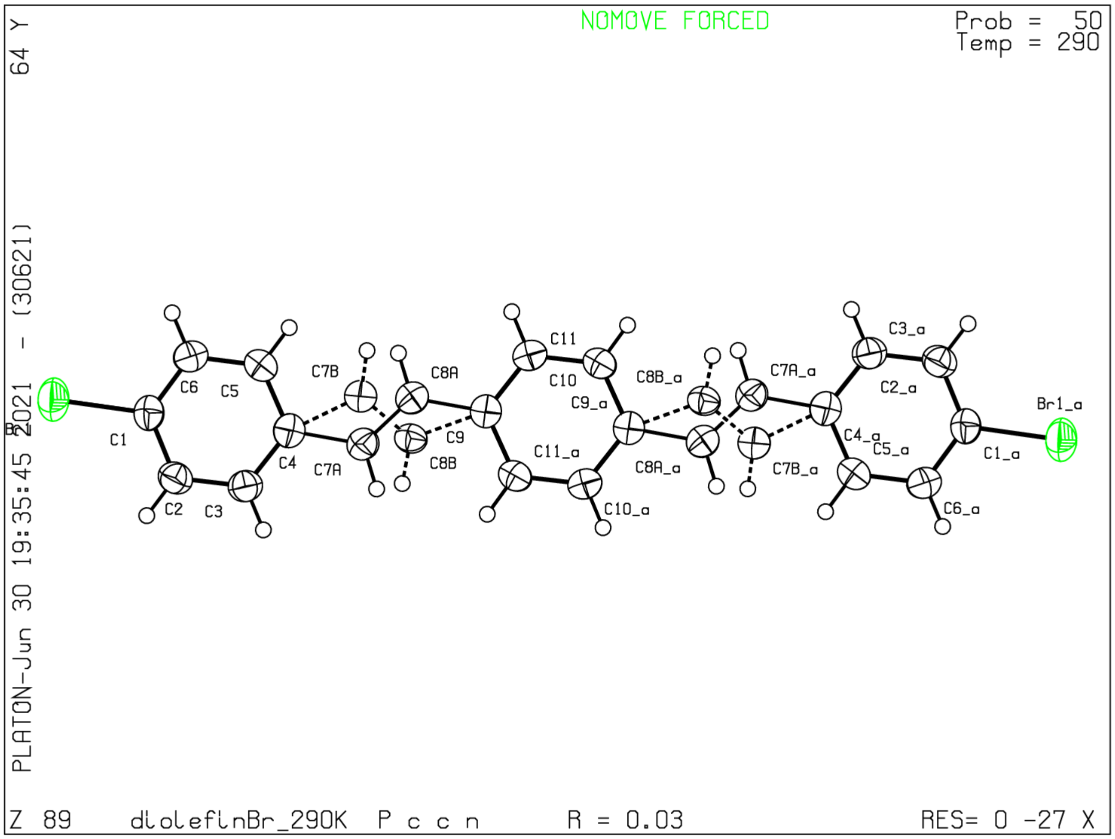

Supplement: Supplementary file 3 [file m-09-00031-sup3.zip › diolefin-I + diolefin-Br.pdf]
