# Supplementary material for: Differences in thermal expansion and motion ability for herringbone and face-to-face π-stacked solids
Source: IUCrJ. 2021 Nov 3;9(Pt 1):31–42. doi: 10.1107/S2052252521009593 (PMC8733877; doi:10.1107/S2052252521009593)

checkCIF/PLATON report

Structure factors have been supplied for datablock(s) azoBr_a_190, azoBr_a_210, azoBr_a_230, azoBr_a_250, azoBr_a_270, azoBr_a_290, azoI_190, azoI_210, azoI_230, azoI_250, azoI_270, azoI_290

THIS REPORT IS FOR GUIDANCE ONLY. IF USED AS PART OF A REVIEW PROCEDURE FOR PUBLICATION, IT SHOULD NOT REPLACE THE EXPERTISE OF AN EXPERIENCED CRYSTALLOGRAPHIC REFEREE.

No syntax errors found.   CIF dictionary   Interpreting this report

Datablock: azoI_190

```
Bond precision:  = 0.0000 A              Wavelength=1.54178

Cell:             a=7.35645(13)     b=28.3296(4)     c=6.01030(9)
                  alpha=90          beta=90          gamma=90
Temperature:      190 K

                 Calculated                 Reported
Volume           1252.58(3)                 1252.58(3)
Space group      P c c n                    P c c n
Hall group       -P 2ab 2ac                 -P 2ab 2ac
Moiety formula   C12 H8 I2 N2               C12 H8 I2 N2
Sum formula      C12 H8 I2 N2               C12 H8 I2 N2
Mr               434.00                     434.00
Dx,g cm-3        2.301                      2.301
Z                4                          4
Mu (mm-1)        39.225                     39.225
F000             800.0                      800.0
F000'            800.34
h,k,lmax         9,35,7                     8,35,7
Nref             1323                       1304
Tmin,Tmax        0.032,0.513                0.094,0.643
Tmin'            0.005

Correction method= # Reported T Limits: Tmin=0.094 Tmax=0.643
AbsCorr = GAUSSIAN

Data completeness= 0.986          Theta(max)= 77.387

R(reflections)= 0.0203( 1237)     wR2(reflections)= 0.0557( 1304)

S = 1.136                  Npar= 137
```

The following ALERTS were generated. Each ALERT has the format
test-name_ALERT_alert-type_alert-level.
Click on the hyperlinks for more details of the test.

Alert level C

```
PLAT088_ALERT_3_C Poor Data / Parameter Ratio ....................       9.52 Note
```

Author Response: Due to the number of atoms that were modeled as positionally disordered.

Alert level G

```
PLAT002_ALERT_2_G Number of Distance or Angle Restraints on AtSite          8 Note
PLAT003_ALERT_2_G Number of Uiso or Uij Restrained non-H Atoms ...         16 Report
PLAT172_ALERT_4_G The CIF-Embedded .res File Contains DFIX Records          2 Report
PLAT178_ALERT_4_G The CIF-Embedded .res File Contains SIMU Records          1 Report
PLAT187_ALERT_4_G The CIF-Embedded .res File Contains RIGU Records          1 Report
PLAT301_ALERT_3_G Main Residue  Disorder ..............(Resd  1  )       100% Note
PLAT301_ALERT_3_G Main Residue  Disorder ..............(Resd  2  )       100% Note
PLAT304_ALERT_4_G Non-Integer Number of Atoms in ..... (Resd  1  )      22.25 Check
PLAT304_ALERT_4_G Non-Integer Number of Atoms in ..... (Resd  2  )       1.75 Check
PLAT860_ALERT_3_G Number of Least-Squares Restraints .............        334 Note
PLAT912_ALERT_4_G Missing # of FCF Reflections Above STh/L=  0.600          7 Note
PLAT933_ALERT_2_G Number of OMIT Records in Embedded .res File ...          1 Note
```

```
   0 ALERT level A = Most likely a serious problem - resolve or explain
   0 ALERT level B = A potentially serious problem, consider carefully
   1 ALERT level C = Check. Ensure it is not caused by an omission or oversight
  12 ALERT level G = General information/check it is not something unexpected

   0 ALERT type 1 CIF construction/syntax error, inconsistent or missing data
   3 ALERT type 2 Indicator that the structure model may be wrong or deficient
   4 ALERT type 3 Indicator that the structure quality may be low
   6 ALERT type 4 Improvement, methodology, query or suggestion
   0 ALERT type 5 Informative message, check
```

Datablock: azoI_210

```
Bond precision:  = 0.0000 A             Wavelength=1.54178

Cell:            a=7.37426(12)     b=28.3456(4)     c=6.01735(9)
                 alpha=90          beta=90          gamma=90
Temperature:     210 K
```

	Calculated	Reported
Volume	1257.79(3)	1257.79(3)
Space group	P c c n	P c c n
Hall group	-P 2ab 2ac	-P 2ab 2ac
Moiety formula	C12 H8 I2 N2	C12 H8 I2 N2
Sum formula	C12 H8 I2 N2	C12 H8 I2 N2
Mr	434.00	434.00
Dx,g cm-3	2.292	2.292
Z	4	4
Mu (mm-1)	39.062	39.062
F000	800.0	800.0
F000’	800.34	
h,k,lmax	9,35,7	9,35,7
Nref	1332	1304
Tmin,Tmax	0.033,0.515	0.505,1.000
Tmin’	0.005	

Correction method= # Reported T Limits: Tmin=0.505 Tmax=1.000
AbsCorr = GAUSSIAN

Data completeness= 0.979   Theta(max)= 77.405

R(reflections)= 0.0258(1228)   wR2(reflections)= 0.0731(1304)

S = 1.105   Npar= 137

The following ALERTS were generated. Each ALERT has the format
test-name_ALERT_alert-type_alert-level.
Click on the hyperlinks for more details of the test.

Alert level C

PLAT088_ALERT_3_C Poor Data / Parameter Ratio .... 9.52 Note

Author Response: Due to the number of atoms that were modeled as positionally disordered.

PLAT911_ALERT_3_C Missing FCF Refl Between Thmin & STh/L= 0.600 3 Report

Author Response: These reflections were omitted during the absorption correction.

Alert level G

PLAT002_ALERT_2_G Number of Distance or Angle Restraints on AtSite 8 Note
PLAT003_ALERT_2_G Number of Uiso or Uij Restrained non-H Atoms ... 16 Report
PLAT172_ALERT_4_G The CIF-Embedded .res File Contains DFIX Records 2 Report
PLAT178_ALERT_4_G The CIF-Embedded .res File Contains SIMU Records 1 Report
PLAT187_ALERT_4_G The CIF-Embedded .res File Contains RIGU Records 1 Report
PLAT301_ALERT_3_G Main Residue Disorder ....(Resd 1) 100% Note

```
PLAT301_ALERT_3_G Main Residue  Disorder .............(Resd  2  )       100% Note
PLAT304_ALERT_4_G Non-Integer Number of Atoms in ..... (Resd  1  )      21.02 Check
PLAT304_ALERT_4_G Non-Integer Number of Atoms in ..... (Resd  2  )       2.98 Check
PLAT860_ALERT_3_G Number of Least-Squares Restraints .............        328 Note
PLAT883_ALERT_1_G No Info/Value for _atom_sites_solution_primary .     Please Do !
PLAT912_ALERT_4_G Missing # of FCF Reflections Above STh/L=  0.600         25 Note
```

```
   0 ALERT level A = Most likely a serious problem - resolve or explain
   0 ALERT level B = A potentially serious problem, consider carefully
   2 ALERT level C = Check. Ensure it is not caused by an omission or oversight
  12 ALERT level G = General information/check it is not something unexpected

   1 ALERT type 1 CIF construction/syntax error, inconsistent or missing data
   2 ALERT type 2 Indicator that the structure model may be wrong or deficient
   5 ALERT type 3 Indicator that the structure quality may be low
   6 ALERT type 4 Improvement, methodology, query or suggestion
   0 ALERT type 5 Informative message, check
```

Datablock: azoI_230

```
Bond precision:  = 0.0000 A            Wavelength=1.54178

Cell:            a=7.39123(10)     b=28.3542(3)     c=6.02553(8)
                 alpha=90          beta=90          gamma=90
Temperature:     230 K
```

	Calculated	Reported
Volume	1262.79(3)	1262.78(3)
Space group	P c c n	P c c n
Hall group	-P 2ab 2ac	-P 2ab 2ac
Moiety formula	C12 H8 I2 N2	C12 H8 I2 N2
Sum formula	C12 H8 I2 N2	C12 H8 I2 N2
Mr	434.00	434.00
Dx,g cm-3	2.283	2.283
Z	4	4
Mu (mm-1)	38.908	38.908
F000	800.0	800.0
F000’	800.34	
h,k,lmax	9,35,7	9,35,7
Nref	1341	1310
Tmin,Tmax	0.033,0.516	0.095,0.636
Tmin’	0.005	

```
Correction method= # Reported T Limits: Tmin=0.095 Tmax=0.636
AbsCorr = GAUSSIAN

Data completeness= 0.977          Theta(max)= 77.558
```

```
R(reflections)= 0.0214( 1203)     wR2(reflections)= 0.0613( 1310)

S = 1.084                     Npar= 137
```

The following ALERTS were generated. Each ALERT has the format
test-name_ALERT_alert-type_alert-level.
Click on the hyperlinks for more details of the test.

Alert level C

```
PLAT088_ALERT_3_C Poor Data / Parameter Ratio ....................       9.56 Note
```

Author Response: Due to the number of atoms that were modeled as positionally disordered.

```
PLAT911_ALERT_3_C Missing FCF Refl Between Thmin & STh/L=    0.600          2 Report
```

Author Response: These reflections were omitted during the absorption correction.

Alert level G

```
PLAT002_ALERT_2_G Number of Distance or Angle Restraints on AtSite          8 Note
PLAT003_ALERT_2_G Number of Uiso or Uij Restrained non-H Atoms ...         16 Report
PLAT172_ALERT_4_G The CIF-Embedded .res File Contains DFIX Records          2 Report
PLAT178_ALERT_4_G The CIF-Embedded .res File Contains SIMU Records          1 Report
PLAT187_ALERT_4_G The CIF-Embedded .res File Contains RIGU Records          1 Report
PLAT301_ALERT_3_G Main Residue  Disorder .............(Resd  1  )       100% Note
PLAT301_ALERT_3_G Main Residue  Disorder .............(Resd  2  )       100% Note
PLAT304_ALERT_4_G Non-Integer Number of Atoms in ..... (Resd  1  )      20.40 Check
PLAT304_ALERT_4_G Non-Integer Number of Atoms in ..... (Resd  2  )       3.60 Check
PLAT860_ALERT_3_G Number of Least-Squares Restraints ............        328 Note
PLAT883_ALERT_1_G No Info/Value for _atom_sites_solution_primary .     Please Do !
PLAT912_ALERT_4_G Missing # of FCF Reflections Above STh/L=  0.600         30 Note
```

```
   0 ALERT level A = Most likely a serious problem - resolve or explain
   0 ALERT level B = A potentially serious problem, consider carefully
   2 ALERT level C = Check. Ensure it is not caused by an omission or oversight
  12 ALERT level G = General information/check it is not something unexpected

   1 ALERT type 1 CIF construction/syntax error, inconsistent or missing data
   2 ALERT type 2 Indicator that the structure model may be wrong or deficient
   5 ALERT type 3 Indicator that the structure quality may be low
   6 ALERT type 4 Improvement, methodology, query or suggestion
   0 ALERT type 5 Informative message, check
```

Datablock: azoI_250

```
Bond precision:  = 0.0000 A                Wavelength=1.54178
```

Cell:	a=7.4085(2)	b=28.3671(6)	c=6.03045(15)
	alpha=90	beta=90	gamma=90
Temperature:	250 K		

	Calculated	Reported
Volume	1267.35(5)	1267.34(5)
Space group	P c c n	P c c n
Hall group	-P 2ab 2ac	-P 2ab 2ac
Moiety formula	C12 H8 I2 N2	C12 H8 I2 N2
Sum formula	C12 H8 I2 N2	C12 H8 I2 N2
Mr	434.00	434.00
Dx,g cm-3	2.275	2.275
Z	4	4
Mu (mm-1)	38.768	38.768
F000	800.0	800.0
F000’	800.34	
h,k,lmax	9,35,7	9,35,7
Nref	1342	1324
Tmin,Tmax	0.034,0.517	0.102,0.636
Tmin’	0.005	

Correction method= # Reported T Limits: Tmin=0.102 Tmax=0.636
AbsCorr = GAUSSIAN

Data completeness= 0.987 Theta(max)= 77.302

R(reflections)= 0.0199(1208) wR2(reflections)= 0.0523(1324)

S = 1.058 Npar= 137

The following ALERTS were generated. Each ALERT has the format
test-name_ALERT_alert-type_alert-level.
Click on the hyperlinks for more details of the test.

Alert level C

PLAT088_ALERT_3_C Poor Data / Parameter Ratio .... 9.66 Note

Author Response: Due to the number of atoms that were modeled as positionally disordered.

Alert level G

PLAT002_ALERT_2_G Number of Distance or Angle Restraints on AtSite 8 Note
PLAT003_ALERT_2_G Number of Uiso or Uij Restrained non-H Atoms ... 16 Report
PLAT172_ALERT_4_G The CIF-Embedded .res File Contains DFIX Records 2 Report
PLAT178_ALERT_4_G The CIF-Embedded .res File Contains SIMU Records 1 Report
PLAT187_ALERT_4_G The CIF-Embedded .res File Contains RIGU Records 1 Report
PLAT301_ALERT_3_G Main Residue Disorder ....(Resd 1) 100% Note

```
PLAT301_ALERT_3_G Main Residue  Disorder .............(Resd  2  )       100% Note
PLAT304_ALERT_4_G Non-Integer Number of Atoms in ..... (Resd  1  )      19.20 Check
PLAT304_ALERT_4_G Non-Integer Number of Atoms in ..... (Resd  2  )       4.80 Check
PLAT860_ALERT_3_G Number of Least-Squares Restraints .............        328 Note
PLAT883_ALERT_1_G No Info/Value for _atom_sites_solution_primary .     Please Do !
PLAT912_ALERT_4_G Missing # of FCF Reflections Above STh/L=  0.600         18 Note
```

---

0 **ALERT level A** = Most likely a serious problem - resolve or explain
0 **ALERT level B** = A potentially serious problem, consider carefully
1 **ALERT level C** = Check. Ensure it is not caused by an omission or oversight
12 **ALERT level G** = General information/check it is not something unexpected

1 ALERT type 1 CIF construction/syntax error, inconsistent or missing data
2 ALERT type 2 Indicator that the structure model may be wrong or deficient
4 ALERT type 3 Indicator that the structure quality may be low
6 ALERT type 4 Improvement, methodology, query or suggestion
0 ALERT type 5 Informative message, check

Datablock: azoI_270

Bond precision: = 0.0000 A   Wavelength=1.54178

Cell:	a=7.4262(2)	b=28.3821(6)	c=6.03869(15)
	alpha=90	beta=90	gamma=90
Temperature:	270 K		

	Calculated	Reported
Volume	1272.78(5)	1272.78(5)
Space group	P c c n	P c c n
Hall group	-P 2ab 2ac	-P 2ab 2ac
Moiety formula	C12 H8 I2 N2	C12 H8 I2 N2
Sum formula	C12 H8 I2 N2	C12 H8 I2 N2
Mr	434.00	434.00
Dx,g cm-3	2.265	2.265
Z	4	4
Mu (mm-1)	38.602	38.602
F000	800.0	800.0
F000’	800.34	
h,k,lmax	9,35,7	9,35,7
Nref	1346	1326
Tmin,Tmax	0.034,0.519	0.104,0.636
Tmin’	0.006	

Correction method= # Reported T Limits: Tmin=0.104 Tmax=0.636
AbsCorr = GAUSSIAN

Data completeness= 0.985   Theta(max)= 77.077

```
R(reflections)= 0.0219( 1206)     wR2(reflections)= 0.0589( 1326)

S = 1.067                    Npar= 137
```

```
The following ALERTS were generated. Each ALERT has the format
       test-name_ALERT_alert-type_alert-level.
Click on the hyperlinks for more details of the test.
```

Alert level C

```
PLAT088_ALERT_3_C Poor Data / Parameter Ratio ....................       9.68 Note
```

Author Response: Due to the number of atoms that were modeled as positionally disordered.

```
PLAT911_ALERT_3_C Missing FCF Refl Between Thmin & STh/L=    0.600          3 Report
```

Author Response: These reflections were omitted during the absorption correction.

Alert level G

```
PLAT002_ALERT_2_G Number of Distance or Angle Restraints on AtSite          8 Note
PLAT003_ALERT_2_G Number of Uiso or Uij Restrained non-H Atoms ...         16 Report
PLAT172_ALERT_4_G The CIF-Embedded .res File Contains DFIX Records          2 Report
PLAT178_ALERT_4_G The CIF-Embedded .res File Contains SIMU Records          1 Report
PLAT187_ALERT_4_G The CIF-Embedded .res File Contains RIGU Records          1 Report
PLAT301_ALERT_3_G Main Residue  Disorder .............(Resd  1  )       100% Note
PLAT301_ALERT_3_G Main Residue  Disorder .............(Resd  2  )       100% Note
PLAT304_ALERT_4_G Non-Integer Number of Atoms in ..... (Resd  1  )      18.82 Check
PLAT304_ALERT_4_G Non-Integer Number of Atoms in ..... (Resd  2  )       5.18 Check
PLAT860_ALERT_3_G Number of Least-Squares Restraints .............        328 Note
PLAT883_ALERT_1_G No Info/Value for _atom_sites_solution_primary .     Please Do !
PLAT912_ALERT_4_G Missing # of FCF Reflections Above STh/L=  0.600         17 Note
PLAT992_ALERT_5_G Repd & Actual _reflns_number_gt Values Differ by          2 Check
```

```
   0 ALERT level A = Most likely a serious problem - resolve or explain
   0 ALERT level B = A potentially serious problem, consider carefully
   2 ALERT level C = Check. Ensure it is not caused by an omission or oversight
  13 ALERT level G = General information/check it is not something unexpected

   1 ALERT type 1 CIF construction/syntax error, inconsistent or missing data
   2 ALERT type 2 Indicator that the structure model may be wrong or deficient
   5 ALERT type 3 Indicator that the structure quality may be low
   6 ALERT type 4 Improvement, methodology, query or suggestion
   1 ALERT type 5 Informative message, check
```

Datablock: azoI_290

```
Bond precision:  = 0.0000 A                 Wavelength=1.54178
```

Cell:	a=7.44541(17)	b=28.3989(5)	c=6.04700(12)
	alpha=90	beta=90	gamma=90
Temperature:	290 K		

	Calculated	Reported
Volume	1278.59(4)	1278.59(4)
Space group	P c c n	P c c n
Hall group	-P 2ab 2ac	-P 2ab 2ac
Moiety formula	C12 H8 I2 N2	C12 H8 I2 N2
Sum formula	C12 H8 I2 N2	C12 H8 I2 N2
Mr	434.00	434.00
Dx,g cm-3	2.255	2.255
Z	4	4
Mu (mm-1)	38.427	38.427
F000	800.0	800.0
F000’	800.34	
h,k,lmax	9,35,7	9,35,7
Nref	1355	1334
Tmin,Tmax	0.035,0.520	0.098,0.635
Tmin’	0.006	

Correction method= # Reported T Limits: Tmin=0.098 Tmax=0.635
AbsCorr = GAUSSIAN

Data completeness= 0.985 Theta(max)= 77.276

R(reflections)= 0.0212(1175) wR2(reflections)= 0.0581(1334)

S = 1.056 Npar= 137

The following ALERTS were generated. Each ALERT has the format
test-name_ALERT_alert-type_alert-level.
Click on the hyperlinks for more details of the test.

Alert level C

PLAT088_ALERT_3_C Poor Data / Parameter Ratio .... 9.74 Note

Author Response: Due to the number of atoms that were modeled as positionally disordered.

PLAT911_ALERT_3_C Missing FCF Refl Between Thmin & STh/L= 0.600 4 Report

Author Response: These reflections were omitted during the absorption correction.

Alert level G

PLAT002_ALERT_2_G Number of Distance or Angle Restraints on AtSite 8 Note

```
PLAT003_ALERT_2_G Number of Uiso or Uij Restrained non-H Atoms ...         16 Report
PLAT172_ALERT_4_G The CIF-Embedded .res File Contains DFIX Records          2 Report
PLAT178_ALERT_4_G The CIF-Embedded .res File Contains SIMU Records          1 Report
PLAT187_ALERT_4_G The CIF-Embedded .res File Contains RIGU Records          1 Report
PLAT301_ALERT_3_G Main Residue  Disorder .............(Resd  1  )       100% Note
PLAT301_ALERT_3_G Main Residue  Disorder .............(Resd  2  )       100% Note
PLAT304_ALERT_4_G Non-Integer Number of Atoms in ..... (Resd  1  )      17.54 Check
PLAT304_ALERT_4_G Non-Integer Number of Atoms in ..... (Resd  2  )       6.46 Check
PLAT860_ALERT_3_G Number of Least-Squares Restraints ............         328 Note
PLAT883_ALERT_1_G No Info/Value for _atom_sites_solution_primary .     Please Do !
PLAT912_ALERT_4_G Missing # of FCF Reflections Above STh/L=  0.600         18 Note
```

```
   0 ALERT level A = Most likely a serious problem - resolve or explain
   0 ALERT level B = A potentially serious problem, consider carefully
   2 ALERT level C = Check. Ensure it is not caused by an omission or oversight
  12 ALERT level G = General information/check it is not something unexpected

   1 ALERT type 1 CIF construction/syntax error, inconsistent or missing data
   2 ALERT type 2 Indicator that the structure model may be wrong or deficient
   5 ALERT type 3 Indicator that the structure quality may be low
   6 ALERT type 4 Improvement, methodology, query or suggestion
   0 ALERT type 5 Informative message, check
```

Datablock: azoBr_a_190

```
Bond precision:  C-C = 0.0040 A          Wavelength=1.54184

Cell:             a=3.9440(1)     b=5.8440(1)        c=24.6497(5)
                  alpha=90        beta=92.924(2)     gamma=90
Temperature:      189 K

                 Calculated                 Reported
Volume           567.41(2)                  567.40(2)
Space group      P 21/c                     P 1 21/c 1
Hall group       -P 2ybc                    -P 2ybc
Moiety formula   C12 H8 Br2 N2              C12 H8 Br2 N2
Sum formula      C12 H8 Br2 N2              C12 H8 Br2 N2
Mr               340.00                     340.02
Dx,g cm-3        1.990                      1.990
Z                2                          2
Mu (mm-1)        8.823                      8.823
F000             328.0                      328.0
F000’            325.83
h,k,lmax         4,7,31                     4,6,31
Nref             1182                       1138
Tmin,Tmax        0.140,0.392                0.150,1.000
Tmin’            0.059

Correction method= # Reported T Limits: Tmin=0.150 Tmax=1.000
AbsCorr = GAUSSIAN
```

Data completeness= 0.963   Theta(max)= 76.532

R(reflections)= 0.0267(1122)   wR2(reflections)= 0.0661(1138)

S = 1.200   Npar= 74

The following ALERTS were generated. Each ALERT has the format
test-name_ALERT_alert-type_alert-level.
Click on the hyperlinks for more details of the test.

Alert level C

PLAT906_ALERT_3_C Large K Value in the Analysis of Variance ...... 2.540 Check

Author Response: Due to diffuse scattering about Bragg peaks that was not accounted for during the integration or absorption correction.

PLAT911_ALERT_3_C Missing FCF Refl Between Thmin & STh/L= 0.600 3 Report

Author Response: These reflections were omitted during the absorption correction.

Alert level G

PLAT912_ALERT_4_G Missing # of FCF Reflections Above STh/L= 0.600 18 Note
PLAT955_ALERT_1_G Reported (CIF) and Actual (FCF) Lmax Differ by . 1 Units
PLAT961_ALERT_5_G Dataset Contains no Negative Intensities .... Please Check
PLAT978_ALERT_2_G Number C-C Bonds with Positive Residual Density. 6 Info

---

0 **ALERT level A** = Most likely a serious problem - resolve or explain
0 **ALERT level B** = A potentially serious problem, consider carefully
2 **ALERT level C** = Check. Ensure it is not caused by an omission or oversight
4 **ALERT level G** = General information/check it is not something unexpected

1 ALERT type 1 CIF construction/syntax error, inconsistent or missing data
1 ALERT type 2 Indicator that the structure model may be wrong or deficient
2 ALERT type 3 Indicator that the structure quality may be low
1 ALERT type 4 Improvement, methodology, query or suggestion
1 ALERT type 5 Informative message, check

Datablock: azoBr_a_210

Bond precision: C-C = 0.0040 A   Wavelength=1.54184

Cell:	a=3.9524(1)	b=5.8503(1)	c=24.6570(5)
	alpha=90	beta=92.925(2)	gamma=90
Temperature:	210 K		

	Calculated	Reported
Volume	569.39(2)	569.39(2)
Space group	P 21/c	P 1 21/c 1
Hall group	-P 2ybc	-P 2ybc
Moiety formula	C12 H8 Br2 N2	C12 H8 Br2 N2
Sum formula	C12 H8 Br2 N2	C12 H8 Br2 N2
Mr	340.00	340.02
Dx,g cm-3	1.983	1.983
Z	2	2
Mu (mm-1)	8.792	8.792
F000	328.0	328.0
F000'	325.83	
h,k,lmax	5,7,31	5,6,30
Nref	1196	1132
Tmin,Tmax	0.141,0.394	0.357,1.000
Tmin'	0.059	

Correction method= # Reported T Limits: Tmin=0.357 Tmax=1.000
AbsCorr = GAUSSIAN

Data completeness= 0.946 Theta(max)= 77.428

R(reflections)= 0.0285(1111) wR2(reflections)= 0.0678(1132)

S = 1.216 Npar= 74

The following ALERTS were generated. Each ALERT has the format
test-name_ALERT_alert-type_alert-level.
Click on the hyperlinks for more details of the test.

Alert level C

PLAT906_ALERT_3_C Large K Value in the Analysis of Variance ...... 2.984 Check

Author Response: Due to diffuse scattering about Bragg peaks that was not accounted for during the integration or absorption correction.

PLAT911_ALERT_3_C Missing FCF Refl Between Thmin & STh/L= 0.600 4 Report

Author Response: These reflections were omitted during the absorption correction.

Alert level G

PLAT912_ALERT_4_G Missing # of FCF Reflections Above STh/L= 0.600 33 Note
PLAT953_ALERT_1_G Reported (CIF) and Actual (FCF) Hmax Differ by . 1 Units
PLAT978_ALERT_2_G Number C-C Bonds with Positive Residual Density. 5 Info

---

0 **ALERT level A** = Most likely a serious problem - resolve or explain

0 **ALERT level B** = A potentially serious problem, consider carefully
2 **ALERT level C** = Check. Ensure it is not caused by an omission or oversight
3 **ALERT level G** = General information/check it is not something unexpected

1 ALERT type 1 CIF construction/syntax error, inconsistent or missing data
1 ALERT type 2 Indicator that the structure model may be wrong or deficient
2 ALERT type 3 Indicator that the structure quality may be low
1 ALERT type 4 Improvement, methodology, query or suggestion
0 ALERT type 5 Informative message, check

Datablock: azoBr_a_230

Bond precision: C-C = 0.0042 A Wavelength=1.54184

Cell:	a=3.9619(1)	b=5.8557(1)	c=24.6640(5)
	alpha=90	beta=92.921(2)	gamma=90
Temperature:	230 K		

	Calculated	Reported
Volume	571.45(2)	571.45(2)
Space group	P 21/c	P 1 21/c 1
Hall group	-P 2ybc	-P 2ybc
Moiety formula	C12 H8 Br2 N2	C12 H8 Br2 N2
Sum formula	C12 H8 Br2 N2	C12 H8 Br2 N2
Mr	340.00	340.02
Dx,g cm-3	1.976	1.976
Z	2	2
Mu (mm-1)	8.760	8.760
F000	328.0	328.0
F000’	325.83	
h,k,lmax	5,7,31	5,6,30
Nref	1194	1129
Tmin,Tmax	0.142,0.395	0.357,1.000
Tmin’	0.060	

Correction method= # Reported T Limits: Tmin=0.357 Tmax=1.000
AbsCorr = GAUSSIAN

Data completeness= 0.946 Theta(max)= 77.087

R(reflections)= 0.0295(1109) wR2(reflections)= 0.0688(1129)

S = 1.218 Npar= 74

The following ALERTS were generated. Each ALERT has the format
test-name_ALERT_alert-type_alert-level.
Click on the hyperlinks for more details of the test.

Alert level C

```
PLAT906_ALERT_3_C Large K Value in the Analysis of Variance ......      3.116 Check
```

Author Response: Due to diffuse scattering about Bragg peaks that was not accounted for during the integration or absorption correction.

Alert level G

```
PLAT912_ALERT_4_G Missing # of FCF Reflections Above STh/L=  0.600         38 Note
PLAT961_ALERT_5_G Dataset Contains no Negative Intensities .......     Please Check
PLAT978_ALERT_2_G Number C-C Bonds with Positive Residual Density.          5 Info
```

```
   0 ALERT level A = Most likely a serious problem - resolve or explain
   0 ALERT level B = A potentially serious problem, consider carefully
   1 ALERT level C = Check. Ensure it is not caused by an omission or oversight
   3 ALERT level G = General information/check it is not something unexpected

   0 ALERT type 1 CIF construction/syntax error, inconsistent or missing data
   1 ALERT type 2 Indicator that the structure model may be wrong or deficient
   1 ALERT type 3 Indicator that the structure quality may be low
   1 ALERT type 4 Improvement, methodology, query or suggestion
   1 ALERT type 5 Informative message, check
```

Datablock: azoBr_a_250

Bond precision: C-C = 0.0042 A Wavelength=1.54184

Cell:	a=3.9716(1)	b=5.8616(1)	c=24.6711(5)
	alpha=90	beta=92.918(2)	gamma=90
Temperature:	250 K		

	Calculated	Reported
Volume	573.60(2)	573.60(2)
Space group	P 21/c	P 1 21/c 1
Hall group	-P 2ybc	-P 2ybc
Moiety formula	C12 H8 Br2 N2	C12 H8 Br2 N2
Sum formula	C12 H8 Br2 N2	C12 H8 Br2 N2
Mr	340.00	340.02
Dx,g cm-3	1.969	1.969
Z	2	2
Mu (mm-1)	8.727	8.727
F000	328.0	328.0
F000’	325.83	
h,k,lmax	5,7,31	5,6,31
Nref	1203	1148
Tmin,Tmax	0.144,0.397	0.357,1.000
Tmin’	0.060	

```
Correction method= # Reported T Limits: Tmin=0.357 Tmax=1.000
AbsCorr = GAUSSIAN

Data completeness= 0.954          Theta(max)= 77.497

R(reflections)= 0.0306( 1130)     wR2(reflections)= 0.0701( 1148)

S = 1.213                  Npar= 74
```

The following ALERTS were generated. Each ALERT has the format
test-name_ALERT_alert-type_alert-level.
Click on the hyperlinks for more details of the test.

Alert level C

```
PLAT906_ALERT_3_C Large K Value in the Analysis of Variance ......      3.314 Check
```

Author Response: Due to diffuse scattering about Bragg peaks that was not accounted for during the integration or absorption correction.

```
PLAT911_ALERT_3_C Missing FCF Refl Between Thmin & STh/L=    0.600          2 Report
```

Author Response: These reflections were omitted during the absorption correction.

Alert level G

```
PLAT912_ALERT_4_G Missing # of FCF Reflections Above STh/L=  0.600         27 Note
PLAT933_ALERT_2_G Number of OMIT Records in Embedded .res File ...          1 Note
PLAT955_ALERT_1_G Reported (CIF) and Actual (FCF) Lmax Differ by .          1 Units
PLAT978_ALERT_2_G Number C-C Bonds with Positive Residual Density.          4 Info
```

```
   0 ALERT level A = Most likely a serious problem - resolve or explain
   0 ALERT level B = A potentially serious problem, consider carefully
   2 ALERT level C = Check. Ensure it is not caused by an omission or oversight
   4 ALERT level G = General information/check it is not something unexpected

   1 ALERT type 1 CIF construction/syntax error, inconsistent or missing data
   2 ALERT type 2 Indicator that the structure model may be wrong or deficient
   2 ALERT type 3 Indicator that the structure quality may be low
   1 ALERT type 4 Improvement, methodology, query or suggestion
   0 ALERT type 5 Informative message, check
```

Datablock: azoBr_a_270

```
Bond precision:  C-C = 0.0047 A          Wavelength=1.54184
```

Cell:	a=3.9815(1)	b=5.8686(1)	c=24.6795(5)
	alpha=90	beta=92.925(2)	gamma=90
Temperature:	270 K		

	Calculated	Reported
Volume	575.91(2)	575.91(2)
Space group	P 21/c	P 1 21/c 1
Hall group	-P 2ybc	-P 2ybc
Moiety formula	C12 H8 Br2 N2	C12 H8 Br2 N2
Sum formula	C12 H8 Br2 N2	C12 H8 Br2 N2
Mr	340.00	340.02
Dx,g cm-3	1.961	1.961
Z	2	2
Mu (mm-1)	8.692	8.692
F000	328.0	328.0
F000’	325.83	
h,k,lmax	5,7,31	5,7,30
Nref	1206	1156
Tmin,Tmax	0.145,0.398	0.358,1.000
Tmin’	0.061	

Correction method= # Reported T Limits: Tmin=0.358 Tmax=1.000
AbsCorr = GAUSSIAN

Data completeness= 0.959 Theta(max)= 77.307

R(reflections)= 0.0310(1140) wR2(reflections)= 0.0724(1156)

S = 1.250 Npar= 74

The following ALERTS were generated. Each ALERT has the format
test-name_ALERT_alert-type_alert-level.
Click on the hyperlinks for more details of the test.

Alert level C

PLAT906_ALERT_3_C Large K Value in the Analysis of Variance ...... 3.594 Check

Author Response: Due to diffuse scattering about Bragg peaks that was not accounted for during the integration or absorption correction.

PLAT911_ALERT_3_C Missing FCF Refl Between Thmin & STh/L= 0.600 2 Report

Author Response: These reflections were omitted during the absorption correction.

Alert level G

PLAT912_ALERT_4_G Missing # of FCF Reflections Above STh/L= 0.600 49 Note

```
PLAT961_ALERT_5_G Dataset Contains no Negative Intensities .......     Please Check
PLAT978_ALERT_2_G Number C-C Bonds with Positive Residual Density.          5 Info
```

```
   0 ALERT level A = Most likely a serious problem - resolve or explain
   0 ALERT level B = A potentially serious problem, consider carefully
   2 ALERT level C = Check. Ensure it is not caused by an omission or oversight
   3 ALERT level G = General information/check it is not something unexpected

   0 ALERT type 1 CIF construction/syntax error, inconsistent or missing data
   1 ALERT type 2 Indicator that the structure model may be wrong or deficient
   2 ALERT type 3 Indicator that the structure quality may be low
   1 ALERT type 4 Improvement, methodology, query or suggestion
   1 ALERT type 5 Informative message, check
```

Datablock: azoBr_a_290

Bond precision: C-C = 0.0050 A Wavelength=1.54184

Cell:	a=3.9921(1)	b=5.8744(1)	c=24.6890(5)
	alpha=90	beta=92.925(2)	gamma=90
Temperature:	290 K		

	Calculated	Reported
Volume	578.23(2)	578.23(2)
Space group	P 21/c	P 1 21/c 1
Hall group	-P 2ybc	-P 2ybc
Moiety formula	C12 H8 Br2 N2	C12 H8 Br2 N2
Sum formula	C12 H8 Br2 N2	C12 H8 Br2 N2
Mr	340.00	340.02
Dx,g cm-3	1.953	1.953
Z	2	2
Mu (mm-1)	8.657	8.657
F000	328.0	328.0
F000’	325.83	
h,k,lmax	5,7,31	5,7,30
Nref	1212	1153
Tmin,Tmax	0.146,0.399	0.358,1.000
Tmin’	0.062	

Correction method= # Reported T Limits: Tmin=0.358 Tmax=1.000
AbsCorr = GAUSSIAN

Data completeness= 0.951 Theta(max)= 77.148

R(reflections)= 0.0333(1130) wR2(reflections)= 0.0770(1153)

S = 1.217 Npar= 74

The following ALERTS were generated. Each ALERT has the format
test-name_ALERT_alert-type_alert-level.
Click on the hyperlinks for more details of the test.

Alert level C

PLAT906_ALERT_3_C Large K Value in the Analysis of Variance ...... 3.737 Check

Author Response: Due to diffuse scattering about Bragg peaks that was not accounted for during the integration or absorption correction.

PLAT911_ALERT_3_C Missing FCF Refl Between Thmin & STh/L= 0.600 3 Report

Author Response: These reflections were omitted during the absorption correction.

Alert level G

PLAT912_ALERT_4_G Missing # of FCF Reflections Above STh/L= 0.600 56 Note
PLAT978_ALERT_2_G Number C-C Bonds with Positive Residual Density. 3 Info

0 **ALERT level A** = Most likely a serious problem - resolve or explain
0 **ALERT level B** = A potentially serious problem, consider carefully
2 **ALERT level C** = Check. Ensure it is not caused by an omission or oversight
2 **ALERT level G** = General information/check it is not something unexpected

0 ALERT type 1 CIF construction/syntax error, inconsistent or missing data
1 ALERT type 2 Indicator that the structure model may be wrong or deficient
2 ALERT type 3 Indicator that the structure quality may be low
1 ALERT type 4 Improvement, methodology, query or suggestion
0 ALERT type 5 Informative message, check

It is advisable to attempt to resolve as many as possible of the alerts in all categories. Often the minor alerts point to easily fixed oversights, errors and omissions in your CIF or refinement strategy, so attention to these fine details can be worthwhile. In order to resolve some of the more serious problems it may be necessary to carry out additional measurements or structure refinements. However, the purpose of your study may justify the reported deviations and the more serious of these should normally be commented upon in the discussion or experimental section of a paper or in the "special_details" fields of the CIF. checkCIF was carefully designed to identify outliers and unusual parameters, but every test has its limitations and alerts that are not important in a particular case may appear. Conversely, the absence of alerts does not guarantee there are no aspects of the results needing attention. It is up to the individual to critically assess their own results and, if necessary, seek expert advice.

Publication of your CIF in IUCr journals

A basic structural check has been run on your CIF. These basic checks will be run on all CIFs submitted for publication in IUCr journals (*Acta Crystallographica*, *Journal of Applied Crystallography*, *Journal of Synchrotron Radiation*); however, if you intend to submit to *Acta Crystallographica Section C* or *E* or *IUCrData*, you should make sure that full publication checks are run on the final version of your CIF prior to submission.

Publication of your CIF in other journals

Please refer to the *Notes for Authors* of the relevant journal for any special instructions relating to CIF submission.

PLATON version of 03/06/2021; check.def file version of 02/06/2021

Datablock azoI_190 - ellipsoid plot

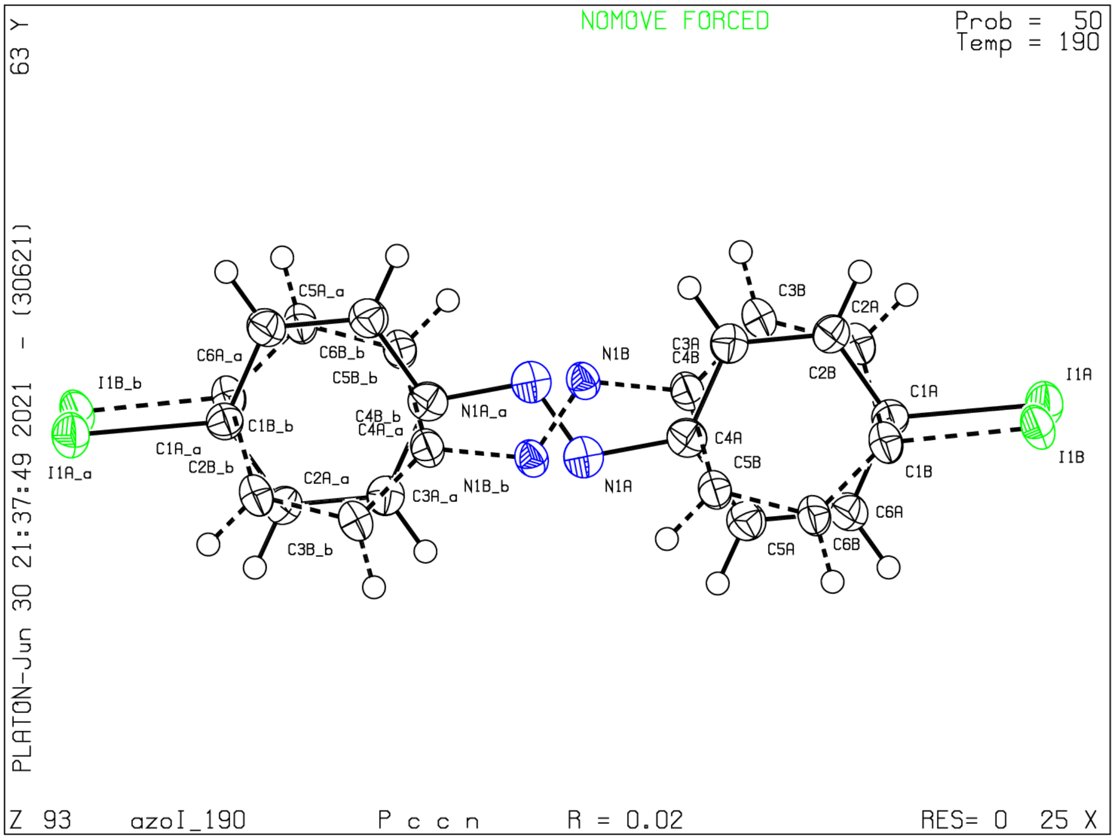

Datablock azoI_210 - ellipsoid plot

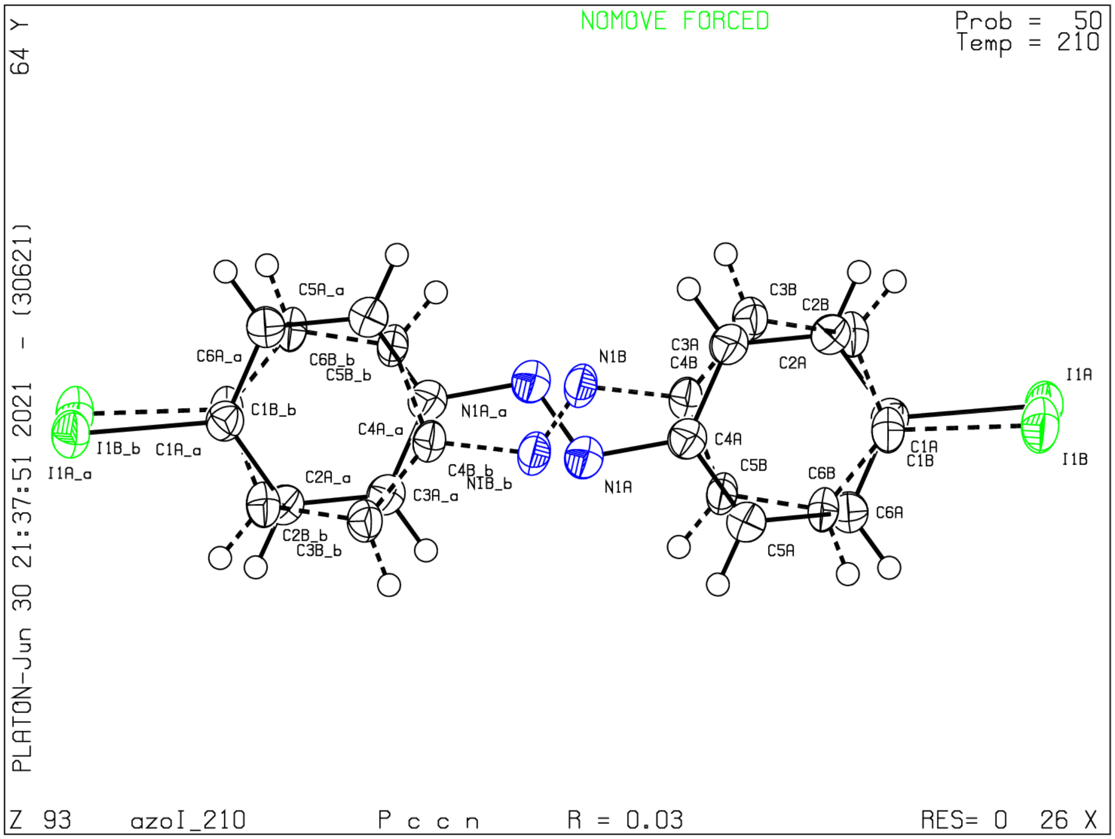

Datablock azoI_230 - ellipsoid plot

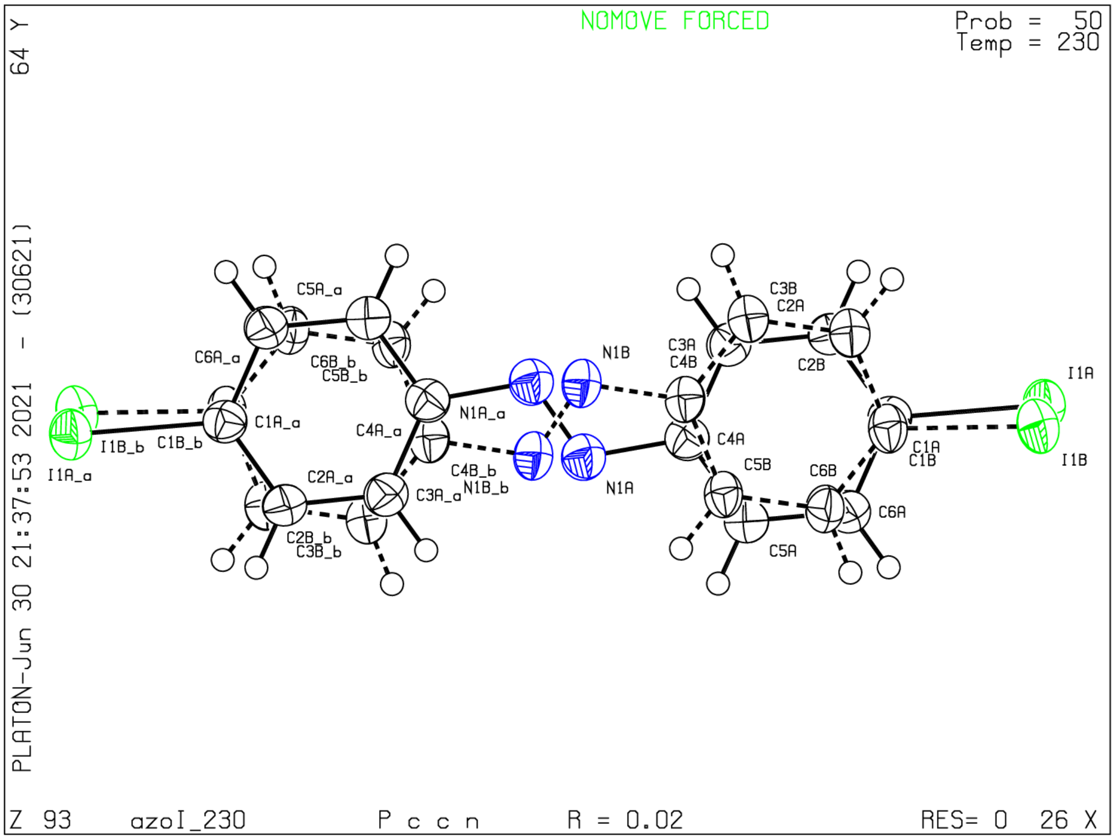

Datablock azoI_250 - ellipsoid plot

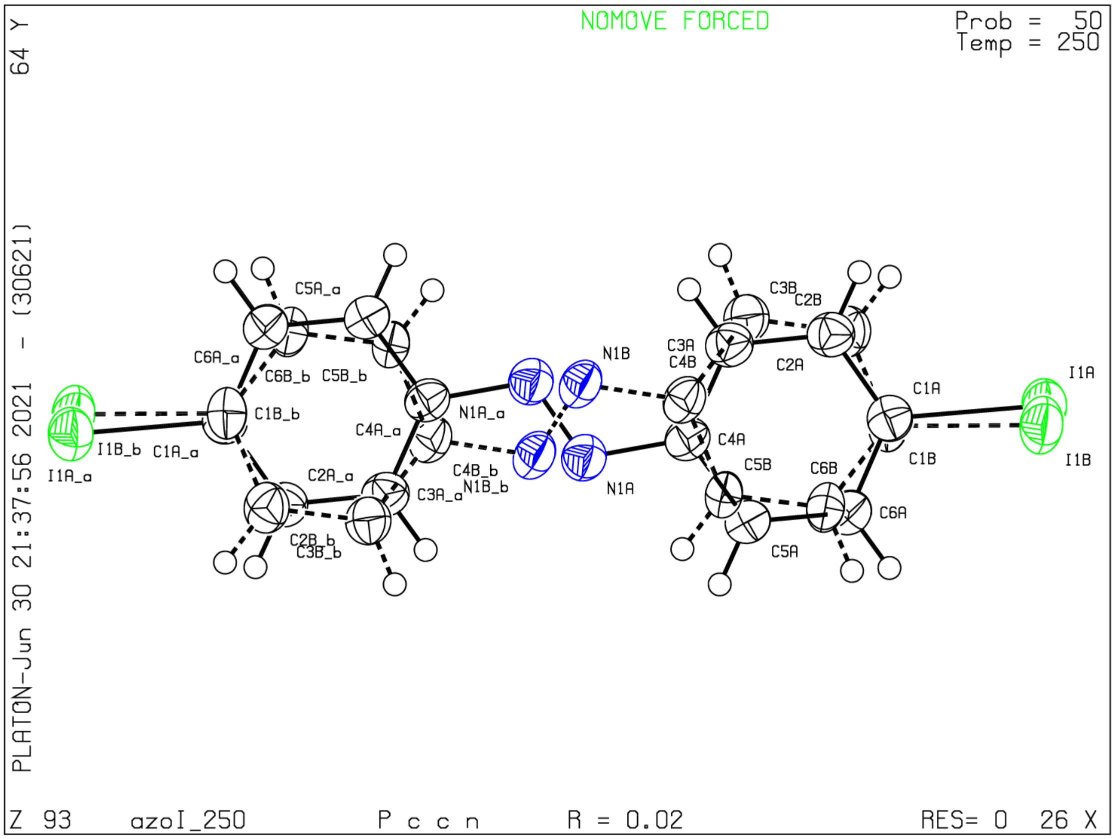

Datablock azoI_270 - ellipsoid plot

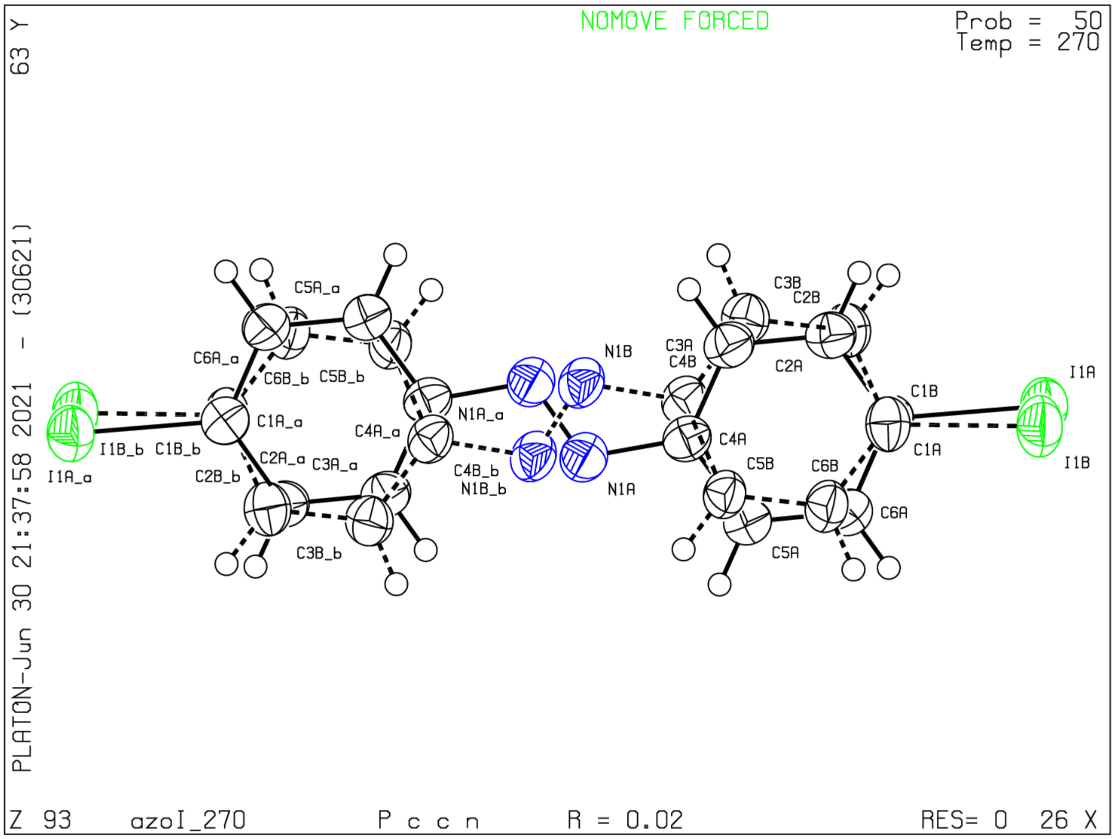

Datablock azoI_290 - ellipsoid plot

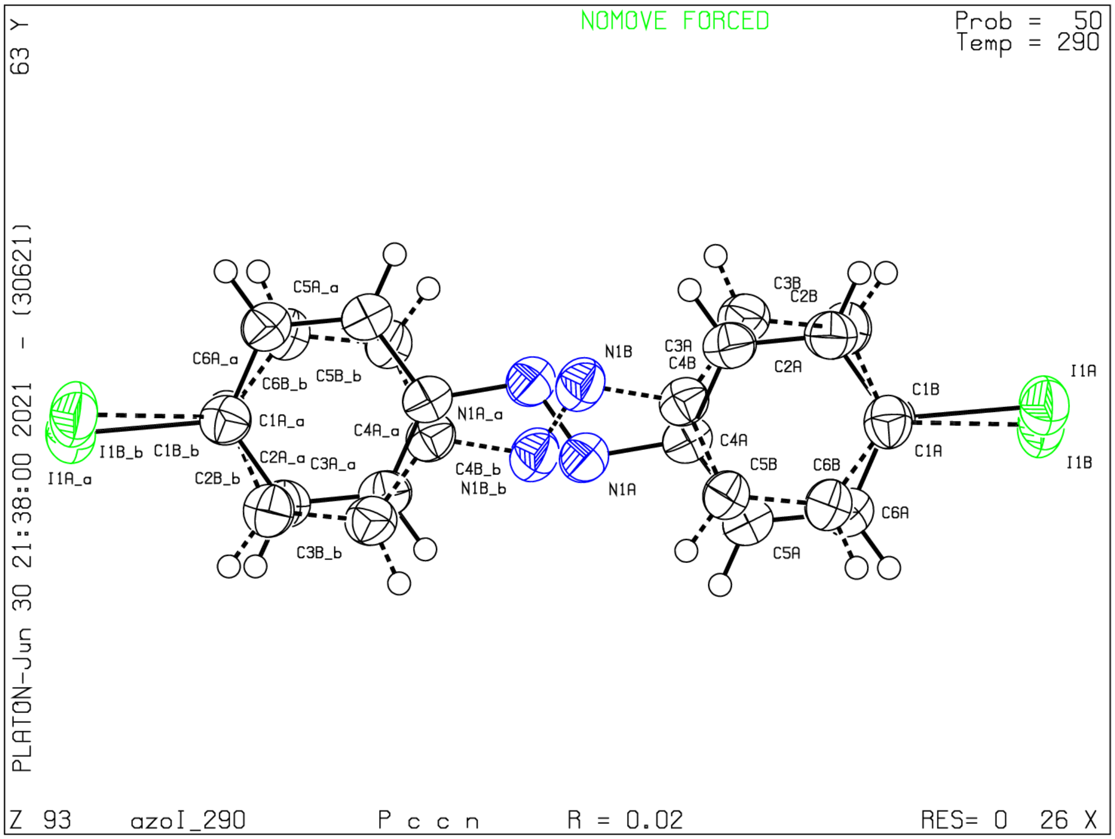

Datablock azoBr_a_190 - ellipsoid plot

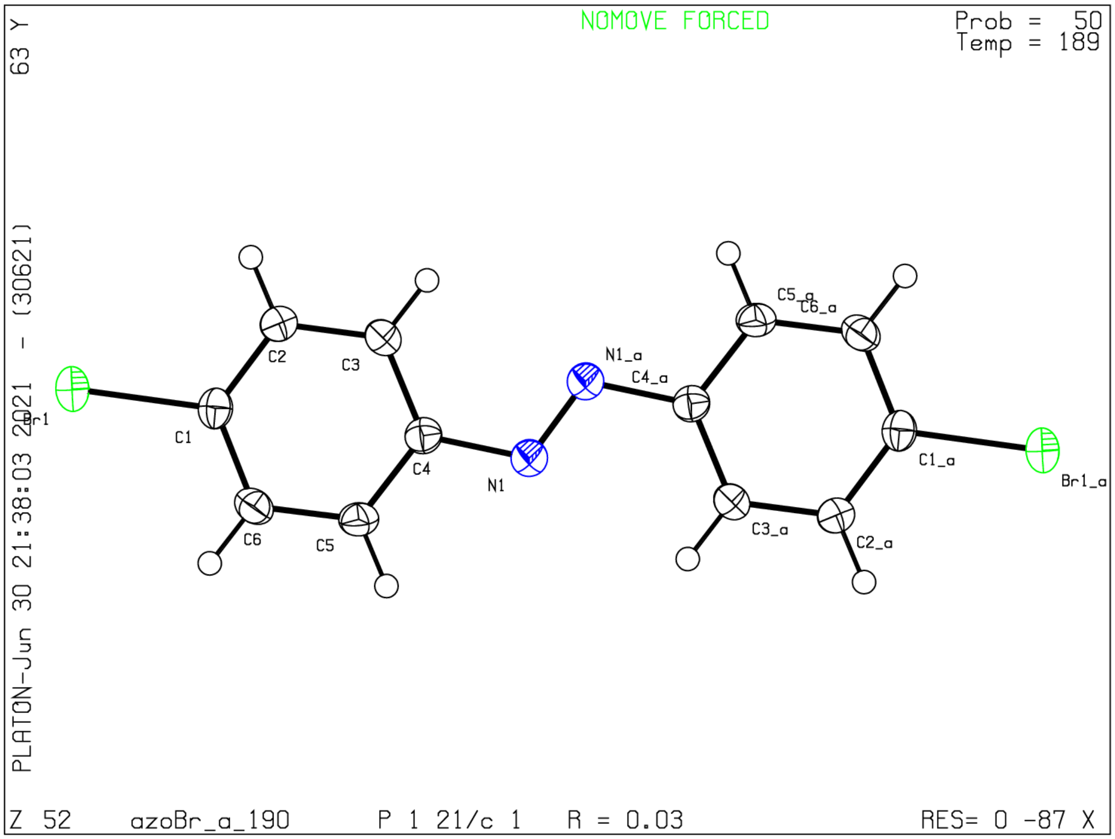

Datablock azoBr_a_210 - ellipsoid plot

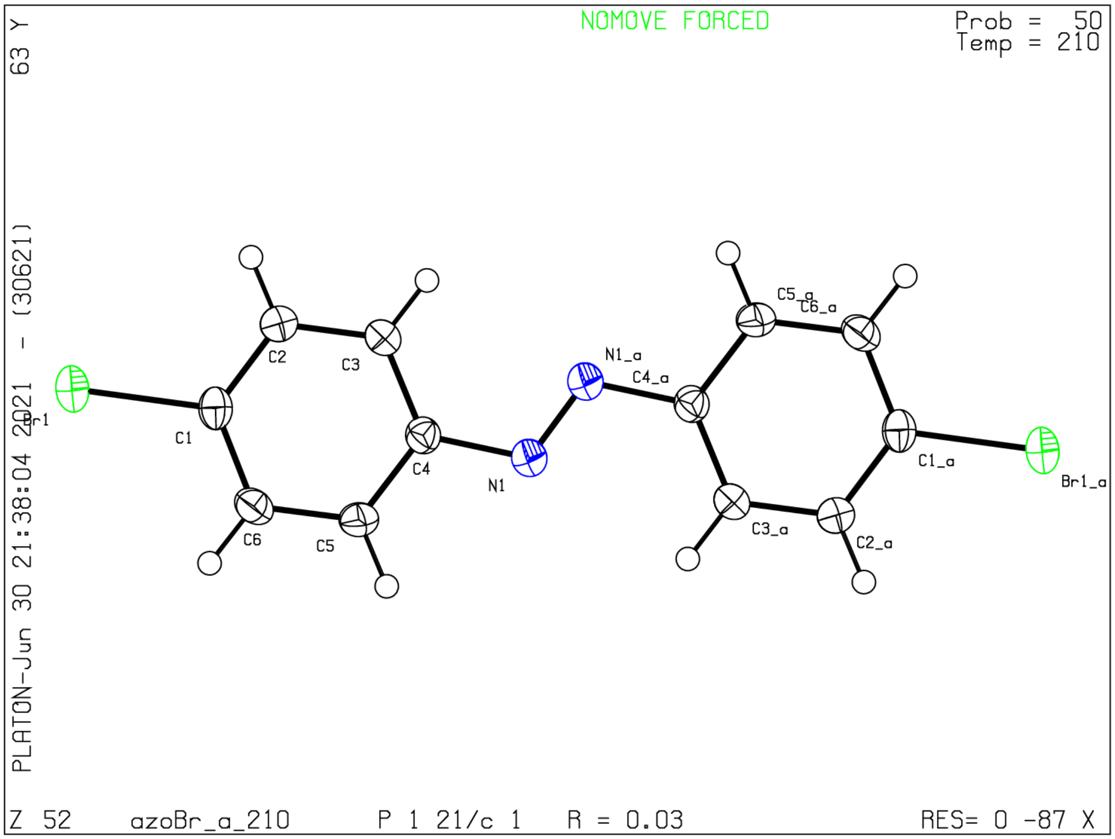

Datablock azoBr_a_230 - ellipsoid plot

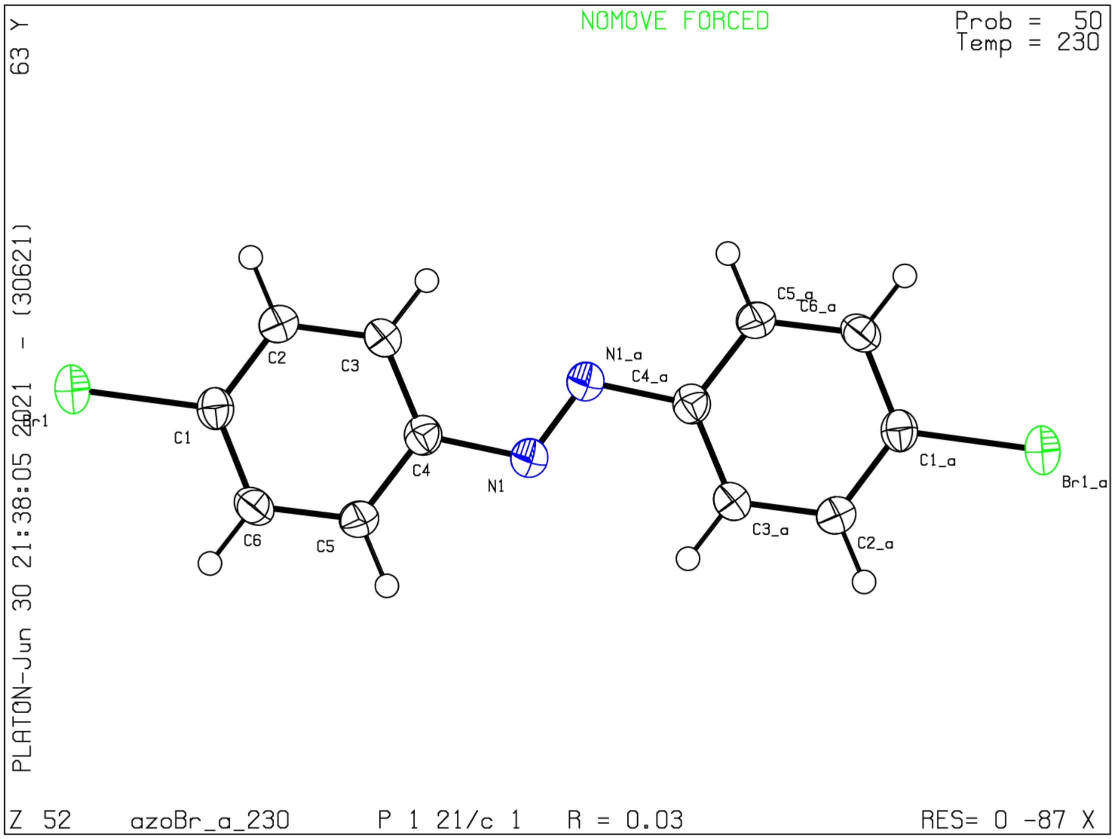

Datablock azoBr_a_250 - ellipsoid plot

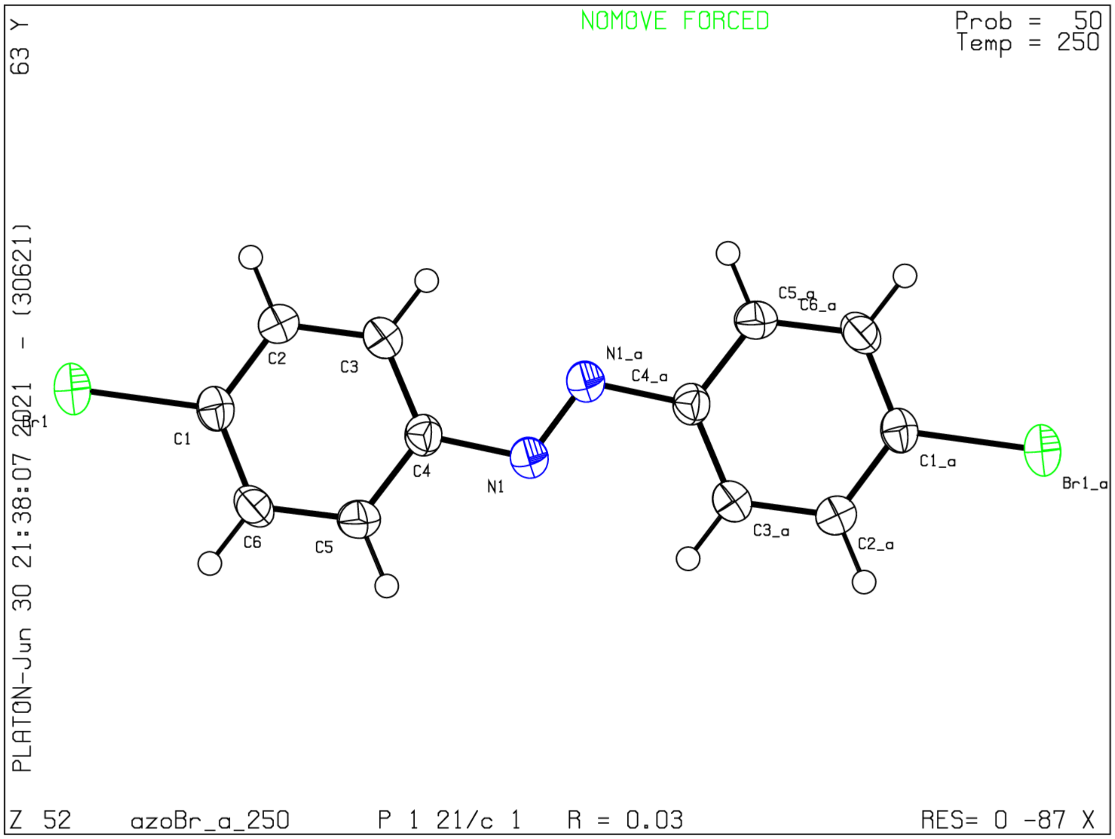

Datablock azoBr_a_270 - ellipsoid plot

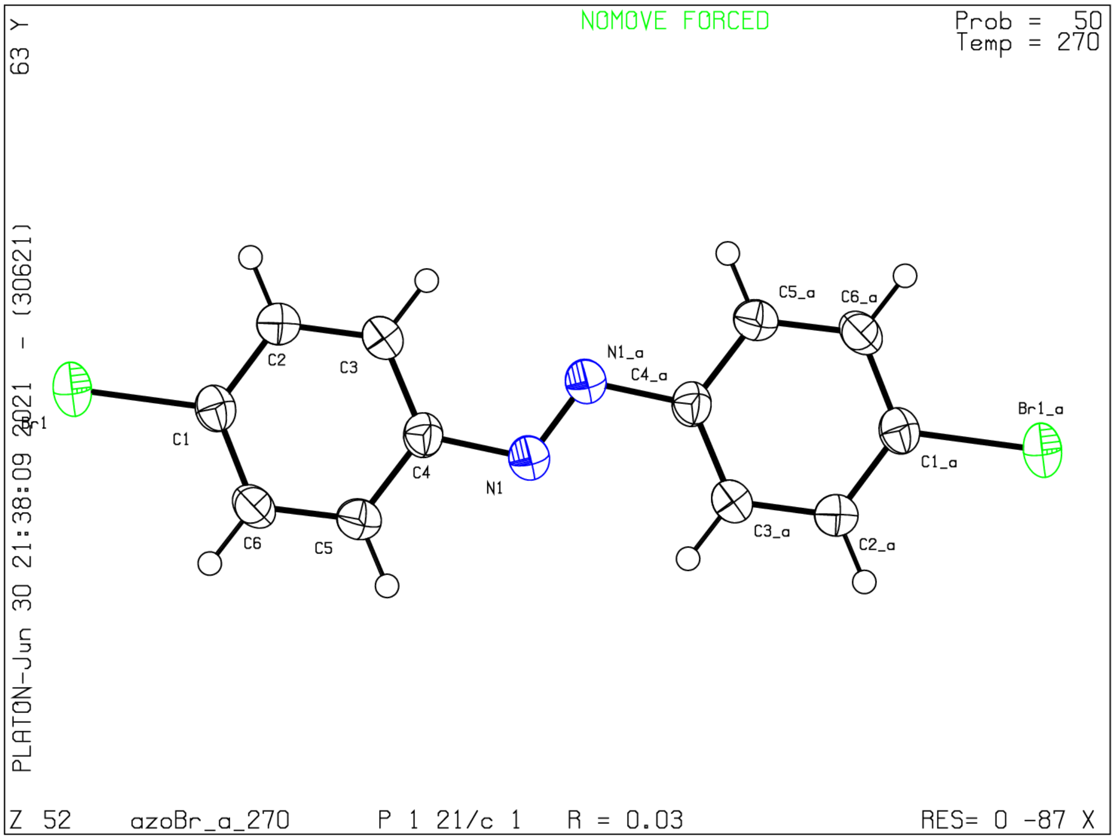

Datablock azoBr_a_290 - ellipsoid plot

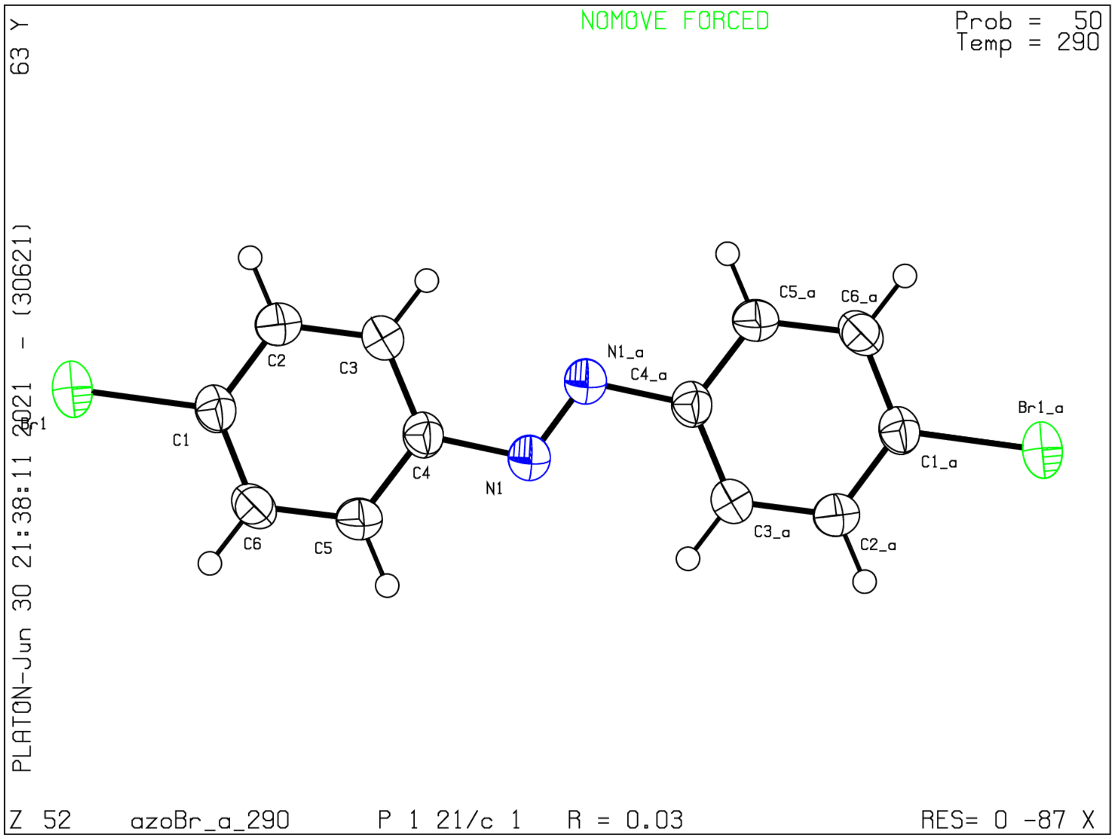

Supplement: Supplementary file 3 [file azo-I + azo-Br a.pdf › azo-I + azo-Br a.pdf]
