# Supplementary material for: Differences in thermal expansion and motion ability for herringbone and face-to-face π-stacked solids
Source: IUCrJ. 2021 Nov 3;9(Pt 1):31–42. doi: 10.1107/S2052252521009593 (PMC8733877; doi:10.1107/S2052252521009593)

checkCIF/PLATON report

Structure factors have been supplied for datablock(s) diazoBr_190K, diazoBr_210K, diazoBr_230K, diazoBr_250K, diazoBr_270K, diazoBr_290K, diazoI_190, diazoI_210, diazoI_230, diazoI_250, diazoI_270, diazoI_290

THIS REPORT IS FOR GUIDANCE ONLY. IF USED AS PART OF A REVIEW PROCEDURE FOR PUBLICATION, IT SHOULD NOT REPLACE THE EXPERTISE OF AN EXPERIENCED CRYSTALLOGRAPHIC REFEREE.

No syntax errors found. CIF dictionary Interpreting this report

Datablock: diazoI_190

```
Bond precision:  C-C = 0.0068 A          Wavelength=1.54184

Cell:            a=7.2748(1)     b=40.2019(5)       c=5.9306(1)
                 alpha=90        beta=90.905(1)     gamma=90
Temperature:     190 K

                Calculated                 Reported
Volume          1734.25(4)                 1734.25(4)
Space group     P 21/c                     P 1 21/c 1
Hall group      -P 2ybc                    -P 2ybc
Moiety formula  C18 H12 I2 N4              2(C9 H6 I N2)
Sum formula     C18 H12 I2 N4              C18 H12 I2 N4
Mr              538.12                     538.12
Dx,g cm-3       2.061                      2.061
Z               4                          4
Mu (mm-1)       28.535                     28.535
F000            1016.0                     1016.0
F000'           1016.58
h,k,lmax        9,50,7                     8,50,7
Nref            3690                       3580
Tmin,Tmax       0.207,0.710                0.256,0.765
Tmin'           0.133

Correction method= # Reported T Limits: Tmin=0.256 Tmax=0.765
AbsCorr = GAUSSIAN

Data completeness= 0.970          Theta(max)= 77.523

R(reflections)= 0.0400( 3271)     wR2(reflections)= 0.1065( 3580)

S = 1.103                   Npar= 238
```

The following ALERTS were generated. Each ALERT has the format
test-name_ALERT_alert-type_alert-level.
Click on the hyperlinks for more details of the test.

Alert level B

PLAT971_ALERT_2_B Check Calcd Resid. Dens. 1.04A From I1 2.67 eA-3

Author Response: These are spurious peaks most likely from a minor twin component (<5%) that was present in the sample. Attempts to integrate the data with both components did not improve the residual density issue in this structure.

Alert level C

PLAT094_ALERT_2_C Ratio of Maximum / Minimum Residual Density .... 2.78 Report

Author Response: Due to the spurious peaks about iodine atoms.

PLAT906_ALERT_3_C Large K Value in the Analysis of Variance ...... 2.910 Check

Author Response: This was due to diffuse scattering about Bragg peaks that was not account for during the integration and absorption correction.

PLAT911_ALERT_3_C Missing FCF Refl Between Thmin & STh/L= 0.600 17 Report

Author Response: These reflections were omitted during the absorption correction.

PLAT971_ALERT_2_C Check Calcd Resid. Dens. 1.05A From I2 2.14 eA-3

Author Response: These are spurious peaks most likely from a minor twin component (<5%) that was present in the sample. Attempts to integrate the data with both components did not improve the residual density issue in this structure.

PLAT971_ALERT_2_C Check Calcd Resid. Dens. 1.04A From I2 1.76 eA-3

Author Response: These are spurious peaks most likely from a minor twin component (<5%) that was present in the sample. Attempts to integrate the data with both components did not improve the residual density issue in this structure.

Alert level G

PLAT002_ALERT_2_G Number of Distance or Angle Restraints on AtSite 6 Note
PLAT003_ALERT_2_G Number of Uiso or Uij Restrained non-H Atoms ... 4 Report
PLAT012_ALERT_1_G N.O.K. _shelx_res_checksum Found in CIF ...... Please Check
PLAT013_ALERT_1_G N.O.K. _shelx_hkl_checksum Found in CIF ...... Please Check
PLAT042_ALERT_1_G Calc. and Reported Moiety Formula Strings Differ Please Check

```
PLAT172_ALERT_4_G The CIF-Embedded .res File Contains DFIX Records          2 Report
PLAT178_ALERT_4_G The CIF-Embedded .res File Contains SIMU Records          1 Report
PLAT301_ALERT_3_G Main Residue  Disorder .............(Resd  1  )        17% Note
PLAT860_ALERT_3_G Number of Least-Squares Restraints .............         42 Note
PLAT910_ALERT_3_G Missing # of FCF Reflection(s) Below Theta(Min).          1 Note
PLAT912_ALERT_4_G Missing # of FCF Reflections Above STh/L=  0.600         66 Note
PLAT933_ALERT_2_G Number of OMIT Records in Embedded .res File ...          1 Note
PLAT978_ALERT_2_G Number C-C Bonds with Positive Residual Density.          0 Info
```

---

0 **ALERT level A** = Most likely a serious problem - resolve or explain
1 **ALERT level B** = A potentially serious problem, consider carefully
5 **ALERT level C** = Check. Ensure it is not caused by an omission or oversight
13 **ALERT level G** = General information/check it is not something unexpected

3 ALERT type 1 CIF construction/syntax error, inconsistent or missing data
8 ALERT type 2 Indicator that the structure model may be wrong or deficient
5 ALERT type 3 Indicator that the structure quality may be low
3 ALERT type 4 Improvement, methodology, query or suggestion
0 ALERT type 5 Informative message, check

Datablock: diazoI_210

Bond precision: C-C = 0.0073 A Wavelength=1.54184

Cell:	a=7.2891(1)	b=40.2211(5)	c=5.9383(1)
	alpha=90	beta=90.858(2)	gamma=90
Temperature:	210 K		

	Calculated	Reported
Volume	1740.77(4)	1740.77(4)
Space group	P 21/c	P 1 21/c 1
Hall group	-P 2ybc	-P 2ybc
Moiety formula	C18 H12 I2 N4	2(C9 H6 I N2)
Sum formula	C18 H12 I2 N4	C18 H12 I2 N4
Mr	538.12	538.12
Dx,g cm-3	2.053	2.053
Z	4	4
Mu (mm-1)	28.428	28.428
F000	1016.0	1016.0
F000’	1016.58	
h,k,lmax	9,50,7	8,50,7
Nref	3706	3580
Tmin,Tmax	0.208,0.711	0.258,0.767
Tmin’	0.133	

Correction method= # Reported T Limits: Tmin=0.258 Tmax=0.767
AbsCorr = GAUSSIAN

Data completeness= 0.966 Theta(max)= 77.531

```
R(reflections)= 0.0442( 3204)     wR2(reflections)= 0.1168( 3580)

S = 1.090                    Npar= 238
```

The following ALERTS were generated. Each ALERT has the format
test-name_ALERT_alert-type_alert-level.
Click on the hyperlinks for more details of the test.

Alert level C

```
PLAT906_ALERT_3_C Large K Value in the Analysis of Variance ......      2.722 Check
```

Author Response: This was due to diffuse scattering about Bragg peaks that was not account for during the integration and absorption correction.

```
PLAT911_ALERT_3_C Missing FCF Refl Between Thmin & STh/L=    0.600         15 Report
```

Author Response: These reflections were omitted during the absorption correction.

```
PLAT971_ALERT_2_C Check Calcd Resid. Dens.  1.19A   From I1              2.38 eA-3
```

Author Response: These are spurious peaks most likely from a minor twin component (<5%) that was present in the sample. Attempts to integrate the data with both components did not improve the residual density issue in this structure.

```
PLAT971_ALERT_2_C Check Calcd Resid. Dens.  1.23A   From I2              1.68 eA-3
```

Author Response: These are spurious peaks most likely from a minor twin component (<5%) that was present in the sample. Attempts to integrate the data with both components did not improve the residual density issue in this structure.

```
PLAT971_ALERT_2_C Check Calcd Resid. Dens.  1.11A   From I2              1.57 eA-3
```

Author Response: These are spurious peaks most likely from a minor twin component (<5%) that was present in the sample. Attempts to integrate the data with both components did not improve the residual density issue in this structure.

Alert level G

```
PLAT002_ALERT_2_G Number of Distance or Angle Restraints on AtSite         6 Note
PLAT003_ALERT_2_G Number of Uiso or Uij Restrained non-H Atoms ...         4 Report
PLAT012_ALERT_1_G N.O.K.   _shelx_res_checksum Found in CIF ......     Please Check
PLAT013_ALERT_1_G N.O.K.   _shelx_hkl_checksum Found in CIF ......     Please Check
PLAT042_ALERT_1_G Calc. and Reported Moiety Formula Strings Differ     Please Check
PLAT172_ALERT_4_G The CIF-Embedded .res File Contains DFIX Records         2 Report
PLAT178_ALERT_4_G The CIF-Embedded .res File Contains SIMU Records         1 Report
```

```
PLAT301_ALERT_3_G Main Residue  Disorder ..............(Resd  1  )        17% Note
PLAT860_ALERT_3_G Number of Least-Squares Restraints .............         42 Note
PLAT912_ALERT_4_G Missing # of FCF Reflections Above STh/L=  0.600         83 Note
PLAT933_ALERT_2_G Number of OMIT Records in Embedded .res File ...          1 Note
PLAT978_ALERT_2_G Number C-C Bonds with Positive Residual Density.          0 Info
```

---

   0 **ALERT level A** = Most likely a serious problem - resolve or explain
   0 **ALERT level B** = A potentially serious problem, consider carefully
   5 **ALERT level C** = Check. Ensure it is not caused by an omission or oversight
  12 **ALERT level G** = General information/check it is not something unexpected

   3 ALERT type 1 CIF construction/syntax error, inconsistent or missing data
   7 ALERT type 2 Indicator that the structure model may be wrong or deficient
   4 ALERT type 3 Indicator that the structure quality may be low
   3 ALERT type 4 Improvement, methodology, query or suggestion
   0 ALERT type 5 Informative message, check

Datablock: diazoI_230

Bond precision: C-C = 0.0081 A   Wavelength=1.54184

Cell:	a=7.3039(1)	b=40.2474(5)	c=5.9459(1)
	alpha=90	beta=90.785(2)	gamma=90
Temperature:	230 K		

	Calculated	Reported
Volume	1747.71(4)	1747.71(4)
Space group	P 21/c	P 1 21/c 1
Hall group	-P 2ybc	-P 2ybc
Moiety formula	C18 H12 I2 N4	2(C9 H6 I N2)
Sum formula	C18 H12 I2 N4	C18 H12 I2 N4
Mr	538.12	538.12
Dx,g cm-3	2.045	2.045
Z	4	4
Mu (mm-1)	28.315	28.315
F000	1016.0	1016.0
F000’	1016.58	
h,k,lmax	9,51,7	8,51,7
Nref	3719	3550
Tmin,Tmax	0.210,0.712	0.270,0.768
Tmin’	0.134	

Correction method= # Reported T Limits: Tmin=0.270 Tmax=0.768
AbsCorr = GAUSSIAN

Data completeness= 0.955   Theta(max)= 77.702

R(reflections)= 0.0472(3019)   wR2(reflections)= 0.1255(3550)

S = 1.077 Npar= 280

The following ALERTS were generated. Each ALERT has the format
test-name_ALERT_alert-type_alert-level.
Click on the hyperlinks for more details of the test.

Alert level C

PLAT241_ALERT_2_C High 'MainMol' Ueq as Compared to Neighbors of I2 Check

Author Response: Due to the spurious peaks about iodine atoms.

PLAT342_ALERT_3_C Low Bond Precision on C-C Bonds .... 0.00808 Ang.

Author Response: Due to amount of modeled disorder within the structure.

PLAT906_ALERT_3_C Large K Value in the Analysis of Variance ...... 3.019 Check

Author Response: This was due to diffuse scattering about Bragg peaks that was not account for during the integration and absorption correction.

PLAT911_ALERT_3_C Missing FCF Refl Between Thmin & STh/L= 0.600 28 Report

Author Response: These reflections were omitted during the absorption correction.

PLAT971_ALERT_2_C Check Calcd Resid. Dens. 1.15A From I1 2.22 eA-3

Author Response: These are spurious peaks most likely from a minor twin component (<5%) that was present in the sample. Attempts to integrate the data with both components did not improve the residual density issue in this structure.

PLAT971_ALERT_2_C Check Calcd Resid. Dens. 0.81A From I2 1.56 eA-3

Author Response: These are spurious peaks most likely from a minor twin component (<5%) that was present in the sample. Attempts to integrate the data with both components did not improve the residual density issue in this structure.

PLAT971_ALERT_2_C Check Calcd Resid. Dens. 1.14A From I2 1.55 eA-3

Author Response: These are spurious peaks most likely from a minor twin component (<5%) that was present in the sample. Attempts to integrate the data with both components did not improve the residual density issue in this structure.

Alert level G

```
PLAT002_ALERT_2_G Number of Distance or Angle Restraints on AtSite          7 Note
PLAT003_ALERT_2_G Number of Uiso or Uij Restrained non-H Atoms ...         16 Report
PLAT012_ALERT_1_G N.O.K.   _shelx_res_checksum Found in CIF ......     Please Check
PLAT013_ALERT_1_G N.O.K.   _shelx_hkl_checksum Found in CIF ......     Please Check
PLAT042_ALERT_1_G Calc. and Reported Moiety Formula Strings Differ     Please Check
PLAT083_ALERT_2_G SHELXL Second Parameter in WGHT  Unusually Large       5.45 Why ?
PLAT172_ALERT_4_G The CIF-Embedded .res File Contains DFIX Records          2 Report
PLAT178_ALERT_4_G The CIF-Embedded .res File Contains SIMU Records          1 Report
PLAT301_ALERT_3_G Main Residue  Disorder ..............(Resd  1  )        67% Note
PLAT811_ALERT_5_G No ADDSYM Analysis: Too Many Excluded Atoms ....          ! Info
PLAT860_ALERT_3_G Number of Least-Squares Restraints .............        252 Note
PLAT912_ALERT_4_G Missing # of FCF Reflections Above STh/L=  0.600        111 Note
PLAT933_ALERT_2_G Number of OMIT Records in Embedded .res File ...          1 Note
PLAT954_ALERT_1_G Reported (CIF) and Actual (FCF) Kmax Differ by .          1 Units
PLAT978_ALERT_2_G Number C-C Bonds with Positive Residual Density.          0 Info
```

---

0 **ALERT level A** = Most likely a serious problem - resolve or explain
0 **ALERT level B** = A potentially serious problem, consider carefully
7 **ALERT level C** = Check. Ensure it is not caused by an omission or oversight
15 **ALERT level G** = General information/check it is not something unexpected

4 ALERT type 1 CIF construction/syntax error, inconsistent or missing data
9 ALERT type 2 Indicator that the structure model may be wrong or deficient
5 ALERT type 3 Indicator that the structure quality may be low
3 ALERT type 4 Improvement, methodology, query or suggestion
1 ALERT type 5 Informative message, check

Datablock: diazoI_250

Bond precision: C-C = 0.0114 A   Wavelength=1.54184

Cell:	a=7.31514(13)	b=40.2986(5)	c=5.95370(13)
	alpha=90	beta=90.6425(19)	gamma=90
Temperature:	250 K		

	Calculated	Reported
Volume	1754.98(5)	1754.98(5)
Space group	P 21/c	P 1 21/c 1
Hall group	-P 2ybc	-P 2ybc
Moiety formula	C18 H12 I2 N4	2(C9 H6 I N2)
Sum formula	C18 H12 I2 N4	C18 H12 I2 N4
Mr	538.12	538.12
Dx,g cm-3	2.037	2.037
Z	4	4
Mu (mm-1)	28.198	28.198
F000	1016.0	1016.0
F000’	1016.58	
h,k,lmax	8,48,7	8,48,7
Nref	3132	3111
Tmin,Tmax	0.212,0.713	0.274,0.762
Tmin’	0.135	

Correction method= # Reported T Limits: Tmin=0.274 Tmax=0.762
AbsCorr = GAUSSIAN

Data completeness= 0.993 Theta(max)= 67.077

R(reflections)= 0.0537(2603) wR2(reflections)= 0.1476(3111)

S = 1.062 Npar= 281

The following ALERTS were generated. Each ALERT has the format
test-name_ALERT_alert-type_alert-level.
Click on the hyperlinks for more details of the test.

Alert level B

PLAT971_ALERT_2_B Check Calcd Resid. Dens. 1.11A From I1 2.80 eA-3

Author Response: These are spurious peaks most likely from a minor twin component (<5%) that was present in the sample. Attempts to integrate the data with both components did not improve the residual density issue in this structure.

Alert level C

PLAT094_ALERT_2_C Ratio of Maximum / Minimum Residual Density .... 2.16 Report

Author Response: Due to the spurious peaks about iodine atoms.

PLAT241_ALERT_2_C High ’MainMol’ Ueq as Compared to Neighbors of I2 Check

Author Response: Due to the spurious peaks about iodine atoms.

```
PLAT342_ALERT_3_C Low Bond Precision on  C-C Bonds ..............    0.01142 Ang.
```

Author Response: Due to amount of modeled disorder within the structure.

```
PLAT906_ALERT_3_C Large K Value in the Analysis of Variance ......      3.433 Check
```

Author Response: This was due to diffuse scattering about Bragg peaks that was not account for during the integration and absorption correction.

```
PLAT911_ALERT_3_C Missing FCF Refl Between Thmin & STh/L=    0.597         21 Report
```

Author Response: These reflections were omitted during the absorption correction.

```
PLAT971_ALERT_2_C Check Calcd Resid. Dens.  1.14A   From I2              1.82 eA-3
```

Author Response: These are spurious peaks most likely from a minor twin component (<5%) that was present in the sample. Attempts to integrate the data with both components did not improve the residual density issue in this structure.

```
PLAT971_ALERT_2_C Check Calcd Resid. Dens.  1.10A   From I2              1.59 eA-3
```

Author Response: These are spurious peaks most likely from a minor twin component (<5%) that was present in the sample. Attempts to integrate the data with both components did not improve the residual density issue in this structure.

```
PLAT977_ALERT_2_C Check Negative Difference Density on H15B            -0.32 eA-3
```

Author Response: The hydrogen position was checked and appears to be correction based on the results of data collections at 230K and 270K.

Alert level G

```
PLAT002_ALERT_2_G Number of Distance or Angle Restraints on AtSite          7 Note
PLAT003_ALERT_2_G Number of Uiso or Uij Restrained non-H Atoms ...         16 Report
PLAT012_ALERT_1_G N.O.K.   _shelx_res_checksum Found in CIF ......     Please Check
PLAT013_ALERT_1_G N.O.K.   _shelx_hkl_checksum Found in CIF ......     Please Check
PLAT042_ALERT_1_G Calc. and Reported Moiety Formula Strings Differ     Please Check
PLAT083_ALERT_2_G SHELXL Second Parameter in WGHT  Unusually Large      13.90 Why ?
PLAT172_ALERT_4_G The CIF-Embedded .res File Contains DFIX Records          2 Report
PLAT178_ALERT_4_G The CIF-Embedded .res File Contains SIMU Records          1 Report
PLAT301_ALERT_3_G Main Residue  Disorder .............(Resd  1  )         67% Note
PLAT811_ALERT_5_G No ADDSYM Analysis: Too Many Excluded Atoms ....          ! Info
PLAT860_ALERT_3_G Number of Least-Squares Restraints ............         252 Note
PLAT909_ALERT_3_G Percentage of I>2sig(I) Data at Theta(Max) Still        57% Note
PLAT933_ALERT_2_G Number of OMIT Records in Embedded .res File ...          4 Note
```

PLAT978_ALERT_2_G Number C-C Bonds with Positive Residual Density. 0 Info

---

0 **ALERT level A** = Most likely a serious problem - resolve or explain
1 **ALERT level B** = A potentially serious problem, consider carefully
8 **ALERT level C** = Check. Ensure it is not caused by an omission or oversight
14 **ALERT level G** = General information/check it is not something unexpected

3 ALERT type 1 CIF construction/syntax error, inconsistent or missing data
11 ALERT type 2 Indicator that the structure model may be wrong or deficient
6 ALERT type 3 Indicator that the structure quality may be low
2 ALERT type 4 Improvement, methodology, query or suggestion
1 ALERT type 5 Informative message, check

Datablock: diazoI_270

Bond precision: C-C = 0.0082 A Wavelength=1.54184

Cell:	a=7.3174(1)	b=40.4302(4)	c=5.9603(1)
	alpha=90	beta=90.066(1)	gamma=90
Temperature:	270 K		

	Calculated	Reported
Volume	1763.32(4)	1763.32(4)
Space group	P 21/c	P 1 21/c 1
Hall group	-P 2ybc	-P 2ybc
Moiety formula	C18 H12 I2 N4	2(C9 H6 I N2)
Sum formula	C18 H12 I2 N4	C18 H12 I2 N4
Mr	538.12	538.12
Dx,g cm-3	2.027	2.027
Z	4	4
Mu (mm-1)	28.065	28.065
F000	1016.0	1016.0
F000’	1016.58	
h,k,lmax	8,48,7	8,48,7
Nref	3149	3130
Tmin,Tmax	0.213,0.714	0.254,0.757
Tmin’	0.137	

Correction method= # Reported T Limits: Tmin=0.254 Tmax=0.757
AbsCorr = GAUSSIAN

Data completeness= 0.994 Theta(max)= 67.071

R(reflections)= 0.0401(2428) wR2(reflections)= 0.0939(3130)

S = 1.056 Npar= 342

The following ALERTS were generated. Each ALERT has the format
test-name_ALERT_alert-type_alert-level.
Click on the hyperlinks for more details of the test.

Alert level C

PLAT088_ALERT_3_C Poor Data / Parameter Ratio .... 9.15 Note

Author Response: Due to the amount of disorder within the structure at this temperature.

PLAT241_ALERT_2_C High 'MainMol' Ueq as Compared to Neighbors of I1 Check

Author Response: Due to the spurious peaks about iodine atoms.

PLAT241_ALERT_2_C High 'MainMol' Ueq as Compared to Neighbors of I2 Check

Author Response: Due to the spurious peaks about iodine atoms.

PLAT342_ALERT_3_C Low Bond Precision on C-C Bonds .... 0.00817 Ang.

Author Response: Due to amount of modeled disorder within the structure.

PLAT906_ALERT_3_C Large K Value in the Analysis of Variance ...... 4.312 Check

Author Response: This was due to diffuse scattering about Bragg peaks that was not account for during the integration and absorption correction.

PLAT911_ALERT_3_C Missing FCF Refl Between Thmin & STh/L= 0.597 19 Report

Author Response: These reflections were omitted during the absorption correction.

Alert level G

```
PLAT002_ALERT_2_G Number of Distance or Angle Restraints on AtSite        14 Note
PLAT003_ALERT_2_G Number of Uiso or Uij Restrained non-H Atoms ...        32 Report
PLAT012_ALERT_1_G N.O.K.   _shelx_res_checksum Found in CIF ......     Please Check
PLAT013_ALERT_1_G N.O.K.   _shelx_hkl_checksum Found in CIF ......     Please Check
PLAT042_ALERT_1_G Calc. and Reported Moiety Formula Strings Differ     Please Check
PLAT142_ALERT_4_G s.u. on b - Axis Small or Missing .............    0.00040 Ang.
PLAT172_ALERT_4_G The CIF-Embedded .res File Contains DFIX Records         2 Report
PLAT178_ALERT_4_G The CIF-Embedded .res File Contains SIMU Records         2 Report
PLAT301_ALERT_3_G Main Residue  Disorder ..............(Resd  1  )       67% Note
PLAT301_ALERT_3_G Main Residue  Disorder ..............(Resd  2  )       67% Note
PLAT811_ALERT_5_G No ADDSYM Analysis: Too Many Excluded Atoms ....         ! Info
PLAT860_ALERT_3_G Number of Least-Squares Restraints ............        504 Note
PLAT909_ALERT_3_G Percentage of I>2sig(I) Data at Theta(Max) Still       54% Note
PLAT933_ALERT_2_G Number of OMIT Records in Embedded .res File ...         5 Note
PLAT978_ALERT_2_G Number C-C Bonds with Positive Residual Density.         2 Info
```

```
   0 ALERT level A = Most likely a serious problem - resolve or explain
   0 ALERT level B = A potentially serious problem, consider carefully
   6 ALERT level C = Check. Ensure it is not caused by an omission or oversight
  15 ALERT level G = General information/check it is not something unexpected

   3 ALERT type 1 CIF construction/syntax error, inconsistent or missing data
   6 ALERT type 2 Indicator that the structure model may be wrong or deficient
   8 ALERT type 3 Indicator that the structure quality may be low
   3 ALERT type 4 Improvement, methodology, query or suggestion
   1 ALERT type 5 Informative message, check
```

Datablock: diazoI_290

```
Bond precision:  C-C = 0.0078 A          Wavelength=1.54184

Cell:            a=7.3493(1)     b=40.3729(4)       c=5.9677(1)
                 alpha=90        beta=90.006(1)     gamma=90
Temperature:     290 K
```

	Calculated	Reported
Volume	1770.69(4)	1770.69(4)
Space group	P 21/c	P 1 21/c 1
Hall group	-P 2ybc	-P 2ybc
Moiety formula	C18 H12 I2 N4	2(C9 H6 I N2)
Sum formula	C18 H12 I2 N4	C18 H12 I2 N4
Mr	538.12	538.12
Dx,g cm-3	2.019	2.019
Z	4	4
Mu (mm-1)	27.948	27.948
F000	1016.0	1016.0
F000’	1016.58	
h,k,lmax	8,48,7	8,48,7
Nref	3151	3130
Tmin,Tmax	0.215,0.715	0.490,0.897
Tmin’	0.138	

```
Correction method= # Reported T Limits: Tmin=0.490 Tmax=0.897
AbsCorr = GAUSSIAN

Data completeness= 0.993          Theta(max)= 67.071

R(reflections)= 0.0400( 2216)     wR2(reflections)= 0.1151( 3130)

S = 1.083                  Npar= 342
```

The following ALERTS were generated. Each ALERT has the format
test-name_ALERT_alert-type_alert-level.
Click on the hyperlinks for more details of the test.

Alert level C

PLAT088_ALERT_3_C Poor Data / Parameter Ratio .... 9.15 Note

Author Response: Due to the amount of disorder within the structure at this temperature.

PLAT241_ALERT_2_C High 'MainMol' Ueq as Compared to Neighbors of I1 Check

Author Response: Due to the spurious peaks about iodine atoms.

PLAT241_ALERT_2_C High 'MainMol' Ueq as Compared to Neighbors of I2 Check

Author Response: Due to the spurious peaks about iodine atoms.

PLAT906_ALERT_3_C Large K Value in the Analysis of Variance ...... 33.850 Check

Author Response: This was due to diffuse scattering about Bragg peaks that was not account for during the integration and absorption correction along with the loss of some high angle reflections.

PLAT906_ALERT_3_C Large K Value in the Analysis of Variance ...... 2.007 Check

Author Response: This was due to diffuse scattering about Bragg peaks that was not account for during the integration and absorption correction along with the loss of some high angle reflections.

PLAT911_ALERT_3_C Missing FCF Refl Between Thmin & STh/L= 0.597 22 Report

Author Response: These reflections were omitted during the absorption correction.

Alert level G

```
PLAT002_ALERT_2_G Number of Distance or Angle Restraints on AtSite         14 Note
PLAT003_ALERT_2_G Number of Uiso or Uij Restrained non-H Atoms ...         32 Report
PLAT012_ALERT_1_G N.O.K.   _shelx_res_checksum Found in CIF ......     Please Check
PLAT013_ALERT_1_G N.O.K.   _shelx_hkl_checksum Found in CIF ......     Please Check
PLAT042_ALERT_1_G Calc. and Reported Moiety Formula Strings Differ     Please Check
PLAT142_ALERT_4_G s.u. on b - Axis Small or Missing .............    0.00040 Ang.
PLAT172_ALERT_4_G The CIF-Embedded .res File Contains DFIX Records          2 Report
PLAT178_ALERT_4_G The CIF-Embedded .res File Contains SIMU Records          2 Report
PLAT301_ALERT_3_G Main Residue  Disorder .............(Resd  1  )        67% Note
PLAT301_ALERT_3_G Main Residue  Disorder .............(Resd  2  )        67% Note
PLAT811_ALERT_5_G No ADDSYM Analysis: Too Many Excluded Atoms ....          ! Info
PLAT860_ALERT_3_G Number of Least-Squares Restraints ............         510 Note
PLAT909_ALERT_3_G Percentage of I>2sig(I) Data at Theta(Max) Still        48% Note
PLAT933_ALERT_2_G Number of OMIT Records in Embedded .res File ...          6 Note
```

PLAT978_ALERT_2_G Number C-C Bonds with Positive Residual Density. 3 Info

0 **ALERT level A** = Most likely a serious problem - resolve or explain
0 **ALERT level B** = A potentially serious problem, consider carefully
6 **ALERT level C** = Check. Ensure it is not caused by an omission or oversight
15 **ALERT level G** = General information/check it is not something unexpected

3 ALERT type 1 CIF construction/syntax error, inconsistent or missing data
6 ALERT type 2 Indicator that the structure model may be wrong or deficient
8 ALERT type 3 Indicator that the structure quality may be low
3 ALERT type 4 Improvement, methodology, query or suggestion
1 ALERT type 5 Informative message, check

Datablock: diazoBr_190K

Bond precision: C-C = 0.0061 A Wavelength=1.54178

Cell:	a=3.90194(10)	b=5.88142(12)	c=34.9288(8)
	alpha=90	beta=91.343(2)	gamma=90
Temperature:	190 K		

	Calculated	Reported
Volume	801.36(3)	801.36(3)
Space group	P 21/n	P 21/n
Hall group	-P 2yn	-P 2yn
Moiety formula	C18 H12 Br2 N4	?
Sum formula	C18 H12 Br2 N4	C18 H12 Br2 N4
Mr	444.12	444.14
Dx,g cm-3	1.841	1.841
Z	2	2
Mu (mm-1)	6.469	6.469
F000	436.0	436.0
F000’	434.13	
h,k,lmax	4,7,44	4,7,44
Nref	1683	1633
Tmin,Tmax	0.600,0.890	0.274,0.978
Tmin’	0.390	

Correction method= # Reported T Limits: Tmin=0.274 Tmax=0.978
AbsCorr = GAUSSIAN

Data completeness= 0.970 Theta(max)= 76.946

R(reflections)= 0.0414(1526) wR2(reflections)= 0.0947(1633)

S = 1.158 Npar= 109

The following ALERTS were generated. Each ALERT has the format
test-name_ALERT_alert-type_alert-level.
Click on the hyperlinks for more details of the test.

Alert level C

PLAT341_ALERT_3_C Low Bond Precision on C-C Bonds .... 0.00611 Ang.

Author Response: Due to diffuse scattering near Bragg peaks.

PLAT906_ALERT_3_C Large K Value in the Analysis of Variance ...... 3.607 Check

Author Response: Due to diffuse scattering near Bragg peaks.

PLAT911_ALERT_3_C Missing FCF Refl Between Thmin & STh/L= 0.600 13 Report

Alert level G

PLAT912_ALERT_4_G Missing # of FCF Reflections Above STh/L= 0.600 38 Note
PLAT933_ALERT_2_G Number of OMIT Records in Embedded .res File ... 3 Note
PLAT978_ALERT_2_G Number C-C Bonds with Positive Residual Density. 2 Info

0 **ALERT level A** = Most likely a serious problem - resolve or explain
0 **ALERT level B** = A potentially serious problem, consider carefully
3 **ALERT level C** = Check. Ensure it is not caused by an omission or oversight
3 **ALERT level G** = General information/check it is not something unexpected

0 ALERT type 1 CIF construction/syntax error, inconsistent or missing data
2 ALERT type 2 Indicator that the structure model may be wrong or deficient
3 ALERT type 3 Indicator that the structure quality may be low
1 ALERT type 4 Improvement, methodology, query or suggestion
0 ALERT type 5 Informative message, check

Datablock: diazoBr_210K

Bond precision: C-C = 0.0032 A Wavelength=1.54178

Cell:	a=3.90964(6)	b=5.88619(7)	c=34.9231(5)
	alpha=90	beta=91.3351(12)	gamma=90
Temperature:	210 K		

	Calculated	Reported
Volume	803.463(19)	803.465(18)
Space group	P 21/n	P 21/n
Hall group	-P 2yn	-P 2yn
Moiety formula	C18 H12 Br2 N4	?
Sum formula	C18 H12 Br2 N4	C18 H12 Br2 N4
Mr	444.12	444.14
Dx,g cm-3	1.836	1.836
Z	2	2
Mu (mm-1)	6.452	6.452
F000	436.0	436.0
F000’	434.13	
h,k,lmax	4,7,44	4,7,44
Nref	1692	1670
Tmin,Tmax	0.601,0.890	0.663,1.000
Tmin’	0.391	

Correction method= # Reported T Limits: Tmin=0.663 Tmax=1.000
AbsCorr = GAUSSIAN

Data completeness= 0.987 Theta(max)= 77.327

R(reflections)= 0.0278(1569) wR2(reflections)= 0.0699(1670)

S = 1.090 Npar= 109

The following ALERTS were generated. Each ALERT has the format
test-name_ALERT_alert-type_alert-level.
Click on the hyperlinks for more details of the test.

Alert level C

PLAT906_ALERT_3_C Large K Value in the Analysis of Variance ...... 2.207 Check

Author Response: Due to diffuse scattering near Bragg peaks.

PLAT911_ALERT_3_C Missing FCF Refl Between Thmin & STh/L= 0.600 3 Report

---

Alert level G

PLAT912_ALERT_4_G Missing # of FCF Reflections Above STh/L= 0.600 20 Note
PLAT978_ALERT_2_G Number C-C Bonds with Positive Residual Density. 5 Info

---

0 **ALERT level A** = Most likely a serious problem - resolve or explain
0 **ALERT level B** = A potentially serious problem, consider carefully
2 **ALERT level C** = Check. Ensure it is not caused by an omission or oversight
2 **ALERT level G** = General information/check it is not something unexpected

0 ALERT type 1 CIF construction/syntax error, inconsistent or missing data

1 ALERT type 2 Indicator that the structure model may be wrong or deficient
2 ALERT type 3 Indicator that the structure quality may be low
1 ALERT type 4 Improvement, methodology, query or suggestion
0 ALERT type 5 Informative message, check

Datablock: diazoBr_230K

Bond precision: C-C = 0.0030 A Wavelength=1.54178

Cell:	a=3.91829(6)	b=5.89122(7)	c=34.9307(5)
	alpha=90	beta=91.3238(12)	gamma=90
Temperature:	230 K		

	Calculated	Reported
Volume	806.108(19)	806.109(18)
Space group	P 21/n	P 21/n
Hall group	-P 2yn	-P 2yn
Moiety formula	C18 H12 Br2 N4	?
Sum formula	C18 H12 Br2 N4	C18 H12 Br2 N4
Mr	444.12	444.14
Dx,g cm-3	1.830	1.830
Z	2	2
Mu (mm-1)	6.430	6.430
F000	436.0	436.0
F000’	434.13	
h,k,lmax	4,7,44	4,7,44
Nref	1698	1676
Tmin,Tmax	0.602,0.891	0.662,1.000
Tmin’	0.392	

Correction method= # Reported T Limits: Tmin=0.662 Tmax=1.000
AbsCorr = GAUSSIAN

Data completeness= 0.987 Theta(max)= 77.427

R(reflections)= 0.0270(1563) wR2(reflections)= 0.0708(1676)

S = 1.132 Npar= 109

The following ALERTS were generated. Each ALERT has the format
test-name_ALERT_alert-type_alert-level.
Click on the hyperlinks for more details of the test.

Alert level C

PLAT911_ALERT_3_C Missing FCF Refl Between Thmin & STh/L= 0.600 3 Report

Alert level G

```
PLAT912_ALERT_4_G Missing # of FCF Reflections Above STh/L=  0.600         20 Note
PLAT978_ALERT_2_G Number C-C Bonds with Positive Residual Density.          4 Info
```

```
   0 ALERT level A = Most likely a serious problem - resolve or explain
   0 ALERT level B = A potentially serious problem, consider carefully
   1 ALERT level C = Check. Ensure it is not caused by an omission or oversight
   2 ALERT level G = General information/check it is not something unexpected

   0 ALERT type 1 CIF construction/syntax error, inconsistent or missing data
   1 ALERT type 2 Indicator that the structure model may be wrong or deficient
   1 ALERT type 3 Indicator that the structure quality may be low
   1 ALERT type 4 Improvement, methodology, query or suggestion
   0 ALERT type 5 Informative message, check
```

Datablock: diazoBr_250K

Bond precision: C-C = 0.0030 A Wavelength=1.54178

Cell:	a=3.92669(6)	b=5.89602(7)	c=34.9384(5)
	alpha=90	beta=91.3143(13)	gamma=90
Temperature:	250 K		

	Calculated	Reported
Volume	808.676(19)	808.676(19)
Space group	P 21/n	P 21/n
Hall group	-P 2yn	-P 2yn
Moiety formula	C18 H12 Br2 N4	?
Sum formula	C18 H12 Br2 N4	C18 H12 Br2 N4
Mr	444.12	444.14
Dx,g cm-3	1.824	1.824
Z	2	2
Mu (mm-1)	6.410	6.410
F000	436.0	436.0
F000’	434.13	
h,k,lmax	4,7,44	4,7,44
Nref	1701	1675
Tmin,Tmax	0.603,0.891	0.661,1.000
Tmin’	0.393	

Correction method= # Reported T Limits: Tmin=0.661 Tmax=1.000
AbsCorr = GAUSSIAN

Data completeness= 0.985 Theta(max)= 77.487

R(reflections)= 0.0275(1547) wR2(reflections)= 0.0693(1675)

S = 1.088 Npar= 109

The following ALERTS were generated. Each ALERT has the format
test-name_ALERT_alert-type_alert-level.
Click on the hyperlinks for more details of the test.

Alert level C

PLAT911_ALERT_3_C Missing FCF Refl Between Thmin & STh/L= 0.600 5 Report

---

Alert level G

PLAT912_ALERT_4_G Missing # of FCF Reflections Above STh/L= 0.600 22 Note
PLAT933_ALERT_2_G Number of OMIT Records in Embedded .res File ... 1 Note
PLAT978_ALERT_2_G Number C-C Bonds with Positive Residual Density. 5 Info

---

0 **ALERT level A** = Most likely a serious problem - resolve or explain
0 **ALERT level B** = A potentially serious problem, consider carefully
1 **ALERT level C** = Check. Ensure it is not caused by an omission or oversight
3 **ALERT level G** = General information/check it is not something unexpected

0 ALERT type 1 CIF construction/syntax error, inconsistent or missing data
2 ALERT type 2 Indicator that the structure model may be wrong or deficient
1 ALERT type 3 Indicator that the structure quality may be low
1 ALERT type 4 Improvement, methodology, query or suggestion
0 ALERT type 5 Informative message, check

Datablock: diazoBr_270K

Bond precision: C-C = 0.0030 A Wavelength=1.54178

Cell:	a=3.93600(7)	b=5.90113(9)	c=34.9507(6)
	alpha=90	beta=91.2861(16)	gamma=90
Temperature:	270 K		

	Calculated	Reported
Volume	811.59(2)	811.59(2)
Space group	P 21/n	P 21/n
Hall group	-P 2yn	-P 2yn
Moiety formula	C18 H12 Br2 N4	?
Sum formula	C18 H12 Br2 N4	C18 H12 Br2 N4
Mr	444.12	444.14
Dx,g cm-3	1.817	1.817
Z	2	2
Mu (mm-1)	6.387	6.387
F000	436.0	436.0
F000’	434.13	
h,k,lmax	4,7,44	4,7,44
Nref	1703	1674
Tmin,Tmax	0.605,0.891	0.660,1.000
Tmin’	0.394	

Correction method= # Reported T Limits: Tmin=0.660 Tmax=1.000
AbsCorr = GAUSSIAN

Data completeness= 0.983   Theta(max)= 77.356

R(reflections)= 0.0283(1533)   wR2(reflections)= 0.0744(1674)

S = 1.083   Npar= 109

The following ALERTS were generated. Each ALERT has the format
test-name_ALERT_alert-type_alert-level.
Click on the hyperlinks for more details of the test.

Alert level C

PLAT911_ALERT_3_C Missing FCF Refl Between Thmin & STh/L= 0.600 6 Report

---

Alert level G

PLAT912_ALERT_4_G Missing # of FCF Reflections Above STh/L= 0.600 24 Note
PLAT933_ALERT_2_G Number of OMIT Records in Embedded .res File ... 1 Note
PLAT978_ALERT_2_G Number C-C Bonds with Positive Residual Density. 4 Info

---

0 **ALERT level A** = Most likely a serious problem - resolve or explain
0 **ALERT level B** = A potentially serious problem, consider carefully
1 **ALERT level C** = Check. Ensure it is not caused by an omission or oversight
3 **ALERT level G** = General information/check it is not something unexpected

0 ALERT type 1 CIF construction/syntax error, inconsistent or missing data
2 ALERT type 2 Indicator that the structure model may be wrong or deficient
1 ALERT type 3 Indicator that the structure quality may be low
1 ALERT type 4 Improvement, methodology, query or suggestion
0 ALERT type 5 Informative message, check

Datablock: diazoBr_290K

Bond precision: C-C = 0.0030 A Wavelength=1.54178

Cell:	a=3.94658(7)	b=5.90597(8)	c=34.9552(5)
	alpha=90	beta=91.2515(15)	gamma=90
Temperature:	290 K		

	Calculated	Reported
Volume	814.56(2)	814.56(2)
Space group	P 21/n	P 21/n
Hall group	-P 2yn	-P 2yn
Moiety formula	C18 H12 Br2 N4	?
Sum formula	C18 H12 Br2 N4	C18 H12 Br2 N4
Mr	444.12	444.14
Dx,g cm-3	1.811	1.811
Z	2	2
Mu (mm-1)	6.364	6.364
F000	436.0	436.0
F000’	434.13	
h,k,lmax	4,7,44	4,7,44
Nref	1705	1664
Tmin,Tmax	0.606,0.892	0.659,1.000
Tmin’	0.396	

Correction method= # Reported T Limits: Tmin=0.659 Tmax=1.000
AbsCorr = GAUSSIAN

Data completeness= 0.976 Theta(max)= 77.202

R(reflections)= 0.0279(1492) wR2(reflections)= 0.0751(1664)

S = 1.113 Npar= 109

The following ALERTS were generated. Each ALERT has the format
test-name_ALERT_alert-type_alert-level.
Click on the hyperlinks for more details of the test.

Alert level C

PLAT911_ALERT_3_C Missing FCF Refl Between Thmin & STh/L= 0.600 8 Report

---

Alert level G

PLAT912_ALERT_4_G Missing # of FCF Reflections Above STh/L= 0.600 34 Note
PLAT933_ALERT_2_G Number of OMIT Records in Embedded .res File ... 1 Note
PLAT978_ALERT_2_G Number C-C Bonds with Positive Residual Density. 2 Info

---

```
0 ALERT level A = Most likely a serious problem - resolve or explain
0 ALERT level B = A potentially serious problem, consider carefully
1 ALERT level C = Check. Ensure it is not caused by an omission or oversight
3 ALERT level G = General information/check it is not something unexpected

0 ALERT type 1 CIF construction/syntax error, inconsistent or missing data
2 ALERT type 2 Indicator that the structure model may be wrong or deficient
1 ALERT type 3 Indicator that the structure quality may be low
1 ALERT type 4 Improvement, methodology, query or suggestion
0 ALERT type 5 Informative message, check
```

Validation response form

Please find below a validation response form (VRF) that can be filled in and pasted into your CIF.

```
# start Validation Reply Form
_vrf_PLAT911_diazoBr_190K
;
PROBLEM: Missing FCF Refl Between Thmin & STh/L=    0.600         13 Report
RESPONSE: ...
;
_vrf_PLAT911_diazoBr_210K
;
PROBLEM: Missing FCF Refl Between Thmin & STh/L=    0.600          3 Report
RESPONSE: ...
;
_vrf_PLAT911_diazoBr_230K
;
PROBLEM: Missing FCF Refl Between Thmin & STh/L=    0.600          3 Report
RESPONSE: ...
;
_vrf_PLAT911_diazoBr_250K
;
PROBLEM: Missing FCF Refl Between Thmin & STh/L=    0.600          5 Report
RESPONSE: ...
;
_vrf_PLAT911_diazoBr_270K
;
PROBLEM: Missing FCF Refl Between Thmin & STh/L=    0.600          6 Report
RESPONSE: ...
;
_vrf_PLAT911_diazoBr_290K
;
PROBLEM: Missing FCF Refl Between Thmin & STh/L=    0.600          8 Report
RESPONSE: ...
;
# end Validation Reply Form
```

It is advisable to attempt to resolve as many as possible of the alerts in all categories. Often the minor alerts point to easily fixed oversights, errors and omissions in your CIF or refinement strategy, so attention to these fine details can be worthwhile. In order to resolve some of the more serious problems it may be necessary to carry out additional measurements or structure refinements. However, the purpose of your study may justify the reported deviations and the more serious of these should normally be commented upon in the discussion or experimental section of a paper or in the "special_details" fields of the CIF. checkCIF was carefully designed to identify outliers and unusual parameters, but every test has its limitations and alerts that are not important in a particular case may appear. Conversely, the absence of alerts does not guarantee there are no aspects of the results needing attention. It is up to the individual to critically assess their own results and, if necessary, seek expert advice.

Publication of your CIF in IUCr journals

A basic structural check has been run on your CIF. These basic checks will be run on all CIFs submitted for publication in IUCr journals (*Acta Crystallographica*, *Journal of Applied Crystallography*, *Journal of Synchrotron Radiation*); however, if you intend to submit to *Acta Crystallographica Section C* or *E* or *IUCrData*, you should make sure that full publication checks are run on the final version of your CIF prior to submission.

Publication of your CIF in other journals

Please refer to the *Notes for Authors* of the relevant journal for any special instructions relating to CIF submission.

PLATON version of 03/06/2021; check.def file version of 02/06/2021

Datablock diazoI_190 - ellipsoid plot

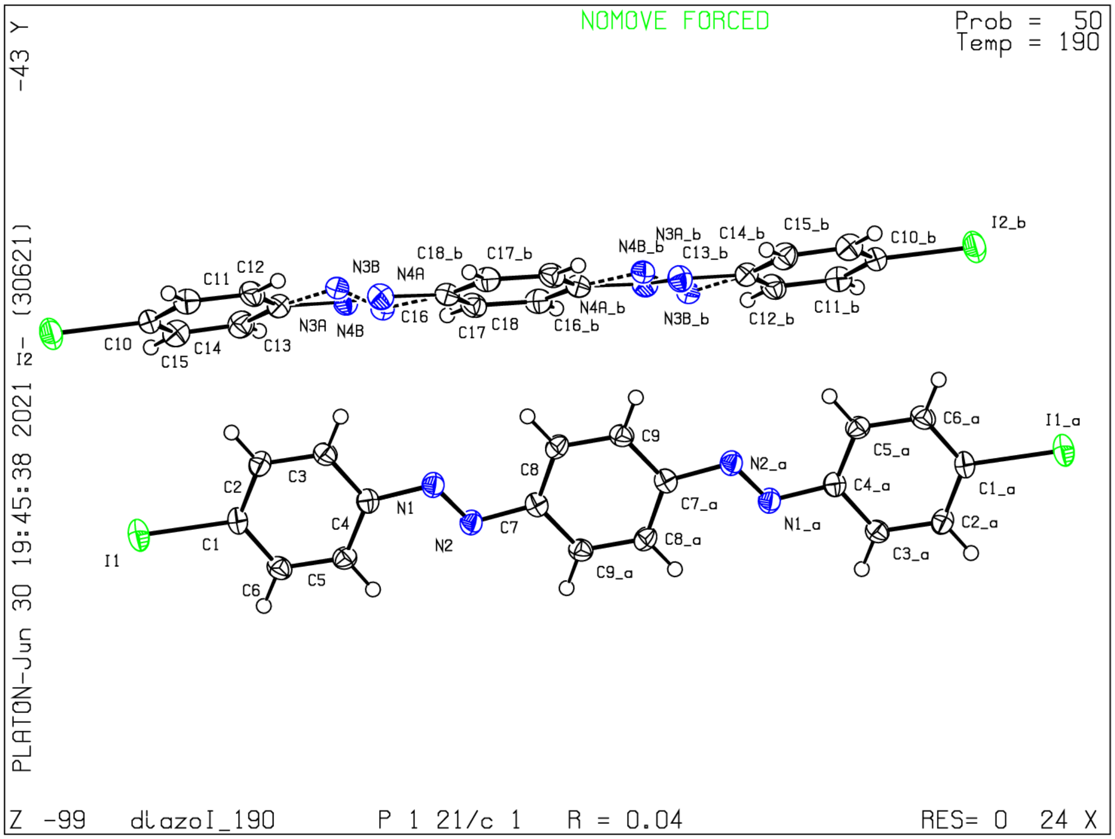

Datablock diazoI_210 - ellipsoid plot

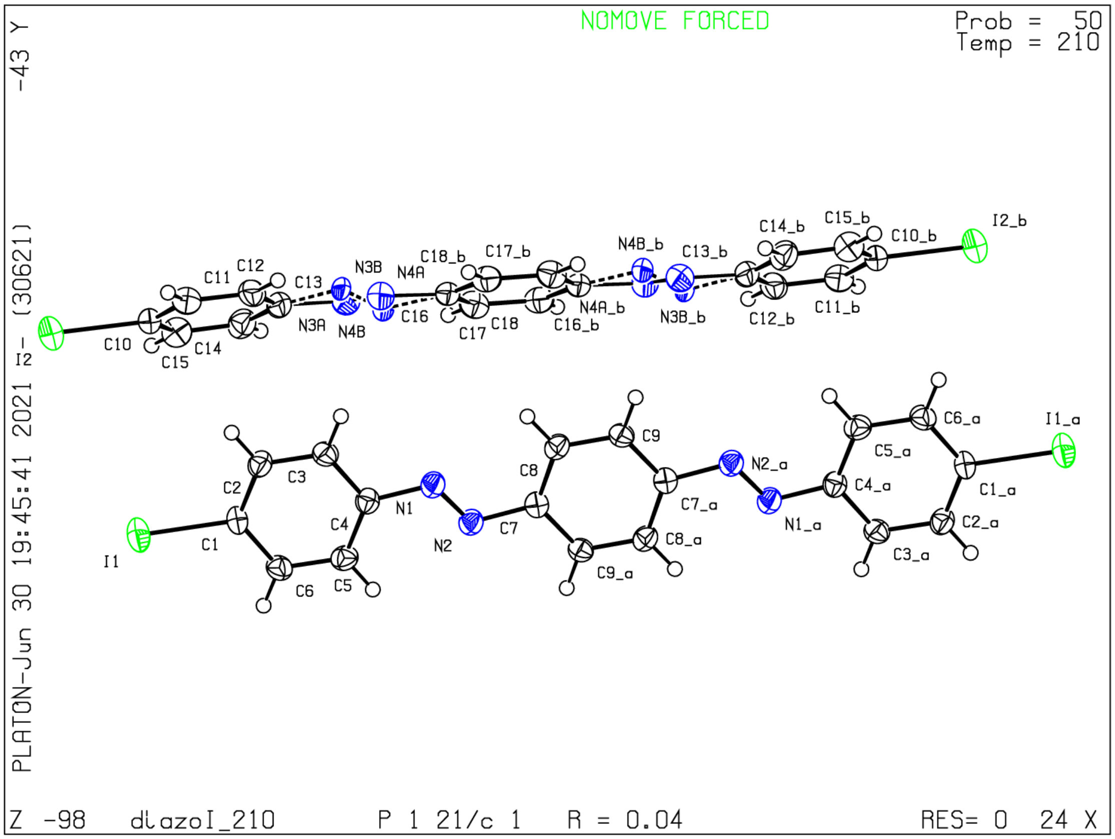

Datablock diazoI_230 - ellipsoid plot

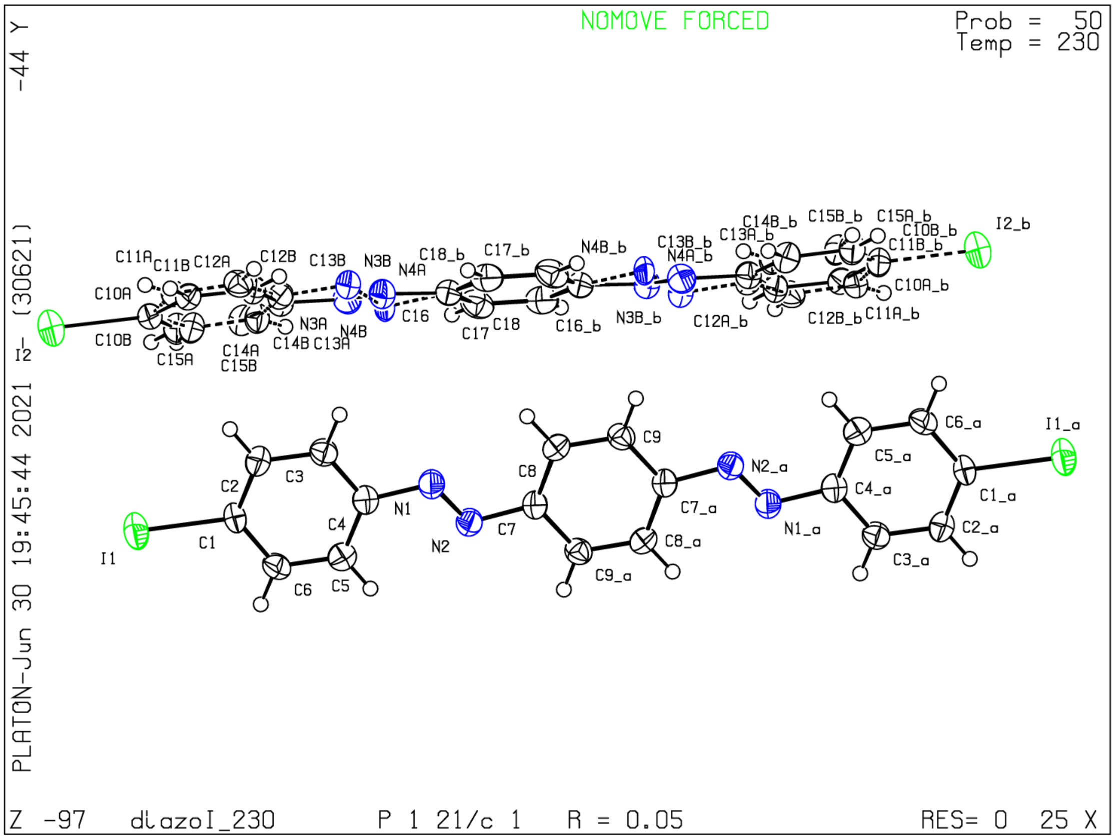

Datablock diazoI_250 - ellipsoid plot

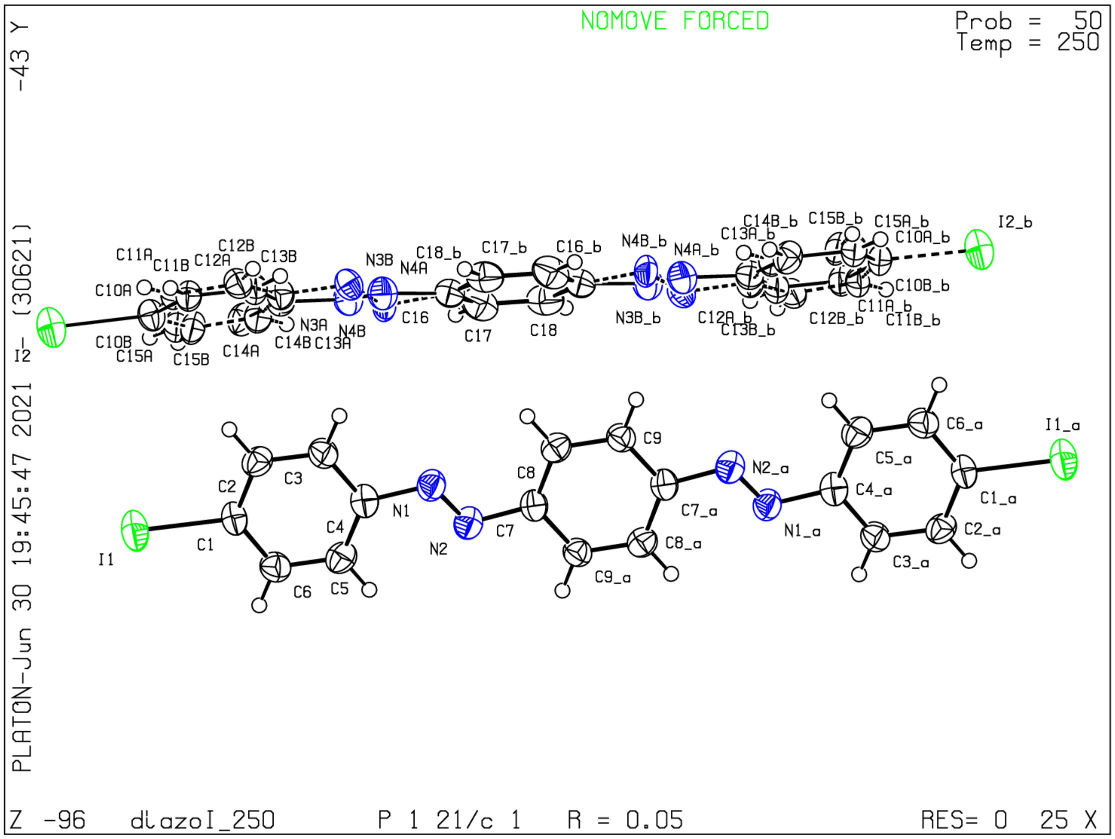

Datablock diazoI_270 - ellipsoid plot

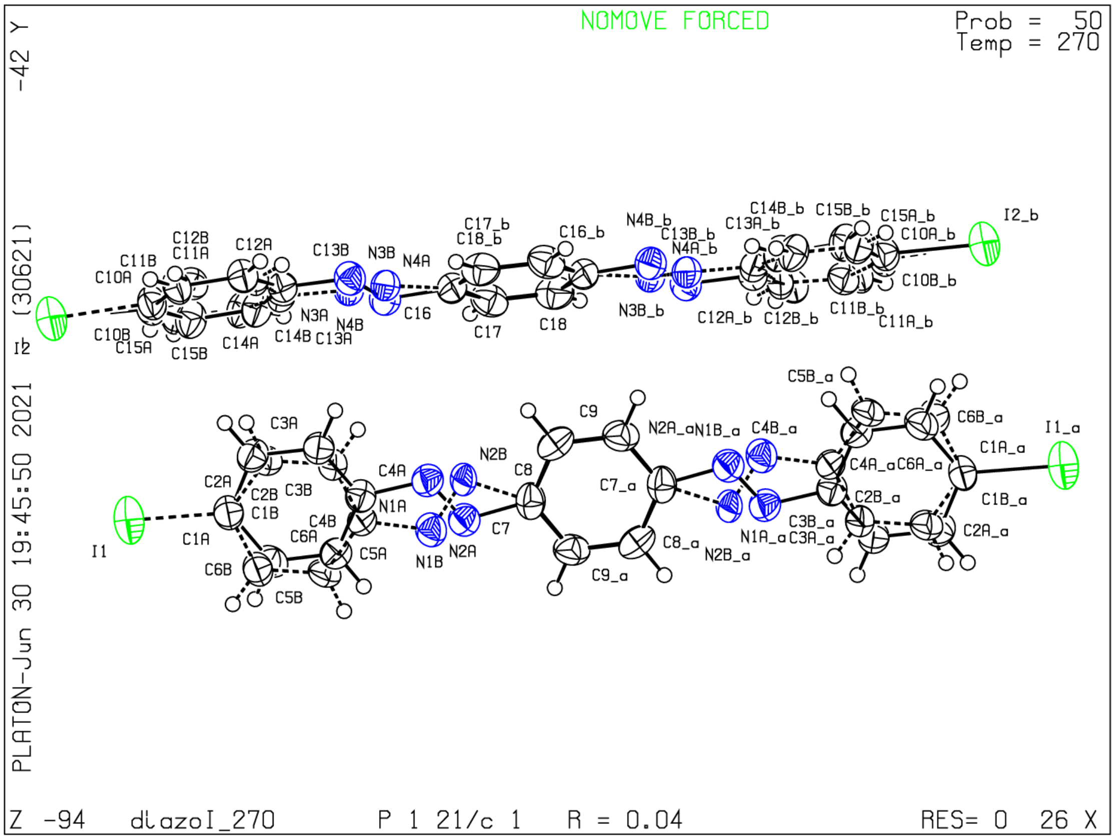

Datablock diazoI_290 - ellipsoid plot

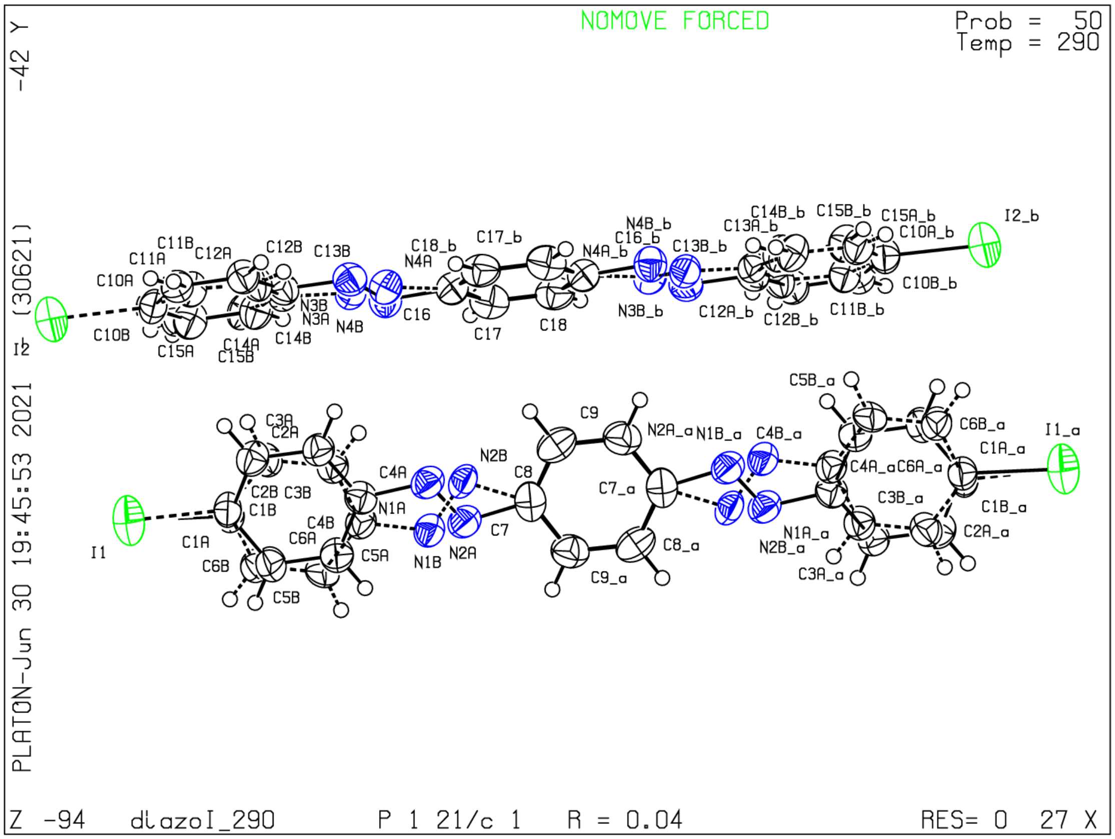

Datablock diazoBr_190K - ellipsoid plot

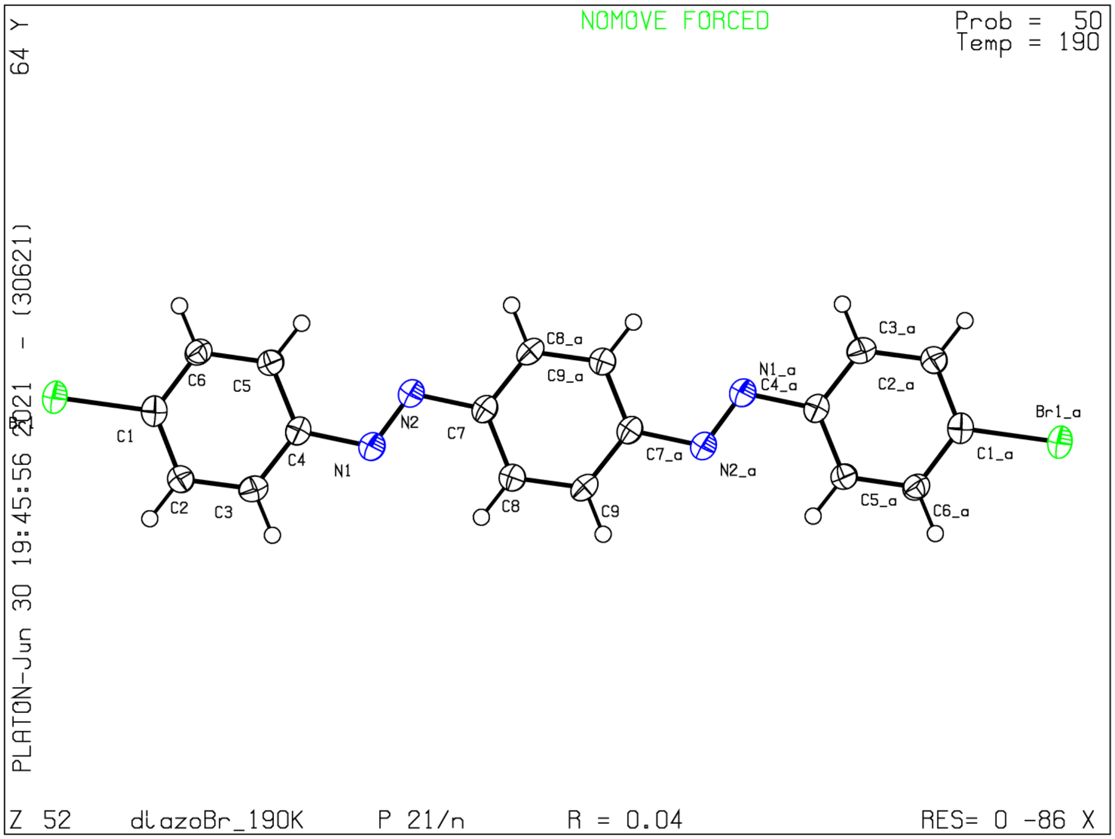

Datablock diazoBr_210K - ellipsoid plot

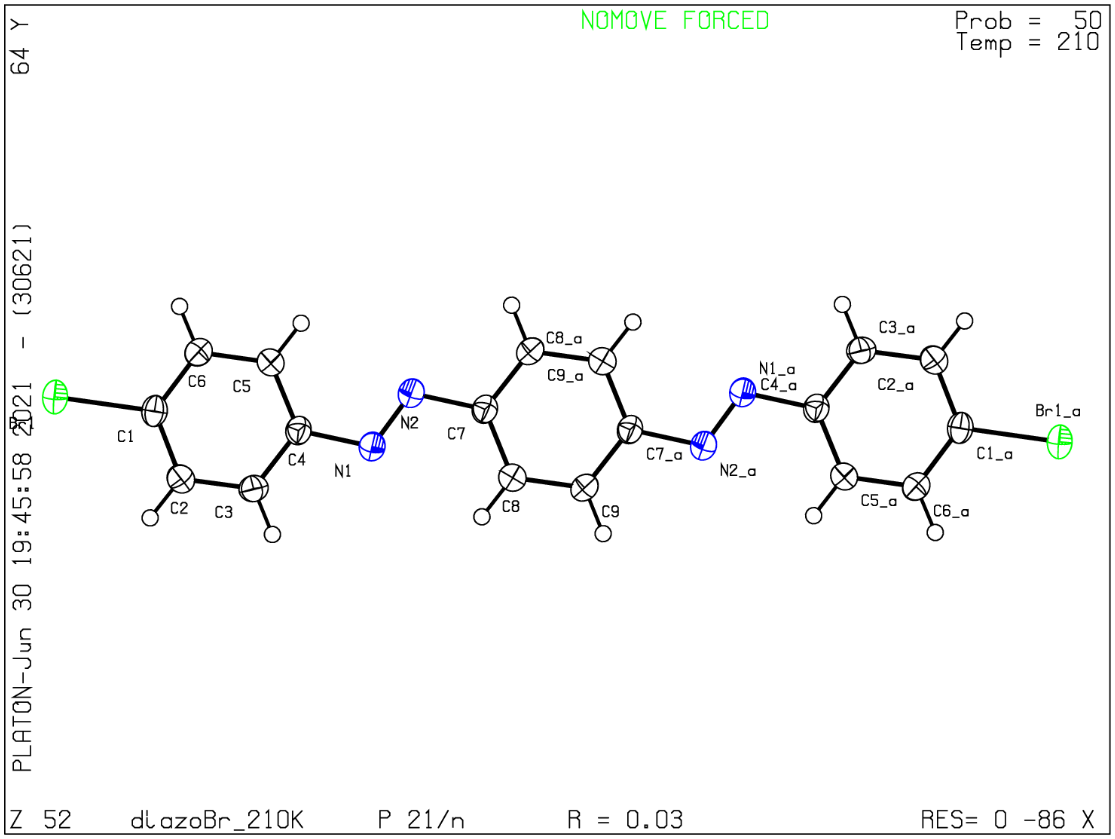

Datablock diazoBr_230K - ellipsoid plot

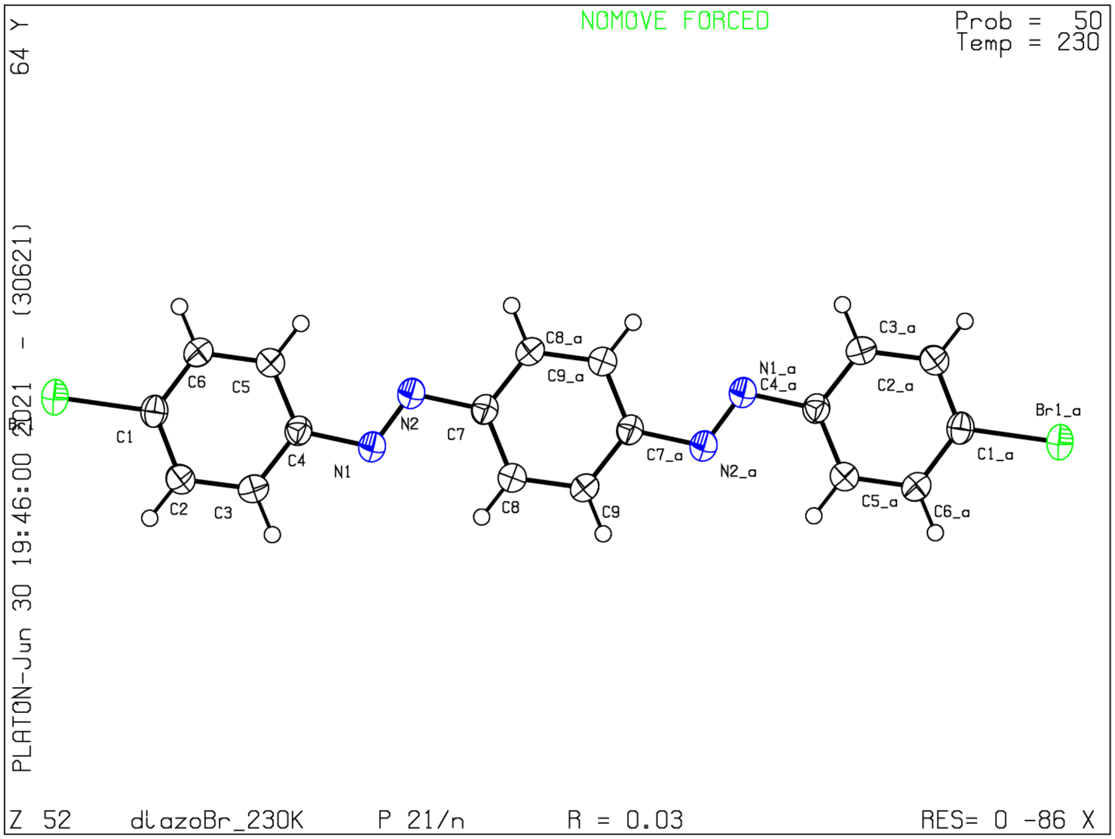

Datablock diazoBr_250K - ellipsoid plot

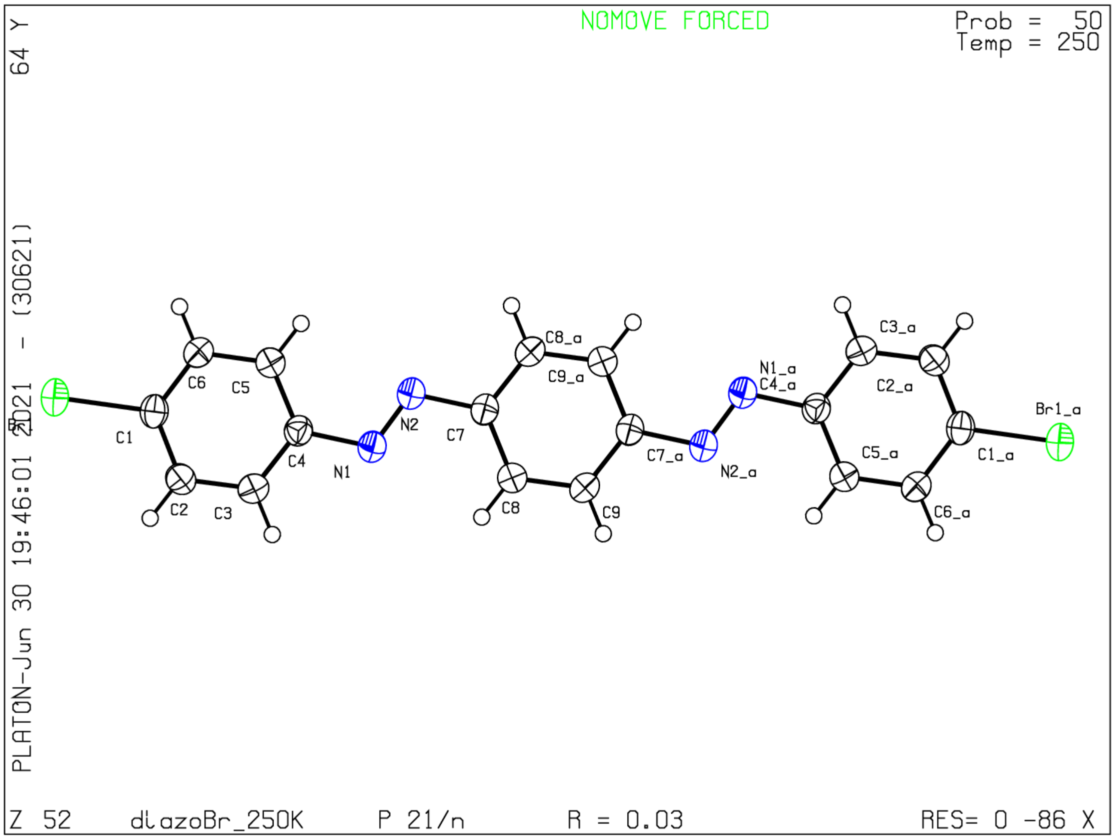

Datablock diazoBr_270K - ellipsoid plot

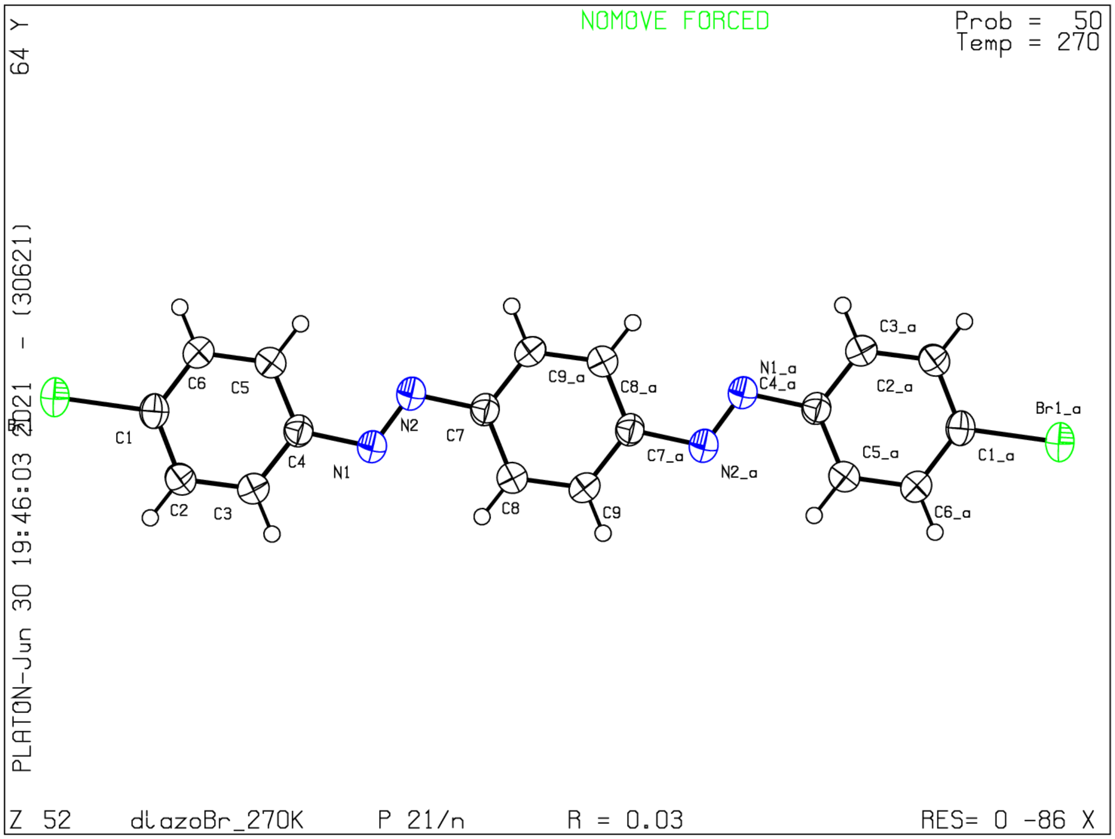

Datablock diazoBr_290K - ellipsoid plot

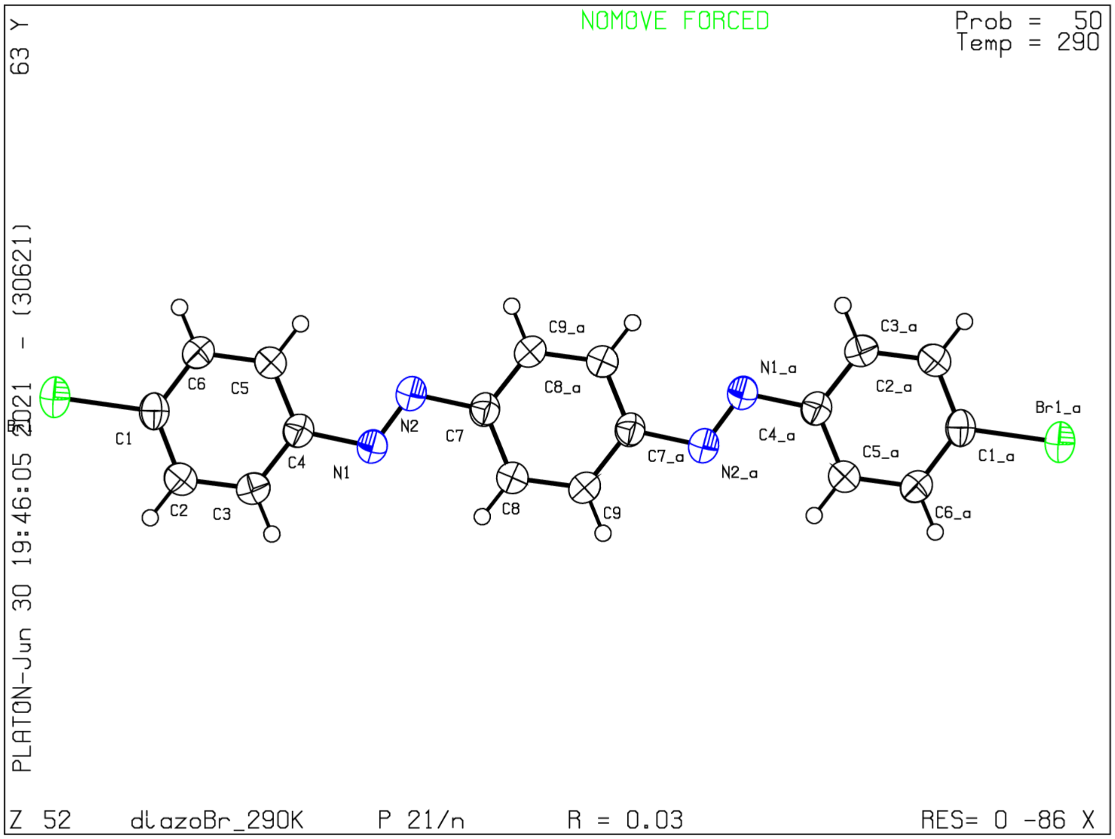

Supplement: Supplementary file 3 [file diazo-I + diazo-Br.pdf › diazo-I + diazo-Br.pdf]
