# Supplementary material for: Differences in thermal expansion and motion ability for herringbone and face-to-face π-stacked solids
Source: IUCrJ. 2021 Nov 3;9(Pt 1):31–42. doi: 10.1107/S2052252521009593 (PMC8733877; doi:10.1107/S2052252521009593)

checkCIF/PLATON report

Structure factors have been supplied for datablock(s) diazoIBr_190, diazoIBr_210, diazoIBr_230, diazoIBr_250, diazoIBr_270, diazoIBr_290

THIS REPORT IS FOR GUIDANCE ONLY. IF USED AS PART OF A REVIEW PROCEDURE FOR PUBLICATION, IT SHOULD NOT REPLACE THE EXPERTISE OF AN EXPERIENCED CRYSTALLOGRAPHIC REFEREE.

No syntax errors found. CIF dictionary Interpreting this report

Datablock: diazoIBr_190

```
Bond precision:  C-C = 0.0040 A          Wavelength=1.54184

Cell:            a=3.97438(5)    b=5.87297(7)        c=35.5177(4)
                 alpha=90        beta=92.1233(10)    gamma=90
Temperature:     190 K

                 Calculated                 Reported
Volume           828.464(17)                828.464(16)
Space group      P 21/n                     P 1 21/n 1
Hall group       -P 2yn                     -P 2yn
Moiety formula   C18 H12 Br I N4            C18 H12 Br I N4
Sum formula      C18 H12 Br I N4            C18 H12 Br I N4
Mr               491.12                     491.13
Dx,g cm-3        1.969                      1.969
Z                2                          2
Mu (mm-1)        18.062                     18.062
F000             472.0                      472.0
F000’            471.11
h,k,lmax         5,7,44                     4,7,44
Nref             1747                       1701
Tmin,Tmax        0.184,0.763                0.248,0.902
Tmin’            0.118

Correction method= # Reported T Limits: Tmin=0.248 Tmax=0.902
AbsCorr = GAUSSIAN

Data completeness= 0.974          Theta(max)= 77.648

R(reflections)= 0.0250( 1608)     wR2(reflections)= 0.0619( 1701)

S = 1.054                  Npar= 119
```

The following ALERTS were generated. Each ALERT has the format
test-name_ALERT_alert-type_alert-level.
Click on the hyperlinks for more details of the test.

Alert level C

PLAT911_ALERT_3_C Missing FCF Refl Between Thmin & STh/L= 0.600 7 Report

Author Response: These reflections were omitted during the absorption correction.

Alert level G

PLAT300_ALERT_4_G Atom Site Occupancy of I1 Constrained at 0.5 Check
PLAT300_ALERT_4_G Atom Site Occupancy of Br1 Constrained at 0.5 Check
PLAT301_ALERT_3_G Main Residue Disorder ....(Resd 1) 8% Note
PLAT910_ALERT_3_G Missing # of FCF Reflection(s) Below Theta(Min). 1 Note
PLAT912_ALERT_4_G Missing # of FCF Reflections Above STh/L= 0.600 32 Note
PLAT933_ALERT_2_G Number of OMIT Records in Embedded .res File ... 2 Note
PLAT978_ALERT_2_G Number C-C Bonds with Positive Residual Density. 6 Info

---

0 **ALERT level A** = Most likely a serious problem - resolve or explain
0 **ALERT level B** = A potentially serious problem, consider carefully
1 **ALERT level C** = Check. Ensure it is not caused by an omission or oversight
7 **ALERT level G** = General information/check it is not something unexpected

0 ALERT type 1 CIF construction/syntax error, inconsistent or missing data
2 ALERT type 2 Indicator that the structure model may be wrong or deficient
3 ALERT type 3 Indicator that the structure quality may be low
3 ALERT type 4 Improvement, methodology, query or suggestion
0 ALERT type 5 Informative message, check

Datablock: diazoIBr_210

Bond precision: C-C = 0.0040 A Wavelength=1.54184

Cell:	a=3.98303(4)	b=5.87894(6)	c=35.5219(4)
	alpha=90	beta=92.1041(9)	gamma=90
Temperature:	210 K		

	Calculated	Reported
Volume	831.220(15)	831.219(15)
Space group	P 21/n	P 1 21/n 1
Hall group	-P 2yn	-P 2yn
Moiety formula	C18 H12 Br I N4	C18 H12 Br I N4
Sum formula	C18 H12 Br I N4	C18 H12 Br I N4
Mr	491.12	491.13
Dx,g cm-3	1.962	1.962
Z	2	2
Mu (mm-1)	18.002	18.002
F000	472.0	472.0
F000’	471.11	
h,k,lmax	5,7,44	4,7,44
Nref	1747	1712
Tmin,Tmax	0.185,0.763	0.252,0.902
Tmin’	0.119	

Correction method= # Reported T Limits: Tmin=0.252 Tmax=0.902
AbsCorr = GAUSSIAN

Data completeness= 0.980 Theta(max)= 77.308

R(reflections)= 0.0246(1621) wR2(reflections)= 0.0629(1712)

S = 1.057 Npar= 119

The following ALERTS were generated. Each ALERT has the format
test-name_ALERT_alert-type_alert-level.
Click on the hyperlinks for more details of the test.

Alert level C

PLAT911_ALERT_3_C Missing FCF Refl Between Thmin & STh/L= 0.600 2 Report

Author Response: These reflections were omitted during the absorption correction.

Alert level G

PLAT300_ALERT_4_G Atom Site Occupancy of I1 Constrained at 0.5 Check
PLAT300_ALERT_4_G Atom Site Occupancy of Br1 Constrained at 0.5 Check
PLAT301_ALERT_3_G Main Residue Disorder ....(Resd 1) 8% Note
PLAT910_ALERT_3_G Missing # of FCF Reflection(s) Below Theta(Min). 1 Note
PLAT912_ALERT_4_G Missing # of FCF Reflections Above STh/L= 0.600 26 Note
PLAT933_ALERT_2_G Number of OMIT Records in Embedded .res File ... 3 Note
PLAT978_ALERT_2_G Number C-C Bonds with Positive Residual Density. 6 Info

---

0 **ALERT level A** = Most likely a serious problem - resolve or explain
0 **ALERT level B** = A potentially serious problem, consider carefully
1 **ALERT level C** = Check. Ensure it is not caused by an omission or oversight
7 **ALERT level G** = General information/check it is not something unexpected

```
   0 ALERT type 1 CIF construction/syntax error, inconsistent or missing data
   2 ALERT type 2 Indicator that the structure model may be wrong or deficient
   3 ALERT type 3 Indicator that the structure quality may be low
   3 ALERT type 4 Improvement, methodology, query or suggestion
   0 ALERT type 5 Informative message, check
```

Datablock: diazoIBr_230

Bond precision: C-C = 0.0040 A Wavelength=1.54184

Cell:	a=3.99201(4)	b=5.88369(7)	c=35.5287(4)
	alpha=90	beta=92.0859(9)	gamma=90
Temperature:	230 K		

	Calculated	Reported
Volume	833.936(16)	833.936(15)
Space group	P 21/n	P 1 21/n 1
Hall group	-P 2yn	-P 2yn
Moiety formula	C18 H12 Br I N4	C18 H12 Br I N4
Sum formula	C18 H12 Br I N4	C18 H12 Br I N4
Mr	491.12	491.13
Dx,g cm-3	1.956	1.956
Z	2	2
Mu (mm-1)	17.943	17.943
F000	472.0	472.0
F000’	471.11	
h,k,lmax	5,7,44	4,7,44
Nref	1758	1706
Tmin,Tmax	0.187,0.764	0.247,0.901
Tmin’	0.119	

Correction method= # Reported T Limits: Tmin=0.247 Tmax=0.901
AbsCorr = GAUSSIAN

Data completeness= 0.970 Theta(max)= 77.520

R(reflections)= 0.0247(1593) wR2(reflections)= 0.0629(1706)

S = 1.065 Npar= 119

The following ALERTS were generated. Each ALERT has the format
test-name_ALERT_alert-type_alert-level.
Click on the hyperlinks for more details of the test.

Alert level C

```
PLAT911_ALERT_3_C Missing FCF Refl Between Thmin & STh/L=    0.600          5 Report
```

Author Response: These reflections were omitted during the absorption correction.

Alert level G

```
PLAT300_ALERT_4_G Atom Site Occupancy of I1         Constrained at        0.5 Check
PLAT300_ALERT_4_G Atom Site Occupancy of Br1        Constrained at        0.5 Check
PLAT301_ALERT_3_G Main Residue  Disorder .............(Resd  1  )          8% Note
PLAT910_ALERT_3_G Missing # of FCF Reflection(s) Below Theta(Min).          1 Note
PLAT912_ALERT_4_G Missing # of FCF Reflections Above STh/L=  0.600         33 Note
PLAT933_ALERT_2_G Number of OMIT Records in Embedded .res File ...          3 Note
PLAT978_ALERT_2_G Number C-C Bonds with Positive Residual Density.          2 Info
```

```
   0 ALERT level A = Most likely a serious problem - resolve or explain
   0 ALERT level B = A potentially serious problem, consider carefully
   1 ALERT level C = Check. Ensure it is not caused by an omission or oversight
   7 ALERT level G = General information/check it is not something unexpected

   0 ALERT type 1 CIF construction/syntax error, inconsistent or missing data
   2 ALERT type 2 Indicator that the structure model may be wrong or deficient
   3 ALERT type 3 Indicator that the structure quality may be low
   3 ALERT type 4 Improvement, methodology, query or suggestion
   0 ALERT type 5 Informative message, check
```

Datablock: diazoIBr_250

Bond precision: C-C = 0.0040 A Wavelength=1.54184

Cell:	a=4.00144(4)	b=5.88842(7)	c=35.5378(4)
	alpha=90	beta=92.0671(9)	gamma=90
Temperature:	250 K		

	Calculated	Reported
Volume	836.803(16)	836.801(16)
Space group	P 21/n	P 1 21/n 1
Hall group	-P 2yn	-P 2yn
Moiety formula	C18 H12 Br I N4	C18 H12 Br I N4
Sum formula	C18 H12 Br I N4	C18 H12 Br I N4
Mr	491.12	491.13
Dx,g cm-3	1.949	1.949
Z	2	2
Mu (mm-1)	17.882	17.882
F000	472.0	472.0
F000’	471.11	
h,k,lmax	5,7,44	4,7,44
Nref	1771	1704
Tmin,Tmax	0.188,0.765	0.247,0.901
Tmin’	0.120	

```
Correction method= # Reported T Limits: Tmin=0.247 Tmax=0.901
AbsCorr = GAUSSIAN

Data completeness= 0.962          Theta(max)= 77.515

R(reflections)= 0.0250( 1589)     wR2(reflections)= 0.0645( 1704)

S = 1.048                   Npar= 119
```

The following ALERTS were generated. Each ALERT has the format
test-name_ALERT_alert-type_alert-level.
Click on the hyperlinks for more details of the test.

Alert level C

```
PLAT911_ALERT_3_C Missing FCF Refl Between Thmin & STh/L=    0.600          7 Report
```

Author Response: These reflections were omitted during the absorption correction.

Alert level G

```
PLAT300_ALERT_4_G Atom Site Occupancy of I1         Constrained at        0.5 Check
PLAT300_ALERT_4_G Atom Site Occupancy of Br1        Constrained at        0.5 Check
PLAT301_ALERT_3_G Main Residue  Disorder ..............(Resd  1  )         8% Note
PLAT910_ALERT_3_G Missing # of FCF Reflection(s) Below Theta(Min).          1 Note
PLAT912_ALERT_4_G Missing # of FCF Reflections Above STh/L=  0.600         44 Note
PLAT933_ALERT_2_G Number of OMIT Records in Embedded .res File ...          4 Note
PLAT978_ALERT_2_G Number C-C Bonds with Positive Residual Density.          5 Info
```

---

0 **ALERT level A** = Most likely a serious problem - resolve or explain
0 **ALERT level B** = A potentially serious problem, consider carefully
1 **ALERT level C** = Check. Ensure it is not caused by an omission or oversight
7 **ALERT level G** = General information/check it is not something unexpected

0 ALERT type 1 CIF construction/syntax error, inconsistent or missing data
2 ALERT type 2 Indicator that the structure model may be wrong or deficient
3 ALERT type 3 Indicator that the structure quality may be low
3 ALERT type 4 Improvement, methodology, query or suggestion
0 ALERT type 5 Informative message, check

Datablock: diazoIBr_270

```
Bond precision:  C-C = 0.0043 A          Wavelength=1.54184

Cell:           a=4.01141(4)    b=5.89288(7)       c=35.5462(4)
                alpha=90        beta=92.0454(9)    gamma=90
Temperature:    270 K
```

	Calculated	Reported
Volume	839.733(16)	839.734(16)
Space group	P 21/n	P 1 21/n 1
Hall group	-P 2yn	-P 2yn
Moiety formula	C18 H12 Br I N4	C18 H12 Br I N4
Sum formula	C18 H12 Br I N4	C18 H12 Br I N4
Mr	491.12	491.13
Dx,g cm-3	1.942	1.942
Z	2	2
Mu (mm-1)	17.819	17.819
F000	472.0	472.0
F000'	471.11	
h,k,lmax	5,7,44	4,7,45
Nref	1777	1704
Tmin,Tmax	0.189,0.765	0.246,0.901
Tmin'	0.121	

Correction method= # Reported T Limits: Tmin=0.246 Tmax=0.901
AbsCorr = GAUSSIAN

Data completeness= 0.959 Theta(max)= 77.571

R(reflections)= 0.0252(1576) wR2(reflections)= 0.0655(1704)

S = 1.047 Npar= 119

The following ALERTS were generated. Each ALERT has the format
test-name_ALERT_alert-type_alert-level.
Click on the hyperlinks for more details of the test.

Alert level C

PLAT911_ALERT_3_C Missing FCF Refl Between Thmin & STh/L= 0.600 3 Report

Author Response: These reflections were omitted during the absorption correction.

Alert level G

PLAT300_ALERT_4_G Atom Site Occupancy of I1 Constrained at 0.5 Check
PLAT300_ALERT_4_G Atom Site Occupancy of Br1 Constrained at 0.5 Check
PLAT301_ALERT_3_G Main Residue Disorder ....(Resd 1) 8% Note
PLAT910_ALERT_3_G Missing # of FCF Reflection(s) Below Theta(Min). 1 Note
PLAT912_ALERT_4_G Missing # of FCF Reflections Above STh/L= 0.600 52 Note
PLAT933_ALERT_2_G Number of OMIT Records in Embedded .res File ... 3 Note
PLAT955_ALERT_1_G Reported (CIF) and Actual (FCF) Lmax Differ by . 1 Units
PLAT978_ALERT_2_G Number C-C Bonds with Positive Residual Density. 4 Info

---

0 **ALERT level A** = Most likely a serious problem - resolve or explain
0 **ALERT level B** = A potentially serious problem, consider carefully
1 **ALERT level C** = Check. Ensure it is not caused by an omission or oversight

8 **ALERT level G** = General information/check it is not something unexpected

1 ALERT type 1 CIF construction/syntax error, inconsistent or missing data
2 ALERT type 2 Indicator that the structure model may be wrong or deficient
3 ALERT type 3 Indicator that the structure quality may be low
3 ALERT type 4 Improvement, methodology, query or suggestion
0 ALERT type 5 Informative message, check

Datablock: diazoIBr_290

Bond precision: C-C = 0.0049 A Wavelength=1.54184

Cell:	a=4.02175(5)	b=5.89808(7)	c=35.5519(4)
	alpha=90	beta=92.0206(10)	gamma=90
Temperature:	290 K		

	Calculated	Reported
Volume	842.788(17)	842.789(17)
Space group	P 21/n	P 1 21/n 1
Hall group	-P 2yn	-P 2yn
Moiety formula	C18 H12 Br I N4	C18 H12 Br I N4
Sum formula	C18 H12 Br I N4	C18 H12 Br I N4
Mr	491.12	491.13
Dx,g cm-3	1.935	1.935
Z	2	2
Mu (mm-1)	17.755	17.755
F000	472.0	472.0
F000’	471.11	
h,k,lmax	5,7,44	4,7,45
Nref	1780	1695
Tmin,Tmax	0.190,0.766	0.245,0.902
Tmin’	0.122	

Correction method= # Reported T Limits: Tmin=0.245 Tmax=0.902
AbsCorr = GAUSSIAN

Data completeness= 0.952 Theta(max)= 77.478

R(reflections)= 0.0263(1551) wR2(reflections)= 0.0667(1695)

S = 1.063 Npar= 119

The following ALERTS were generated. Each ALERT has the format
test-name_ALERT_alert-type_alert-level.
Click on the hyperlinks for more details of the test.

Alert level C

```
PLAT911_ALERT_3_C Missing FCF Refl Between Thmin & STh/L=    0.600          8 Report
```

Author Response: These reflections were omitted during the absorption correction.

Alert level G

```
PLAT300_ALERT_4_G Atom Site Occupancy of I1        Constrained at        0.5 Check
PLAT300_ALERT_4_G Atom Site Occupancy of Br1       Constrained at        0.5 Check
PLAT301_ALERT_3_G Main Residue  Disorder .............(Resd  1  )         8% Note
PLAT910_ALERT_3_G Missing # of FCF Reflection(s) Below Theta(Min).         1 Note
PLAT912_ALERT_4_G Missing # of FCF Reflections Above STh/L=  0.600        57 Note
PLAT933_ALERT_2_G Number of OMIT Records in Embedded .res File ...         4 Note
PLAT955_ALERT_1_G Reported (CIF) and Actual (FCF) Lmax Differ by .         1 Units
PLAT978_ALERT_2_G Number C-C Bonds with Positive Residual Density.         3 Info
```

```
   0 ALERT level A = Most likely a serious problem - resolve or explain
   0 ALERT level B = A potentially serious problem, consider carefully
   1 ALERT level C = Check. Ensure it is not caused by an omission or oversight
   8 ALERT level G = General information/check it is not something unexpected

   1 ALERT type 1 CIF construction/syntax error, inconsistent or missing data
   2 ALERT type 2 Indicator that the structure model may be wrong or deficient
   3 ALERT type 3 Indicator that the structure quality may be low
   3 ALERT type 4 Improvement, methodology, query or suggestion
   0 ALERT type 5 Informative message, check
```

It is advisable to attempt to resolve as many as possible of the alerts in all categories. Often the minor alerts point to easily fixed oversights, errors and omissions in your CIF or refinement strategy, so attention to these fine details can be worthwhile. In order to resolve some of the more serious problems it may be necessary to carry out additional measurements or structure refinements. However, the purpose of your study may justify the reported deviations and the more serious of these should normally be commented upon in the discussion or experimental section of a paper or in the "special_details" fields of the CIF. checkCIF was carefully designed to identify outliers and unusual parameters, but every test has its limitations and alerts that are not important in a particular case may appear. Conversely, the absence of alerts does not guarantee there are no aspects of the results needing attention. It is up to the individual to critically assess their own results and, if necessary, seek expert advice.

Publication of your CIF in IUCr journals

A basic structural check has been run on your CIF. These basic checks will be run on all CIFs submitted for publication in IUCr journals (*Acta Crystallographica*, *Journal of Applied Crystallography*, *Journal of Synchrotron Radiation*); however, if you intend to submit to *Acta Crystallographica Section C* or *E* or *IUCrData*, you should make sure that full publication checks are run on the final version of your CIF prior to submission.

Publication of your CIF in other journals

Please refer to the *Notes for Authors* of the relevant journal for any special instructions relating to CIF submission.

PLATON version of 03/06/2021; check.def file version of 02/06/2021

Datablock diazoIBr_190 - ellipsoid plot

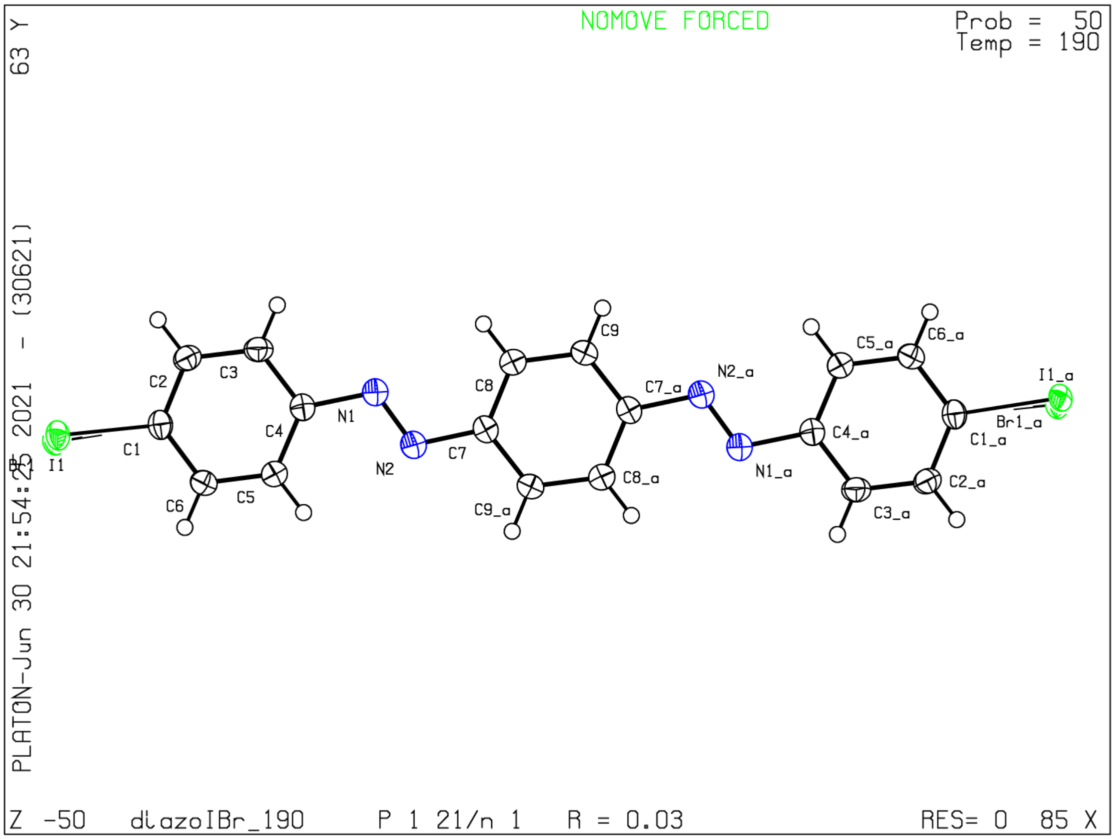

Datablock diazoIBr_210 - ellipsoid plot

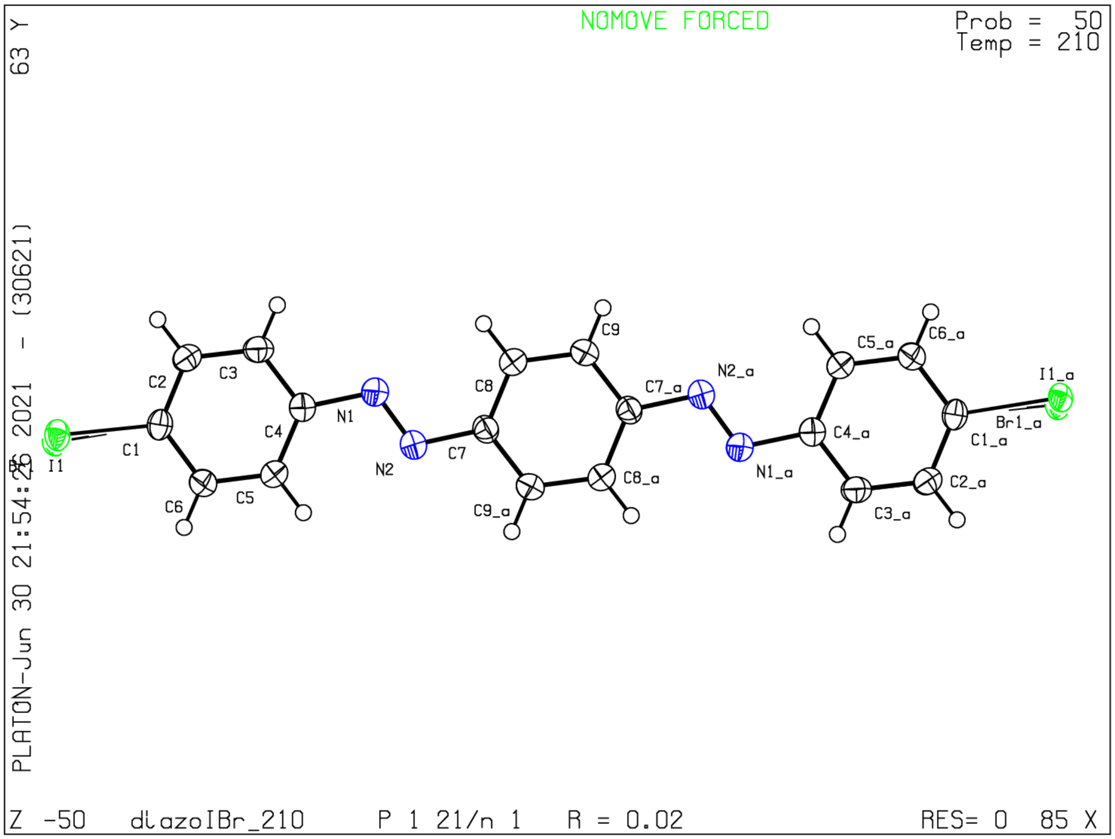

Datablock diazoIBr_230 - ellipsoid plot

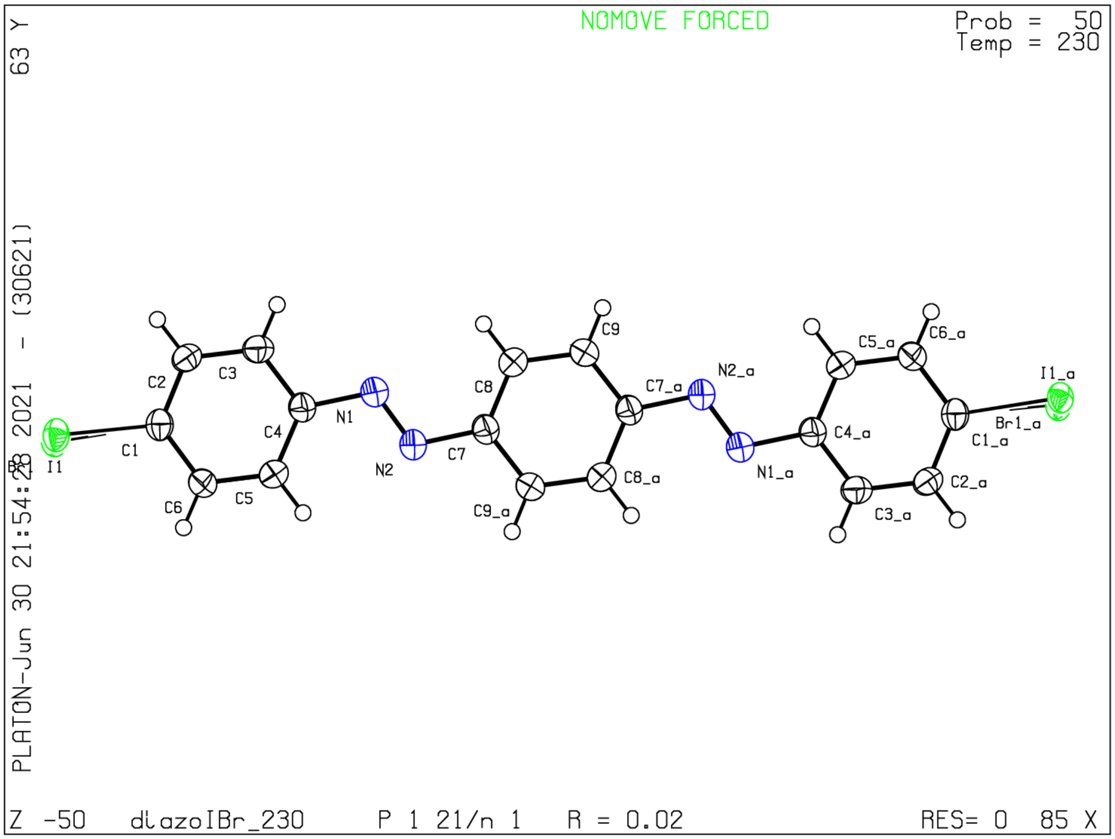

Datablock diazoIBr_250 - ellipsoid plot

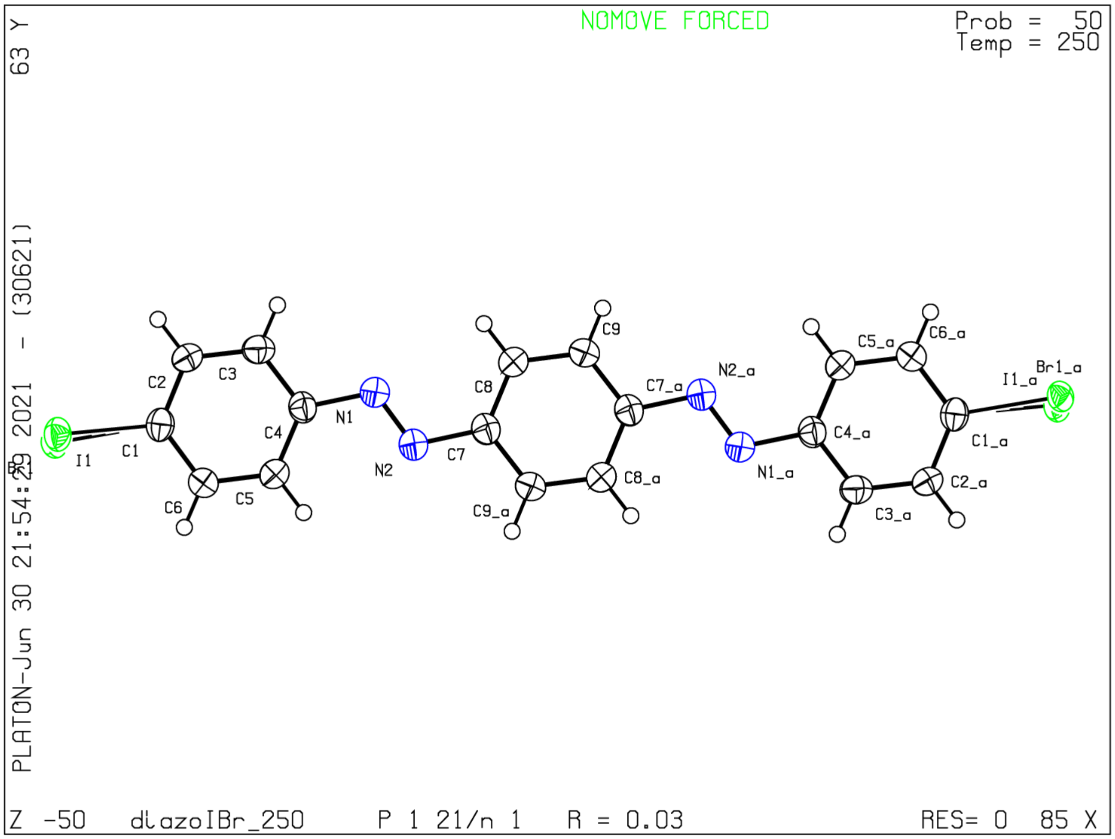

Datablock diazoIBr_270 - ellipsoid plot

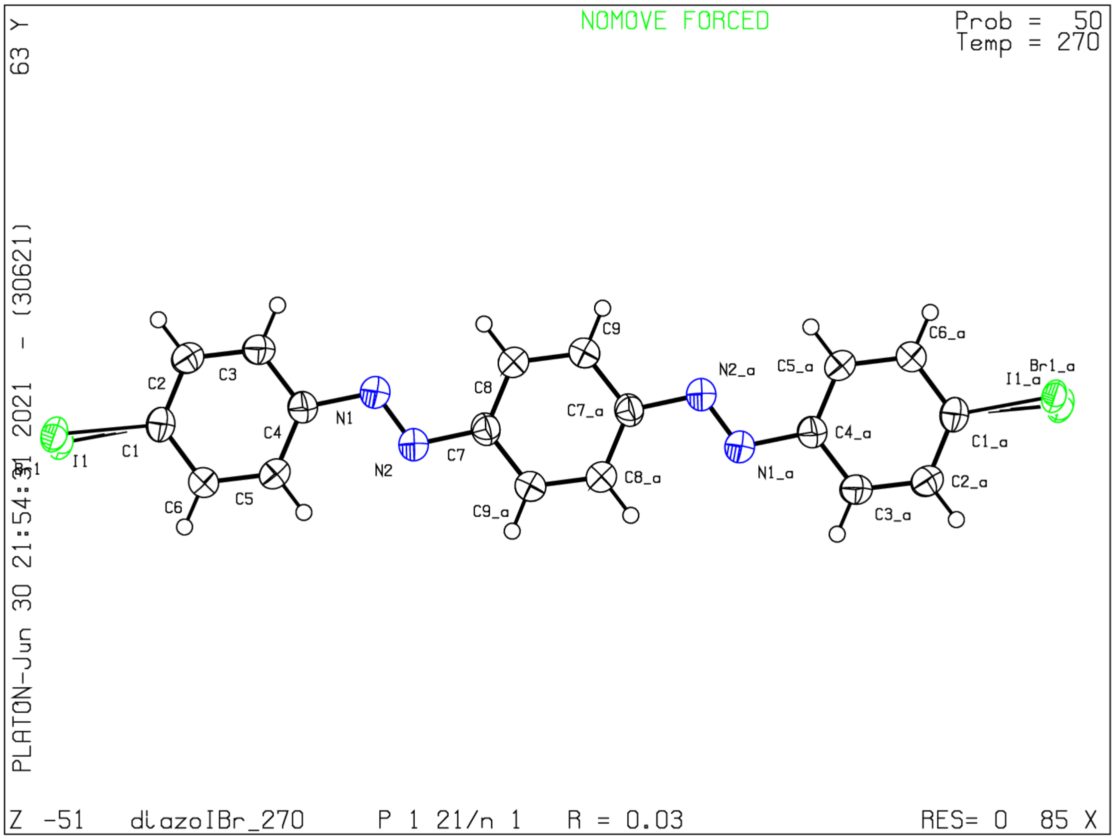

Datablock diazoIBr_290 - ellipsoid plot

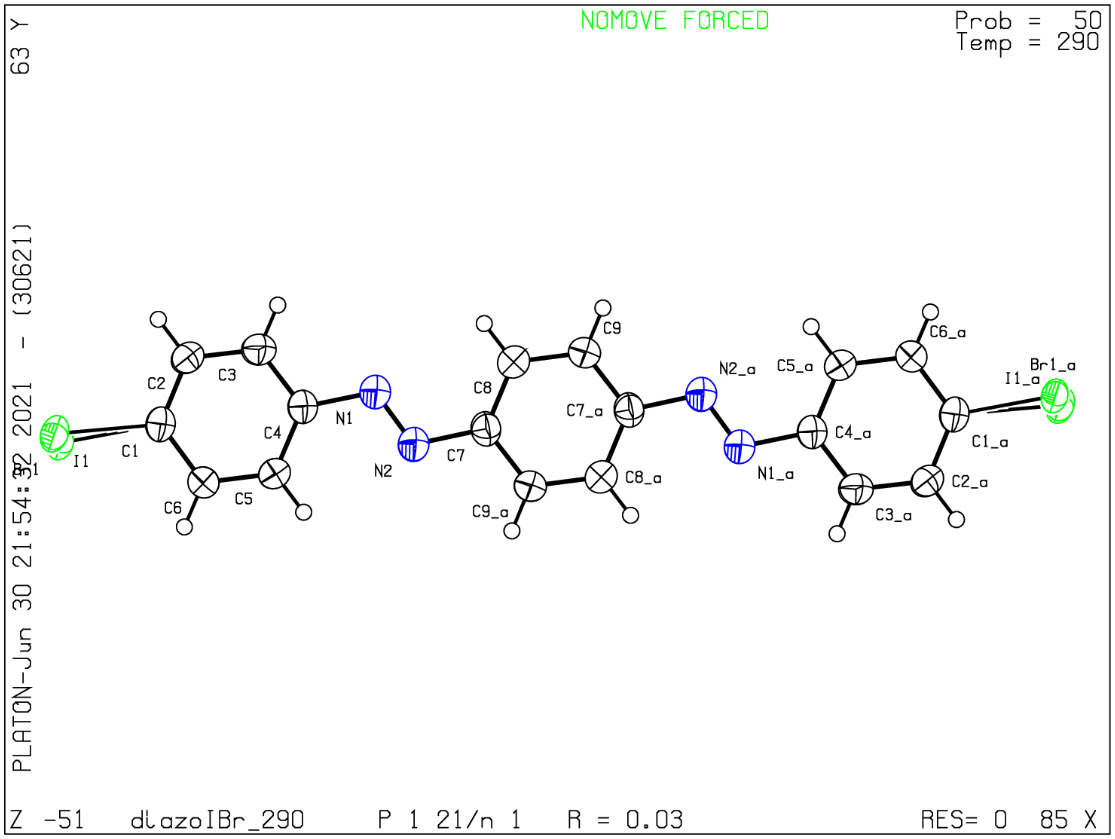

Supplement: Supplementary file 3 [file diazo-I Br.pdf › diazo-I Br.pdf]
