# Supplementary material for: Differences in thermal expansion and motion ability for herringbone and face-to-face π-stacked solids
Source: IUCrJ. 2021 Nov 3;9(Pt 1):31–42. doi: 10.1107/S2052252521009593 (PMC8733877; doi:10.1107/S2052252521009593)

checkCIF/PLATON report

Structure factors have been supplied for datablock(s) diimineBr_190K, diimineBr_210K, diimineBr_230K, diimineBr_250K, diimineBr_270K, diimineBr_290K, diimineI_190K, diimineI_210K, diimineI_230K, diimineI_250K, diimineI_270K, diimineI_290K

THIS REPORT IS FOR GUIDANCE ONLY. IF USED AS PART OF A REVIEW PROCEDURE FOR PUBLICATION, IT SHOULD NOT REPLACE THE EXPERTISE OF AN EXPERIENCED CRYSTALLOGRAPHIC REFEREE.

No syntax errors found.   CIF dictionary   Interpreting this report

Datablock: diimineI_190K

```
Bond precision:  C-C = 0.0050 A          Wavelength=1.54178

Cell:           a=7.42990(9)    b=40.5096(5)        c=5.87627(7)
                alpha=90        beta=90.5291(11)    gamma=90
Temperature:    190 K

                Calculated                Reported
Volume          1768.58(4)                1768.58(4)
Space group     P 21/c                    P 21/c
Hall group      -P 2ybc                   -P 2ybc
Moiety formula  C20 H14 I2 N2             ?
Sum formula     C20 H14 I2 N2             C20 H14 I2 N2
Mr              536.13                    536.13
Dx,g cm-3       2.013                     2.014
Z               4                         4
Mu (mm-1)       27.944                    27.944
F000            1016.0                    1016.0
F000'           1016.51
h,k,lmax        9,51,7                    9,50,7
Nref            3762                      3699
Tmin,Tmax       0.022,0.198               0.421,0.944
Tmin'           0.002

Correction method= # Reported T Limits: Tmin=0.421 Tmax=0.944
AbsCorr = GAUSSIAN

Data completeness= 0.983          Theta(max)= 77.467

R(reflections)= 0.0383( 3412)     wR2(reflections)= 0.1041( 3699)

S = 1.070                   Npar= 217
```

The following ALERTS were generated. Each ALERT has the format
test-name_ALERT_alert-type_alert-level.
Click on the hyperlinks for more details of the test.

Alert level C

PLAT971_ALERT_2_C Check Calcd Resid. Dens. 1.05A From C1 1.75 eA-3

Author Response: Due to absorption correction issues at this temperature.

PLAT971_ALERT_2_C Check Calcd Resid. Dens. 1.34A From I1 1.64 eA-3

Author Response: Due to absorption correction issues at this temperature.

PLAT971_ALERT_2_C Check Calcd Resid. Dens. 1.09A From I2 1.57 eA-3

Author Response: Due to absorption correction issues at this temperature.

PLAT972_ALERT_2_C Check Calcd Resid. Dens. 0.87A From I1 -2.01 eA-3

Author Response: Due to absorption correction issues at this temperature.

PLAT972_ALERT_2_C Check Calcd Resid. Dens. 0.94A From I1 -1.89 eA-3

Author Response: Due to absorption correction issues at this temperature.

PLAT972_ALERT_2_C Check Calcd Resid. Dens. 0.89A From I1 -1.78 eA-3

Author Response: Due to absorption correction issues at this temperature.

PLAT972_ALERT_2_C Check Calcd Resid. Dens. 1.00A From I2 -1.77 eA-3

Author Response: Due to absorption correction issues at this temperature.

PLAT972_ALERT_2_C Check Calcd Resid. Dens. 1.00A From I2 -1.74 eA-3

Author Response: Due to absorption correction issues at this temperature.

PLAT972_ALERT_2_C Check Calcd Resid. Dens. 0.88A From I1 -1.68 eA-3

Author Response: Due to absorption correction issues at this temperature.

PLAT972_ALERT_2_C Check Calcd Resid. Dens. 1.05A From I2 -1.66 eA-3

Author Response: Due to absorption correction issues at this temperature.

```
PLAT972_ALERT_2_C Check Calcd Resid. Dens.  1.02A   From I2            -1.62 eA-3
```

Author Response: Due to absorption correction issues at this temperature.

Alert level G

```
PLAT912_ALERT_4_G Missing # of FCF Reflections Above STh/L=  0.600         61 Note
PLAT978_ALERT_2_G Number C-C Bonds with Positive Residual Density.          2 Info
```

```
   0 ALERT level A = Most likely a serious problem - resolve or explain
   0 ALERT level B = A potentially serious problem, consider carefully
  11 ALERT level C = Check. Ensure it is not caused by an omission or oversight
   2 ALERT level G = General information/check it is not something unexpected

   0 ALERT type 1 CIF construction/syntax error, inconsistent or missing data
  12 ALERT type 2 Indicator that the structure model may be wrong or deficient
   0 ALERT type 3 Indicator that the structure quality may be low
   1 ALERT type 4 Improvement, methodology, query or suggestion
   0 ALERT type 5 Informative message, check
```

Datablock: diimineI_210K

```
Bond precision:  C-C = 0.0050 A          Wavelength=1.54178

Cell:           a=7.44656(9)    b=40.5029(5)       c=5.88383(6)
                alpha=90        beta=90.5288(11)    gamma=90
Temperature:    210 K

                Calculated                  Reported
Volume          1774.53(4)                  1774.53(4)
Space group     P 21/c                      P 21/c
Hall group      -P 2ybc                     -P 2ybc
Moiety formula  C20 H14 I2 N2               ?
Sum formula     C20 H14 I2 N2               C20 H14 I2 N2
Mr              536.13                      536.13
Dx,g cm-3       2.007                       2.007
Z               4                           4
Mu (mm-1)       27.850                      27.850
F000            1016.0                      1016.0
F000'           1016.51
h,k,lmax        9,51,7                      9,51,7
Nref            3779                        3719
Tmin,Tmax       0.022,0.199                 0.420,0.931
Tmin'           0.002

Correction method= # Reported T Limits: Tmin=0.420 Tmax=0.931
AbsCorr = GAUSSIAN
```

Data completeness= 0.984   Theta(max)= 77.475

R(reflections)= 0.0378(3408)   wR2(reflections)= 0.1014(3719)

S = 1.034   Npar= 217

The following ALERTS were generated. Each ALERT has the format
test-name_ALERT_alert-type_alert-level.
Click on the hyperlinks for more details of the test.

Alert level C

PLAT971_ALERT_2_C Check Calcd Resid. Dens. 1.04A From C1 1.65 eA-3

Author Response: Due to absorption correction issues at this temperature.

PLAT972_ALERT_2_C Check Calcd Resid. Dens. 0.86A From I1 -1.92 eA-3

Author Response: Due to absorption correction issues at this temperature.

PLAT972_ALERT_2_C Check Calcd Resid. Dens. 0.94A From I1 -1.84 eA-3

Author Response: Due to absorption correction issues at this temperature.

PLAT972_ALERT_2_C Check Calcd Resid. Dens. 0.87A From I1 -1.66 eA-3

Author Response: Due to absorption correction issues at this temperature.

PLAT972_ALERT_2_C Check Calcd Resid. Dens. 0.98A From I1 -1.63 eA-3

Author Response: Due to absorption correction issues at this temperature.

PLAT972_ALERT_2_C Check Calcd Resid. Dens. 1.01A From I2 -1.61 eA-3

Author Response: Due to absorption correction issues at this temperature.

PLAT972_ALERT_2_C Check Calcd Resid. Dens. 1.06A From I2 -1.61 eA-3

Author Response: Due to absorption correction issues at this temperature.

PLAT972_ALERT_2_C Check Calcd Resid. Dens. 0.74A From I2 -1.60 eA-3

Author Response: Due to absorption correction issues at this temperature.

PLAT972_ALERT_2_C Check Calcd Resid. Dens. 1.03A From I2 -1.53 eA-3

Author Response: Due to absorption correction issues at this temperature.

Alert level G

```
PLAT912_ALERT_4_G Missing # of FCF Reflections Above STh/L=  0.600         60 Note
PLAT954_ALERT_1_G Reported (CIF) and Actual (FCF) Kmax Differ by .          1 Units
PLAT978_ALERT_2_G Number C-C Bonds with Positive Residual Density.          6 Info
```

```
   0 ALERT level A = Most likely a serious problem - resolve or explain
   0 ALERT level B = A potentially serious problem, consider carefully
   9 ALERT level C = Check. Ensure it is not caused by an omission or oversight
   3 ALERT level G = General information/check it is not something unexpected

   1 ALERT type 1 CIF construction/syntax error, inconsistent or missing data
  10 ALERT type 2 Indicator that the structure model may be wrong or deficient
   0 ALERT type 3 Indicator that the structure quality may be low
   1 ALERT type 4 Improvement, methodology, query or suggestion
   0 ALERT type 5 Informative message, check
```

Datablock: diimineI_230K

Bond precision: C-C = 0.0050 A Wavelength=1.54178

Cell:	a=7.46346(10)	b=40.4985(5)	c=5.89180(7)
	alpha=90	beta=90.5284(11)	gamma=90
Temperature:	230 K		

	Calculated	Reported
Volume	1780.77(4)	1780.78(4)
Space group	P 21/c	P 21/c
Hall group	-P 2ybc	-P 2ybc
Moiety formula	C20 H14 I2 N2	?
Sum formula	C20 H14 I2 N2	C20 H14 I2 N2
Mr	536.13	536.13
Dx,g cm-3	2.000	2.000
Z	4	4
Mu (mm-1)	27.753	27.753
F000	1016.0	1016.0
F000’	1016.51	
h,k,lmax	9,51,7	9,51,7
Nref	3789	3729
Tmin,Tmax	0.023,0.200	0.420,0.916
Tmin’	0.002	

Correction method= # Reported T Limits: Tmin=0.420 Tmax=0.916
AbsCorr = GAUSSIAN

Data completeness= 0.984 Theta(max)= 77.352

```
R(reflections)= 0.0377( 3388)     wR2(reflections)= 0.0977( 3729)

S = 1.066                     Npar= 217
```

The following ALERTS were generated. Each ALERT has the format
test-name_ALERT_alert-type_alert-level.
Click on the hyperlinks for more details of the test.

Alert level C

```
PLAT972_ALERT_2_C Check Calcd Resid. Dens.  0.86A   From I1            -1.72 eA-3
```

Author Response: Due to absorption correction issues at this temperature.

```
PLAT972_ALERT_2_C Check Calcd Resid. Dens.  0.90A   From I1            -1.67 eA-3
```

Author Response: Due to absorption correction issues at this temperature.

```
PLAT972_ALERT_2_C Check Calcd Resid. Dens.  0.82A   From I1            -1.57 eA-3
```

Author Response: Due to absorption correction issues at this temperature.

```
PLAT972_ALERT_2_C Check Calcd Resid. Dens.  1.02A   From I2            -1.52 eA-3
```

Author Response: Due to absorption correction issues at this temperature.

```
PLAT972_ALERT_2_C Check Calcd Resid. Dens.  0.75A   From I2            -1.51 eA-3
```

Author Response: Due to absorption correction issues at this temperature.

Alert level G

```
PLAT912_ALERT_4_G Missing # of FCF Reflections Above STh/L=  0.600        61 Note
PLAT954_ALERT_1_G Reported (CIF) and Actual (FCF) Kmax Differ by .         1 Units
PLAT978_ALERT_2_G Number C-C Bonds with Positive Residual Density.         3 Info
```

```
   0 ALERT level A = Most likely a serious problem - resolve or explain
   0 ALERT level B = A potentially serious problem, consider carefully
   5 ALERT level C = Check. Ensure it is not caused by an omission or oversight
   3 ALERT level G = General information/check it is not something unexpected

   1 ALERT type 1 CIF construction/syntax error, inconsistent or missing data
   6 ALERT type 2 Indicator that the structure model may be wrong or deficient
   0 ALERT type 3 Indicator that the structure quality may be low
   1 ALERT type 4 Improvement, methodology, query or suggestion
   0 ALERT type 5 Informative message, check
```

Datablock: diimineI_250K

Bond precision: C-C = 0.0049 A Wavelength=1.54178

Cell:	a=7.48192(10)	b=40.4930(5)	c=5.90037(7)
	alpha=90	beta=90.4998(12)	gamma=90
Temperature:	250 K		

	Calculated	Reported
Volume	1787.54(4)	1787.54(4)
Space group	P 21/c	P 21/c
Hall group	-P 2ybc	-P 2ybc
Moiety formula	C20 H14 I2 N2	?
Sum formula	C20 H14 I2 N2	C20 H14 I2 N2
Mr	536.13	536.13
Dx,g cm-3	1.992	1.992
Z	4	4
Mu (mm-1)	27.648	27.648
F000	1016.0	1016.0
F000’	1016.51	
h,k,lmax	9,51,7	9,50,7
Nref	3815	3759
Tmin,Tmax	0.023,0.201	0.423,0.919
Tmin’	0.002	

Correction method= # Reported T Limits: Tmin=0.423 Tmax=0.919
AbsCorr = GAUSSIAN

Data completeness= 0.985 Theta(max)= 77.529

R(reflections)= 0.0363(3359) wR2(reflections)= 0.0968(3759)

S = 1.029 Npar= 217

The following ALERTS were generated. Each ALERT has the format
test-name_ALERT_alert-type_alert-level.
Click on the hyperlinks for more details of the test.

Alert level G

PLAT912_ALERT_4_G Missing # of FCF Reflections Above STh/L= 0.600 56 Note
PLAT978_ALERT_2_G Number C-C Bonds with Positive Residual Density. 1 Info

---

0 **ALERT level A** = Most likely a serious problem - resolve or explain
0 **ALERT level B** = A potentially serious problem, consider carefully
0 **ALERT level C** = Check. Ensure it is not caused by an omission or oversight
2 **ALERT level G** = General information/check it is not something unexpected

0 ALERT type 1 CIF construction/syntax error, inconsistent or missing data
1 ALERT type 2 Indicator that the structure model may be wrong or deficient
0 ALERT type 3 Indicator that the structure quality may be low
1 ALERT type 4 Improvement, methodology, query or suggestion
0 ALERT type 5 Informative message, check

Datablock: diimineI_270K

Bond precision: C-C = 0.0050 A Wavelength=1.54178

Cell:	a=7.4993(3)	b=40.4748(15)	c=5.9105(2)
	alpha=90	beta=90.496(4)	gamma=90
Temperature:	270 K		

	Calculated	Reported
Volume	1793.96(12)	1793.94(12)
Space group	P 21/c	P 21/c
Hall group	-P 2ybc	-P 2ybc
Moiety formula	C20 H14 I2 N2	?
Sum formula	C20 H14 I2 N2	C20 H14 I2 N2
Mr	536.13	536.13
Dx,g cm-3	1.985	1.985
Z	4	4
Mu (mm-1)	27.549	27.549
F000	1016.0	1016.0
F000'	1016.51	
h,k,lmax	9,51,7	9,50,7
Nref	3817	3719
Tmin,Tmax	0.024,0.202	0.425,0.909
Tmin'	0.002	

Correction method= # Reported T Limits: Tmin=0.425 Tmax=0.909
AbsCorr = GAUSSIAN

Data completeness= 0.974 Theta(max)= 77.268

R(reflections)= 0.0345(3206) wR2(reflections)= 0.0941(3719)

S = 1.042 Npar= 217

The following ALERTS were generated. Each ALERT has the format
test-name_ALERT_alert-type_alert-level.
Click on the hyperlinks for more details of the test.

Alert level C

PLAT911_ALERT_3_C Missing FCF Refl Between Thmin & STh/L= 0.600 5 Report

Alert level G

PLAT912_ALERT_4_G Missing # of FCF Reflections Above STh/L= 0.600 93 Note
PLAT978_ALERT_2_G Number C-C Bonds with Positive Residual Density. 2 Info

0 **ALERT level A** = Most likely a serious problem - resolve or explain
0 **ALERT level B** = A potentially serious problem, consider carefully
1 **ALERT level C** = Check. Ensure it is not caused by an omission or oversight
2 **ALERT level G** = General information/check it is not something unexpected

0 ALERT type 1 CIF construction/syntax error, inconsistent or missing data
1 ALERT type 2 Indicator that the structure model may be wrong or deficient
1 ALERT type 3 Indicator that the structure quality may be low
1 ALERT type 4 Improvement, methodology, query or suggestion
0 ALERT type 5 Informative message, check

Datablock: diimineI_290K

Bond precision: C-C = 0.0065 A Wavelength=1.54178

Cell:	a=7.51877(11)	b=40.4776(5)	c=5.91959(8)
	alpha=90	beta=90.5114(13)	gamma=90
Temperature:	290 K		

	Calculated	Reported
Volume	1801.51(4)	1801.51(4)
Space group	P 21/c	P 21/c
Hall group	-P 2ybc	-P 2ybc
Moiety formula	C20 H14 I2 N2	?
Sum formula	C20 H14 I2 N2	C20 H14 I2 N2
Mr	536.13	536.13
Dx,g cm-3	1.977	1.977
Z	4	4
Mu (mm-1)	27.433	27.433
F000	1016.0	1016.0
F000’	1016.51	
h,k,lmax	9,51,7	9,50,7
Nref	3842	3780
Tmin,Tmax	0.024,0.204	0.424,0.933
Tmin’	0.002	

Correction method= # Reported T Limits: Tmin=0.424 Tmax=0.933
AbsCorr = GAUSSIAN

Data completeness= 0.984 Theta(max)= 77.486

R(reflections)= 0.0487(3199) wR2(reflections)= 0.1326(3780)

S = 1.054 Npar= 217

```
The following ALERTS were generated. Each ALERT has the format
       test-name_ALERT_alert-type_alert-level.
Click on the hyperlinks for more details of the test.
```

Alert level C

```
PLAT906_ALERT_3_C Large K Value in the Analysis of Variance ......      4.783 Check
```

Author Response: Due to weak diffuse scatter around Bragg peaks.

Alert level G

```
PLAT912_ALERT_4_G Missing # of FCF Reflections Above STh/L=  0.600         63 Note
PLAT978_ALERT_2_G Number C-C Bonds with Positive Residual Density.          1 Info
PLAT992_ALERT_5_G Repd & Actual _reflns_number_gt Values Differ by          2 Check
```

```
   0 ALERT level A = Most likely a serious problem - resolve or explain
   0 ALERT level B = A potentially serious problem, consider carefully
   1 ALERT level C = Check. Ensure it is not caused by an omission or oversight
   3 ALERT level G = General information/check it is not something unexpected

   0 ALERT type 1 CIF construction/syntax error, inconsistent or missing data
   1 ALERT type 2 Indicator that the structure model may be wrong or deficient
   1 ALERT type 3 Indicator that the structure quality may be low
   1 ALERT type 4 Improvement, methodology, query or suggestion
   1 ALERT type 5 Informative message, check
```

Datablock: diimineBr_190K

```
Bond precision:  C-C = 0.0070 A          Wavelength=1.54178

Cell:            a=7.38842(9)     b=38.9510(4)      c=5.89251(5)
                 alpha=90         beta=90.428(1)    gamma=90
Temperature:     190 K
```

	Calculated	Reported
Volume	1695.74(3)	1695.74(3)
Space group	P 21/c	P 21/c
Hall group	-P 2ybc	-P 2ybc
Moiety formula	C20 H14 Br2 N2	?
Sum formula	C20 H14 Br2 N2	C20 H14 Br2 N2
Mr	442.13	442.15
Dx,g cm-3	1.732	1.732
Z	4	4
Mu (mm-1)	6.075	6.075
F000	872.0	872.0
F000’	868.19	
h,k,lmax	9,49,7	9,49,7
Nref	3604	3537
Tmin,Tmax	0.299,0.896	0.320,1.000
Tmin’	0.103	

Correction method= # Reported T Limits: Tmin=0.320 Tmax=1.000
AbsCorr = GAUSSIAN

Data completeness= 0.981 Theta(max)= 77.401

R(reflections)= 0.0527(3238) wR2(reflections)= 0.1305(3537)

S = 1.093 Npar= 217

The following ALERTS were generated. Each ALERT has the format
test-name_ALERT_alert-type_alert-level.
Click on the hyperlinks for more details of the test.

Alert level C

PLAT341_ALERT_3_C Low Bond Precision on C-C Bonds .... 0.007 Ang.

Author Response: Due to the weak diffraction of high angle reflections.

PLAT906_ALERT_3_C Large K Value in the Analysis of Variance ...... 7.132 Check

Author Response: Due to weak high angle data and diffuse scattering about Bragg peaks that was not modeled.

PLAT911_ALERT_3_C Missing FCF Refl Between Thmin & STh/L= 0.600 8 Report

---

Alert level G

PLAT083_ALERT_2_G SHELXL Second Parameter in WGHT Unusually Large 12.53 Why ?
PLAT143_ALERT_4_G s.u. on c - Axis Small or Missing .... 0.00005 Ang.
PLAT912_ALERT_4_G Missing # of FCF Reflections Above STh/L= 0.600 59 Note
PLAT933_ALERT_2_G Number of OMIT Records in Embedded .res File ... 3 Note
PLAT954_ALERT_1_G Reported (CIF) and Actual (FCF) Kmax Differ by . 1 Units

PLAT978_ALERT_2_G Number C-C Bonds with Positive Residual Density. 2 Info

---

0 **ALERT level A** = Most likely a serious problem - resolve or explain
0 **ALERT level B** = A potentially serious problem, consider carefully
3 **ALERT level C** = Check. Ensure it is not caused by an omission or oversight
6 **ALERT level G** = General information/check it is not something unexpected

1 ALERT type 1 CIF construction/syntax error, inconsistent or missing data
3 ALERT type 2 Indicator that the structure model may be wrong or deficient
3 ALERT type 3 Indicator that the structure quality may be low
2 ALERT type 4 Improvement, methodology, query or suggestion
0 ALERT type 5 Informative message, check

Datablock: diimineBr_210K

Bond precision: C-C = 0.0060 A Wavelength=1.54178

Cell:	a=7.40704(9)	b=38.9343(3)	c=5.90140(6)
	alpha=90	beta=90.4197(10)	gamma=90
Temperature:	210 K		

	Calculated	Reported
Volume	1701.85(3)	1701.85(3)
Space group	P 21/c	P 21/c
Hall group	-P 2ybc	-P 2ybc
Moiety formula	C20 H14 Br2 N2	?
Sum formula	C20 H14 Br2 N2	C20 H14 Br2 N2
Mr	442.13	442.15
Dx,g cm-3	1.726	1.726
Z	4	4
Mu (mm-1)	6.053	6.053
F000	872.0	872.0
F000’	868.19	
h,k,lmax	9,49,7	9,49,7
Nref	3621	3552
Tmin,Tmax	0.300,0.897	0.320,1.000
Tmin’	0.104	

Correction method= # Reported T Limits: Tmin=0.320 Tmax=1.000
AbsCorr = GAUSSIAN

Data completeness= 0.981 Theta(max)= 77.368

R(reflections)= 0.0481(3225) wR2(reflections)= 0.1373(3552)

S = 1.101 Npar= 217

The following ALERTS were generated. Each ALERT has the format
test-name_ALERT_alert-type_alert-level.
Click on the hyperlinks for more details of the test.

Alert level C

```
PLAT906_ALERT_3_C Large K Value in the Analysis of Variance ......      3.612 Check
```

Author Response: Due to weak high angle data and diffuse scattering about Bragg peaks that was not modeled.

```
PLAT911_ALERT_3_C Missing FCF Refl Between Thmin & STh/L=    0.600          9 Report
```

Alert level G

```
PLAT083_ALERT_2_G SHELXL Second Parameter in WGHT  Unusually Large       6.50 Why ?
PLAT142_ALERT_4_G s.u. on b - Axis Small or Missing .............    0.00030 Ang.
PLAT912_ALERT_4_G Missing # of FCF Reflections Above STh/L=  0.600         59 Note
PLAT933_ALERT_2_G Number of OMIT Records in Embedded .res File ...          4 Note
PLAT954_ALERT_1_G Reported (CIF) and Actual (FCF) Kmax Differ by .          1 Units
PLAT978_ALERT_2_G Number C-C Bonds with Positive Residual Density.          5 Info
```

0 **ALERT level A** = Most likely a serious problem - resolve or explain
0 **ALERT level B** = A potentially serious problem, consider carefully
2 **ALERT level C** = Check. Ensure it is not caused by an omission or oversight
6 **ALERT level G** = General information/check it is not something unexpected

1 ALERT type 1 CIF construction/syntax error, inconsistent or missing data
3 ALERT type 2 Indicator that the structure model may be wrong or deficient
2 ALERT type 3 Indicator that the structure quality may be low
2 ALERT type 4 Improvement, methodology, query or suggestion
0 ALERT type 5 Informative message, check

Datablock: diimineBr_230K

Bond precision:	C-C = 0.0062 A		Wavelength=1.54178
Cell:	a=7.42568(9)	b=38.9298(4)	c=5.90945(6)
	alpha=90	beta=90.4212(10)	gamma=90
Temperature:	230 K		

	Calculated	Reported
Volume	1708.26(3)	1708.26(3)
Space group	P 21/c	P 21/c
Hall group	-P 2ybc	-P 2ybc
Moiety formula	C20 H14 Br2 N2	?
Sum formula	C20 H14 Br2 N2	C20 H14 Br2 N2
Mr	442.13	442.15
Dx,g cm-3	1.719	1.719
Z	4	4
Mu (mm-1)	6.030	6.030
F000	872.0	872.0
F000’	868.19	
h,k,lmax	9,49,7	9,49,7
Nref	3640	3567
Tmin,Tmax	0.302,0.897	0.319,1.000
Tmin’	0.105	

Correction method= # Reported T Limits: Tmin=0.319 Tmax=1.000
AbsCorr = GAUSSIAN

Data completeness= 0.980 Theta(max)= 77.477

R(reflections)= 0.0504(3194) wR2(reflections)= 0.1435(3567)

S = 1.103 Npar= 217

The following ALERTS were generated. Each ALERT has the format
test-name_ALERT_alert-type_alert-level.
Click on the hyperlinks for more details of the test.

Alert level C

PLAT341_ALERT_3_C Low Bond Precision on C-C Bonds .... 0.0062 Ang.

Author Response: Due to the weak diffraction of high angle reflections.

PLAT906_ALERT_3_C Large K Value in the Analysis of Variance ...... 3.780 Check

Author Response: Due to weak high angle data and diffuse scattering about Bragg peaks that was not modeled.

PLAT911_ALERT_3_C Missing FCF Refl Between Thmin & STh/L= 0.600 10 Report

---

Alert level G

PLAT083_ALERT_2_G SHELXL Second Parameter in WGHT Unusually Large 6.03 Why ?
PLAT912_ALERT_4_G Missing # of FCF Reflections Above STh/L= 0.600 63 Note
PLAT933_ALERT_2_G Number of OMIT Records in Embedded .res File ... 5 Note
PLAT954_ALERT_1_G Reported (CIF) and Actual (FCF) Kmax Differ by . 1 Units
PLAT978_ALERT_2_G Number C-C Bonds with Positive Residual Density. 5 Info

0 **ALERT level A** = Most likely a serious problem - resolve or explain
0 **ALERT level B** = A potentially serious problem, consider carefully
3 **ALERT level C** = Check. Ensure it is not caused by an omission or oversight
5 **ALERT level G** = General information/check it is not something unexpected

1 ALERT type 1 CIF construction/syntax error, inconsistent or missing data
3 ALERT type 2 Indicator that the structure model may be wrong or deficient
3 ALERT type 3 Indicator that the structure quality may be low
1 ALERT type 4 Improvement, methodology, query or suggestion
0 ALERT type 5 Informative message, check

Datablock: diimineBr_250K

Bond precision: C-C = 0.0060 A Wavelength=1.54178

Cell:	a=7.44522(10)	b=38.9215(4)	c=5.91739(6)
	alpha=90	beta=90.4009(11)	gamma=90
Temperature:	250 K		

	Calculated	Reported
Volume	1714.69(3)	1714.69(3)
Space group	P 21/c	P 21/c
Hall group	-P 2ybc	-P 2ybc
Moiety formula	C20 H14 Br2 N2	?
Sum formula	C20 H14 Br2 N2	C20 H14 Br2 N2
Mr	442.13	442.15
Dx,g cm-3	1.713	1.713
Z	4	4
Mu (mm-1)	6.008	6.008
F000	872.0	872.0
F000’	868.19	
h,k,lmax	9,49,7	9,49,7
Nref	3655	3585
Tmin,Tmax	0.303,0.897	0.323,1.000
Tmin’	0.106	

Correction method= # Reported T Limits: Tmin=0.323 Tmax=1.000
AbsCorr = GAUSSIAN

Data completeness= 0.981 Theta(max)= 77.454

R(reflections)= 0.0498(3139) wR2(reflections)= 0.1400(3585)

S = 1.063 Npar= 217

The following ALERTS were generated. Each ALERT has the format
test-name_ALERT_alert-type_alert-level.
Click on the hyperlinks for more details of the test.

Alert level C

```
PLAT906_ALERT_3_C Large K Value in the Analysis of Variance ......      3.662 Check
```

Author Response: Due to weak high angle data and diffuse scattering about Bragg peaks that was not modeled.

```
PLAT911_ALERT_3_C Missing FCF Refl Between Thmin & STh/L=    0.600          9 Report
```

Alert level G

```
PLAT083_ALERT_2_G SHELXL Second Parameter in WGHT  Unusually Large       5.26 Why ?
PLAT912_ALERT_4_G Missing # of FCF Reflections Above STh/L=  0.600         61 Note
PLAT933_ALERT_2_G Number of OMIT Records in Embedded .res File ...          5 Note
PLAT954_ALERT_1_G Reported (CIF) and Actual (FCF) Kmax Differ by .          1 Units
PLAT978_ALERT_2_G Number C-C Bonds with Positive Residual Density.          5 Info
```

---

0 **ALERT level A** = Most likely a serious problem - resolve or explain
0 **ALERT level B** = A potentially serious problem, consider carefully
2 **ALERT level C** = Check. Ensure it is not caused by an omission or oversight
5 **ALERT level G** = General information/check it is not something unexpected

1 ALERT type 1 CIF construction/syntax error, inconsistent or missing data
3 ALERT type 2 Indicator that the structure model may be wrong or deficient
2 ALERT type 3 Indicator that the structure quality may be low
1 ALERT type 4 Improvement, methodology, query or suggestion
0 ALERT type 5 Informative message, check

Datablock: diimineBr_270K

Bond precision: C-C = 0.0060 A   Wavelength=1.54178

Cell:	a=7.46626(15)	b=38.9089(5)	c=5.92704(9)
	alpha=90	beta=90.4136(17)	gamma=90
Temperature:	270 K		

	Calculated	Reported
Volume	1721.78(5)	1721.78(5)
Space group	P 21/c	P 21/c
Hall group	-P 2ybc	-P 2ybc
Moiety formula	C20 H14 Br2 N2	?
Sum formula	C20 H14 Br2 N2	C20 H14 Br2 N2
Mr	442.13	442.15
Dx,g cm-3	1.706	1.706
Z	4	4
Mu (mm-1)	5.983	5.983
F000	872.0	872.0
F000’	868.19	
h,k,lmax	9,49,7	9,49,7
Nref	3663	3585
Tmin,Tmax	0.305,0.898	0.306,1.000
Tmin’	0.106	

Correction method= # Reported T Limits: Tmin=0.306 Tmax=1.000
AbsCorr = GAUSSIAN

Data completeness= 0.979 Theta(max)= 77.327

R(reflections)= 0.0516(2976) wR2(reflections)= 0.1457(3585)

S = 1.085 Npar= 217

The following ALERTS were generated. Each ALERT has the format
test-name_ALERT_alert-type_alert-level.
Click on the hyperlinks for more details of the test.

Alert level C

PLAT341_ALERT_3_C Low Bond Precision on C-C Bonds .... 0.00605 Ang.

Author Response: Due to the weak diffraction of high angle reflections.

PLAT906_ALERT_3_C Large K Value in the Analysis of Variance ...... 4.514 Check

Author Response: Due to weak high angle data and diffuse scattering about Bragg peaks that was not modeled.

PLAT911_ALERT_3_C Missing FCF Refl Between Thmin & STh/L= 0.600 13 Report

---

Alert level G

PLAT912_ALERT_4_G Missing # of FCF Reflections Above STh/L= 0.600 65 Note
PLAT933_ALERT_2_G Number of OMIT Records in Embedded .res File ... 5 Note
PLAT954_ALERT_1_G Reported (CIF) and Actual (FCF) Kmax Differ by . 1 Units
PLAT978_ALERT_2_G Number C-C Bonds with Positive Residual Density. 3 Info

0 **ALERT level A** = Most likely a serious problem - resolve or explain
0 **ALERT level B** = A potentially serious problem, consider carefully
3 **ALERT level C** = Check. Ensure it is not caused by an omission or oversight
4 **ALERT level G** = General information/check it is not something unexpected

1 ALERT type 1 CIF construction/syntax error, inconsistent or missing data
2 ALERT type 2 Indicator that the structure model may be wrong or deficient
3 ALERT type 3 Indicator that the structure quality may be low
1 ALERT type 4 Improvement, methodology, query or suggestion
0 ALERT type 5 Informative message, check

Datablock: diimineBr_290K

Bond precision: C-C = 0.0051 A Wavelength=1.54178

Cell:	a=7.48669(12)	b=38.8999(4)	c=5.93712(7)
	alpha=90	beta=90.4022(13)	gamma=90
Temperature:	290 K		

	Calculated	Reported
Volume	1729.03(4)	1729.03(4)
Space group	P 21/c	P 21/c
Hall group	-P 2ybc	-P 2ybc
Moiety formula	C20 H14 Br2 N2	?
Sum formula	C20 H14 Br2 N2	C20 H14 Br2 N2
Mr	442.13	442.15
Dx,g cm-3	1.699	1.699
Z	4	4
Mu (mm-1)	5.958	5.958
F000	872.0	872.0
F000’	868.19	
h,k,lmax	9,49,7	9,49,7
Nref	3685	3607
Tmin,Tmax	0.306,0.898	0.315,1.000
Tmin’	0.107	

Correction method= # Reported T Limits: Tmin=0.315 Tmax=1.000
AbsCorr = GAUSSIAN

Data completeness= 0.979 Theta(max)= 77.530

R(reflections)= 0.0468(3065) wR2(reflections)= 0.1271(3607)

S = 1.087 Npar= 217

```
The following ALERTS were generated. Each ALERT has the format
       test-name_ALERT_alert-type_alert-level.
Click on the hyperlinks for more details of the test.
```

Alert level C

```
PLAT906_ALERT_3_C Large K Value in the Analysis of Variance ......      4.650 Check
```

Author Response: Due to weak high angle data and diffuse scattering about Bragg peaks that was not modeled.

```
PLAT911_ALERT_3_C Missing FCF Refl Between Thmin & STh/L=    0.600          7 Report
```

Alert level G

```
PLAT912_ALERT_4_G Missing # of FCF Reflections Above STh/L=  0.600         72 Note
PLAT933_ALERT_2_G Number of OMIT Records in Embedded .res File ...          5 Note
PLAT954_ALERT_1_G Reported (CIF) and Actual (FCF) Kmax Differ by .          1 Units
PLAT978_ALERT_2_G Number C-C Bonds with Positive Residual Density.          4 Info
```

```
   0 ALERT level A = Most likely a serious problem - resolve or explain
   0 ALERT level B = A potentially serious problem, consider carefully
   2 ALERT level C = Check. Ensure it is not caused by an omission or oversight
   4 ALERT level G = General information/check it is not something unexpected

   1 ALERT type 1 CIF construction/syntax error, inconsistent or missing data
   2 ALERT type 2 Indicator that the structure model may be wrong or deficient
   2 ALERT type 3 Indicator that the structure quality may be low
   1 ALERT type 4 Improvement, methodology, query or suggestion
   0 ALERT type 5 Informative message, check
```

Validation response form

Please find below a validation response form (VRF) that can be filled in and pasted into your CIF.

```
# start Validation Reply Form
_vrf_PLAT911_diimineI_270K
;
PROBLEM: Missing FCF Refl Between Thmin & STh/L=    0.600          5 Report
RESPONSE: ...
;
_vrf_PLAT911_diimineBr_190K
;
PROBLEM: Missing FCF Refl Between Thmin & STh/L=    0.600          8 Report
RESPONSE: ...
;
_vrf_PLAT911_diimineBr_210K
;
PROBLEM: Missing FCF Refl Between Thmin & STh/L=    0.600          9 Report
RESPONSE: ...
;
_vrf_PLAT911_diimineBr_230K
;
PROBLEM: Missing FCF Refl Between Thmin & STh/L=    0.600         10 Report
RESPONSE: ...
;
_vrf_PLAT911_diimineBr_250K
```

```
;
PROBLEM: Missing FCF Refl Between Thmin & STh/L=    0.600          9 Report
RESPONSE: ...
;
_vrf_PLAT911_diimineBr_270K
;
PROBLEM: Missing FCF Refl Between Thmin & STh/L=    0.600         13 Report
RESPONSE: ...
;
_vrf_PLAT911_diimineBr_290K
;
PROBLEM: Missing FCF Refl Between Thmin & STh/L=    0.600          7 Report
RESPONSE: ...
;
# end Validation Reply Form
```

It is advisable to attempt to resolve as many as possible of the alerts in all categories. Often the minor alerts point to easily fixed oversights, errors and omissions in your CIF or refinement strategy, so attention to these fine details can be worthwhile. In order to resolve some of the more serious problems it may be necessary to carry out additional measurements or structure refinements. However, the purpose of your study may justify the reported deviations and the more serious of these should normally be commented upon in the discussion or experimental section of a paper or in the "special_details" fields of the CIF. checkCIF was carefully designed to identify outliers and unusual parameters, but every test has its limitations and alerts that are not important in a particular case may appear. Conversely, the absence of alerts does not guarantee there are no aspects of the results needing attention. It is up to the individual to critically assess their own results and, if necessary, seek expert advice.

Publication of your CIF in IUCr journals

A basic structural check has been run on your CIF. These basic checks will be run on all CIFs submitted for publication in IUCr journals (*Acta Crystallographica*, *Journal of Applied Crystallography*, *Journal of Synchrotron Radiation*); however, if you intend to submit to *Acta Crystallographica Section C* or *E* or *IUCrData*, you should make sure that full publication checks are run on the final version of your CIF prior to submission.

Publication of your CIF in other journals

Please refer to the *Notes for Authors* of the relevant journal for any special instructions relating to CIF submission.

PLATON version of 03/06/2021; check.def file version of 02/06/2021

Datablock diimineI_190K - ellipsoid plot

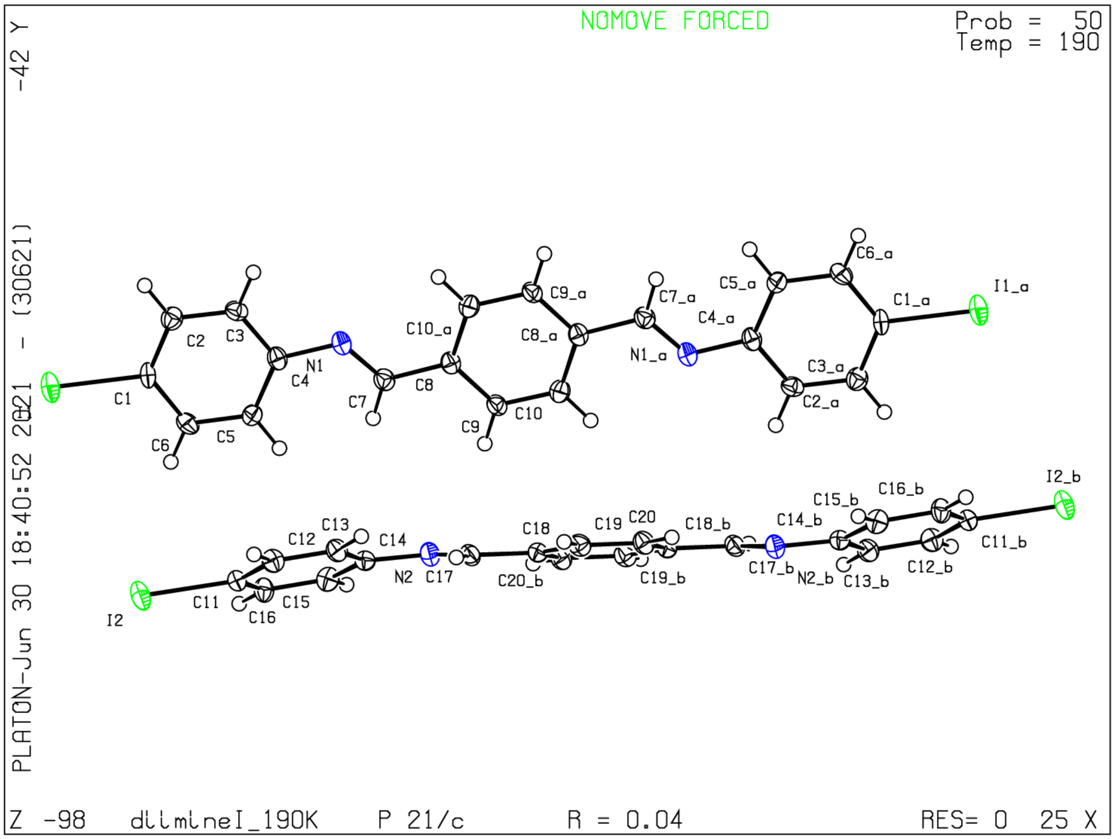

Datablock diimineI_210K - ellipsoid plot

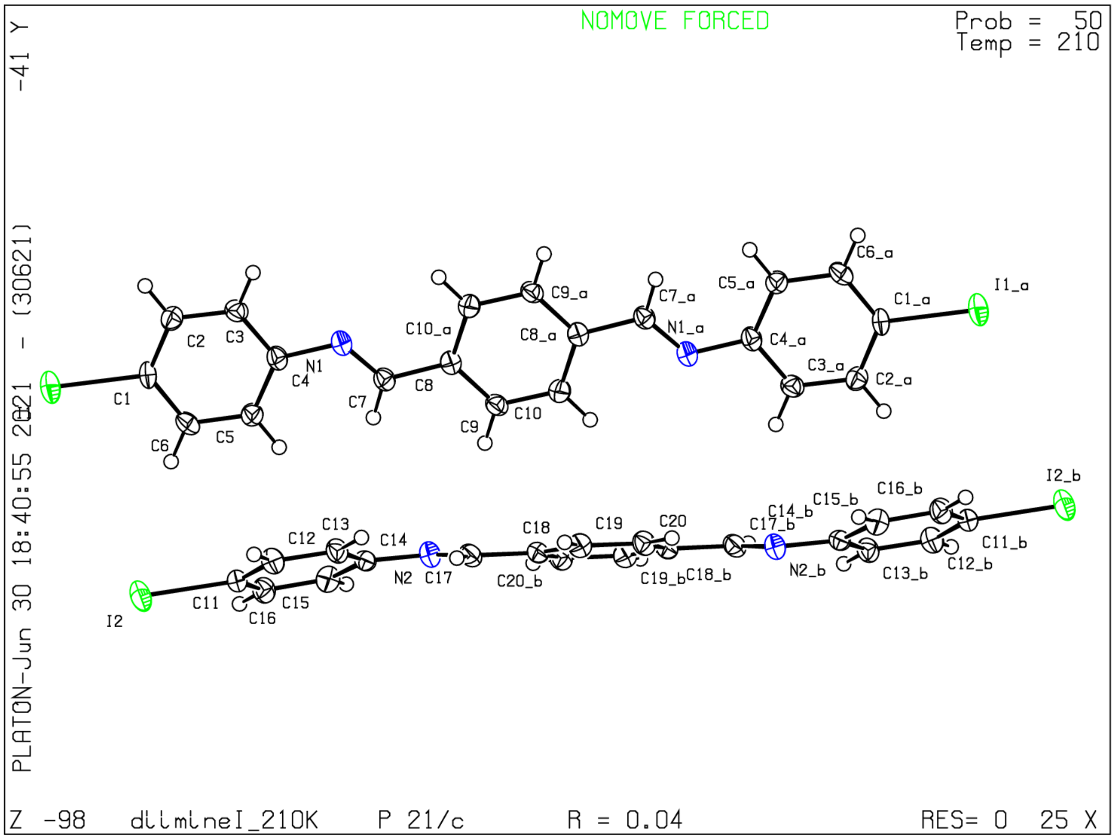

Datablock diimineI_230K - ellipsoid plot

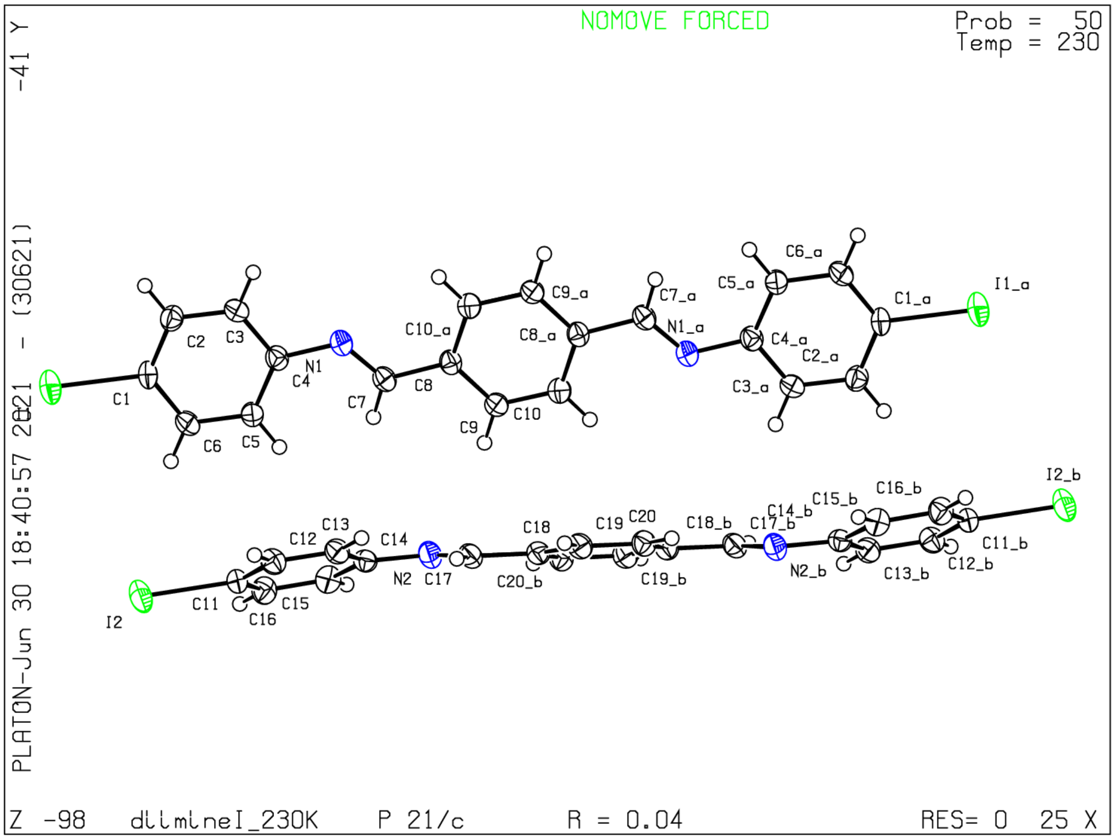

Datablock diimineI_250K - ellipsoid plot

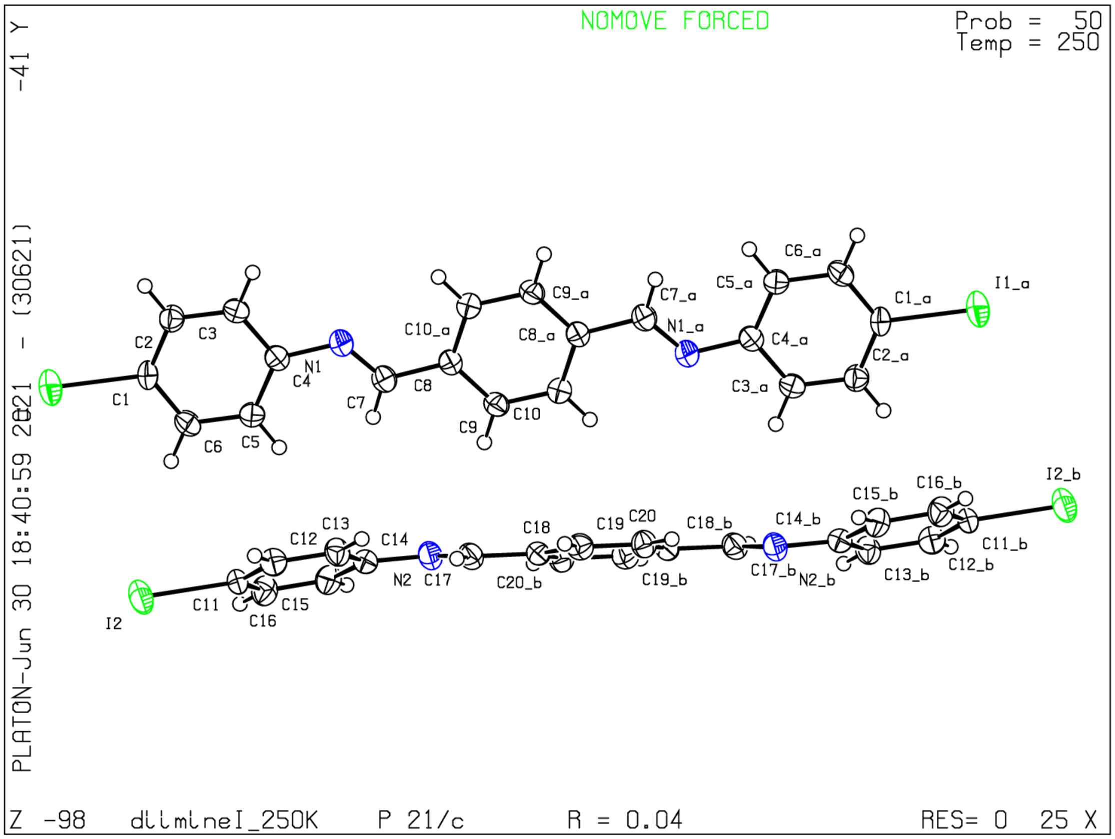

Datablock diimineI_270K - ellipsoid plot

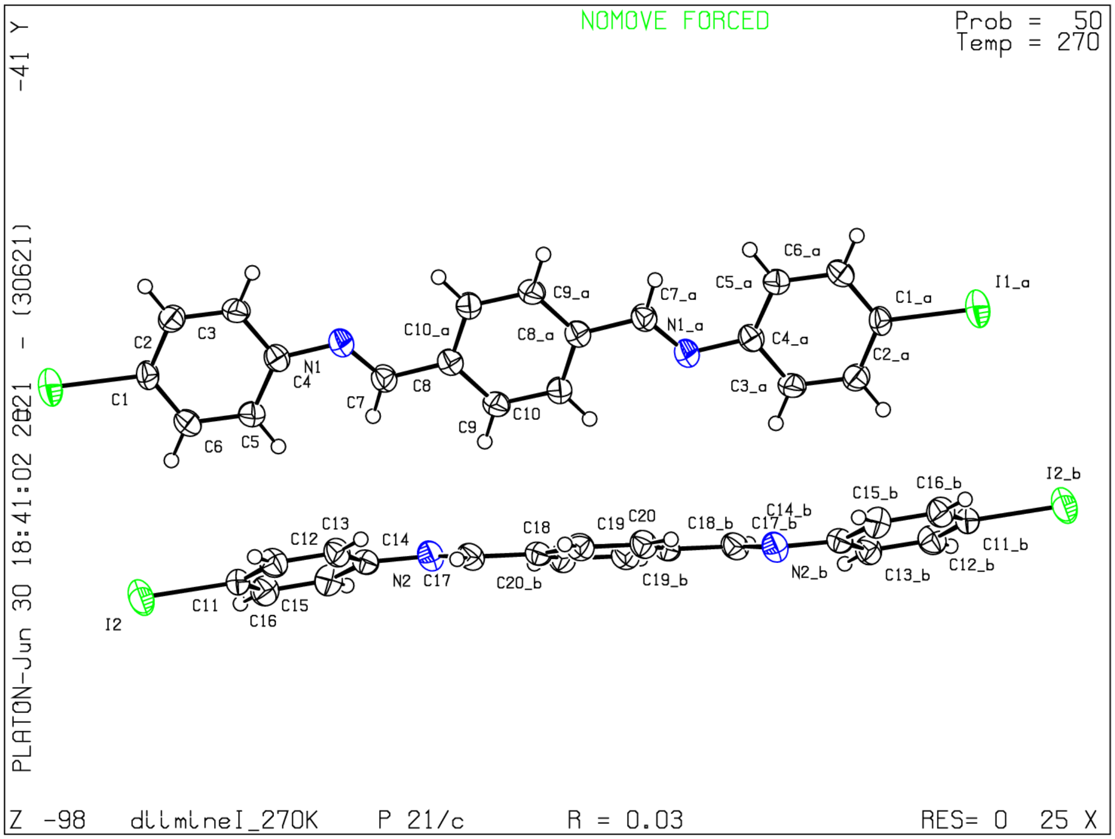

Datablock diimineI_290K - ellipsoid plot

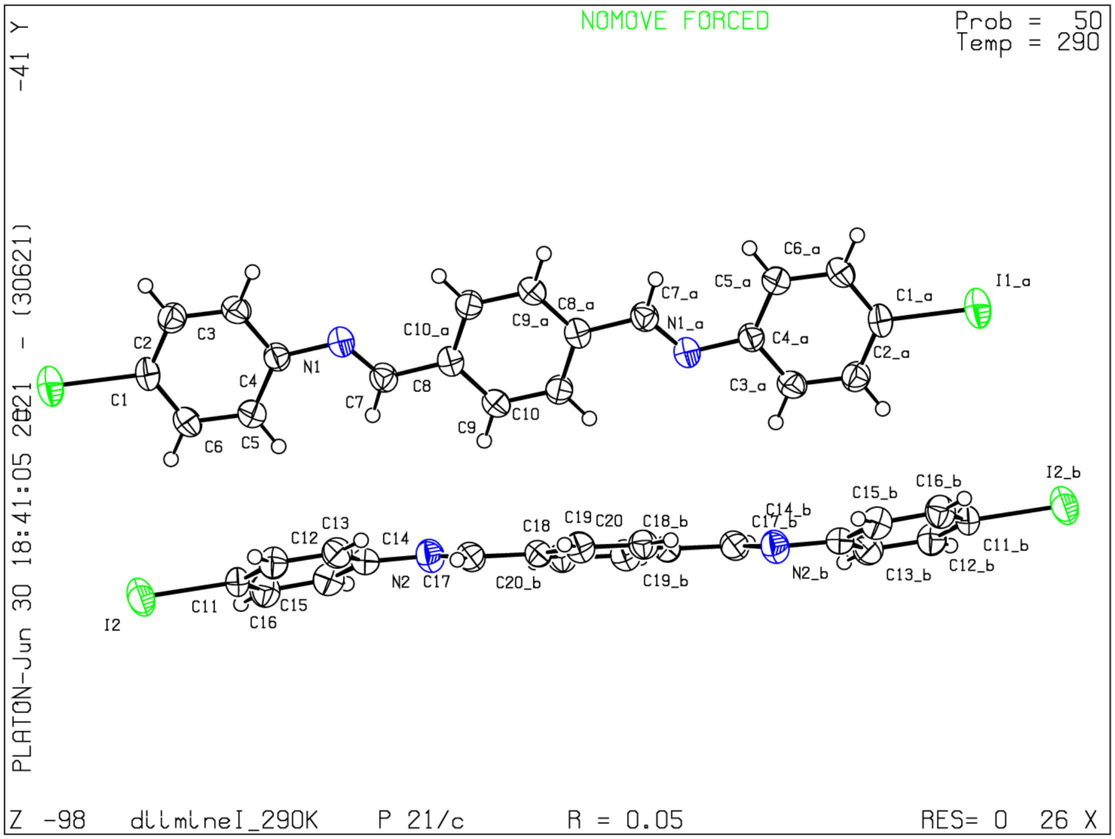

Datablock diimineBr_190K - ellipsoid plot

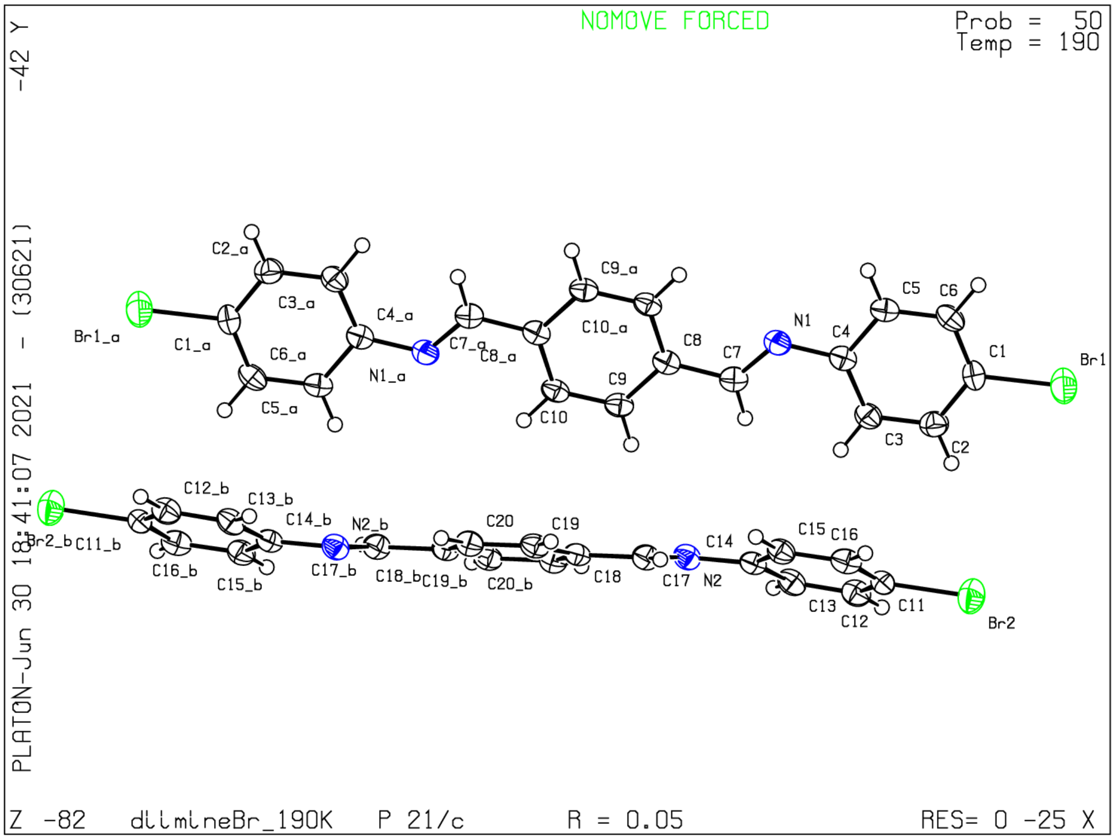

Datablock diimineBr_210K - ellipsoid plot

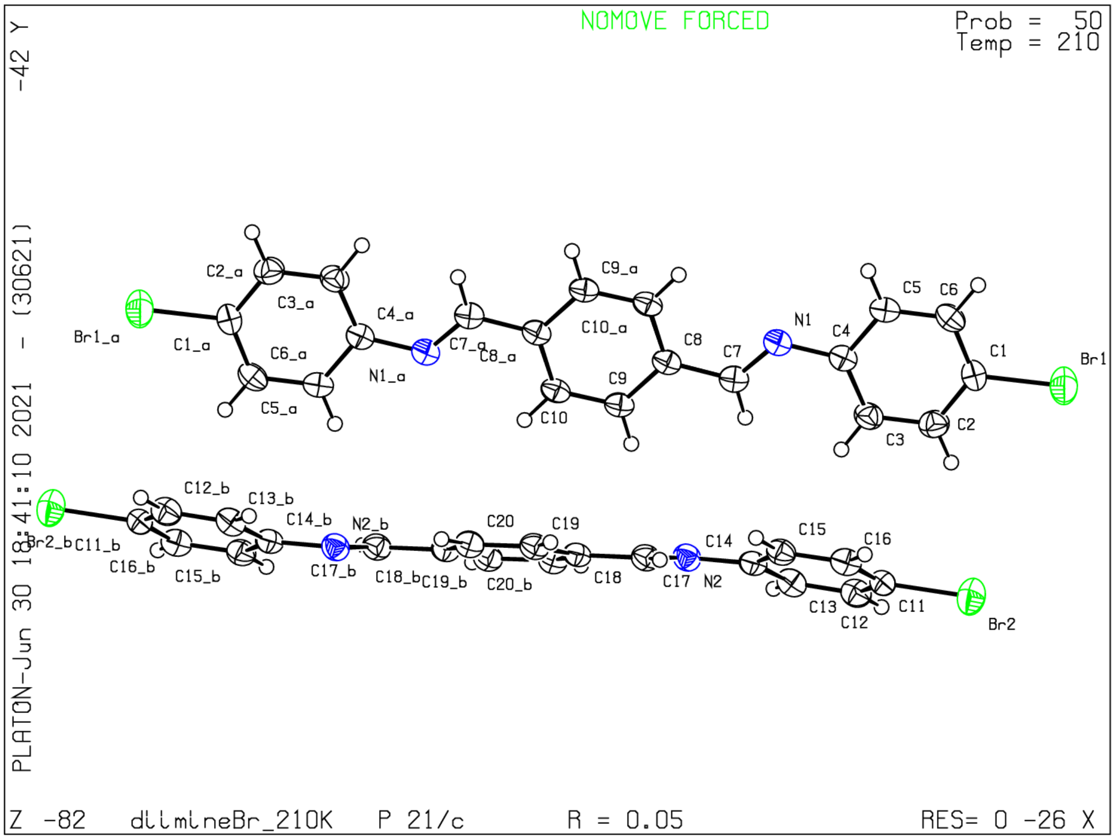

Datablock diimineBr_230K - ellipsoid plot

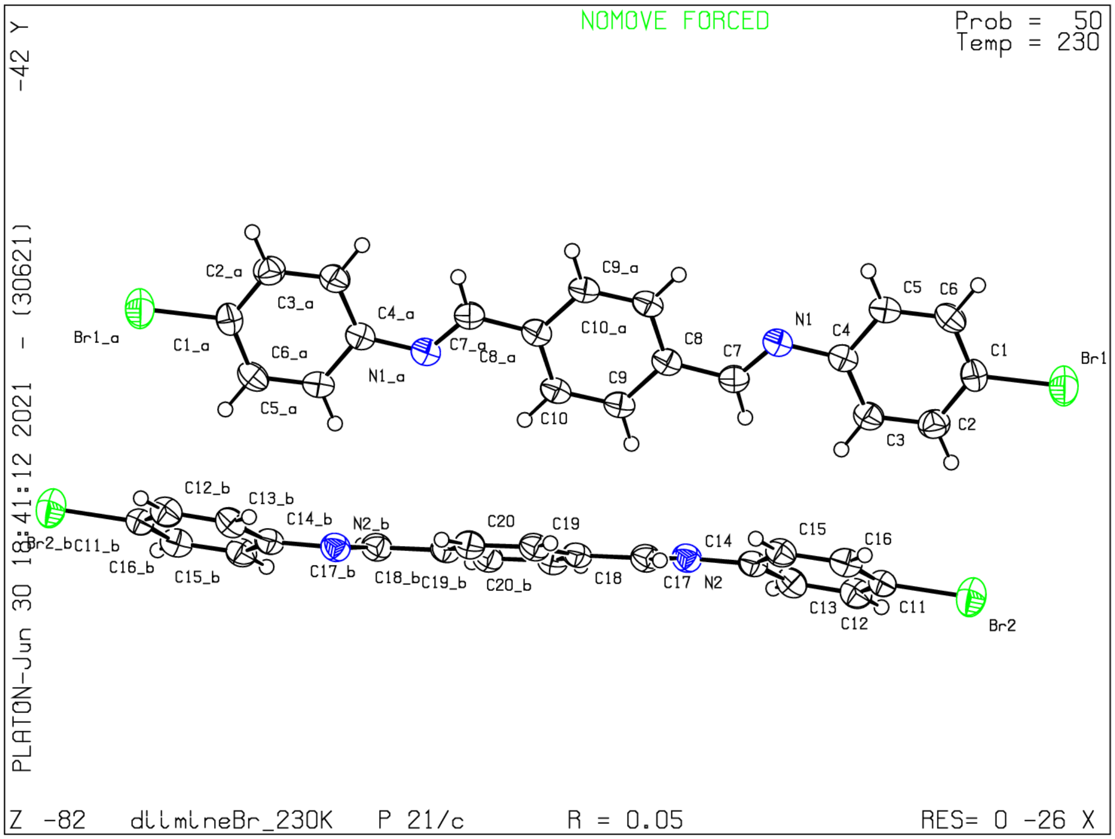

Datablock diimineBr_250K - ellipsoid plot

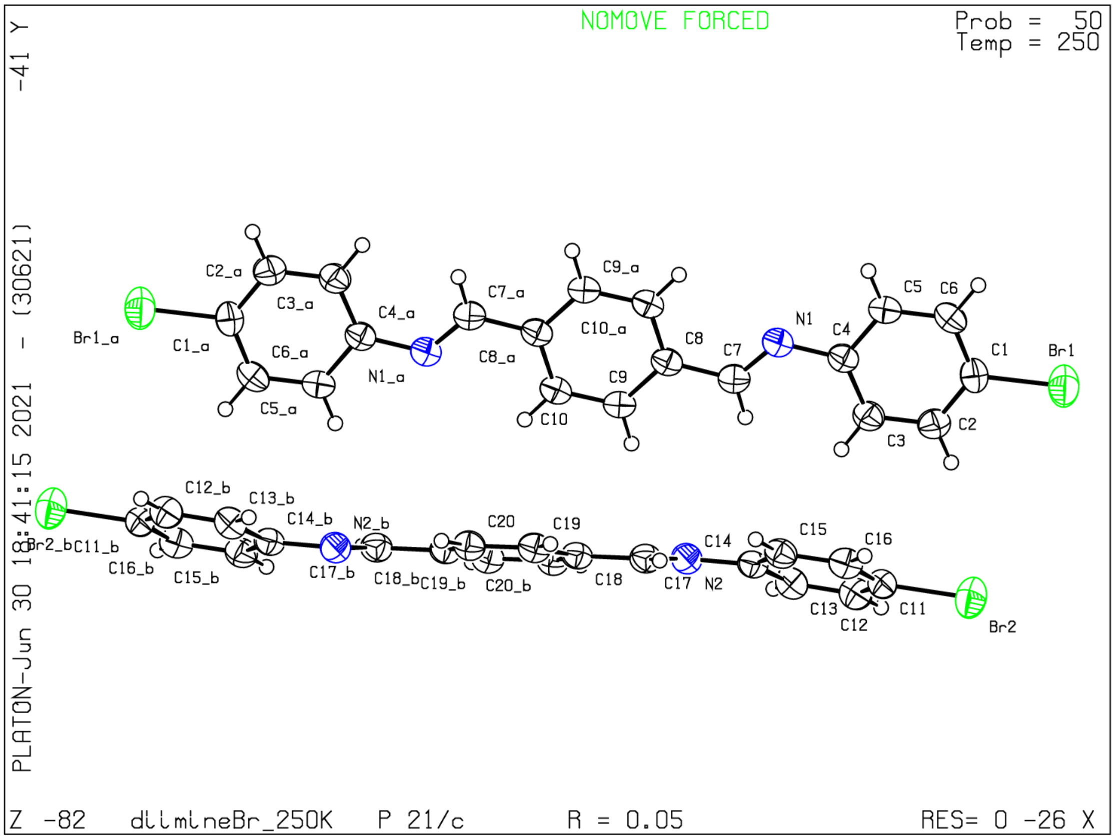

Datablock diimineBr_270K - ellipsoid plot

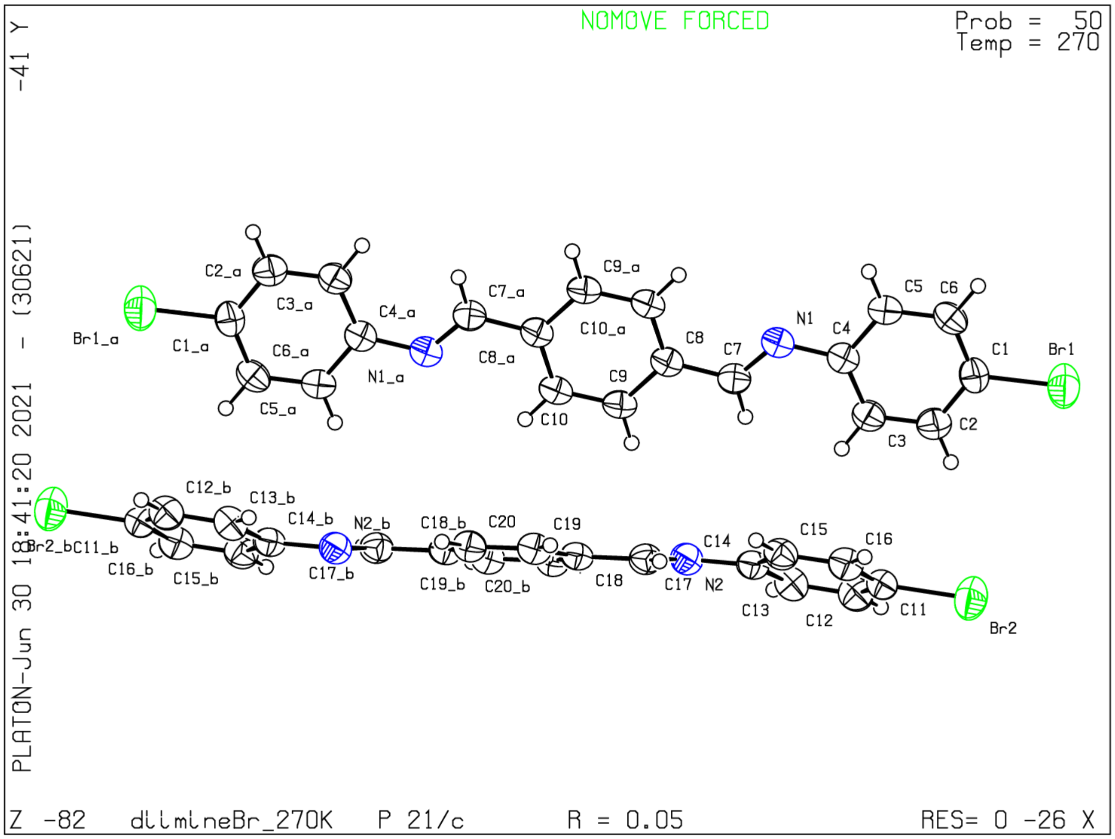

Datablock diimineBr_290K - ellipsoid plot

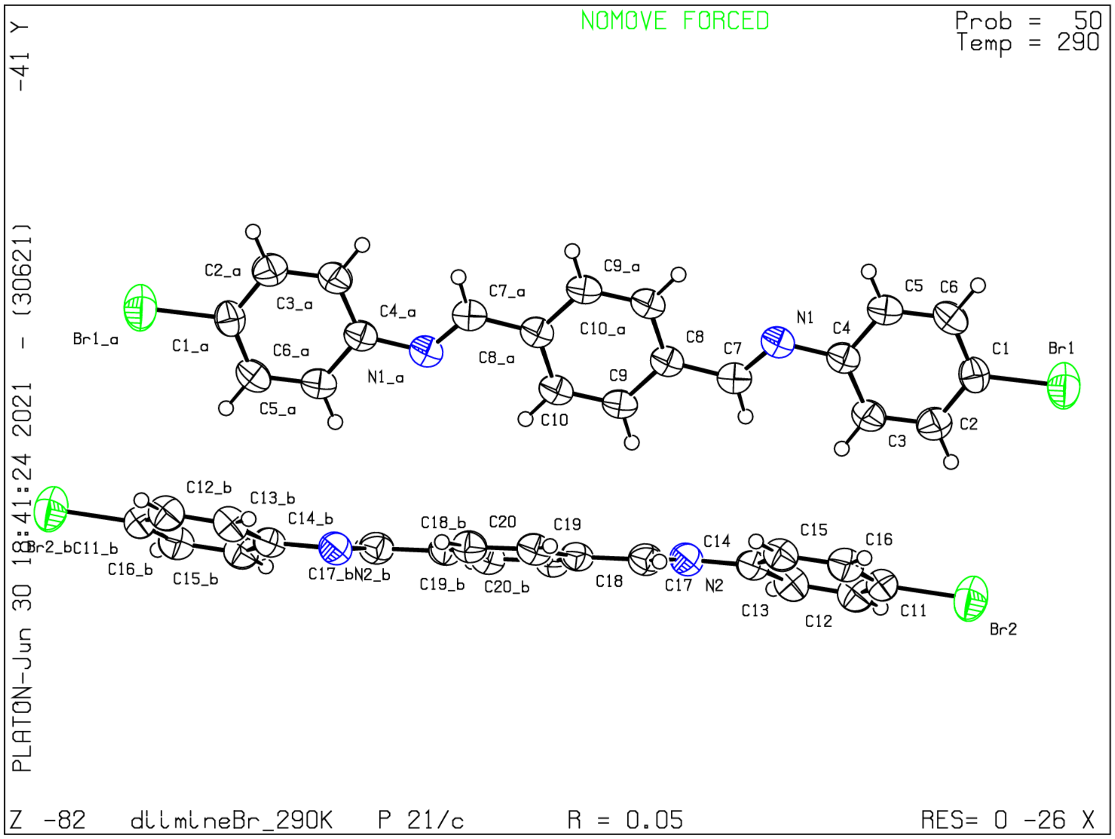

Supplement: Supplementary file 3 [file diimines.pdf › diimines.pdf]
