# Supplementary material for: Differences in thermal expansion and motion ability for herringbone and face-to-face π-stacked solids
Source: IUCrJ. 2021 Nov 3;9(Pt 1):31–42. doi: 10.1107/S2052252521009593 (PMC8733877; doi:10.1107/S2052252521009593)

checkCIF/PLATON report

Structure factors have been supplied for datablock(s) diolefinIBr_190, diolefinIBr_210, diolefinIBr_230, diolefinIBr_250, diolefinIBr_270, diolefinIBr_290

THIS REPORT IS FOR GUIDANCE ONLY. IF USED AS PART OF A REVIEW PROCEDURE FOR PUBLICATION, IT SHOULD NOT REPLACE THE EXPERTISE OF AN EXPERIENCED CRYSTALLOGRAPHIC REFEREE.

No syntax errors found. CIF dictionary Interpreting this report

Datablock: diolefinIBr_190

```
Bond precision:  C-C = 0.0040 A         Wavelength=1.54184

Cell:            a=7.38099(11)     b=40.1967(6)     c=5.92440(6)
                 alpha=90          beta=90          gamma=90
Temperature:     190 K

                Calculated                 Reported
Volume          1757.72(4)                 1757.72(4)
Space group     P c c n                    P c c n
Hall group      -P 2ab 2ac                 -P 2ab 2ac
Moiety formula  C22 H16 Br I               C22 H16 Br I
Sum formula     C22 H16 Br I               C22 H16 Br I
Mr              487.15                     487.16
Dx,g cm-3       1.841                      1.841
Z               4                          4
Mu (mm-1)       16.951                     16.951
F000            944.0                      944.0
F000’           942.07
h,k,lmax        9,50,7                     9,50,7
Nref            1869                       1860
Tmin,Tmax       0.160,0.888                0.184,1.000
Tmin’           0.037

Correction method= # Reported T Limits: Tmin=0.184 Tmax=1.000
AbsCorr = GAUSSIAN

Data completeness= 0.995          Theta(max)= 77.552

R(reflections)= 0.0264( 1728)     wR2(reflections)= 0.0681( 1860)

S = 1.077                  Npar= 119
```

The following ALERTS were generated. Each ALERT has the format
test-name_ALERT_alert-type_alert-level.
Click on the hyperlinks for more details of the test.

Alert level C

PLAT906_ALERT_3_C Large K Value in the Analysis of Variance ...... 2.193 Check

Author Response: Due to slight diffuse scattering about the major Bragg peaks that was not accounted for during the integration and absorption correction.

Alert level G

```
PLAT230_ALERT_2_G Hirshfeld Test Diff for    I1       --C1       .        7.7 s.u.
PLAT230_ALERT_2_G Hirshfeld Test Diff for    Br1      --C1       .        8.2 s.u.
PLAT300_ALERT_4_G Atom Site Occupancy of I1         Constrained at        0.5 Check
PLAT300_ALERT_4_G Atom Site Occupancy of Br1        Constrained at        0.5 Check
PLAT301_ALERT_3_G Main Residue  Disorder .............(Resd  1  )          8% Note
PLAT912_ALERT_4_G Missing # of FCF Reflections Above STh/L=  0.600         10 Note
PLAT933_ALERT_2_G Number of OMIT Records in Embedded .res File ...          3 Note
PLAT978_ALERT_2_G Number C-C Bonds with Positive Residual Density.          5 Info
```

---

0 **ALERT level A** = Most likely a serious problem - resolve or explain
0 **ALERT level B** = A potentially serious problem, consider carefully
1 **ALERT level C** = Check. Ensure it is not caused by an omission or oversight
8 **ALERT level G** = General information/check it is not something unexpected

0 ALERT type 1 CIF construction/syntax error, inconsistent or missing data
4 ALERT type 2 Indicator that the structure model may be wrong or deficient
2 ALERT type 3 Indicator that the structure quality may be low
3 ALERT type 4 Improvement, methodology, query or suggestion
0 ALERT type 5 Informative message, check

Datablock: diolefinIBr_210

Bond precision: C-C = 0.0030 A Wavelength=1.54184

Cell:	a=7.39817(7)	b=40.1869(3)	c=5.93303(4)
	alpha=90	beta=90	gamma=90
Temperature:	210 K		

	Calculated	Reported
Volume	1763.95(2)	1763.95(3)
Space group	P c c n	P c c n
Hall group	-P 2ab 2ac	-P 2ab 2ac
Moiety formula	C22 H16 Br I	C22 H16 Br I
Sum formula	C22 H16 Br I	C22 H16 Br I
Mr	487.15	487.16
Dx,g cm-3	1.834	1.834
Z	4	4
Mu (mm-1)	16.891	16.891
F000	944.0	944.0
F000’	942.07	
h,k,lmax	9,50,7	9,50,7
Nref	1878	1870
Tmin,Tmax	0.161,0.888	0.179,1.000
Tmin’	0.038	

Correction method= # Reported T Limits: Tmin=0.179 Tmax=1.000
AbsCorr = GAUSSIAN

Data completeness= 0.996   Theta(max)= 77.657

R(reflections)= 0.0212(1781)   wR2(reflections)= 0.0550(1870)

S = 1.127   Npar= 119

The following ALERTS were generated. Each ALERT has the format
test-name_ALERT_alert-type_alert-level.
Click on the hyperlinks for more details of the test.

Alert level G

```
PLAT142_ALERT_4_G s.u. on b - Axis Small or Missing .............    0.00030 Ang.
PLAT143_ALERT_4_G s.u. on c - Axis Small or Missing .............    0.00004 Ang.
PLAT300_ALERT_4_G Atom Site Occupancy of I1        Constrained at        0.5 Check
PLAT300_ALERT_4_G Atom Site Occupancy of Br1       Constrained at        0.5 Check
PLAT301_ALERT_3_G Main Residue  Disorder .............(Resd  1  )         8% Note
PLAT912_ALERT_4_G Missing # of FCF Reflections Above STh/L=  0.600         9 Note
PLAT933_ALERT_2_G Number of OMIT Records in Embedded .res File ...         3 Note
PLAT978_ALERT_2_G Number C-C Bonds with Positive Residual Density.        11 Info
```

---

0 **ALERT level A** = Most likely a serious problem - resolve or explain
0 **ALERT level B** = A potentially serious problem, consider carefully
0 **ALERT level C** = Check. Ensure it is not caused by an omission or oversight
8 **ALERT level G** = General information/check it is not something unexpected

0 ALERT type 1 CIF construction/syntax error, inconsistent or missing data
2 ALERT type 2 Indicator that the structure model may be wrong or deficient
1 ALERT type 3 Indicator that the structure quality may be low
5 ALERT type 4 Improvement, methodology, query or suggestion
0 ALERT type 5 Informative message, check

Datablock: diolefinIBr_230

```
Bond precision:   C-C = 0.0030 A          Wavelength=1.54184

Cell:             a=7.41588(6)     b=40.1789(3)     c=5.94234(4)
                  alpha=90         beta=90          gamma=90
Temperature:      230 K
```

	Calculated	Reported
Volume	1770.59(2)	1770.59(2)
Space group	P c c n	P c c n
Hall group	-P 2ab 2ac	-P 2ab 2ac
Moiety formula	C22 H16 Br I	C22 H16 Br I
Sum formula	C22 H16 Br I	C22 H16 Br I
Mr	487.15	487.16
Dx,g cm-3	1.827	1.828
Z	4	4
Mu (mm-1)	16.828	16.828
F000	944.0	944.0
F000’	942.07	
h,k,lmax	9,50,7	9,50,7
Nref	1886	1880
Tmin,Tmax	0.163,0.889	0.187,1.000
Tmin’	0.038	

```
Correction method= # Reported T Limits: Tmin=0.187 Tmax=1.000
AbsCorr = GAUSSIAN

Data completeness= 0.997          Theta(max)= 77.575

R(reflections)= 0.0216( 1774)     wR2(reflections)= 0.0552( 1880)

S = 1.060                  Npar= 119
```

The following ALERTS were generated. Each ALERT has the format
test-name_ALERT_alert-type_alert-level.
Click on the hyperlinks for more details of the test.

Alert level G

```
PLAT142_ALERT_4_G s.u. on b - Axis Small or Missing .............    0.00030 Ang.
PLAT143_ALERT_4_G s.u. on c - Axis Small or Missing .............    0.00004 Ang.
PLAT300_ALERT_4_G Atom Site Occupancy of I1        Constrained at        0.5 Check
PLAT300_ALERT_4_G Atom Site Occupancy of Br1       Constrained at        0.5 Check
PLAT301_ALERT_3_G Main Residue  Disorder .............(Resd  1  )         8% Note
PLAT912_ALERT_4_G Missing # of FCF Reflections Above STh/L=  0.600         6 Note
PLAT933_ALERT_2_G Number of OMIT Records in Embedded .res File ...         3 Note
PLAT978_ALERT_2_G Number C-C Bonds with Positive Residual Density.         9 Info
```

---

0 **ALERT level A** = Most likely a serious problem - resolve or explain
0 **ALERT level B** = A potentially serious problem, consider carefully
0 **ALERT level C** = Check. Ensure it is not caused by an omission or oversight
8 **ALERT level G** = General information/check it is not something unexpected

0 ALERT type 1 CIF construction/syntax error, inconsistent or missing data
2 ALERT type 2 Indicator that the structure model may be wrong or deficient
1 ALERT type 3 Indicator that the structure quality may be low
5 ALERT type 4 Improvement, methodology, query or suggestion
0 ALERT type 5 Informative message, check

Datablock: diolefinIBr_250

Bond precision: C-C = 0.0030 A Wavelength=1.54184

Cell:	a=7.43466(7)	b=40.1695(3)	c=5.95124(4)
	alpha=90	beta=90	gamma=90
Temperature:	250 K		

	Calculated	Reported
Volume	1777.32(2)	1777.32(3)
Space group	P c c n	P c c n
Hall group	-P 2ab 2ac	-P 2ab 2ac
Moiety formula	C22 H16 Br I	C22 H16 Br I
Sum formula	C22 H16 Br I	C22 H16 Br I
Mr	487.15	487.16
Dx,g cm-3	1.821	1.821
Z	4	4
Mu (mm-1)	16.764	16.764
F000	944.0	944.0
F000’	942.07	
h,k,lmax	9,50,7	9,50,7
Nref	1891	1886
Tmin,Tmax	0.164,0.889	0.189,1.000
Tmin’	0.038	

Correction method= # Reported T Limits: Tmin=0.189 Tmax=1.000
AbsCorr = GAUSSIAN

Data completeness= 0.997 Theta(max)= 77.588

R(reflections)= 0.0211(1760) wR2(reflections)= 0.0529(1886)

S = 1.089 Npar= 140

The following ALERTS were generated. Each ALERT has the format
test-name_ALERT_alert-type_alert-level.
Click on the hyperlinks for more details of the test.

Alert level C

```
PLAT906_ALERT_3_C Large K Value in the Analysis of Variance ......      2.029 Check
```

Author Response: Due to slight diffuse scattering about the major Bragg peaks that was not accounted for during the integration and absorption correction.

Alert level G

```
PLAT002_ALERT_2_G Number of Distance or Angle Restraints on AtSite          6 Note
PLAT003_ALERT_2_G Number of Uiso or Uij Restrained non-H Atoms ...          4 Report
PLAT142_ALERT_4_G s.u. on b - Axis Small or Missing .............     0.00030 Ang.
PLAT143_ALERT_4_G s.u. on c - Axis Small or Missing .............     0.00004 Ang.
PLAT172_ALERT_4_G The CIF-Embedded .res File Contains DFIX Records          2 Report
PLAT178_ALERT_4_G The CIF-Embedded .res File Contains SIMU Records          1 Report
PLAT187_ALERT_4_G The CIF-Embedded .res File Contains RIGU Records          1 Report
PLAT230_ALERT_2_G Hirshfeld Test Diff for    I1       --C1       .        5.7 s.u.
PLAT300_ALERT_4_G Atom Site Occupancy of I1         Constrained at        0.5 Check
PLAT300_ALERT_4_G Atom Site Occupancy of Br1        Constrained at        0.5 Check
PLAT301_ALERT_3_G Main Residue  Disorder ..............(Resd  1  )        25% Note
PLAT410_ALERT_2_G Short Intra H...H Contact  H3       ..H7B      .       1.56 Ang.
                                                      x,y,z  =      1_555 Check
PLAT410_ALERT_2_G Short Intra H...H Contact  H10      ..H8B      .       1.61 Ang.
                                                      x,y,z  =      1_555 Check
PLAT860_ALERT_3_G Number of Least-Squares Restraints ............          48 Note
PLAT912_ALERT_4_G Missing # of FCF Reflections Above STh/L=  0.600          5 Note
PLAT933_ALERT_2_G Number of OMIT Records in Embedded .res File ...          3 Note
PLAT978_ALERT_2_G Number C-C Bonds with Positive Residual Density.         11 Info
```

---

0 **ALERT level A** = Most likely a serious problem - resolve or explain
0 **ALERT level B** = A potentially serious problem, consider carefully
1 **ALERT level C** = Check. Ensure it is not caused by an omission or oversight
17 **ALERT level G** = General information/check it is not something unexpected

0 ALERT type 1 CIF construction/syntax error, inconsistent or missing data
7 ALERT type 2 Indicator that the structure model may be wrong or deficient
3 ALERT type 3 Indicator that the structure quality may be low
8 ALERT type 4 Improvement, methodology, query or suggestion
0 ALERT type 5 Informative message, check

Datablock: diolefinIBr_270

Bond precision:	C-C = 0.0030 A		Wavelength=1.54184
Cell:	a=7.45535(7)	b=40.1523(3)	c=5.96072(4)
	alpha=90	beta=90	gamma=90
Temperature:	270 K		

	Calculated	Reported
Volume	1784.34(2)	1784.34(3)
Space group	P c c n	P c c n
Hall group	-P 2ab 2ac	-P 2ab 2ac
Moiety formula	C22 H16 Br I	C22 H16 Br I
Sum formula	C22 H16 Br I	C22 H16 Br I
Mr	487.15	487.16
Dx,g cm-3	1.813	1.813
Z	4	4
Mu (mm-1)	16.698	16.698
F000	944.0	944.0
F000’	942.07	
h,k,lmax	9,50,7	9,50,7
Nref	1901	1894
Tmin,Tmax	0.165,0.890	0.186,1.000
Tmin’	0.039	

Correction method= # Reported T Limits: Tmin=0.186 Tmax=1.000
AbsCorr = GAUSSIAN

Data completeness= 0.996 Theta(max)= 77.538

R(reflections)= 0.0222(1754) wR2(reflections)= 0.0545(1894)

S = 1.115 Npar= 140

The following ALERTS were generated. Each ALERT has the format
test-name_ALERT_alert-type_alert-level.
Click on the hyperlinks for more details of the test.

Alert level C

PLAT906_ALERT_3_C Large K Value in the Analysis of Variance ...... 2.258 Check

Author Response: Due to slight diffuse scattering about the major Bragg peaks that was not accounted for during the integration and absorption correction.

Alert level G

PLAT002_ALERT_2_G Number of Distance or Angle Restraints on AtSite 6 Note
PLAT003_ALERT_2_G Number of Uiso or Uij Restrained non-H Atoms ... 4 Report
PLAT142_ALERT_4_G s.u. on b - Axis Small or Missing .... 0.00030 Ang.
PLAT143_ALERT_4_G s.u. on c - Axis Small or Missing .... 0.00004 Ang.
PLAT172_ALERT_4_G The CIF-Embedded .res File Contains DFIX Records 2 Report
PLAT178_ALERT_4_G The CIF-Embedded .res File Contains SIMU Records 1 Report
PLAT187_ALERT_4_G The CIF-Embedded .res File Contains RIGU Records 1 Report
PLAT230_ALERT_2_G Hirshfeld Test Diff for I1 --C1 . 5.3 s.u.
PLAT300_ALERT_4_G Atom Site Occupancy of I1 Constrained at 0.5 Check
PLAT300_ALERT_4_G Atom Site Occupancy of Br1 Constrained at 0.5 Check

```
PLAT301_ALERT_3_G Main Residue  Disorder ..............(Resd  1  )        25% Note
PLAT410_ALERT_2_G Short Intra H...H Contact  H3       ..H7B      .       1.57 Ang.
                                                     x,y,z  =     1_555 Check
PLAT410_ALERT_2_G Short Intra H...H Contact  H10      ..H8B      .       1.66 Ang.
                                                     x,y,z  =     1_555 Check
PLAT860_ALERT_3_G Number of Least-Squares Restraints .............         48 Note
PLAT912_ALERT_4_G Missing # of FCF Reflections Above STh/L=  0.600          7 Note
PLAT933_ALERT_2_G Number of OMIT Records in Embedded .res File ...          3 Note
PLAT978_ALERT_2_G Number C-C Bonds with Positive Residual Density.         11 Info
```

   0 **ALERT level A** = Most likely a serious problem - resolve or explain
   0 **ALERT level B** = A potentially serious problem, consider carefully
   1 **ALERT level C** = Check. Ensure it is not caused by an omission or oversight
  17 **ALERT level G** = General information/check it is not something unexpected

```
   0 ALERT type 1 CIF construction/syntax error, inconsistent or missing data
   7 ALERT type 2 Indicator that the structure model may be wrong or deficient
   3 ALERT type 3 Indicator that the structure quality may be low
   8 ALERT type 4 Improvement, methodology, query or suggestion
   0 ALERT type 5 Informative message, check
```

Datablock: diolefinIBr_290

Bond precision: C-C = 0.0032 A Wavelength=1.54184

Cell:	a=7.4779(1)	b=40.1339(4)	c=5.9710(1)
	alpha=90	beta=90	gamma=90
Temperature:	290 K		

	Calculated	Reported
Volume	1792.00(4)	1792.00(4)
Space group	P c c n	P c c n
Hall group	-P 2ab 2ac	-P 2ab 2ac
Moiety formula	C22 H16 Br I	C22 H16 Br I
Sum formula	C22 H16 Br I	C22 H16 Br I
Mr	487.15	487.16
Dx,g cm-3	1.806	1.806
Z	4	4
Mu (mm-1)	16.627	16.627
F000	944.0	944.0
F000’	942.07	
h,k,lmax	9,50,7	9,50,7
Nref	1912	1903
Tmin,Tmax	0.167,0.890	0.186,1.000
Tmin’	0.039	

Correction method= # Reported T Limits: Tmin=0.186 Tmax=1.000
AbsCorr = GAUSSIAN

```
Data completeness= 0.995          Theta(max)= 77.469

R(reflections)= 0.0224( 1724)     wR2(reflections)= 0.0561( 1903)

S = 1.065                   Npar= 140
```

The following ALERTS were generated. Each ALERT has the format
test-name_ALERT_alert-type_alert-level.
Click on the hyperlinks for more details of the test.

Alert level C

```
PLAT906_ALERT_3_C Large K Value in the Analysis of Variance ......      2.296 Check
```

Author Response: Due to slight diffuse scattering about the major Bragg peaks that was not accounted for during the integration and absorption correction.

Alert level G

```
PLAT002_ALERT_2_G Number of Distance or Angle Restraints on AtSite          6 Note
PLAT003_ALERT_2_G Number of Uiso or Uij Restrained non-H Atoms ...          4 Report
PLAT142_ALERT_4_G s.u. on b - Axis Small or Missing .............     0.00040 Ang.
PLAT172_ALERT_4_G The CIF-Embedded .res File Contains DFIX Records          2 Report
PLAT178_ALERT_4_G The CIF-Embedded .res File Contains SIMU Records          1 Report
PLAT187_ALERT_4_G The CIF-Embedded .res File Contains RIGU Records          1 Report
PLAT230_ALERT_2_G Hirshfeld Test Diff for    I1       --C1       .        7.3 s.u.
PLAT300_ALERT_4_G Atom Site Occupancy of I1         Constrained at        0.5 Check
PLAT300_ALERT_4_G Atom Site Occupancy of Br1        Constrained at        0.5 Check
PLAT301_ALERT_3_G Main Residue  Disorder ..............(Resd  1  )        25% Note
PLAT410_ALERT_2_G Short Intra H...H Contact  H3       ..H7B      .       1.58 Ang.
                                                        x,y,z  =     1_555 Check
PLAT410_ALERT_2_G Short Intra H...H Contact  H10      ..H8B      .       1.68 Ang.
                                                        x,y,z  =     1_555 Check
PLAT860_ALERT_3_G Number of Least-Squares Restraints ............          48 Note
PLAT912_ALERT_4_G Missing # of FCF Reflections Above STh/L=  0.600          9 Note
PLAT933_ALERT_2_G Number of OMIT Records in Embedded .res File ...          3 Note
PLAT978_ALERT_2_G Number C-C Bonds with Positive Residual Density.          8 Info
```

---

0 **ALERT level A** = Most likely a serious problem - resolve or explain
0 **ALERT level B** = A potentially serious problem, consider carefully
1 **ALERT level C** = Check. Ensure it is not caused by an omission or oversight
16 **ALERT level G** = General information/check it is not something unexpected

0 ALERT type 1 CIF construction/syntax error, inconsistent or missing data
7 ALERT type 2 Indicator that the structure model may be wrong or deficient
3 ALERT type 3 Indicator that the structure quality may be low
7 ALERT type 4 Improvement, methodology, query or suggestion
0 ALERT type 5 Informative message, check

It is advisable to attempt to resolve as many as possible of the alerts in all categories. Often the minor alerts point to easily fixed oversights, errors and omissions in your CIF or refinement strategy, so attention to these fine details can be worthwhile. In order to resolve some of the more serious problems it may be necessary to carry out additional measurements or structure refinements. However, the purpose of your study may justify the reported deviations and the more serious of these should normally be commented upon in the discussion or experimental section of a paper or in the "special_details" fields of the CIF. checkCIF was carefully designed to identify outliers and unusual parameters, but every test has its limitations and alerts that are not important in a particular case may appear. Conversely, the absence of alerts does not guarantee there are no aspects of the results needing attention. It is up to the individual to critically assess their own results and, if necessary, seek expert advice.

Publication of your CIF in IUCr journals

A basic structural check has been run on your CIF. These basic checks will be run on all CIFs submitted for publication in IUCr journals (*Acta Crystallographica*, *Journal of Applied Crystallography*, *Journal of Synchrotron Radiation*); however, if you intend to submit to *Acta Crystallographica Section C* or *E* or *IUCrData*, you should make sure that full publication checks are run on the final version of your CIF prior to submission.

Publication of your CIF in other journals

Please refer to the *Notes for Authors* of the relevant journal for any special instructions relating to CIF submission.

PLATON version of 03/06/2021; check.def file version of 02/06/2021

Datablock diolefinIBr_190 - ellipsoid plot

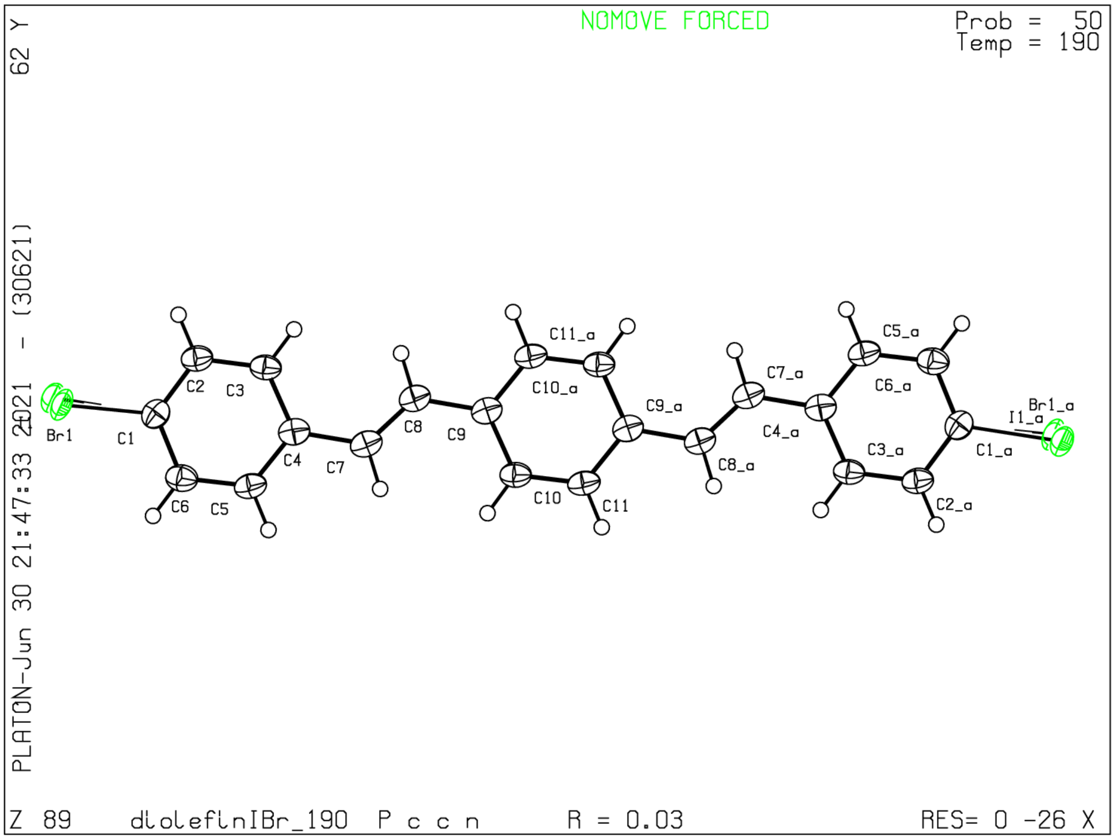

Datablock diolefinIBr_210 - ellipsoid plot

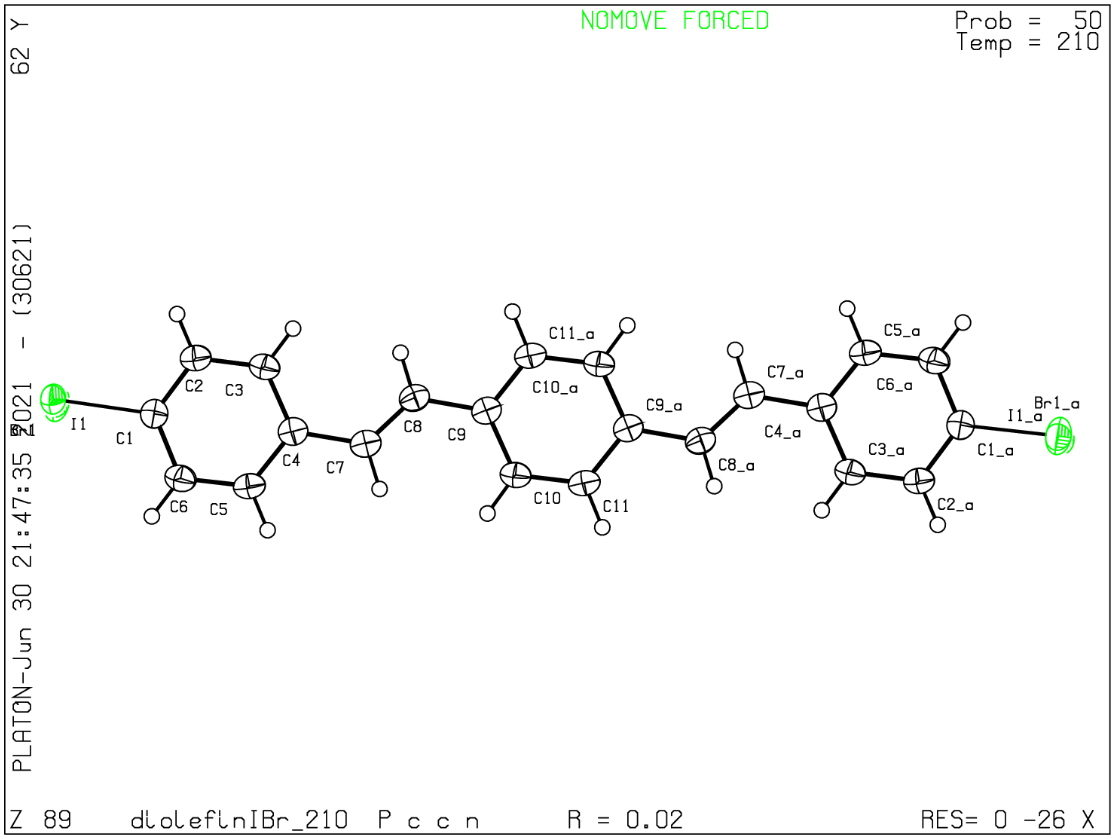

Datablock diolefinIBr_230 - ellipsoid plot

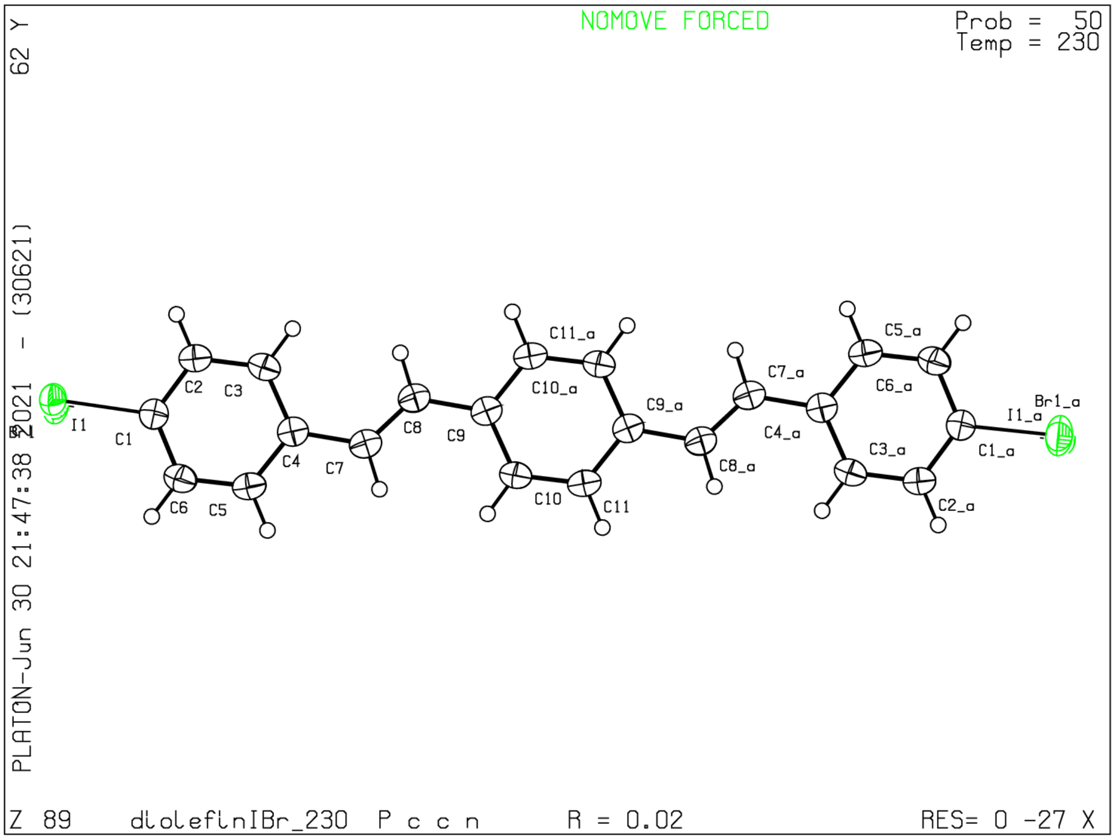

Datablock diolefinIBr_250 - ellipsoid plot

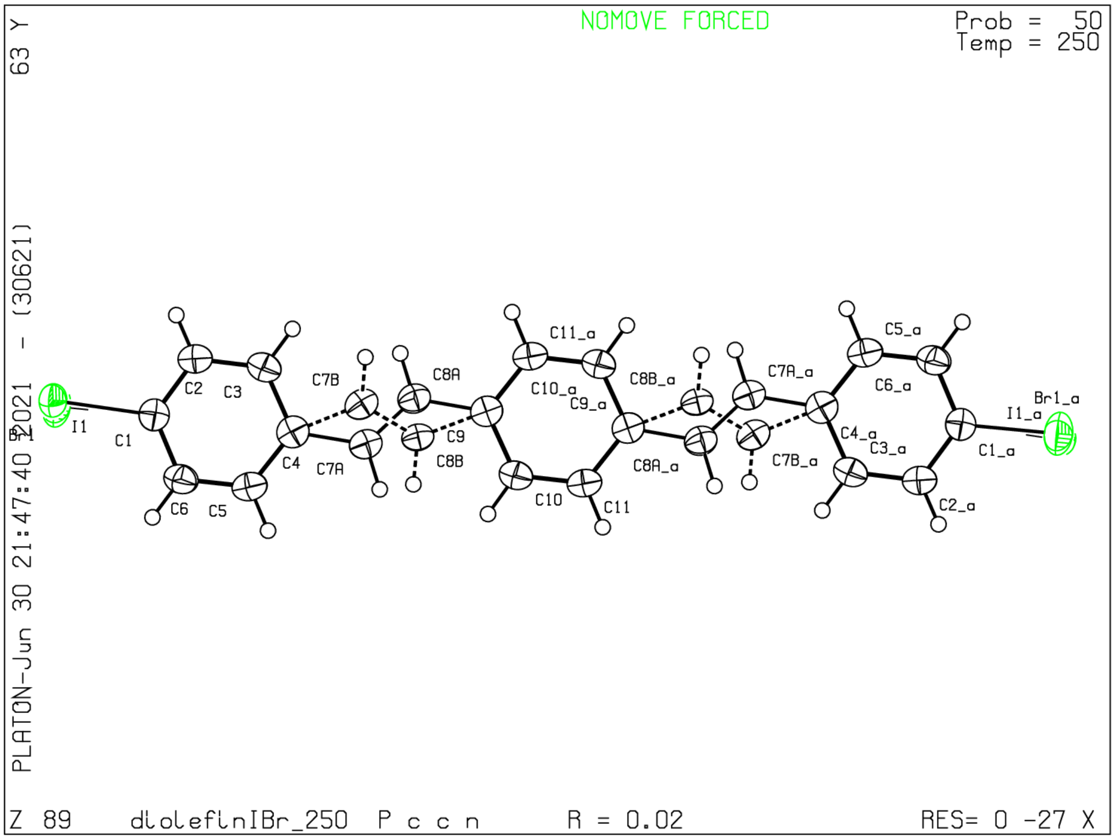

Datablock diolefinIBr_270 - ellipsoid plot

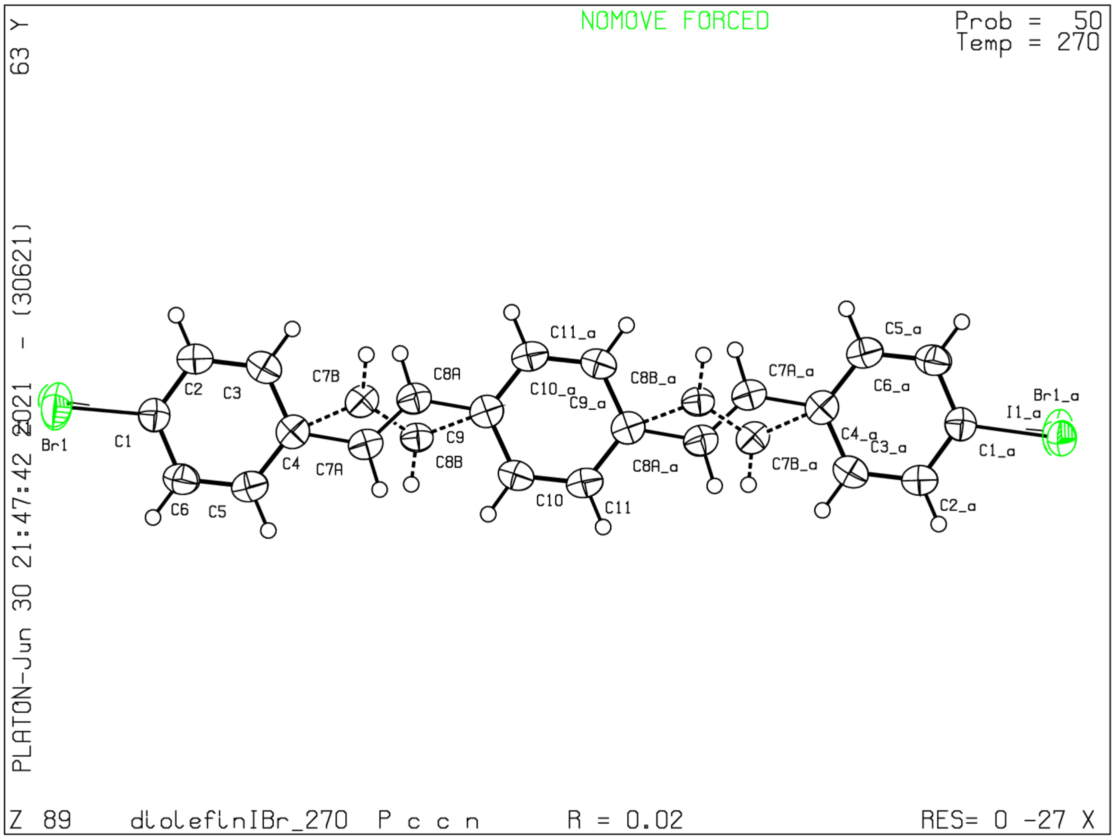

Datablock diolefinIBr_290 - ellipsoid plot

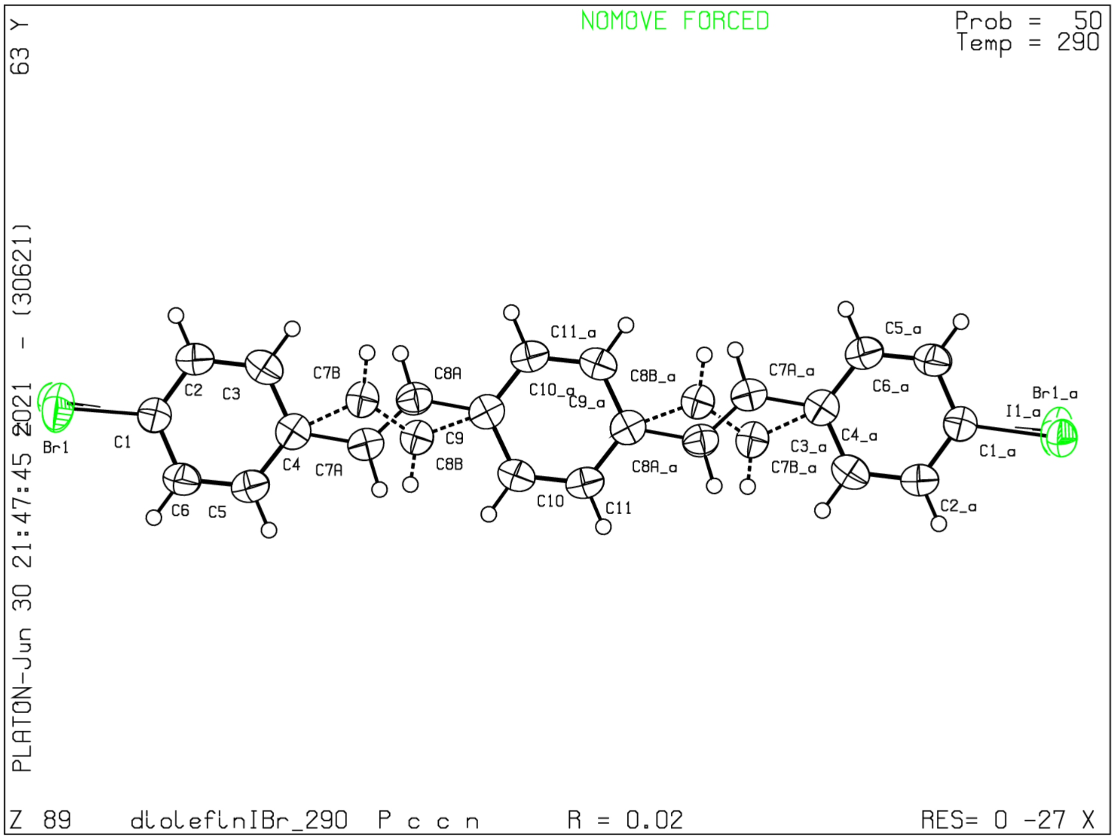

Supplement: Supplementary file 3 [file diolefin-I Br.pdf › diolefin-I Br.pdf]
