# Supplementary material for: Differences in thermal expansion and motion ability for herringbone and face-to-face π-stacked solids
Source: IUCrJ. 2021 Nov 3;9(Pt 1):31–42. doi: 10.1107/S2052252521009593 (PMC8733877; doi:10.1107/S2052252521009593)

checkCIF/PLATON report

Structure factors have been supplied for datablock(s) imineBr_190K, imineBr_210K, imineBr_230K, imineBr_250K, imineBr_270K, imineBr_290K, imineI_190K, imineI_210K, imineI_230K, imineI_250K, imineI_270K, imineI_290K

THIS REPORT IS FOR GUIDANCE ONLY. IF USED AS PART OF A REVIEW PROCEDURE FOR PUBLICATION, IT SHOULD NOT REPLACE THE EXPERTISE OF AN EXPERIENCED CRYSTALLOGRAPHIC REFEREE.

No syntax errors found.   CIF dictionary   Interpreting this report

Datablock: imineI_190K

Bond precision:	= 0.0000 A		Wavelength=1.54178
Cell:	a=7.38838(8)	b=28.6946(2)	c=5.98258(6)
	alpha=90	beta=90	gamma=90
Temperature:	190 K		

	Calculated	Reported
Volume	1268.35(2)	1268.35(2)
Space group	P c c n	P c c n
Hall group	-P 2ab 2ac	-P 2ab 2ac
Moiety formula	C11.36 H7.87 I1.75 N0.87, C1.64 H1.13 I0.25 N0.13	?
Sum formula	C13 H9 I2 N	C13 H9 I2 N
Mr	433.01	433.01
Dx,g cm-3	2.268	2.268
Z	4	4
Mu (mm-1)	38.711	38.711
F000	800.0	800.0
F000’	800.31	
h,k,lmax	9,36,7	9,36,7
Nref	1345	1322
Tmin,Tmax	0.011,0.081	0.016,0.245
Tmin’	0.002	

Correction method= # Reported T Limits: Tmin=0.016 Tmax=0.245
AbsCorr = GAUSSIAN

Data completeness= 0.983   Theta(max)= 77.152

R(reflections)= 0.0291(1193)   wR2(reflections)= 0.0844(1322)

S = 1.105   Npar= 154

The following ALERTS were generated. Each ALERT has the format
test-name_ALERT_alert-type_alert-level.
Click on the hyperlinks for more details of the test.

Alert level C

```
PLAT088_ALERT_3_C Poor Data / Parameter Ratio ....................       8.58 Note
```

Author Response: Due to the whole molecule disorder of the structure.

Alert level G

```
PLAT002_ALERT_2_G Number of Distance or Angle Restraints on AtSite          8 Note
PLAT003_ALERT_2_G Number of Uiso or Uij Restrained non-H Atoms ...         18 Report
PLAT142_ALERT_4_G s.u. on b - Axis Small or Missing ..............    0.00020 Ang.
PLAT172_ALERT_4_G The CIF-Embedded .res File Contains DFIX Records          3 Report
PLAT178_ALERT_4_G The CIF-Embedded .res File Contains SIMU Records          2 Report
PLAT187_ALERT_4_G The CIF-Embedded .res File Contains RIGU Records          1 Report
PLAT301_ALERT_3_G Main Residue  Disorder ..............(Resd  1  )       100% Note
PLAT302_ALERT_4_G Anion/Solvent/Minor-Residue Disorder (Resd  2  )       100% Note
PLAT304_ALERT_4_G Non-Integer Number of Atoms in ..... (Resd  1  )      21.85 Check
PLAT304_ALERT_4_G Non-Integer Number of Atoms in ..... (Resd  2  )       3.15 Check
PLAT779_ALERT_4_G Suspect or Irrelevant (Bond) Angle(s) in CIF ...       6.00 Deg.
              C7A  -C4A  -N1A     1_555   1_555   5_665 ......     #   15 Check
PLAT779_ALERT_4_G Suspect or Irrelevant (Bond) Angle(s) in CIF ...      18.00 Deg.
              N1B  -C4B  -C7B     5_665   1_555   1_555 ......     #   38 Check
PLAT860_ALERT_3_G Number of Least-Squares Restraints .............        370 Note
PLAT912_ALERT_4_G Missing # of FCF Reflections Above STh/L=  0.600         23 Note
```

```
   0 ALERT level A = Most likely a serious problem - resolve or explain
   0 ALERT level B = A potentially serious problem, consider carefully
   1 ALERT level C = Check. Ensure it is not caused by an omission or oversight
  14 ALERT level G = General information/check it is not something unexpected

   0 ALERT type 1 CIF construction/syntax error, inconsistent or missing data
   2 ALERT type 2 Indicator that the structure model may be wrong or deficient
   3 ALERT type 3 Indicator that the structure quality may be low
  10 ALERT type 4 Improvement, methodology, query or suggestion
   0 ALERT type 5 Informative message, check
```

Datablock: imineI_210K

```
Bond precision:   = 0.0000 A              Wavelength=1.54178

Cell:             a=7.40853(8)      b=28.6988(2)      c=5.99136(5)
                  alpha=90          beta=90           gamma=90
Temperature:      210 K
```

	Calculated	Reported
Volume	1273.86(2)	1273.86(2)
Space group	P c c n	P c c n
Hall group	-P 2ab 2ac	-P 2ab 2ac
Moiety formula	C11.21 H7.76 I1.72 N0.86, C1.79 H1.24 I0.28 N0.14	?
Sum formula	C13 H9 I2 N	C13 H9 I2 N
Mr	433.01	433.01
Dx,g cm-3	2.258	2.258
Z	4	4
Mu (mm-1)	38.544	38.544
F000	800.0	800.0
F000’	800.31	
h,k,lmax	9,36,7	9,36,7
Nref	1348	1327
Tmin,Tmax	0.011,0.082	0.016,0.246
Tmin’	0.002	

Correction method= # Reported T Limits: Tmin=0.016 Tmax=0.246
AbsCorr = GAUSSIAN

Data completeness= 0.984 Theta(max)= 77.092

R(reflections)= 0.0271(1179) wR2(reflections)= 0.0784(1327)

S = 1.094 Npar= 154

The following ALERTS were generated. Each ALERT has the format
test-name_ALERT_alert-type_alert-level.
Click on the hyperlinks for more details of the test.

Alert level C

PLAT088_ALERT_3_C Poor Data / Parameter Ratio .... 8.62 Note

Author Response: Due to the whole molecule disorder of the structure.

Alert level G

```
PLAT002_ALERT_2_G Number of Distance or Angle Restraints on AtSite          8 Note
PLAT003_ALERT_2_G Number of Uiso or Uij Restrained non-H Atoms ...         18 Report
PLAT142_ALERT_4_G s.u. on b - Axis Small or Missing .............     0.00020 Ang.
PLAT143_ALERT_4_G s.u. on c - Axis Small or Missing .............     0.00005 Ang.
PLAT172_ALERT_4_G The CIF-Embedded .res File Contains DFIX Records          3 Report
PLAT178_ALERT_4_G The CIF-Embedded .res File Contains SIMU Records          2 Report
PLAT187_ALERT_4_G The CIF-Embedded .res File Contains RIGU Records          1 Report
PLAT301_ALERT_3_G Main Residue  Disorder .............(Resd  1  )        100% Note
PLAT302_ALERT_4_G Anion/Solvent/Minor-Residue Disorder (Resd  2  )        100% Note
PLAT304_ALERT_4_G Non-Integer Number of Atoms in ..... (Resd  1  )       21.55 Check
PLAT304_ALERT_4_G Non-Integer Number of Atoms in ..... (Resd  2  )        3.45 Check
PLAT722_ALERT_1_G Angle   Calc      126.00, Rep      124.20 Dev...        1.80 Degree
```

```
              N1B     -C7B     -H7B        1_555  1_555  1_555     #   45 Check
PLAT779_ALERT_4_G Suspect or Irrelevant (Bond) Angle(s) in CIF ...       8.50 Deg.
              N1A  -C4A  -C7A     5_665   1_555   1_555 ......     #   15 Check
PLAT860_ALERT_3_G Number of Least-Squares Restraints .............        370 Note
PLAT912_ALERT_4_G Missing # of FCF Reflections Above STh/L=  0.600         20 Note
PLAT954_ALERT_1_G Reported (CIF) and Actual (FCF) Kmax Differ by .          1 Units
PLAT992_ALERT_5_G Repd & Actual _reflns_number_gt Values Differ by          2 Check
```

```
   0 ALERT level A = Most likely a serious problem - resolve or explain
   0 ALERT level B = A potentially serious problem, consider carefully
   1 ALERT level C = Check. Ensure it is not caused by an omission or oversight
  17 ALERT level G = General information/check it is not something unexpected

   2 ALERT type 1 CIF construction/syntax error, inconsistent or missing data
   2 ALERT type 2 Indicator that the structure model may be wrong or deficient
   3 ALERT type 3 Indicator that the structure quality may be low
  10 ALERT type 4 Improvement, methodology, query or suggestion
   1 ALERT type 5 Informative message, check
```

Datablock: imineI_230K

Bond precision: = 0.0000 A Wavelength=1.54178

Cell:	a=7.42795(8)	b=28.7031(2)	c=5.99925(6)
	alpha=90	beta=90	gamma=90
Temperature:	230 K		

	Calculated	Reported
Volume	1279.07(2)	1279.07(2)
Space group	P c c n	P c c n
Hall group	-P 2ab 2ac	-P 2ab 2ac
Moiety formula	C10.86 H7.52 I1.67 N0.84, C2.14 H1.48 I0.33 N0.16	?
Sum formula	C13 H9 I2 N	C13 H9 I2 N
Mr	433.01	433.01
Dx,g cm-3	2.249	2.249
Z	4	4
Mu (mm-1)	38.387	38.386
F000	800.0	800.0
F000’	800.31	
h,k,lmax	9,36,7	9,36,7
Nref	1357	1326
Tmin,Tmax	0.012,0.082	0.015,0.245
Tmin’	0.002	

Correction method= # Reported T Limits: Tmin=0.015 Tmax=0.245
AbsCorr = GAUSSIAN

```
Data completeness= 0.977          Theta(max)= 77.312

R(reflections)= 0.0304( 1151)     wR2(reflections)= 0.0840( 1326)

S = 1.096                 Npar= 154
```

The following ALERTS were generated. Each ALERT has the format
test-name_ALERT_alert-type_alert-level.
Click on the hyperlinks for more details of the test.

Alert level C

```
PLAT088_ALERT_3_C Poor Data / Parameter Ratio ....................       8.61 Note
```

Author Response: Due to the whole molecule disorder of the structure.

```
PLAT911_ALERT_3_C Missing FCF Refl Between Thmin & STh/L=    0.600          7 Report
```

Alert level G

```
PLAT002_ALERT_2_G Number of Distance or Angle Restraints on AtSite          8 Note
PLAT003_ALERT_2_G Number of Uiso or Uij Restrained non-H Atoms ...         18 Report
PLAT142_ALERT_4_G s.u. on b - Axis Small or Missing .............    0.00020 Ang.
PLAT172_ALERT_4_G The CIF-Embedded .res File Contains DFIX Records          3 Report
PLAT178_ALERT_4_G The CIF-Embedded .res File Contains SIMU Records          2 Report
PLAT187_ALERT_4_G The CIF-Embedded .res File Contains RIGU Records          1 Report
PLAT301_ALERT_3_G Main Residue  Disorder .............(Resd  1  )       100% Note
PLAT302_ALERT_4_G Anion/Solvent/Minor-Residue Disorder (Resd  2  )       100% Note
PLAT304_ALERT_4_G Non-Integer Number of Atoms in ..... (Resd  1  )      20.88 Check
PLAT304_ALERT_4_G Non-Integer Number of Atoms in ..... (Resd  2  )       4.12 Check
PLAT779_ALERT_4_G Suspect or Irrelevant (Bond) Angle(s) in CIF ...       9.00 Deg.
              C7A  -C4A  -N1A     1_555   1_555   5_665 ......     #   15 Check
PLAT860_ALERT_3_G Number of Least-Squares Restraints ............        370 Note
PLAT910_ALERT_3_G Missing # of FCF Reflection(s) Below Theta(Min).          1 Note
PLAT912_ALERT_4_G Missing # of FCF Reflections Above STh/L=  0.600         22 Note
PLAT954_ALERT_1_G Reported (CIF) and Actual (FCF) Kmax Differ by .          1 Units
```

---

   0 **ALERT level A** = Most likely a serious problem - resolve or explain
   0 **ALERT level B** = A potentially serious problem, consider carefully
   2 **ALERT level C** = Check. Ensure it is not caused by an omission or oversight
  15 **ALERT level G** = General information/check it is not something unexpected

```
   1 ALERT type 1 CIF construction/syntax error, inconsistent or missing data
   2 ALERT type 2 Indicator that the structure model may be wrong or deficient
   5 ALERT type 3 Indicator that the structure quality may be low
   9 ALERT type 4 Improvement, methodology, query or suggestion
   0 ALERT type 5 Informative message, check
```

Datablock: imineI_250K

```
Bond precision:  = 0.0000 A            Wavelength=1.54178
```

Cell:	a=7.44717(12)	b=28.7099(3)	c=6.00627(9)
	alpha=90	beta=90	gamma=90
Temperature:	250 K		

	Calculated	Reported
Volume	1284.19(3)	1284.18(3)
Space group	P c c n	P c c n
Hall group	-P 2ab 2ac	-P 2ab 2ac
Moiety formula	C10.84 H7.51 I1.67 N0.83, C2.16 H1.49 I0.33 N0.17	?
Sum formula	C13 H9 I2 N	C13 H9 I2 N
Mr	433.01	433.01
Dx,g cm-3	2.240	2.240
Z	4	4
Mu (mm-1)	38.233	38.234
F000	800.0	800.0
F000’	800.31	
h,k,lmax	9,36,7	9,36,7
Nref	1359	1319
Tmin,Tmax	0.012,0.083	0.015,0.245
Tmin’	0.002	

Correction method= # Reported T Limits: Tmin=0.015 Tmax=0.245
AbsCorr = GAUSSIAN

Data completeness= 0.971 Theta(max)= 77.045

R(reflections)= 0.0316(1145) wR2(reflections)= 0.0871(1319)

S = 1.069 Npar= 154

The following ALERTS were generated. Each ALERT has the format
test-name_ALERT_alert-type_alert-level.
Click on the hyperlinks for more details of the test.

Alert level C

PLAT088_ALERT_3_C Poor Data / Parameter Ratio .... 8.56 Note

Author Response: Due to the whole molecule disorder of the structure.

PLAT911_ALERT_3_C Missing FCF Refl Between Thmin & STh/L= 0.600 6 Report

---

Alert level G

PLAT002_ALERT_2_G Number of Distance or Angle Restraints on AtSite 8 Note
PLAT003_ALERT_2_G Number of Uiso or Uij Restrained non-H Atoms ... 18 Report
PLAT172_ALERT_4_G The CIF-Embedded .res File Contains DFIX Records 3 Report
PLAT178_ALERT_4_G The CIF-Embedded .res File Contains SIMU Records 2 Report
PLAT187_ALERT_4_G The CIF-Embedded .res File Contains RIGU Records 1 Report

```
PLAT301_ALERT_3_G Main Residue  Disorder .............(Resd  1  )       100% Note
PLAT302_ALERT_4_G Anion/Solvent/Minor-Residue Disorder (Resd  2  )       100% Note
PLAT304_ALERT_4_G Non-Integer Number of Atoms in ..... (Resd  1  )      20.85 Check
PLAT304_ALERT_4_G Non-Integer Number of Atoms in ..... (Resd  2  )       4.15 Check
PLAT779_ALERT_4_G Suspect or Irrelevant (Bond) Angle(s) in CIF ...       7.20 Deg.
              N1A  -C4A  -C7A     5_665   1_555   1_555 ......     #   15 Check
PLAT779_ALERT_4_G Suspect or Irrelevant (Bond) Angle(s) in CIF ...      42.80 Deg.
              H7A  -C7A  -H7B     1_555   1_555   5_665 ......     #   27 Check
PLAT860_ALERT_3_G Number of Least-Squares Restraints ............         370 Note
PLAT912_ALERT_4_G Missing # of FCF Reflections Above STh/L=  0.600         34 Note
PLAT954_ALERT_1_G Reported (CIF) and Actual (FCF) Kmax Differ by .          1 Units
```

---

   0 **ALERT level A** = Most likely a serious problem - resolve or explain
   0 **ALERT level B** = A potentially serious problem, consider carefully
   2 **ALERT level C** = Check. Ensure it is not caused by an omission or oversight
  14 **ALERT level G** = General information/check it is not something unexpected

   1 ALERT type 1 CIF construction/syntax error, inconsistent or missing data
   2 ALERT type 2 Indicator that the structure model may be wrong or deficient
   4 ALERT type 3 Indicator that the structure quality may be low
   9 ALERT type 4 Improvement, methodology, query or suggestion
   0 ALERT type 5 Informative message, check

Datablock: imineI_270K

Bond precision: = 0.0000 A   Wavelength=1.54178

Cell:	a=7.46643(8)	b=28.7114(2)	c=6.01595(6)
	alpha=90	beta=90	gamma=90
Temperature:	270 K		

	Calculated	Reported
Volume	1289.65(2)	1289.65(2)
Space group	P c c n	P c c n
Hall group	-P 2ab 2ac	-P 2ab 2ac
Moiety formula	C10.31 H7.14 I1.59 N0.79, C2.69 H1.86 I0.41 N0.21	?
Sum formula	C13 H9 I2 N	C13 H9 I2 N
Mr	433.01	433.01
Dx,g cm-3	2.230	2.230
Z	4	4
Mu (mm-1)	38.072	38.072
F000	800.0	800.0
F000’	800.31	
h,k,lmax	9,36,7	9,36,7
Nref	1372	1305
Tmin,Tmax	0.012,0.084	0.015,0.245
Tmin’	0.002	

```
Correction method= # Reported T Limits: Tmin=0.015 Tmax=0.245
AbsCorr = GAUSSIAN

Data completeness= 0.951          Theta(max)= 77.302

R(reflections)= 0.0295( 1107)     wR2(reflections)= 0.0845( 1305)

S = 1.081                  Npar= 154
```

The following ALERTS were generated. Each ALERT has the format
test-name_ALERT_alert-type_alert-level.
Click on the hyperlinks for more details of the test.

Alert level C

```
PLAT088_ALERT_3_C Poor Data / Parameter Ratio ....................       8.47 Note
```

Author Response: Due to the whole molecule disorder of the structure.

```
PLAT911_ALERT_3_C Missing FCF Refl Between Thmin & STh/L=    0.600          9 Report
```

Alert level G

```
PLAT002_ALERT_2_G Number of Distance or Angle Restraints on AtSite          8 Note
PLAT003_ALERT_2_G Number of Uiso or Uij Restrained non-H Atoms ...         18 Report
PLAT142_ALERT_4_G s.u. on b - Axis Small or Missing .............    0.00020 Ang.
PLAT143_ALERT_4_G s.u. on c - Axis Small or Missing .............    0.00006 Ang.
PLAT172_ALERT_4_G The CIF-Embedded .res File Contains DFIX Records          3 Report
PLAT178_ALERT_4_G The CIF-Embedded .res File Contains SIMU Records          2 Report
PLAT187_ALERT_4_G The CIF-Embedded .res File Contains RIGU Records          1 Report
PLAT301_ALERT_3_G Main Residue  Disorder .............(Resd  1  )       100% Note
PLAT302_ALERT_4_G Anion/Solvent/Minor-Residue Disorder (Resd  2  )       100% Note
PLAT304_ALERT_4_G Non-Integer Number of Atoms in ..... (Resd  1  )      19.83 Check
PLAT304_ALERT_4_G Non-Integer Number of Atoms in ..... (Resd  2  )       5.17 Check
PLAT779_ALERT_4_G Suspect or Irrelevant (Bond) Angle(s) in CIF ...       6.00 Deg.
              C7A  -C4A  -N1A     1_555   1_555   5_665 ......     #   15 Check
PLAT860_ALERT_3_G Number of Least-Squares Restraints ............        370 Note
PLAT912_ALERT_4_G Missing # of FCF Reflections Above STh/L=  0.600         57 Note
PLAT954_ALERT_1_G Reported (CIF) and Actual (FCF) Kmax Differ by .          1 Units
```

---

0 **ALERT level A** = Most likely a serious problem - resolve or explain
0 **ALERT level B** = A potentially serious problem, consider carefully
2 **ALERT level C** = Check. Ensure it is not caused by an omission or oversight
15 **ALERT level G** = General information/check it is not something unexpected

1 ALERT type 1 CIF construction/syntax error, inconsistent or missing data
2 ALERT type 2 Indicator that the structure model may be wrong or deficient
4 ALERT type 3 Indicator that the structure quality may be low
10 ALERT type 4 Improvement, methodology, query or suggestion
0 ALERT type 5 Informative message, check

Datablock: imineI_290K

Bond precision: = 0.0000 A Wavelength=1.54178

Cell:	a=7.4880(1)	b=28.7165(2)	c=6.0248(1)
	alpha=90	beta=90	gamma=90
Temperature:	290 K		

	Calculated	Reported
Volume	1295.51(3)	1295.51(3)
Space group	P c c n	P c c n
Hall group	-P 2ab 2ac	-P 2ab 2ac
Moiety formula	C10.10 H7 I1.55 N0.78, C2.90 H2 I0.45 N0.22	?
Sum formula	C13 H9 I2 N	C13 H9 I2 N
Mr	433.01	433.01
Dx,g cm-3	2.220	2.220
Z	4	4
Mu (mm-1)	37.899	37.899
F000	800.0	800.0
F000'	800.31	
h,k,lmax	9,36,7	9,35,7
Nref	1374	1310
Tmin,Tmax	0.012,0.085	0.015,0.245
Tmin'	0.002	

Correction method= # Reported T Limits: Tmin=0.015 Tmax=0.245
AbsCorr = GAUSSIAN

Data completeness= 0.953 Theta(max)= 77.069

R(reflections)= 0.0312(1084) wR2(reflections)= 0.0905(1310)

S = 1.027 Npar= 154

The following ALERTS were generated. Each ALERT has the format
test-name_ALERT_alert-type_alert-level.
Click on the hyperlinks for more details of the test.

Alert level C

PLAT088_ALERT_3_C Poor Data / Parameter Ratio .... 8.51 Note

Author Response: Due to the whole molecule disorder of the structure.

PLAT911_ALERT_3_C Missing FCF Refl Between Thmin & STh/L= 0.600 10 Report

---

Alert level G

PLAT002_ALERT_2_G Number of Distance or Angle Restraints on AtSite 8 Note
PLAT003_ALERT_2_G Number of Uiso or Uij Restrained non-H Atoms ... 18 Report
PLAT142_ALERT_4_G s.u. on b - Axis Small or Missing .... 0.00020 Ang.

```
PLAT172_ALERT_4_G The CIF-Embedded .res File Contains DFIX Records          3 Report
PLAT178_ALERT_4_G The CIF-Embedded .res File Contains SIMU Records          2 Report
PLAT187_ALERT_4_G The CIF-Embedded .res File Contains RIGU Records          1 Report
PLAT301_ALERT_3_G Main Residue  Disorder .............(Resd  1  )       100% Note
PLAT302_ALERT_4_G Anion/Solvent/Minor-Residue Disorder (Resd  2  )       100% Note
PLAT304_ALERT_4_G Non-Integer Number of Atoms in ..... (Resd  1  )      19.43 Check
PLAT304_ALERT_4_G Non-Integer Number of Atoms in ..... (Resd  2  )       5.57 Check
PLAT779_ALERT_4_G Suspect or Irrelevant (Bond) Angle(s) in CIF ...       7.90 Deg.
              N1A  -C4A  -C7A     5_665   1_555   1_555 ......     #   15 Check
PLAT860_ALERT_3_G Number of Least-Squares Restraints .............        364 Note
PLAT912_ALERT_4_G Missing # of FCF Reflections Above STh/L=  0.600         54 Note
PLAT992_ALERT_5_G Repd & Actual _reflns_number_gt Values Differ by          2 Check
```

---

   0 **ALERT level A** = Most likely a serious problem - resolve or explain
   0 **ALERT level B** = A potentially serious problem, consider carefully
   2 **ALERT level C** = Check. Ensure it is not caused by an omission or oversight
  14 **ALERT level G** = General information/check it is not something unexpected

   0 ALERT type 1 CIF construction/syntax error, inconsistent or missing data
   2 ALERT type 2 Indicator that the structure model may be wrong or deficient
   4 ALERT type 3 Indicator that the structure quality may be low
   9 ALERT type 4 Improvement, methodology, query or suggestion
   1 ALERT type 5 Informative message, check

Datablock: imineBr_190K

Bond precision: C-C = 0.0038 A   Wavelength=1.54178

Cell:	a=3.98510(7)	b=5.86861(9)	c=24.9445(4)
	alpha=90	beta=92.4943(15)	gamma=90
Temperature:	190 K		

	Calculated	Reported
Volume	582.824(17)	582.823(17)
Space group	P 21/c	P 21/c
Hall group	-P 2ybc	-P 2ybc
Moiety formula	C13 H9 Br2 N	?
Sum formula	C13 H9 Br2 N	C13 H9 Br2 N
Mr	339.01	339.03
Dx,g cm-3	1.932	1.932
Z	2	2
Mu (mm-1)	8.561	8.561
F000	328.0	328.0
F000’	325.81	
h,k,lmax	5,7,31	4,7,31
Nref	1223	1208
Tmin,Tmax	0.466,0.902	0.650,1.000
Tmin’	0.353	

Correction method= # Reported T Limits: Tmin=0.650 Tmax=1.000
AbsCorr = GAUSSIAN

Data completeness= 0.988   Theta(max)= 77.181

R(reflections)= 0.0249(1123)   wR2(reflections)= 0.0691(1208)

S = 1.096   Npar= 83

The following ALERTS were generated. Each ALERT has the format
test-name_ALERT_alert-type_alert-level.
Click on the hyperlinks for more details of the test.

Alert level G

```
PLAT002_ALERT_2_G Number of Distance or Angle Restraints on AtSite          1 Note
PLAT003_ALERT_2_G Number of Uiso or Uij Restrained non-H Atoms ...          3 Report
PLAT172_ALERT_4_G The CIF-Embedded .res File Contains DFIX Records          1 Report
PLAT178_ALERT_4_G The CIF-Embedded .res File Contains SIMU Records          1 Report
PLAT187_ALERT_4_G The CIF-Embedded .res File Contains RIGU Records          1 Report
PLAT300_ALERT_4_G Atom Site Occupancy of N1         Constrained at        0.5 Check
PLAT300_ALERT_4_G Atom Site Occupancy of C7         Constrained at        0.5 Check
PLAT300_ALERT_4_G Atom Site Occupancy of H7         Constrained at        0.5 Check
PLAT301_ALERT_3_G Main Residue  Disorder ..............(Resd  1  )        13% Note
PLAT410_ALERT_2_G Short Intra H...H Contact  H3       ..H7       .       2.10 Ang.
                                                 1-x,-y,1-z  =      3_656 Check
PLAT789_ALERT_4_G Atoms with Negative _atom_site_disorder_group  #          3 Check
PLAT860_ALERT_3_G Number of Least-Squares Restraints .............         70 Note
PLAT912_ALERT_4_G Missing # of FCF Reflections Above STh/L=  0.600         12 Note
PLAT933_ALERT_2_G Number of OMIT Records in Embedded .res File ...          1 Note
PLAT978_ALERT_2_G Number C-C Bonds with Positive Residual Density.          3 Info
```

---

0 **ALERT level A** = Most likely a serious problem - resolve or explain
0 **ALERT level B** = A potentially serious problem, consider carefully
0 **ALERT level C** = Check. Ensure it is not caused by an omission or oversight
15 **ALERT level G** = General information/check it is not something unexpected

0 ALERT type 1 CIF construction/syntax error, inconsistent or missing data
5 ALERT type 2 Indicator that the structure model may be wrong or deficient
2 ALERT type 3 Indicator that the structure quality may be low
8 ALERT type 4 Improvement, methodology, query or suggestion
0 ALERT type 5 Informative message, check

Datablock: imineBr_210K

Bond precision: C-C = 0.0033 A   Wavelength=1.54178

Cell:	a=3.99682(7)	b=5.87524(8)	c=24.9435(4)
	alpha=90	beta=92.5186(13)	gamma=90
Temperature:	210 K		

	Calculated	Reported
Volume	585.164(16)	585.164(15)
Space group	P 21/c	P 21/c
Hall group	-P 2ybc	-P 2ybc
Moiety formula	C13 H9 Br2 N	?
Sum formula	C13 H9 Br2 N	C13 H9 Br2 N
Mr	339.01	339.03
Dx,g cm-3	1.924	1.924
Z	2	2
Mu (mm-1)	8.527	8.527
F000	328.0	328.0
F000’	325.81	
h,k,lmax	5,7,31	4,7,31
Nref	1233	1209
Tmin,Tmax	0.468,0.903	0.650,1.000
Tmin’	0.354	

Correction method= # Reported T Limits: Tmin=0.650 Tmax=1.000
AbsCorr = GAUSSIAN

Data completeness= 0.981 Theta(max)= 77.156

R(reflections)= 0.0251(1122) wR2(reflections)= 0.0673(1209)

S = 1.081 Npar= 83

The following ALERTS were generated. Each ALERT has the format
test-name_ALERT_alert-type_alert-level.
Click on the hyperlinks for more details of the test.

Alert level G

```
PLAT002_ALERT_2_G Number of Distance or Angle Restraints on AtSite          1 Note
PLAT003_ALERT_2_G Number of Uiso or Uij Restrained non-H Atoms ...           3 Report
PLAT172_ALERT_4_G The CIF-Embedded .res File Contains DFIX Records          1 Report
PLAT178_ALERT_4_G The CIF-Embedded .res File Contains SIMU Records          1 Report
PLAT187_ALERT_4_G The CIF-Embedded .res File Contains RIGU Records          1 Report
PLAT300_ALERT_4_G Atom Site Occupancy of N1         Constrained at        0.5 Check
PLAT300_ALERT_4_G Atom Site Occupancy of C7         Constrained at        0.5 Check
PLAT300_ALERT_4_G Atom Site Occupancy of H7         Constrained at        0.5 Check
PLAT301_ALERT_3_G Main Residue  Disorder ..............(Resd  1  )        13% Note
PLAT410_ALERT_2_G Short Intra H...H Contact  H3       ..H7       .       2.10 Ang.
                                                 1-x,-y,1-z  =      3_656 Check
PLAT789_ALERT_4_G Atoms with Negative _atom_site_disorder_group  #          3 Check
PLAT860_ALERT_3_G Number of Least-Squares Restraints .............         70 Note
PLAT912_ALERT_4_G Missing # of FCF Reflections Above STh/L=  0.600         15 Note
PLAT933_ALERT_2_G Number of OMIT Records in Embedded .res File ...          1 Note
PLAT978_ALERT_2_G Number C-C Bonds with Positive Residual Density.          4 Info
```

---

0 **ALERT level A** = Most likely a serious problem - resolve or explain
0 **ALERT level B** = A potentially serious problem, consider carefully
0 **ALERT level C** = Check. Ensure it is not caused by an omission or oversight
15 **ALERT level G** = General information/check it is not something unexpected

```
   0 ALERT type 1 CIF construction/syntax error, inconsistent or missing data
   5 ALERT type 2 Indicator that the structure model may be wrong or deficient
   2 ALERT type 3 Indicator that the structure quality may be low
   8 ALERT type 4 Improvement, methodology, query or suggestion
   0 ALERT type 5 Informative message, check
```

Datablock: imineBr_230K

```
Bond precision:  C-C = 0.0037 A          Wavelength=1.54178

Cell:           a=4.00914(7)    b=5.88041(8)       c=24.9488(4)
                alpha=90        beta=92.5377(13)   gamma=90
Temperature:    230 K

                Calculated                 Reported
Volume          587.601(16)                587.602(15)
Space group     P 21/c                     P 21/c
Hall group      -P 2ybc                    -P 2ybc
Moiety formula  C13 H9 Br2 N               ?
Sum formula     C13 H9 Br2 N               C13 H9 Br2 N
Mr              339.01                     339.03
Dx,g cm-3       1.916                      1.916
Z               2                          2
Mu (mm-1)       8.491                      8.491
F000            328.0                      328.0
F000'           325.81
h,k,lmax        5,7,31                     4,7,31
Nref            1239                       1209
Tmin,Tmax       0.469,0.903                0.650,1.000
Tmin'           0.356

Correction method= # Reported T Limits: Tmin=0.650 Tmax=1.000
AbsCorr = GAUSSIAN

Data completeness= 0.976          Theta(max)= 77.330

R(reflections)= 0.0255( 1101)     wR2(reflections)= 0.0682( 1209)

S = 1.087                    Npar= 83
```

The following ALERTS were generated. Each ALERT has the format
       test-name_ALERT_alert-type_alert-level.
Click on the hyperlinks for more details of the test.

Alert level G

```
PLAT002_ALERT_2_G Number of Distance or Angle Restraints on AtSite          1 Note
PLAT003_ALERT_2_G Number of Uiso or Uij Restrained non-H Atoms ...          3 Report
```

```
PLAT172_ALERT_4_G The CIF-Embedded .res File Contains DFIX Records          1 Report
PLAT178_ALERT_4_G The CIF-Embedded .res File Contains SIMU Records          1 Report
PLAT187_ALERT_4_G The CIF-Embedded .res File Contains RIGU Records          1 Report
PLAT300_ALERT_4_G Atom Site Occupancy of N1         Constrained at        0.5 Check
PLAT300_ALERT_4_G Atom Site Occupancy of C7         Constrained at        0.5 Check
PLAT300_ALERT_4_G Atom Site Occupancy of H7         Constrained at        0.5 Check
PLAT301_ALERT_3_G Main Residue  Disorder ..............(Resd  1  )        13% Note
PLAT410_ALERT_2_G Short Intra H...H Contact  H3       ..H7       .       2.10 Ang.
                                                 1-x,-y,1-z  =      3_656 Check
PLAT789_ALERT_4_G Atoms with Negative _atom_site_disorder_group  #          3 Check
PLAT860_ALERT_3_G Number of Least-Squares Restraints .............         70 Note
PLAT912_ALERT_4_G Missing # of FCF Reflections Above STh/L=  0.600         19 Note
PLAT933_ALERT_2_G Number of OMIT Records in Embedded .res File ...          1 Note
PLAT978_ALERT_2_G Number C-C Bonds with Positive Residual Density.          2 Info
```

```
   0 ALERT level A = Most likely a serious problem - resolve or explain
   0 ALERT level B = A potentially serious problem, consider carefully
   0 ALERT level C = Check. Ensure it is not caused by an omission or oversight
  15 ALERT level G = General information/check it is not something unexpected

   0 ALERT type 1 CIF construction/syntax error, inconsistent or missing data
   5 ALERT type 2 Indicator that the structure model may be wrong or deficient
   2 ALERT type 3 Indicator that the structure quality may be low
   8 ALERT type 4 Improvement, methodology, query or suggestion
   0 ALERT type 5 Informative message, check
```

Datablock: imineBr_250K

```
Bond precision:  C-C = 0.0038 A          Wavelength=1.54178

Cell:            a=4.02157(7)    b=5.88488(9)         c=24.9463(4)
                 alpha=90        beta=92.5537(14)     gamma=90
Temperature:     250 K

                 Calculated                 Reported
Volume           589.804(17)                589.805(16)
Space group      P 21/c                     P 21/c
Hall group       -P 2ybc                    -P 2ybc
Moiety formula   C13 H9 Br2 N               ?
Sum formula      C13 H9 Br2 N               C13 H9 Br2 N
Mr               339.01                     339.03
Dx,g cm-3        1.909                      1.909
Z                2                          2
Mu (mm-1)        8.460                      8.460
F000             328.0                      328.0
F000'            325.81
h,k,lmax         5,7,31                     4,7,31
Nref             1243                       1213
Tmin,Tmax        0.471,0.903                0.650,1.000
Tmin'            0.357
```

```
Correction method= # Reported T Limits: Tmin=0.650 Tmax=1.000
AbsCorr = GAUSSIAN

Data completeness= 0.976          Theta(max)= 77.130

R(reflections)= 0.0272( 1103)     wR2(reflections)= 0.0708( 1213)

S = 1.101                  Npar= 83
```

The following ALERTS were generated. Each ALERT has the format
test-name_ALERT_alert-type_alert-level.
Click on the hyperlinks for more details of the test.

Alert level G

```
PLAT002_ALERT_2_G Number of Distance or Angle Restraints on AtSite          1 Note
PLAT003_ALERT_2_G Number of Uiso or Uij Restrained non-H Atoms ...          3 Report
PLAT172_ALERT_4_G The CIF-Embedded .res File Contains DFIX Records          1 Report
PLAT178_ALERT_4_G The CIF-Embedded .res File Contains SIMU Records          1 Report
PLAT187_ALERT_4_G The CIF-Embedded .res File Contains RIGU Records          1 Report
PLAT300_ALERT_4_G Atom Site Occupancy of N1         Constrained at        0.5 Check
PLAT300_ALERT_4_G Atom Site Occupancy of C7         Constrained at        0.5 Check
PLAT300_ALERT_4_G Atom Site Occupancy of H7         Constrained at        0.5 Check
PLAT301_ALERT_3_G Main Residue  Disorder ..............(Resd  1  )        13% Note
PLAT410_ALERT_2_G Short Intra H...H Contact  H3       ..H7       .       2.11 Ang.
                                                 1-x,-y,1-z  =      3_656 Check
PLAT789_ALERT_4_G Atoms with Negative _atom_site_disorder_group  #          3 Check
PLAT860_ALERT_3_G Number of Least-Squares Restraints .............         70 Note
PLAT912_ALERT_4_G Missing # of FCF Reflections Above STh/L=  0.600         16 Note
PLAT933_ALERT_2_G Number of OMIT Records in Embedded .res File ...          1 Note
PLAT978_ALERT_2_G Number C-C Bonds with Positive Residual Density.          4 Info
```

---

   0 **ALERT level A** = Most likely a serious problem - resolve or explain
   0 **ALERT level B** = A potentially serious problem, consider carefully
   0 **ALERT level C** = Check. Ensure it is not caused by an omission or oversight
  15 **ALERT level G** = General information/check it is not something unexpected

```
   0 ALERT type 1 CIF construction/syntax error, inconsistent or missing data
   5 ALERT type 2 Indicator that the structure model may be wrong or deficient
   2 ALERT type 3 Indicator that the structure quality may be low
   8 ALERT type 4 Improvement, methodology, query or suggestion
   0 ALERT type 5 Informative message, check
```

Datablock: imineBr_270K

```
Bond precision:  C-C = 0.0040 A          Wavelength=1.54178

Cell:           a=4.03549(11)     b=5.88966(12)     c=24.9442(5)
                alpha=90          beta=92.566(2)    gamma=90
Temperature:    270 K
```

	Calculated	Reported
Volume	592.27(2)	592.27(2)
Space group	P 21/c	P 21/c
Hall group	-P 2ybc	-P 2ybc
Moiety formula	C13 H9 Br2 N	?
Sum formula	C13 H9 Br2 N	C13 H9 Br2 N
Mr	339.01	339.03
Dx,g cm-3	1.901	1.901
Z	2	2
Mu (mm-1)	8.424	8.424
F000	328.0	328.0
F000’	325.81	
h,k,lmax	5,7,31	4,7,31
Nref	1253	1200
Tmin,Tmax	0.472,0.904	0.646,1.000
Tmin’	0.358	

Correction method= # Reported T Limits: Tmin=0.646 Tmax=1.000
AbsCorr = GAUSSIAN

Data completeness= 0.958 Theta(max)= 77.322

R(reflections)= 0.0281(1078) wR2(reflections)= 0.0732(1200)

S = 1.109 Npar= 83

The following ALERTS were generated. Each ALERT has the format
test-name_ALERT_alert-type_alert-level.
Click on the hyperlinks for more details of the test.

Alert level C

```
PLAT911_ALERT_3_C Missing FCF Refl Between Thmin & STh/L=    0.600          4 Report
```

Alert level G

```
PLAT002_ALERT_2_G Number of Distance or Angle Restraints on AtSite          1 Note
PLAT003_ALERT_2_G Number of Uiso or Uij Restrained non-H Atoms ...          3 Report
PLAT172_ALERT_4_G The CIF-Embedded .res File Contains DFIX Records          1 Report
PLAT178_ALERT_4_G The CIF-Embedded .res File Contains SIMU Records          1 Report
PLAT187_ALERT_4_G The CIF-Embedded .res File Contains RIGU Records          1 Report
PLAT300_ALERT_4_G Atom Site Occupancy of N1         Constrained at        0.5 Check
PLAT300_ALERT_4_G Atom Site Occupancy of C7         Constrained at        0.5 Check
PLAT300_ALERT_4_G Atom Site Occupancy of H7         Constrained at        0.5 Check
PLAT301_ALERT_3_G Main Residue  Disorder ..............(Resd  1  )        13% Note
PLAT410_ALERT_2_G Short Intra H...H Contact  H3       ..H7       .       2.10 Ang.
                                                  1-x,-y,1-z  =      3_656 Check
PLAT789_ALERT_4_G Atoms with Negative _atom_site_disorder_group  #          3 Check
PLAT860_ALERT_3_G Number of Least-Squares Restraints .............         70 Note
PLAT912_ALERT_4_G Missing # of FCF Reflections Above STh/L=  0.600         34 Note
PLAT933_ALERT_2_G Number of OMIT Records in Embedded .res File ...          1 Note
PLAT978_ALERT_2_G Number C-C Bonds with Positive Residual Density.          3 Info
```

```
  0 ALERT level A = Most likely a serious problem - resolve or explain
  0 ALERT level B = A potentially serious problem, consider carefully
  1 ALERT level C = Check. Ensure it is not caused by an omission or oversight
 15 ALERT level G = General information/check it is not something unexpected

  0 ALERT type 1 CIF construction/syntax error, inconsistent or missing data
  5 ALERT type 2 Indicator that the structure model may be wrong or deficient
  3 ALERT type 3 Indicator that the structure quality may be low
  8 ALERT type 4 Improvement, methodology, query or suggestion
  0 ALERT type 5 Informative message, check
```

Datablock: imineBr_290K

```
Bond precision:  C-C = 0.0040 A          Wavelength=1.54178

Cell:           a=4.04957(9)    b=5.89465(11)       c=24.9465(5)
                alpha=90        beta=92.5589(18)     gamma=90
Temperature:    290 K

                Calculated                 Reported
Volume          594.90(2)                  594.90(2)
Space group     P 21/c                     P 21/c
Hall group      -P 2ybc                    -P 2ybc
Moiety formula  C13 H9 Br2 N               ?
Sum formula     C13 H9 Br2 N               C13 H9 Br2 N
Mr              339.01                     339.03
Dx,g cm-3       1.893                      1.893
Z               2                          2
Mu (mm-1)       8.387                      8.387
F000            328.0                      328.0
F000'           325.81
h,k,lmax        5,7,31                     4,7,31
Nref            1262                       1212
Tmin,Tmax       0.474,0.904                0.646,1.000
Tmin'           0.360

Correction method= # Reported T Limits: Tmin=0.646 Tmax=1.000
AbsCorr = GAUSSIAN

Data completeness= 0.960          Theta(max)= 77.332

R(reflections)= 0.0262( 1056)     wR2(reflections)= 0.0719( 1212)

S = 1.065                 Npar= 83
```

The following ALERTS were generated. Each ALERT has the format
test-name_ALERT_alert-type_alert-level.
Click on the hyperlinks for more details of the test.

Alert level C

```
PLAT911_ALERT_3_C Missing FCF Refl Between Thmin & STh/L=    0.600          8 Report
```

Alert level G

```
PLAT002_ALERT_2_G Number of Distance or Angle Restraints on AtSite          1 Note
PLAT003_ALERT_2_G Number of Uiso or Uij Restrained non-H Atoms ...          3 Report
PLAT172_ALERT_4_G The CIF-Embedded .res File Contains DFIX Records          1 Report
PLAT178_ALERT_4_G The CIF-Embedded .res File Contains SIMU Records          1 Report
PLAT187_ALERT_4_G The CIF-Embedded .res File Contains RIGU Records          1 Report
PLAT300_ALERT_4_G Atom Site Occupancy of N1         Constrained at        0.5 Check
PLAT300_ALERT_4_G Atom Site Occupancy of C7         Constrained at        0.5 Check
PLAT300_ALERT_4_G Atom Site Occupancy of H7         Constrained at        0.5 Check
PLAT301_ALERT_3_G Main Residue  Disorder ..............(Resd  1  )        13% Note
PLAT410_ALERT_2_G Short Intra H...H Contact  H3       ..H7       .       2.09 Ang.
                                                 1-x,-y,1-z  =      3_656 Check
PLAT789_ALERT_4_G Atoms with Negative _atom_site_disorder_group  #          3 Check
PLAT860_ALERT_3_G Number of Least-Squares Restraints .............         70 Note
PLAT912_ALERT_4_G Missing # of FCF Reflections Above STh/L=  0.600         24 Note
PLAT933_ALERT_2_G Number of OMIT Records in Embedded .res File ...          1 Note
PLAT978_ALERT_2_G Number C-C Bonds with Positive Residual Density.          2 Info
```

```
   0 ALERT level A = Most likely a serious problem - resolve or explain
   0 ALERT level B = A potentially serious problem, consider carefully
   1 ALERT level C = Check. Ensure it is not caused by an omission or oversight
  15 ALERT level G = General information/check it is not something unexpected

   0 ALERT type 1 CIF construction/syntax error, inconsistent or missing data
   5 ALERT type 2 Indicator that the structure model may be wrong or deficient
   3 ALERT type 3 Indicator that the structure quality may be low
   8 ALERT type 4 Improvement, methodology, query or suggestion
   0 ALERT type 5 Informative message, check
```

Validation response form

Please find below a validation response form (VRF) that can be filled in and pasted into your CIF.

```
# start Validation Reply Form
_vrf_PLAT911_imineI_230K
;
PROBLEM: Missing FCF Refl Between Thmin & STh/L=    0.600          7 Report
RESPONSE: ...
;
_vrf_PLAT911_imineI_250K
;
PROBLEM: Missing FCF Refl Between Thmin & STh/L=    0.600          6 Report
RESPONSE: ...
;
_vrf_PLAT911_imineI_270K
;
PROBLEM: Missing FCF Refl Between Thmin & STh/L=    0.600          9 Report
RESPONSE: ...
;
_vrf_PLAT911_imineI_290K
;
PROBLEM: Missing FCF Refl Between Thmin & STh/L=    0.600         10 Report
RESPONSE: ...
;
_vrf_PLAT911_imineBr_270K
```

```
;
PROBLEM: Missing FCF Refl Between Thmin & STh/L=    0.600          4 Report
RESPONSE: ...
;
_vrf_PLAT911_imineBr_290K
;
PROBLEM: Missing FCF Refl Between Thmin & STh/L=    0.600          8 Report
RESPONSE: ...
;
# end Validation Reply Form
```

It is advisable to attempt to resolve as many as possible of the alerts in all categories. Often the minor alerts point to easily fixed oversights, errors and omissions in your CIF or refinement strategy, so attention to these fine details can be worthwhile. In order to resolve some of the more serious problems it may be necessary to carry out additional measurements or structure refinements. However, the purpose of your study may justify the reported deviations and the more serious of these should normally be commented upon in the discussion or experimental section of a paper or in the "special_details" fields of the CIF. checkCIF was carefully designed to identify outliers and unusual parameters, but every test has its limitations and alerts that are not important in a particular case may appear. Conversely, the absence of alerts does not guarantee there are no aspects of the results needing attention. It is up to the individual to critically assess their own results and, if necessary, seek expert advice.

Publication of your CIF in IUCr journals

A basic structural check has been run on your CIF. These basic checks will be run on all CIFs submitted for publication in IUCr journals (*Acta Crystallographica*, *Journal of Applied Crystallography*, *Journal of Synchrotron Radiation*); however, if you intend to submit to *Acta Crystallographica Section C* or *E* or *IUCrData*, you should make sure that full publication checks are run on the final version of your CIF prior to submission.

Publication of your CIF in other journals

Please refer to the *Notes for Authors* of the relevant journal for any special instructions relating to CIF submission.

PLATON version of 03/06/2021; check.def file version of 02/06/2021

Datablock imineI_190K - ellipsoid plot

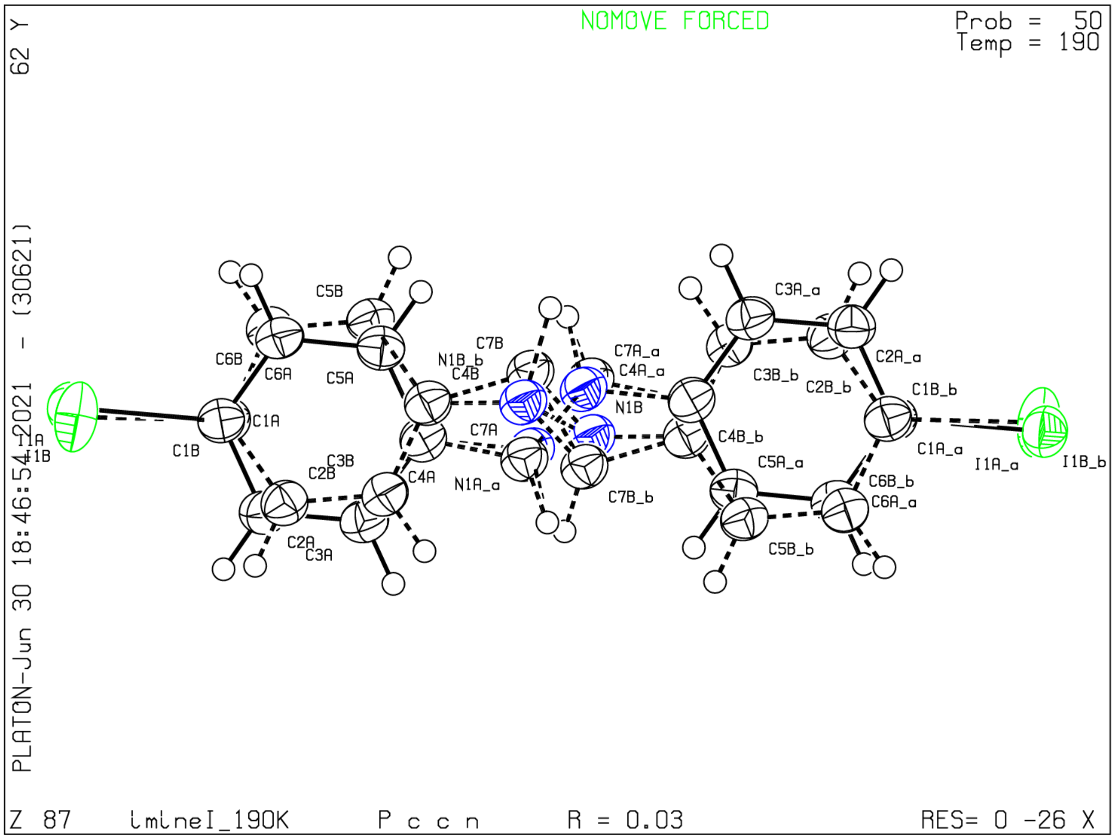

Datablock imineI_210K - ellipsoid plot

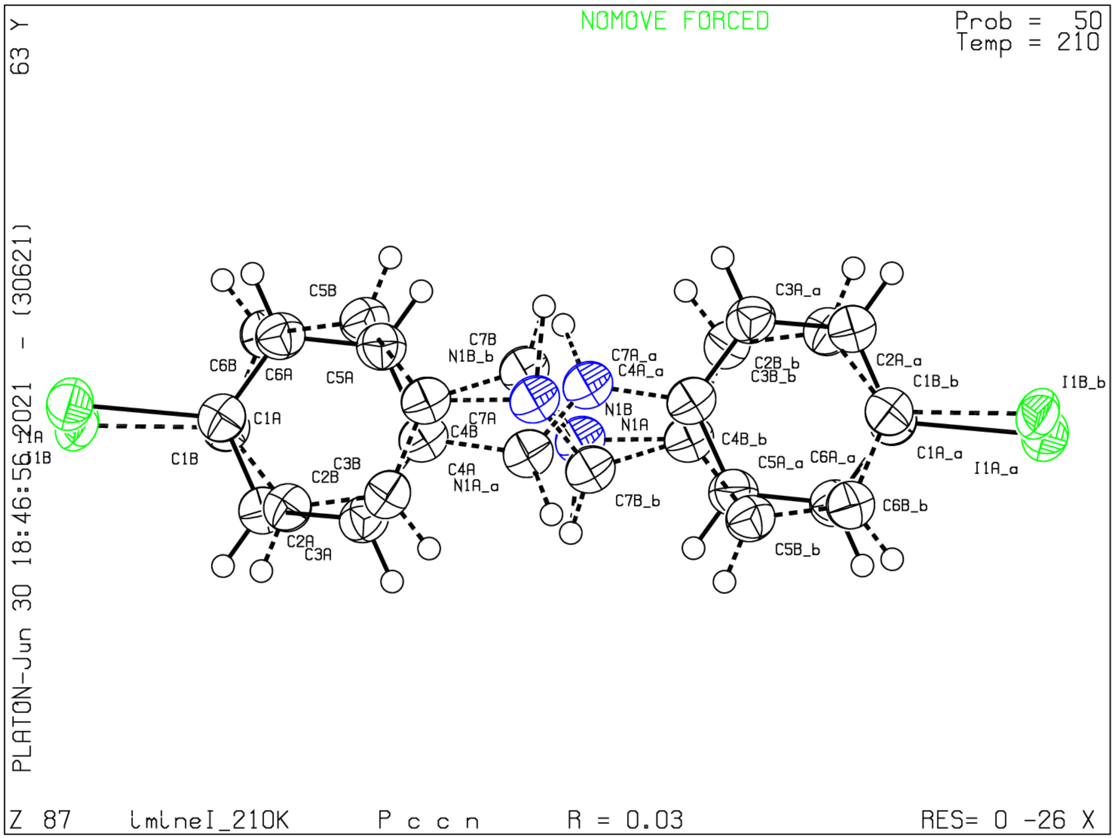

Datablock imineI_230K - ellipsoid plot

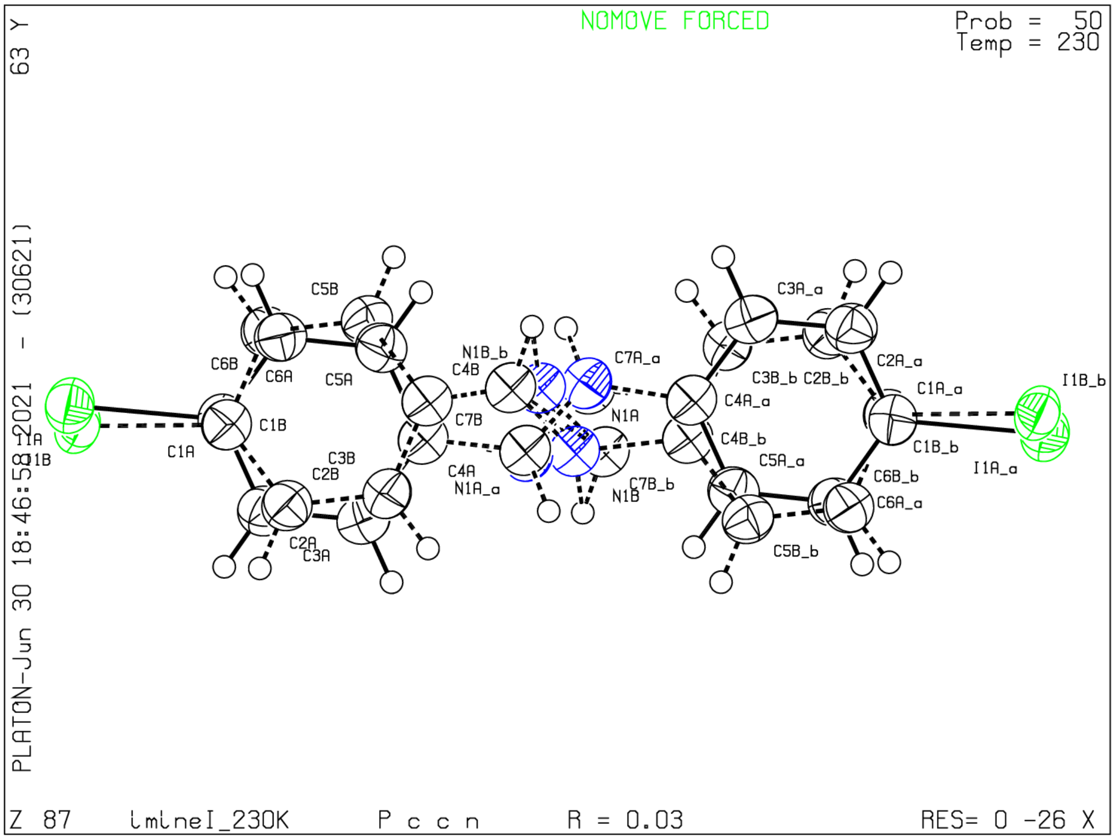

Datablock imineI_250K - ellipsoid plot

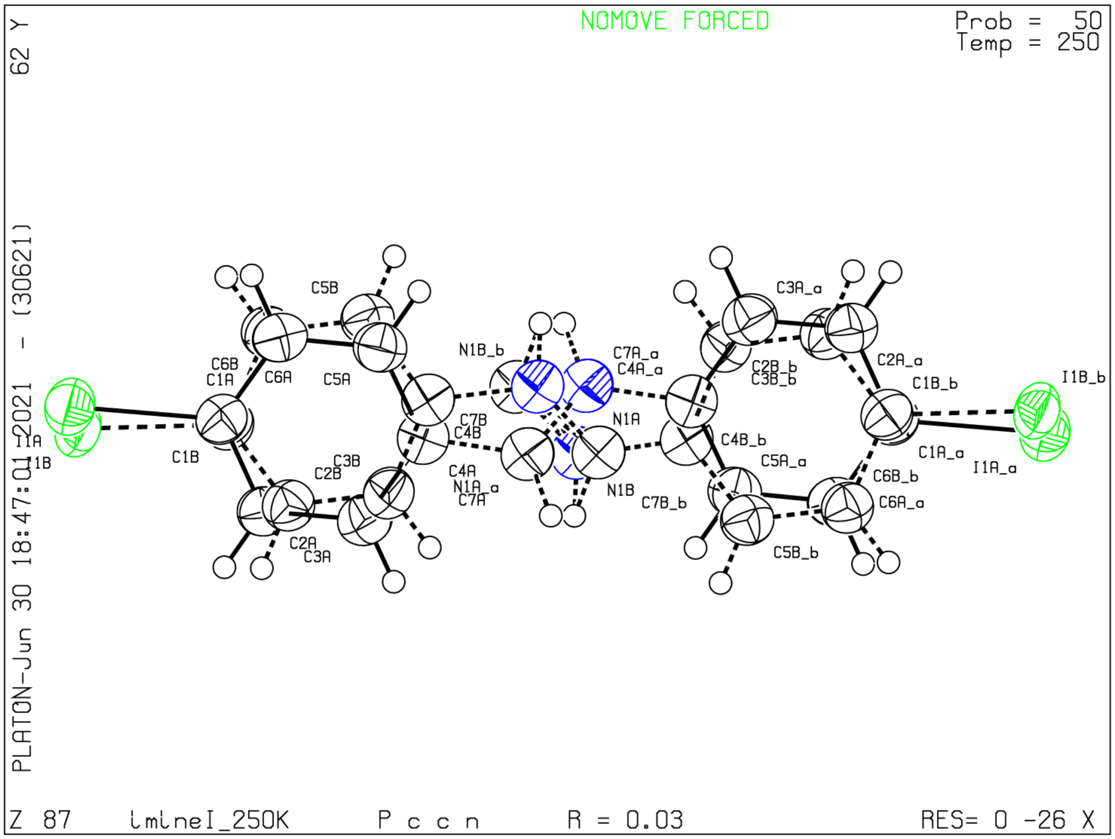

Datablock imineI_270K - ellipsoid plot

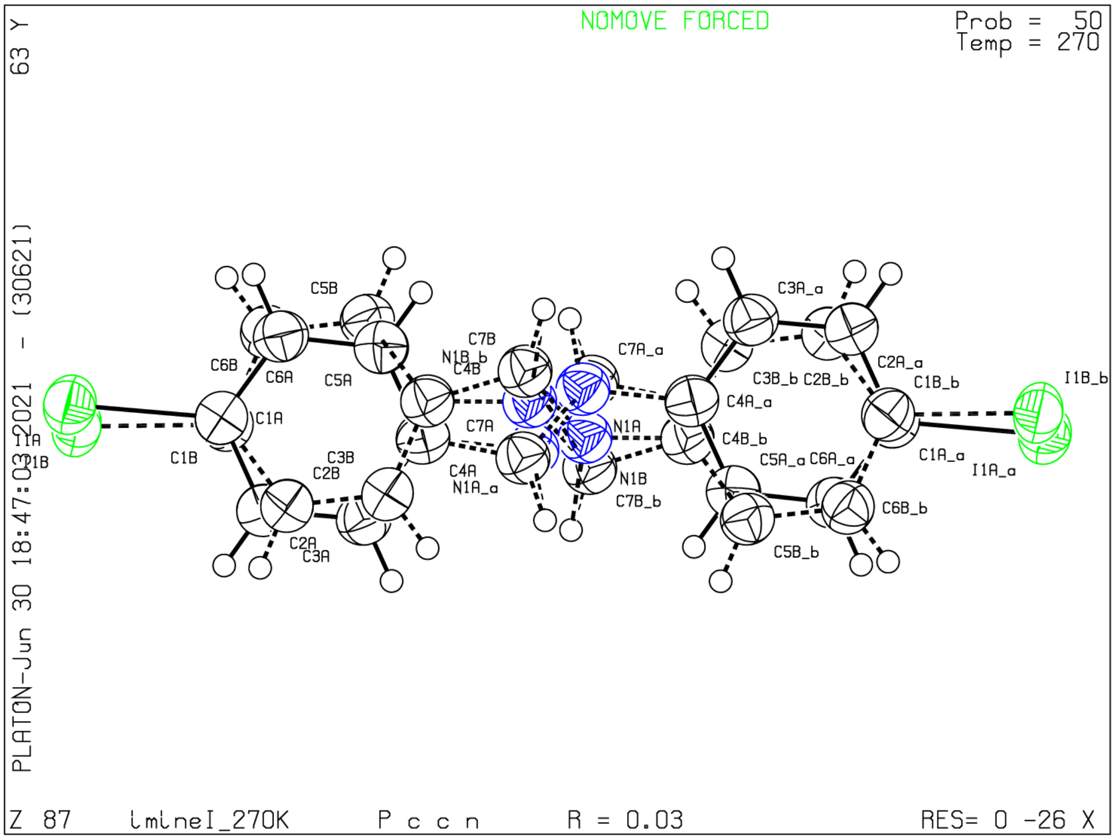

Datablock imineI_290K - ellipsoid plot

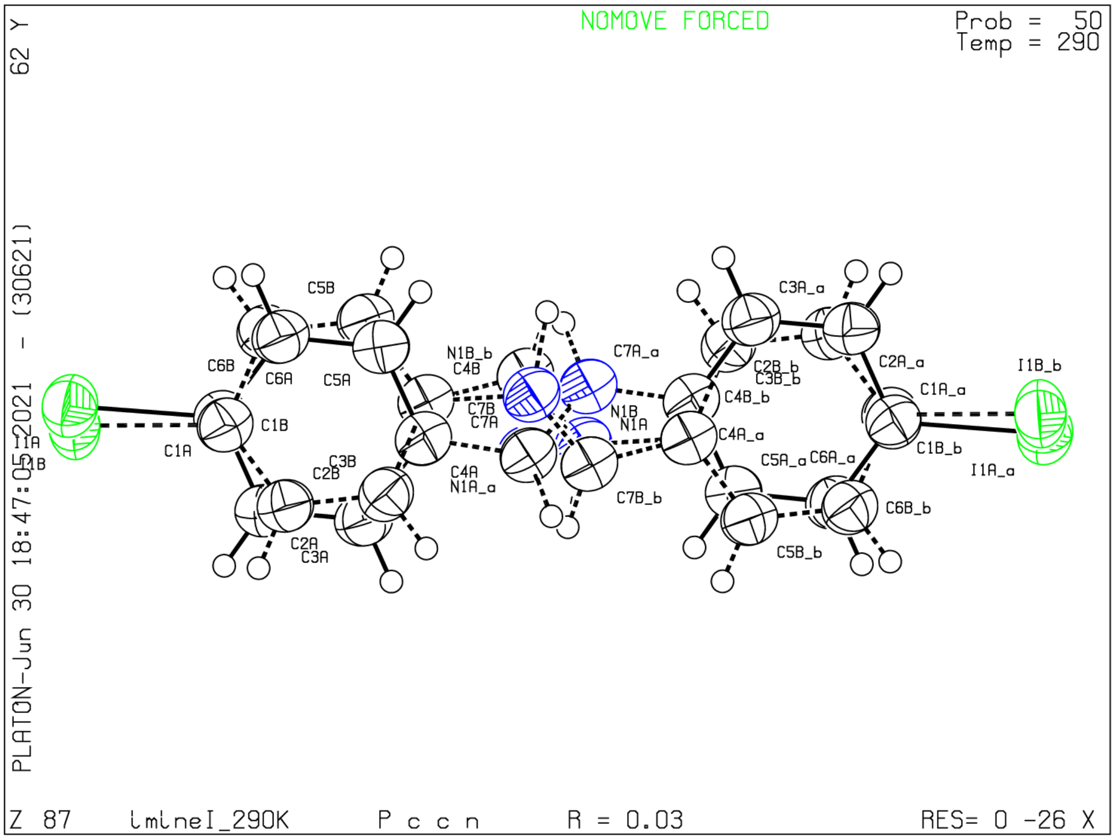

Datablock imineBr_190K - ellipsoid plot

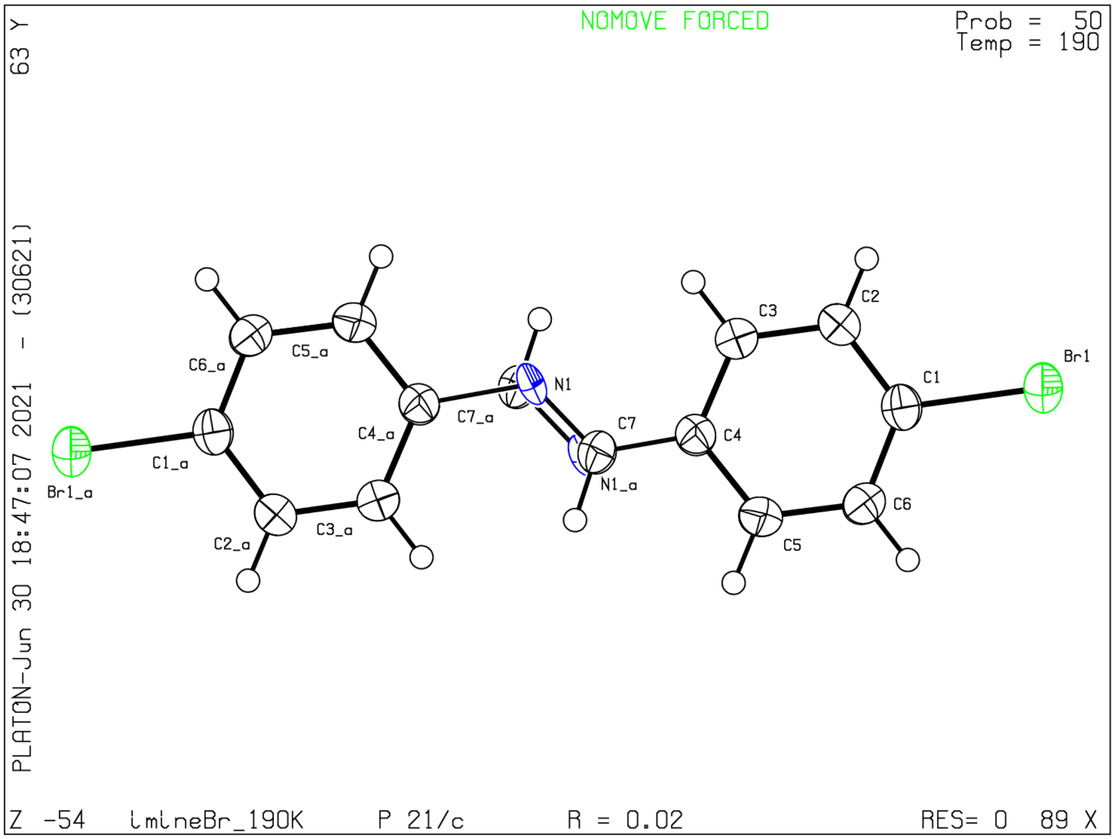

Datablock imineBr_210K - ellipsoid plot

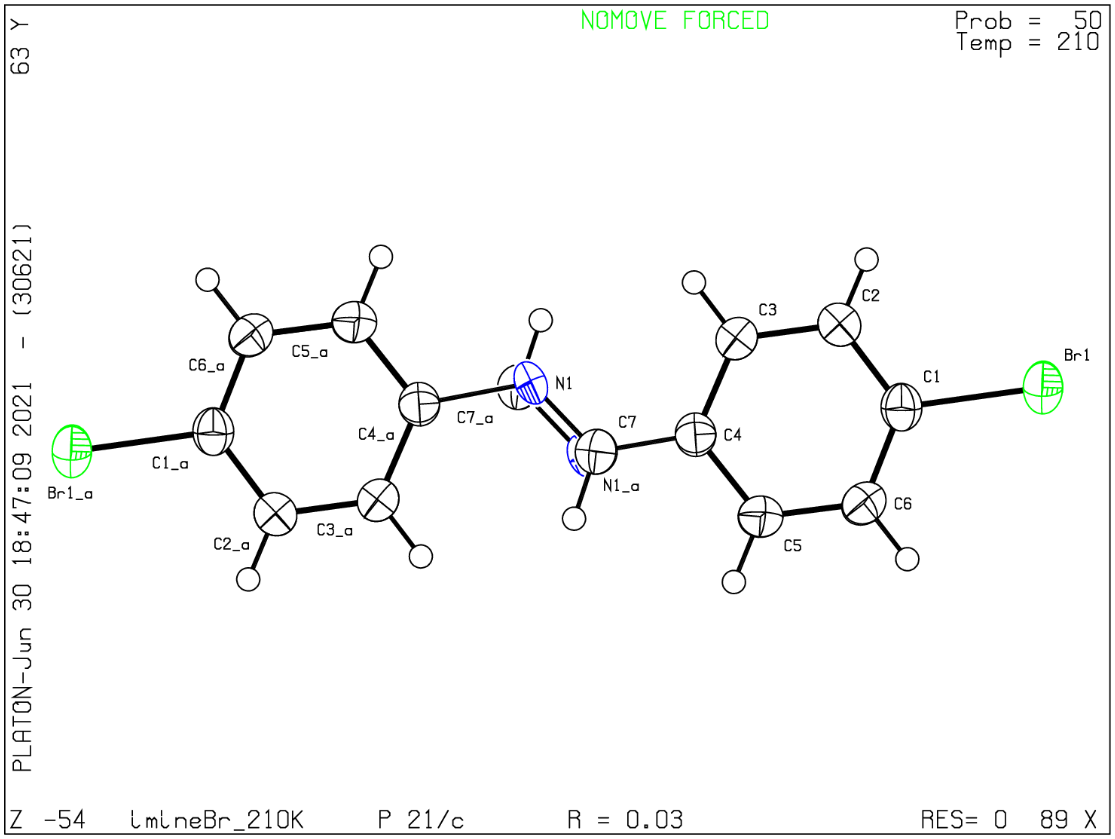

Datablock imineBr_230K - ellipsoid plot

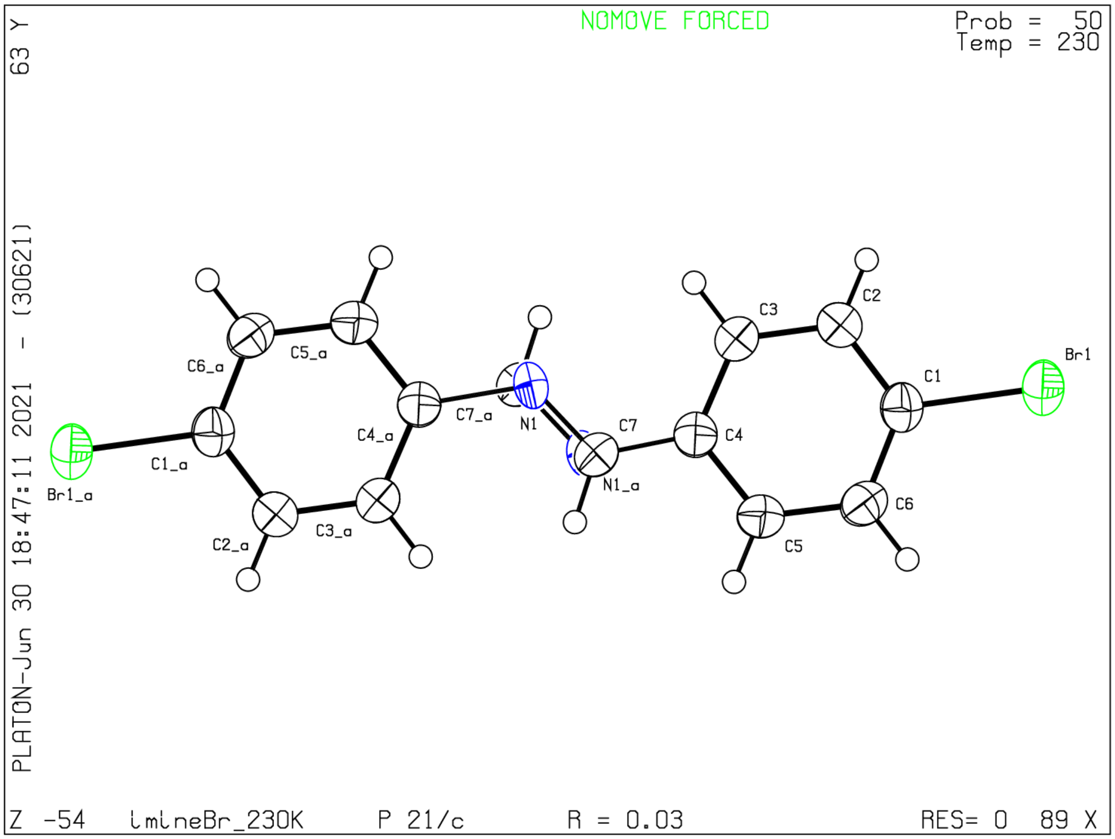

Datablock imineBr_250K - ellipsoid plot

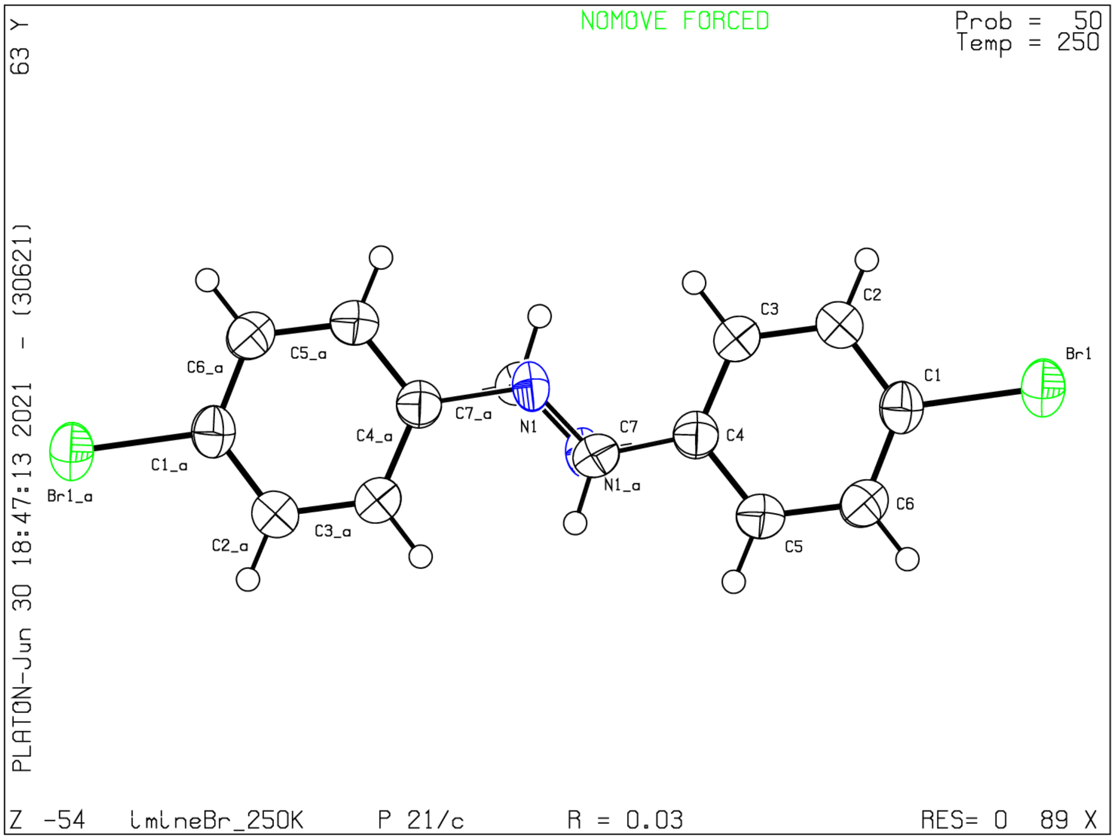

Datablock imineBr_270K - ellipsoid plot

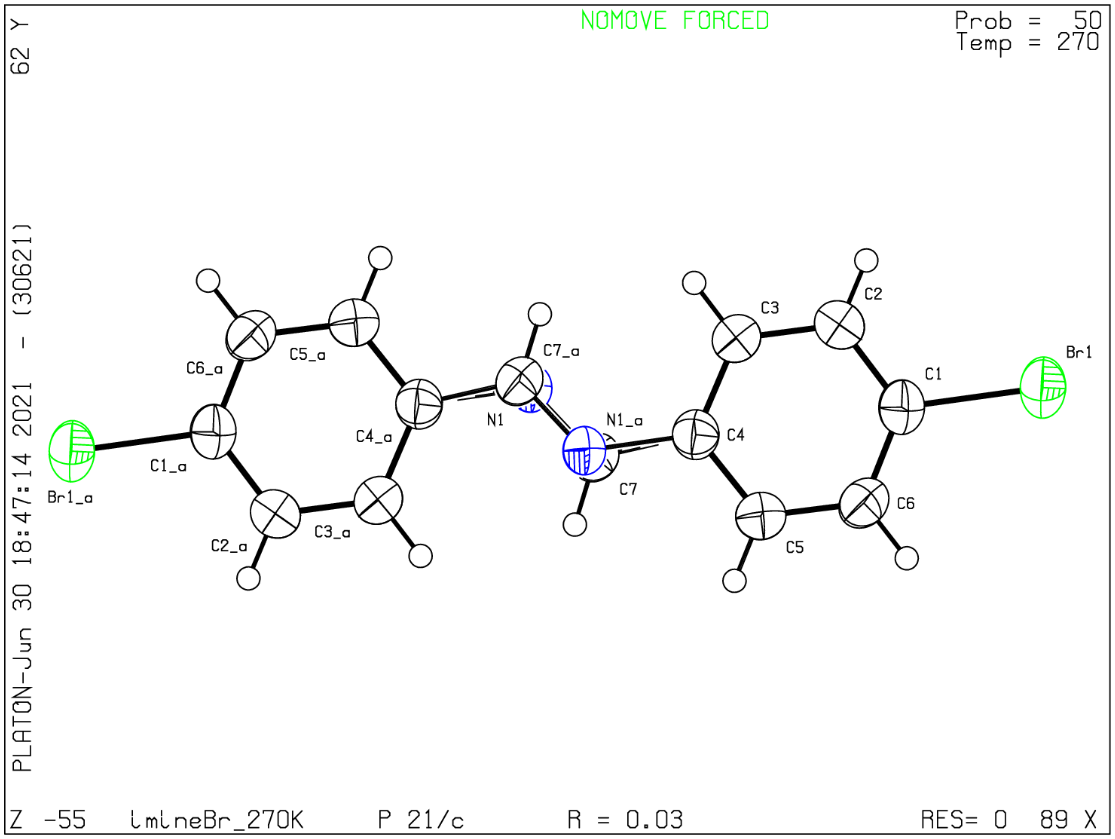

Datablock imineBr_290K - ellipsoid plot

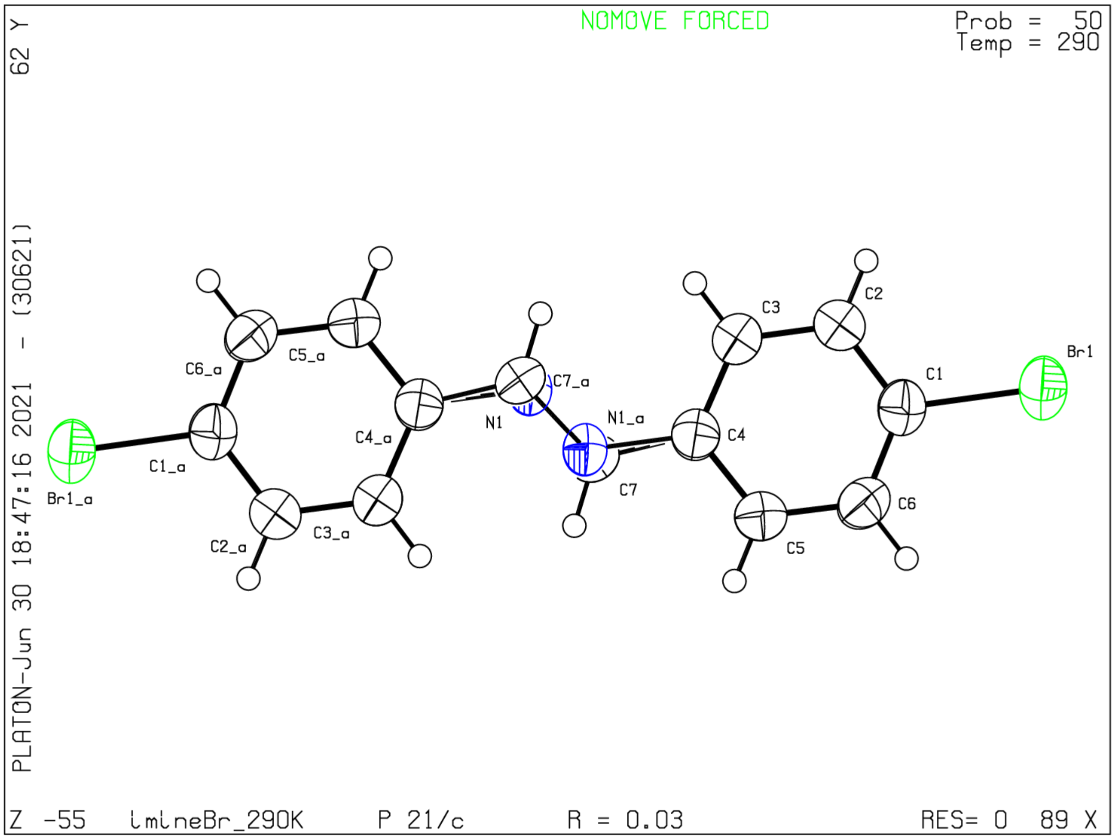

Supplement: Supplementary file 3 [file imines.pdf › imines.pdf]
