# Supplementary material for: Differences in thermal expansion and motion ability for herringbone and face-to-face π-stacked solids
Source: IUCrJ. 2021 Nov 3;9(Pt 1):31–42. doi: 10.1107/S2052252521009593 (PMC8733877; doi:10.1107/S2052252521009593)

checkCIF/PLATON report

Structure factors have been supplied for datablock(s) olefinBr_190, olefinBr_210, olefinBr_230, olefinBr_250, olefinBr_270, olefinBr_290, olefinI_190, olefinI_210, olefinI_230, olefinI_250, olefinI_270

THIS REPORT IS FOR GUIDANCE ONLY. IF USED AS PART OF A REVIEW PROCEDURE FOR PUBLICATION, IT SHOULD NOT REPLACE THE EXPERTISE OF AN EXPERIENCED CRYSTALLOGRAPHIC REFEREE.

No syntax errors found. CIF dictionary Interpreting this report

Datablock: olefinI_190

```
Bond precision:  C-C = 0.0070 A          Wavelength=1.54178

Cell:            a=7.3723(2)     b=28.9496(8)     c=5.98149(14)
                 alpha=90        beta=90          gamma=90
Temperature:     190 K

                Calculated                  Reported
Volume          1276.60(6)                  1276.60(6)
Space group     P c c n                     P c c n
Hall group      -P 2ab 2ac                  -P 2ab 2ac
Moiety formula  C14 H10 I2                  ?
Sum formula     C14 H10 I2                  C14 H10 I2
Mr              432.02                      432.02
Dx,g cm-3       2.248                       2.248
Z               4                           4
Mu (mm-1)       38.435                      38.435
F000            800.0                       800.0
F000'           800.27
h,k,lmax        9,36,7                      9,36,7
Nref            1354                        1330
Tmin,Tmax                                   0.014,0.092
Tmin'

Correction method= # Reported T Limits: Tmin=0.014 Tmax=0.092
AbsCorr = SPHERE

Data completeness= 0.982          Theta(max)= 77.390

R(reflections)= 0.0534( 1234)     wR2(reflections)= 0.1294( 1330)

S = 1.143                   Npar= 73
```

The following ALERTS were generated. Each ALERT has the format
test-name_ALERT_alert-type_alert-level.
Click on the hyperlinks for more details of the test.

Alert level B

PLAT094_ALERT_2_B Ratio of Maximum / Minimum Residual Density .... 4.22 Report

Author Response: Due to spurious electron density around the iodine atom that wasn't modeled.

Alert level C

PLAT971_ALERT_2_C Check Calcd Resid. Dens. 0.95A From I1 2.15 eA-3

Author Response: Due to spurious electron density around the iodine atom that wasn't modeled.

PLAT971_ALERT_2_C Check Calcd Resid. Dens. 0.78A From I1 2.01 eA-3

Author Response: Due to spurious electron density around the iodine atom that wasn't modeled.

PLAT971_ALERT_2_C Check Calcd Resid. Dens. 0.88A From I1 1.98 eA-3

Author Response: Due to spurious electron density around the iodine atom that wasn't modeled.

PLAT971_ALERT_2_C Check Calcd Resid. Dens. 0.73A From I1 1.83 eA-3

Author Response: Due to spurious electron density around the iodine atom that wasn't modeled.

Alert level G

PLAT912_ALERT_4_G Missing # of FCF Reflections Above STh/L= 0.600 25 Note
PLAT978_ALERT_2_G Number C-C Bonds with Positive Residual Density. 5 Info

---

0 **ALERT level A** = Most likely a serious problem - resolve or explain
1 **ALERT level B** = A potentially serious problem, consider carefully
4 **ALERT level C** = Check. Ensure it is not caused by an omission or oversight
2 **ALERT level G** = General information/check it is not something unexpected

0 ALERT type 1 CIF construction/syntax error, inconsistent or missing data
6 ALERT type 2 Indicator that the structure model may be wrong or deficient
0 ALERT type 3 Indicator that the structure quality may be low
1 ALERT type 4 Improvement, methodology, query or suggestion
0 ALERT type 5 Informative message, check

Datablock: olefinI_210

Bond precision: C-C = 0.0064 A Wavelength=1.54178

Cell:	a=7.39255(12)	b=28.9478(4)	c=5.99085(9)
	alpha=90	beta=90	gamma=90
Temperature:	210 K		

	Calculated	Reported
Volume	1282.03(3)	1282.03(3)
Space group	P c c n	P c c n
Hall group	-P 2ab 2ac	-P 2ab 2ac
Moiety formula	C14 H10 I2	?
Sum formula	C14 H10 I2	C14 H10 I2
Mr	432.02	432.02
Dx,g cm-3	2.238	2.238
Z	4	4
Mu (mm-1)	38.272	38.272
F000	800.0	800.0
F000’	800.27	
h,k,lmax	9,36,7	9,36,7
Nref	1360	1341
Tmin,Tmax		0.014,0.091
Tmin’		

Correction method= # Reported T Limits: Tmin=0.014 Tmax=0.091
AbsCorr = SPHERE

Data completeness= 0.986 Theta(max)= 77.389

R(reflections)= 0.0524(1228) wR2(reflections)= 0.1241(1341)

S = 1.154 Npar= 73

The following ALERTS were generated. Each ALERT has the format
test-name_ALERT_alert-type_alert-level.
Click on the hyperlinks for more details of the test.

Alert level B

PLAT094_ALERT_2_B Ratio of Maximum / Minimum Residual Density .... 4.60 Report

Author Response: Due to spurious electron density around the iodine atom that wasn’t modeled.

Alert level C

PLAT906_ALERT_3_C Large K Value in the Analysis of Variance ...... 2.072 Check

Author Response: A result of diffuse scattering around the Bragg peaks.

PLAT971_ALERT_2_C Check Calcd Resid. Dens. 0.93A From I1 2.29 eA-3

Author Response: Due to spurious electron density around the iodine atom that wasn't modeled.

PLAT971_ALERT_2_C Check Calcd Resid. Dens. 0.88A From I1 1.95 eA-3

Author Response: Due to spurious electron density around the iodine atom that wasn't modeled.

PLAT971_ALERT_2_C Check Calcd Resid. Dens. 0.79A From I1 1.85 eA-3

Author Response: Due to spurious electron density around the iodine atom that wasn't modeled.

PLAT971_ALERT_2_C Check Calcd Resid. Dens. 0.75A From I1 1.62 eA-3

Author Response: Due to spurious electron density around the iodine atom that wasn't modeled.

Alert level G

PLAT912_ALERT_4_G Missing # of FCF Reflections Above STh/L= 0.600 18 Note
PLAT978_ALERT_2_G Number C-C Bonds with Positive Residual Density. 6 Info

0 **ALERT level A** = Most likely a serious problem - resolve or explain
1 **ALERT level B** = A potentially serious problem, consider carefully
5 **ALERT level C** = Check. Ensure it is not caused by an omission or oversight
2 **ALERT level G** = General information/check it is not something unexpected

0 ALERT type 1 CIF construction/syntax error, inconsistent or missing data
6 ALERT type 2 Indicator that the structure model may be wrong or deficient
1 ALERT type 3 Indicator that the structure quality may be low
1 ALERT type 4 Improvement, methodology, query or suggestion
0 ALERT type 5 Informative message, check

Datablock: olefinI_230

Bond precision: C-C = 0.0051 A Wavelength=1.54178

Cell:	a=7.40898(16)	b=28.9461(6)	c=6.00040(12)
	alpha=90	beta=90	gamma=90
Temperature:	230 K		

	Calculated	Reported
Volume	1286.85(5)	1286.85(4)
Space group	P c c n	P c c n
Hall group	-P 2ab 2ac	-P 2ab 2ac
Moiety formula	C14 H10 I2	?
Sum formula	C14 H10 I2	C14 H10 I2
Mr	432.02	432.02
Dx,g cm-3	2.230	2.230
Z	4	4
Mu (mm-1)	38.129	38.129
F000	800.0	800.0
F000’	800.27	
h,k,lmax	9,36,7	9,36,7
Nref	1362	1351
Tmin,Tmax		0.260,0.342
Tmin’		

Correction method= # Reported T Limits: Tmin=0.260 Tmax=0.342
AbsCorr = SPHERE

Data completeness= 0.992 Theta(max)= 77.166

R(reflections)= 0.0281(1225) wR2(reflections)= 0.0773(1351)

S = 1.074 Npar= 73

The following ALERTS were generated. Each ALERT has the format
test-name_ALERT_alert-type_alert-level.
Click on the hyperlinks for more details of the test.

Alert level G

PLAT910_ALERT_3_G Missing # of FCF Reflection(s) Below Theta(Min). 1 Note
PLAT912_ALERT_4_G Missing # of FCF Reflections Above STh/L= 0.600 11 Note
PLAT978_ALERT_2_G Number C-C Bonds with Positive Residual Density. 0 Info

Author Response: The residual electron density was located mostly around the I atom.

```
   0 ALERT level A = Most likely a serious problem - resolve or explain
   0 ALERT level B = A potentially serious problem, consider carefully
   0 ALERT level C = Check. Ensure it is not caused by an omission or oversight
   3 ALERT level G = General information/check it is not something unexpected

   0 ALERT type 1 CIF construction/syntax error, inconsistent or missing data
   1 ALERT type 2 Indicator that the structure model may be wrong or deficient
   1 ALERT type 3 Indicator that the structure quality may be low
   1 ALERT type 4 Improvement, methodology, query or suggestion
   0 ALERT type 5 Informative message, check
```

Datablock: olefinI_250

```
Bond precision:  C-C = 0.0070 A          Wavelength=1.54178

Cell:            a=7.4287(3)     b=28.9400(9)     c=6.00927(19)
                 alpha=90        beta=90          gamma=90
Temperature:     250 K

                Calculated                 Reported
Volume          1291.91(8)                 1291.91(7)
Space group     P c c n                    P c c n
Hall group      -P 2ab 2ac                 -P 2ab 2ac
Moiety formula  C14 H10 I2                 ?
Sum formula     C14 H10 I2                 C14 H10 I2
Mr              432.02                     432.02
Dx,g cm-3       2.221                      2.221
Z               4                          4
Mu (mm-1)       37.979                     37.979
F000            800.0                      800.0
F000'           800.27
h,k,lmax        9,36,7                     9,36,7
Nref            1372                       1360
Tmin,Tmax                                  0.261,0.344
Tmin'

Correction method= # Reported T Limits: Tmin=0.261 Tmax=0.344
AbsCorr = SPHERE

Data completeness= 0.991          Theta(max)= 77.455

R(reflections)= 0.0361( 1189)     wR2(reflections)= 0.1064( 1360)

S = 1.078                   Npar= 73
```

The following ALERTS were generated. Each ALERT has the format
test-name_ALERT_alert-type_alert-level.
Click on the hyperlinks for more details of the test.

Alert level G

PLAT912_ALERT_4_G Missing # of FCF Reflections Above STh/L= 0.600 12 Note
PLAT978_ALERT_2_G Number C-C Bonds with Positive Residual Density. 0 Info

Author Response: The residual electron density was located mostly around the I atom.

---

0 **ALERT level A** = Most likely a serious problem - resolve or explain
0 **ALERT level B** = A potentially serious problem, consider carefully
0 **ALERT level C** = Check. Ensure it is not caused by an omission or oversight
2 **ALERT level G** = General information/check it is not something unexpected

0 ALERT type 1 CIF construction/syntax error, inconsistent or missing data
1 ALERT type 2 Indicator that the structure model may be wrong or deficient
0 ALERT type 3 Indicator that the structure quality may be low
1 ALERT type 4 Improvement, methodology, query or suggestion
0 ALERT type 5 Informative message, check

Datablock: olefinI_270

Bond precision: C-C = 0.0088 A Wavelength=1.54178

Cell:	a=7.4485(3)	b=28.9467(11)	c=6.0127(3)
	alpha=90	beta=90	gamma=90
Temperature:	270 K		

	Calculated	Reported
Volume	1296.40(10)	1296.39(9)
Space group	P c c n	P c c n
Hall group	-P 2ab 2ac	-P 2ab 2ac
Moiety formula	C14 H10 I2	?
Sum formula	C14 H10 I2	C14 H10 I2
Mr	432.02	432.02
Dx,g cm-3	2.214	2.213
Z	4	4
Mu (mm-1)	37.848	37.848
F000	800.0	800.0
F000’	800.27	
h,k,lmax	9,36,7	9,36,7
Nref	1381	1367
Tmin,Tmax		0.262,0.344
Tmin’		

Correction method= # Reported T Limits: Tmin=0.262 Tmax=0.344
AbsCorr = SPHERE

Data completeness= 0.990 Theta(max)= 77.749

R(reflections)= 0.0414(1140) wR2(reflections)= 0.1338(1367)

S = 1.105 Npar= 74

The following ALERTS were generated. Each ALERT has the format
test-name_ALERT_alert-type_alert-level.
Click on the hyperlinks for more details of the test.

Alert level C

```
PLAT230_ALERT_2_C Hirshfeld Test Diff for    I1       --C4       .        5.3 s.u.
PLAT342_ALERT_3_C Low Bond Precision on  C-C Bonds ..............    0.00875 Ang.
```

Author Response: The residual electron density was located mostly around the I atom.

Alert level G

```
PLAT883_ALERT_1_G No Info/Value for _atom_sites_solution_primary .     Please Do !
PLAT912_ALERT_4_G Missing # of FCF Reflections Above STh/L=  0.600         13 Note
PLAT954_ALERT_1_G Reported (CIF) and Actual (FCF) Kmax Differ by .          1 Units
PLAT978_ALERT_2_G Number C-C Bonds with Positive Residual Density.          2 Info
```

---

0 **ALERT level A** = Most likely a serious problem - resolve or explain
0 **ALERT level B** = A potentially serious problem, consider carefully
2 **ALERT level C** = Check. Ensure it is not caused by an omission or oversight
4 **ALERT level G** = General information/check it is not something unexpected

2 ALERT type 1 CIF construction/syntax error, inconsistent or missing data
2 ALERT type 2 Indicator that the structure model may be wrong or deficient
1 ALERT type 3 Indicator that the structure quality may be low
1 ALERT type 4 Improvement, methodology, query or suggestion
0 ALERT type 5 Informative message, check

Datablock: olefinBr_190

Bond precision: C-C = 0.0081 A Wavelength=1.54178

Cell:	a=7.3293(2)	b=27.6552(12)	c=5.9082(2)
	alpha=90	beta=90	gamma=90
Temperature:	190 K		

	Calculated	Reported
Volume	1197.55(7)	1197.55(8)
Space group	P c c n	P c c n
Hall group	-P 2ab 2ac	-P 2ab 2ac
Moiety formula	C14 H10 Br2	?
Sum formula	C14 H10 Br2	C14 H10 Br2
Mr	338.02	338.04
Dx,g cm-3	1.875	1.875
Z	4	4
Mu (mm-1)	8.305	8.305
F000	656.0	656.0
F000’	651.59	
h,k,lmax	9,35,7	9,34,7
Nref	1279	1230
Tmin,Tmax	0.464,0.898	0.545,1.000
Tmin’	0.236	

Correction method= # Reported T Limits: Tmin=0.545 Tmax=1.000
AbsCorr = GAUSSIAN

Data completeness= 0.962 Theta(max)= 77.822

R(reflections)= 0.0505(1028) wR2(reflections)= 0.1538(1230)

S = 1.163 Npar= 73

The following ALERTS were generated. Each ALERT has the format
test-name_ALERT_alert-type_alert-level.
Click on the hyperlinks for more details of the test.

Alert level C

PLAT341_ALERT_3_C Low Bond Precision on C-C Bonds .... 0.00813 Ang.

Author Response: Due to weak high angle data.

PLAT906_ALERT_3_C Large K Value in the Analysis of Variance ...... 3.887 Check

Author Response: Due to diffuse scattering around the Bragg peaks.

PLAT911_ALERT_3_C Missing FCF Refl Between Thmin & STh/L= 0.600 4 Report

---

Alert level G

PLAT083_ALERT_2_G SHELXL Second Parameter in WGHT Unusually Large 7.04 Why ?
PLAT912_ALERT_4_G Missing # of FCF Reflections Above STh/L= 0.600 45 Note
PLAT933_ALERT_2_G Number of OMIT Records in Embedded .res File ... 5 Note
PLAT978_ALERT_2_G Number C-C Bonds with Positive Residual Density. 2 Info

---

0 **ALERT level A** = Most likely a serious problem - resolve or explain

0 **ALERT level B** = A potentially serious problem, consider carefully
3 **ALERT level C** = Check. Ensure it is not caused by an omission or oversight
4 **ALERT level G** = General information/check it is not something unexpected

0 ALERT type 1 CIF construction/syntax error, inconsistent or missing data
3 ALERT type 2 Indicator that the structure model may be wrong or deficient
3 ALERT type 3 Indicator that the structure quality may be low
1 ALERT type 4 Improvement, methodology, query or suggestion
0 ALERT type 5 Informative message, check

Datablock: olefinBr_210

Bond precision: C-C = 0.0035 A Wavelength=1.54178

Cell:	a=7.34625(11)	b=27.6646(4)	c=5.91922(8)
	alpha=90	beta=90	gamma=90
Temperature:	210 K		

	Calculated	Reported
Volume	1202.97(3)	1202.97(3)
Space group	P c c n	P c c n
Hall group	-P 2ab 2ac	-P 2ab 2ac
Moiety formula	C14 H10 Br2	?
Sum formula	C14 H10 Br2	C14 H10 Br2
Mr	338.02	338.04
Dx,g cm-3	1.866	1.866
Z	4	4
Mu (mm-1)	8.268	8.268
F000	656.0	656.0
F000’	651.59	
h,k,lmax	9,35,7	9,34,7
Nref	1277	1259
Tmin,Tmax	0.466,0.898	0.546,1.000
Tmin’	0.237	

Correction method= # Reported T Limits: Tmin=0.546 Tmax=1.000
AbsCorr = GAUSSIAN

Data completeness= 0.986 Theta(max)= 77.332

R(reflections)= 0.0287(1155) wR2(reflections)= 0.0744(1259)

S = 1.070 Npar= 73

The following ALERTS were generated. Each ALERT has the format
test-name_ALERT_alert-type_alert-level.
Click on the hyperlinks for more details of the test.

Alert level C

```
PLAT906_ALERT_3_C Large K Value in the Analysis of Variance ......      2.328 Check
```

Author Response: Due to diffuse scattering around the Bragg peaks.

Alert level G

```
PLAT912_ALERT_4_G Missing # of FCF Reflections Above STh/L=  0.600         17 Note
PLAT933_ALERT_2_G Number of OMIT Records in Embedded .res File ...          5 Note
PLAT978_ALERT_2_G Number C-C Bonds with Positive Residual Density.          6 Info
```

```
   0 ALERT level A = Most likely a serious problem - resolve or explain
   0 ALERT level B = A potentially serious problem, consider carefully
   1 ALERT level C = Check. Ensure it is not caused by an omission or oversight
   3 ALERT level G = General information/check it is not something unexpected

   0 ALERT type 1 CIF construction/syntax error, inconsistent or missing data
   2 ALERT type 2 Indicator that the structure model may be wrong or deficient
   1 ALERT type 3 Indicator that the structure quality may be low
   1 ALERT type 4 Improvement, methodology, query or suggestion
   0 ALERT type 5 Informative message, check
```

Datablock: olefinBr_230

Bond precision: C-C = 0.0038 A   Wavelength=1.54178

Cell:	a=7.36051(14)	b=27.6702(6)	c=5.92784(11)
	alpha=90	beta=90	gamma=90
Temperature:	230 K		

	Calculated	Reported
Volume	1207.30(4)	1207.30(4)
Space group	P c c n	P c c n
Hall group	-P 2ab 2ac	-P 2ab 2ac
Moiety formula	C14 H10 Br2	?
Sum formula	C14 H10 Br2	C14 H10 Br2
Mr	338.02	338.04
Dx,g cm-3	1.860	1.860
Z	4	4
Mu (mm-1)	8.238	8.238
F000	656.0	656.0
F000’	651.59	
h,k,lmax	9,35,7	9,34,7
Nref	1282	1246
Tmin,Tmax	0.427,0.898	0.548,1.000
Tmin’	0.261	

Correction method= # Reported T Limits: Tmin=0.548 Tmax=1.000
AbsCorr = GAUSSIAN

Data completeness= 0.972 Theta(max)= 77.315

R(reflections)= 0.0273(1111) wR2(reflections)= 0.0768(1246)

S = 1.107 Npar= 73

The following ALERTS were generated. Each ALERT has the format
test-name_ALERT_alert-type_alert-level.
Click on the hyperlinks for more details of the test.

Alert level C

PLAT911_ALERT_3_C Missing FCF Refl Between Thmin & STh/L= 0.600 12 Report

---

Alert level G

PLAT912_ALERT_4_G Missing # of FCF Reflections Above STh/L= 0.600 25 Note
PLAT933_ALERT_2_G Number of OMIT Records in Embedded .res File ... 5 Note
PLAT978_ALERT_2_G Number C-C Bonds with Positive Residual Density. 6 Info

---

0 **ALERT level A** = Most likely a serious problem - resolve or explain
0 **ALERT level B** = A potentially serious problem, consider carefully
1 **ALERT level C** = Check. Ensure it is not caused by an omission or oversight
3 **ALERT level G** = General information/check it is not something unexpected

0 ALERT type 1 CIF construction/syntax error, inconsistent or missing data
2 ALERT type 2 Indicator that the structure model may be wrong or deficient
1 ALERT type 3 Indicator that the structure quality may be low
1 ALERT type 4 Improvement, methodology, query or suggestion
0 ALERT type 5 Informative message, check

Datablock: olefinBr_250

Bond precision: C-C = 0.0040 A Wavelength=1.54178

Cell:	a=7.37825(13)	b=27.6789(5)	c=5.93730(11)
	alpha=90	beta=90	gamma=90
Temperature:	250 K		

	Calculated	Reported
Volume	1212.53(4)	1212.53(4)
Space group	P c c n	P c c n
Hall group	-P 2ab 2ac	-P 2ab 2ac
Moiety formula	C14 H10 Br2	?
Sum formula	C14 H10 Br2	C14 H10 Br2
Mr	338.02	338.04
Dx,g cm-3	1.852	1.852
Z	4	4
Mu (mm-1)	8.203	8.203
F000	656.0	656.0
F000’	651.59	
h,k,lmax	9,35,7	9,34,7
Nref	1286	1252
Tmin,Tmax	0.429,0.899	0.544,1.000
Tmin’	0.263	

Correction method= # Reported T Limits: Tmin=0.544 Tmax=1.000
AbsCorr = GAUSSIAN

Data completeness= 0.974 Theta(max)= 77.373

R(reflections)= 0.0284(1100) wR2(reflections)= 0.0780(1252)

S = 1.049 Npar= 73

The following ALERTS were generated. Each ALERT has the format
test-name_ALERT_alert-type_alert-level.
Click on the hyperlinks for more details of the test.

Alert level C

PLAT911_ALERT_3_C Missing FCF Refl Between Thmin & STh/L= 0.600 13 Report

---

Alert level G

PLAT912_ALERT_4_G Missing # of FCF Reflections Above STh/L= 0.600 22 Note
PLAT933_ALERT_2_G Number of OMIT Records in Embedded .res File ... 5 Note
PLAT978_ALERT_2_G Number C-C Bonds with Positive Residual Density. 7 Info

---

0 **ALERT level A** = Most likely a serious problem - resolve or explain
0 **ALERT level B** = A potentially serious problem, consider carefully
1 **ALERT level C** = Check. Ensure it is not caused by an omission or oversight
3 **ALERT level G** = General information/check it is not something unexpected

0 ALERT type 1 CIF construction/syntax error, inconsistent or missing data
2 ALERT type 2 Indicator that the structure model may be wrong or deficient
1 ALERT type 3 Indicator that the structure quality may be low
1 ALERT type 4 Improvement, methodology, query or suggestion
0 ALERT type 5 Informative message, check

Datablock: olefinBr_270

Bond precision: C-C = 0.0040 A Wavelength=1.54178

Cell:	a=7.39602(15)	b=27.6823(6)	c=5.94943(12)
	alpha=90	beta=90	gamma=90
Temperature:	270 K		

	Calculated	Reported
Volume	1218.08(4)	1218.08(4)
Space group	P c c n	P c c n
Hall group	-P 2ab 2ac	-P 2ab 2ac
Moiety formula	C14 H10 Br2	?
Sum formula	C14 H10 Br2	C14 H10 Br2
Mr	338.02	338.04
Dx,g cm-3	1.843	1.843
Z	4	4
Mu (mm-1)	8.165	8.165
F000	656.0	656.0
F000’	651.59	
h,k,lmax	9,34,7	9,34,7
Nref	1288	1264
Tmin,Tmax	0.430,0.899	0.544,1.000
Tmin’	0.264	

Correction method= # Reported T Limits: Tmin=0.544 Tmax=1.000
AbsCorr = GAUSSIAN

Data completeness= 0.981 Theta(max)= 76.867

R(reflections)= 0.0305(1073) wR2(reflections)= 0.0793(1264)

S = 1.054 Npar= 73

The following ALERTS were generated. Each ALERT has the format
test-name_ALERT_alert-type_alert-level.
Click on the hyperlinks for more details of the test.

Alert level C

PLAT906_ALERT_3_C Large K Value in the Analysis of Variance ...... 3.149 Check

Author Response: Due to diffuse scattering around the Bragg peaks.

PLAT911_ALERT_3_C Missing FCF Refl Between Thmin & STh/L= 0.600 4 Report

---

Alert level G

```
PLAT912_ALERT_4_G Missing # of FCF Reflections Above STh/L=  0.600         20 Note
PLAT933_ALERT_2_G Number of OMIT Records in Embedded .res File ...          5 Note
PLAT978_ALERT_2_G Number C-C Bonds with Positive Residual Density.          5 Info
```

---

0 **ALERT level A** = Most likely a serious problem - resolve or explain
0 **ALERT level B** = A potentially serious problem, consider carefully
2 **ALERT level C** = Check. Ensure it is not caused by an omission or oversight
3 **ALERT level G** = General information/check it is not something unexpected

0 ALERT type 1 CIF construction/syntax error, inconsistent or missing data
2 ALERT type 2 Indicator that the structure model may be wrong or deficient
2 ALERT type 3 Indicator that the structure quality may be low
1 ALERT type 4 Improvement, methodology, query or suggestion
0 ALERT type 5 Informative message, check

Datablock: olefinBr_290

Bond precision: C-C = 0.0044 A   Wavelength=1.54178

Cell:	a=7.4125(2)	b=27.6845(8)	c=5.95711(19)
	alpha=90	beta=90	gamma=90
Temperature:	290 K		

	Calculated	Reported
Volume	1222.47(6)	1222.46(6)
Space group	P c c n	P c c n
Hall group	-P 2ab 2ac	-P 2ab 2ac
Moiety formula	C14 H10 Br2	?
Sum formula	C14 H10 Br2	C14 H10 Br2
Mr	338.02	338.04
Dx,g cm-3	1.837	1.837
Z	4	4
Mu (mm-1)	8.136	8.136
F000	656.0	656.0
F000’	651.59	
h,k,lmax	9,35,7	9,34,7
Nref	1294	1211
Tmin,Tmax	0.432,0.900	0.458,1.000
Tmin’	0.265	

Correction method= # Reported T Limits: Tmin=0.458 Tmax=1.000
AbsCorr = GAUSSIAN

Data completeness= 0.936   Theta(max)= 77.113

R(reflections)= 0.0352(992)   wR2(reflections)= 0.0992(1211)

```
S = 1.063                    Npar= 73
```

```
The following ALERTS were generated. Each ALERT has the format
       test-name_ALERT_alert-type_alert-level.
Click on the hyperlinks for more details of the test.
```

Alert level C

```
PLAT029_ALERT_3_C _diffrn_measured_fraction_theta_full value Low .      0.972 Why?
```

Author Response: Due to poor diffraction of high angle data.

```
PLAT911_ALERT_3_C Missing FCF Refl Between Thmin & STh/L=    0.600         31 Report
```

Alert level G

```
PLAT912_ALERT_4_G Missing # of FCF Reflections Above STh/L=  0.600         52 Note
PLAT933_ALERT_2_G Number of OMIT Records in Embedded .res File ...          5 Note
PLAT978_ALERT_2_G Number C-C Bonds with Positive Residual Density.          1 Info
```

```
   0 ALERT level A = Most likely a serious problem - resolve or explain
   0 ALERT level B = A potentially serious problem, consider carefully
   2 ALERT level C = Check. Ensure it is not caused by an omission or oversight
   3 ALERT level G = General information/check it is not something unexpected

   0 ALERT type 1 CIF construction/syntax error, inconsistent or missing data
   2 ALERT type 2 Indicator that the structure model may be wrong or deficient
   2 ALERT type 3 Indicator that the structure quality may be low
   1 ALERT type 4 Improvement, methodology, query or suggestion
   0 ALERT type 5 Informative message, check
```

Validation response form

Please find below a validation response form (VRF) that can be filled in and pasted into your CIF.

```
# start Validation Reply Form
_vrf_PLAT230_olefinI_270
;
PROBLEM: Hirshfeld Test Diff for    I1       --C4       .        5.3 s.u.
RESPONSE: ...
;
_vrf_PLAT911_olefinBr_190
;
PROBLEM: Missing FCF Refl Between Thmin & STh/L=    0.600          4 Report
RESPONSE: ...
;
_vrf_PLAT911_olefinBr_230
;
PROBLEM: Missing FCF Refl Between Thmin & STh/L=    0.600         12 Report
RESPONSE: ...
;
_vrf_PLAT911_olefinBr_250
;
PROBLEM: Missing FCF Refl Between Thmin & STh/L=    0.600         13 Report
RESPONSE: ...
;
```

```
_vrf_PLAT911_olefinBr_270
;
PROBLEM: Missing FCF Refl Between Thmin & STh/L=    0.600          4 Report
RESPONSE: ...
;
_vrf_PLAT911_olefinBr_290
;
PROBLEM: Missing FCF Refl Between Thmin & STh/L=    0.600         31 Report
RESPONSE: ...
;
# end Validation Reply Form
```

It is advisable to attempt to resolve as many as possible of the alerts in all categories. Often the minor alerts point to easily fixed oversights, errors and omissions in your CIF or refinement strategy, so attention to these fine details can be worthwhile. In order to resolve some of the more serious problems it may be necessary to carry out additional measurements or structure refinements. However, the purpose of your study may justify the reported deviations and the more serious of these should normally be commented upon in the discussion or experimental section of a paper or in the "special_details" fields of the CIF. checkCIF was carefully designed to identify outliers and unusual parameters, but every test has its limitations and alerts that are not important in a particular case may appear. Conversely, the absence of alerts does not guarantee there are no aspects of the results needing attention. It is up to the individual to critically assess their own results and, if necessary, seek expert advice.

Publication of your CIF in IUCr journals

A basic structural check has been run on your CIF. These basic checks will be run on all CIFs submitted for publication in IUCr journals (*Acta Crystallographica*, *Journal of Applied Crystallography*, *Journal of Synchrotron Radiation*); however, if you intend to submit to *Acta Crystallographica Section C* or *E* or *IUCrData*, you should make sure that full publication checks are run on the final version of your CIF prior to submission.

Publication of your CIF in other journals

Please refer to the *Notes for Authors* of the relevant journal for any special instructions relating to CIF submission.

PLATON version of 03/06/2021; check.def file version of 02/06/2021

Datablock olefinI_190 - ellipsoid plot

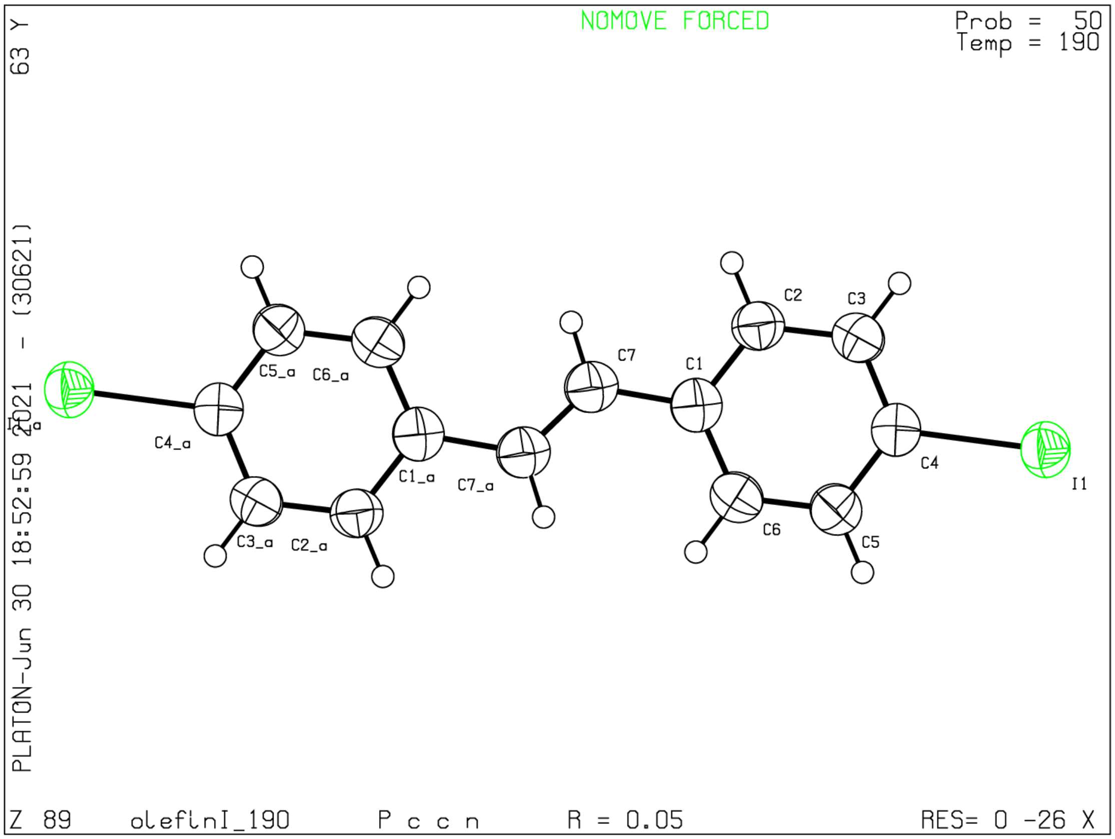

Datablock olefinI_210 - ellipsoid plot

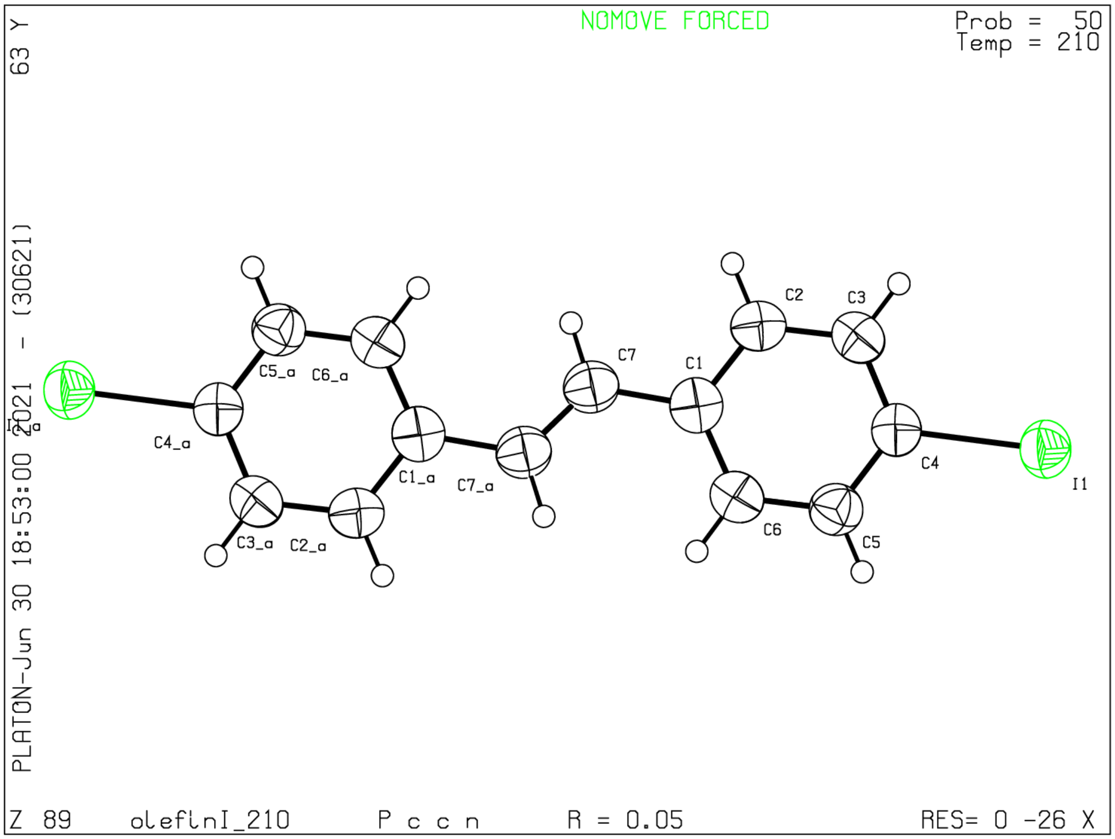

Datablock olefinI_230 - ellipsoid plot

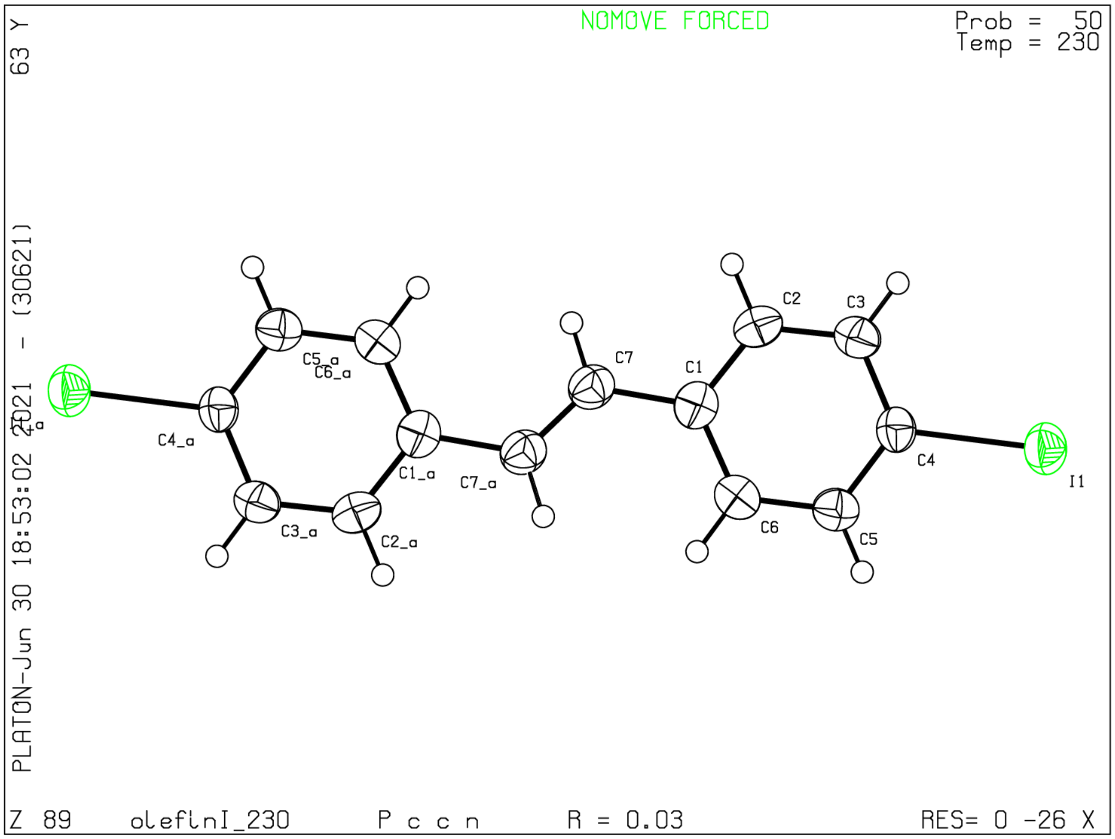

Datablock olefinI_250 - ellipsoid plot

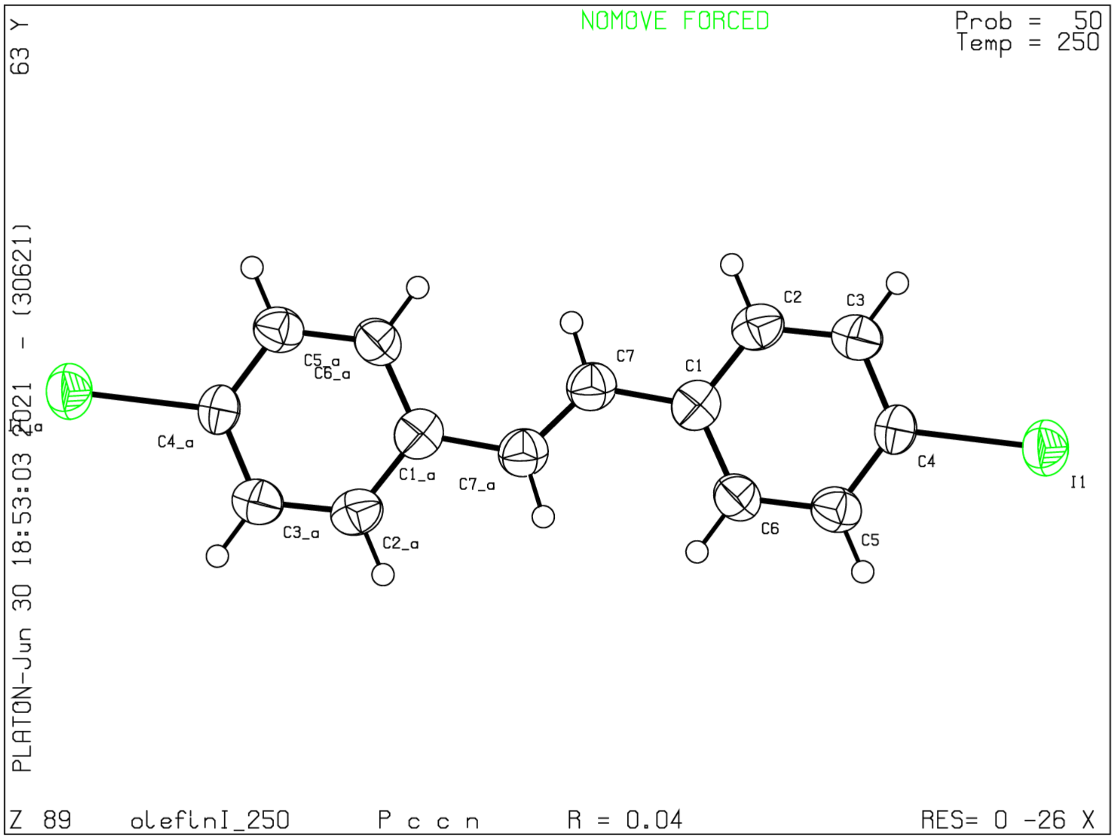

Datablock olefinI_270 - ellipsoid plot

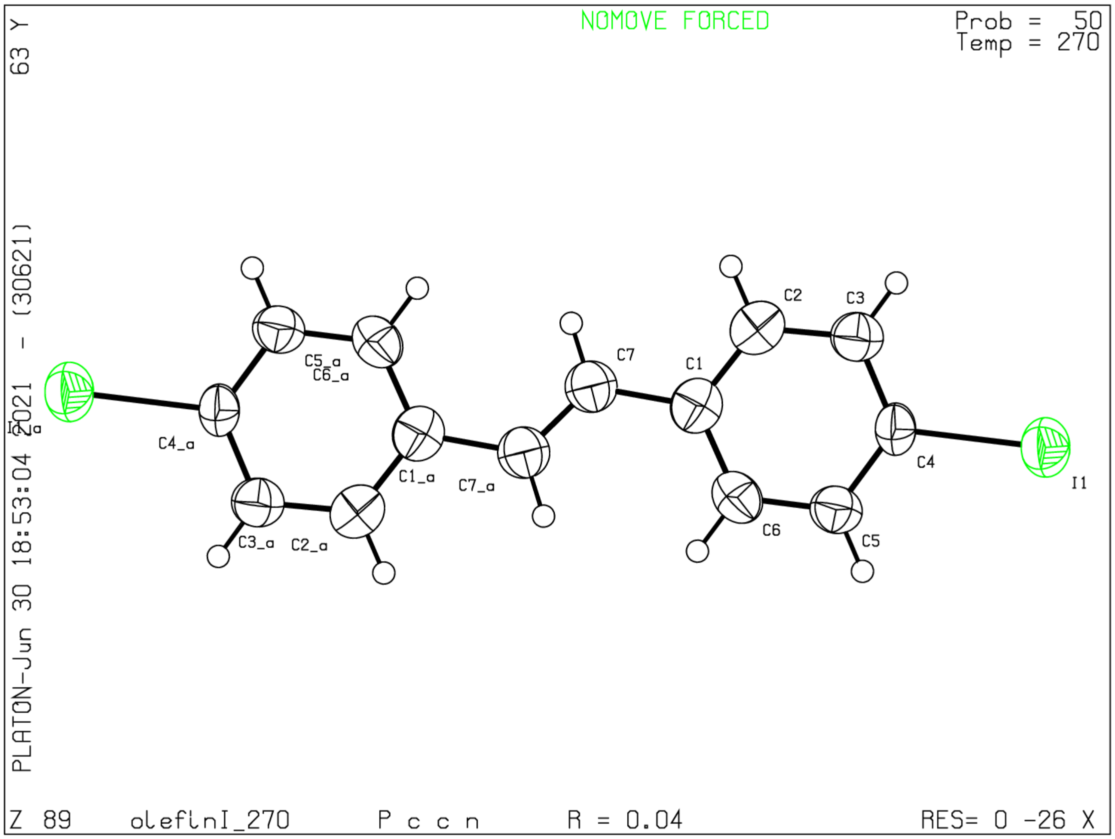

Datablock olefinBr_190 - ellipsoid plot

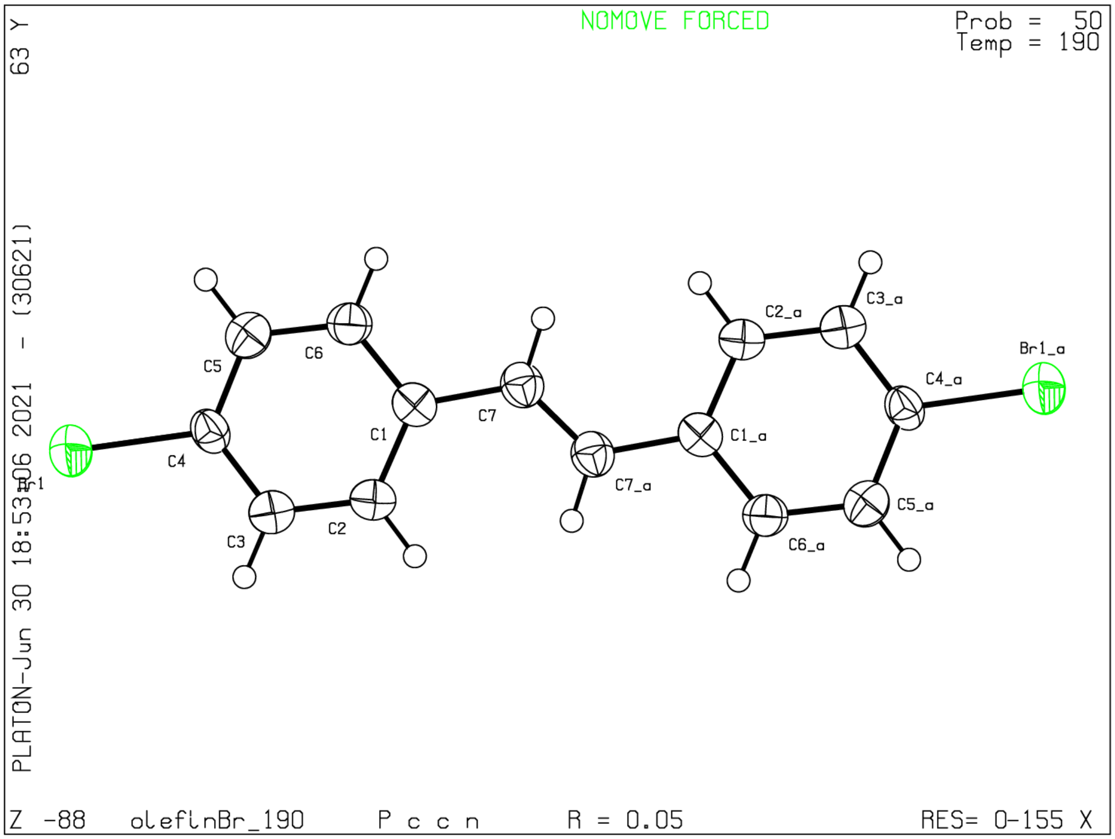

Datablock olefinBr_210 - ellipsoid plot

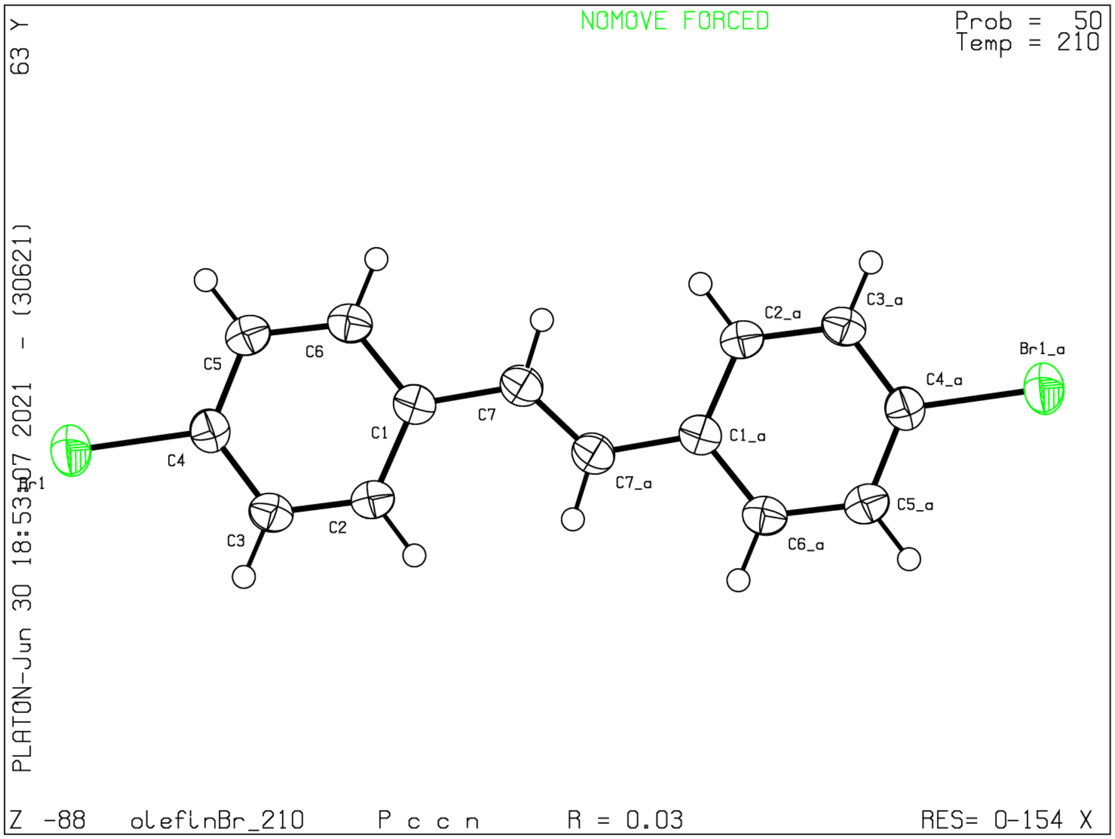

Datablock olefinBr_230 - ellipsoid plot

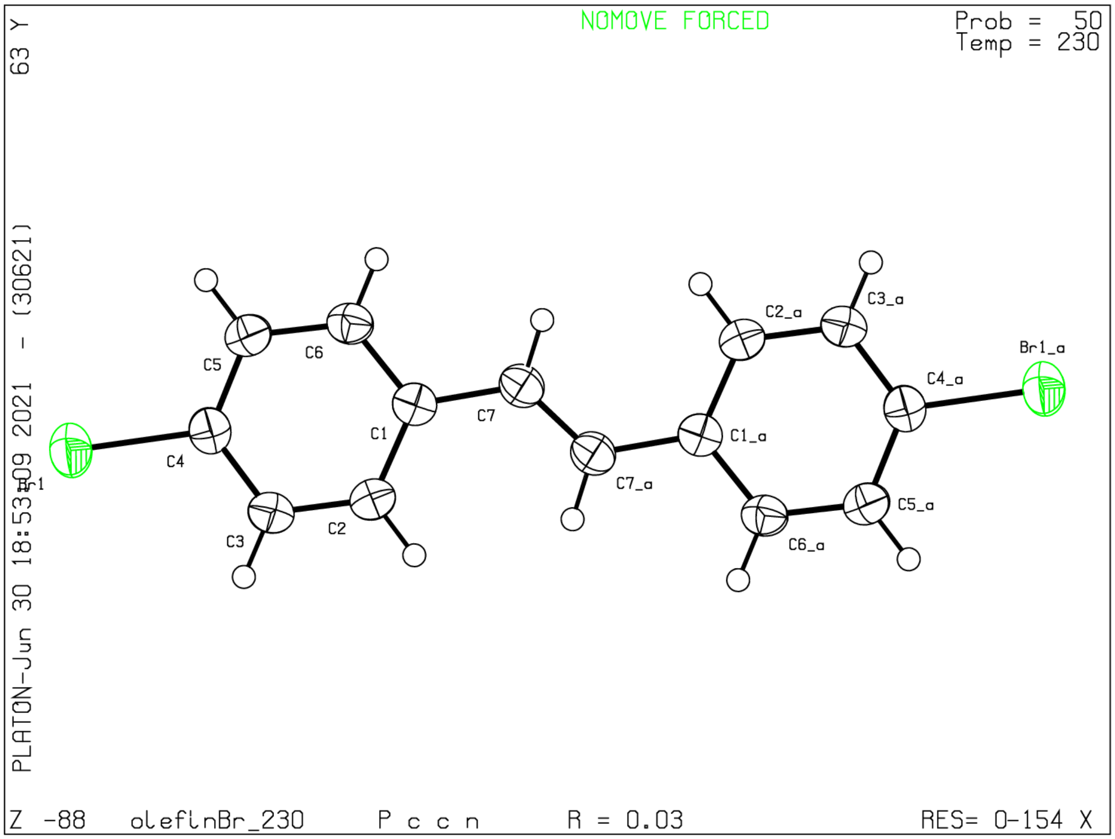

Datablock olefinBr_250 - ellipsoid plot

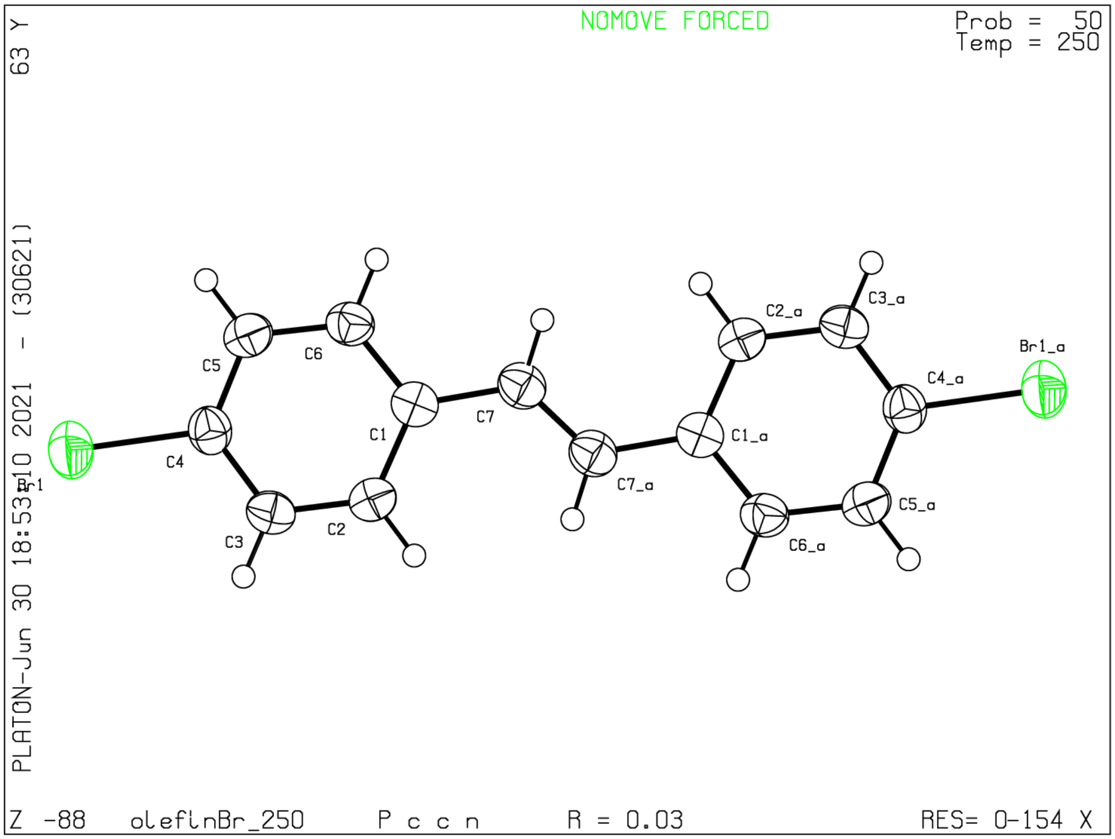

Datablock olefinBr_270 - ellipsoid plot

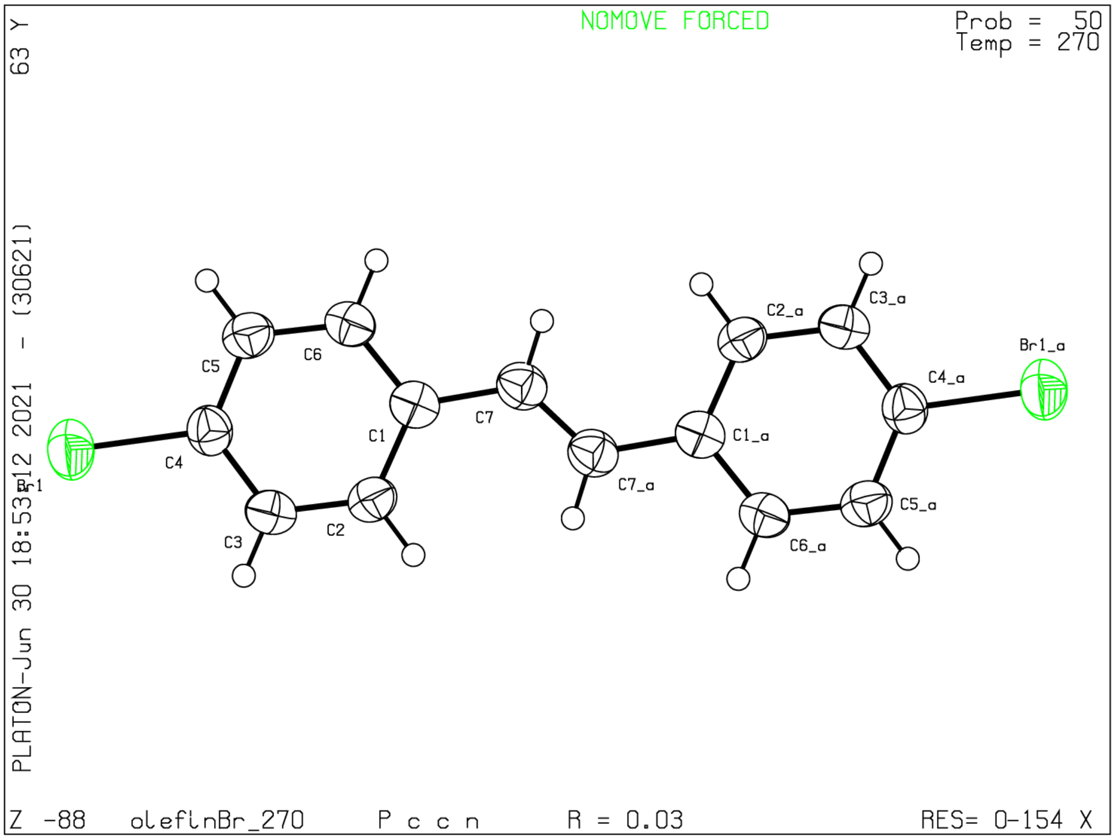

Datablock olefinBr_290 - ellipsoid plot

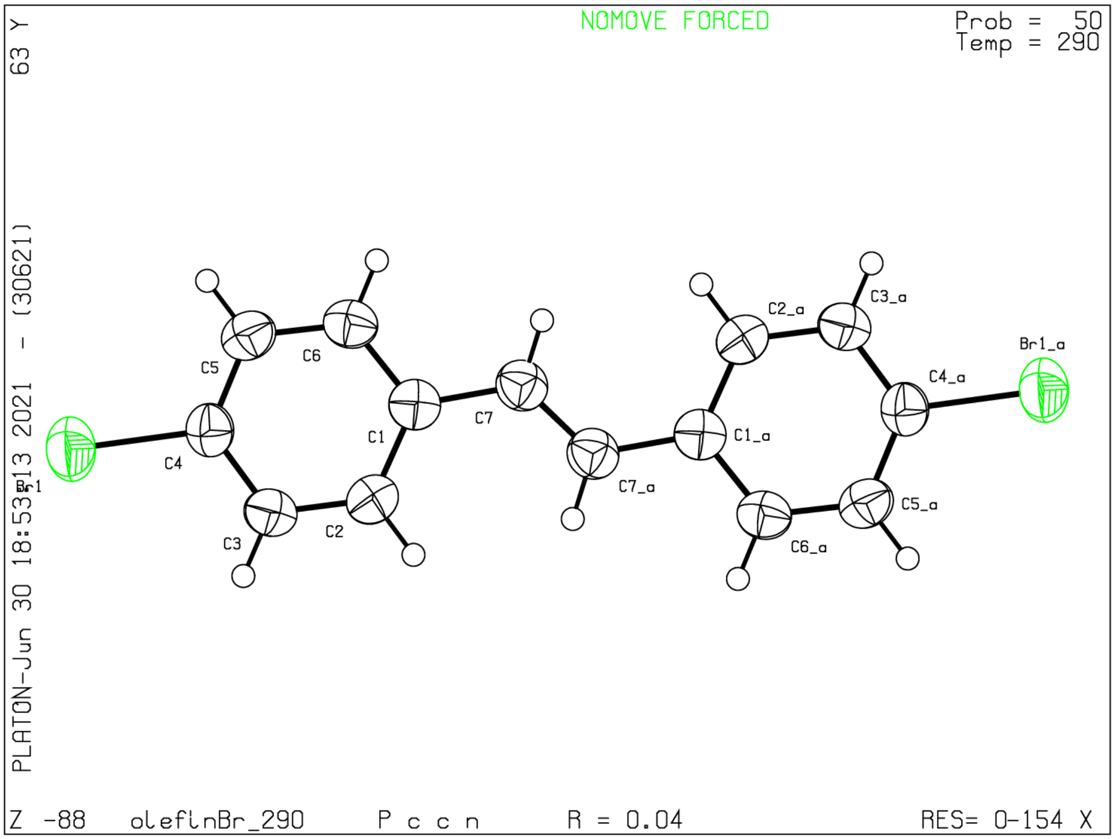

Supplement: Supplementary file 3 [file olefin-I + olefin-Br.pdf › olefin-I + olefin-Br.pdf]
