# Supplementary material for: Differences in thermal expansion and motion ability for herringbone and face-to-face π-stacked solids
Source: IUCrJ. 2021 Nov 3;9(Pt 1):31–42. doi: 10.1107/S2052252521009593 (PMC8733877; doi:10.1107/S2052252521009593)

checkCIF/PLATON report

Structure factors have been supplied for datablock(s) olefinIBr_190K, olefinIBr_210K, olefinIBr_230K, olefinIBr_250K, olefinIBr_270K, olefinIBr_290K

THIS REPORT IS FOR GUIDANCE ONLY. IF USED AS PART OF A REVIEW PROCEDURE FOR PUBLICATION, IT SHOULD NOT REPLACE THE EXPERTISE OF AN EXPERIENCED CRYSTALLOGRAPHIC REFEREE.

No syntax errors found. CIF dictionary Interpreting this report

Datablock: olefinIBr_190K

```
Bond precision:  C-C = 0.0059 A          Wavelength=1.54178

Cell:            a=7.36421(7)     b=28.3440(3)     c=5.95563(5)
                 alpha=90         beta=90          gamma=90
Temperature:     190 K

                Calculated                 Reported
Volume          1243.13(2)                 1243.13(2)
Space group     P c c n                    P c c n
Hall group      -P 2ab 2ac                 -P 2ab 2ac
Moiety formula  C14 H10 Br I               ?
Sum formula     C14 H10 Br I               C14 H10 Br I
Mr              385.02                     385.03
Dx,g cm-3       2.057                      2.057
Z               4                          4
Mu (mm-1)       23.735                     23.735
F000            728.0                      728.0
F000'           725.68
h,k,lmax        9,35,7                     9,35,7
Nref            1315                       1305
Tmin,Tmax       0.049,0.770                0.385,1.000
Tmin'           0.001

Correction method= # Reported T Limits: Tmin=0.385 Tmax=1.000
AbsCorr = GAUSSIAN

Data completeness= 0.992          Theta(max)= 77.304

R(reflections)= 0.0308( 1270)     wR2(reflections)= 0.0804( 1305)

S = 1.153                   Npar= 76
```

```
The following ALERTS were generated. Each ALERT has the format
       test-name_ALERT_alert-type_alert-level.
Click on the hyperlinks for more details of the test.
```

Alert level C

```
PLAT112_ALERT_2_C ADDSYM Detects New (Pseudo) Symm. Elem       b/2         85 %Fit
```

Author Response: Higher symmetry was checked using PLATON, but no higher space group was suggested.

```
PLAT906_ALERT_3_C Large K Value in the Analysis of Variance ......      2.550 Check
```

Author Response: Due to diffuse scattering about Bragg peaks that was not accounted for during the integration.

Alert level G

```
PLAT143_ALERT_4_G s.u. on c - Axis Small or Missing ..............    0.00005 Ang.
PLAT171_ALERT_4_G The CIF-Embedded .res File Contains EADP Records          1 Report
PLAT230_ALERT_2_G Hirshfeld Test Diff for    I1       --C1       .        7.5 s.u.
PLAT230_ALERT_2_G Hirshfeld Test Diff for    Br1      --C1       .        7.5 s.u.
PLAT300_ALERT_4_G Atom Site Occupancy of I1         Constrained at        0.5 Check
PLAT300_ALERT_4_G Atom Site Occupancy of Br1        Constrained at        0.5 Check
PLAT301_ALERT_3_G Main Residue  Disorder ..............(Resd  1  )        13% Note
PLAT779_ALERT_4_G Suspect or Irrelevant (Bond) Angle(s) in CIF ...       1.10 Deg.
              BR1  -C1   -I1      1_555   1_555   1_555 ......     #   24 Check
PLAT912_ALERT_4_G Missing # of FCF Reflections Above STh/L=  0.600          9 Note
PLAT913_ALERT_3_G Missing # of Very Strong Reflections in FCF ....          1 Note
PLAT978_ALERT_2_G Number C-C Bonds with Positive Residual Density.          4 Info
```

```
   0 ALERT level A = Most likely a serious problem - resolve or explain
   0 ALERT level B = A potentially serious problem, consider carefully
   2 ALERT level C = Check. Ensure it is not caused by an omission or oversight
  11 ALERT level G = General information/check it is not something unexpected

   0 ALERT type 1 CIF construction/syntax error, inconsistent or missing data
   4 ALERT type 2 Indicator that the structure model may be wrong or deficient
   3 ALERT type 3 Indicator that the structure quality may be low
   6 ALERT type 4 Improvement, methodology, query or suggestion
   0 ALERT type 5 Informative message, check
```

Datablock: olefinIBr_210K

```
Bond precision:   C-C = 0.0055 A          Wavelength=1.54178

Cell:             a=7.37950(7)      b=28.3436(3)      c=5.96456(5)
                  alpha=90          beta=90           gamma=90
Temperature:      210 K
```

	Calculated	Reported
Volume	1247.56(2)	1247.557(19)
Space group	P c c n	P c c n
Hall group	-P 2ab 2ac	-P 2ab 2ac
Moiety formula	C14 H10 Br I	?
Sum formula	C14 H10 Br I	C14 H10 Br I
Mr	385.02	385.03
Dx,g cm-3	2.050	2.050
Z	4	4
Mu (mm-1)	23.651	23.651
F000	728.0	728.0
F000’	725.68	
h,k,lmax	9,35,7	9,35,7
Nref	1320	1307
Tmin,Tmax	0.050,0.771	0.384,1.000
Tmin’	0.001	

Correction method= # Reported T Limits: Tmin=0.384 Tmax=1.000
AbsCorr = GAUSSIAN

Data completeness= 0.990 Theta(max)= 77.358

R(reflections)= 0.0297(1264) wR2(reflections)= 0.0797(1307)

S = 1.135 Npar= 82

The following ALERTS were generated. Each ALERT has the format
test-name_ALERT_alert-type_alert-level.
Click on the hyperlinks for more details of the test.

Alert level C

```
PLAT112_ALERT_2_C ADDSYM Detects New (Pseudo) Symm. Elem       b/2         85 %Fit
```

Author Response: Higher symmetry was checked using PLATON, but no higher space group was suggested.

Alert level G

```
PLAT003_ALERT_2_G Number of Uiso or Uij Restrained non-H Atoms ...          2 Report
PLAT143_ALERT_4_G s.u. on c - Axis Small or Missing .............     0.00005 Ang.
PLAT178_ALERT_4_G The CIF-Embedded .res File Contains SIMU Records          1 Report
PLAT230_ALERT_2_G Hirshfeld Test Diff for    I1       --C1       .       10.7 s.u.
PLAT230_ALERT_2_G Hirshfeld Test Diff for    Br1      --C1       .       14.0 s.u.
PLAT300_ALERT_4_G Atom Site Occupancy of I1        Constrained at         0.5 Check
PLAT300_ALERT_4_G Atom Site Occupancy of Br1       Constrained at         0.5 Check
PLAT301_ALERT_3_G Main Residue  Disorder ..............(Resd  1  )        13% Note
PLAT779_ALERT_4_G Suspect or Irrelevant (Bond) Angle(s) in CIF ...       0.70 Deg.
            BR1  -C1   -I1      1_555   1_555   1_555 ......     #   24 Check
PLAT860_ALERT_3_G Number of Least-Squares Restraints ............           6 Note
PLAT912_ALERT_4_G Missing # of FCF Reflections Above STh/L=  0.600         13 Note
```

```
PLAT913_ALERT_3_G Missing # of Very Strong Reflections in FCF ....          1 Note
PLAT978_ALERT_2_G Number C-C Bonds with Positive Residual Density.          4 Info
```

```
   0 ALERT level A = Most likely a serious problem - resolve or explain
   0 ALERT level B = A potentially serious problem, consider carefully
   1 ALERT level C = Check. Ensure it is not caused by an omission or oversight
  13 ALERT level G = General information/check it is not something unexpected

   0 ALERT type 1 CIF construction/syntax error, inconsistent or missing data
   5 ALERT type 2 Indicator that the structure model may be wrong or deficient
   3 ALERT type 3 Indicator that the structure quality may be low
   6 ALERT type 4 Improvement, methodology, query or suggestion
   0 ALERT type 5 Informative message, check
```

Datablock: olefinIBr_230K

```
Bond precision:  C-C = 0.0055 A          Wavelength=1.54178

Cell:            a=7.39554(8)     b=28.3456(3)     c=5.97312(5)
                 alpha=90         beta=90          gamma=90
Temperature:     230 K
```

	Calculated	Reported
Volume	1252.15(2)	1252.15(2)
Space group	P c c n	P c c n
Hall group	-P 2ab 2ac	-P 2ab 2ac
Moiety formula	C14 H10 Br I	?
Sum formula	C14 H10 Br I	C14 H10 Br I
Mr	385.02	385.03
Dx,g cm-3	2.042	2.042
Z	4	4
Mu (mm-1)	23.564	23.564
F000	728.0	728.0
F000’	725.68	
h,k,lmax	9,35,7	9,35,7
Nref	1329	1313
Tmin,Tmax	0.050,0.772	0.383,1.000
Tmin’	0.001	

```
Correction method= # Reported T Limits: Tmin=0.383 Tmax=1.000
AbsCorr = GAUSSIAN

Data completeness= 0.988          Theta(max)= 77.318

R(reflections)= 0.0297( 1265)     wR2(reflections)= 0.0815( 1313)

S = 1.105                   Npar= 82
```

The following ALERTS were generated. Each ALERT has the format
test-name_ALERT_alert-type_alert-level.
Click on the hyperlinks for more details of the test.

Alert level C

```
PLAT112_ALERT_2_C ADDSYM Detects New (Pseudo) Symm. Elem       b/2         85 %Fit
```

Author Response: Higher symmetry was checked using PLATON, but no higher space group was suggested.

```
PLAT911_ALERT_3_C Missing FCF Refl Between Thmin & STh/L=    0.600           2 Report
```

Alert level G

```
PLAT003_ALERT_2_G Number of Uiso or Uij Restrained non-H Atoms ...           2 Report
PLAT143_ALERT_4_G s.u. on c - Axis Small or Missing .............    0.00005 Ang.
PLAT178_ALERT_4_G The CIF-Embedded .res File Contains SIMU Records           1 Report
PLAT187_ALERT_4_G The CIF-Embedded .res File Contains RIGU Records           1 Report
PLAT230_ALERT_2_G Hirshfeld Test Diff for    I1       --C1       .       10.4 s.u.
PLAT230_ALERT_2_G Hirshfeld Test Diff for    Br1      --C1       .       12.7 s.u.
PLAT300_ALERT_4_G Atom Site Occupancy of I1         Constrained at        0.5 Check
PLAT300_ALERT_4_G Atom Site Occupancy of Br1        Constrained at        0.5 Check
PLAT301_ALERT_3_G Main Residue  Disorder ..............(Resd  1  )        13% Note
PLAT779_ALERT_4_G Suspect or Irrelevant (Bond) Angle(s) in CIF ...       2.00 Deg.
              BR1  -C1   -I1      1_555   1_555   1_555 ......     #   24 Check
PLAT860_ALERT_3_G Number of Least-Squares Restraints ............          65 Note
PLAT912_ALERT_4_G Missing # of FCF Reflections Above STh/L=  0.600         14 Note
PLAT913_ALERT_3_G Missing # of Very Strong Reflections in FCF ....          1 Note
PLAT933_ALERT_2_G Number of OMIT Records in Embedded .res File ...          1 Note
PLAT978_ALERT_2_G Number C-C Bonds with Positive Residual Density.          3 Info
```

```
   0 ALERT level A = Most likely a serious problem - resolve or explain
   0 ALERT level B = A potentially serious problem, consider carefully
   2 ALERT level C = Check. Ensure it is not caused by an omission or oversight
  15 ALERT level G = General information/check it is not something unexpected

   0 ALERT type 1 CIF construction/syntax error, inconsistent or missing data
   6 ALERT type 2 Indicator that the structure model may be wrong or deficient
   4 ALERT type 3 Indicator that the structure quality may be low
   7 ALERT type 4 Improvement, methodology, query or suggestion
   0 ALERT type 5 Informative message, check
```

Datablock: olefinIBr_250K

```
Bond precision:  C-C = 0.0055 A          Wavelength=1.54178

Cell:            a=7.41258(8)      b=28.3456(3)      c=5.98174(5)
                 alpha=90          beta=90           gamma=90
Temperature:     250 K
```

	Calculated	Reported
Volume	1256.85(2)	1256.85(2)
Space group	P c c n	P c c n
Hall group	-P 2ab 2ac	-P 2ab 2ac
Moiety formula	C14 H10 Br I	?
Sum formula	C14 H10 Br I	C14 H10 Br I
Mr	385.02	385.03
Dx,g cm-3	2.035	2.035
Z	4	4
Mu (mm-1)	23.476	23.476
F000	728.0	728.0
F000'	725.68	
h,k,lmax	9,35,7	9,35,7
Nref	1336	1319
Tmin,Tmax	0.051,0.772	0.383,1.000
Tmin'	0.001	

Correction method= # Reported T Limits: Tmin=0.383 Tmax=1.000
AbsCorr = GAUSSIAN

Data completeness= 0.987 Theta(max)= 77.433

R(reflections)= 0.0299(1258) wR2(reflections)= 0.0840(1319)

S = 1.101 Npar= 82

The following ALERTS were generated. Each ALERT has the format
test-name_ALERT_alert-type_alert-level.
Click on the hyperlinks for more details of the test.

Alert level C

PLAT112_ALERT_2_C ADDSYM Detects New (Pseudo) Symm. Elem b/2 85 %Fit

Author Response: Higher symmetry was checked using PLATON, but no higher space group was suggested.

PLAT911_ALERT_3_C Missing FCF Refl Between Thmin & STh/L= 0.600 2 Report

---

Alert level G

```
PLAT003_ALERT_2_G Number of Uiso or Uij Restrained non-H Atoms ...          2 Report
PLAT143_ALERT_4_G s.u. on c - Axis Small or Missing .............     0.00005 Ang.
PLAT178_ALERT_4_G The CIF-Embedded .res File Contains SIMU Records          1 Report
PLAT187_ALERT_4_G The CIF-Embedded .res File Contains RIGU Records          1 Report
PLAT230_ALERT_2_G Hirshfeld Test Diff for    I1       --C1       .       10.3 s.u.
PLAT230_ALERT_2_G Hirshfeld Test Diff for    Br1      --C1       .       11.0 s.u.
PLAT300_ALERT_4_G Atom Site Occupancy of I1        Constrained at         0.5 Check
PLAT300_ALERT_4_G Atom Site Occupancy of Br1       Constrained at         0.5 Check
PLAT301_ALERT_3_G Main Residue  Disorder .............(Resd  1  )         13% Note
PLAT779_ALERT_4_G Suspect or Irrelevant (Bond) Angle(s) in CIF ...       3.00 Deg.
              BR1  -C1   -I1      1_555   1_555   1_555 ......     #   24 Check
```

```
PLAT860_ALERT_3_G Number of Least-Squares Restraints .............         65 Note
PLAT912_ALERT_4_G Missing # of FCF Reflections Above STh/L=  0.600         16 Note
PLAT913_ALERT_3_G Missing # of Very Strong Reflections in FCF ....          1 Note
PLAT933_ALERT_2_G Number of OMIT Records in Embedded .res File ...          1 Note
PLAT978_ALERT_2_G Number C-C Bonds with Positive Residual Density.          4 Info
```

---

0 **ALERT level A** = Most likely a serious problem - resolve or explain
0 **ALERT level B** = A potentially serious problem, consider carefully
2 **ALERT level C** = Check. Ensure it is not caused by an omission or oversight
15 **ALERT level G** = General information/check it is not something unexpected

0 ALERT type 1 CIF construction/syntax error, inconsistent or missing data
6 ALERT type 2 Indicator that the structure model may be wrong or deficient
4 ALERT type 3 Indicator that the structure quality may be low
7 ALERT type 4 Improvement, methodology, query or suggestion
0 ALERT type 5 Informative message, check

Datablock: olefinIBr_270K

Bond precision: C-C = 0.0055 A Wavelength=1.54178

Cell:	a=7.42999(8)	b=28.3468(3)	c=5.99099(5)
	alpha=90	beta=90	gamma=90
Temperature:	270 K		

	Calculated	Reported
Volume	1261.80(2)	1261.80(2)
Space group	P c c n	P c c n
Hall group	-P 2ab 2ac	-P 2ab 2ac
Moiety formula	C14 H10 Br I	?
Sum formula	C14 H10 Br I	C14 H10 Br I
Mr	385.02	385.03
Dx,g cm-3	2.027	2.027
Z	4	4
Mu (mm-1)	23.384	23.384
F000	728.0	728.0
F000’	725.68	
h,k,lmax	9,35,7	9,35,7
Nref	1338	1326
Tmin,Tmax	0.052,0.773	0.384,1.000
Tmin’	0.001	

Correction method= # Reported T Limits: Tmin=0.384 Tmax=1.000
AbsCorr = GAUSSIAN

Data completeness= 0.991 Theta(max)= 77.298

R(reflections)= 0.0306(1259) wR2(reflections)= 0.0837(1326)

S = 1.099 Npar= 82

The following ALERTS were generated. Each ALERT has the format
test-name_ALERT_alert-type_alert-level.
Click on the hyperlinks for more details of the test.

Alert level C

```
PLAT112_ALERT_2_C ADDSYM Detects New (Pseudo) Symm. Elem       b/2         85 %Fit
```

Author Response: Higher symmetry was checked using PLATON, but no higher space group was suggested.

```
PLAT911_ALERT_3_C Missing FCF Refl Between Thmin & STh/L=    0.600          2 Report
```

Alert level G

```
PLAT003_ALERT_2_G Number of Uiso or Uij Restrained non-H Atoms ...          2 Report
PLAT143_ALERT_4_G s.u. on c - Axis Small or Missing .............    0.00005 Ang.
PLAT178_ALERT_4_G The CIF-Embedded .res File Contains SIMU Records          1 Report
PLAT187_ALERT_4_G The CIF-Embedded .res File Contains RIGU Records          1 Report
PLAT230_ALERT_2_G Hirshfeld Test Diff for    Br1      --C1       .        5.5 s.u.
PLAT300_ALERT_4_G Atom Site Occupancy of I1         Constrained at        0.5 Check
PLAT300_ALERT_4_G Atom Site Occupancy of Br1        Constrained at        0.5 Check
PLAT301_ALERT_3_G Main Residue  Disorder ..............(Resd  1  )        13% Note
PLAT779_ALERT_4_G Suspect or Irrelevant (Bond) Angle(s) in CIF ...       6.50 Deg.
              BR1  -C1   -I1      1_555   1_555   1_555 ......     #   24 Check
PLAT860_ALERT_3_G Number of Least-Squares Restraints ............          65 Note
PLAT912_ALERT_4_G Missing # of FCF Reflections Above STh/L=  0.600         11 Note
PLAT913_ALERT_3_G Missing # of Very Strong Reflections in FCF ....          1 Note
PLAT933_ALERT_2_G Number of OMIT Records in Embedded .res File ...          1 Note
PLAT978_ALERT_2_G Number C-C Bonds with Positive Residual Density.          4 Info
```

---

0 **ALERT level A** = Most likely a serious problem - resolve or explain
0 **ALERT level B** = A potentially serious problem, consider carefully
2 **ALERT level C** = Check. Ensure it is not caused by an omission or oversight
14 **ALERT level G** = General information/check it is not something unexpected

0 ALERT type 1 CIF construction/syntax error, inconsistent or missing data
5 ALERT type 2 Indicator that the structure model may be wrong or deficient
4 ALERT type 3 Indicator that the structure quality may be low
7 ALERT type 4 Improvement, methodology, query or suggestion
0 ALERT type 5 Informative message, check

Datablock: olefinIBr_290K

Bond precision: C-C = 0.0058 A Wavelength=1.54178

Cell:	a=7.44877(12)	b=28.3440(4)	c=6.00033(8)
	alpha=90	beta=90	gamma=90
Temperature:	290 K		

	Calculated	Reported
Volume	1266.84(3)	1266.84(3)
Space group	P c c n	P c c n
Hall group	-P 2ab 2ac	-P 2ab 2ac
Moiety formula	C14 H10 Br I	?
Sum formula	C14 H10 Br I	C14 H10 Br I
Mr	385.02	385.03
Dx,g cm-3	2.019	2.019
Z	4	4
Mu (mm-1)	23.291	23.291
F000	728.0	728.0
F000’	725.68	
h,k,lmax	9,35,7	9,35,7
Nref	1346	1330
Tmin,Tmax	0.053,0.774	0.384,1.000
Tmin’	0.001	

Correction method= # Reported T Limits: Tmin=0.384 Tmax=1.000
AbsCorr = GAUSSIAN

Data completeness= 0.988   Theta(max)= 77.375

R(reflections)= 0.0312(1232)   wR2(reflections)= 0.0838(1330)

S = 1.125   Npar= 82

The following ALERTS were generated. Each ALERT has the format
test-name_ALERT_alert-type_alert-level.
Click on the hyperlinks for more details of the test.

Alert level C

```
PLAT112_ALERT_2_C ADDSYM Detects New (Pseudo) Symm. Elem       b/2         85 %Fit
```

Author Response: Higher symmetry was checked using PLATON, but no higher space group was suggested.

```
PLAT911_ALERT_3_C Missing FCF Refl Between Thmin & STh/L=    0.600           2 Report
```

Alert level G

```
PLAT003_ALERT_2_G Number of Uiso or Uij Restrained non-H Atoms ...           2 Report
PLAT178_ALERT_4_G The CIF-Embedded .res File Contains SIMU Records           1 Report
PLAT187_ALERT_4_G The CIF-Embedded .res File Contains RIGU Records           1 Report
PLAT300_ALERT_4_G Atom Site Occupancy of I1         Constrained at         0.5 Check
PLAT300_ALERT_4_G Atom Site Occupancy of Br1        Constrained at         0.5 Check
PLAT301_ALERT_3_G Main Residue  Disorder ..............(Resd  1  )        13% Note
PLAT779_ALERT_4_G Suspect or Irrelevant (Bond) Angle(s) in CIF ...       5.30 Deg.
              BR1  -C1   -I1      1_555   1_555   1_555 ......     #   24 Check
PLAT860_ALERT_3_G Number of Least-Squares Restraints ............          65 Note
PLAT912_ALERT_4_G Missing # of FCF Reflections Above STh/L=  0.600          14 Note
PLAT933_ALERT_2_G Number of OMIT Records in Embedded .res File ...           1 Note
```

```
PLAT978_ALERT_2_G Number C-C Bonds with Positive Residual Density.          2 Info
PLAT992_ALERT_5_G Repd & Actual _reflns_number_gt Values Differ by          3 Check
```

```
   0 ALERT level A = Most likely a serious problem - resolve or explain
   0 ALERT level B = A potentially serious problem, consider carefully
   2 ALERT level C = Check. Ensure it is not caused by an omission or oversight
  12 ALERT level G = General information/check it is not something unexpected

   0 ALERT type 1 CIF construction/syntax error, inconsistent or missing data
   4 ALERT type 2 Indicator that the structure model may be wrong or deficient
   3 ALERT type 3 Indicator that the structure quality may be low
   6 ALERT type 4 Improvement, methodology, query or suggestion
   1 ALERT type 5 Informative message, check
```

Validation response form

Please find below a validation response form (VRF) that can be filled in and pasted into your CIF.

```
# start Validation Reply Form
_vrf_PLAT911_olefinIBr_230K
;
PROBLEM: Missing FCF Refl Between Thmin & STh/L=    0.600          2 Report
RESPONSE: ...
;
_vrf_PLAT911_olefinIBr_250K
;
PROBLEM: Missing FCF Refl Between Thmin & STh/L=    0.600          2 Report
RESPONSE: ...
;
_vrf_PLAT911_olefinIBr_270K
;
PROBLEM: Missing FCF Refl Between Thmin & STh/L=    0.600          2 Report
RESPONSE: ...
;
_vrf_PLAT911_olefinIBr_290K
;
PROBLEM: Missing FCF Refl Between Thmin & STh/L=    0.600          2 Report
RESPONSE: ...
;
# end Validation Reply Form
```

It is advisable to attempt to resolve as many as possible of the alerts in all categories. Often the minor alerts point to easily fixed oversights, errors and omissions in your CIF or refinement strategy, so attention to these fine details can be worthwhile. In order to resolve some of the more serious problems it may be necessary to carry out additional measurements or structure refinements. However, the purpose of your study may justify the reported deviations and the more serious of these should normally be commented upon in the discussion or experimental section of a paper or in the "special_details" fields of the CIF. checkCIF was carefully designed to identify outliers and unusual parameters, but every test has its limitations and alerts that are not important in a particular case may appear. Conversely, the absence of alerts does not guarantee there are no aspects of the results needing attention. It is up to the individual to critically assess their own results and, if necessary, seek expert advice.

Publication of your CIF in IUCr journals

A basic structural check has been run on your CIF. These basic checks will be run on all CIFs submitted for publication in IUCr journals (*Acta Crystallographica*, *Journal of Applied Crystallography*, *Journal of Synchrotron Radiation*); however, if you intend to submit to *Acta Crystallographica Section C* or *E* or *IUCrData*, you should make sure that full publication checks are run on the final version of your CIF prior to submission.

Publication of your CIF in other journals

Please refer to the *Notes for Authors* of the relevant journal for any special instructions relating to CIF submission.

PLATON version of 03/06/2021; check.def file version of 02/06/2021

Datablock olefinIBr_190K - ellipsoid plot

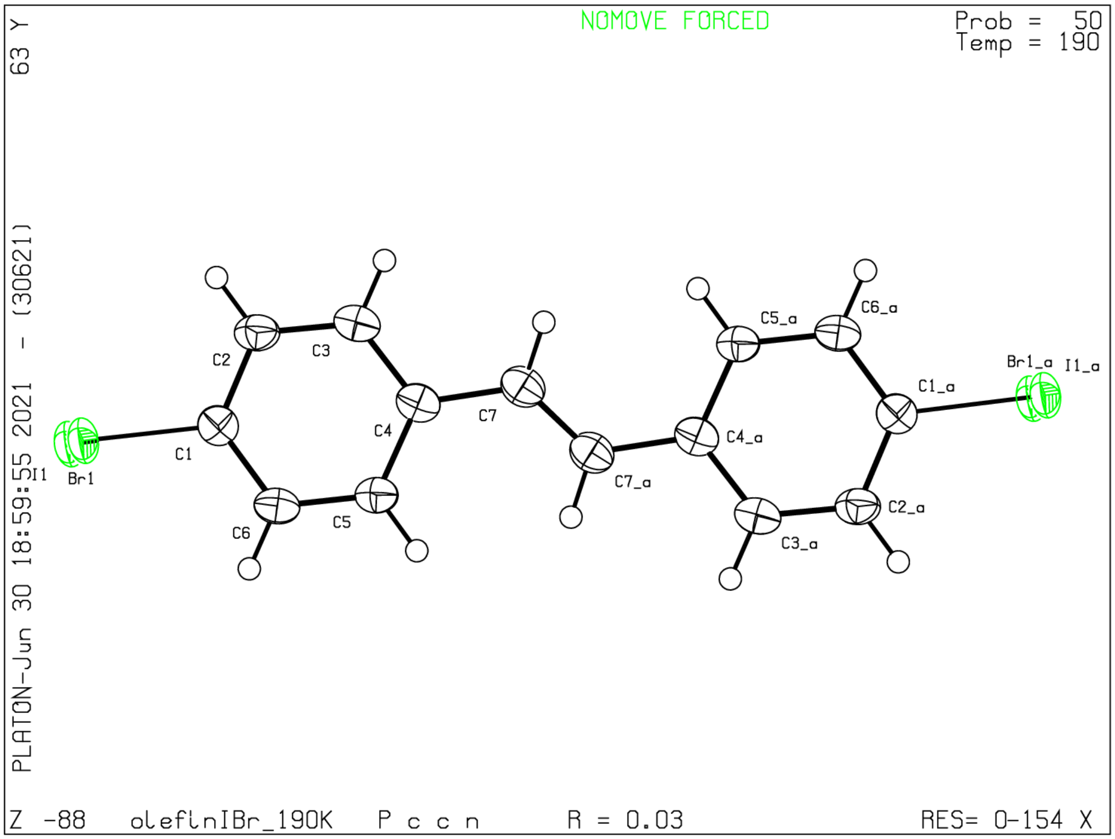

Datablock olefinIBr_210K - ellipsoid plot

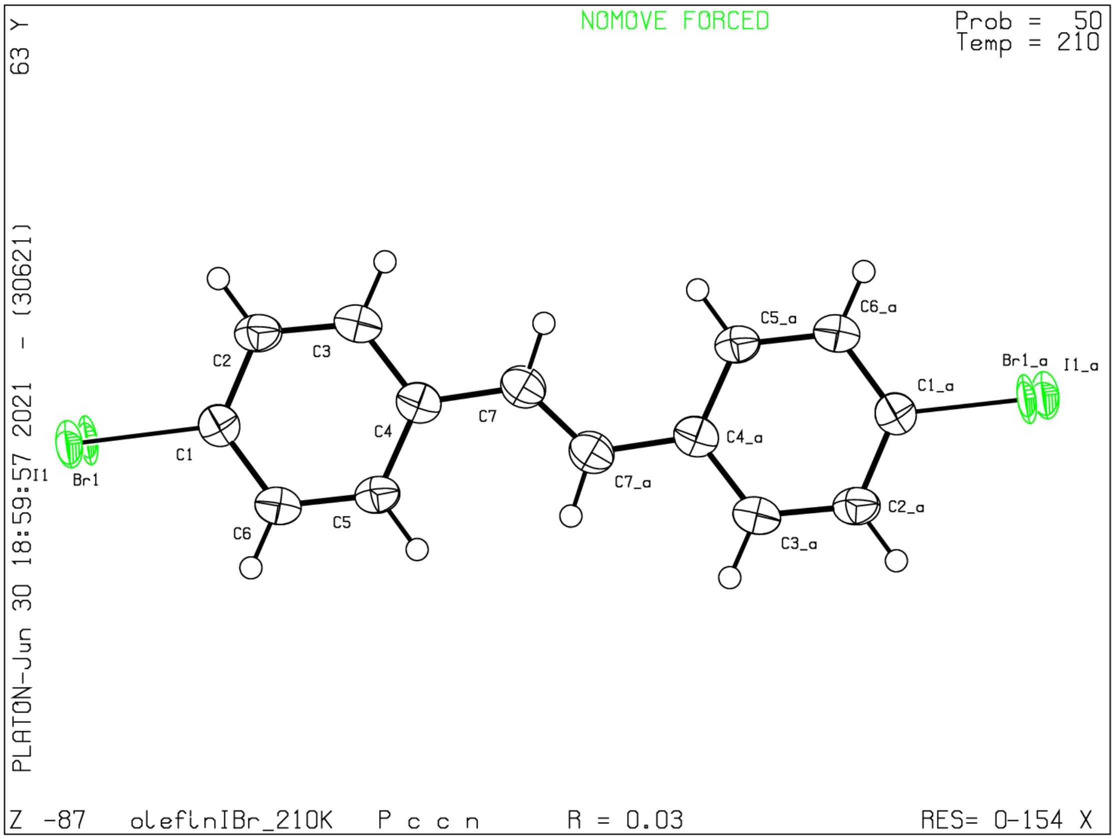

Datablock olefinIBr_230K - ellipsoid plot

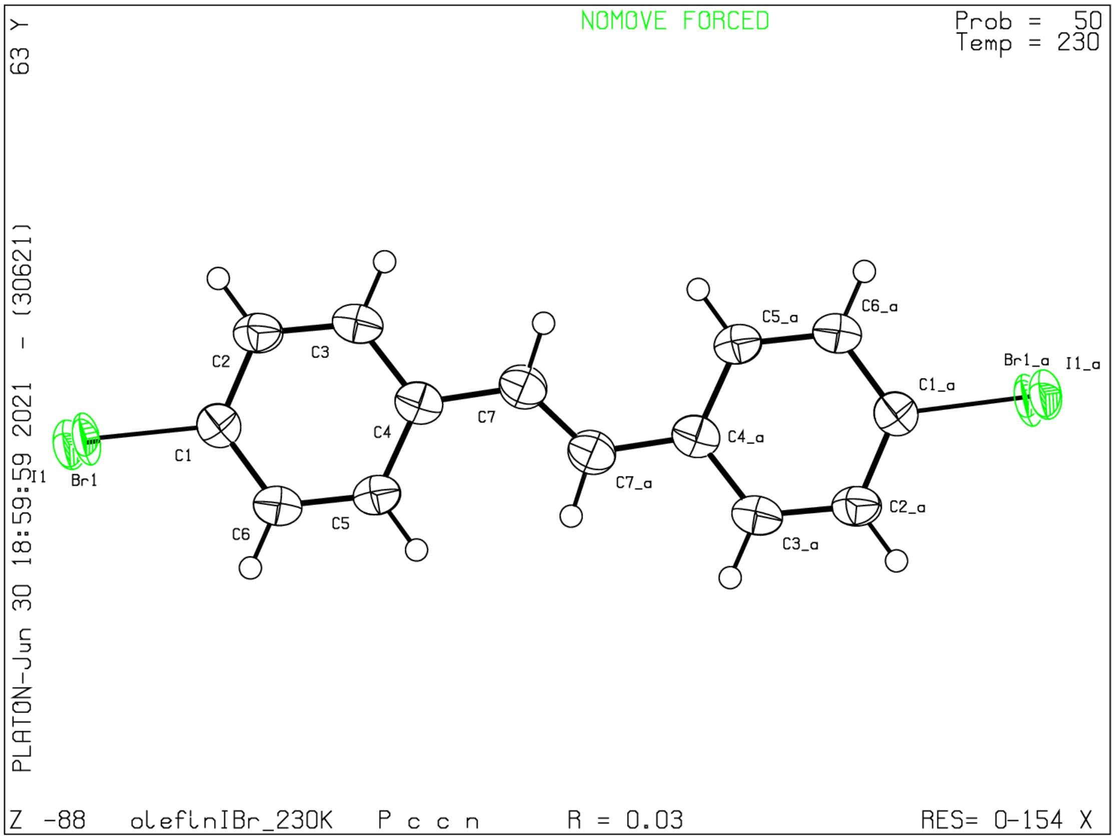

Datablock olefinIBr_250K - ellipsoid plot

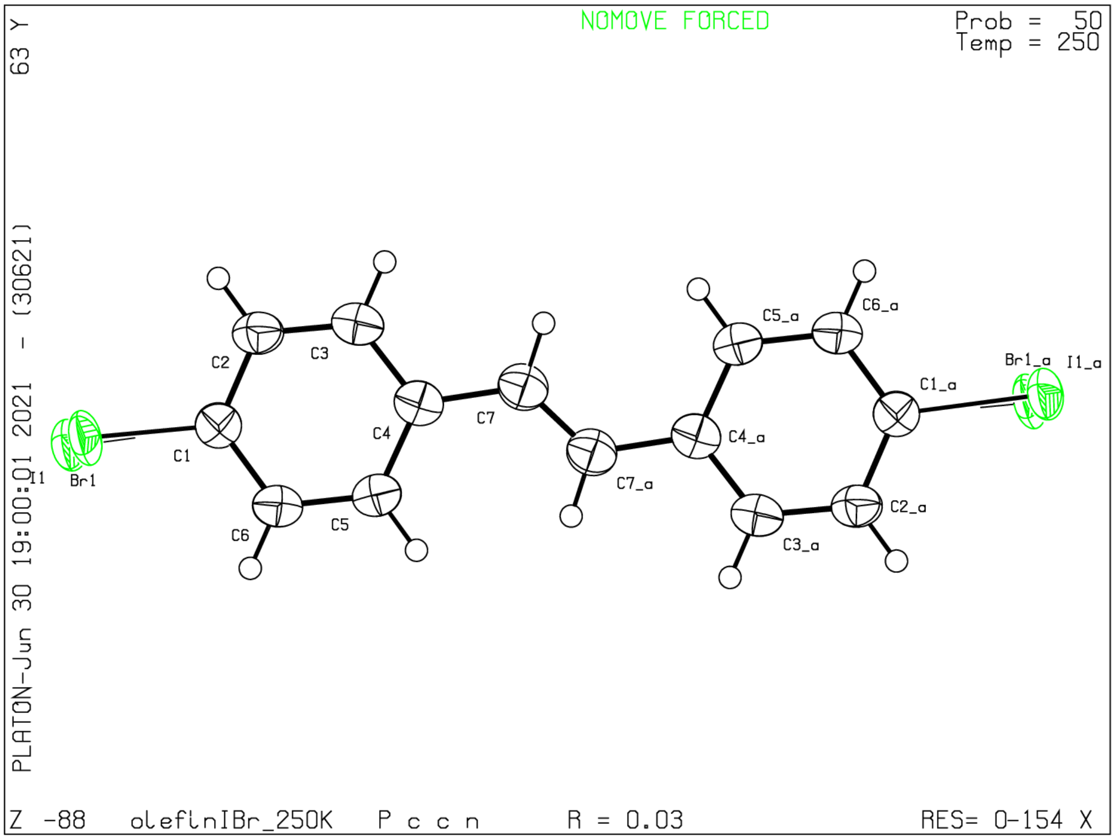

Datablock olefinIBr_270K - ellipsoid plot

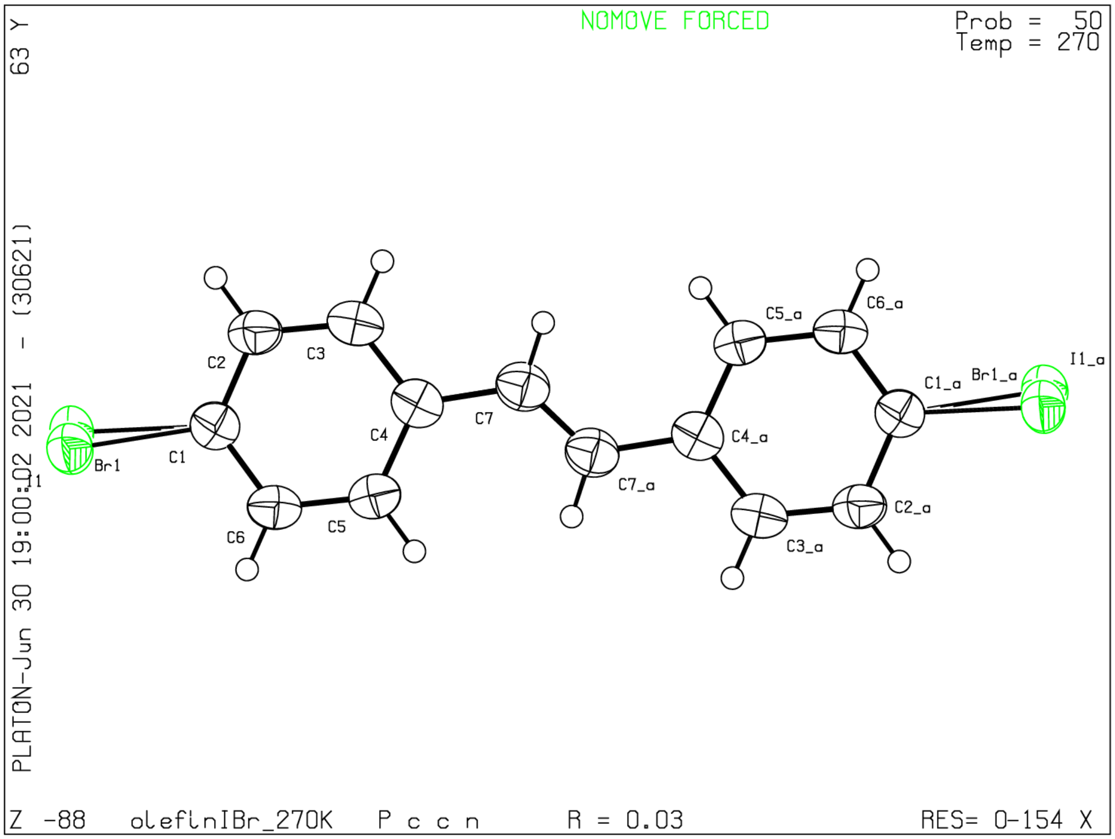

Datablock olefinIBr_290K - ellipsoid plot

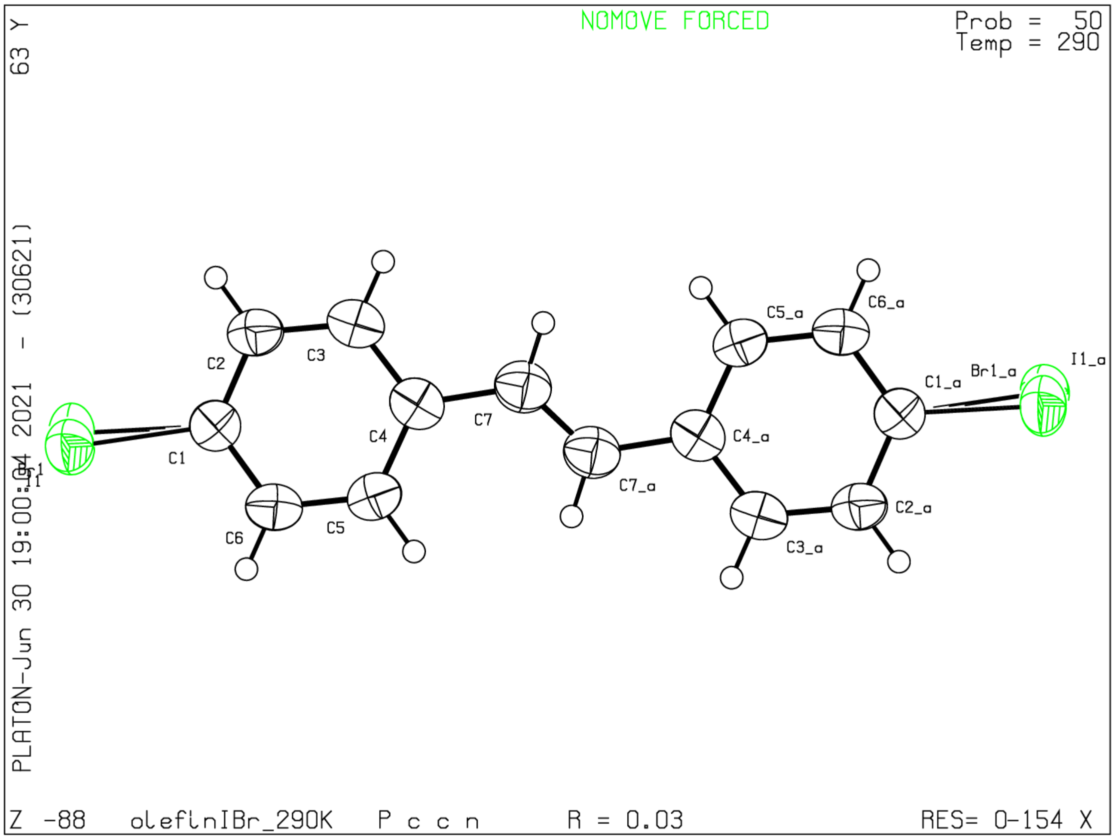

Supplement: Supplementary file 3 [file olefin-I Br.pdf › olefin-I Br.pdf]
